# Modular Synthesis of α,α-Diaryl α-Amino Esters via Bi(V)-Mediated Arylation/S_N_2-Displacement of Kukhtin–Ramirez Intermediates

**DOI:** 10.1021/acs.orglett.2c03201

**Published:** 2022-10-24

**Authors:** Alessio Calcatelli, Ross M. Denton, Liam T. Ball

**Affiliations:** † School of Chemistry, University of Nottingham, Nottingham NG7 2RD, U.K.; ‡ GlaxoSmithKline Carbon Neutral Laboratories for Sustainable Chemistry, University of Nottingham, 6 Triumph Road, Nottingham NG7 2GA, U.K.

## Abstract

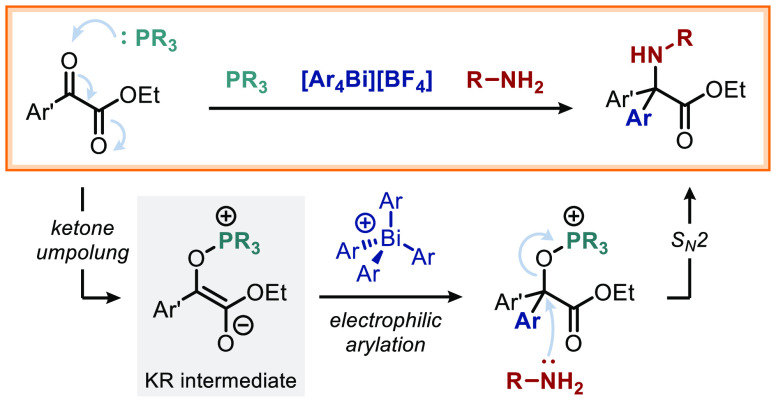

We report a concise and modular approach to α,α-diaryl α-amino esters from readily available α-keto esters. This mild, one-pot protocol proceeds via ketone umpolung, with *in situ* formation of a Kukhtin–Ramirez intermediate preceding sequential electrophilic arylation by Bi(V) and S_N_2 displacement by an amine. The methodology is compatible with a wide range of anilines and primary amines - including derivatives of drugs and proteinogenic amino acids - Bi(V) arylating agents, and α-keto ester substrates.

Quaternary amino acids possess enhanced chemical and metabolic stability, greater lipophilicity, and distinct conformational preferences relative to the canonical amino acids.^1−4^ As such, quaternary amino acids have been used widely in the investigation of peptide structure and biological activity, the rational design of foldamers,^1^ and the development of peptoid-based therapeutics.^5,6^ While α,α-dialkyl substitution is typically associated with the nucleation and stabilization of a helical secondary structure,^1,7,8^ α,α-diaryl amino acids exhibit a context-dependent conformational preference that often favors extended geometries.^9−13^ The complementary conformational properties and chemical characters (*i.e*., lipophilic vs aromatic) of these two classes of quaternary amino acids makes them individually valuable to peptide design and, therefore, important targets for chemical synthesis.

α,α-Diaryl amino acids can be disconnected via any one of the four bonds to the quaternary center. Despite the diversity of approaches—which include amination,^14−18^ arylation,^19−31^ and carboxylation^32^ with both electrophilic and nucleophilic reagents—the vast majority are united by a common feature: only one bond to the quaternary center is disconnected in each retrosynthetic operation. The synthesis of α,α-diaryl amino acids therefore typically requires multiple steps, which ultimately reduces their efficiency. Strategies that simultaneously disconnect two or more bonds to the quaternary center are currently limited to Greaney’s tandem S_N_2/Smiles arylation methodology (Scheme 1A, top).^33^ While an important conceptual advance, the scope and practicality of this approach are inherently limited by the need for electron-poor aryl partners, and the fact that both the aryl and the nitrogen components originate from a single sulfonamide precursor. There thus remains an unmet need for a more general and convergent route to α,α-diaryl amino acids in which each fragment is derived from separate sources.

**Scheme 1:**
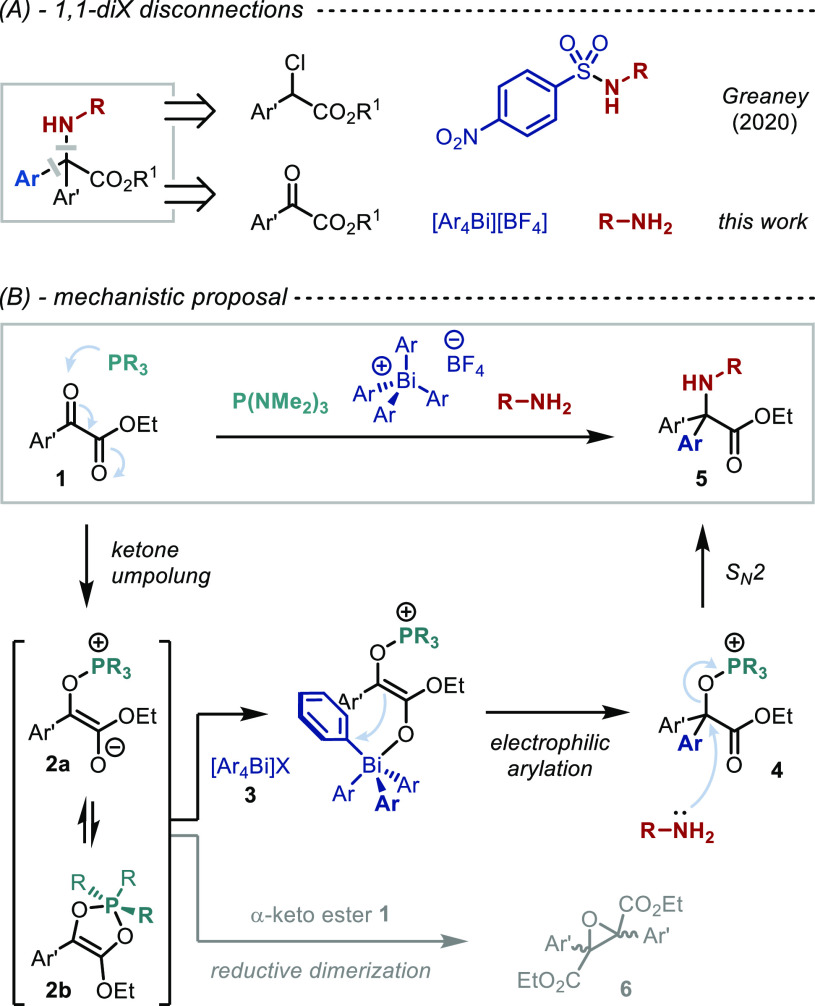
Strategies for the Synthesis of α,α-Diaryl Amino Esters

Herein we demonstrate a novel two-bond disconnection that allows for the modular assembly of α,α-diaryl α-amino acids in a single step from independently variable reagents (Scheme 1A, bottom). Installation of diverse aryl and amino components at the quaternary center is achieved via net deoxygenation of an α-keto ester core. The methodology proceeds under mild conditions and tolerates synthetically valuable functionality, such as esters, alkenes, alkynes, and aryl bromides.

We recognized that the formal polarity inversion of α-keto esters **1** can be achieved by addition of a nucleophilic phosphine to form the corresponding Kukhtin–Ramirez (KR) intermediate **2** (Scheme 1B).^34^ While interception of KR-type intermediates has been demonstrated with numerous electrophiles,^34−38^ electrophilic arylation has never been reported. We envisaged that **2a**, the phosphonium enolate isomer of the KR intermediate, would react with tetraarylbismuthonium reagents **3**(39−42) to form electrophilic alkoxyphosphonium salt **4**. Subsequent displacement of phosphine oxide by a nitrogen nucleophile would complete the sequence, ultimately providing concise and modular access to the desired α,α-diaryl α-amino acid derivative **5**. While S_N_2 reactions at quaternary centers are typically very rare, such substitutions α to esters are facilitated by the planar and electron-withdrawing nature of the adjacent carbonyl moiety.^43−48^

Overall, this strategy introduces the aryl group and the amine moiety in the same synthetic step, but from different sources and without the need of prefunctionalization of the starting materials. Crucial to success is (1) avoiding competitive reductive dimerization of the α-keto ester that affords epoxide **6**, (2) use of a Bi(V) reagent with a non-nucleophilic counterion to avoid competitive attack on **4**, and (3) chemo-compatibility between a nucleophilic amine and an electrophilic Bi(V) species.

Key steps toward the implementation of the proposed three-component amino arylation reaction are summarized in Table 1. Ultimately, rigorous optimization (see the Supporting Information) revealed that dropwise addition of P(NMe_2_)_3_ over 1 min to a solution of α-keto ester **1a**, tetraarylbismuthonium salt **3a**, and aniline **7** at 0 °C, followed by stirring for 16 h at room temperature, gave the amino-arylated product **8** in reproducibly high yields (Table 1, entry 1).

**Table 1:**
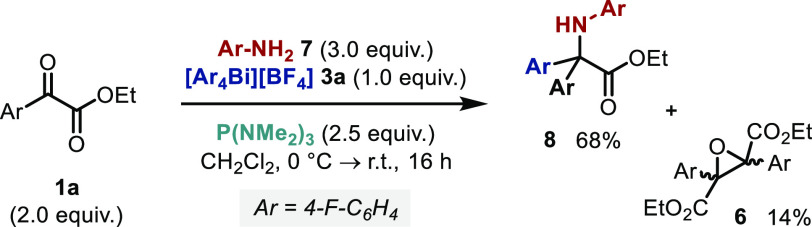
Summary of Reaction Optimization^a^

Entry	Deviation from Above	% **8**	% **6**
1	equiv: **1a** (1.5), **7** (1.5), **3a** (1.0), P(NMe_2_)_3_ (2.0)	80	3
2	PPh_3_/P(*n*-Bu)_3_/P(OEt)_3_	<5	0
3	no phosphine	<5	0
4	–78 °C → rt	64	18
5	rt	70	3
6	40 °C	61	5
7	THF	56	7
8	toluene	<5	0
9	**1a** added last	35	6
10	**1a** added over 1 h, [**3a**]_0_ = 0.2 M	<5	0
11	P(NMe_2_)_3_ added over 1 h	<5	0

a Reactions performed on a 0.1 mmol scale using anhydrous CH_2_Cl_2_ ([**3a**]_0_ = 0.1 M). Yields determined by ^19^F NMR spectroscopic analysis vs internal standard (PhCF_3_); yields of epoxide **6** are calculated relative to α-keto ester **1a**.

Neither PPh_3_, P(*n*-Bu)_3_, nor P(OEt)_3_ (Table 1, entry 2) proved to be a competent alternative to P(NMe_2_)_3_. This is consistent with Ramirez’s observation that formation of the oxyphosphonium enolate **2a** (Scheme 1B) is favored for phosphines that are sterically bulky and highly nucleophilic, such as P(NMe_2_)_3_,^49−51,37^ whereas smaller phosphines favor formation of phospholene **2b** while less nucleophilic phosphines are unreactive.^50,52,53^ Importantly, a control reaction demonstrated the key role of the phosphine in promoting the desired reactivity (entry 3).

Although the yield of amino acid **8** decreased significantly when P(NMe_2_)_3_ was added at either lower or higher temperatures, a comparable yield was obtained at room temperature (Table 1, entries 4–6). Furthermore, an assessment of different solvents revealed that relatively polar media are required (entries 7 and 8). The observed sensitivity of the reaction may reflect the fact that the distribution of the KR intermediate between the isomeric phosphonium enolate **2a** and dioxaphospholene **2b** forms (Scheme 1B) depends strongly on reaction conditions.^54−56^ Indeed, detailed VT-NMR studies have shown a delicate correlation between the speciation of KR intermediates—which ultimately controls product distribution—and the properties of the phosphine, solvent, and temperature.^57^

To suppress the formation of epoxide **6**,^38,37,57^ which was consistently observed as a side product during optimization, the order and rate of reagent addition was investigated. However, the yield of amino acid **8** was significantly reduced when α-keto ester **1a** was added as the last component, or when either **1a** or P(NMe_2_)_3_ were added over 1 h (entries 9–11). In contrast, the use of more economic stoichiometries of **1a**, **7**, and P(NMe_2_)_3_ (added over 1 min) afforded amino acid **8** in high yield and reduced the formation of epoxide **6** to <5% (entry 1).

Having identified suitable conditions, the scope and limitations of the methodology were investigated (Scheme 2). Variation of the amine nucleophile revealed that electronically (**8**–**14**) and sterically (**15**) diverse anilines afford the corresponding amino acids in high yields (Scheme 2A). The reaction is compatible with synthetically versatile aryl bromides (**13**) and potentially sensitive functional groups such as esters (**11**) and terminal alkynes (**14**).

**Scheme 2:**
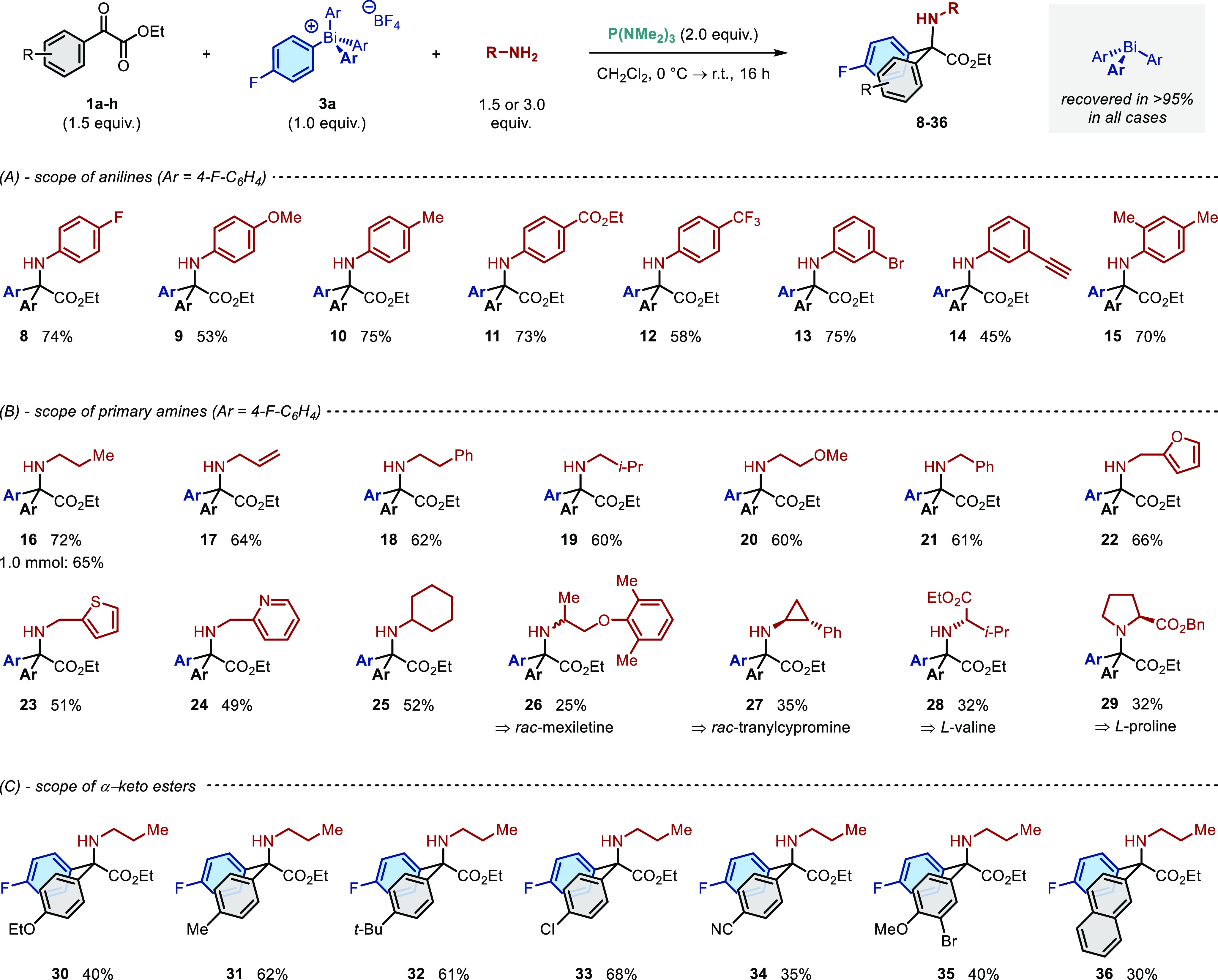
Scope with Respect to Amine Nucleophile and α-Keto Ester Substrate Reactions performed on a 0.5 mmol scale using anhydrous CH_2_Cl_2_ ([**3a**]_0_ = 0.1 M) and either 1.5 equiv of aniline nucleophile or 3.0 equiv of aliphatic amine nucleophile. Yields refer to material isolated after purification.

In all cases, the triarylbismuth co-product can be isolated in >95% yield during product purification (Scheme 2, top). Converting this recovered material back into the corresponding bismuthonium salt (see the Supporting Information) reduces the waste that would otherwise be associated with the use of these reagents.

The methodology is equally applicable to primary amine nucleophiles (Scheme 2B) bearing linear aliphatic (**16**–**20**), benzylic (**21**–**24**), and α-branched (**25**) substituents. The functional group tolerance includes alkenes (**17**) and both electron-rich and electron-poor heterocycles (**22**–**24**). Significantly, a variety of complex amines derived from natural products and pharmaceutical compounds can be employed, affording amino acid conjugates of *rac-*mexiletine (**26**) and *rac-*tranylcypromine (**27**). The use of proteinogenic amino acids as the nucleophile generates *N*-linked dipeptides (**28**, **29**), a motif that forms the core of several highly successful ACE inhibitors—including enalapril, lisinopril, and benazepril^58,59^—and the opine family of natural products.^60,61^ The lower yield obtained for structurally complex amines presumably reflects the steric demands of the reaction, which requires an S_N_2 substitution at a quaternary center (Scheme 1B).

Variation of the α-keto ester counterpart revealed a significant sensitivity toward the identity of the substrate (Scheme 2C). Thus, while moderately electron-donating and -withdrawing substituents are well accommodated (**31**–**33**), lower yields are obtained at either electronic extreme (**30**, **34**).

A particular benefit of the present three-component strategy is that it allows any given aryl group to be introduced from either the α-keto ester or the bismuthonium salt, such that two distinct disconnections are viable for each amino acid target (Scheme 3). The practicality of this retrosynthetic flexibility is illustrated in the comparable yields obtained across three target compounds, irrespective of the combination of aryl moieties or the disconnection employed. Notably, this series of studies also demonstrates the scope with respect to the bismuthonium salt, which spans from electron poor (**33**, *disconnection 2*) to electron rich (**37**, *disconnection 2*).

**Scheme 3:**
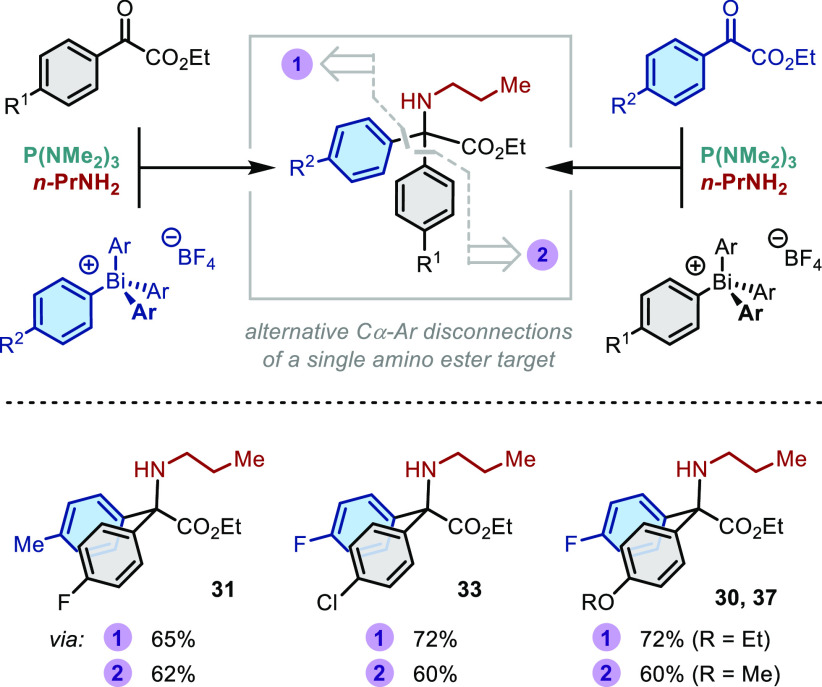
Comparison of C_quat_–Ar Disconnections Reactions performed on a 0.5 mmol scale using anhydrous CH_2_Cl_2_ ([**3**]_0_ = 0.1 M); yields determined by ^19^F NMR spectroscopic analysis vs internal standard (PhCF_3_).

Finally, we sought to extend our methodology to the synthesis of *N*-unsubstituted amino acids of type **38** (Scheme 4), a potentially valuable building block for drug discovery.^62,63^ Gratifyingly, **38** can either be accessed directly by simply using a solution of ammonia as the nitrogen nucleophile (*path i*) or indirectly *via* deprotection of the corresponding *N*-benzyl (*path ii*), *N*-*p*-methoxyphenyl (*path iii*), or *N*-allyl (*path iv*) amino esters.^64^

**Scheme 4:**
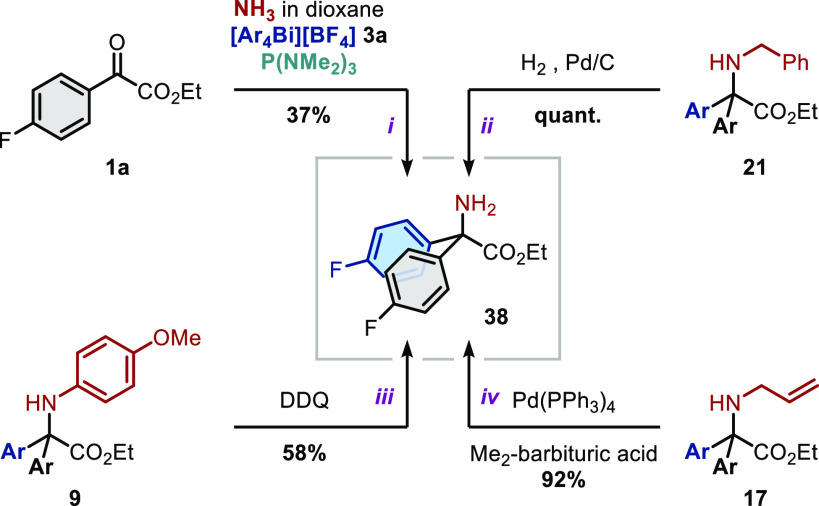
Synthesis of Primary Amino Esters See the Supporting Information for full experimental details.

In summary, we have developed a modular and versatile approach to α,α-diaryl α-amino esters in which two bonds to the quaternary center are formed in a single operation. Net deoxygenation of an α-keto ester substrate is achieved by reaction of the umpoled Kukhtin–Ramirez intermediate with an electrophilic Bi(V) arylating agent, prior to S_N_2-displacement of the resulting alkoxyphosphonium intermediate by an amine nucleophile. Each of the three components can be varied independently, giving concise access to diverse amino esters featuring synthetically valuable functionality. The scope with respect to the amine nucleophile includes both anilines and aliphatic amines; use of biologically relevant amine nucleophiles affords new drug conjugates and N-linked dipeptides, whereas the use of ammonia provides direct access to a key primary amino acid building block.

## Supporting Information Available

The data underlying this study are available in the published article and its online Supporting Information. The Supporting Information is available free of charge on the ACS Publications Web site: Experimental procedures, spectra, and optimization data (PDF) FAIR Data, including the primary NMR FID files for the following compounds: **1a**, **1c**, **1g**, **1h**, **3a**–**3d**, **8**–**38** and triarylbismuthines. The Supporting Information is available free of charge at https://pubs.acs.org/doi/10.1021/acs.orglett.2c03201.

Experimental procedures, spectra, and optimization data (PDF)
FAIR data, including the primary NMR FID files, for compounds **3a**–**3d** (ZIP)
FAIR data, including the primary NMR FID files, for compounds **8**–**12** (ZIP)
FAIR data, including the primary NMR FID files, for compounds **13**–**17** (ZIP)
FAIR data, including the primary NMR FID files, for compounds **18**–**22** (ZIP)
FAIR data, including the primary NMR FID files, for compounds **23**–**27** (ZIP)
FAIR data, including the primary NMR FID files, for compounds **28**–**32** (ZIP)
FAIR data, including the primary NMR FID files, for compounds **33**–**38** (ZIP)
FAIR data, including the primary NMR FID files, for compounds Bi compounds (ZIP)
FAIR data, including the primary NMR FID files, for compounds keto esters (ZIP)
