# Supplementary material for: Modular Synthesis of α,α-Diaryl α-Amino Esters via Bi(V)-Mediated Arylation/SN2-Displacement of Kukhtin–Ramirez Intermediates
Source: Org Lett. 2022 Oct 24;24(43):8002–7. doi: 10.1021/acs.orglett.2c03201 (PMC9641671; doi:10.1021/acs.orglett.2c03201)
Supplement: Supplementary file 1 — ol2c03201_si_001.pdf [file ol2c03201_si_001.pdf]

# SUPPORTING INFORMATION

## Modular Synthesis of α,α-Diaryl α-Amino Esters *via* Bi(V)-Mediated Arylation / $S_N2$-Displacement of Kukhtin-Ramirez Intermediates

Alessio Calcatelli[1], Ross M. Denton[1,2] and Liam T. Ball[1,*]

*Email: liam.ball@nottingham.ac.uk

orcid: A.C., 0000-0002-2571-5841; R.M.D., 0000-0003-1267-1971;
L.T.B., 0000-0003-3849-9006.

[1] School of Chemistry, University of Nottingham, Nottingham NG7 2RD, U.K.

[2] GlaxoSmithKline Carbon Neutral Laboratories for Sustainable Chemistry, University of Nottingham, 6 Triumph Road, Nottingham, NG7 2GA, U.K.

# Table of contents

1. **General Experimental Information** ........ 3
2. **Further Optimization Experiments** ........ 5
3. **Synthesis and Characterization of Organobismuth Compounds** ........ 6
   **3.1 General Procedure 1 (GP1) - Synthesis of Triarylbismuthines** ........ 6
   **3.2 Characterization Data for Triarylbismuthines** ........ 7
   **3.3 General Procedure 2 (GP2) - Synthesis of Tetraarylbismuthonium Tetrafluoroborate Salts** ........ 11
   **3.4 Characterization Data for Tetraarylbismuthonium Tetrafluoroborate Salts** ........ 12
   **3.5 General Procedure 3 (GP3) - Synthesis of α-Keto Esters *via* Friedel-Crafts Acylation** ........ 16
   **3.6 General Procedure 4 (GP4) - Synthesis of α-Keto Esters *via* Grignard Addition** ........ 17
   **3.7 Characterization Data for α-Keto Esters** ........ 18
4. **Synthesis and Characterization of Amino Esters** ........ 21
   **4.1 General Procedure 5 (GP5) - Synthesis of α,α-Diaryl α-Amino Esters** ........ 21
   **4.2 Characterization Data for N-Aryl Amino Esters (Manuscript Scheme 2A)** ........ 22
   **4.3 Characterization Data for N-Alkyl Amino Esters (Manuscript Scheme 2B)** ........ 30
   **4.4 Characterization Data for α,α-Aryl,Aryl′ Amino Esters (Manuscript Schemes 2C & 3)** ........ 44
   **4.4 Characterization Data for *N*-Unsubstituted Amino Esters (Manuscript Scheme 4)** ........ 52
5. **Product Derivatizations (Manuscript Scheme 4)** ........ 53
   **5.1 *N*-Debenzylation** ........ 53
   **5.2 *N*-Deallylation** ........ 53
   **5.3 *N*-De-*para*-methoxyphenylation** ........ 54
6. **Limitations of Substrate Scope** ........ 55
7. **NMR Spectra** ........ 56
8. **References** ........ 177

# 1. General Experimental Information

Procedures employing oxygen- and/or moisture-sensitive materials were performed with anhydrous solvents (*vide infra*) using standard inert-atmosphere techniques (atmosphere of anhydrous dinitrogen). Analytical thin-layer chromatography was performed on precoated aluminium-backed plates (Silica Gel 60 F254; Merck), and visualized using a combination of UV light (254 nm) and acidic ethanolic vanillin, aqueous basic potassium permanganate or iodine stains. Manual flash column chromatography was performed using Scharlab 60 silica gel (35-70 mesh); automated flash column chromatography was performed on disposable columns pre-packed with 50 µm spherical silica gel using a Büchi C-850 or C-815 equipped with a UV-vis DAD (200-800 nm) and an ELSD. Preparative HPLC was performed on a Büchi Prep Pure C18 column (100 Å, 10 µm, 250 × 30mm) using a Büchi C-850 equipped with a UV-vis DAD (200-800 nm) and an ELSD.

NMR spectra were recorded at 25 °C on Bruker Avance 500 or 400 spectrometers ($^{1}$H, 500 / 400 MHz; $^{13}$C{$^{1}$H}, 125 / 100 MHz; $^{19}$F NMR, 471 / 376 MHz; $^{31}$P NMR, 162 MHz). Chemical shifts are reported in ppm; coupling constants, *J*, are reported in Hz and are uncorrected for digitization. The following abbreviations (and their combinations) are used to label the multiplicities: s (singlet), d (doublet), t (triplet), q (quartet), quint (quintet), sept (septet), m (multiplet), br (broad), and app (apparent). Structural assignments were made with the assistance of COSY, HSQC and HMBC.

$^{1}$H and $^{13}$C{$^{1}$H} chemical shifts are reported relative to tetramethylsilane, and are referenced to the appropriate residual solvent peaks:

- $CDCl_3$: $\delta_H$ = 7.26 ppm, $\delta_C$ = 77.16 ppm
- DMSO-$d_6$: $\delta_H$ = 2.50 ppm, $\delta_C$ = 39.52 ppm
- $CD_3OD$: $\delta_H$ = 3.31 ppm, $\delta_C$ = 49.00 ppm.

$^{19}$F chemical shifts are reported relative to $CFCl_3$.

Infrared spectra of neat compounds were recorded over the range 4000-600 cm$^{-1}$ using either a PerkinElmer Spectrum 1000 Series FTIR spectrometer with an ATR diamond cell, or a Bruker Alpha FTIR spectrometer fitted with a Bruker Platinum ATR Quicksnap™ diamond cell. Melting points were measured using Stuart SMP10 or Gallenkamp melting point apparatus in open capillaries. High resolution electrospray ionization mass spectra (HRMS) were recorded using a Bruker ESI-TOF MicroTOF II spectrometer. Optical rotation values were recorded using a ADP400$^{+}$ BS polarimeter.

Reagent grade solvents (Fisher Technical) were employed. $CH_2Cl_2$ was dried over activated 3Å molecular sieves. MeCN and THF were dried using an Inert PureSolv Grubbs-type system (activated alumina columns, argon atmosphere).

Unless stated otherwise, all reagents were obtained from commercial sources and were used as received.

## 2. Further Optimization Experiments

**Table S1.** Variation of reagent stoichiometry.[a]

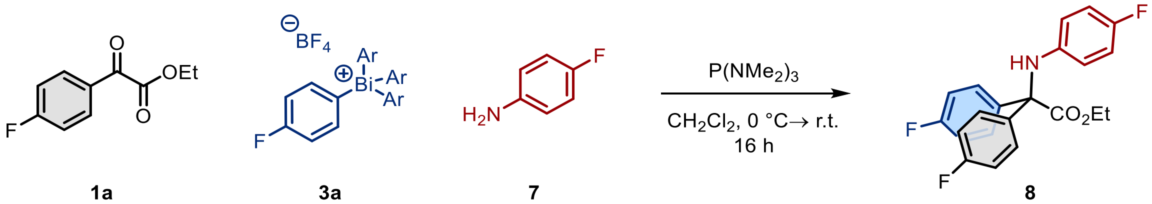


| Entry | Equivalents | | | | % Yield 8 |
|---|---|---|---|---|---|
| | **1a** | **3a** | **7** | **P(NMe$_2$)$_3$** | |
| **1** | 2 | 1 | 3 | 2.5 | 72 |
| **2**[b] | 2 | 1 | 3 | 2.5 | 62 |
| **3** | 2 | 1 | 2 | 2.5 | 78 |
| **4** | 1.5 | 1 | 3 | 2 | 74 |
| **5** | 1.5 | 1 | 2 | 2 | 77 |
| **6** | 1.5 | 1 | 1.5 | 2 | 80 |
| **7** | 1 | 1 | 3 | 1.5 | 66 |

[a] Reactions performed on a 0.1 mmol scale using anhydrous $CH_2Cl_2$ ([**3a**]$_0$ = 0.1 M) following GP5; yields determined by $^{19}$F NMR spectroscopic analysis *vs* internal standard ($PhCF_3$). [b] 2 equiv. $NaHCO_3$ added at $t_0$.

# 3. Synthesis and Characterization of Organobismuth Compounds

## 3.1 General Procedure 1 (GP1) - Synthesis of Triarylbismuthines

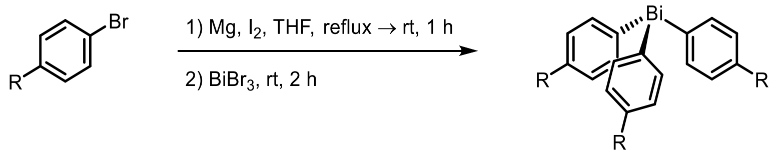


A flame dried Schlenk tube containing magnesium turnings (792 mg, 33.0 mmol, 3.3 equiv.) was evacuated and back filled with nitrogen three times. Iodine (2 crystals) was added and the Schlenk tube was gently heated until purple vapour was observed. After cooling to rt, anhydrous THF (15 mL) was added and the mixture was stirred. The aryl bromide (33.0 mmol, 3.3 equiv.) was then added dropwise so that the reaction mixture came to, and maintained, a gentle reflux. The mixture was stirred for a further hour without heating.

The freshly prepared Grignard reagent was added dropwise to a stirred solution of bismuth tribromide (4.48 g, 10.0 mmol, 1.00 equiv.) in anhydrous THF (20 mL). The reaction mixture was stirred for 2 h, then quenched with MeOH (10 mL) and filtered through a pad of silica gel (4 cm depth, 6 cm diameter; eluent: $Et_2O$). The filtrate was washed with water (3 × 20 mL), dried over $MgSO_4$ and concentrated under reduced pressure. The resulting crude material was recrystallised from EtOH, yielding the desired product as a solid.

## 3.2 Characterization Data for Triarylbismuthines

### Tri(4-fluorophenyl)bismuthine

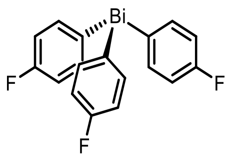


Following GP1, tri(4-fluorophenyl)bismuthine was obtained from bismuth tribromide (4.48 g, 10.0 mmol) and 4-fluorobromobenzene (5.55 mL, 5.77 g, 33.0 mmol). The crude product was crystallised from EtOH to afford the product as a colourless solid (4.70 g, 9.50 mmol, 95% yield). The characterisation data are in agreement with the literature.[3]

**m.p. /°C:** 90 – 92.

**$^{1}$H NMR (500 MHz, $CDCl_3$):** $\delta_H$ 7.66 (dd, *J* = 8.4, 6.2 Hz, 6H), 7.09 (app t, *J* = 9.0 Hz, 6H).

**$^{13}$C{$^{1}$H} NMR (126 MHz, $CDCl_3$):** $\delta_C$ 163.5 (d, *J* = 247.3 Hz), 149.6, 139.3 (d, *J* = 7.0 Hz), 118.1 (d, *J* = 19.7 Hz).

**$^{19}$F NMR (471 MHz, $CDCl_3$):** $\delta_F$ -112.67 (tt, *J* = 9.4, 6.1 Hz).

**$\tilde{\upsilon}$ (ATR)/cm$^{-1}$:** 1572, 1481, 1382, 1209, 1156, 1015, 811, 504, 411.

**HRMS** calcd for $C_{12}H_8BiF_2^+$: 399.0398 $[M-C_6H_4F]^+$; found ($ESI^+$): 399.0398.

**Tri(4-methylphenyl)bismuthine**

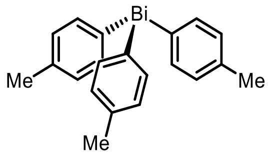


Following GP1, tri(4-methylphenyl)bismuthine was obtained from bismuth tribromide (4.48 g, 10.0 mmol) and 4-bromotoluene (4.06 mL, 5.66 g, 33.0 mmol). The crude product was crystallised from EtOH to afford the product as a colourless solid (4.75 g, 9.80 mmol, 98% yield). The characterisation data are in agreement with the literature.[4,5] IR data for this compound have not been reported previously.

**m.p. /°C:** 118 – 120.

**$^{1}$H NMR (500 MHz, CDCl$_3$):** $\delta_H$ 7.65 (d, *J* = 7.6 Hz, 6H), 7.22 (d, *J* = 7.6 Hz, 6H), 2.35 (s, 9H).

**$^{13}$C{$^{1}$H} NMR (126 MHz, CDCl$_3$):** $\delta_C$ 151.1 (br), 137.6, 137.4, 131.3, 21.6.

**ῦ (ATR)/cm$^{-1}$:** 3055, 2951, 1485, 1441, 1308, 1207, 1051, 1010, 907, 787, 731, 562, 472.

**HRMS** calcd for $C_{14}H_{14}Bi^{+}$: 391.0893 [M-$C_7H_7$]$^{+}$; found (ESI$^{+}$): 391.0888.

**Tri(4-methoxyphenyl)bismuthine**

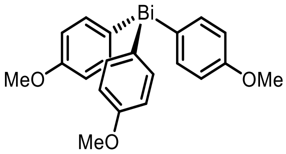


Following GP1, tri(4-methoxyphenyl)bismuthine was obtained from bismuth tribromide (4.48 g, 10.0 mmol) and 4-bromoanisole (4.14 mL, 6.17 g, 33.0 mmol). The crude product was crystallised from EtOH to afford the product as a colourless solid (4.78 g, 9.00 mmol, 90% yield). The characterisation data are in agreement with the literature.[6,7] HRMS data for this compound have not been reported previously.

**m.p. /°C:** 182 – 184.

**$^1$H NMR (500 MHz, CDCl$_3$):** $\delta_H$ 7.64 (d, *J* = 8.4 Hz, 6H), 6.94 (d, *J* = 8.4 Hz, 6H), 3.82 (s, 9H).

**$^{13}$C{$^1$H} NMR (126 MHz, CDCl$_3$):** $\delta_C$ 159.3, 139.1, 138.7, 116.3, 55.1.

**$\tilde{\upsilon}$ (ATR)/cm$^{-1}$:** 3051, 2690, 2834, 1576, 1486, 1305, 1277, 1237,1114, 1098, 1000, 815, 758,516.

**HRMS** calcd for $C_{21}H_{21}BiNaO_3^+$: 553.1187 [M+H]$^+$; found (ESI$^+$): 553.1211.

**Tri(4-chlorophenyl)bismuthine**

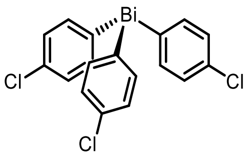


Following GP1, tri(4-chlorophenyl)bismuthine was obtained from bismuth tribromide (4.48 g, 10.0 mmol) and 4-chlorobromobenzene (3.95 mL, 6.32 g, 33.0 mmol). The crude product was crystallised from EtOH to afford the product as a colourless solid (4.50 g, 8.30 mmol, 83% yield). The characterisation data are in agreement with the literature.[8] $^{1}$H NMR and HRMS data for this compound have not been reported previously.

**m.p. /°C:** 103 – 105.

**$^{1}$H NMR (400 MHz, CDCl$_3$):** $\delta_H$ 7.63 (d, *J* = 8.6 Hz, 6H), 7.38 – 7.34 (d, *J* = 8.6, 6H).

**$^{13}$C{$^{1}$H} NMR (101 MHz, CDCl$_3$):** $\delta_C$ 152.8, 138.8, 134.5, 130.9.

**ῦ (ATR)/cm$^{-1}$:** 3044, 1558, 1470, 1376, 1470, 1376, 1299, 1086, 1044, 1005, 905, 803, 730, 715, 668, 480.

**HRMS** calcd for $C_{12}H_8BiCl_2^+$: 430.9802 [M-$C_6H_4$Cl]$^+$; found (ESI$^+$): 430.9812.

## 3.3 General Procedure 2 (GP2) - Synthesis of Tetraarylbismuthonium Tetrafluoroborate Salts

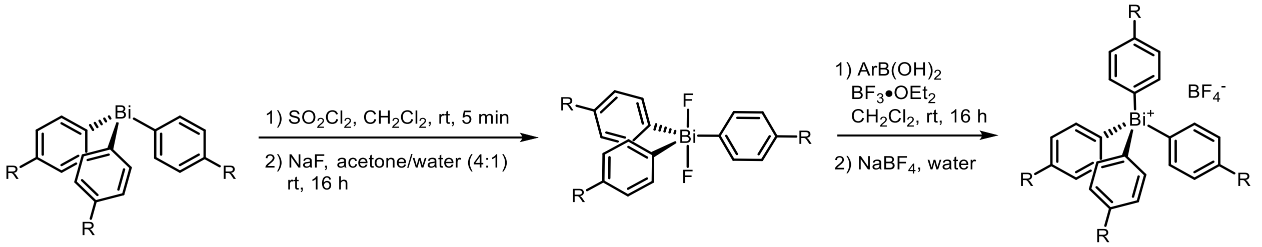


Sulfuryl chloride (80 µL, 1.0 mmol) was added dropwise to a solution of triarylbismuthine (1.0 mmol) in anhydrous $CH_2Cl_2$ (5 mL) at room temperature. After 5 minutes the solvent was evaporated under reduced pressure and the crude material was dissolved in acetone (20 mL) and water (5 mL). Sodium fluoride (210 mg, 5.0 mmol) was added and the reaction was stirred at room temperature for 3 h. The mixture was extracted with $CH_2Cl_2$ (3 × 20 mL) and the combined organic phases were washed with water (3 × 10 mL). The solvent was removed *in vacuo* and the residue was dissolved in acetone (20 mL). Water (5 mL) and NaF (210 mg, 5.0 mmol) were added, and the reaction was stirred for 16 h. The mixture was again extracted with $CH_2Cl_2$ (3 × 20 mL), and the combined organic portions were washed with water (3 × 10 mL), dried over $MgSO_4$, filtered, and the solvent evaporated under reduced pressure. The crude triarylbismuth difluoride was used directly in the next step without further purification.

A round bottom flask was charged with the crude difluorotriarylbismuth (1.0 mmol) and the corresponding *p*-substituted boronic acid (1.1 mmol). The round bottom flask was evacuated and back filled with anhydrous dinitrogen three times, then anhydrous $CH_2Cl_2$ (10 mL) was added and the stirred mixture was cooled to 0 °C. $BF_3$•$OEt_2$ (170 µL, 1.5 mmol, 1.5 equiv.) was added dropwise at 0 °C, then the reaction was stirred at room temperature for 16 h. Sodium tetrafluoroborate (0.50 g, 5.0 mmol) and water (10 mL) were added, and the organic phase was separated. The aqueous phase was extracted with $CH_2Cl_2$ (3 × 10 mL), and the combined organic portions were dried over $MgSO_4$ and concentrated under reduced pressure. The crude material was purified by crystallisation from $CH_2Cl_2$ – CyHex.

## 3.4 Characterization Data for Tetraarylbismuthonium Tetrafluoroborate Salts

### Tetra(4-fluorophenyl)bismuthonium Tetrafluoroborate (3a)

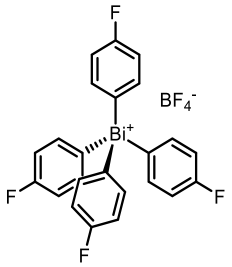


Following GP2, compound **3a** was obtained from tri(4-fluorophenyl)bismuthine (0.98 g, 2.00 mmol) and 4-fluorobenzene boronic acid (0.34 g, 2.40 mmol). The crude product was crystallised from $CH_2Cl_2$ - CyHex to afford the product as a colourless solid (1.08 g, 1.60 mmol, 80% yield).

**m.p. /°C:** 241 – 243.

**$^1$H NMR (400 MHz, $CDCl_3$):** $\delta_H$ 7.81 (dd, *J* = 8.7, 5.2, 8H), 7.40 (app t, *J* = 8.7 Hz, 8H).

**$^{13}$C{$^1$H} NMR (101 MHz, $CDCl_3$):** $\delta_C$ 165.1 (d, *J* = 255.7 Hz), 137.9 (d, *J* = 8.5 Hz), 132.9 (d, *J* = 3.4 Hz), 119.9 (d, *J* = 22.0 Hz).

**$^{19}$F NMR (376 MHz, $CDCl_3$):** -103.88 (tt, *J* = 8.4, 5.2 Hz, 4F), -147.68 and -147.71 (4F, $^{10}BF_4$ and $^{11}BF_4$).

**$\tilde{\upsilon}$ (ATR)/cm$^{-1}$:** 1589, 1574, 1483, 1392, 1303, 1230, 1160, 1056, 1002, 819, 570, 500, 413.

**HRMS** calcd for $C_{24}H_{16}BiF_4^+$: 589.0987 $[M-BF_4]^+$; found (ESI$^+$): 589.1003.

**Tetra(4-methylphenyl)bismuthonium Tetrafluoroborate (3b)**

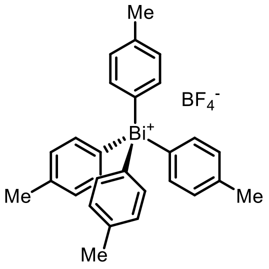


Following GP2, compound **3b** was obtained from tri(4-methylphenyl)bismuthine (0.96 g, 2.00 mmol) and 4-methylbenzene boronic acid (0.33 g, 2.40 mmol). The crude product was crystallised from $CH_2Cl_2$ - CyHex to afford the product as an off-white solid (1.30 g, 1.95 mmol, 95% yield). The characterisation data are in agreement with the literature.[9] $^{13}C\{^1H\}$ NMR and HRMS data for this compound have not been reported previously.

**m.p. /°C:** 152 – 154.

**$^1$H NMR (400 MHz, CDCl$_3$):** $\delta_H$ 7.66 (d, *J* = 7.8 Hz, 8H), 7.50 (d, *J* = 7.8 Hz, 8H), 2.46 (s, 12H).

**$^{13}$C{$^1$H} NMR (101 MHz, CDCl$_3$):** $\delta_C$ 143.2, 135.6, 133.4, 133.0. 21.6.

**ῦ (ATR)/cm$^{-1}$:** 2956, 1487, 1392, 1188, 1055, 1032, 1004, 796, 475.

**HRMS** calcd for $C_{28}H_{28}Bi^+$: 573.1990 $[M-BF_4]^+$; found (ESI$^+$): 573.2015.

**Tetra(4-methoxyphenyl)bismuthonium Tetrafluoroborate (3c)**

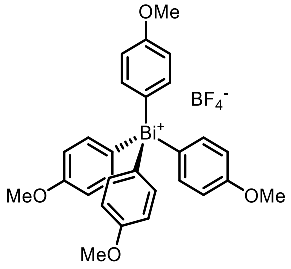


Following GP2, compound **3c** was obtained from tri(4-methoxy phenyl)bismuthine (1.06 g, 2.00 mmol) and 4-methoxybenzene boronic acid (0.36 g, 2.40 mmol). The crude product was crystallised from $CH_2Cl_2$ - CyHex to afford the product as a brown solid (1.20 g, 1.64 mmol, 82% yield).

**m.p. /°C:** 156 – 158.

**$^1$H NMR (500 MHz, $CDCl_3$):** $\delta_H$ 7.68 (d, *J* = 8.7 Hz, 8H), 7.21 (d, *J* = 8.8 Hz, 8H), 3.89 (s, 12H).

**$^{13}$C{$^1$H} NMR (126 MHz, $CDCl_3$):** $\delta_C$ 162.8, 137.1, 126.0, 118.1, 55.7.

**ῦ (ATR)/cm$^{-1}$:** 1578, 1568, 1488, 1294, 1251, 1177, 1053, 1018, 912, 820, 729, 664, 584, 512, 471.

**HRMS** calcd for $C_{28}H_{28}BiO_4^+$: 637.1798 $[M-BF_4]^+$; found ($ESI^+$): 637.1786.

**Tetra(4-chlorophenyl)bismuthonium Tetrafluoroborate (3d)**

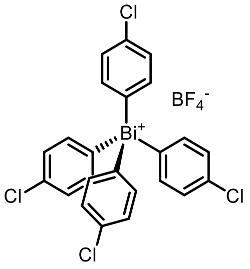


Following GP2, compound 3**d** was obtained from tri(4-chloro phenyl)bismuthine (1.08 g, 2.00 mmol) and 4-chlorophenyl boronic acid (0.38 g, 2.40 mmol). The crude product was crystallised from $CH_2Cl_2$ - CyHex to afford the product as an off-white solid (0.60 g, 0.81 mmol, 40% yield). The characterisation data are in agreement with the literature.[10] $^{13}C\{^1H\}$ NMR and HRMS data for this compound have not been reported previously.

**m.p. /°C:** 177 – 175.

**$^1$H NMR (500 MHz, CDCl$_3$):** $\delta_H$ 7.71 (d, *J* = 8.4 Hz, 8H), 7.63 (d, *J* = 8.4 Hz, 8H).

**$^{13}$C{$^1$H} NMR (126 MHz, CDCl$_3$):** $\delta_C$ 139.6, 136.8, 136.5, 132.6.

**ῦ (ATR)/cm$^{-1}$:** 1594, 1461, 1339, 1367, 1346, 1087, 1039, 805, 776, 671.

**HRMS** calcd for $C_{24}H_{16}BiCl_4^+$: 652.9794 $[M-BF_4]^+$; found (ESI$^+$): 652.9805.

### 3.5 General Procedure 3 (GP3) - Synthesis of α-Keto Esters *via* Friedel-Crafts Acylation

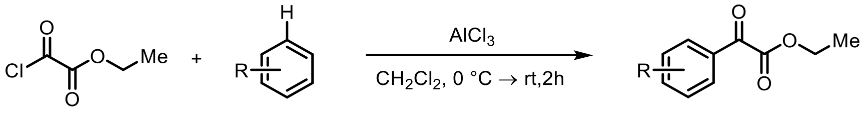


According to literature procedure:[11] a suspension of $AlCl_3$ (2.26 g, 17.0 mmol) in anhydrous $CH_2Cl_2$ (12 mL) was stirred at 0 °C under an atmosphere of anhydrous dinitrogen. Mono-ethyl oxalyl chloride (1.90 mL, 17.0 mmol) was added dropwise over 15 min before the appropriate arene (9.0 mmol) was added dropwise over 15 min. The cooling bath was then removed and the reaction was stirred at r.t. for 1 h. The mixture was cooled to 0 °C, then water(5 mL) and 1M aqueous HCl (5 mL) were added sequentially. The organic phase was separated, and the aqueous phase was extracted with $CH_2Cl_2$ (3 × 15 mL). The combined organic portions were washed with a saturated aqueous solution of $NaHCO_3$ (40 mL) and brine (40 mL), dried over $MgSO_4$, and concentrated onto silica gel for purification by column chromatography (silica gel; see individual compound entries for eluent).

## 3.6 General Procedure 4 (GP4) - Synthesis of α-Keto Esters *via* Grignard Addition

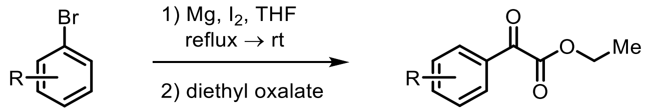


According to literature procedure:[11] A flame dried Schlenk tube containing magnesium turnings (396 mg, 11.0 mmol) was evacuated and back-filled with nitrogen three times. Iodine (2 crystals) was added and the Schlenk tube was gently heated until purple vapour was observed. After cooling to rt, anhydrous THF (30 mL) was added and the mixture was stirred. The aryl bromide (11.0 mmol) was then added dropwise so that the reaction mixture came to, and maintained, a gentle reflux. The mixture was stirred for a further hour without heating.

The freshly prepared Grignard reagent was added dropwise over 1 h to a solution of diethyl oxalate (1.36 mL, 10.0 mmol, 1.00 equiv.) in anhydrous THF (60 mL) at –78 °C. The reaction mixture was stirred for 1 h at –78 °C before being allowed to warm to room temperature over 30 min. The reaction mixture was quenched with water (100 mL) and the aqueous phase was separated and extracted with diethyl ether (3 × 50 mL). The combined organic portions were washed with brine, dried over $MgSO_4$ and concentrated onto silica gel. The crude material was purified by column chromatography on silica gel (see individual compound entries for eluent).

## 3.7 Characterization Data for α-Keto Esters

### Ethyl 2-(4-fluorophenyl)-2-oxoacetate (1a)

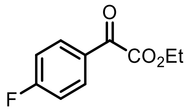

Following GP3, ethyl 2-(4-fluorophenyl)-2-oxoacetate **1a** was obtained from fluorobenzene (1.90 mL, 9.00 mmol). Purification by column chromatography (100% to 9:1 CyHex: EtOAc) afforded the product as a yellow oil (1.34g, 6.84 mmol, 76% yield). The characterisation data are in agreement with the literature.[12] IR and HRMS data for this compound have not been reported previously.

**$^1$H NMR (400 MHz, CDCl$_3$):** $\delta_H$ 8.10 (dd, *J* = 8.9, 5.3 Hz, 2H), 7.21 (app t, *J* = 8.9 Hz, 2H), 4.47 (q, *J* = 7.2 Hz, 2H), 1.45 (t, *J* = 7.2 Hz, 3H).

**$^{13}$C{$^1$H} NMR (101 MHz, CDCl$_3$):** $\delta_C$ 184.6, 166.8 (d, *J* = 258.3 Hz), 163.4, 133.0 (d, *J* = 9.8 Hz), 129.0 (d, *J* = 2.8 Hz), 116.3 (d, *J* = 22.2 Hz), 62.5, 14.1.

**$^{19}$F NMR (376 MHz, CDCl$_3$):** $\delta_F$ -101.24 (tt, *J* = 8.3, 5.3 Hz).

**ῦ (ATR)/cm$^{-1}$ :** 1732, 1685, 1597, 1234, 1194, 1154, 1013, 980, 843, 635, 505.

**HRMS** calcd for $C_{10}H_9FNaO_3^+$: 219.0428 [M+Na]$^+$; found (ESI$^+$) 219.0430

**Ethyl 2-oxo-2-(*p*-tolyl)acetate (1c)**

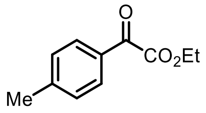


Following GP4, ethyl 2-oxo-2-(*p*-tolyl)acetate **1c** was obtained from 1-bromo-4-methylbenzene (1.23 mL, 10.0 mmol). Purification by column chromatography (100 % to 9:1 CyHex : EtOAc) afforded the product as a yellow oil (1.17 g, 6.10 mmol, 61% yield). The characterisation data are in agreement with the literature.[12] IR and HRMS data for this compound have not been reported previously.

**$^1$H NMR (400 MHz, $CDCl_3$):** $\delta_H$ 7.93 (d, *J* = 8.2 Hz, 2H), 7.33 (d, *J* = 8.2 Hz, 2H), 4.47 (d, *J* = 7.1 Hz, 2H), 2.46 (s, 3H), 1.44 (t, *J* = 7.1 Hz, 3H).

**$^{13}$C{$^1$H} NMR (101 MHz, $CDCl_3$):** $\delta_C$ 186.1, 164.0, 146.2, 130.2, 130.1, 129.6, 62.2, 21.9, 14.1.

**$\tilde{\upsilon}$ (ATR)/cm$^{-1}$:** 1732, 1679, 1604, 1318, 1214, 1173, 1013, 976, 827, 683, 479.

**HRMS** calcd for $C_{11}H_{12}NaO_3^+$: 215.0679 [M+Na]$^+$; found (ESI$^+$) 215.0681.

**Ethyl 2-(3-bromo-4-methoxyphenyl)-2-oxoacetate (1g)**

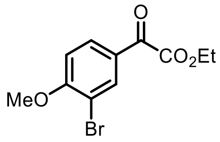


Following GP3, ethyl 2-(3-bromo-4-methoxyphenyl)-2-oxoacetate **1g** was obtained from 1-bromo-2-methoxybenzene (1.13 mL, 9.00 mmol). Purification by column chromatography (100 % to 95:5 CyHex : EtOAc) afforded the product as a yellow oil (1.21 g, 4.21 mmol, 47 % yield).

**$^1$H NMR (400 MHz, $CDCl_3$):** $\delta_H$ 8.29 (d, *J* = 2.1 Hz, 1H), 8.04 (dd, *J* = 8.7, 2.1 Hz, 1H), 7.00 (d, *J* = 8.7 Hz, 1H), 4.47 (q, *J* = 7.2 Hz, 2H), 4.02 (s, 3H), 1.45 (t, *J* = 7.2 Hz, 3H).

**$^{13}$C{$^1$H} NMR (101 MHz, $CDCl_3$):** $\delta_C$ 183.6, 163.4, 161.1, 135.4, 131.8, 126.6, 112.4, 111.3, 62.5, 56.7, 14.2.

**$\tilde{\upsilon}$ (ATR)/cm$^{-1}$:** 1721, 1670, 1589, 1559, 1491, 1310, 1281, 1255, 1181, 1016, 947, 826, 755, 669, 447.

**HRMS** calcd for $C_{11}H_{12}BrO_4^+$: 286.9913 [M+H]$^+$; found (ESI$^+$) 286.9907

**Ethyl 2-(naphthalen-2-yl)-2-oxoacetate (1h)**

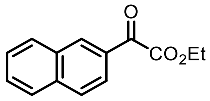


Following GP4, ethyl 2-(naphthalen-2-yl)-2-oxoacetate **1h** was obtained from 2-bromonaphtalene (1.64 mL, 10.0 mmol). Purification by column chromatography (100 % to 9:1 CyHex : EtOAc) afforded the product as a yellow oil (415 mg, 1.75 mmol, 17% yield). The characterisation data are in agreement with the literature.[12] IR and HRMS data for this compound have not been reported previously.

**$^1$H NMR (400 MHz, $CDCl_3$):** $\delta_H$ 8.59 (s, 1H), 8.08 (dd, *J* = 8.7, 1.8 Hz, 1H), 8.04 – 7.88 (m, 3H), 7.69 (ddd, *J* = 8.2, 6.8, 1.4 Hz, 1H), 7.61 (ddd, *J* = 8.2, 6.8, 1.4 Hz, 1H), 4.54 (q, *J* = 7.1 Hz, 2H), 1.49 (t, *J* = 7.1 Hz, 3H).

**$^{13}$C{$^1$H} NMR (101 MHz, $CDCl_3$):** $\delta_C$ 186.4, 164.0, 136.4, 133.5, 132.3, 130.0, 129.9, 129.6, 129.0, 128.0, 127.2, 124.0, 62.4, 14.2.

**$\tilde{\upsilon}$ (ATR)/cm$^{-1}$:** 1719, 1670, 1550, 1451, 1300, 1271, 1163, 1048, 908, 860, 734, 702, 646, 445.

**HRMS** calcd for $C_{14}H_{13}O_3^+$: 229.0859 [M+H]$^+$; found (ESI$^+$) 229.0863

# 4. Synthesis and Characterization of Amino Esters

## 4.1 General Procedure 5 (GP5) - Synthesis of α,α-Diaryl α-Amino Esters

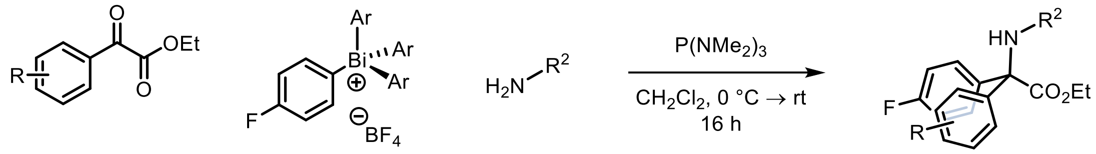


A microwave tube equipped with a magnetic stirring bar was charged with tetra(4-fluorophenyl)bismuthonium tetrafluoroborate **3a** (338 mg, 0.50 mmol) and α-keto ester **1** (0.75 mmol). The tube was sealed with a rubber septum, evacuated, and backfilled with nitrogen three times, then anhydrous $CH_2Cl_2$ (5 mL) was added. The resulting mixture was cooled to 0 °C. The appropriate aniline (0.75 mmol) or primary amine (1.5 mmol) was added, the $P(NMe_2)_3$ (181 μL, 1.0 mmol) was added dropwise over 1 min at 0 °C. The resulting mixture was stirred and allowed to warm to rt over 16 h.

<u>Work-up for anilines:</u> the resulting mixture was washed with water (5 mL × 3) and the organic phase was dried over $MgSO_4$. The solvent was removed under reduced pressure and the crude product was purified by preparative HPLC (Büchi Prep Pure C18 column: 100 Å, 10 µm, 250 × 30mm; eluent: 60:40 to 95:5 MeCN:water over 35 minute @ 15 mL/min flow rate).

<u>Work-up for primary amines:</u> the reaction mixture was concentrated onto silica gel and purified by column chromatography on silica gel (see individual compound entries for details).

## 4.2 Characterization Data for N-Aryl Amino Esters (Manuscript Scheme 2A)

**Ethyl 2,2-bis(4-fluorophenyl)-2-((4-fluorophenyl)amino)acetate (8)**

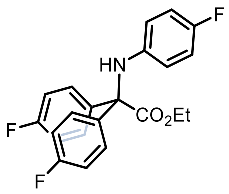


Following GP5, compound **8** was obtained from ethyl 2-(4-fluorophenyl)-2-oxoacetate **1a** (147 mg, 0.75 mmol) and 4-fluoroaniline (83.3 mg, 72.0 μL, 0.75 mmol). The crude product was purified by preparative HPLC to afford the product as an off-white solid (142 mg, 0.37 mmol, 74% yield).

**m.p. /°C:** 130 – 132.

**$^1$H NMR (400 MHz, CDCl$_3$):** $\delta_H$ 7.49 (dd, *J* = 8.9, 5.2 Hz, 4H), 7.00 (app t, *J* = 8.7 Hz, 4H), 6.71 (app t, *J* = 8.7 Hz, 2H), 6.36 (dd, *J* = 9.0, 4.5 Hz, 2H), 5.30 (br, 1H), 4.20 (q, *J* = 7.1 Hz, 2H), 1.09 (t, *J* = 7.1 Hz, 3H).

**$^{13}$C{$^1$H} NMR (101 MHz, CDCl$_3$):** $\delta_C$ 172.7 162.1 (d, *J* = 247.7 Hz), 156.4 (d, *J* = 236.8 Hz), 141.2, 135.7 (d, *J* = 3.4 Hz), 130.2 (d, *J* = 8.1 Hz), 116.9 (d, *J* = 7.2 Hz), 115.3 (d, *J* =21.4 Hz), 115.2 (d, *J* = 21.4 Hz), 70.9, 2.5, 13.8.

**$^{19}$F NMR (376 MHz, CDCl$_3$):** $\delta_F$ -114.30 (tt, *J* = 8.9, 5.2 Hz, 2F), -126.39 (tt, *J* = 9.0, 4.5 Hz, 1F).

**ʋ̃ (ATR)/cm$^{-1}$:** 3398, 2982, 1728, 1602, 1504, 1222, 1159, 1083, 823, 649, 534.

**HRMS** calcd for $C_{22}H_{19}F_3NO_2^+$: 386.1362 [M+H]$^+$; found (ESI$^+$): 386.1367.

**Ethyl 2,2-bis(4-fluorophenyl)-2-((4-methoxyphenyl)amino)acetate (9)**

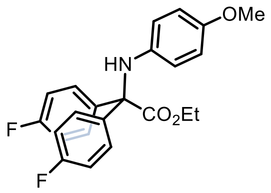


Following GP5, compound **9** was obtained from ethyl 2-(4-fluorophenyl)-2-oxoacetate **1a** (147 mg, 0.75 mmol) and 4-methoxyaniline (92.4 mg, 86.0 μL, 0.75 mmol). The crude product was purified by preparative HPLC to afford the product as a yellow oil (105 mg, 0.26 mmol, 53% yield).

**$^1$H NMR (400 MHz, CDCl$_3$):** $\delta_H$ 7.53 (dd, *J* = 9.0, 5.3 Hz, 4H), 7.01 (app t, *J* = 9.0 Hz, 4H), 6.62 (d, *J* = 9.0 Hz, 2H), 6.40 (d, *J* = 9.0 Hz, 2H), 5.12 (br, 1H), 4.19 (q, *J* = 7.1 Hz, 2H), 3.70 (s, 3H), 1.08 (t, *J* = 7.1 Hz, 3H).

**$^{13}$C{$^1$H} NMR (101 MHz, CDCl$_3$):** $\delta_C$ 172.9, 162.0 (d, *J* = 247.3 Hz), 152.8, 138.8, 136.3 (d, *J* = 3.3 Hz), 130.1 (d, *J* = 8.0 Hz), 117.4, 115.0 (d, *J* = 21.4 Hz), 114.1, 71.2, 62.3, 55.5, 13.8.

**$^{19}$F NMR (376 MHz, CDCl$_3$):** $\delta_F$ -114.68 (tt, *J* = 9.0, 5.4 Hz).

**$\tilde{\upsilon}$ (ATR)/cm$^{-1}$:** 3290, 2980, 1728, 1610, 1580, 1505, 1298, 1233, 1220, 1191, 841, 576.

**HRMS** calcd for $C_{23}H_{21}F_2NNaO_3^+$: 420.1397 [M+Na]$^+$; found (ESI$^+$): 420.1382.

**Ethyl 2,2-bis(4-fluorophenyl)-2-(p-tolylamino)acetate (10)**

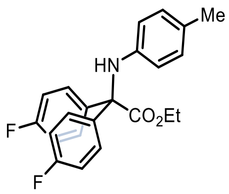


Following GP5, compound **10** was obtained from ethyl 2-(4-fluorophenyl)-2-oxoacetate **1a** (147 mg, 0.75 mmol) and *p*-toluidine (80.4 mg, 83.0 μL, 0.75 mmol). The crude product was purified by preparative HPLC to afford the product as an off-white solid (143 mg, 0.37 mmol, 75% yield).

**m.p. /°C:** 118-120.

**$^1$H NMR (400 MHz, $CDCl_3$):** $\delta_H$ 7.53 (dd, *J* = 8.9, 5.3 Hz, 4H), 7.01 (app t, *J* = 8.7 Hz, 4H), 6.83 (d, *J* = 8.7 Hz, 2H), 6.35 (d, *J* = 8.7 Hz, 2H), 5.27 (br, 1H), 4.19 (q, *J* = 7.1 Hz, 2H), 2.18 (s, 3H), 1.09 (t, *J* = 7.1 Hz, 3H).

**$^{13}$C{$^1$H} NMR (101 MHz, $CDCl_3$):** $\delta_C$ 172.9, 162.0 (d, *J* = 247.4 Hz), 142.5, 136.0 (d, *J* = 3.3 Hz), 130.1 (d, *J* = 8.1 Hz), 129.2, 127.6, 115.8, 115.0 (d, *J* = 21.5 Hz), 70.6, 62.4, 20.4, 13.8.

**$^{19}$F NMR (376 MHz, $CDCl_3$):** $\delta_F$ -114.63 (tt, *J* = 8.4, 5.3 Hz).

**$\tilde{\upsilon}$ (ATR)/cm$^{-1}$:** 3399, 2981, 2921, 1729, 1615, 1505, 1300, 1226, 1193, 1160, 1015, 839, 732, 577.

**HRMS** calcd for $C_{23}H_{22}F_2NO_2^+$: 382.1618 $[M+H]^+$; found ($ESI^+$): 382.1613.

**Ethyl 4-((2-ethoxy-1,1-bis(4-fluorophenyl)-2-oxoethyl)amino)benzoate (11)**

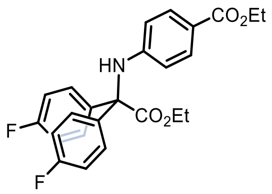


Following GP5, compound **11** was obtained from ethyl 2-(4-fluorophenyl)-2-oxoacetate **1a** (147 mg, 0.75 mmol) and 4-(ethoxycarbonyl) aniline (124 mg, 0.75 mmol). The crude product was purified by preparative HPLC to afford the product as a yellow oil (160 mg, 0.36 mmol, 73% yield).

**$^1$H NMR (400 MHz, $CDCl_3$):** $\delta_H$ 7.72 (d, *J* = 8.9 Hz, 2H), 7.51 (dd, *J* = 9.0, 5.2 Hz, 4H), 7.04 (app t, *J* = 8.7 Hz, 4H), 6.40 (d, *J* = 8.9 Hz, 2H), 6.00 (br, 1H), 4.28 (q, *J* = 7.1 Hz, 2H), 4.22 (q, *J* = 7.1 Hz, 2H), 1.33 (t, *J* = 7.1 Hz, 3H), 1.12 (t, *J* = 7.1 Hz, 3H).

**$^{13}$C{$^1$H} NMR (101 MHz, $CDCl_3$):** $\delta_C$ 172.4, 166.6, 162.3 (d, *J* = 248.3 Hz), 148.6, 134.4 (d, *J* = 3.4 Hz), 130.8, 130.3 (d, *J* = 8.1 Hz), 119.9, 115.3 (d, *J* = 21.5 Hz), 114.5, 70.2, 62.9, 60.3, 14.4, 13.8.

**$^{19}$F NMR (376 MHz, $CDCl_3$):** $\delta_F$ -113.81 (tt, *J* = 8.3, 5.2 Hz).

**ῦ (ATR)/cm$^{-1}$:** 3387, 2982, 1731, 1702, 1604, 1519, 1268, 1229, 1175, 1106, 1016, 837, 733, 578.

**HRMS** calcd for $C_{25}H_{24}F_2NO_4^+$: 440.1668 $[M+H]^+$; found (ESI$^+$): 440.1657.

**Ethyl 2,2-bis(4-fluorophenyl)-2-((4-(trifluoromethyl)phenyl)amino)acetate (12)**

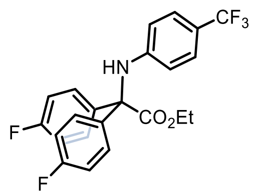


Following GP5, compound **12** was obtained from ethyl 2-(4-fluorophenyl)-2-oxoacetate **1a** (147 mg, 0.75 mmol) and 4-(trifluoromethyl)aniline (120 mg, 94.0 μL, 0.75 mmol). The crude product was purified by preparative HPLC to afford the product as a yellow oil (126 mg, 0.29 mmol, 58% yield).

**$^1$H NMR (400 MHz, CDCl$_3$):** $\delta_H$ 7.52 (dd, *J* = 9.0, 5.2 Hz, 4H), 7.25 (d, *J* = 8.5, 2H), 7.05 (app t, *J* = 9.0 Hz, 4H), 6.45 (d, *J* = 8.5 Hz, 2H), 4.23 (q, *J* = 7.1 Hz, 2H), 1.12 (t, *J* = 7.1 Hz, 3H).

**$^{13}$C{$^1$H} NMR (101 MHz, CDCl$_3$):** $\delta_C$ 172.4, 162.3 (d, *J* = 248.4 Hz), 147.3, 134.4 (d, *J* = 3.4 Hz), 130.3 (d, *J* = 8.1 Hz), 126.0 (q, *J* = 3.9 Hz), 123.4 (q, 278 Hz), 119.9 (q, *J* = 32.6 Hz), 115.3 (d, *J* = 21.5 Hz), 114.8, 70.2, 62.9, 13.7.

**$^{19}$F NMR (376 MHz, CDCl$_3$):** $\delta_F$ -61.26 (s, 3H), -113.75 (tt. *J* = 9.0, 4.5 Hz, 2F).

**ῦ (ATR)/cm$^{-1}$:** 3416, 1730, 1615, 1503. 1318, 1224, 1201, 1158, 1110, 1064, 1014, 829, 578.

**HRMS** calcd for $C_{23}H_{18}F_5NNaO_2^+$: 458.1150 [M+Na]$^+$; found (ESI$^+$): 458.1134.

**Ethyl 2-((3-bromophenyl)amino)-2,2-bis(4-fluorophenyl)acetate (13)**

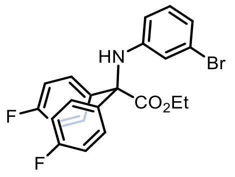


Following GP5, compound **13** was obtained from ethyl 2-(4-fluorophenyl)-2-oxoacetate **1a** (147 mg, 0.75 mmol) and 3-bromoaniline (129 mg, 82.0 μL, 0.75 mmol). The crude product was purified by preparative HPLC to afford the product as a white solid (167 mg, 0.37 mmol, 75% yield).

**m.p. /°C:** 139-141.

**$^1$H NMR (400 MHz, CDCl$_3$):** $\delta_H$ 7.62 – 7.40 (m, 4H), 7.04 (app t, *J* = 8.7 Hz, 4H), 6.92 – 6.71 (m, 2H), 6.71 – 6.55 (m, 1H), 6.55 – 6.25 (m, 1H), 5.60 (br, 1H), 4.23 (q, *J* = 7.1, 2H), 1.12 (t, *J* = 7.1 Hz, 3H).

**$^{13}$C{$^1$H} NMR (101 MHz, CDCl$_3$):** $\delta_C$ 172.5, 162.2 (d, *J* = 248.0 Hz), 146.0, 134.8 (d, *J* = 3.3 Hz), 130.3 (d, *J* = 8.1 Hz), 129.9, 122.5, 121.2, 118.5, 115.2 (d, *J* = 21.6 Hz), 114.0, 70.3, 62.8, 13.8.

**$^{19}$F-NMR (376 MHz, CDCl$_3$):** $\delta_F$ -113.94 – -114.08 (m).

**ῦ (ATR)/cm$^{-1}$:** 3390, 1724, 1591, 1503, 1478, 1249, 1228, 1197, 1160, 838, 760, 732, 545.

**HRMS** calcd for $C_{22}H_{18}BrF_2NNaO_2^+$: 468.0381 [M+Na]$^+$; found (ESI$^+$): 468.0371.

**Ethyl 2-((3-ethynylphenyl)amino)-2,2-bis(4-fluorophenyl)acetate (14)**

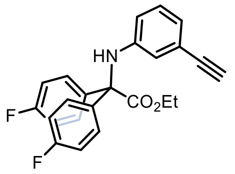


Following GP5, compound **14** was obtained from ethyl 2-(4-fluorophenyl)-2-oxoacetate **1a** (147 mg, 0.75 mmol) and 3-ethynylaniline (87.9 mg, 80.0 μL, 0.75 mmol). The crude product was purified by preparative-HPLC to afford the product as a yellow oil (88.0 mg, 0.22 mmol, 45% yield).

**$^{1}$H NMR (400 MHz, $CDCl_3$):** $\delta_H$ 7.53 (dd, *J* = 8.9, 5.2 Hz, 4H), 7.04 (app t, *J* = 8.7 Hz, 4H), 6.95 (app t, *J* = 7.9 Hz, 1H), 6.88 – 6.60 (m, 1H), 6.63 (app t, *J* = 2.5 Hz, 1H), 6.37 (ddd, *J* = 8.2, 2.5, 1.2 Hz, 1H), 5.54 (br, 1H), 4.22 (q, *J* = 7.1 Hz, 2H), 2.96 (s, 1H), 1.11 (t, *J* = 7.1 Hz, 3H).

**$^{13}$C{$^{1}$H} NMR (101 MHz, $CDCl_3$):** $\delta_C$ 172.6, 162.2 (d, *J* = 247.8 Hz), 144.7, 135.2 (d, *J* = 3.3 Hz), 130.2 (d, *J* = 8.3 Hz), 128.6, 122.34, 122.27, 119.1, 116.2, 115.2 (d, *J* = 21.4 Hz), 83.8, 76.5, 70.4, 62.7, 13.8.

**$^{19}$F NMR (376 MHz, $CDCl_3$):** $\delta_F$ -114.16 (tt, *J* = 8.3, 5.2 Hz).

**ῡ (ATR)/cm$^{-1}$:** 3395, 3295, 2982, 1728, 1600, 1504, 1262, 1161, 1015, 842, 549.

**HRMS** calcd for $C_{24}H_{19}F_2NNaO_2^+$: 414.1276 [M+Na]$^+$; found (ESI$^+$) 414.1285.

**Ethyl 2-((2,4-dimethylphenyl)amino)-2,2-bis(4-fluorophenyl)acetate (15)**

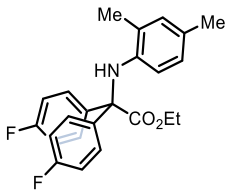


Following GP5, compound **15** was obtained from ethyl 2-(4-fluorophenyl)-2-oxoacetate **1a** (147 mg, 0.75 mmol) and 2,4-dimethylaniline (90.9 mg, 93.0 μL, 0.75 mmol). The crude product was purified by preparative HPLC to afford the product as a colourless oil (138 mg, 0.35 mmol, 70% yield).

**$^1$H NMR (400 MHz, CDCl$_3$):** $\delta_H$ 7.56 (dd, *J* = 9.0, 5.2 Hz, 4H), 7.02 (app t, *J* = 8.7 Hz, 4H), 6.91 (s, 1H), 6.59 (d, *J* = 8.2 Hz, 1H), 6.00 (d, *J* = 8.2 Hz, 1H), 5.29 (br, 1H), 4.21 (q, *J* = 7.1 Hz, 2H), 2.31 (s, 3H), 2.18 (s, 3H), 1.10 (t, *J* = 7.1 Hz, 3H).

**$^{13}$C{$^1$H} NMR (101 MHz, CDCl$_3$):** $\delta_C$ 173.2, 162.1 (d, *J* = 247.3 Hz), 140.4, 135.9 (d, *J* = 3.3 Hz), 131.0, 130.1 (d, *J* = 8.1 Hz), 127.1, 126.4, 123.5, 115.0 (d, *J* = 21.4 Hz), 114.2, 70.4, 62.4, 20.3, 17.9, 13.8.

**$^{19}$F NMR (376 MHz, CDCl$_3$):** $\delta_F$ -114.68 (tt, *J* = 8.4, 5.2 Hz).

**ῦ (ATR)/cm$^{-1}$:** 3419, 2979, 2869, 1728, 1601, 1504, 1307, 1266, 1195, 1014, 831, 731, 583.

**HRMS** calcd for $C_{24}H_{24}F_2NO_2^+$: 396.1770 [M+H]$^+$; found (ESI$^+$): 396.1752.

## 4.3 Characterization Data for N-Alkyl Amino Esters (Manuscript Scheme 2B)

**Ethyl 2,2-bis(4-fluorophenyl)-2-(propylamino)acetate (16)**

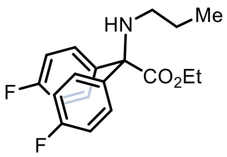


Following GP5, compound **16** was obtained from ethyl 2-(4-fluorophenyl)-2-oxoacetate **1a** (147 mg, 0.75 mmol) and propylamine (88.7 mg, 123 μL, 1.50 mmol). The crude product was purified by column chromatography (100% to 98:2 CyHex: $Et_2O$) to afford the product as a yellow oil (120 mg, 0.36 mmol, 72% yield).

**$^{1}$H NMR (400 MHz, $CDCl_3$):** $\delta_H$ 7.44 (dd, $J$ = 8.9, 5.3 Hz, 4H), 7.01 (app t, $J$ = 8.7 Hz, 4H), 4.25 (q, $J$ = 7.1 Hz, 2H), 2.26 (s, 1H), 2.20 (t, $J$ = 7.1 Hz, 2H), 1.59 – 1.48 (m, 2H), 1.23 (t, $J$ = 7.1 Hz, 3H), 0.92 (t, $J$ = 7.4 Hz, 3H).

**$^{13}$C{$^{1}$H} NMR (101 MHz, $CDCl_3$):** $\delta_C$ 173.4, 161.9 (d, $J$ = 246.9 Hz), 137.5 (d, $J$ = 3.3 Hz), 129.9 (d, $J$ = 8.0 Hz), 114.8 (d, $J$ = 21.5 Hz), 71.7, 61.7, 45.9, 23.7, 14.0, 11.8.

**$^{19}$F NMR (376 MHz, $CDCl_3$):** $\delta_F$ -115.30 (tt, $J$ = 8.7, 5.3 Hz).

**ῡ (ATR)/cm$^{-1}$:** 2960, 2931, 1727, 1602, 1504, 1472, 1223, 1191, 1028, 836, 812, 570.

**HRMS** calcd for $C_{19}H_{22}F_2NO_2^+$: 334.1613 $[M+H]^+$; found ($ESI^+$): 334.1620.

**Ethyl 2,2-bis(4-fluorophenyl)-2-(propylamino)acetate (16) – 1.0 MMOL SCALE**

A flame-dried Schlenck tube containing tetra(4-fluorophenyl)bismuthonium tetrafluoroborate **3a** (676 mg, 1.00 mmol) and ethyl 2-(4-fluorophenyl)-2-oxoacetate **1a** (294 mg, 1.50 mmol) was evacuated and backfilled with anhydrous dinitrogen three times. Anhydrous $CH_2Cl_2$ (10 mL) and *n*-propylamine (247 μL, 3.00 mmol) were added and the stirred mixture was cooled to 0 °C. $P(NMe_2)_3$ (362 μL, 2.00 mmol) was added dropwise over 2 min at 0 °C, then the cooling bath was removed and the reaction was allowed to warm to rt over 16 h. The crude reaction mixture was concentrated directly onto silica gel for dry-loading. Purification by automated column chromatography (silica gel; 100% to 98:2 CyHex: $Et_2O$) afforded **16** as a yellow oil (217 mg, 0.65 mmol, 65% yield). Characterisation data as listed above.

**Ethyl 2-(allylamino)-2,2-bis(4-fluorophenyl) acetate (17)**

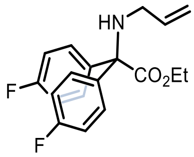

Following GP5, compound **17** was obtained from ethyl 2-(4-fluorophenyl)-2-oxoacetate **1a** (147 mg, 0.75 mmol) and propylamine (85.6 mg, 112 μL, 1.50 mmol). The crude product was purified by column chromatography (100% to 98:2 CyHex: $Et_2O$) to afford the product as a yellow oil (106 mg, 0.32 mmol, 64% yield).

**$^1$H NMR (400 MHz, $CDCl_3$):** $\delta_H$ 7.44 (dd, *J* = 9.0, 5.3 Hz, 4H), 7.02 (app t, *J* = 8.7, 4H), 5.93 (ddt, *J* = 17.2, 10.2, 5.7 Hz, 1H), 5.24 (dd, *J* = 17.2, 1.6 Hz, 1H), 5.10 (dd, *J* = 10.2, 1.6 Hz, 1H), 4.25 (q, *J* = 7.1 Hz, 2H), 2.88 (d, *J* = 5.7 Hz, 2H), 2.38 (br, 1H), 1.23 (t, *J* = 7.1 Hz, 3H).

**$^{13}$C{$^1$H} NMR (101 MHz, $CDCl_3$):** $\delta_C$ 173.2, 162.0 (d, *J* = 246.9 Hz), 137.2 (d, *J* = 3.3 Hz), 136.3, 129.9 (d, *J* = 8.0 Hz), 115.7, 114.8 (d, *J* = 21.4 Hz), 71.6, 61.8, 46.8, 14.0.

**$^{19}$F NMR (376 MHz, $CDCl_3$):** $\delta_F$ -115.04 (tt, *J* = 8.7, 5.3 Hz).

**ῡ (ATR)/cm$^{-1}$:** 3350, 1727, 1591, 1513, 1458, 1239, 1221, 1197, 1160, 838, 766, 742, 541.

**HRMS** calcd for $C_{19}H_{20}F_2NO_2^+$: 332.1457 $[M+H]^+$; found ($ESI^+$): 332.147.

**Ethyl 2,2-bis(4-fluorophenyl)-2-(phenethylamino)acetate (18)**

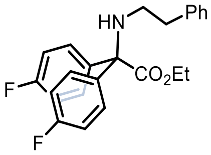


Following GP5, compound **18** was obtained from ethyl 2-(4-fluorophenyl)-2-oxoacetate **1a** (147 mg, 0.75 mmol) and phenyl ethyl amine (182 mg, 188 μL, 1.50 mmol). The crude product was purified by column chromatography (100% to 98:2 CyHex: $Et_2O$) to afford the product as a yellow oil (123 mg, 0.31 mmol, 62% yield).

**$^1$H NMR (400 MHz, CDCl$_3$):** $\delta_H$ 7.34 (dd, *J* = 8.8, 5.3 Hz, 4H), 7.31 – 7.27 (m, 2H), 7.23 – 7.18 (m, 1H), 7.17 – 7.13 (m, 2H), 6.96 (app. t, *J* = 8.5 Hz, 4H), 4.17 (q, *J* = 7.1 Hz, 2H), 2.78 (t, *J* = 6.9 Hz, 2H), 2.50 (t, *J* = 6.9 Hz, 2H), 2.34 (br, 1H), 1.15 (t, *J* = 7.1 Hz, 3H).

**$^{13}$C{$^1$H} NMR (101 MHz, CDCl$_3$):** $\delta_C$ 173.2, 161.9 (d, *J* = 246.5 Hz), 139.8, 137.3 (d, *J* = 3.5 Hz), 129.9 (d, *J* = 8.0 Hz), 128.6 (d, *J* = 37.7 Hz), 126.3, 114.9, 114.7, 71.6, 61.7, 45.2, 36.8, 14.0.

**$^{19}$F NMR (376 MHz, CDCl$_3$):** $\delta_F$ -115.17 (tt, *J* = 8.5, 5.3 Hz).

**ʋ̃ (ATR)/cm$^{-1}$:** 3346, 3026, 1727, 1601, 1505, 1447, 1227, 1200, 1026, 829, 750, 698, 565.

**HRMS** calcd for $C_{24}H_{24}F_2NO_2^+$: 396.1770 [M+H]$^+$; found (ESI$^+$): 396.1793.

**Ethyl 2,2-bis(4-fluorophenyl)-2-(isobutylamino)acetate (19)**

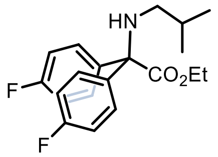


Following GP5, compound **19** was obtained from ethyl 2-(4-fluorophenyl)-2-oxoacetate **1a** (147 mg, 0.75 mmol) and isobutyl amine (110 mg, 149 µL, 1.50 mmol). The crude product was purified by column chromatography (100% to 98:2 CyHex: $Et_2O$) to afford the product as a yellow oil (104 mg, 0.30 mmol, 60% yield).

**$^1$H NMR (400 MHz, $CDCl_3$):** $\delta_H$ 7.46 (dd, *J* = 8.9, 5.2 Hz, 4H), 7.00 (app t, *J* = 8.7 Hz, 4H), 4.25 (q, *J* = 7.1 Hz, 2H), 2.26 (br, 1H), 2.07 (d, *J* = 6.6 Hz, 2H), 1.77 – 1.69 (m, 1H), 1.23 (t, *J* = 7.1 Hz, 3H), 0.93 (d, *J* = 6.6 Hz, 6H).

**$^{13}$C{$^1$H} NMR (101 MHz, $CDCl_3$):** $\delta_C$ 173.4, 161.9 (d, *J* = 246.5 Hz), 137.7 (d, *J* = 3.1 Hz), 129.8 (d, *J* = 8.1 Hz), 114.8 (d, *J* = 21.3 Hz), 71.6, 61.6, 51.7, 29.1, 20.7, 14.1.

**$^{19}$F NMR (376 MHz, $CDCl_3$):** $\delta_F$ -115.37 (tt, *J* = 8.7, 5.2 Hz,).

**ῦ (ATR)/cm$^{-1}$:** 2995, 2928, 1727, 1602, 1504, 1466, 1275, 1224, 1191, 1159, 1028, 1025, 829, 743, 565.

**HRMS** calcd for $C_{20}H_{24}F_2NO_2^+$: 348.1770 $[M+H]^+$; found ($ESI^+$): 348.1760.

**Ethyl 2,2-bis(4-fluorophenyl)-2-((2-methoxyethyl)amino)acetate (20)**

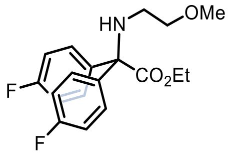


Following GP5, compound **20** was obtained from ethyl 2-(4-fluorophenyl)-2-oxoacetate **1a** (147 mg, 0.75 mmol) and 2-methoxyethylamine (113 mg, 130 μL, 1.50 mmol). The crude product was purified by column chromatography (100% to 98:2 CyHex: $Et_2O$) to afford the product as a yellow oil (105 mg, 0.30 mmol, 60% yield).

**$^1$H NMR (400 MHz, $CDCl_3$):** $\delta_H$ 7.44 (dd, *J* = 8.9, 5.3 Hz, 4H), 7.02 (app t, *J* = 8.7 Hz, 4H), 4.24 (q, *J* = 7.1 Hz, 2H), 3.51 (t, *J* = 5.2 Hz, 2H), 3.36 (s, 3H), 2.44 (t, *J* = 5.2 Hz, 2H), 1.22 (t, *J* = 7.1 Hz, 3H). *N-H signal not observed.*

**$^{13}$C{$^1$H} NMR (101 MHz, $CDCl_3$):** $\delta_C$ 173.0, 162.0 (d, *J* = 247.1 Hz), 137.1, 130.1 (d, *J* = 8.0 Hz), 114.9 (d, *J* = 21.4 Hz), 72.1, 71.6, 61.9, 58.7, 43.4, 14.0.

**$^{19}$F NMR (376 MHz, $CDCl_3$):** $\delta_F$ -115.11 (tt, *J* = 8.6, 5.3 Hz).

**ῦ (ATR)/cm$^{-1}$:** 2922, 1729, 1602, 1505, 1452, 1225, 1191, 1160, 1094, 1027, 836, 578.

**HRMS** calcd for $C_{19}H_{22}F_2NO_3^+$: 350.1562 [M+H]$^+$; found (ESI$^+$): 350.1570.

## Ethyl 2-(benzylamino)-2,2-bis(4-fluorophenyl) acetate (21)

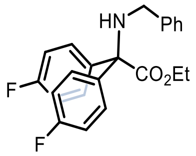


Following GP5, compound **21** was obtained from ethyl 2-(4-fluorophenyl)-2-oxoacetate **1a** (147 mg, 0.75 mmol) and benzylamine (161 mg, 164 μL, 1.50 mmol). The crude product was purified by column chromatography (100% to 98:2 CyHex: $Et_2O$) to afford the product as a yellow oil (116 mg, 0.30 mmol, 61% yield).

**$^1$H NMR (400 MHz, $CDCl_3$):** $\delta_H$ 7.52 (dd, *J* = 9.0, 5.3 Hz, 4H), 7.40 – 7.33 (m, 4H), 7.31 – 7.26 (m, 1H), 7.04 (app t, *J* = 8.7 Hz, 4H), 4.29 (q, *J* = 7.1 Hz, 2H), 3.44 (d, *J* = 5.8 Hz, 2H), 2.59 (t, *J* = 5.8 Hz, 1H), 1.26 (t, *J* = 7.1 Hz, 3H).

**$^{13}$C{$^1$H} NMR (101 MHz, $CDCl_3$):** $\delta_C$ 173.2, 162.0 (d, *J* = 246.9 Hz), 140.1, 137.3 (d, *J* = 3.4 Hz), 129.9 (d, *J* = 8.0 Hz), 128.4, 128.1, 127.1, 114.9 (d, *J* = 21.3 Hz), 71.8, 61.8, 48.4, 14.1.

**$^{19}$F NMR (376 MHz, $CDCl_3$):** $\delta_F$ -114.96 (tt, *J* = 8.5, 5.3 Hz).

**ῡ (ATR)/cm$^{-1}$:** 3029, 1727, 1601, 1504, 1454, 1224, 1188, 1015, 813, 738, 699.

**HRMS** calcd for $C_{23}H_{21}F_2NNaO_2^+$: 404.1433 [M+Na]$^+$; found (ESI$^+$): 404.1437.

**Ethyl 2,2-bis(4-fluorophenyl)-2-((furan-2-ylmethyl)amino)acetate (22)**

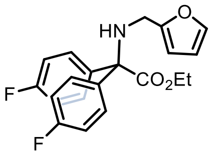


Following GP5, compound **22** was obtained from ethyl 2-(4-fluorophenyl)-2-oxoacetate **1a** (147 mg, 0.75 mmol) and furfurylamine (146 mg, 132 μL, 1.50 mmol). The crude product was purified by column chromatography (100% to 96:4 CyHex: $Et_2O$) to afford the product as a dark red solid (122 mg, 0.33 mmol, 66% yield).

**m.p. /°C:** 60 – 62.

**$^1$H NMR (400 MHz, $CDCl_3$):** $\delta_H$ 7.50 (dd, *J* = 9.0, 5.3 Hz, 4H), 7.38 (d, *J* = 1.9 Hz, 1H), 7.04 (app t, *J* = 8.7 Hz, 4H), 6.33 (dd, *J* = 3.2, 1.9 Hz, 1H), 6.19 (d, *J* = 3.2 Hz, 1H), 4.26 (q, *J* = 7.1 Hz, 2H), 3.45 (s, 2H), 2.77 (br, 1H), 1.24 (t, *J* = 7.1 Hz, 3H).

**$^{13}$C{$^1$H} NMR (101 MHz, $CDCl_3$):** $\delta_C$ 172.8, 162.1 (d, *J* = 247.3 Hz), 153.3, 141.9, 136.7, 130.1 (d, *J* = 8.1 Hz), 114.9 (d, *J* = 21.3 Hz), 110.2, 106.7, 71.5, 62.0, 41.4, 14.0.

**$^{19}$F NMR (376 MHz, $CDCl_3$):** $\delta_F$ -114.79 (tt, *J* = 8.6, 5.3 Hz).

**ῦ (ATR)/cm$^{-1}$:** 3346, 2960, 1731, 1602, 1503, 1447, 1221, 1192, 1030, 841, 811, 751, 620, 577, 506.

**HRMS** calcd for $C_{21}H_{20}F_2NO_3^+$: 372.1407 [M+H]$^+$; found (ESI$^+$): 372.1406.

**Ethyl 2,2-bis(4-fluorophenyl)-2-((thiophen-2-ylmethyl)amino)acetate (23)**

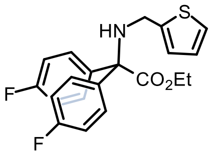


Following GP5, compound **23** was obtained from ethyl 2-(4-fluorophenyl)-2-oxoacetate **1a** (147 mg, 0.75 mmol) and 2-(aminomethyl)thiophene (170 mg, 154 μL, 1.50 mmol). The crude product was purified by column chromatography (100% to 96:4 CyHex: $Et_2O$) to afford the product as a yellow oil (99.0 mg, 0.25 mmol, 51% yield).

**$^1$H NMR (400 MHz, $CDCl_3$):** $\delta_H$ 7.50 (dd, *J* = 9.0, 5.3 Hz, 4H), 7.22 (dd, *J* = 5.1, 1.2 Hz, 1H), 7.02 (app t, *J* = 8.7 Hz, 4H), 6.95 (dd, *J* = 5.1, 3.4 Hz, 1H), 6.90 (dd, *J* = 3.4, 1.2 Hz, 1H), 4.27 (q, *J* = 7.1 Hz, 2H), 3.62 (s, 2H), 2.75 (br, 1H), 1.24 (t, *J* = 7.1 Hz, 3H).

**$^{13}$C{$^1$H} NMR (101 MHz, $CDCl_3$):** $\delta_C$ 172.9, 162.0 (d, *J* = 247.1 Hz), 143.7, 137.0 (d, *J* = 3.3 Hz), 129.9 (d, *J* = 8.0 Hz), 126.7, 124.5, 124.4, 115.0 (d, *J* = 21.3 Hz), 71.6, 61.9, 43.5, 14.1.

**$^{19}$F NMR (376 MHz, $CDCl_3$):** $\delta_F$ -114.75 (tt, *J* = 8.5, 5.3 Hz).

**ῦ (ATR)/cm$^{-1}$:** 3338, 3070, 1726, 1601, 1462, 1225, 1190, 1160, 1026, 832, 700, 566.

**HRMS** calcd for $C_{21}H_{20}F_2NO_2S^+$: 388.1177 [M+H]$^+$; found (ESI$^+$): 388.1190

**Ethyl 2,2-bis(4-fluorophenyl)-2-((pyridin-2-ylmethyl)amino)acetate (24)**

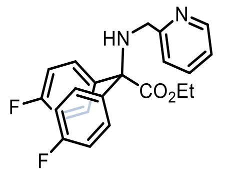


Following GP5, compound **24** was obtained from ethyl 2-(4-fluorophenyl)-2-oxoacetate **1a** (147 mg, 0.75 mmol) and 2-benzylaminopyridine (276 mg, 262 μL, 1.50 mmol). The crude product was purified by column chromatography (100% to 96:4 CyHex: $Et_2O$) to afford the product as a yellow oil (93.0 mg, 0.25 mmol, 49% yield).

**$^1$H NMR (400 MHz, $CDCl_3$):** $\delta_H$ 8.55 (d, *J* = 4.5, 1H), 7.65 (app td, *J* = 7.7, 1.8 Hz, 1H), 7.50 (dd, *J* = 8.9, 5.3 Hz, 4H), 7.31 (d, *J* = 7.7 Hz, 1H), 7.18 (dd, *J* = 7.7, 4.5 Hz, 1H), 7.03 (app t, *J* = 8.7 Hz, 4H), 4.25 (q, *J* = 7.1 Hz, 2H), 3.64 (s, 2H), 1.21 (t, *J* = 7.1 Hz, 3H).

**$^{13}$C{$^1$H} NMR (101 MHz, $CDCl_3$):** $\delta_C$ 173.0, 162.0 (d, *J* = 246.9 Hz), 159.1, 148.8, 137.1 (d, *J* = 3.1 Hz), 136.7, 130.2 (d, *J* = 8.0 Hz), 122.2, 122.0, 114.9 (d, *J* = 21.1 Hz), 71.9, 61.9, 49.4, 14.0.

**$^{19}$F NMR (376 MHz, $CDCl_3$):** $\delta_F$ -114.97 (tt, *J* = 8.4, 5.3 Hz).

**ῦ (ATR)/cm$^{-1}$:** 3054, 1728, 1599, 1544, 1473, 1450, 1225, 1161, 1015, 834, 755, 568.

**HRMS** calcd for $C_{22}H_{20}F_2N_2NaO_2^+$: 405.1385 [M+H]$^+$; found (ESI$^+$): 405.1373.

**Ethyl 2-(cyclohexylamino)-2,2-bis(4-fluorophenyl) acetate (25)**

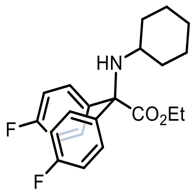


Following GP5, compound **25** was obtained from ethyl 2-(4-fluorophenyl)-2-oxoacetate **1a** (147 mg, 0.75 mmol) and cyclohexylamine (149 mg, 172 μL, 1.50 mmol). The crude product was purified by column chromatography (100% to 98:2 CyHex: $Et_2O$) to afford the product as a colourless oil (97.0 mg, 0.26 mmol, 52% yield).

**$^1$H NMR (400 MHz, $CDCl_3$):** $\delta_H$ 7.47 (dd, *J* = 9.0, 5.4 Hz, 4H), 7.00 (app t, *J* = 8.7 Hz, 4H), 4.21 (q, *J* = 7.1 Hz, 2H), 2.30 (br, 1H), 2.25 – 2.18 (m, 1H), 1.66 – 1.53 (m, 2H), 1.51 – 1.46 (m, 1H), 1.45 – 1.33 (m, 2H), 1.20 (t, *J* = 7.1 Hz, 3H), 1.08 – 0.98 (m, 5H).

**$^{13}$C{$^1$H} NMR (101 MHz, $CDCl_3$):** $\delta_C$ 174.2, 161.9 (d, *J* = 246.6 Hz), 138.5 (d, *J* = 3.0 Hz), 130.2 (d, *J* = 8.0 Hz), 114.6 (d, *J* = 21.3 Hz), 71.5, 61.6, 52.8, 35.2, 25.8, 25.4, 13.9.

**$^{19}$F NMR (376 MHz, $CDCl_3$):** $\delta_F$ -115.26 (tt, *J* = 8.6, 5.4 Hz).

**ṽ (ATR)/cm$^{-1}$:** 2926, 2952, 1726, 1601, 1448, 1224, 1159, 1096, 1027, 837, 812, 574.

**HRMS** calcd for $C_{22}H_{26}F_2NO_2^+$: 374.1926 [M+H]$^+$; found (ESI$^+$): 374.1943.

**_rac_-Ethyl 2-((1-(2,6-dimethylphenoxy)propan-2-yl)amino)-2,2-bis(4-fluorophenyl)acetate (26)**

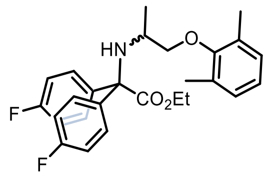


Following GP5, compound **26** was obtained from ethyl 2-(4-fluorophenyl)-2-oxoacetate **1a** (147 mg, 0.75 mmol) and *rac*-mexetiline (269 mg, 1.50 mmol). The free amine was obtained by neutralisation of the corresponding HCl salt. The crude product was purified by column chromatography (100% to 97:3 CyHex: $Et_2O$) to afford the product as a colourless oil (57.0 mg, 0.12 mmol, 25% yield).

**$^{1}$H NMR (400 MHz, $CDCl_3$):** $\delta_H$ 7.53 (dd, *J* = 9.0, 5.3 Hz, 2H), 7.47 (dd, *J* = 9.0, 5.3 Hz, 2H), 7.09 – 6.96 (m, 6H), 6.93 – 6.89 (m, 1H), 4.22 (q, *J* = 7.1 Hz, 2H), 3.39 (dd, *J* = 8.8, 6.6 Hz, 1H), 3.31 (dd, *J* = 8.8, 5.1 Hz, 1H), 2.92 (app td, *J* = 6.4, 5.1 Hz, 1H), 2.80 (s, 6H), 1.20 (t, *J* = 7.1 Hz, 3H), 1.07 (d, *J* = 6.4 Hz, 3H).

**$^{13}$C{$^{1}$H} NMR (101 MHz, $CDCl_3$):** $\delta_C$ 173.8, 162.08 (d, *J* = 247.2), 162.03 (d, *J* = 247.2 ), 155.4, 138.3 (d, *J* = 3.2 Hz), 137.8 (d, *J* = 3.3 Hz), 130.9 (d, *J* = 8.0 Hz), 130.7, 130.0 (d, *J* = 8.0 Hz), 128.8, 123.7, 114.8 (d, *J* = 21.3 Hz), 114.7 (d, *J* = 21.3 Hz), 76.5, 71.5, 61.8, 49.2, 19.5, 16.3, 13.9.

**$^{19}$F NMR (376 MHz, $CDCl_3$):** $\delta_F$ -114.88 (tt, *J* = 8.5, 5.3 Hz, 1H), -114.95 (tt, *J* = 8.5, 5.3 Hz, 1H).

**ῦ (ATR)/cm$^{-1}$:** 2980, 1754, 1624, 1550, 1436, 1393, 1380, 1216, 1257, 1128, 1002, 935, 842, 731.

**HRMS** calcd for $C_{27}H_{30}F_2NO_3^+$: 454.2188 $[M+H]^+$; found ($ESI^+$): 454.2196.

**_rac_-Ethyl 2,2-bis(4-fluorophenyl)-2-((2-phenylcyclopropyl)amino)acetate (27)**

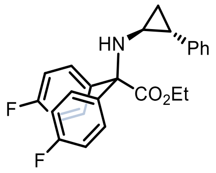


Following GP5, compound **27** was obtained from ethyl 2-(4-fluorophenyl)-2-oxoacetate **1a** (147 mg, 0.75 mmol) and *trans*-2-phenylcyclopropylamine (200 mg, 1.50 mmol). The free amine was obtained by neutralisation of the corresponding HCl salt. The crude product was purified by column chromatography (100% to 98:2 CyHex: $Et_2O$) to afford the product as a colourless oil (71.0 mg, 0.17 mmol, 35% yield).

**$^1$H NMR (400 MHz, $CDCl_3$):** $\delta_H$ δ 7.37 (dd, *J* = 8.8, 5.4 Hz, 2H), 7.32 (dd, *J* = 8.8, 5.4 Hz, 2H), 7.18 – 7.03 (m, 3H), 6.97 (app t, *J* = 8.7 Hz, 2H), 6.91 (app t, *J* = 8.7 Hz, 2H), 6.63 (d, *J* = 7.1, 2H), 4.23 (q, *J* = 7.1 Hz, 2H), 3.37 (br, 1H), 2.23 – 2.00 (m, 1H), 1.67 – 1.37 (m, 1H), 1.20 (t, *J* = 7.1 Hz, 3H), 0.92 (dd, *J* = 9.4, 4.7 Hz, 1H), 0.82 (dt, *J* = 7.5, 5.7 Hz, 1H).

**$^{13}$C{$^1$H} NMR (101 MHz, $CDCl_3$):** $\delta_C$ 173.6, 162.10 (d, *J* = 246.9 Hz), 162.08 (d, *J* = 247.0 Hz), 141.8, 137.3, 136.7, 130.8 (d, *J* = 8.0 Hz), 130.5 (d, *J* = 8.0 Hz), 127.9, 125.3, 114.8 (d, *J* = 21.5 Hz), 114.7 (d, *J* = 21.5 Hz), 72.2, 62.0, 37.8, 26.9, 26.0, 17.0, 13.9.

**$^{19}$F NMR (376 MHz, $CDCl_3$):** $\delta_F$ -115.03 (tt, *J* = 8.5, 5.4 Hz), -115.10 (tt, *J* = 8.5, 5.4 Hz).

**ṽ (ATR)/cm$^{-1}$:** 3348, 2978, 1727, 1602, 1226, 1111, 1079, 1015, 836, 812, 747, 697, 586, 560.

**HRMS** calcd for $C_{25}H_{24}F_2NO_2^+$: 408.1770 [M+H]$^+$; found (ESI$^+$): 408.1794.

**tert-butyl (2-ethoxy-1,1-bis(4-fluorophenyl)-2-oxoethyl)-L-valinate (28)**

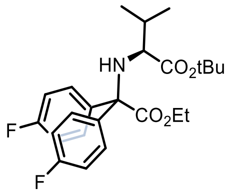


Following GP5, compound **28** was obtained from ethyl 2-(4-fluorophenyl)-2-oxoacetate **1a** (147 mg, 0.75 mmol) and *tert*-butyl L-valinate (260 mg, 1.50 mmol). The free amine was obtained by neutralisation of the corresponding HCl salt. The crude product was purified by column chromatography (100% to 98:2 CyHex: $Et_2O$) to afford the product as a colourless oil (72.0 mg, 0.16 mmol, 32% yield).

**$^{1}$H NMR (400 MHz, $CDCl_3$):** $\delta_H$ 7.46 – 7.37 (m, 4H), 6.99 (t, *J* = 8.8, 2H), 6.98 (t, *J* = 8.8, 2H), 4.23 – 4.10 (m, 2H), 3.15 (br, 1H), 3.00 (d, *J* = 4.1 Hz, 1H), 2.36 – 1.83 (m, 1H), 1.33 (s, 9H), 1.17 (t, *J* = 7.1 Hz, 3H), 0.93 (d, *J* = 7.0 Hz, 3H), 0.90 (d, *J* = 7.0 Hz, 3H).

**$^{13}$C{$^{1}$H} NMR (101 MHz, $CDCl_3$):** $\delta_C$ 173.5, 173.0, 162.0 (d, *J* = 237.3 Hz), 161.9 (d, *J* = 237.3 Hz), 138.5 (d, *J* = 3.3 Hz), 137.7 (d, *J* = 3.3 Hz), 130.9 (d, *J* = 8.0 Hz), 130.1 (d, *J* = 8.0 Hz), 114.7 (d, *J* = 21.3 Hz), 114.6 (d, *J* = 21.3 Hz), 80.8, 71.4, 61.6, 61.4, 33.6, 28.1, 19.0, 18.4, 13.9.

**$^{19}$F NMR (376 MHz, $CDCl_3$):** $\delta_F$ -115.02 (tt, *J* = 8.5, 5.3 Hz), -115.52 (tt, *J* = 8.5, 5.3 Hz).

**ῦ (ATR)/cm$^{-1}$:** 2974, 1723, 1604, 1505, 1466, 1391, 1367, 1226, 1157, 1028, 908, 835, 812, 731, 566.

**HRMS** calcd for $C_{25}H_{32}F_2NO_4^+$: 448.2294 [M+H]$^+$; found (ESI$^+$): 448.2306

$[\alpha]_D^{18}$ = -105.9 (c = 0.09, $CHCl_3$)

**Benzyl (2-ethoxy-1,1-bis(4-fluorophenyl)-2-oxoethyl)-L-prolinate (29)**

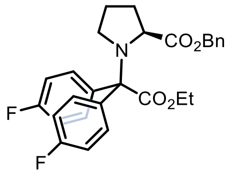


Following GP5, compound **29** was obtained from ethyl 2-(4-fluorophenyl)-2-oxoacetate **1a** (147 mg, 0.75 mmol) and benzyl L-prolinate (307 mg, 1.50 mmol). The crude product was purified by column chromatography (100% to 96:4 CyHex: $Et_2O$) to afford the product as a colourless oil (76.0 mg, 0.16 mmol, 32% yield).

**$^1$H NMR (400 MHz, $CDCl_3$):** $\delta_H$ 7.57 (dd, *J* = 8.9, 5.4 Hz, 2H), 7.44 – 7.24 (m, 7H), 7.01 (app t, *J* = 8.7 Hz, 2H), 6.84 (app t, *J* = 8.7 Hz, 2H), 5.16 – 4.98 (m, 2H), 4.55 – 4.09 (m, 2H), 3.98 (dd, *J* = 8.3, 3.4 Hz, 1H), 3.30 (ddd, *J* = 10.0, 6.8, 3.7 Hz, 1H), 2.85 – 2.46 (m, 1H), 2.38 – 1.73 (m, 3H), 1.71 – 1.59 (m, 1H), 1.26 (t, *J* = 7.1 Hz, 3H).

**$^{13}$C{$^1$H} NMR (101 MHz, $CDCl_3$):** $\delta_C$, 176.0, 172.3, 161.8 (d, *J* = 247.5 Hz), 161.71 (d, *J* = 246.7 Hz), 137.6 (d, *J* = 3.4 Hz), 136.5 (d, *J* = 3.4 Hz), 136.0, 130.5 (d, *J* = 8.0 Hz), 130.5 (d, *J* = 8.0 Hz), 128.5, 128.4, 128.4, 114.8 (d, *J* = 21.7 Hz), 114.6 (d, *J* = 21.7 Hz), 66.2, 62.5, 61.5, 50.4, 31.8, 24.6, 14.1.

**$^{19}$F NMR (376 MHz, $CDCl_3$):** $\delta_F$ -114.76 (tt, *J* = 8.5, 5.3 Hz), -115.67 (tt, *J* = 8.5, 5.3 Hz).

**ῦ (ATR)/cm$^{-1}$:** 3390, 3317, 1726, 1600, 1504, 1229, 1159, 1015, 810, 571, 526.

**HRMS** calcd for $C_{28}H_{28}F_2NO_4^+$: 480.1981 $[M+H]^+$; found ($ESI^+$): 480.1979

$[\alpha]_D^{18}$ = - 50.7 (c = 0.08, $CHCl_3$)

## 4.4 Characterization Data for α,α-Aryl,Aryl′ Amino Esters (Manuscript Schemes 2C & 3)

**Ethyl 2-(4-ethoxyphenyl)-2-(4-fluorophenyl)-2-(propylamino)acetate (30)**

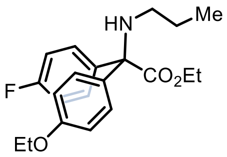


Following GP5, compound **30** was obtained from ethyl 2-(4-ethoxyphenyl)-2-oxoacetate (167 mg, 0.75 mmol) and propylamine (88.7 mg, 123 μL, 1.50 mmol). The crude product was purified by column chromatography (100% to 98:2 CyHex: $Et_2O$) to afford the product as a colourless oil (72 mg, 0.2 mmol, 40% yield).

**$^1$H NMR (400 MHz, $CDCl_3$):** $\delta_H$ 7.47 (dd, *J* = 8.9, 5.3 Hz, 2H), 7.33 (d, *J* = 8.9 Hz, 2H), 7.00 (app t, *J* = 8.7 Hz, 2H), 6.84 (d, *J* = 8.9 Hz, 2H), 4.23 (q, *J* = 7.1 Hz, 2H), 4.04 (q, *J* = 7.0 Hz, 2H), 2.20 (t, *J* = 7.4 Hz, 2H), 1.53 (q, *J* = 7.4 Hz, 2H), 1.43 (t, *J* = 7.0 Hz, 3H), 1.22 (t, *J* = 7.1 Hz, 3H), 0.91 (t, *J* = 7.4 Hz, 3H).

**$^{13}$C{$^1$H} NMR (101 MHz, $CDCl_3$):** $\delta_C$ 173.8, 161.8 (d, *J* = 246.2 Hz), 158.0, 137.7 (d, *J* = 3.2 Hz), 133.7, 130.2 (d, *J* = 8.0 Hz), 129.3, 114.5 (d, *J* = 21.3 Hz), 113.7, 71.7, 63.8, 61.5, 45.9, 23.7, 14.9, 14.0, 11.8.

**$^{19}$F NMR (376 MHz, $CDCl_3$):** $\delta_F$ -115.77 (tt, *J* = 8.5, 5.3 Hz).

**ῦ (ATR)/cm$^{-1}$:** 2960, 1726, 1605, 1505, 1476, 1460, 1298, 1224, 1177, 1160, 1046, 836, 732, 566.

**HRMS** calcd for $C_{21}H_{26}FNO_3^+$: 360.1969 [M+H]$^+$; found (ESI$^+$): 360.1967.

**Ethyl 2-(4-fluorophenyl)-2-(propylamino)-2-(*p*-tolyl)acetate (31)**

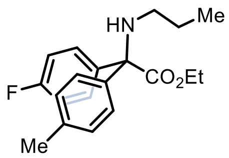


Following GP5, compound **31** was obtained from ethyl 2-oxo-2-(p-tolyl)acetate (144 mg, 0.75 mmol) and propylamine (88.7 mg, 123 μL, 1.50 mmol). The crude product was purified by column chromatography (100% to 98:2 CyHex: $Et_2O$) to afford the product as a colourless oil (102 mg, 0.31 mmol, 62% yield).

**$^1$H NMR (400 MHz, $CDCl_3$):** $\delta_H$ 7.50 (dd, *J* = 8.9, 5.4 Hz, 2H), 7.31 (d, *J* = 8.2 Hz, 2H), 7.14 (d, *J* = 8.2 Hz, 2H), 7.00 (app t, *J* = 8.7 Hz, 2H), 4.24 (q, *J* = 7.1 Hz, 2H), 2.35 (s, 3H), 2.22 (t, *J* = 7.4 Hz, 2H), 1.54 (q, *J* = 7.4 Hz, 2H), 1.23 (t, *J* = 7.1 Hz, 3H), 0.92 (t, *J* = 7.4 Hz, 3H).

**$^{13}$C{$^1$H} NMR (101 MHz, $CDCl_3$):** $\delta_C$ 173.7, 161.8 (d, *J* = 246.1 Hz), 139.0, 137.6 (d, *J* = 3.2 Hz), 137.0, 130.2 (d, *J* = 8.0 Hz), 128.7, 127.9, 114.5 (d, *J* = 21.3 Hz), 72.0, 61.5, 45.9, 23.7, 21.0, 14.1, 11.9.

**$^{19}$F NMR (376 MHz, $CDCl_3$):** $\delta_F$ -115.76 (tt, *J* = 8.5, 5.4 Hz).

**ῦ (ATR)/cm$^{-1}$:** 2969, 1727, 1603, 1505, 1460, 1224, 1182, 1094, 908, 839, 817, 731, 645, 572.

**HRMS** calcd for $C_{20}H_{24}FNO_2^+$: 330.1864 [M+H]$^+$; found (ESI$^+$): 330.1877.

**Ethyl 2-(4-(*tert*-butyl)phenyl)-2-(4-fluorophenyl)-2-(propylamino)acetate (32)**

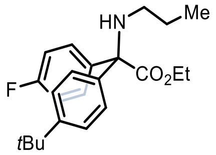


Following GP5, compound **32** was obtained from ethyl 2-(4-(*tert*-butyl)phenyl)-2-oxoacetate (175 mg, 0.75 mmol) and propylamine (88.7 mg, 123 μL, 1.50 mmol). The crude product was purified by column chromatography (100% to 98:2 CyHex: $Et_2O$) to afford the product as a yellow oil (113 mg, 0.31 mmol, 61% yield).

**$^1$H NMR (400 MHz, $CDCl_3$):** $\delta_H$. 7.50 (dd, *J* = 8.9, 5.4 Hz, 2H), 7.34 – 7.30 (br s, 4H), 7.00 (app t, *J* = 8.7 Hz, 2H), 4.25 (q, *J* = 7.1 Hz, 2H), 2.25 (br, 1H), 2.22 (t, *J* = 7.1 Hz, 2H), 1.54 (q, *J* = 7.4 Hz, 2H), 1.33 (s, 9H), 1.23 (t, *J* = 7.1 Hz, 3H), 0.92 (t, *J* = 7.4 Hz, 3H).

**$^{13}$C{$^1$H} NMR (101 MHz, $CDCl_3$):** $\delta_C$ 173.8, 161.8 (d, *J* = 246.0 Hz), 150.0, 138.8, 137.6 (d, *J* = 3.2 Hz), 130.2 (d, *J* = 8.0 Hz), 127.5, 124.9, 114.5 (d, *J* = 21.2 Hz), 72.0, 61.5, 45.9, 34.4, 31.3, 23.7, 14.1, 11.9.

**$^{19}$F-NMR (376 MHz, $CDCl_3$):** $\delta_F$ -115.83 (tt, *J* = 8.6, 5.4 Hz).

**ʋ̃ (ATR)/cm$^{-1}$:** 2960, 2870, 1728, 1505, 1461, 1223, 1187, 1159, 1094, 1030, 909, 839, 732, 582.

**HRMS** calcd for $C_{23}H_{31}FNO_2^+$: 372.2333 [M+H]$^+$; found (ESI$^+$) 372.2336.

**Ethyl 2-(4-chlorophenyl)-2-(4-fluorophenyl)-2-(propylamino)acetate (33)**

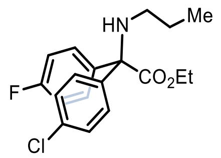


Following GP5, compound **33** was obtained from ethyl 2-(4-chlorophenyl)-2-oxoacetate (159 mg, 0.75 mmol) and propylamine (88.7 mg, 123 μL, 1.50 mmol). The crude product was purified by column chromatography (100% to 98:2 CyHex: $Et_2O$) to afford the product as a colourless oil (119 mg, 0.34 mmol, 68% yield).

**$^{1}$H NMR (400 MHz, $CDCl_3$):** $\delta_H$ δ 7.52 – 7.37 (m, 4H), 7.30 (d, *J* = 8.8 Hz, 2H), 7.01 (app t, *J* = 8.7 Hz, 2H), 4.25 (q, *J* = 7.1 Hz, 2H), 2.20 (t, *J* = 7.1 Hz, 2H), 1.54 (q, *J* = 7.3 Hz, 2H), 1.23 (t, *J* = 7.1 Hz, 3H), 0.92 (t, *J* = 7.4 Hz, 3H).

**$^{13}$C{$^{1}$H} NMR (101 MHz, $CDCl_3$):** $\delta_C$ 173.2, 161.9 (d, *J* = 246.8 Hz), 140.3, 137.2 (d, *J* = 3.3 Hz), 133.1 129.9 (d, *J* = 8.0 Hz), 129.6, 128.1, 114.8 (d, *J* = 21.3 Hz), 71.7, 61.8, 45.9, 23.7, 14.0, 11.8.

**$^{19}$F NMR (376 MHz, $CDCl_3$):** $\delta_F$ -115.14 (tt, *J* = 8.5, 5.3 Hz).

**ῦ (ATR)/cm$^{-1}$:** 2960, 2930, 2728, 1603, 1506, 1489, 1461, 1224, 1160, 1122, 1092, 1014, 861, 820, 732 566, 529.

**HRMS** calcd for $C_{19}H_{21}ClFNNaO_2^+$: 372.1137 [M+Na]$^+$; found (ESI$^+$): 372.1147.

**Ethyl 2-(4-cyanophenyl)-2-(4-fluorophenyl)-2-(propylamino)acetate (34)**

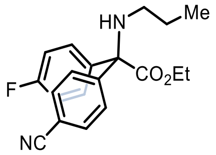


Following GP5, compound **34** was obtained from ethyl 2-(4-cyanophenyl)-2-oxoacetate (152 mg, 0.75 mmol) and propylamine (88.7 mg, 123 μL, 1.50 mmol). The crude product was purified by column chromatography (100% to 98:2 CyHex: $Et_2O$) to afford the product as a colourless oil (59 mg, 0.17 mmol, 35% yield).

**$^1$H NMR (400 MHz, $CDCl_3$):** $\delta_H$ δ 7.70 – 7.53 (m, 4H), 7.42 (dd, *J* = 8.9, 5.3 Hz, 2H), 7.02 (app t, *J* = 8.7 Hz, 2H), 4.27 (q, *J* = 7.1 Hz, 2H), 2.20 (t, *J* = 7.4 Hz, 2H), 1.55 (q, *J* = 7.3 Hz, 2H), 1.28 (br, 1H), 1.24 (t, *J* = 7.1 Hz, 3H), 0.94 (t, *J* = 7.3 Hz, 3H).

**$^{13}$C{$^1$H} NMR (101 MHz, $CDCl_3$):** $\delta_C$ 172.6, 162.0 (d, *J* = 247.5 Hz), 147.1, 136.8 (d, *J* = 3.4 Hz), 131.8, 129.6 (d, *J* = 8.1 Hz), 128.9, 118.7, 115.1 (d, *J* = 21.4 Hz), 111.2, 72.1, 62.0, 45.9, 23.6, 14.0, 11.8.

**$^{19}$F NMR (376 MHz, $CDCl_3$):** $\delta_F$ -114.45 (tt, *J* = 8.3, 5.2 Hz).

**ῦ (ATR)/cm$^{-1}$:** 2961, 2941, 2228, 1728, 1605, 1505, 1461, 1224, 1194, 1160, 1017, 825, 732, 576.

**HRMS** calcd for $C_{20}H_{22}FN_2O_2^+$: 341.1660 [M+H]$^+$; found (ESI$^+$): 341.1663.

**Ethyl 2-(3-bromo-4-methoxyphenyl)-2-(4-fluorophenyl)-2-(propylamino)acetate (35)**

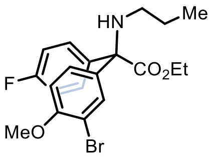


Following GP5, compound **35** was obtained from ethyl 2-(4-(*tert*-butyl)phenyl)-2-oxoacetate (264 mg, 0.75 mmol) and propylamine (88.7 mg, 123 μL, 1.50 mmol). The crude product was purified by column chromatography (100% to 98:2 CyHex: $Et_2O$) to afford the product as a colourless oil (80 mg, 0.20 mmol, 40% yield).

**$^1$H NMR (400 MHz, $CDCl_3$):** $\delta_H$ 7.68 (d, *J* = 2.4 Hz, 1H), 7.43 (dd, *J* = 8.8, 5.5 Hz, 2H), 7.37 (dd, *J* = 8.7, 2.4 Hz, 1H), 7.01 (app t, *J* = 8.7 Hz, 2H), 6.85 (d, *J* = 8.7 Hz, 1H), 4.25 (q, *J* = 7.1 Hz, 2H), 3.90 (s, 3H), 2.27 – 2.10 (m, 3H), 1.54 (q, *J* = 7.4 Hz, 2H), 1.23 (t, *J* = 7.1 Hz, 3H), 0.93 (t, *J* = 7.4 Hz, 3H).

**$^{13}$C{$^1$H} NMR (101 MHz, $CDCl_3$):** $\delta_C$, 173.3, 161.9 (d, *J* = 246.7 Hz), 155.0, 137.4 (d, *J* = 3.3 Hz), 135.4, 133.0, 129.8 (d, *J* = 8.0 Hz), 128.4, 114.8 (d, *J* = 21.3 Hz), 111.13, 111.10, 71.3, 61.7, 56.2, 45.9, 23.7, 14.1, 11.9.

**$^{19}$F NMR (376 MHz, $CDCl_3$):** $\delta_F$ -115.20 (tt, *J* = 8.8, 5.3 Hz).

**$\tilde{\nu}$ (ATR)/cm$^{-1}$:** 3410, 2958, 2923, 1727, 1604, 1506, 1460, 1224, 1186, 1159, 1027, 838, 814.

**HRMS** calcd for $C_{20}H_{24}BrFNO_3^+$: 424.0918 [M+H]$^+$; found (ESI$^+$): 424.0911.

**Ethyl 2-(4-fluorophenyl)-2-(naphthalen-2-yl)-2-(propylamino)acetate (36)**

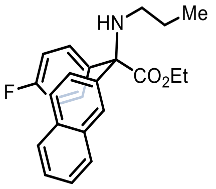


Following GP5, compound **36** was obtained from ethyl 2-(naphthalen-1-yl)-2-oxoacetate (171 mg, 0.75 mmol) and propylamine (88.7 mg, 123 μL, 1.50 mmol). The crude product was purified by column chromatography (100% to 98:2 CyHex: $Et_2O$) to afford the product as a colourless oil (55 mg, 0.15 mmol, 30% yield).

**$^1$H NMR (400 MHz, $CDCl_3$):** $\delta_H$ 8.02 (d, *J* = 1.9 Hz, 1H), 7.87 – 7.80 (m, 2H), 7.78 (d, *J* = 8.7 Hz, 1H), 7.62 – 7.42 (m, 5H), 7.02 (app t, *J* = 8.7 Hz, 2H), 4.27 (q, *J* = 7.1 Hz, 2H), 2.40 (br, 1H), 2.27 (t, *J* = 7.1 Hz, 2H), 1.57 (q, *J* = 7.4 Hz, 2H), 1.23 (t, *J* = 7.1 Hz, 3H), 0.93 (t, *J* = 7.4 Hz, 3H).

**$^{13}$C{$^1$H} NMR (101 MHz, $CDCl_3$):** $\delta_C$,173.5, 161.9 (d, *J* = 246.5 Hz), 139.2, 137.3 (d, *J* = 3.3 Hz), 132.9, 132.5, 130.3 (d, *J* = 8.0 Hz), 128.4, 127.6, 127.4, 126.6, 126.5, 126.2, 126.1, 114.7 (d, *J* = 21.2 Hz), 72.3, 61.7, 46.0, 23.7, 14.1, 11.9.

**$^{19}$F NMR (376 MHz, $CDCl_3$):** $\delta_F$ -115.42 (tt, *J* = 8.5, 5.3 Hz).

**ῦ (ATR)/cm$^{-1}$:** 3344, 3055, 2959, 1726, 1601, 1504, 1460, 1223, 1144, 1030, 817, 748, 734, 649, 478.

**HRMS** calcd for $C_{23}H_{25}FNO_2^+$: 366.1864 [M+H]$^+$; found (ESI$^+$): 366.1875.

**Ethyl 2-(4-fluorophenyl)-2-(4-methoxyphenyl)-2-(propylamino)acetate (37)**

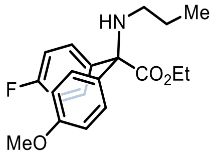


Following GP5, compound **37** was obtained from ethyl 2-(4-fluorophenyl)-2-oxoacetate **1a** (147 mg, 0.75 mmol), propylamine (88.7 mg, 123 μL, 1.50 mmol) and tetra(4-methoxyphenyl)bismuthonium tetrafluoroborate (361 mg, 0.50 mmol) The crude product was purified by column chromatography (100% to 98:2 CyHex: $Et_2O$) to afford the product as a colourless oil (80 mg, 0.2 mmol, 40% yield). The yield determined by $^{19}F$ NMR spectroscopy (*vs* $PhCF_3$) prior to purification was 60%.

**$^{1}$H NMR (500 MHz, $CDCl_3$):** $\delta_H$ 7.48 (dd, *J* = 8.4, 5.3 Hz, 2H), 7.36 (d, *J* = 8.4 Hz, 2H), 7.01 (app t, *J* = 8.7 Hz, 2H), 6.86 (d, *J* = 8.9 Hz, 2H), 4.24 (q, *J* = 7.1 Hz, 2H), 3.82 (s, 3H), 2.23 (m, 2H), 1.55 (d, *J* = 7.2 Hz, 2H), 1.22 (t, *J* = 7.1 Hz, 3H), 0.91 (t, *J* = 7.4 Hz, 3H).

**$^{13}$C{$^{1}$H} NMR (126 MHz, $CDCl_3$):** $\delta_C$,173.6, 161.9 (d, *J* = 247.5 Hz), 158.8, 137.6, 133.8, 130.2, 129.4, 114.7 (d, *J* = 21.3 Hz), 113.3, 71.9, 61.7, 55.2, 46.1, 23.5, 14.0, 11.8.

**$^{19}$F NMR (376 MHz, $CDCl_3$):** -115.75 (tt, *J* = 8.6, 5.3 Hz).

**$\tilde{\upsilon}$ (ATR)/cm$^{-1}$:** 2958, 2929, 2872, 1726, 1605, 1505, 1461, 1249, 1223, 1195, 1176, 1030, 824, 798, 582.

**HRMS** calcd for $C_{20}H_{25}FNO_3^+$: 346.1813 [M+H]$^+$; found (ESI$^+$): 346.1814.

## 4.4 Characterization Data for *N*-Unsubstituted Amino Esters (Manuscript Scheme 4)

**Ethyl 2-amino-2,2-bis(4-fluorophenyl)acetate (38)**

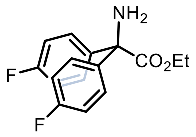


Following GP5, compound **38** was obtained from ethyl 2-(4-fluorophenyl)-2-oxoacetate **1a** (147 mg, 0.75 mmol), and ammonia solution (0.4 M in 1,4-dioxane; 266 µL, 1.50 mmol). The crude product was purified by column chromatography (8:2 CyHex: EtOAc) to afford the product as a colourless oil (54.0 mg, 0.18 mmol, 37% yield).

**$^1$H NMR (400 MHz, CDCl$_3$):** $\delta_H$ δ 7.38 (dd, *J* = 9.0, 5.3 Hz, 4H), 7.03 (app t, *J* = 8.7 Hz, 4H), 4.28 (q, *J* = 7.1 Hz, 2H), 2.30 (s, 2H), 1.26 (t, *J* = 7.1 Hz, 3H).

**$^{13}$C{$^1$H} NMR (101 MHz, CDCl$_3$):** $\delta_C$, 174.5, 162.1 (d, *J* = 247.0 Hz), 139.5 (d, *J* = 3.3 Hz), 129.3 (d, *J* = 8.1 Hz), 115.0 (d, *J* = 21.4 Hz), 67.4, 62.2, 14.0.

**$^{19}$F NMR (376 MHz, CDCl$_3$):** $\delta_F$ -114.89 (tt, *J* = 8.6, 5.3 Hz).

**ῦ (ATR)/cm$^{-1}$:** 3390, 2981, 2927, 1726, 1600, 1504, 1221, 1159, 1086, 1015, 810, 609, 571, 526.

**HRMS** calcd for $C_{16}H_{16}F_2NO_2^+$: 292.1144 $[M+H]^+$; found (ESI$^+$): 292.1159.

## 5. Product Derivatizations (Manuscript Scheme 4)

### 5.1 *N*-Debenzylation

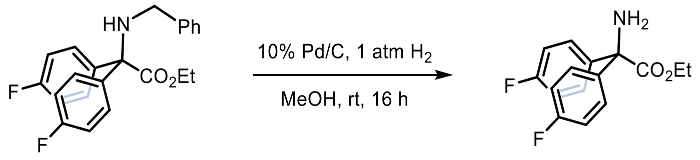


Modification of a literature procedure:[13] A stirred suspension of ethyl 2-(benzylamino)-2,2-bis(4-fluorophenyl)acetate **21** (91.5 mg, 0.25 mmol) and Pd/C (10 wt%; 25 mg) in MeOH (3 mL) was placed under an atmosphere of $H_2$ using a balloon. The reaction was stirred at ambient temperature for 16 h, then filtered through a pad of Celite. The solvent was removed under reduced pressure to yield product **38** as a colourless oil (73.0 mg, 0.25 mmol, quant.).

For characterization data, see Supplementary Section 4.4.

### 5.2 *N*-Deallylation

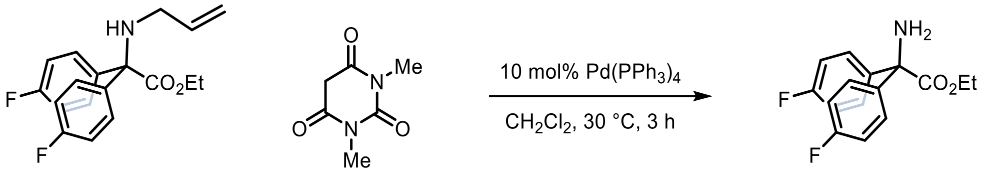


Modification of a literature procedure:[14] A solution of ethyl 2-(allylamino)-2,2-bis(4-fluorophenyl)acetate **17** (83 mg, 0.25 mmol), 1,3-dimethylbarbituric acid (117 mg, 0.75 mmol) and $Pd(PPh_3)_4$ (29.0 mg, 0.025 mmol) in $CH_2Cl_2$ (1.5 mL) was stirred at 30 °C for 3 h. The mixture was filtered through a pad of Celite, and the filtrate was evaporated onto silica gel. Purification by column chromatography (8:2 to 6:4 CyHex : EtOAc + 1% $Et_3N$) afforded product **38** as a colourless oil (67.0 mg, 0.23 mmol, 92% yield).

For characterization data, see Supplementary Section 4.4.

## 5.3 *N*-De-*para*-methoxyphenylation

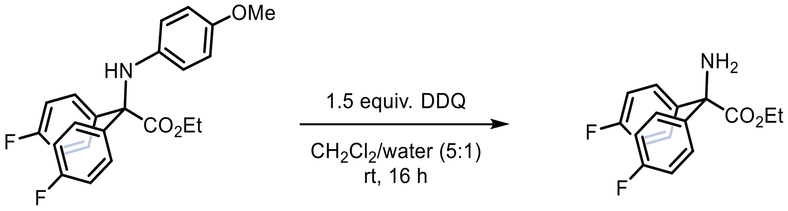


2,3-Dichloro-5,6-dicyano-1,4-benzoquinone (68.0 mg, 0.30 mmol) was added to a stirred mixture of ethyl 2,2-bis(4-fluorophenyl)-2-((4-methoxyphenyl)amino)acetate **9** (80.0 mg, 0.20 mmol) in $CH_2Cl_2$ (1.7 mL) and water (0.3 mL). The reaction was stirred at room temperature for 16 h, then saturated aqueous $NaHCO_3$ (3 mL) was added and the phases were separated. The aqueous portion was extracted with $CH_2Cl_2$ (3 × 5mL), and the combined organic portions were evaporated onto silica gel. Purification by column chromatography (8:2 to 6:4 CyHex : EtOAc + 1% $Et_3N$) afforded product **38** as a colourless oil (35.0 mg, 0.12 mmol).

For characterization data, see Supplementary Section 4.4.

# 6. Limitations of Substrate Scope

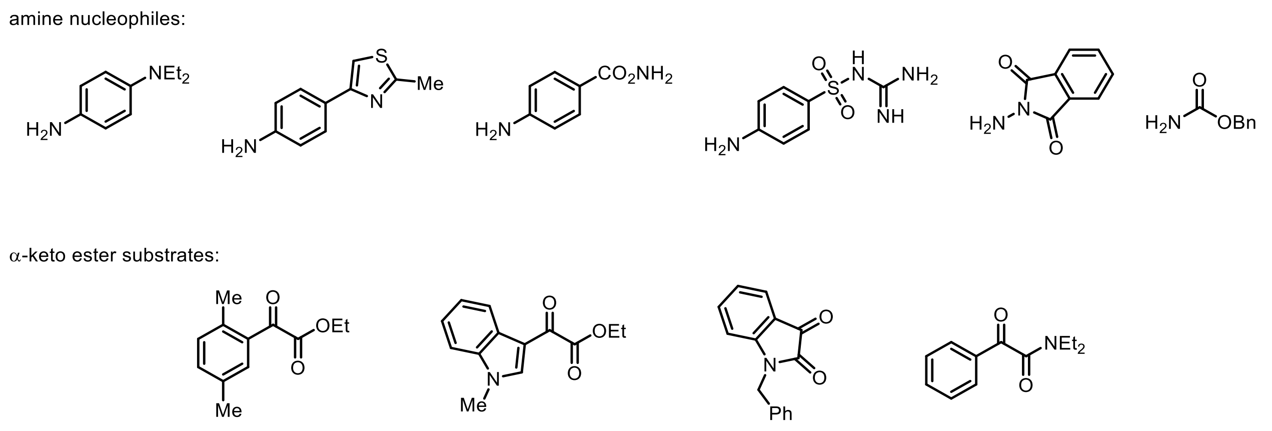


**Scheme S1:** Reactants that afforded the expected products in <5% yield under the standard conditions (see GP5), as determined by NMR spectroscopic analysis *vs* internal standard.

# 7. NMR Spectra

**Tri(4-fluorophenyl)bismuthine - $^{1}H$ NMR (500 MHz, $CDCl_3$):**

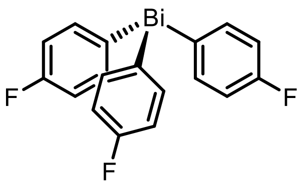

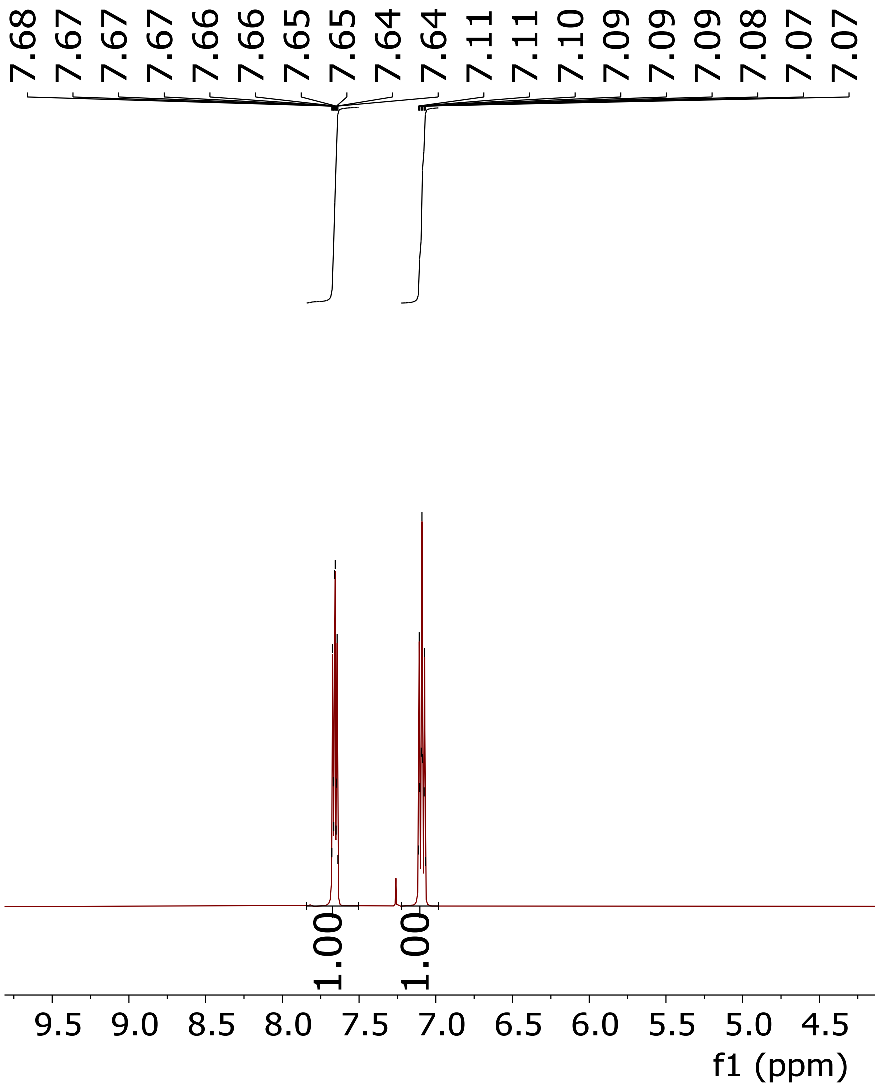

**Tri(4-fluorophenyl)bismuthine - $^{13}C\{^{1}H\}$ NMR (126 MHz, $CDCl_3$):**

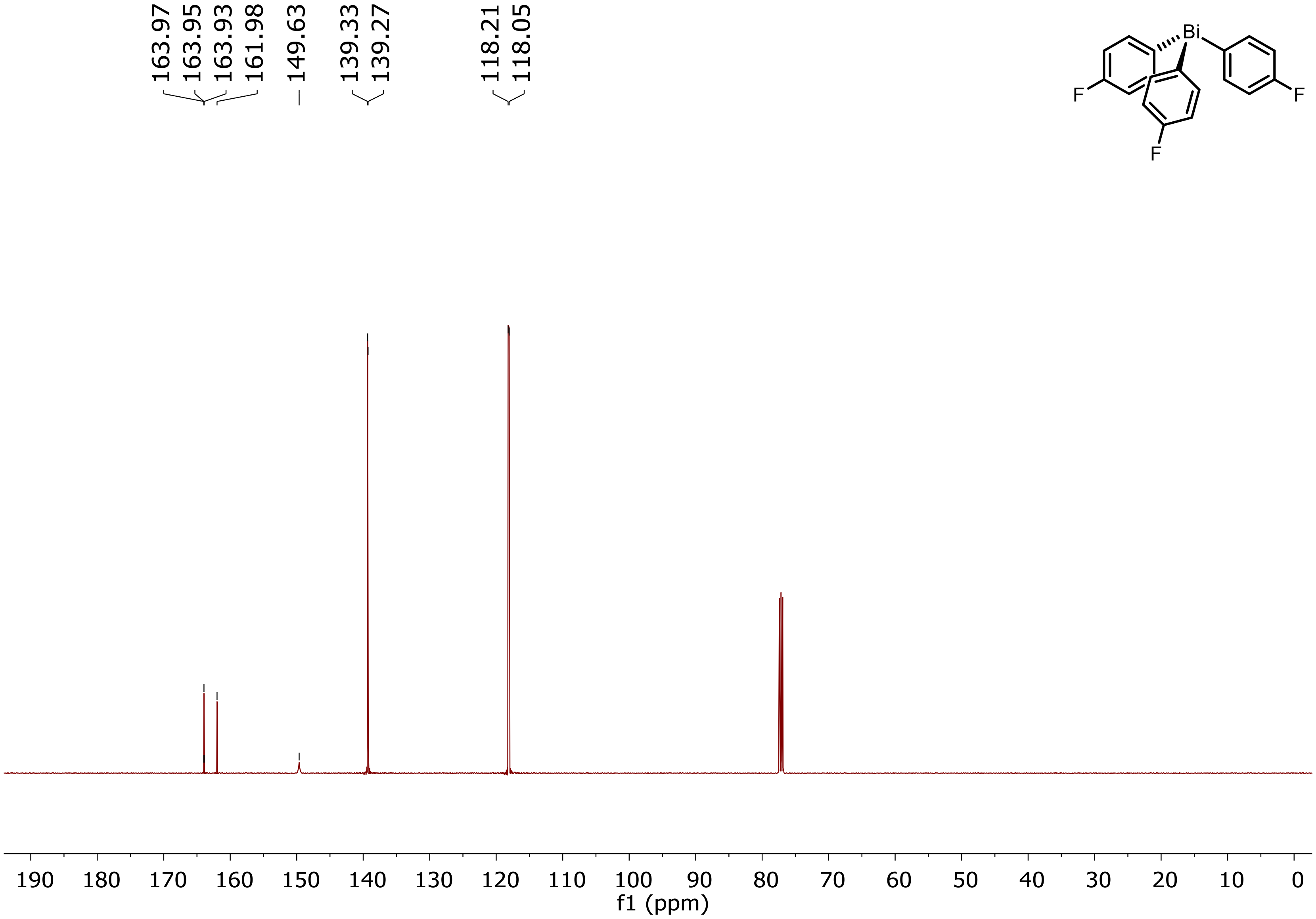

**Tri(4-fluorophenyl)bismuthine - $^{19}F$ NMR (471 MHz, $CDCl_3$):**

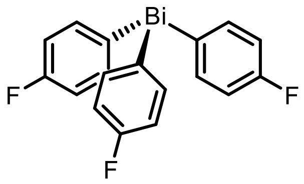


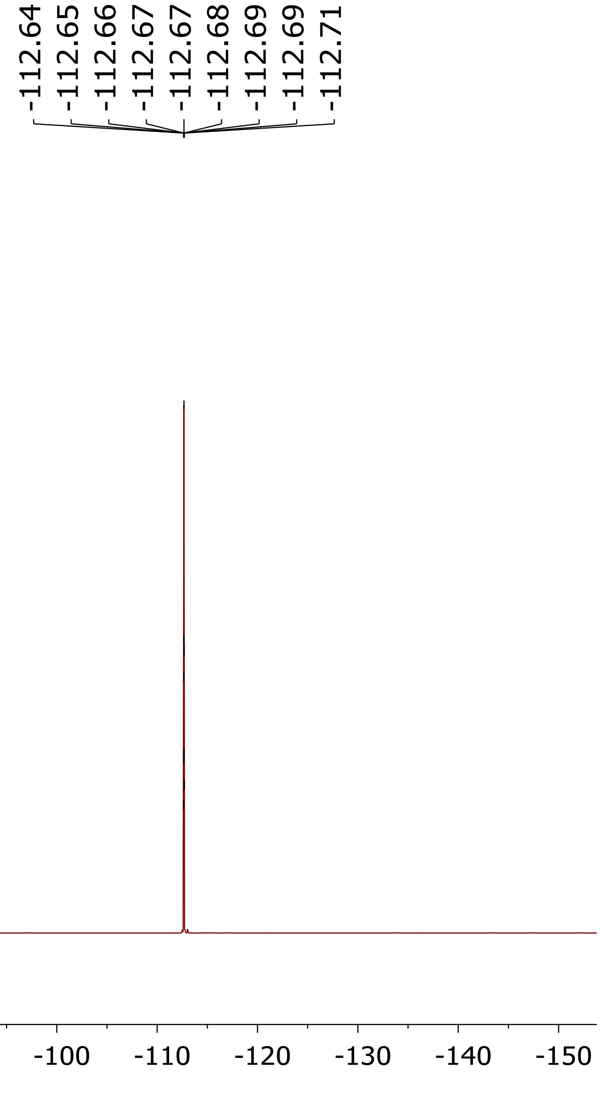

**Tri(4-methylphenyl)bismuthine - $^1$H NMR (500 MHz, $CDCl_3$):**

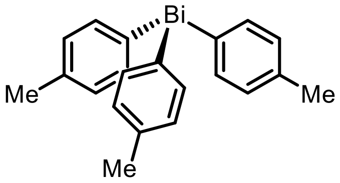

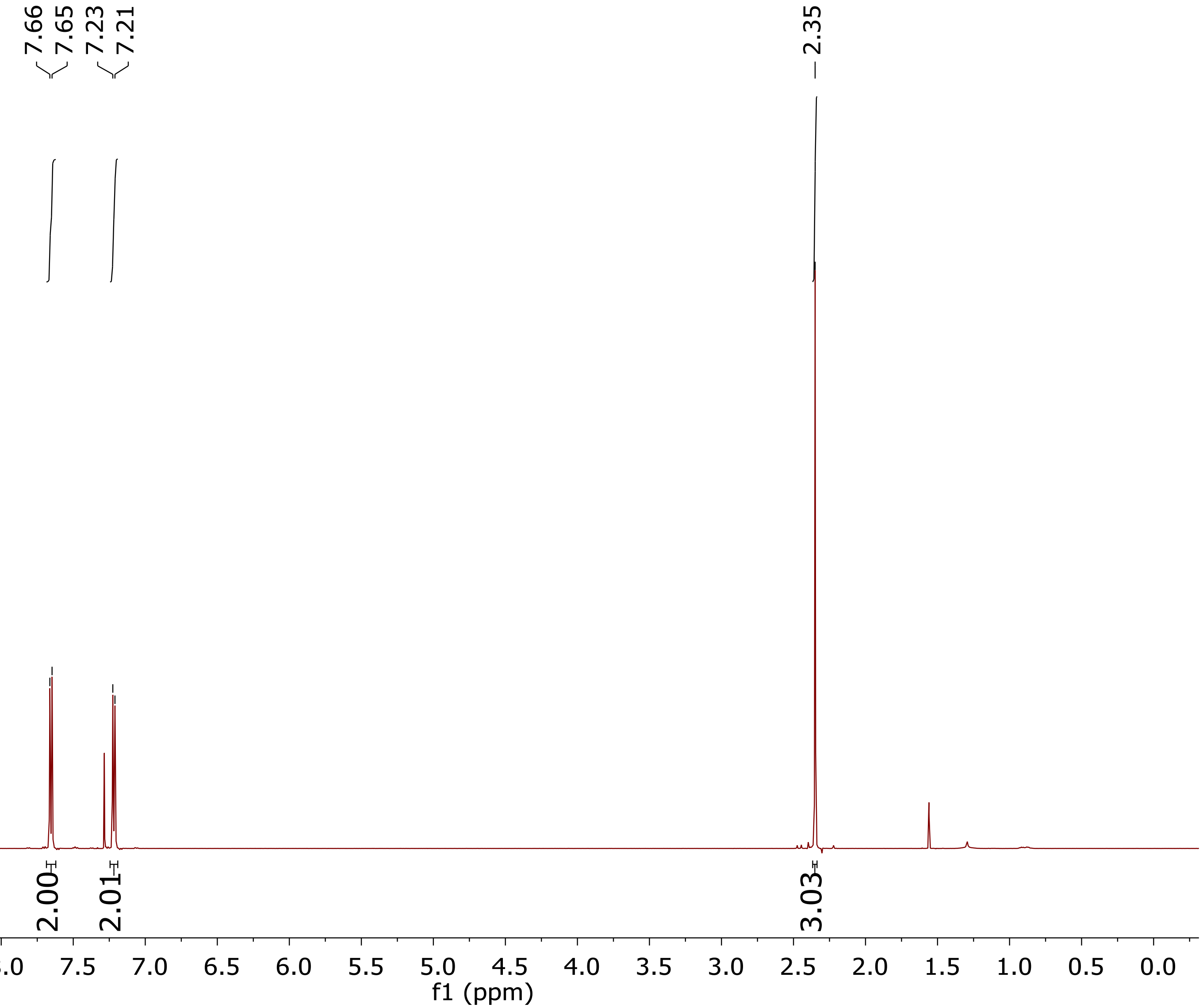

**Tri(4-methylphenyl)bismuthine - $^{13}C\{^{1}H\}$ NMR (126 MHz, $CDCl_3$):**

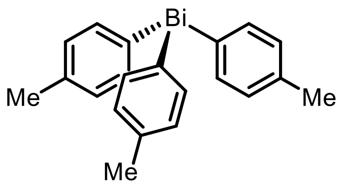


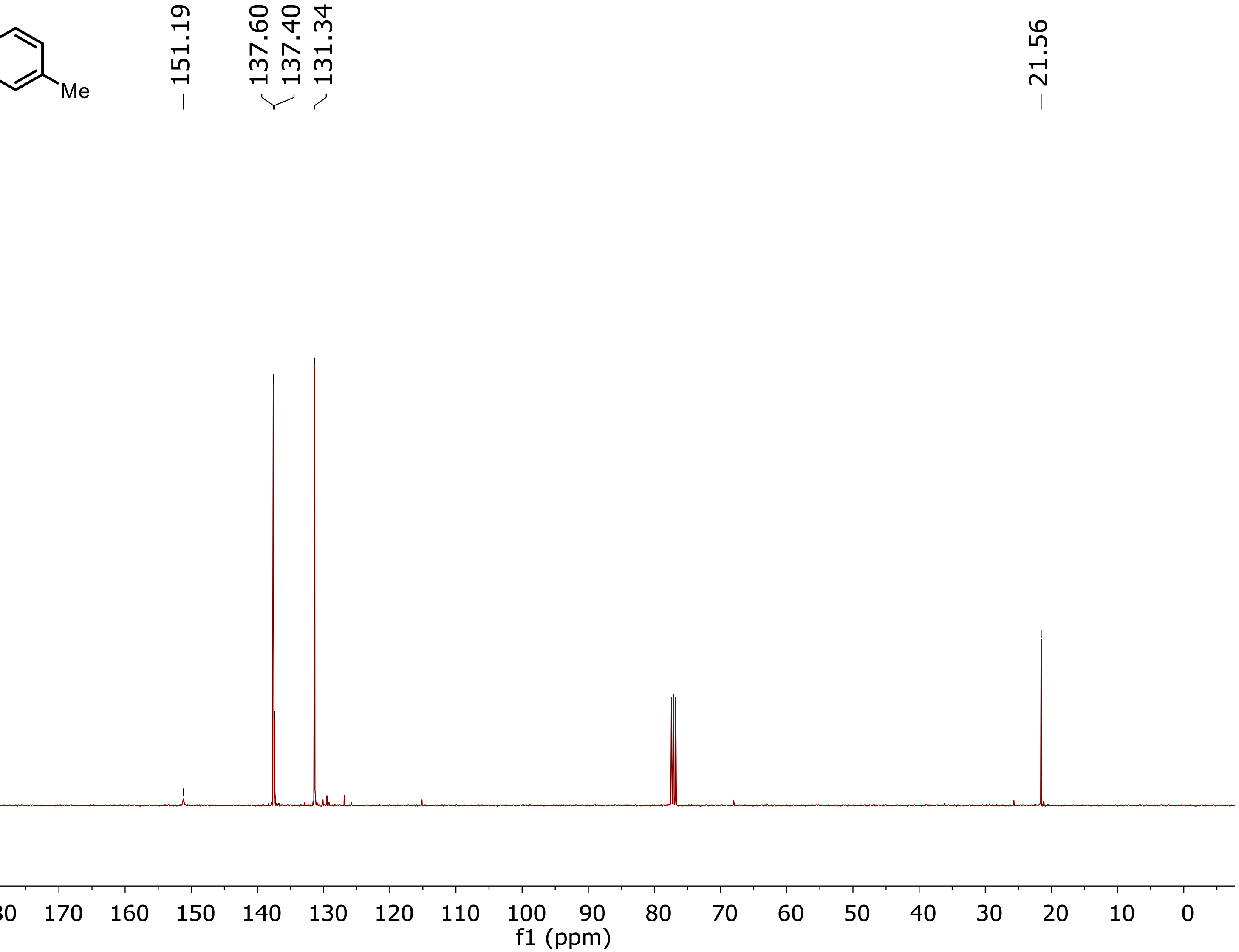

**Tri(4-methoxyphenyl)bismuthine - $^1$H NMR (500 MHz, $CDCl_3$):**

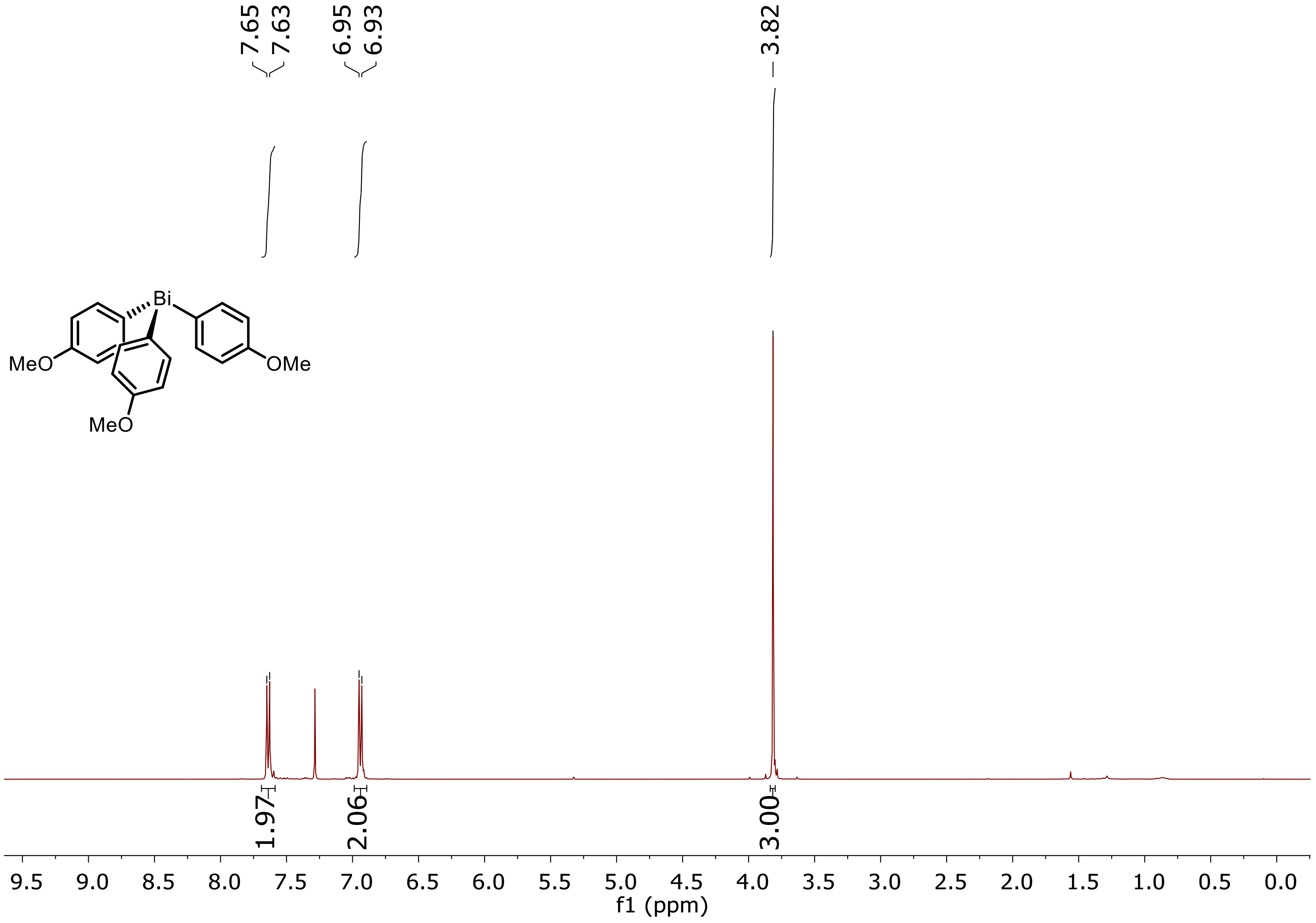

**Tri(4-methoxyphenyl)bismuthine - $^{13}C\{^1H\}$ NMR (126 MHz, $CDCl_3$):**

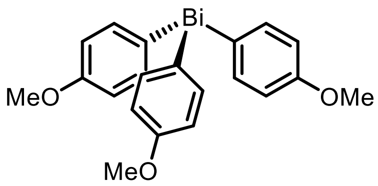

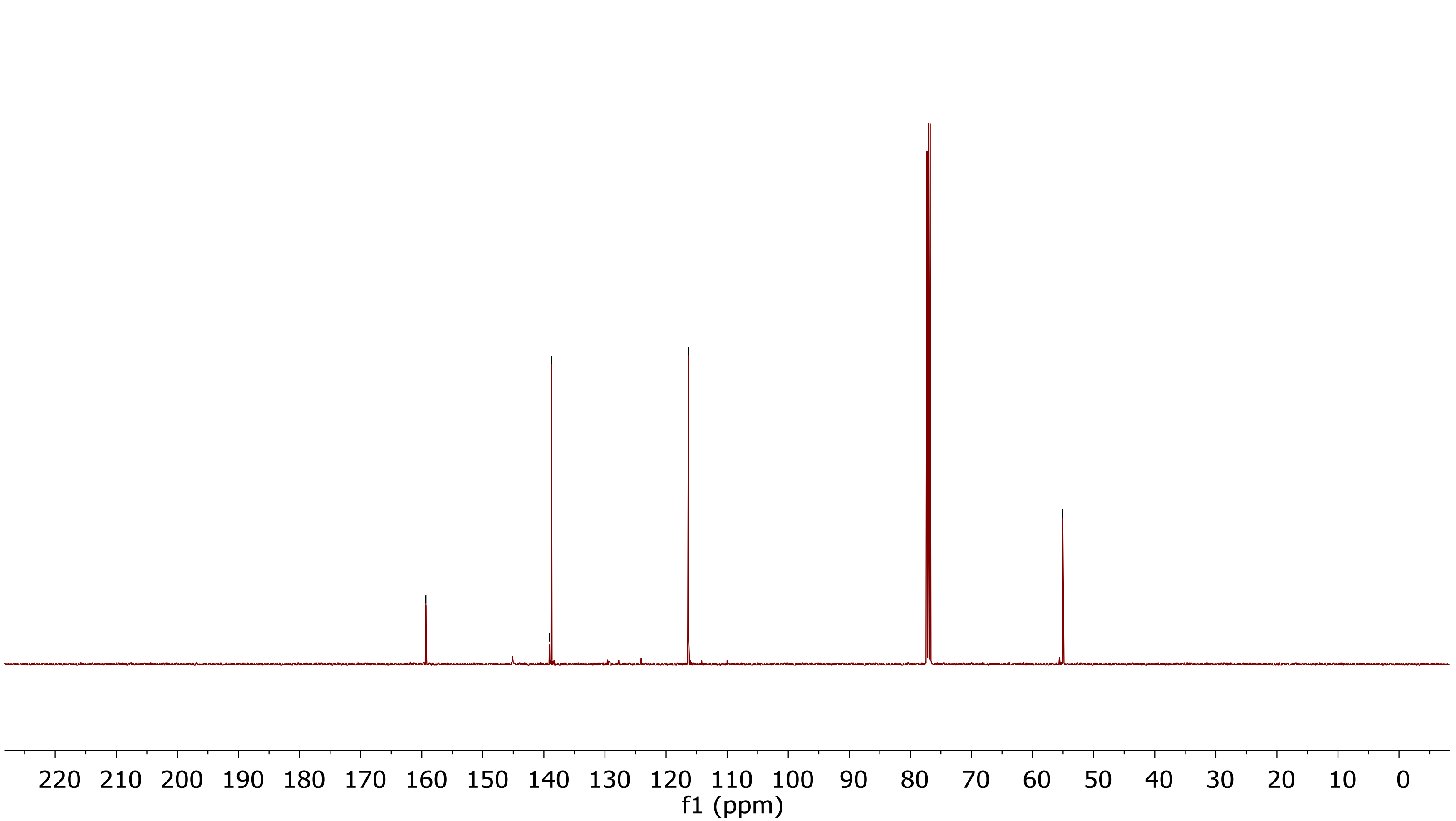

**Tri(4-chlorophenyl)bismuthine - $^{1}$H NMR (400 MHz, $CDCl_3$):**

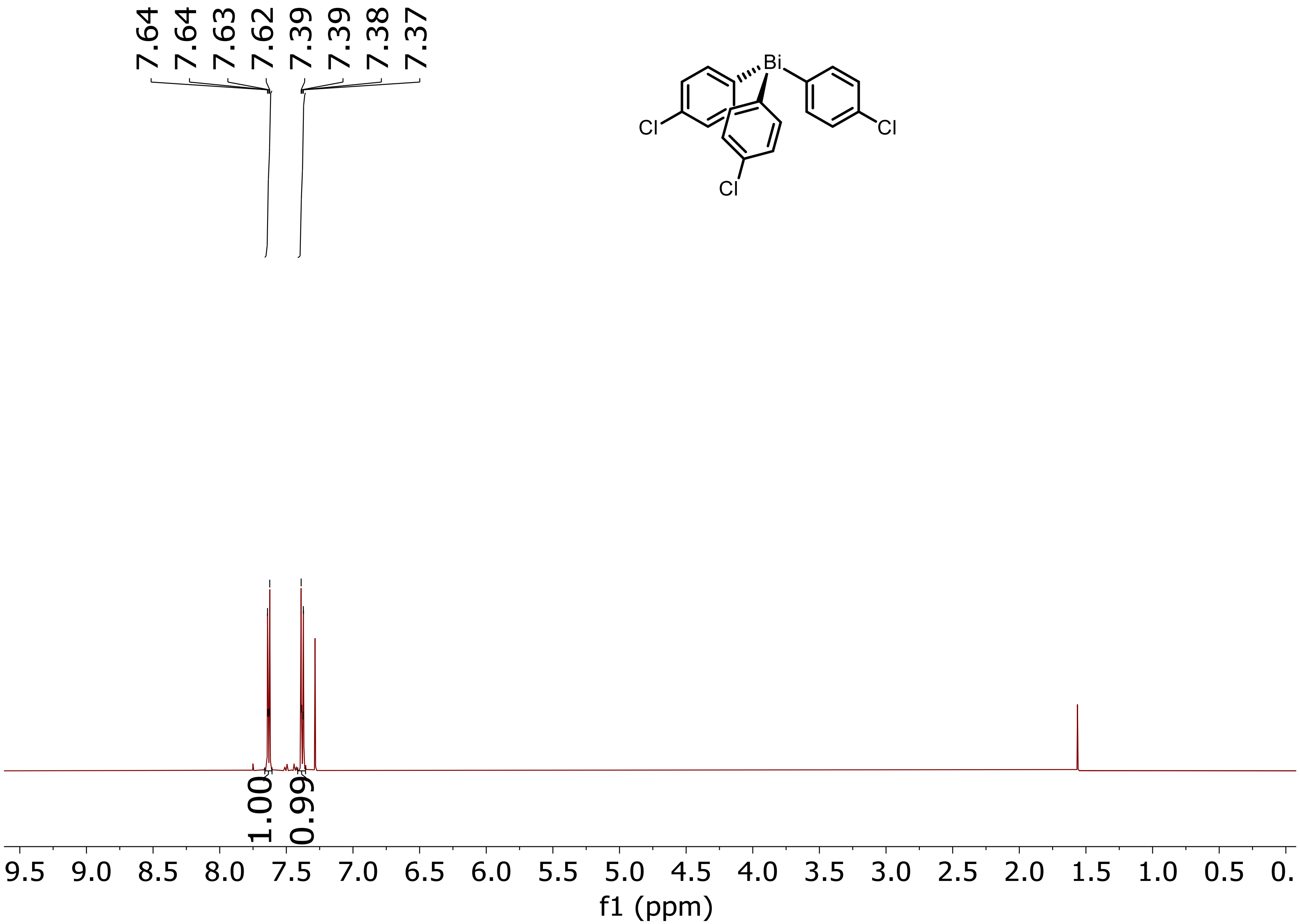

**Tri(4-chlorophenyl)bismuthine - $^{13}C\{^1H\}$ NMR (101 MHz, $CDCl_3$):**

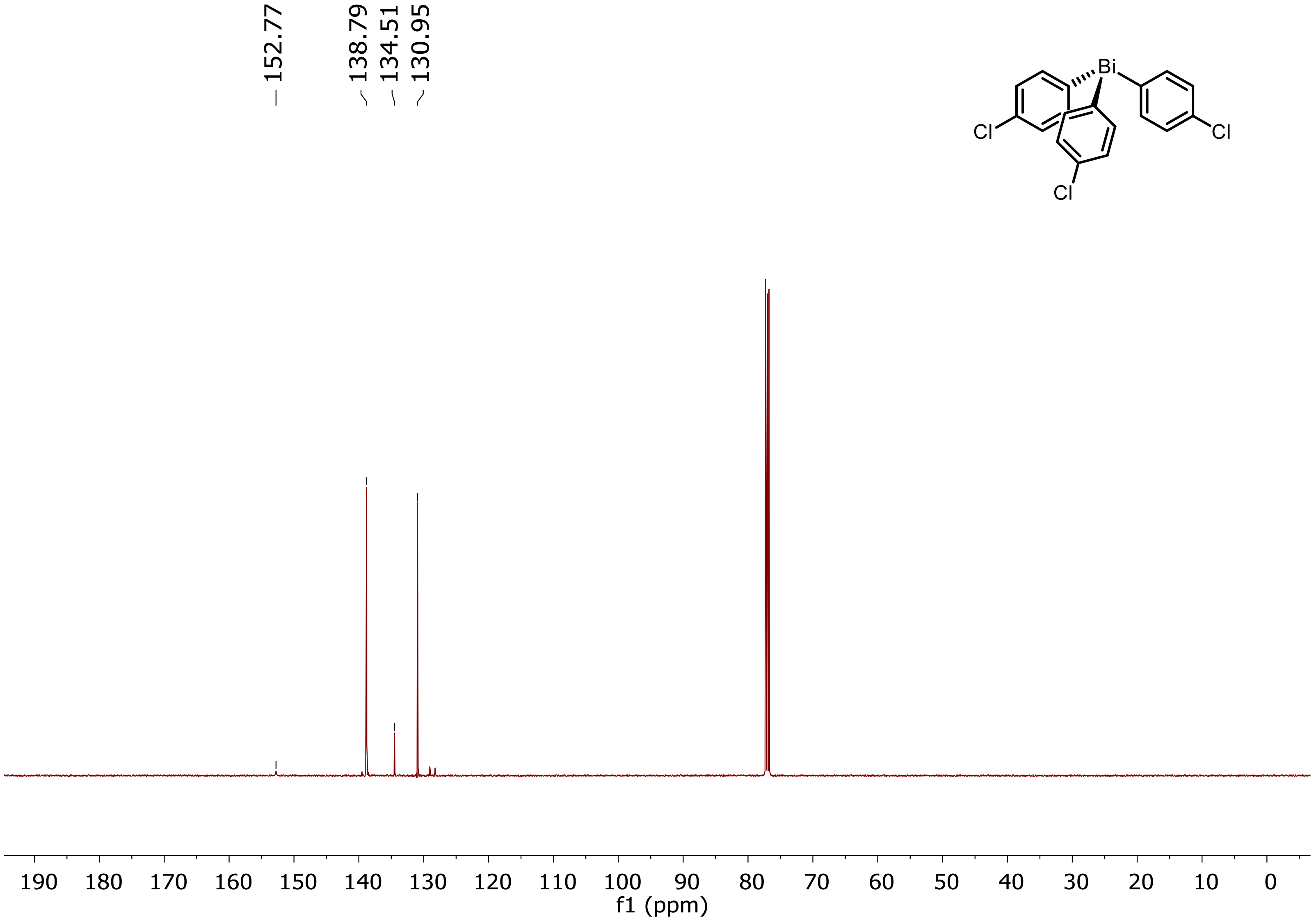

**Ethyl 2-(4-fluorophenyl)-2-oxoacetate (1a) – $^{1}$H NMR (400 MHz, $CDCl_3$):**

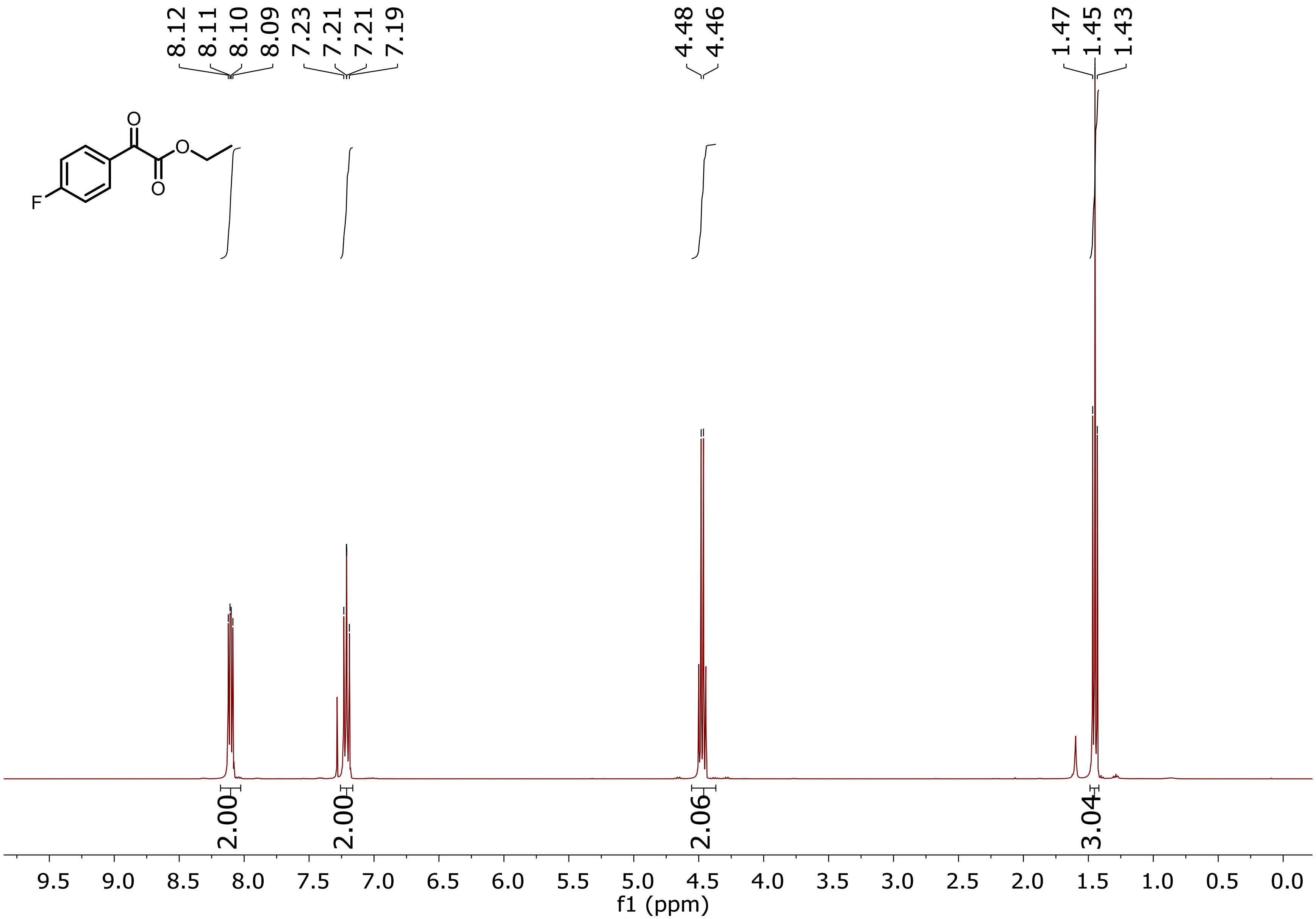

**Ethyl 2-(4-fluorophenyl)-2-oxoacetate (1a) – $^{13}C\{^1H\}$ NMR (101 MHz, $CDCl_3$):**

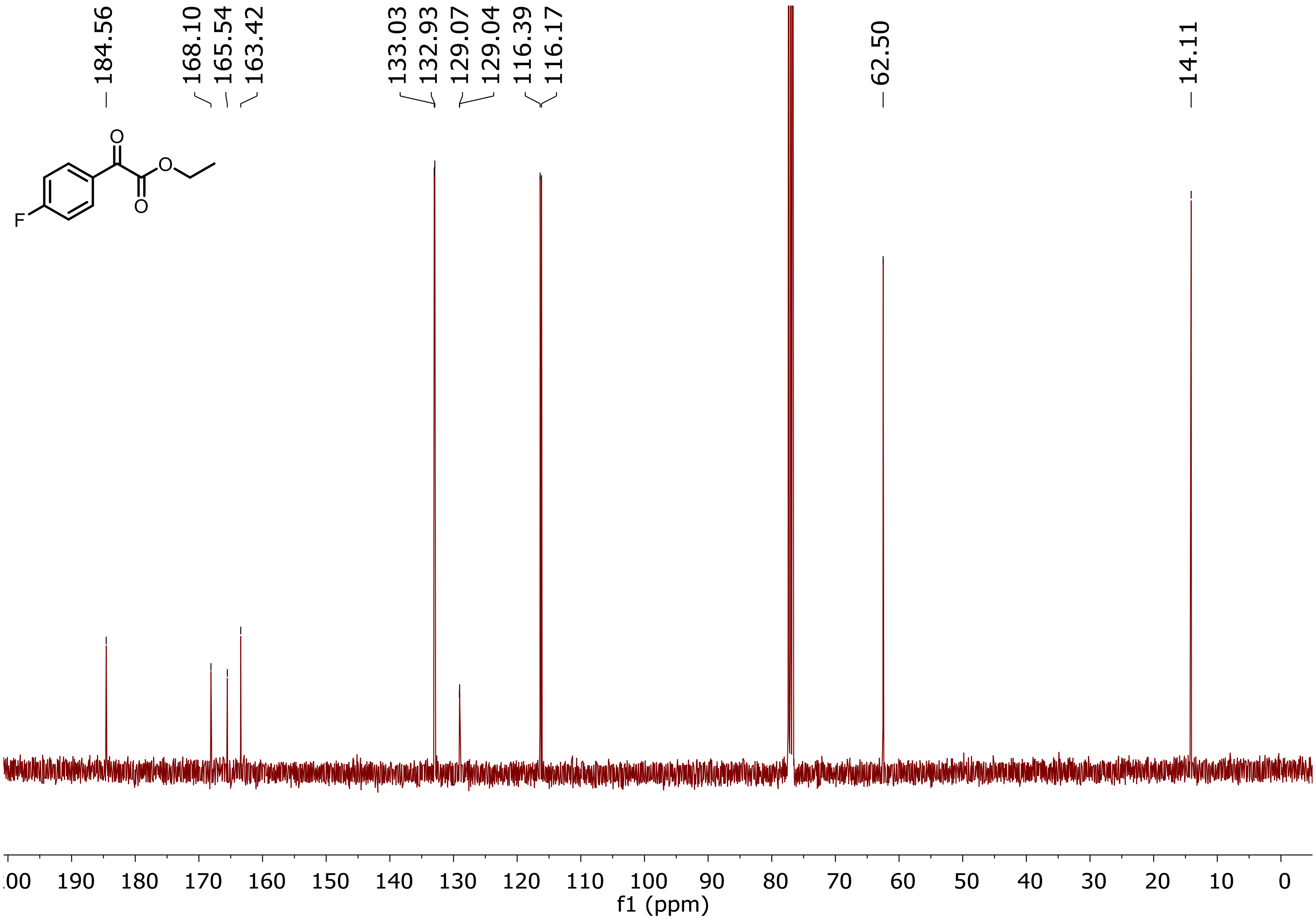

**Ethyl 2-(4-fluorophenyl)-2-oxoacetate (1a) – $^{19}$F NMR (376 MHz, $CDCl_3$):**

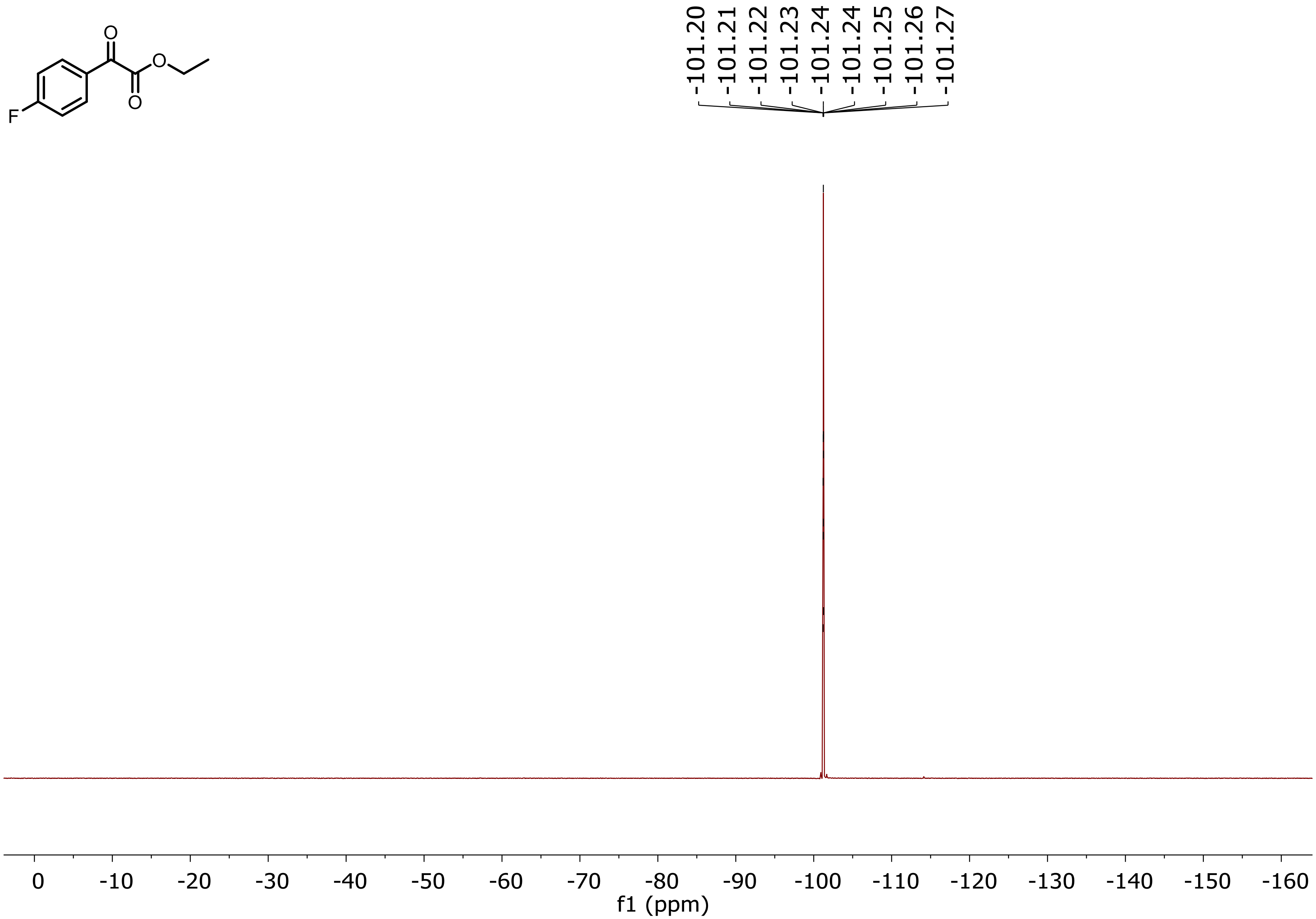

**Ethyl 2-oxo-2-(*p*-tolyl)acetate (1c) – $^{1}$H NMR (400 MHz, $CDCl_3$):**

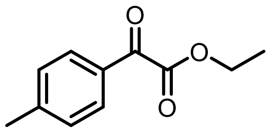

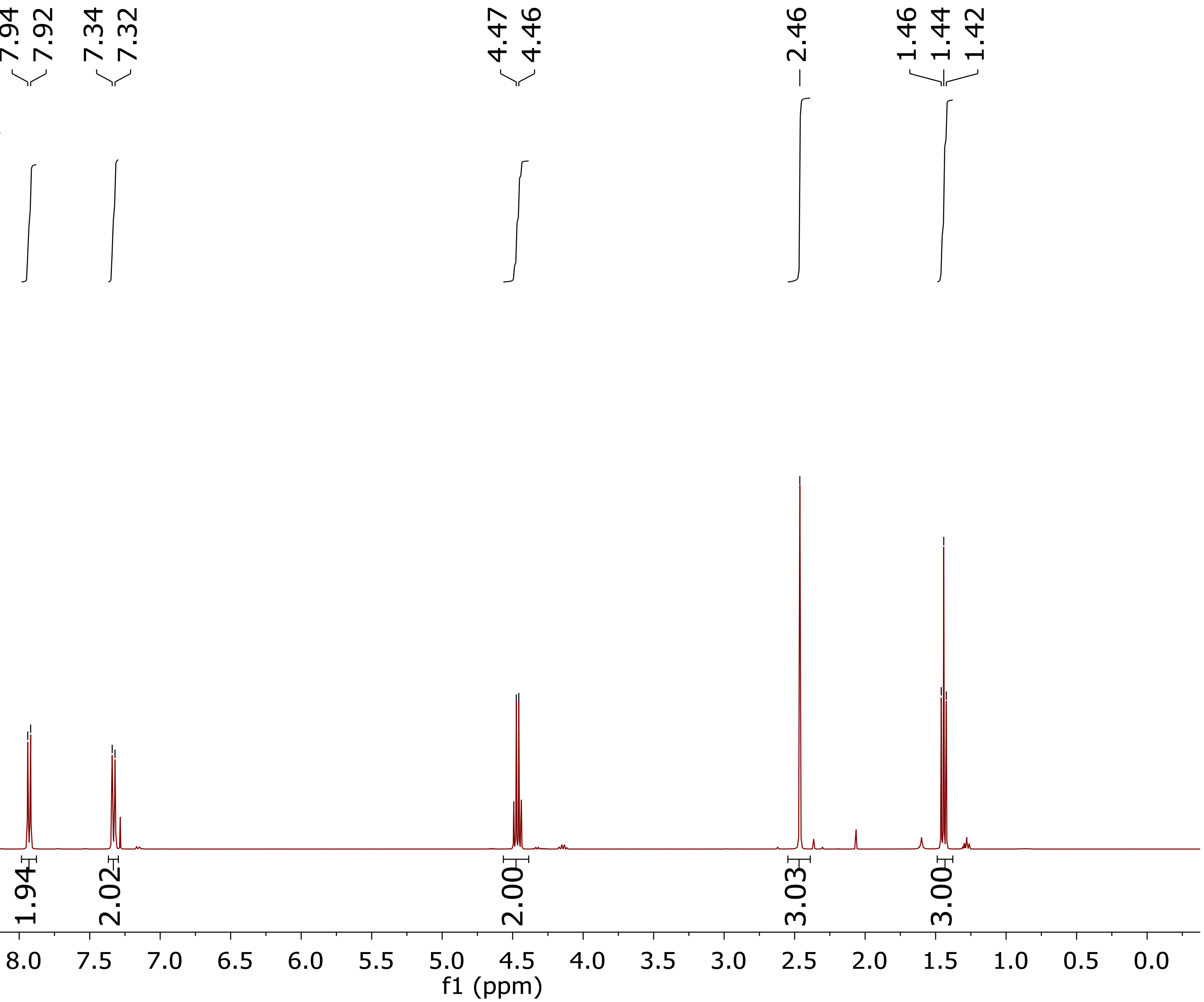

**Ethyl 2-oxo-2-(*p*-tolyl)acetate (1c) – $^{13}C\{^{1}H\}$ NMR (101 MHz, $CDCl_3$):**

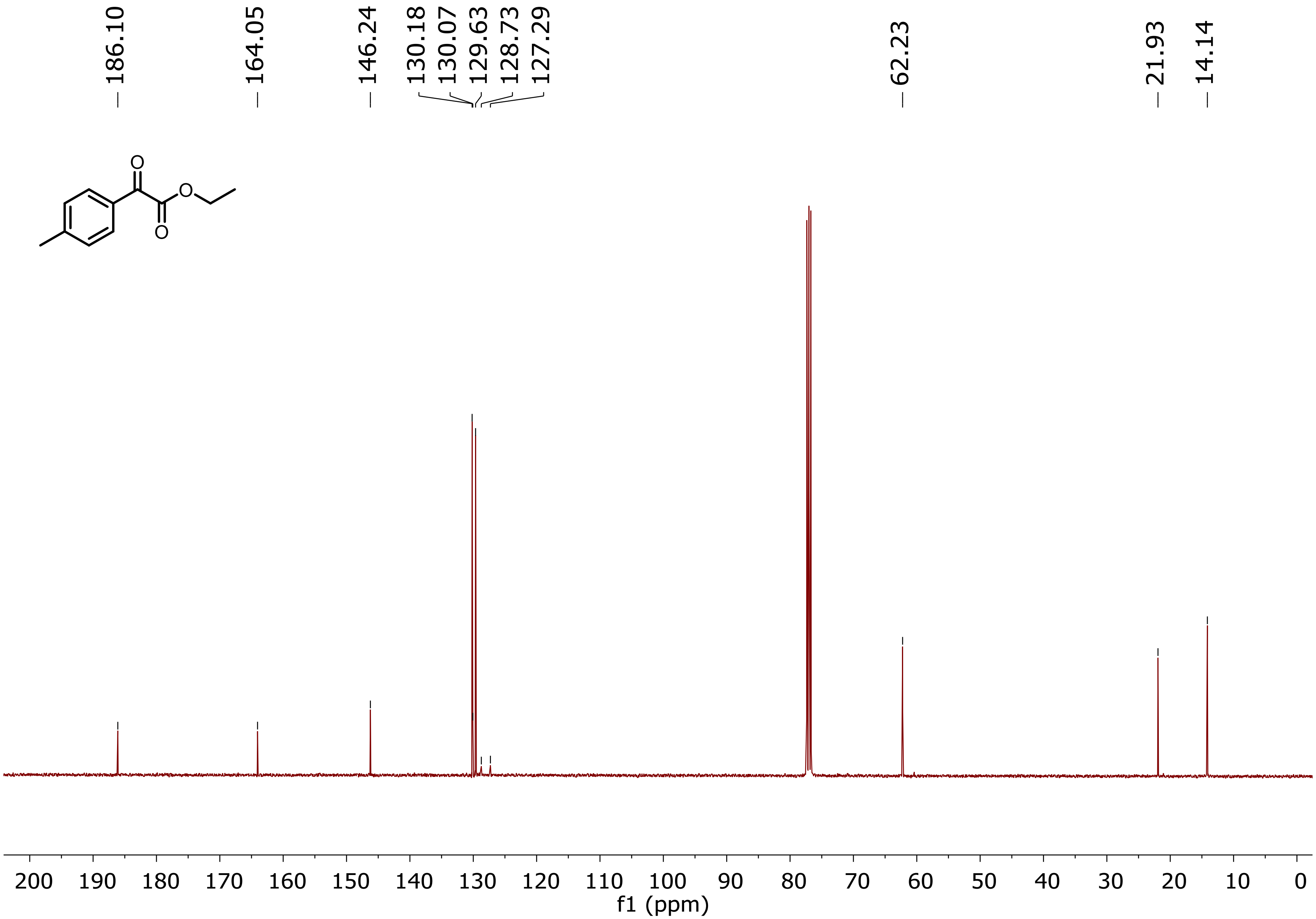

**Ethyl 2-(3-bromo-4-methoxyphenyl)-2-oxoacetate (1g) – $^{1}$H NMR (400 MHz, $CDCl_3$):**

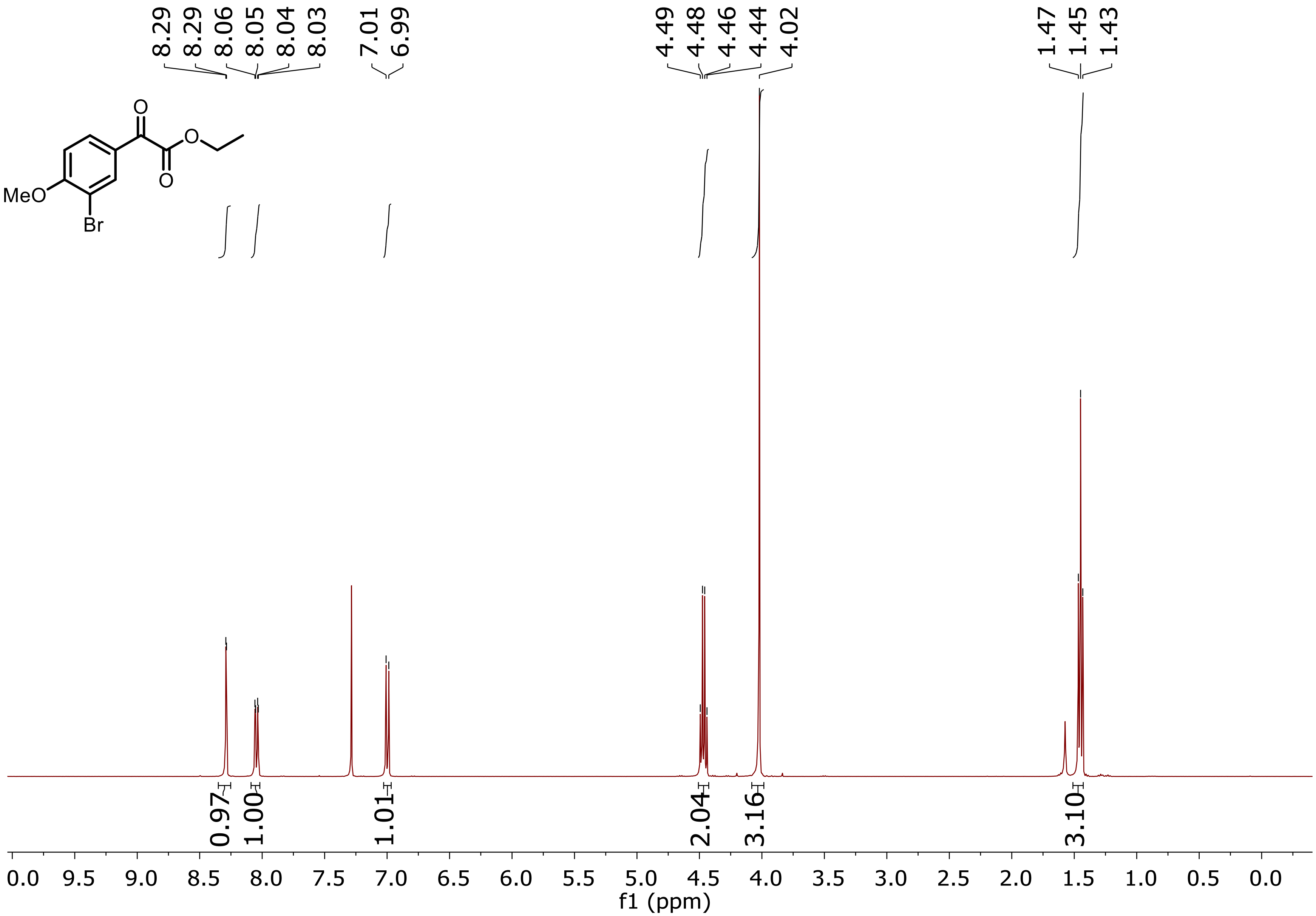

**Ethyl 2-(3-bromo-4-methoxyphenyl)-2-oxoacetate (1g) – $^{13}C\{^{1}H\}$ NMR (101 MHz, $CDCl_3$):**

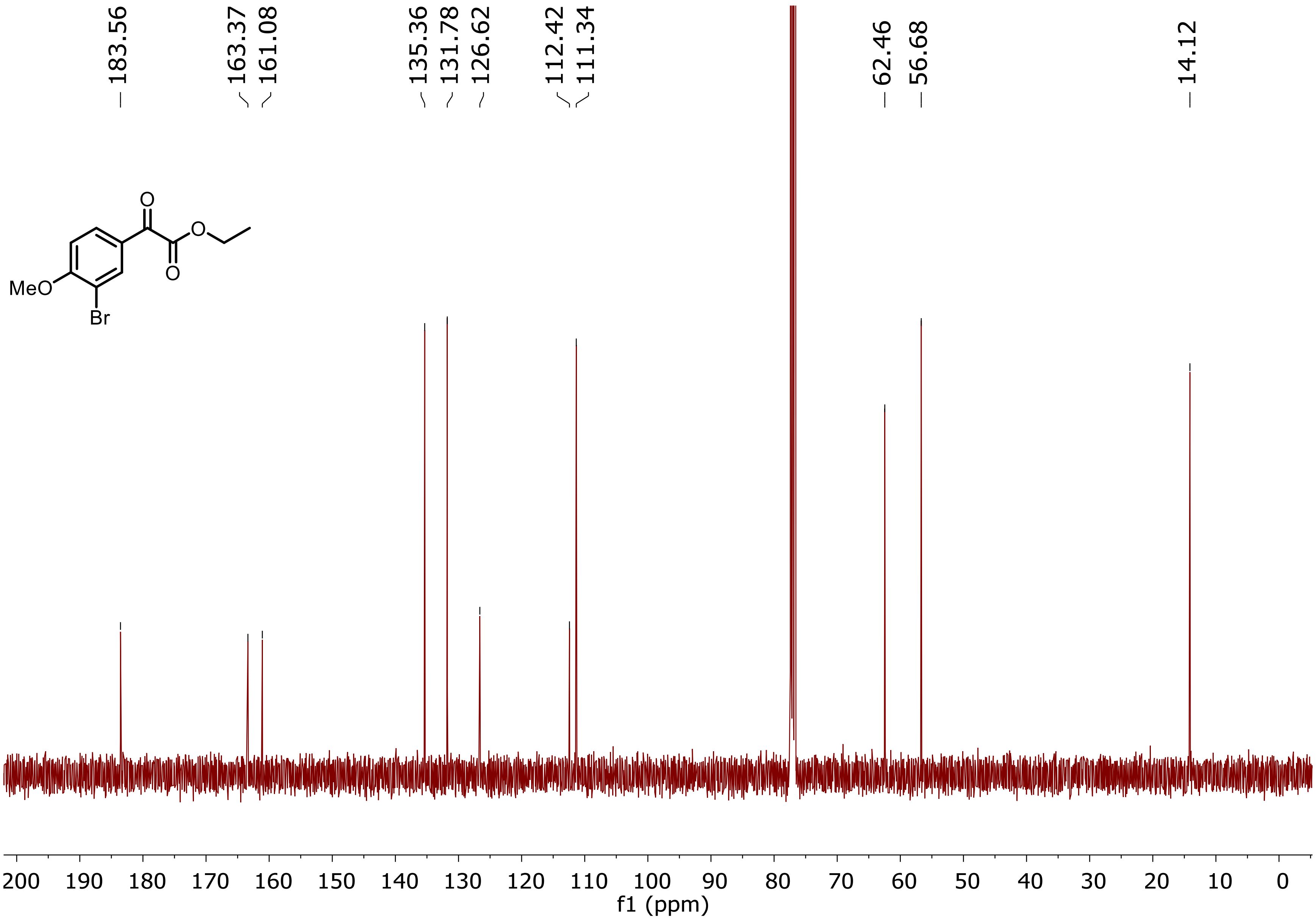

**Ethyl 2-(naphthalen-2-yl)-2-oxoacetate (1h) – $^{1}$H NMR (400 MHz, $CDCl_3$):**

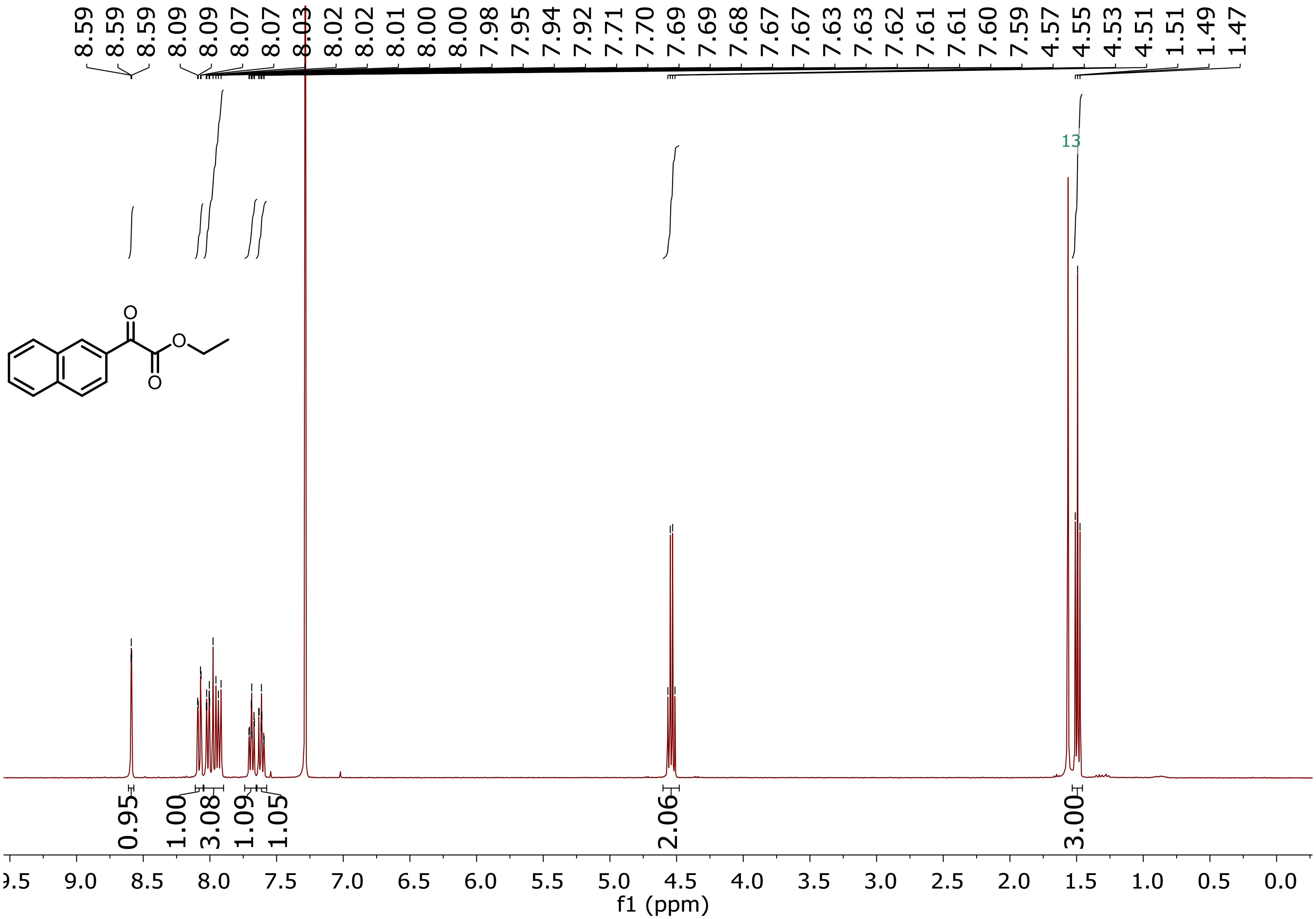

**Ethyl 2-(naphthalen-2-yl)-2-oxoacetate (1h) – $^{13}C\{^{1}H\}$ NMR (101 MHz, $CDCl_3$):**

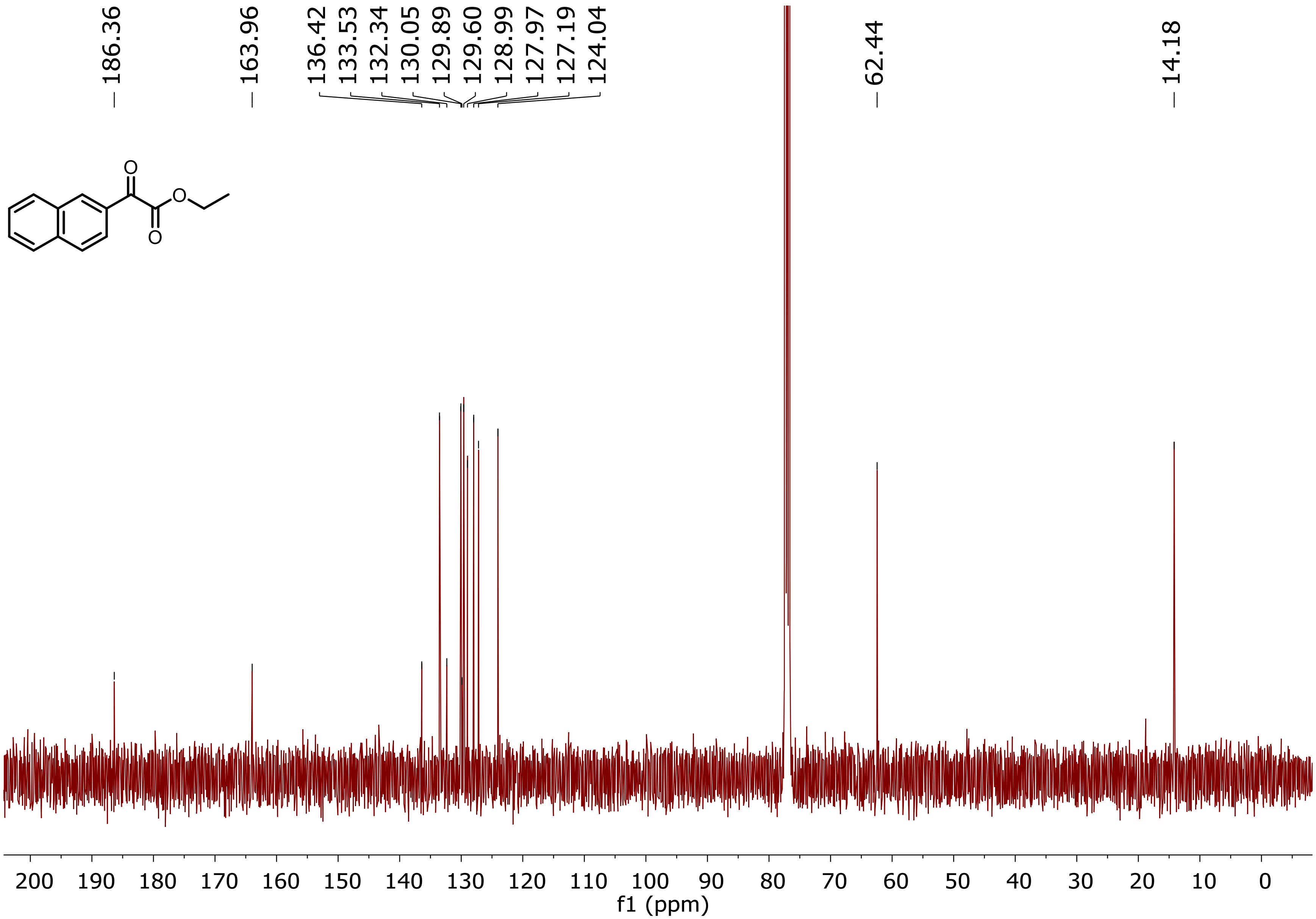

**Tetra(4-fluorophenyl)bismuthonium tetrafluoroborate (3a) - $^{1}$H NMR (400 MHz, $CDCl_3$):**

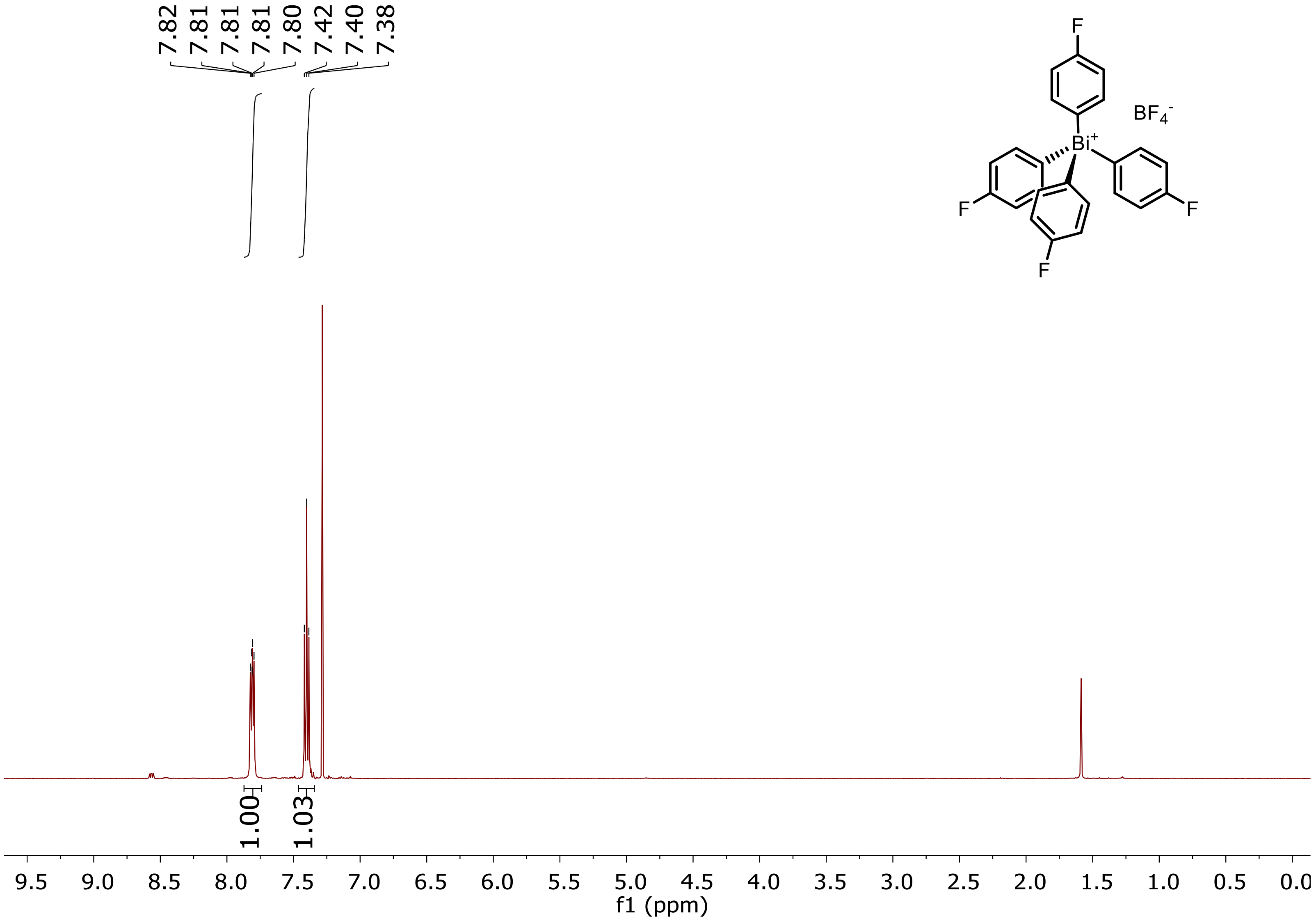

**Tetra(4-fluorophenyl)bismuthonium tetrafluoroborate (3a) - $^{13}C\{^1H\}$ NMR (101 MHz, $CDCl_3$):**

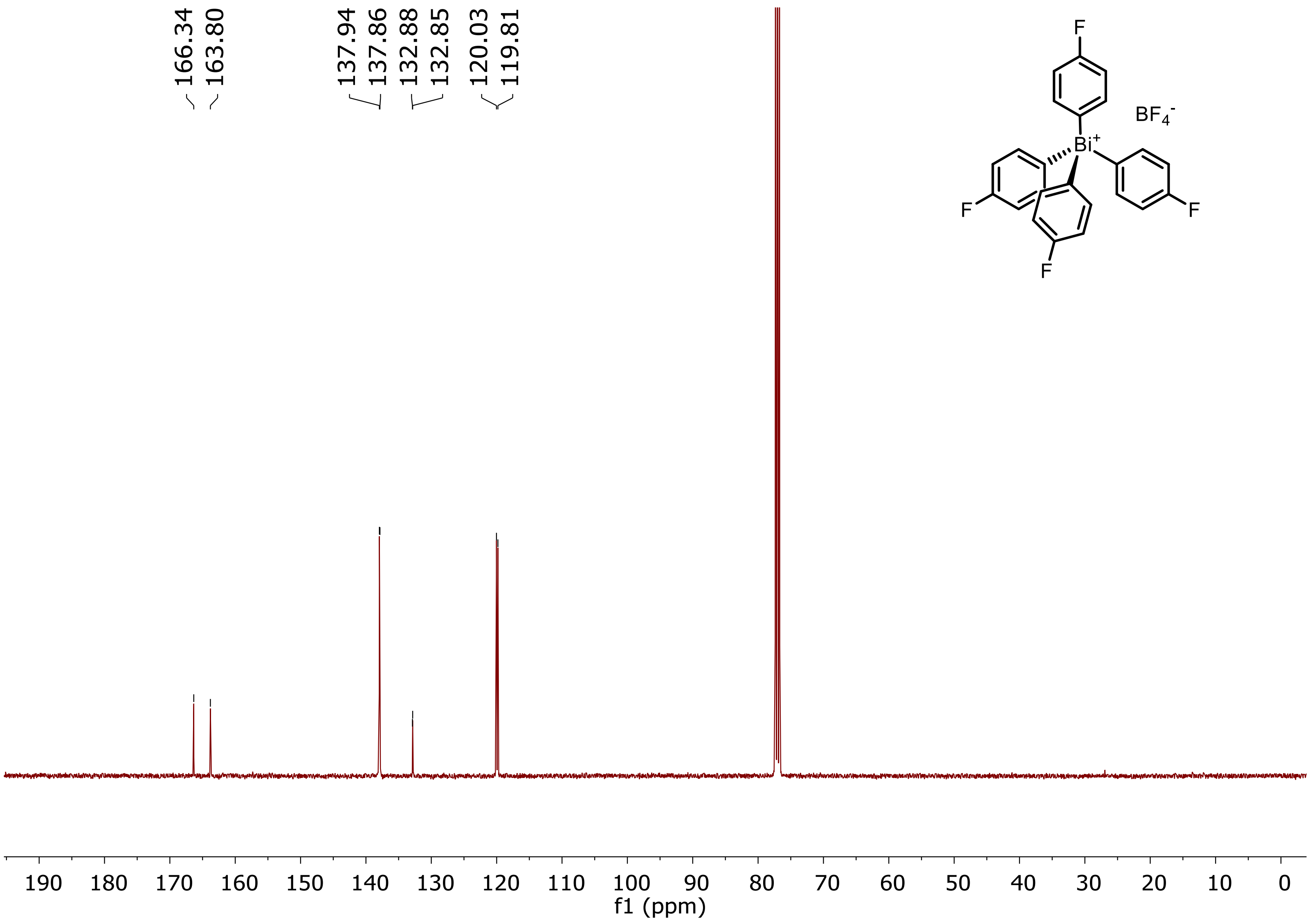

**Tetra(4-fluorophenyl)bismuthonium tetrafluoroborate (3a) -$^{19}$F NMR (376 MHz, $CDCl_3$):**

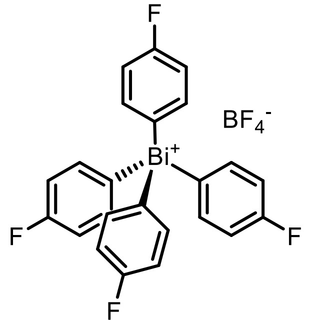

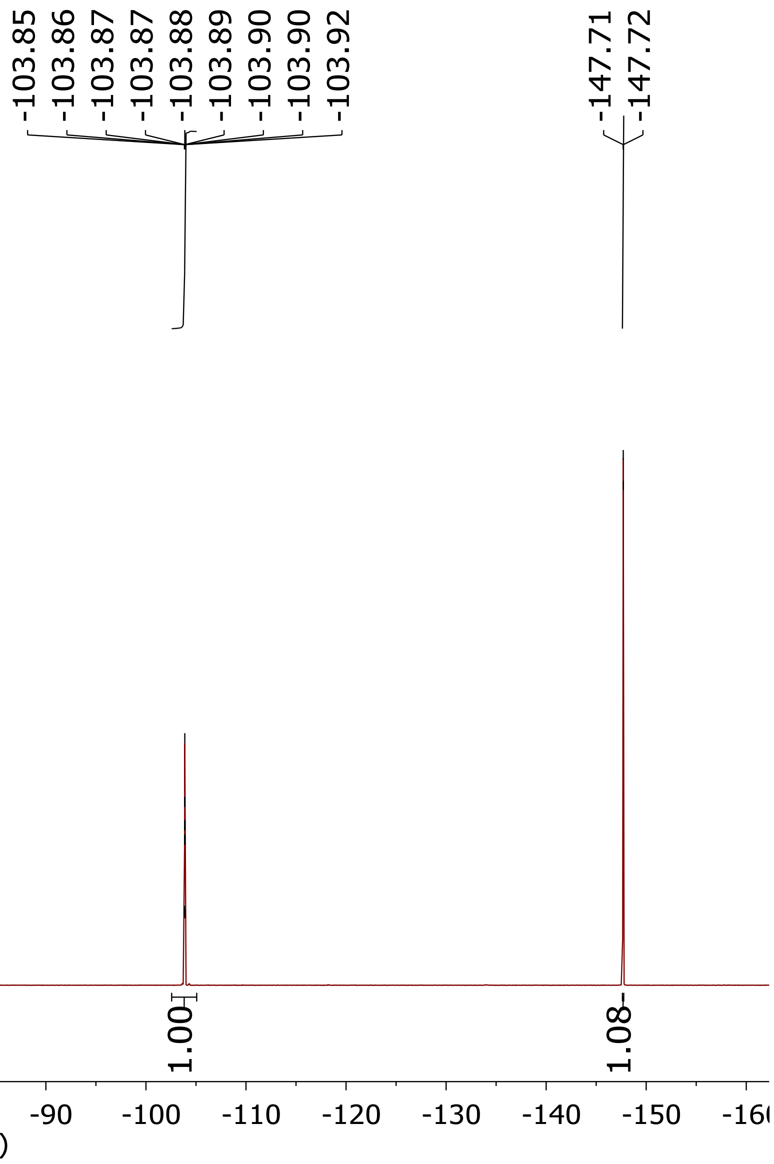

**Tetra(4-methylphenyl)bismuthonium tetrafluoroborate (3b) - $^1$H NMR (400 MHz, $CDCl_3$):**

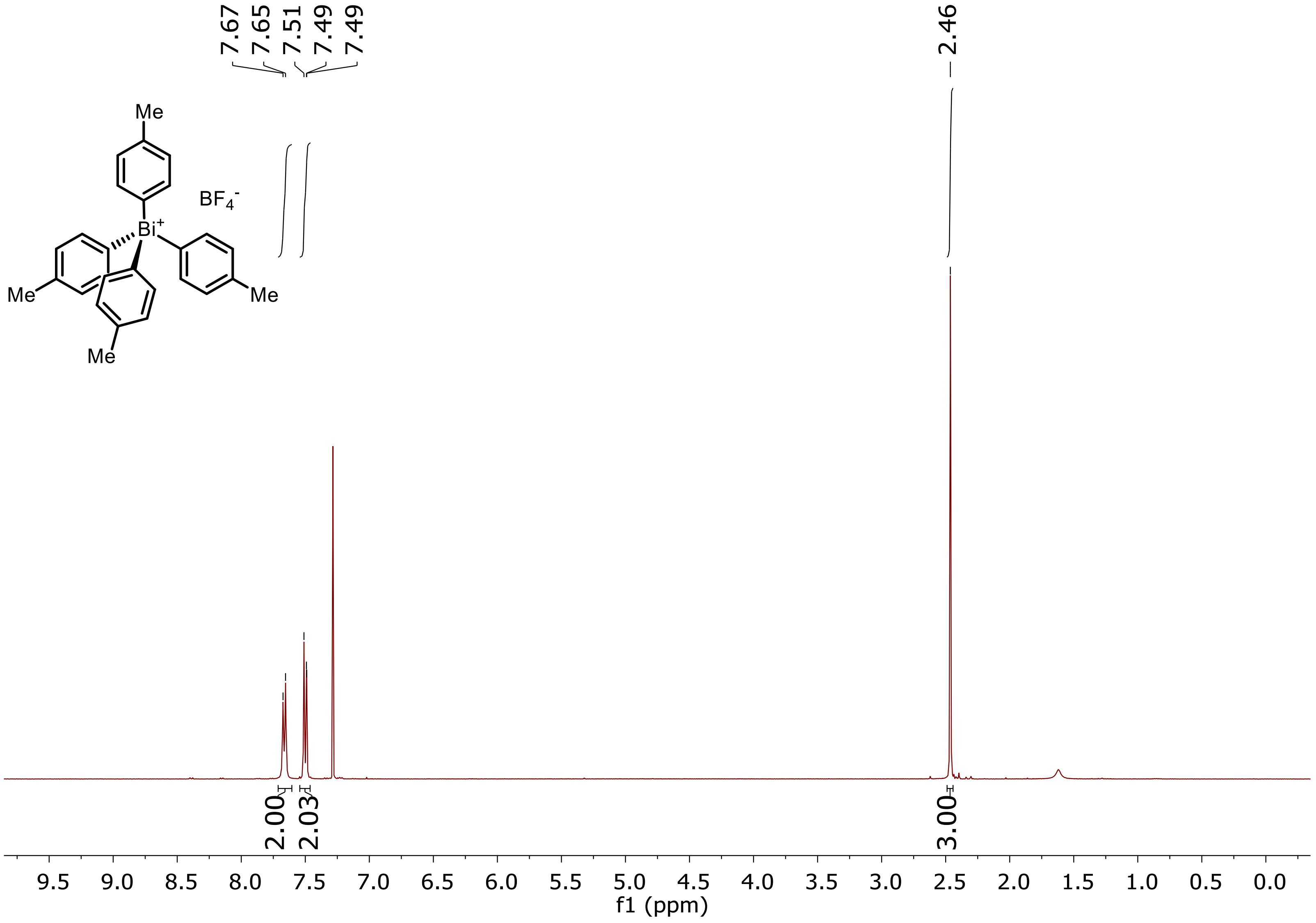

**Tetra(4-methylphenyl)bismuthonium tetrafluoroborate (3b) - $^{13}C\{^{1}H\}$ NMR (101 MHz, $CDCl_3$):**

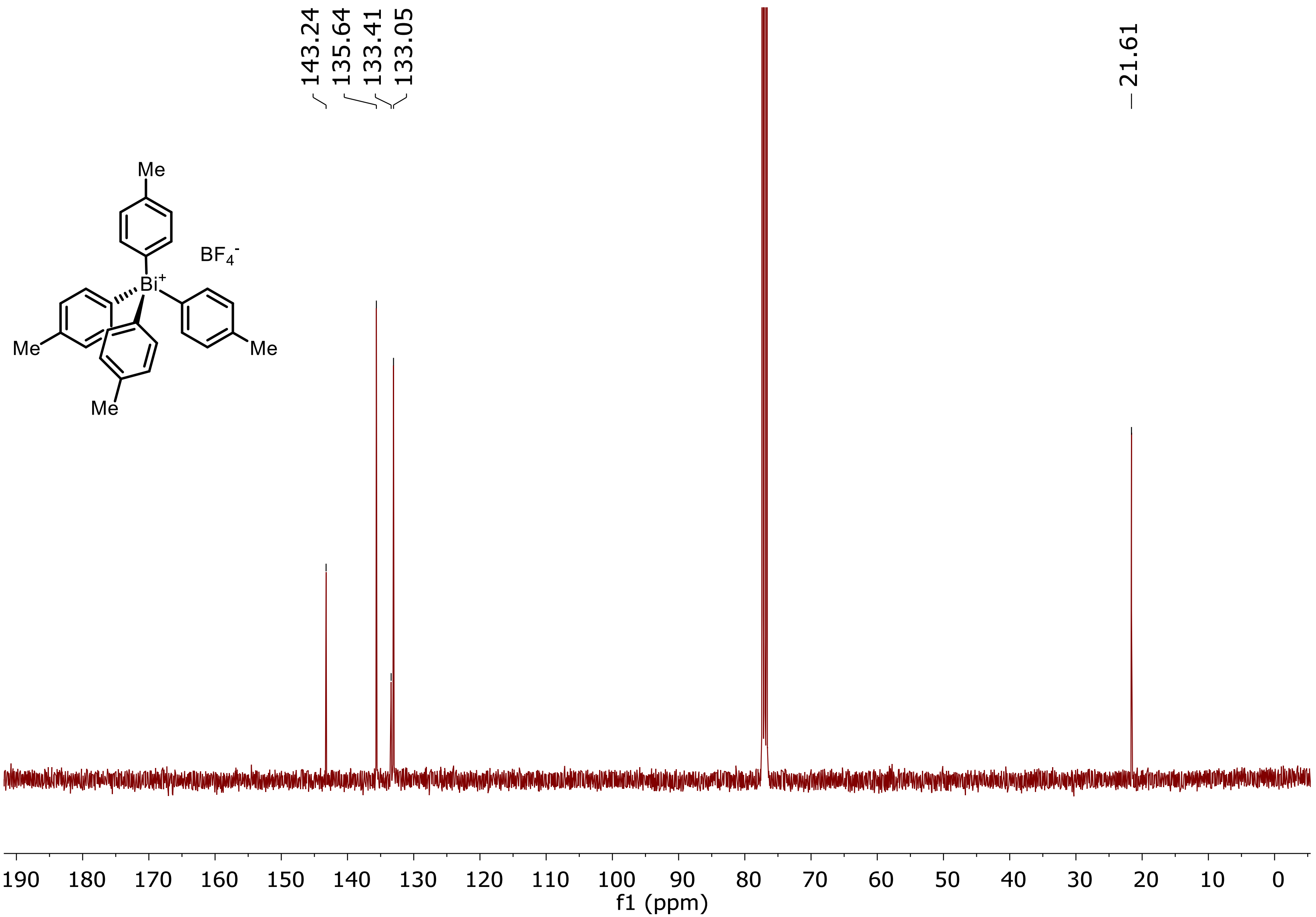

**Tetra(4-methoxyphenyl)bismuthonium tetrafluoroborate (3c) - $^1H$ NMR (500 MHz, $CDCl_3$):**

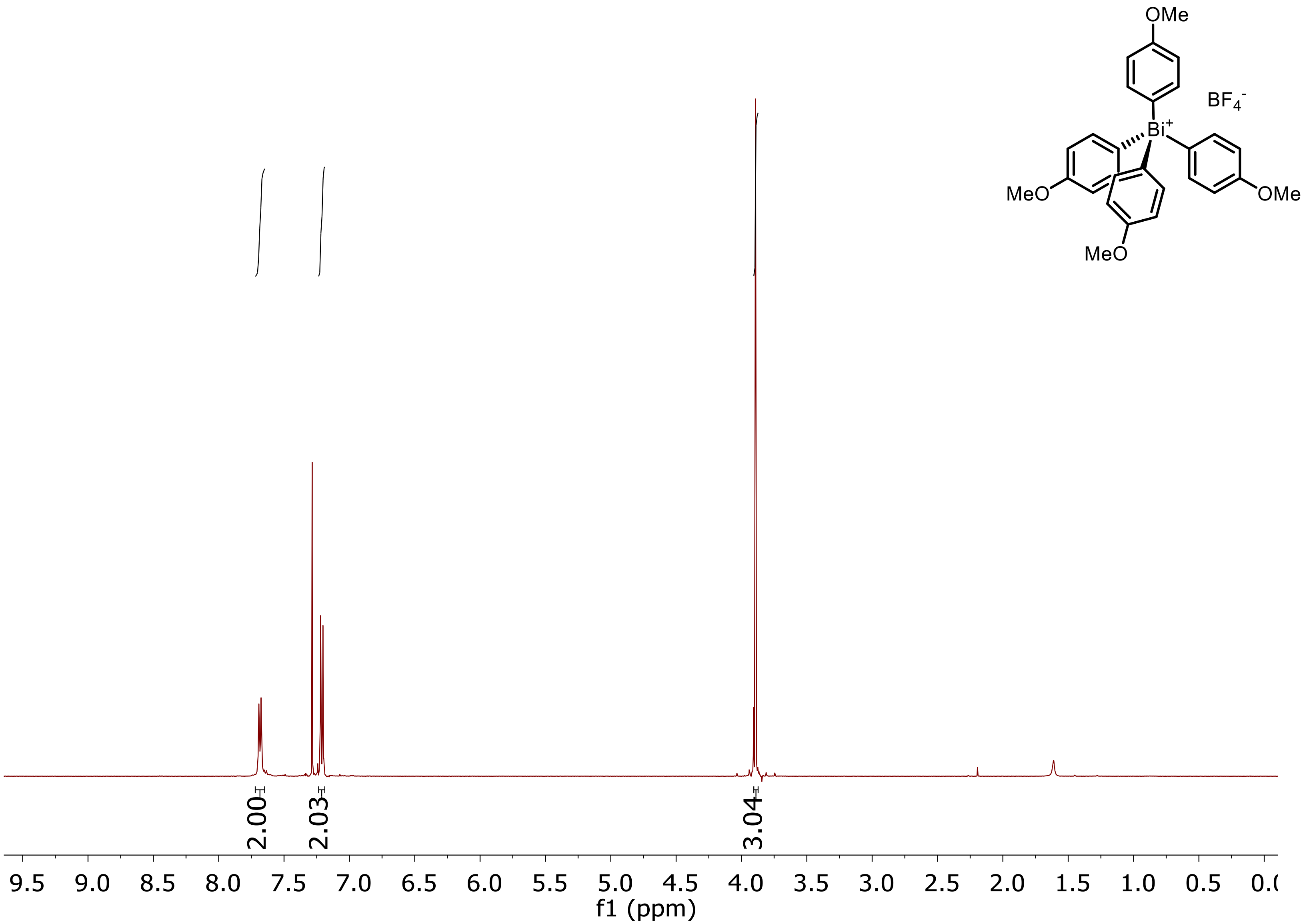

**Tetra(4-methoxyphenyl)bismuthonium tetrafluoroborate (3c) - $^{13}C\{^{1}H\}$ NMR (126 MHz, $CDCl_3$):**

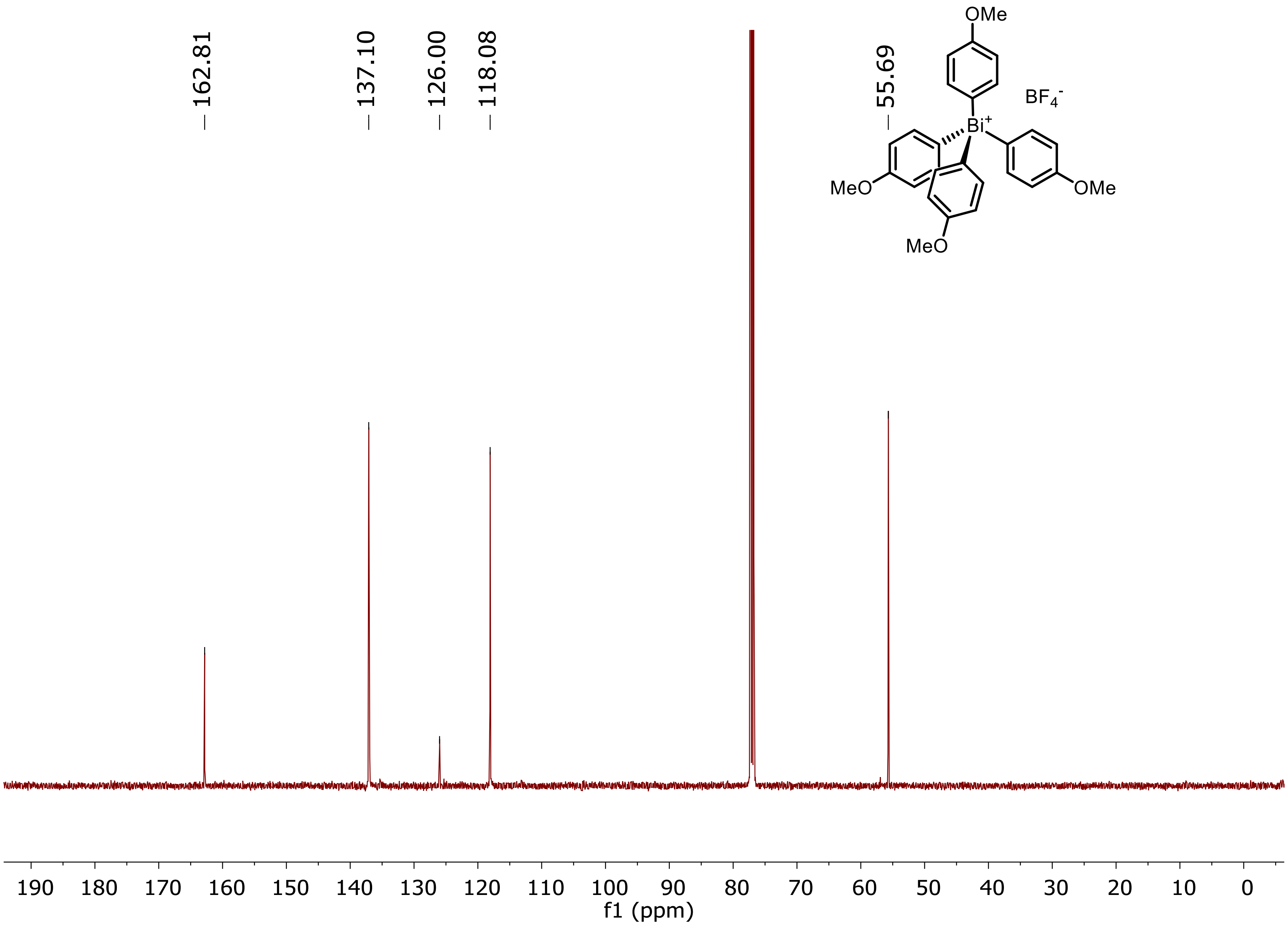

**Tetra(4-chlorophenyl)bismuthonium tetrafluoroborate (3d) - $^{1}$H NMR (400 MHz, $CDCl_3$):**

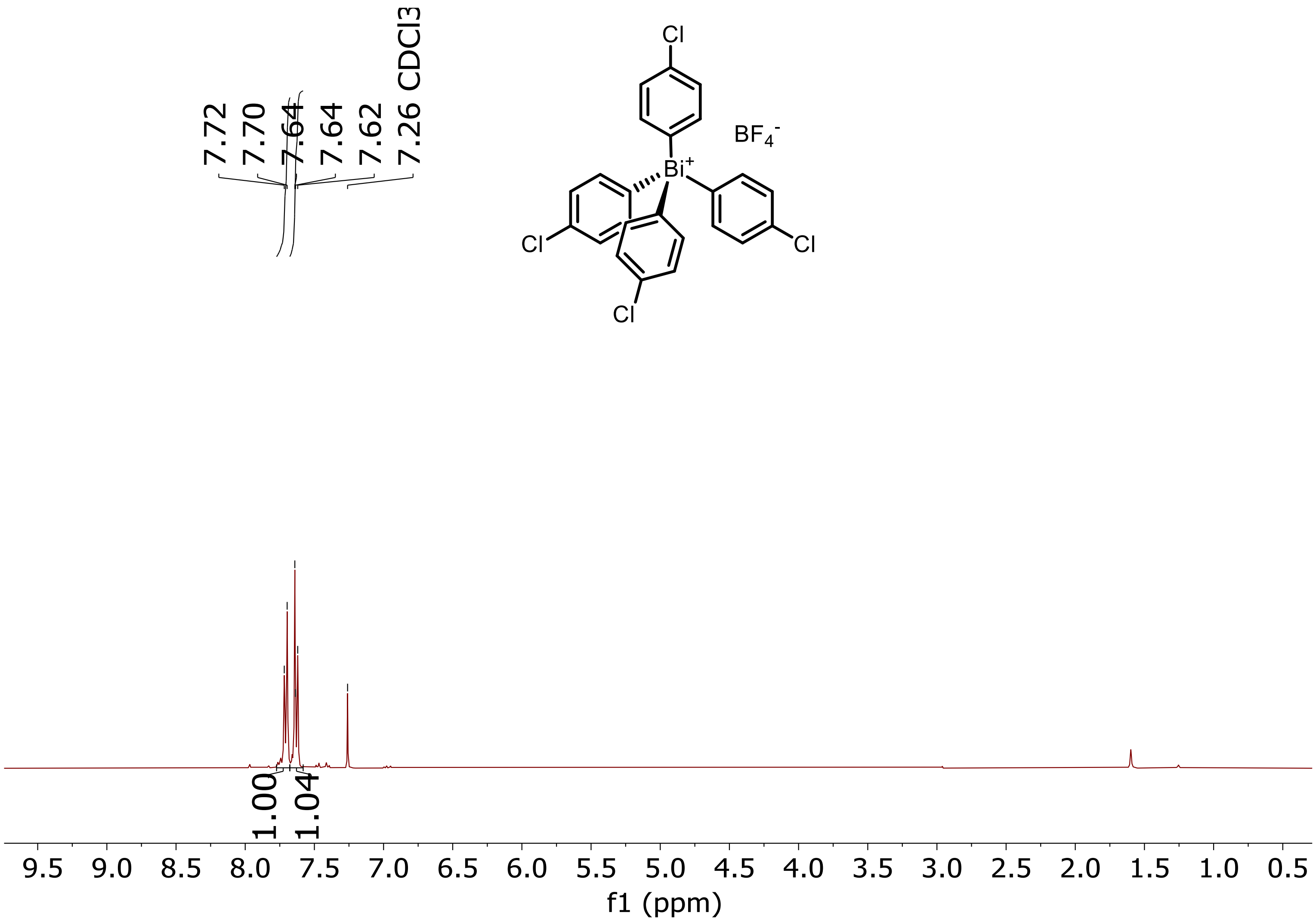

**Tetra(4-chlorophenyl)bismuthonium tetrafluoroborate (3d) - $^{13}C\{^1H\}$ NMR (101 MHz, $CDCl_3$):**

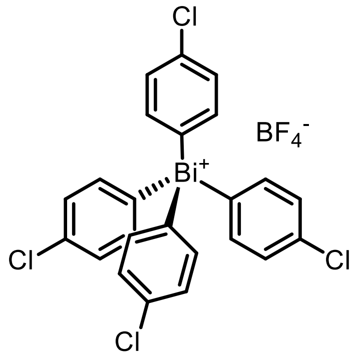

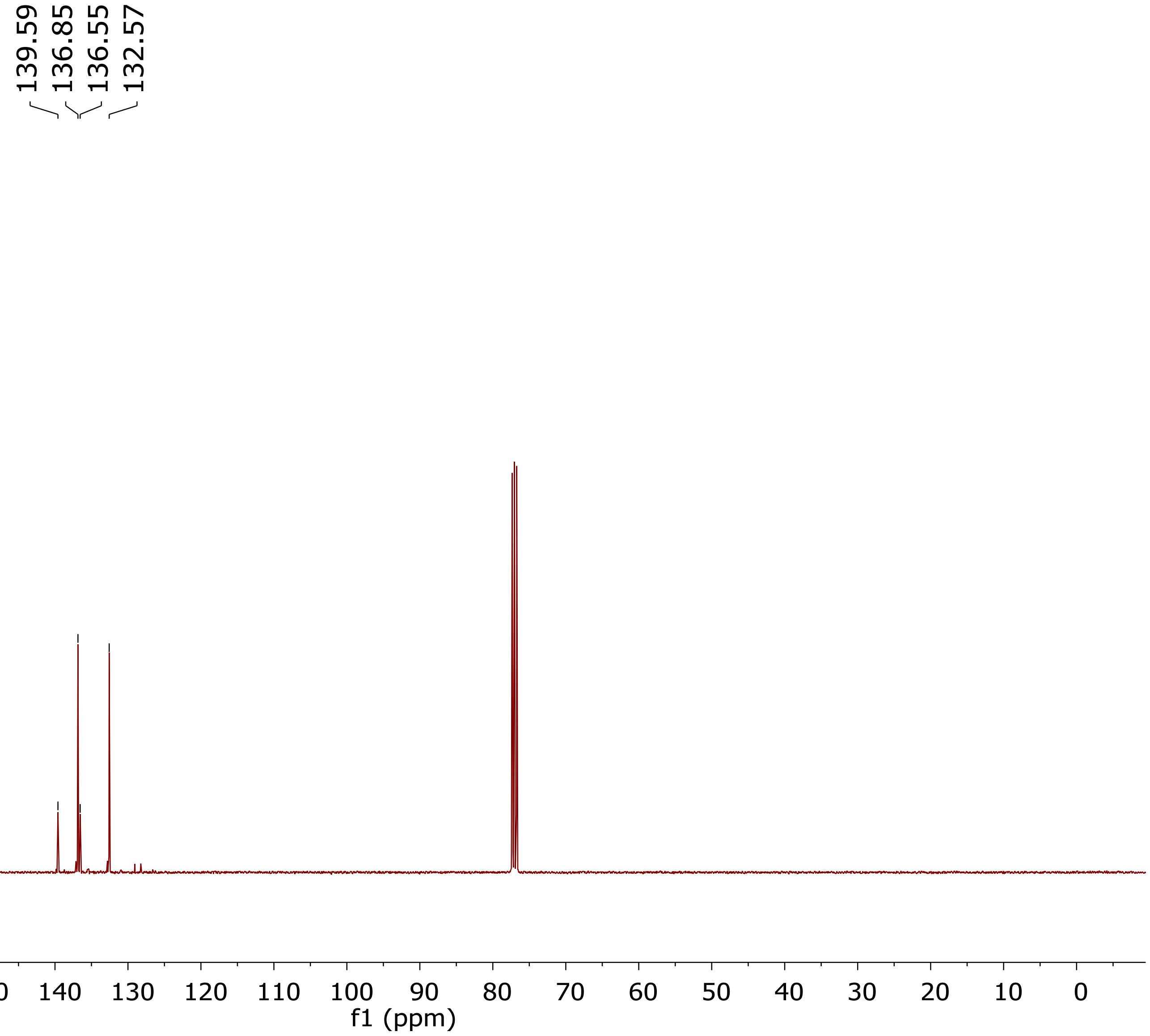

**Ethyl 2,2-bis(4-fluorophenyl)-2-((4-fluorophenyl)amino)acetate (8) - $^{1}$H NMR (400 MHz, $CDCl_3$):**

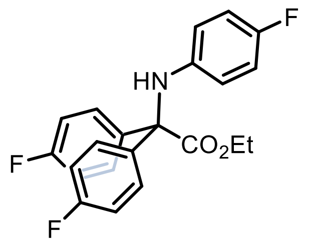


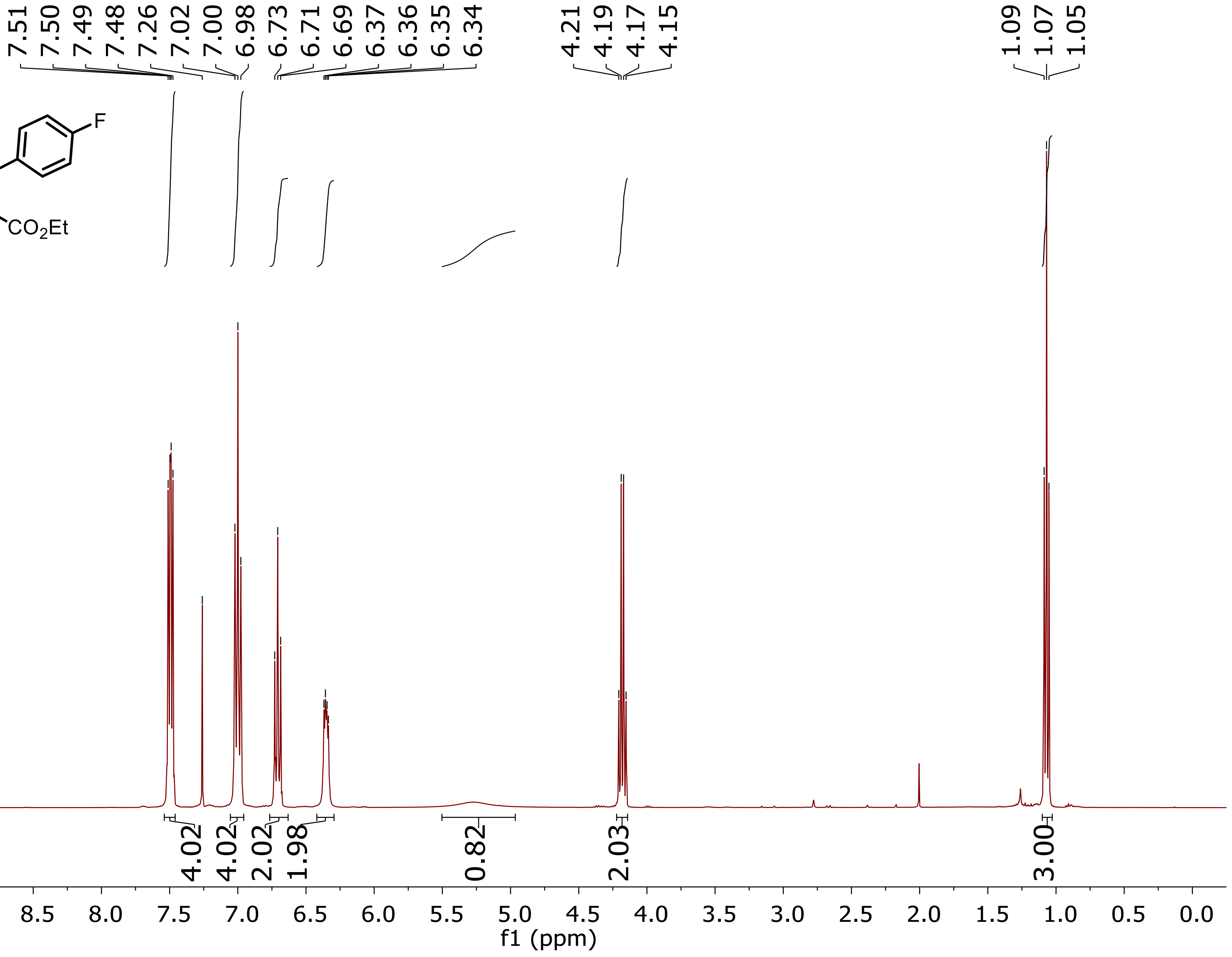

**Ethyl 2,2-bis(4-fluorophenyl)-2-((4-fluorophenyl)amino)acetate (8) - $^{13}C\{^1H\}$ NMR (101 MHz, $CDCl_3$):**

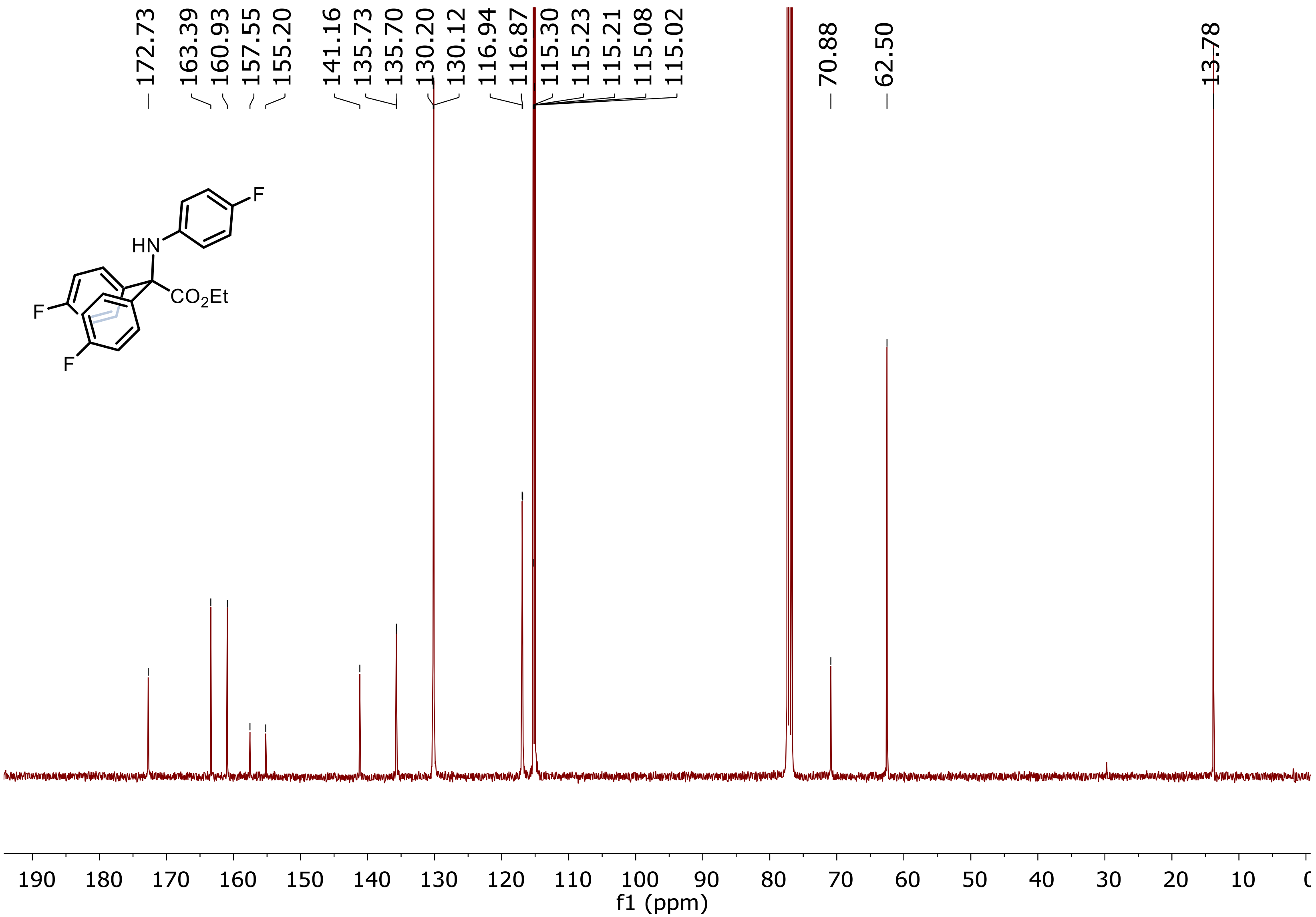

**Ethyl 2,2-bis(4-fluorophenyl)-2-((4-fluorophenyl)amino)acetate (8) - $^{19}F$ NMR (376 MHz, $CDCl_3$):**

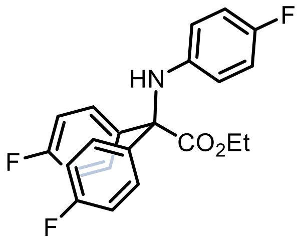


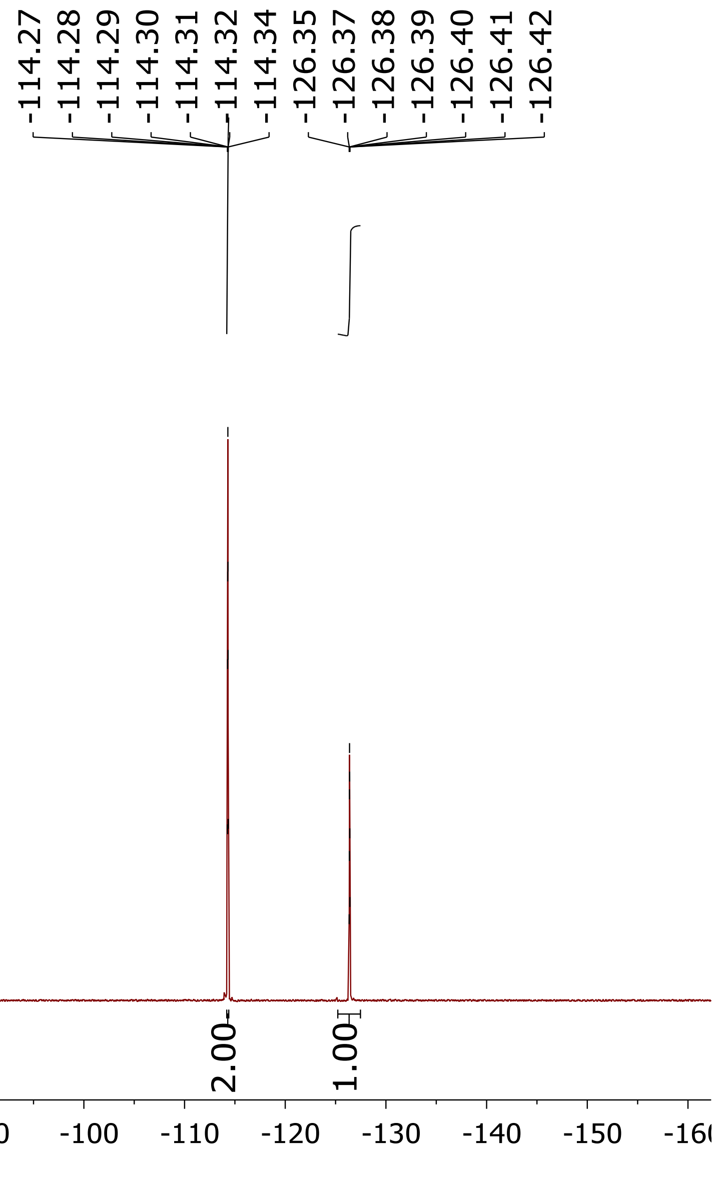

**Ethyl 2,2-bis(4-fluorophenyl)-2-((4-methoxyphenyl)amino)acetate (9) - $^{1}$H NMR (400 MHz, $CDCl_3$):**

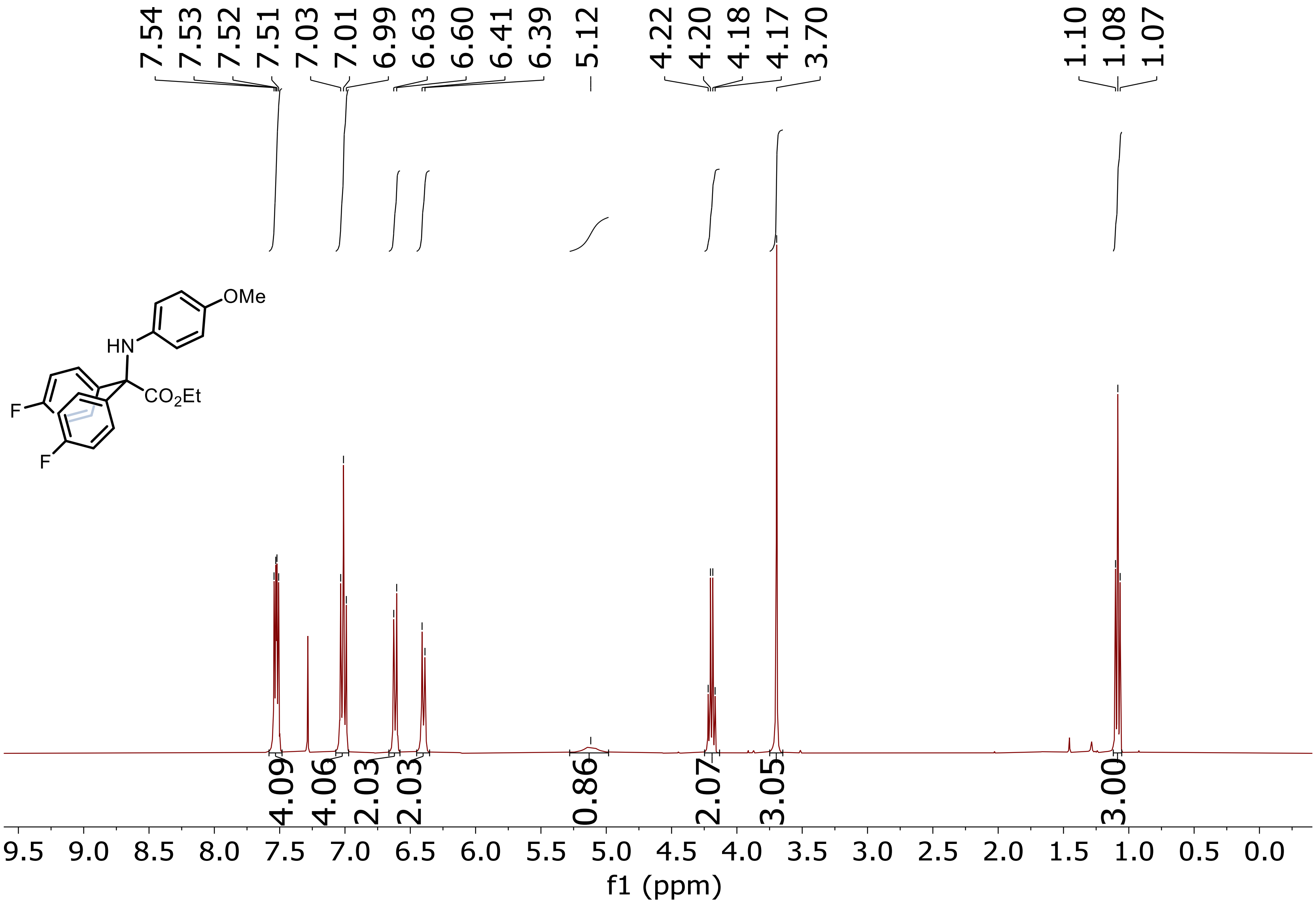

**Ethyl 2,2-bis(4-fluorophenyl)-2-((4-methoxyphenyl)amino)acetate (9) - $^{13}C\{^1H\}$ NMR (101 MHz, $CDCl_3$):**

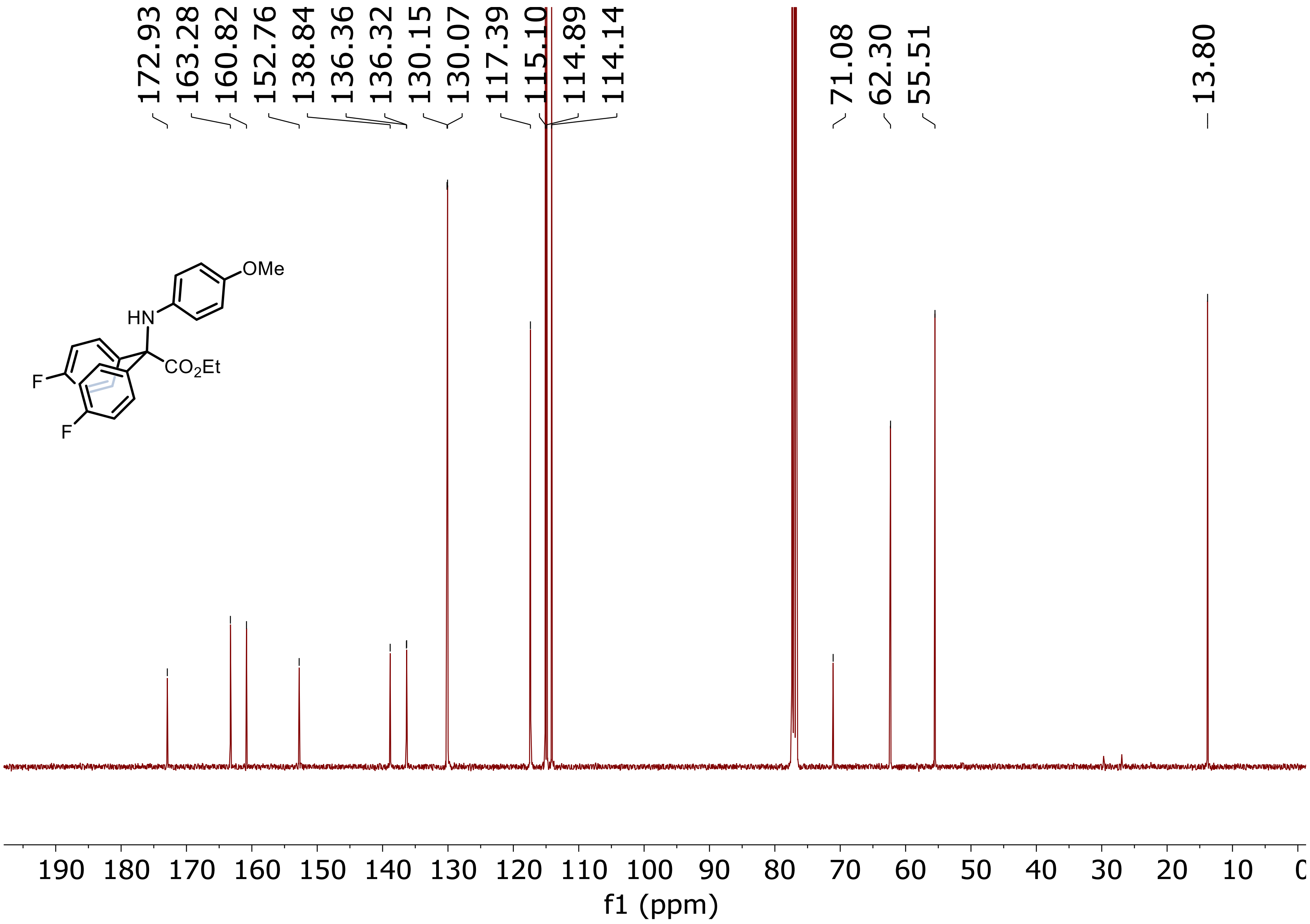

**Ethyl 2,2-bis(4-fluorophenyl)-2-((4-methoxyphenyl)amino)acetate (9) - $^{19}$F NMR (376 MHz, $CDCl_3$):**

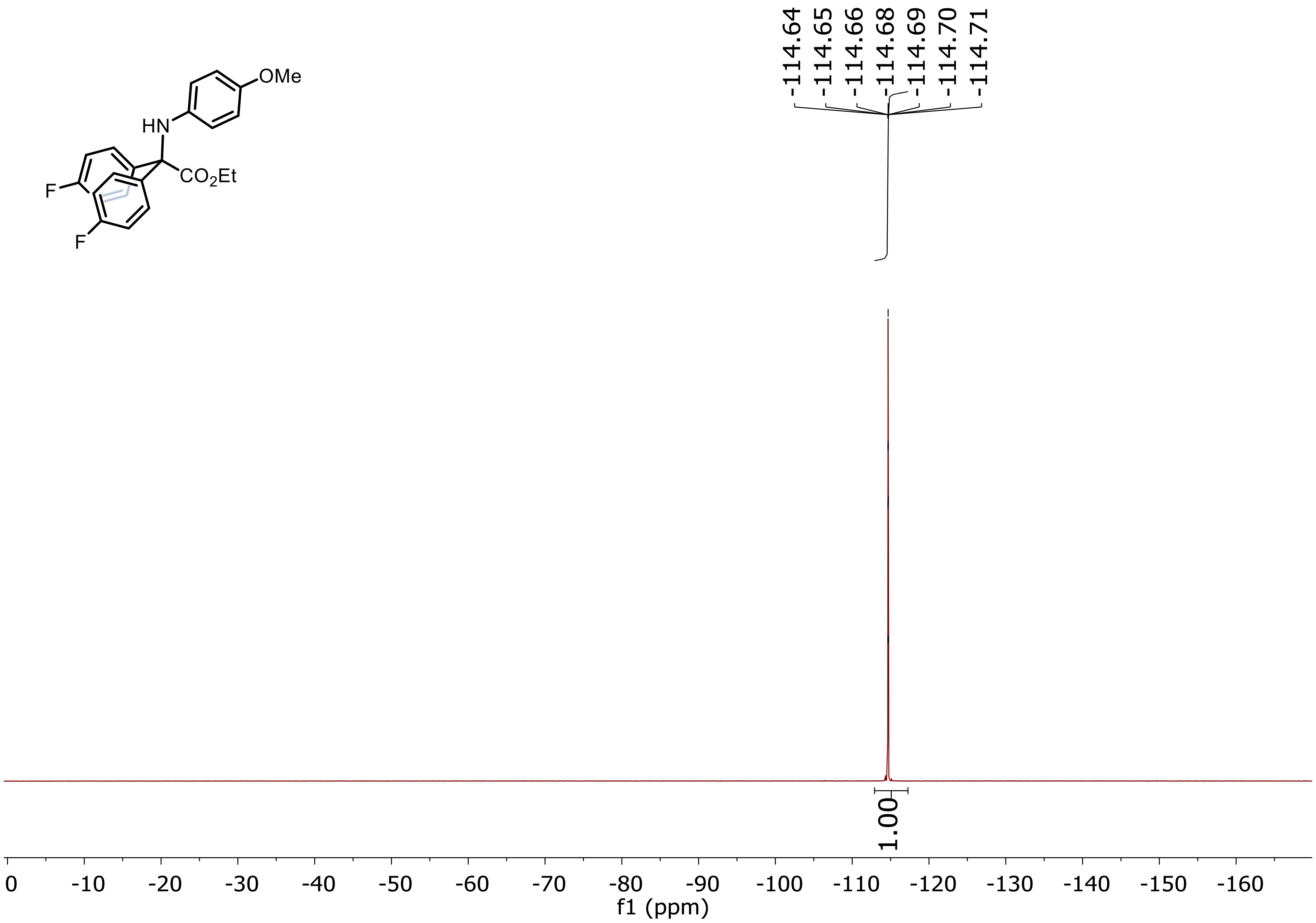

**Ethyl 2,2-bis(4-fluorophenyl)-2-(p-tolylamino)acetate (10) - $^{1}$H NMR (400 MHz, $CDCl_3$):**

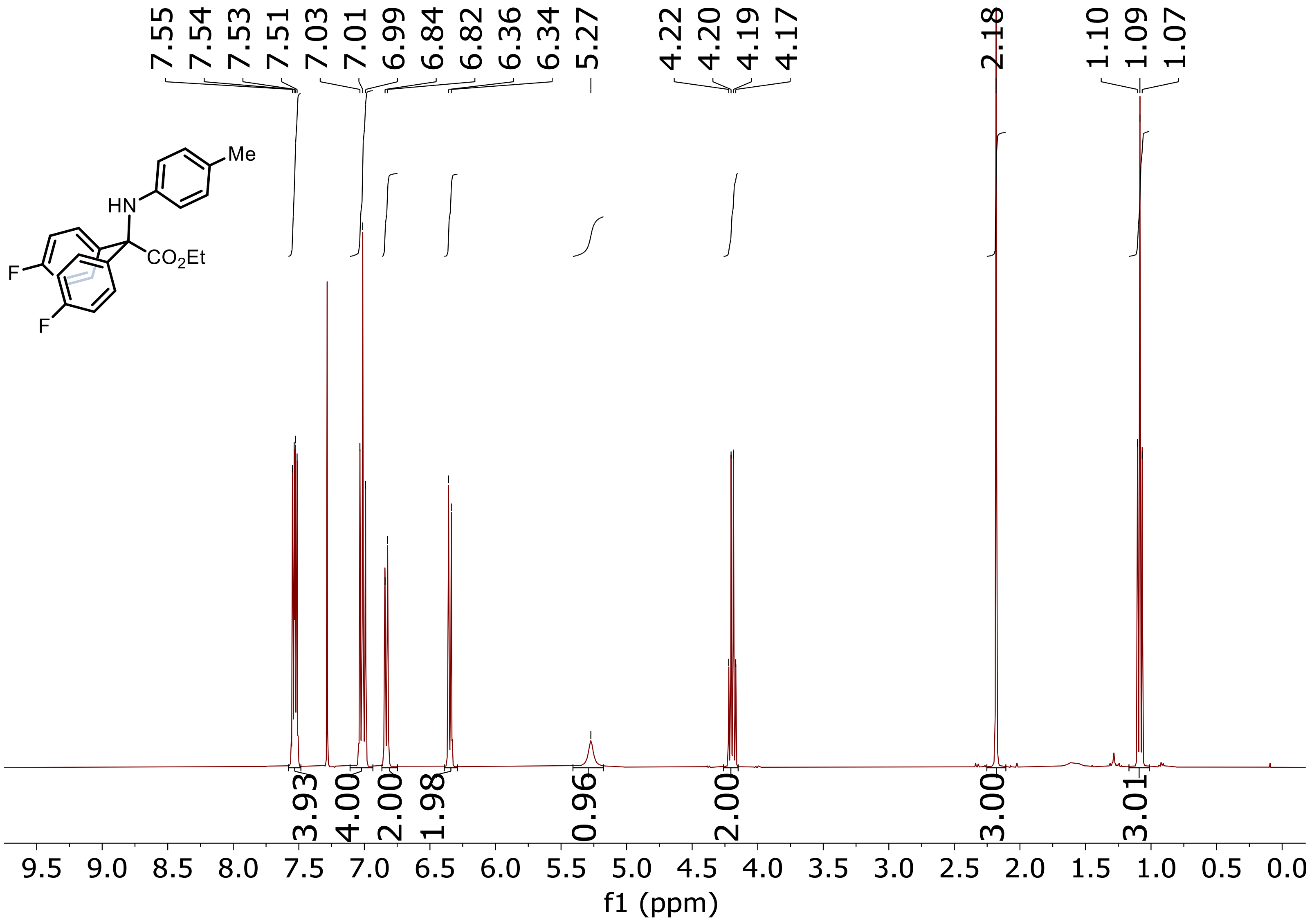

**Ethyl 2,2-bis(4-fluorophenyl)-2-(p-tolylamino)acetate (10) - $^{13}C\{^1H\}$ NMR (101 MHz, $CDCl_3$):**

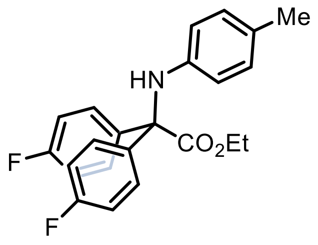


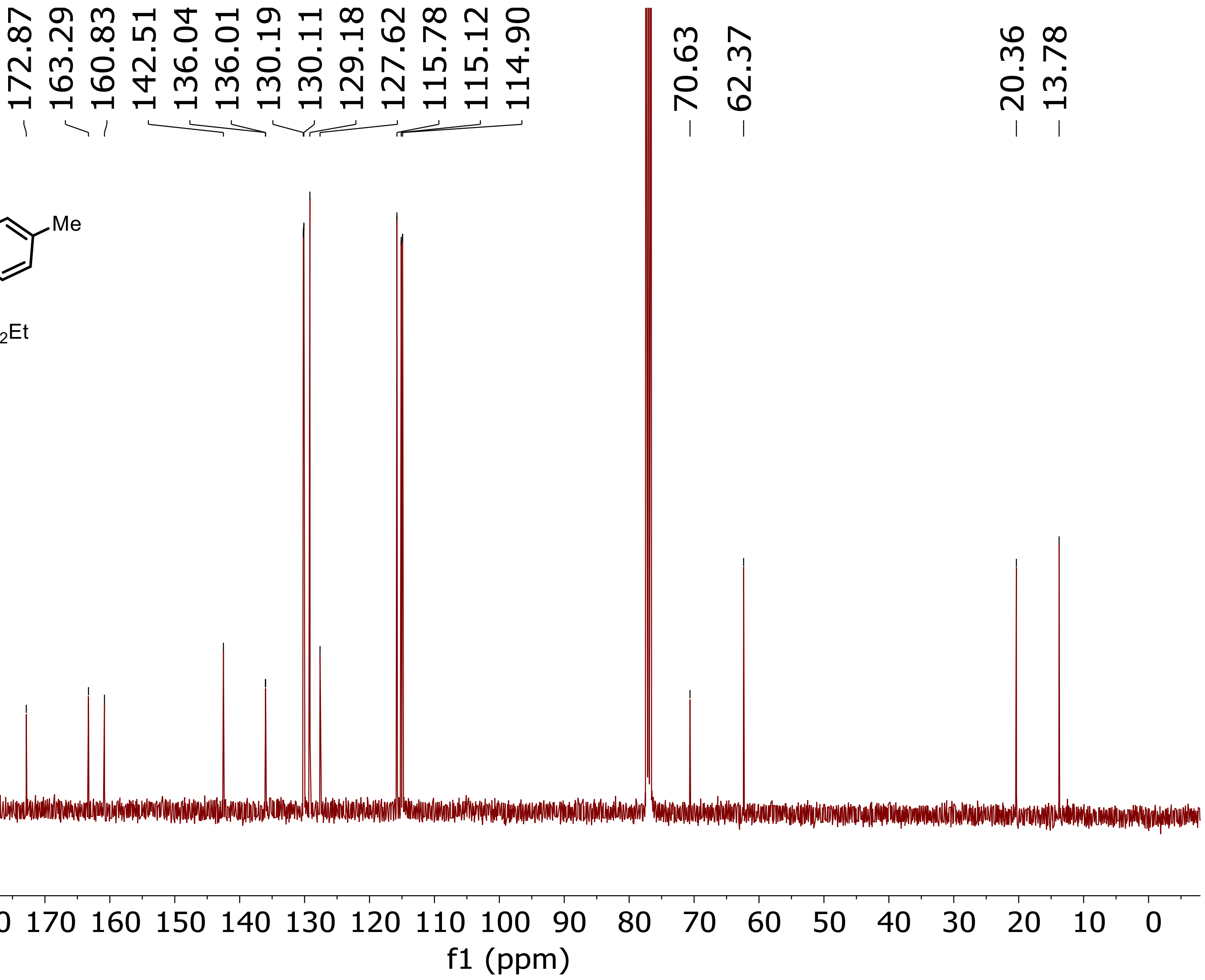

**Ethyl 2,2-bis(4-fluorophenyl)-2-(p-tolylamino)acetate (10) - $^{19}$F NMR (376 MHz, $CDCl_3$):**

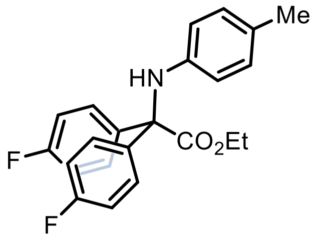

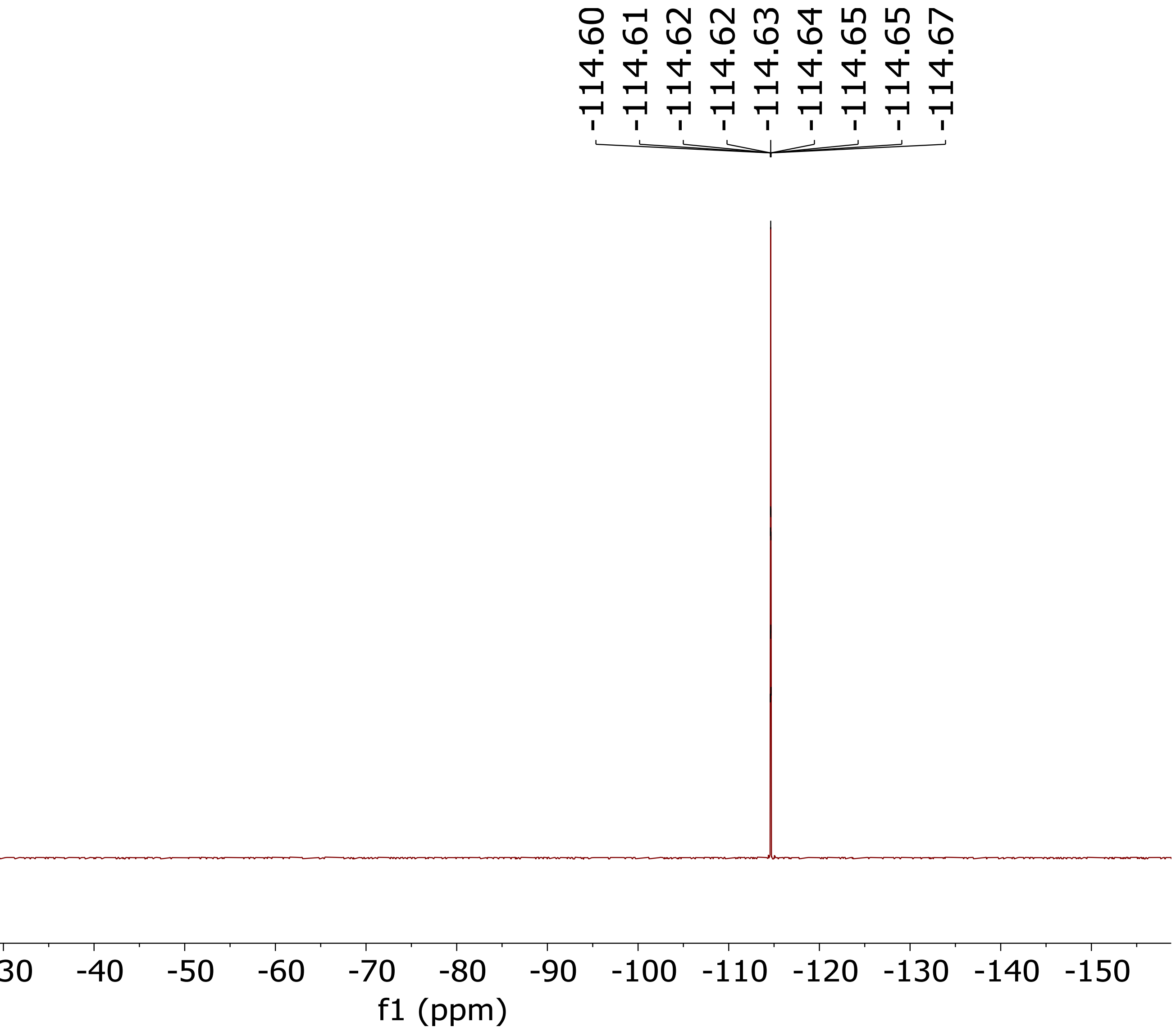

**Ethyl 4-((2-ethoxy-1,1-bis(4-fluorophenyl)-2-oxoethyl)amino)benzoate (11) - $^{1}$H NMR (400 MHz, $CDCl_3$):**

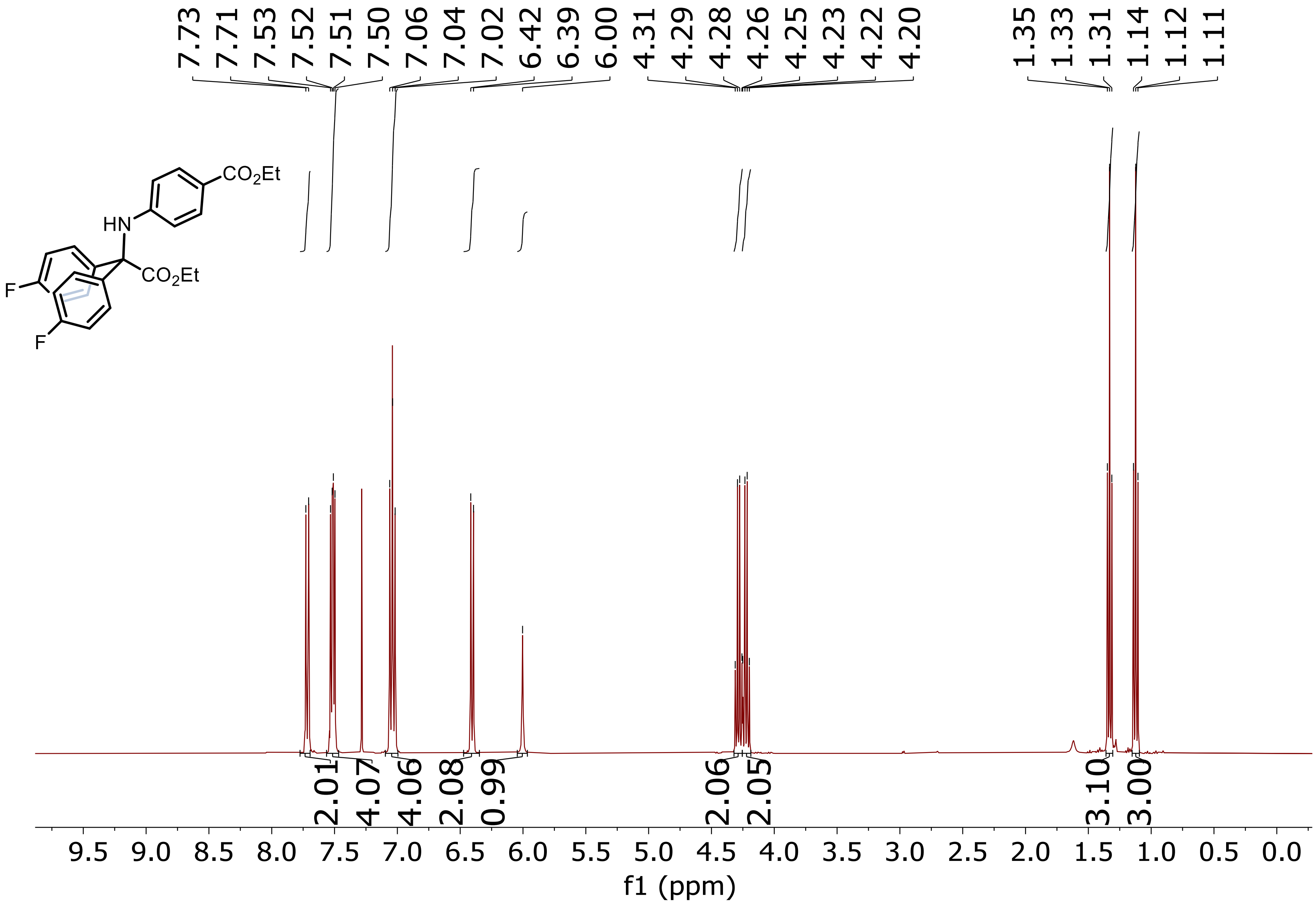

**Ethyl 4-((2-ethoxy-1,1-bis(4-fluorophenyl)-2-oxoethyl)amino)benzoate (11) - $^{13}C\{^1H\}$ NMR (101 MHz, CDCl$_3$):**

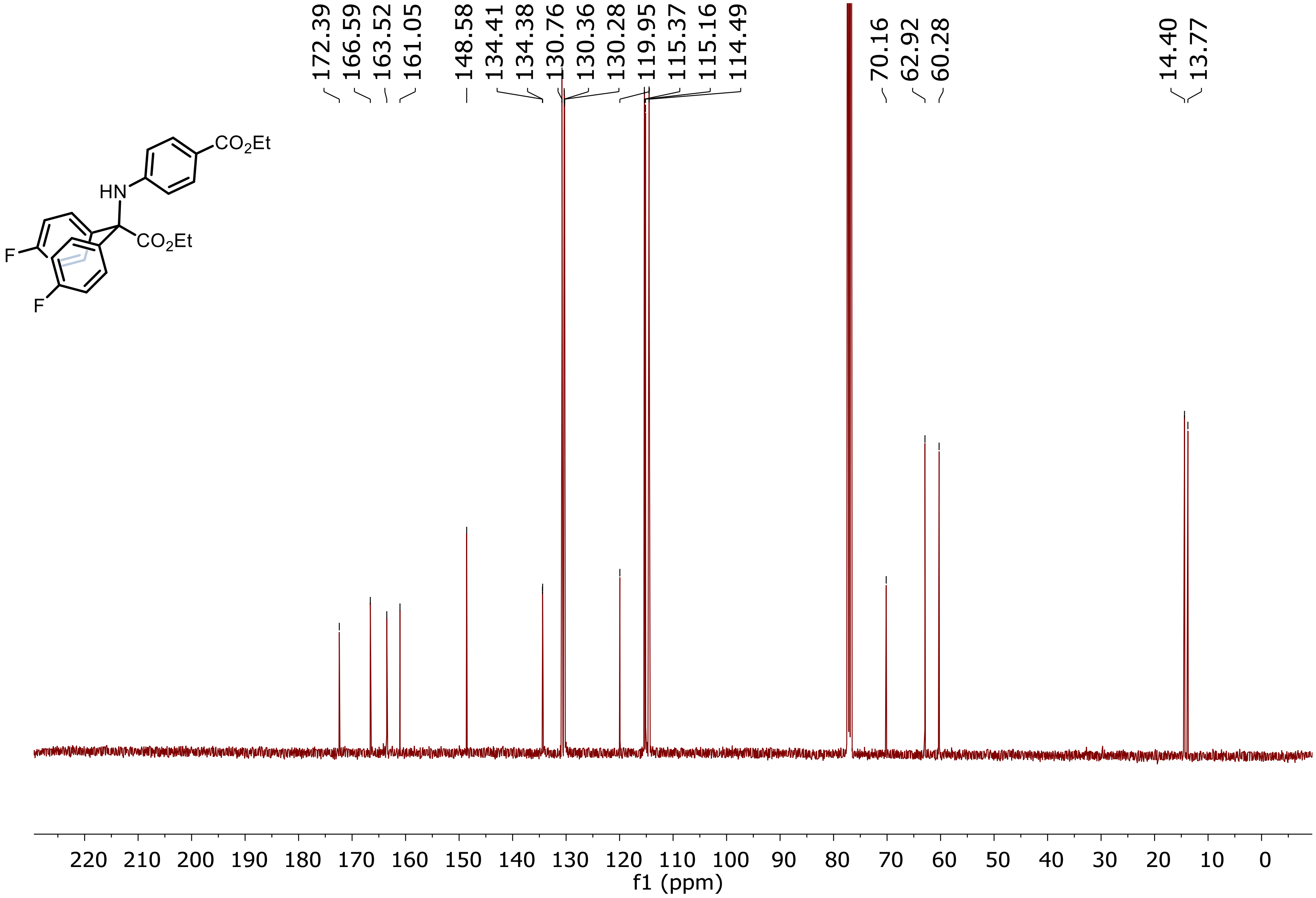

**Ethyl 4-((2-ethoxy-1,1-bis(4-fluorophenyl)-2-oxoethyl)amino)benzoate (11) - $^{19}$F NMR (376 MHz, $CDCl_3$):**

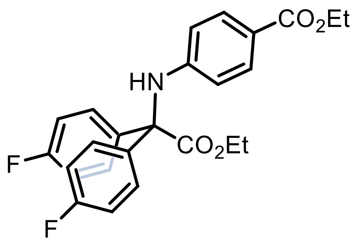


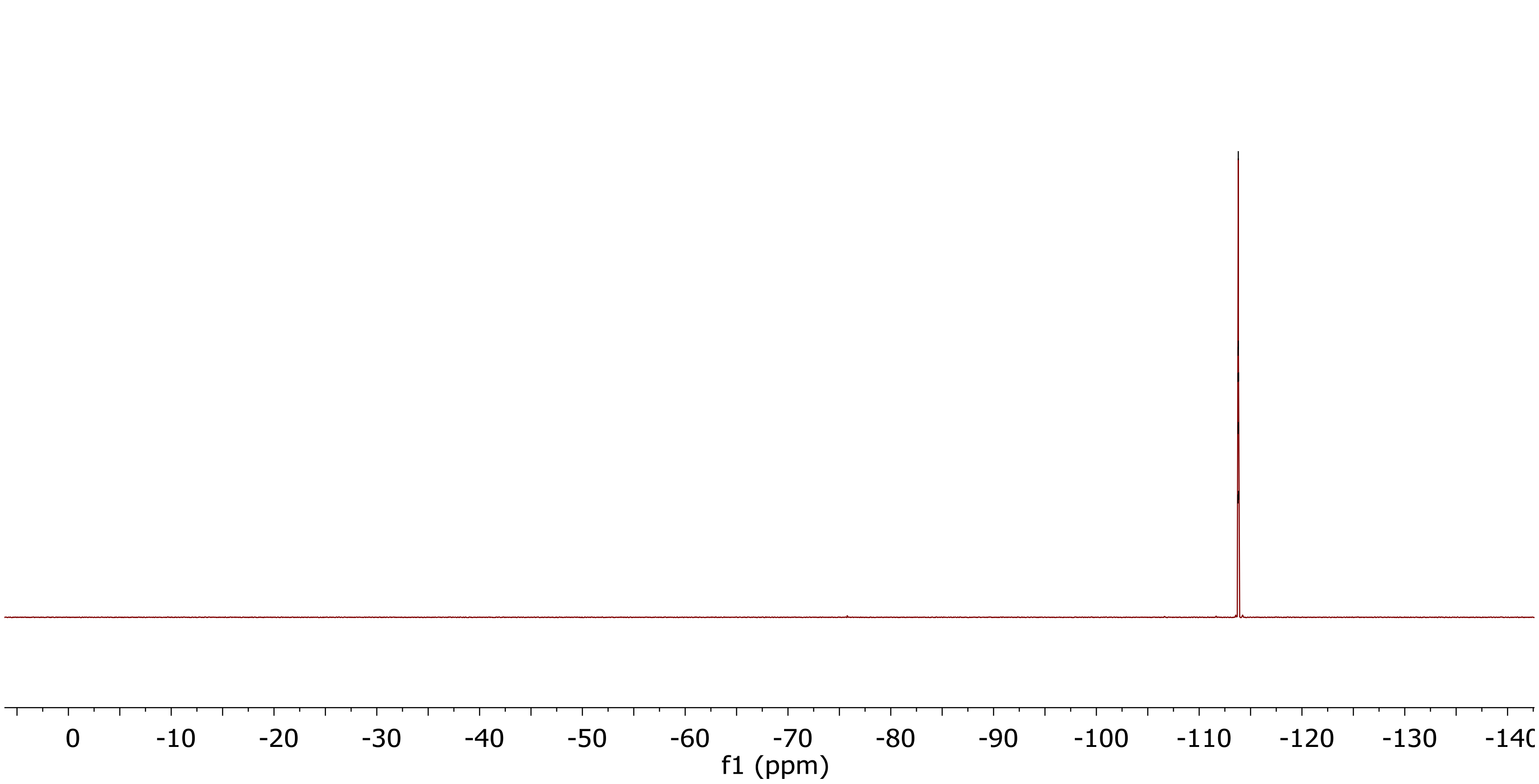

**Ethyl 2,2-bis(4-fluorophenyl)-2-((4-(trifluoromethyl)phenyl)amino)acetate (12) - $^{1}$H NMR (400 MHz, $CDCl_3$):**

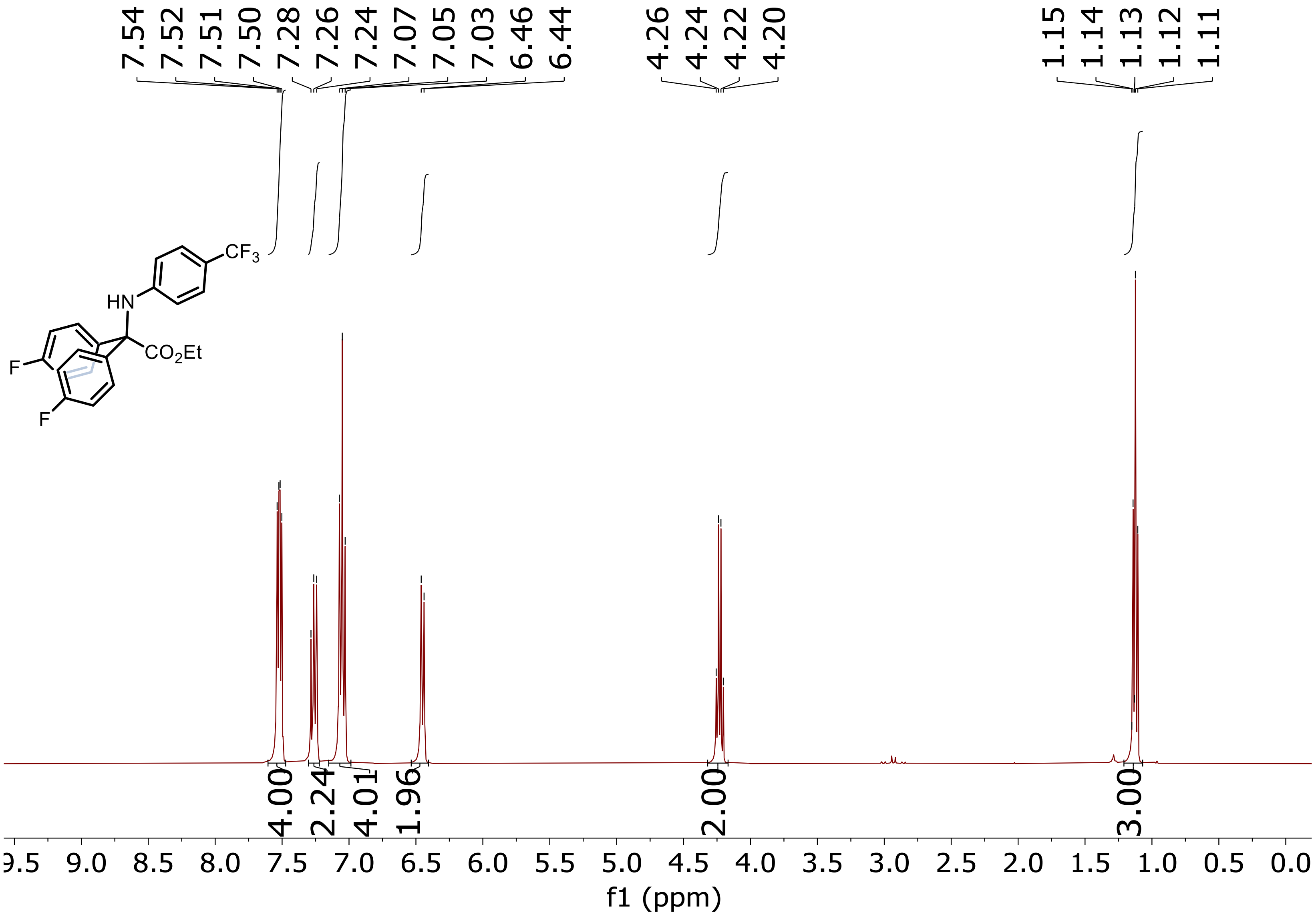

**Ethyl 2,2-bis(4-fluorophenyl)-2-((4-(trifluoromethyl)phenyl)amino)acetate (12) - $^{13}C\{^1H\}$ NMR (101 MHz, $CDCl_3$):**

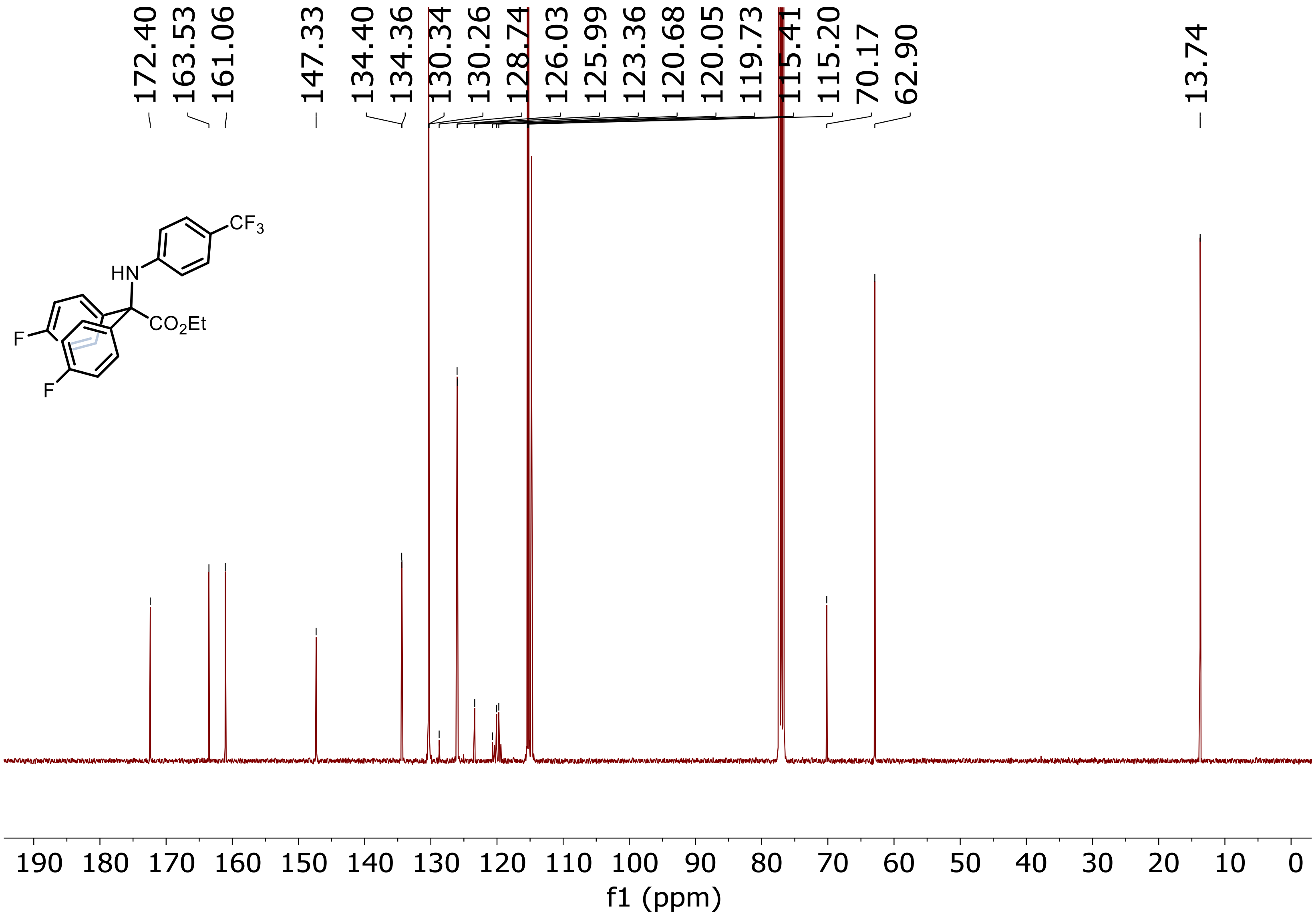

**Ethyl 2,2-bis(4-fluorophenyl)-2-((4-(trifluoromethyl)phenyl)amino)acetate (12) - $^{19}$F NMR (376 MHz, CDCl₃):**

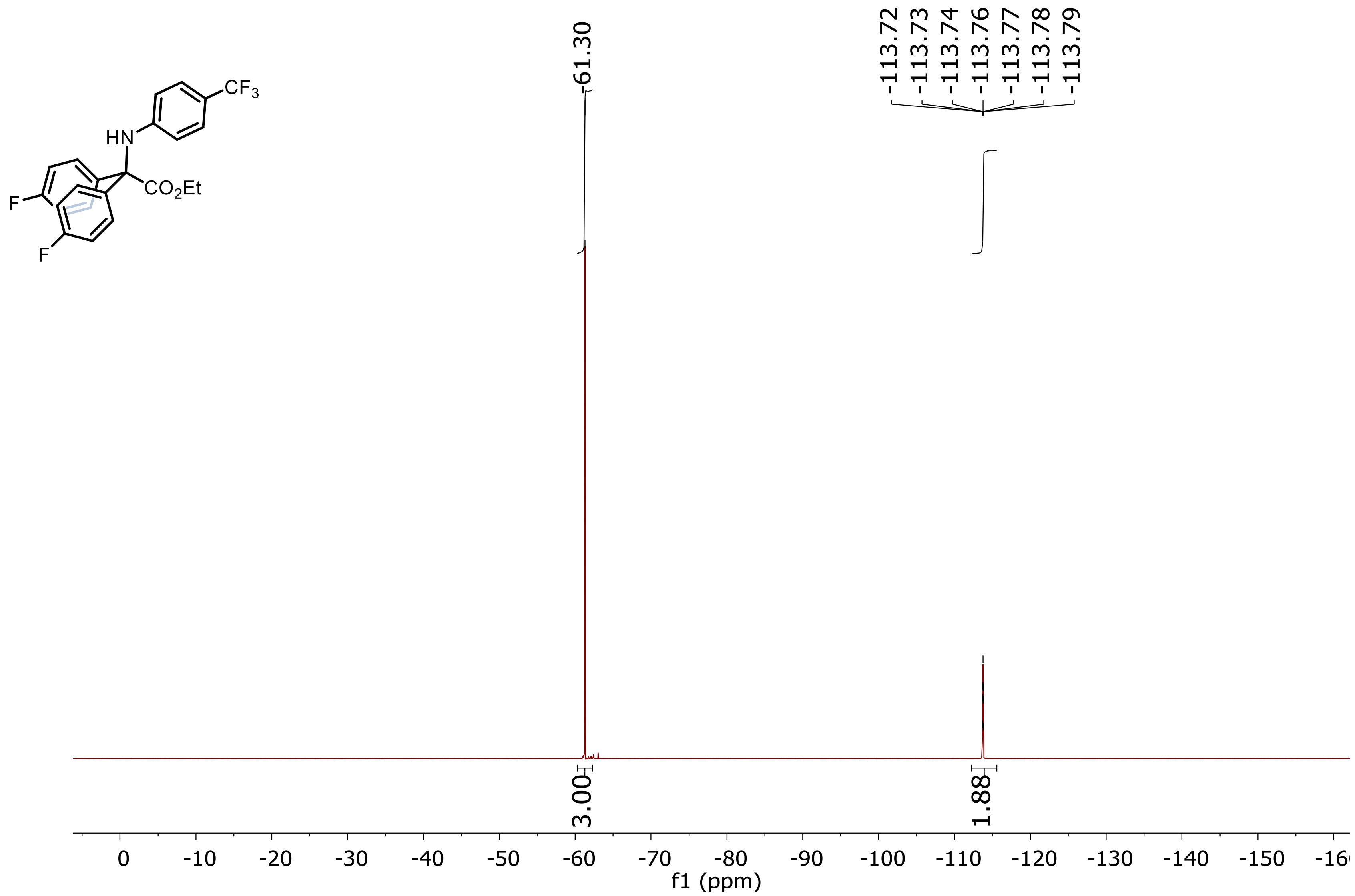

**Ethyl 2-((3-bromophenyl)amino)-2,2-bis(4-fluorophenyl)acetate (13) - $^{1}H$ NMR (400 MHz, $CDCl_3$):**

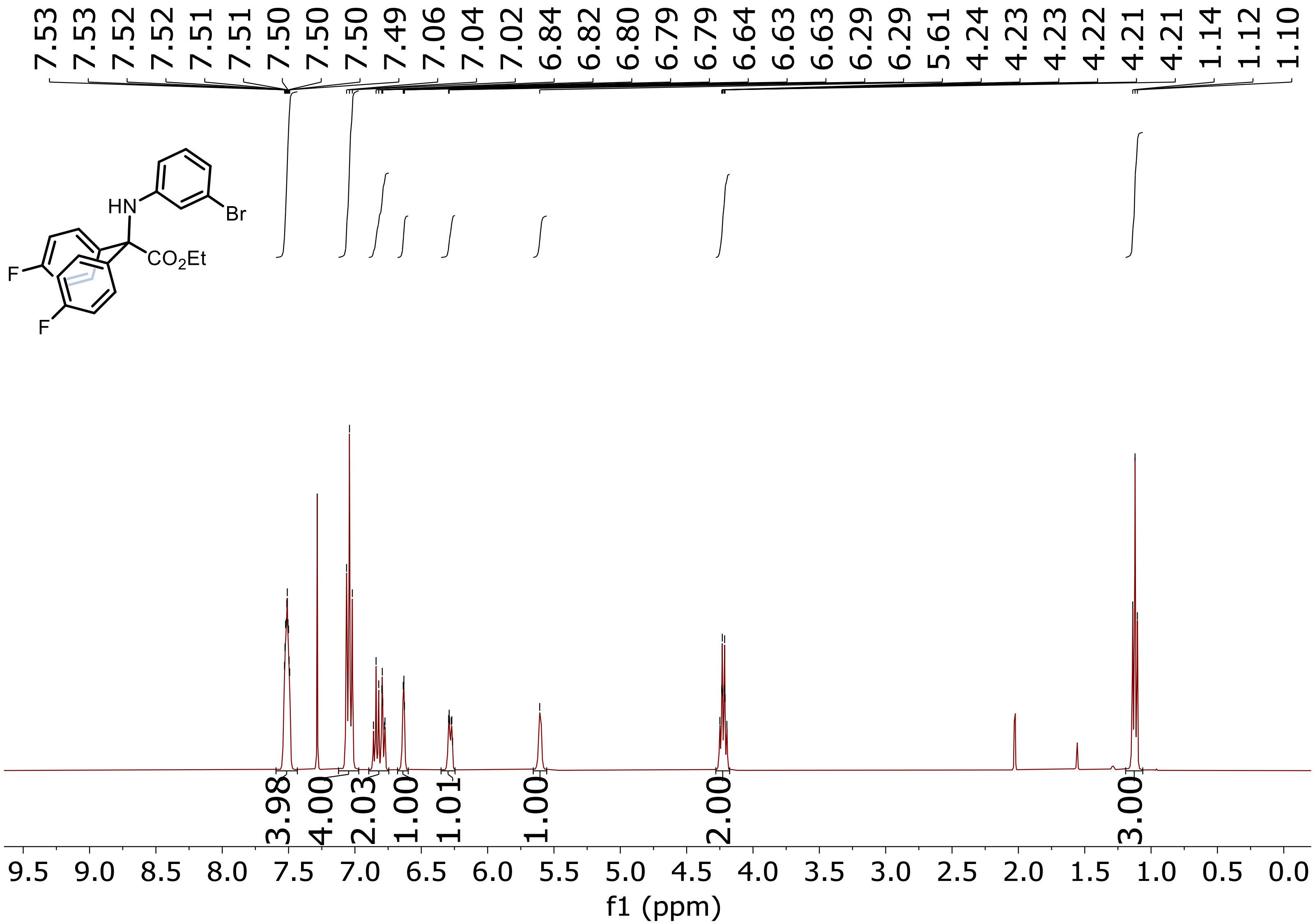

**Ethyl 2-((3-bromophenyl)amino)-2,2-bis(4-fluorophenyl)acetate (13) - $^{13}C\{^{1}H\}$ NMR (101 MHz, $CDCl_3$):**

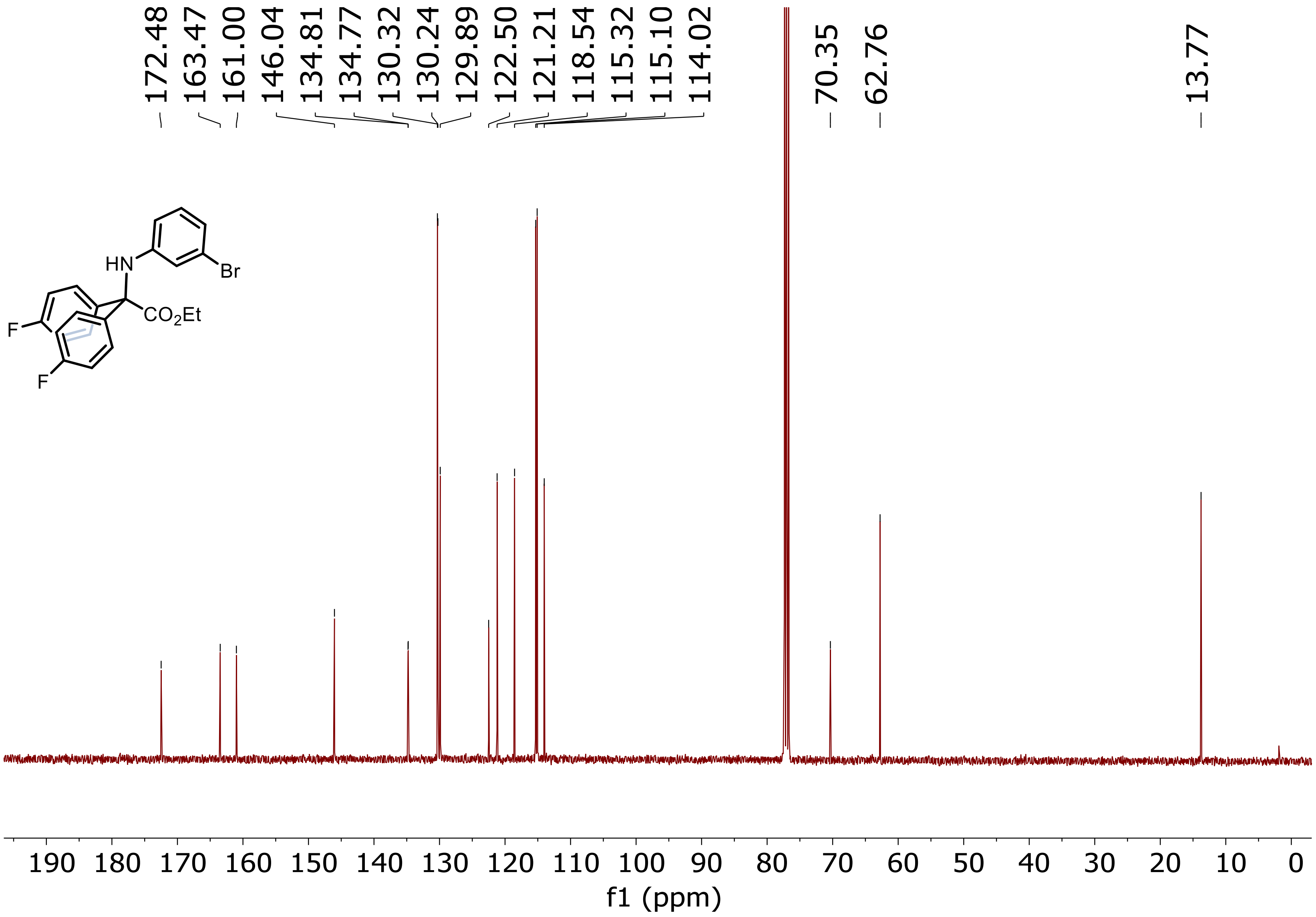

**Ethyl 2-((3-bromophenyl)amino)-2,2-bis(4-fluorophenyl)acetate (13) - $^{19}$F NMR (376 MHz, $CDCl_3$):**

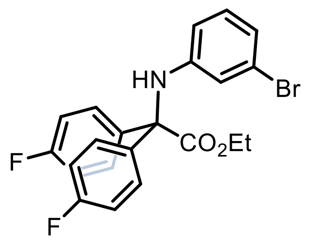


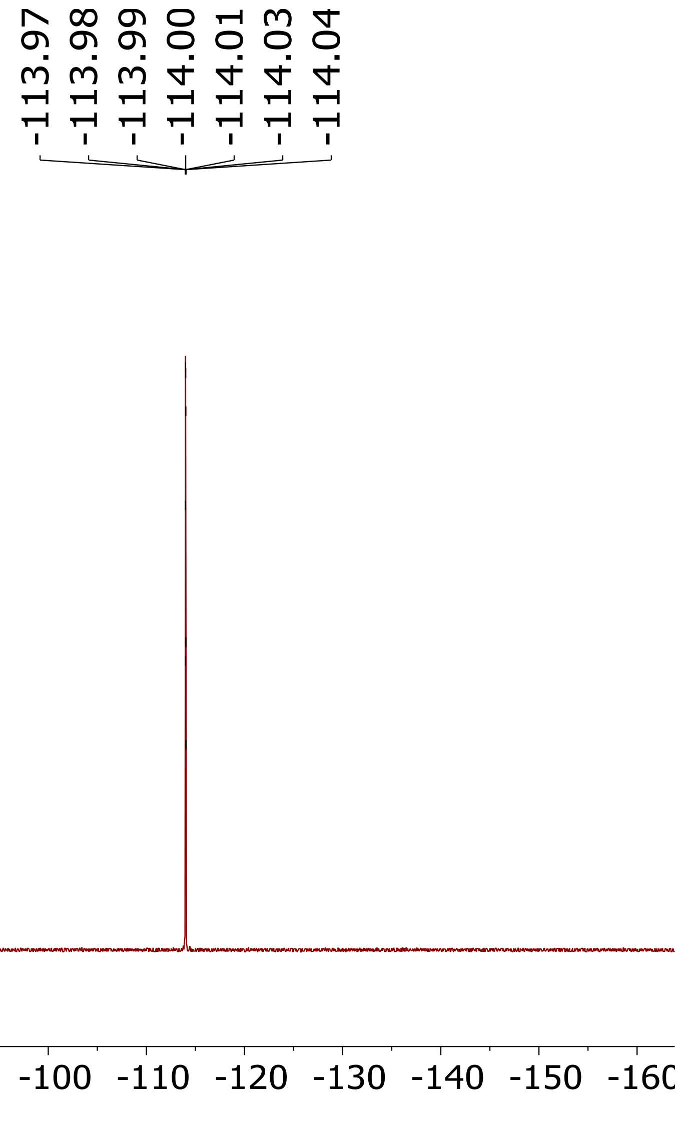

**Ethyl 2-((3-ethynylphenyl)amino)-2,2-bis(4-fluorophenyl)acetate (14) - $^{1}$H NMR (400 MHz, $CDCl_3$):**

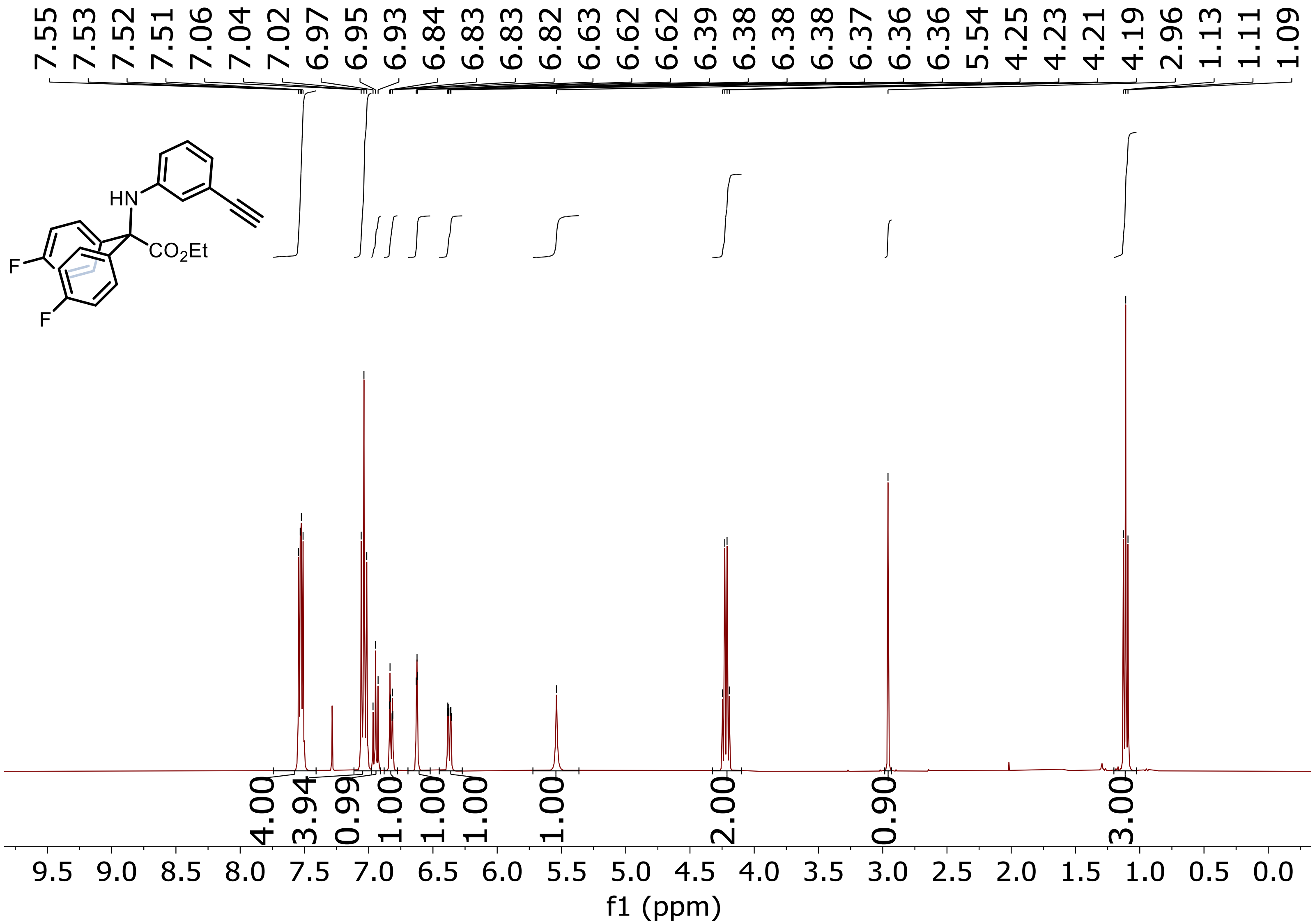

**Ethyl 2-((3-ethynylphenyl)amino)-2,2-bis(4-fluorophenyl)acetate (14) - $^{13}C\{^1H\}$ NMR (101 MHz, $CDCl_3$):**

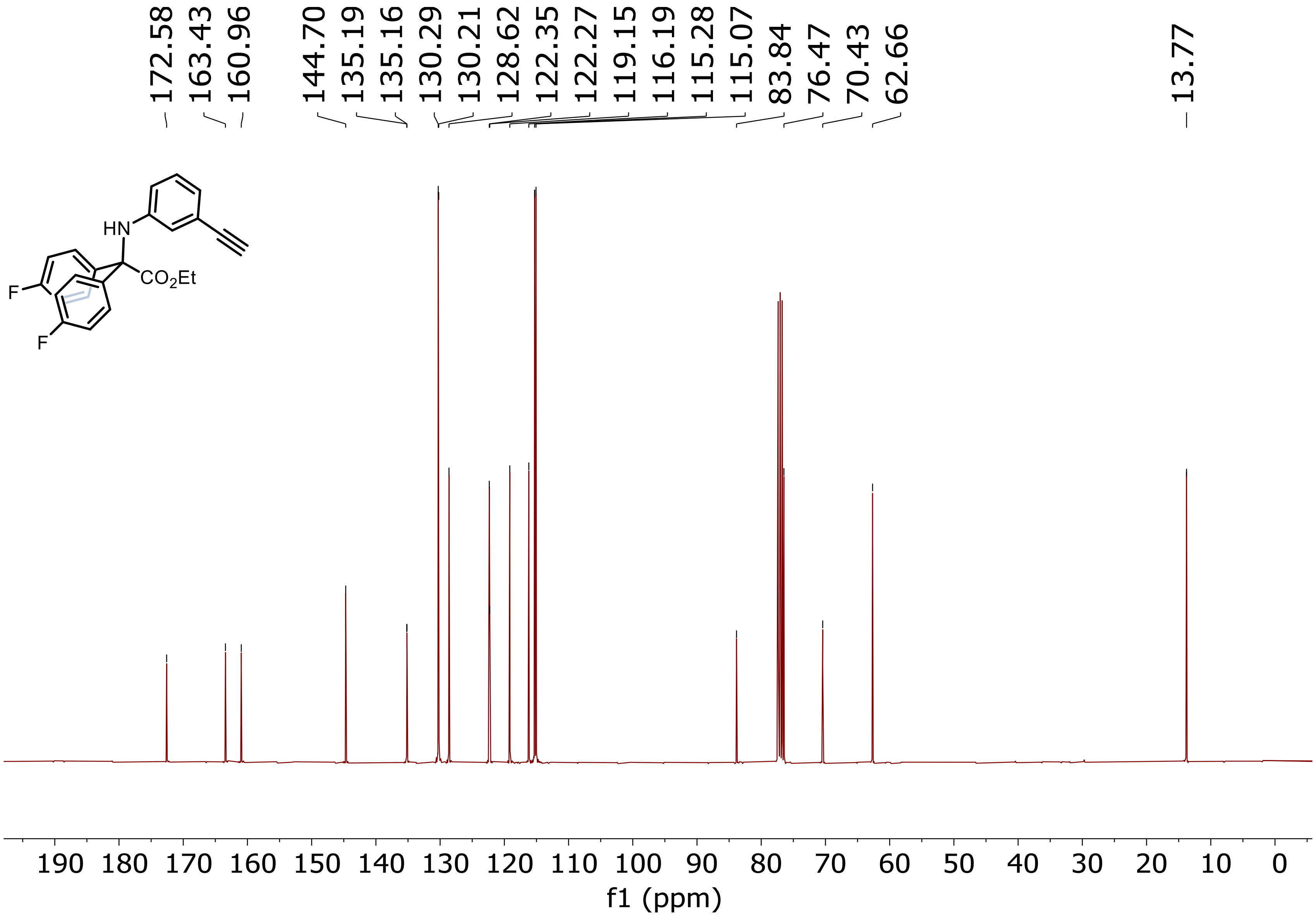

**Ethyl 2-((3-ethynylphenyl)amino)-2,2-bis(4-fluorophenyl)acetate (14) - $^{19}$F NMR (376 MHz, $CDCl_3$):**

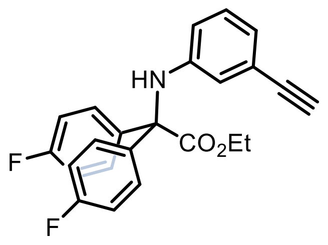


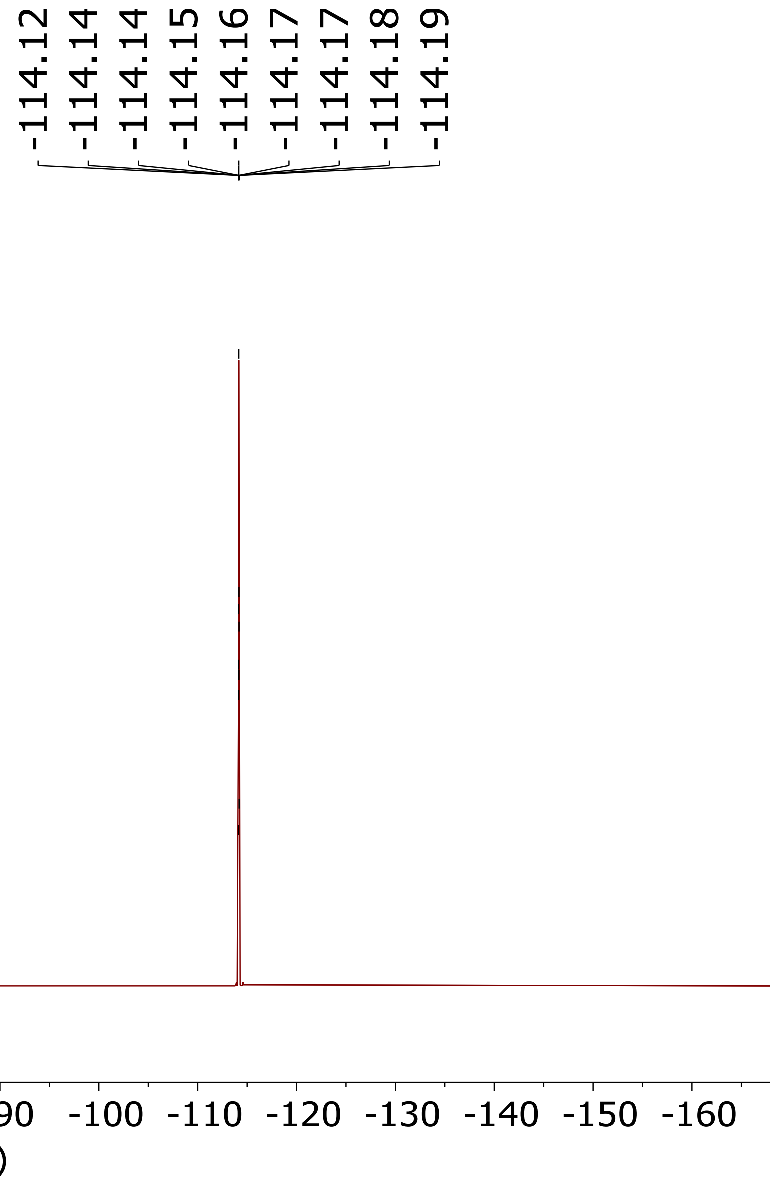

**Ethyl 2-((2,4-dimethylphenyl)amino)-2,2-bis(4-fluorophenyl)acetate (15) - $^1$H NMR (400 MHz, $CDCl_3$):**

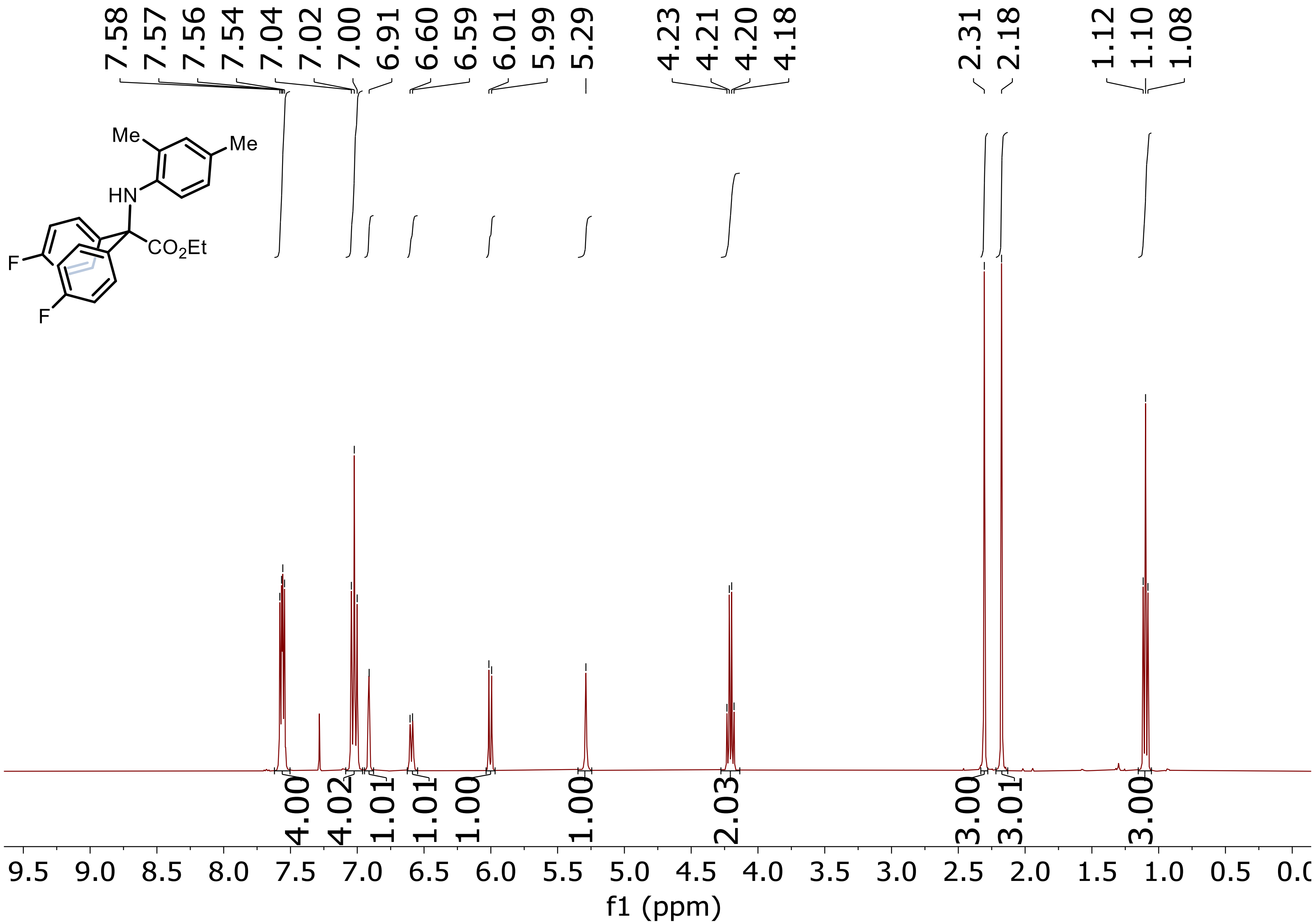

**Ethyl 2-((2,4-dimethylphenyl)amino)-2,2-bis(4-fluorophenyl)acetate (15) - $^{13}C\{^1H\}$ NMR (101 MHz, $CDCl_3$):**

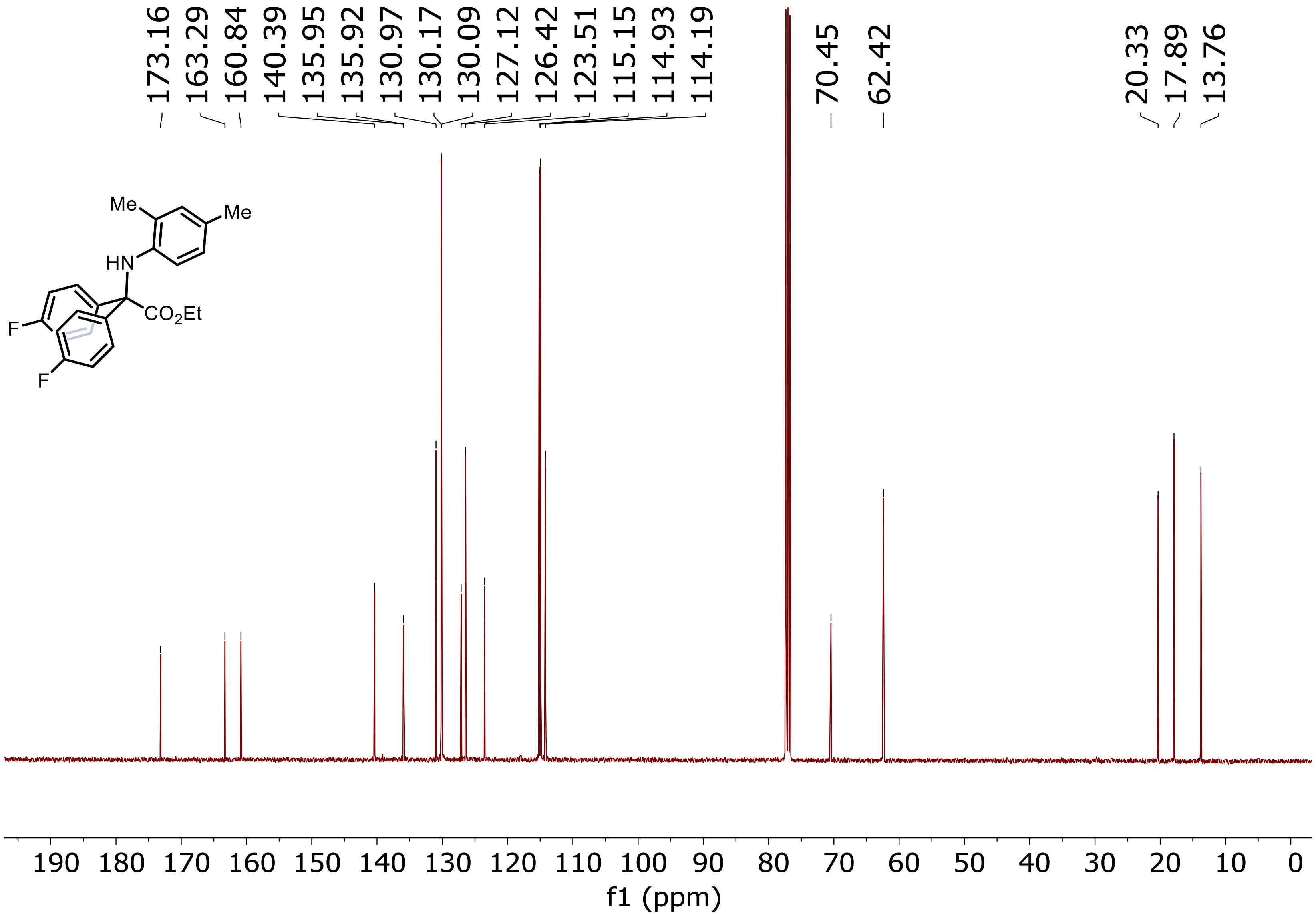

**Ethyl 2-((2,4-dimethylphenyl)amino)-2,2-bis(4-fluorophenyl)acetate (15) - $^{19}$F NMR (376 MHz, $CDCl_3$):**

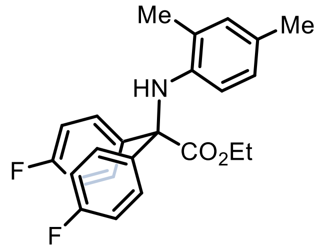


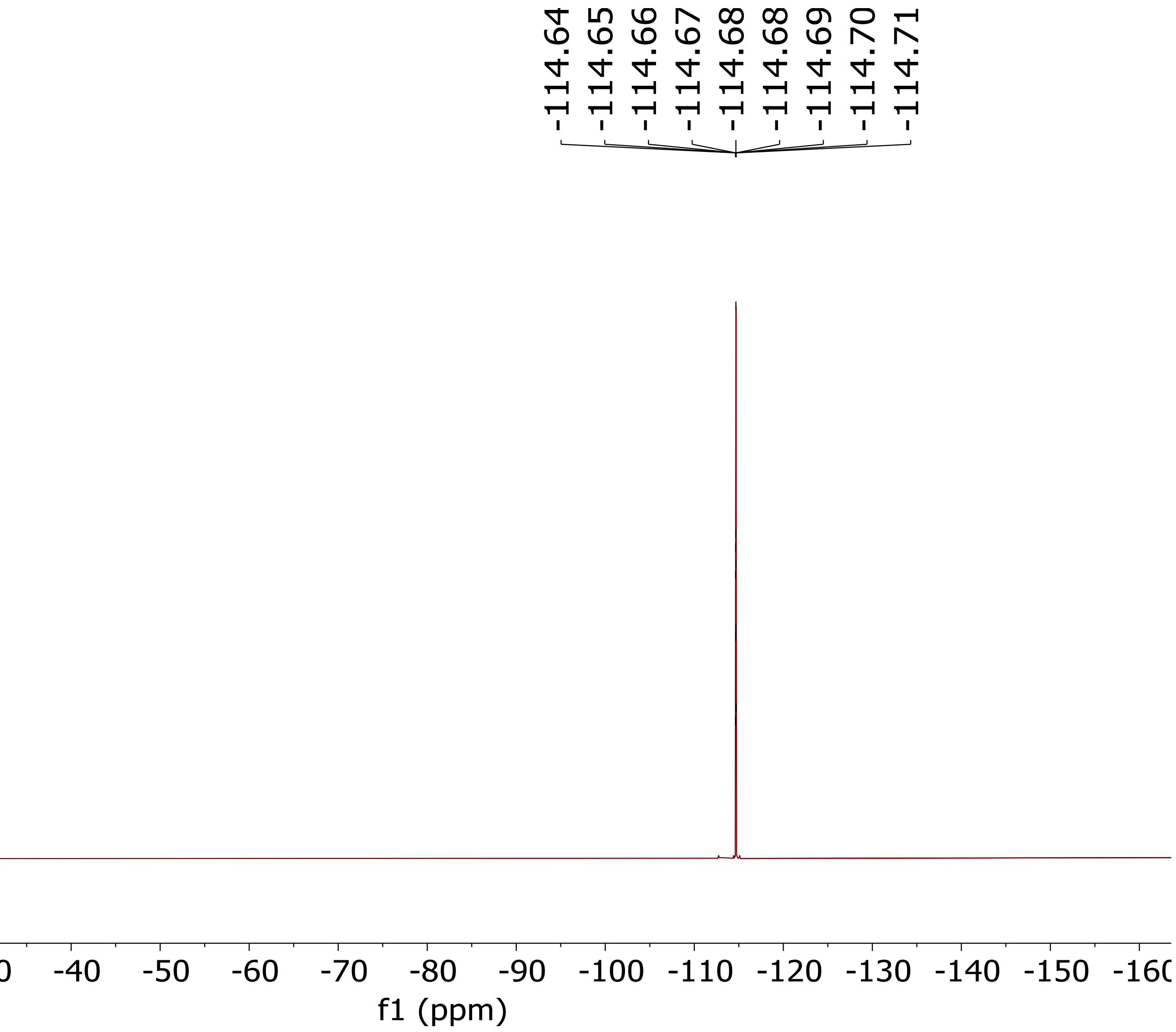

**Ethyl 2,2-bis(4-fluorophenyl)-2-(propyllamino)acetate (16) - $^{1}$H NMR (400 MHz, $CDCl_3$):**

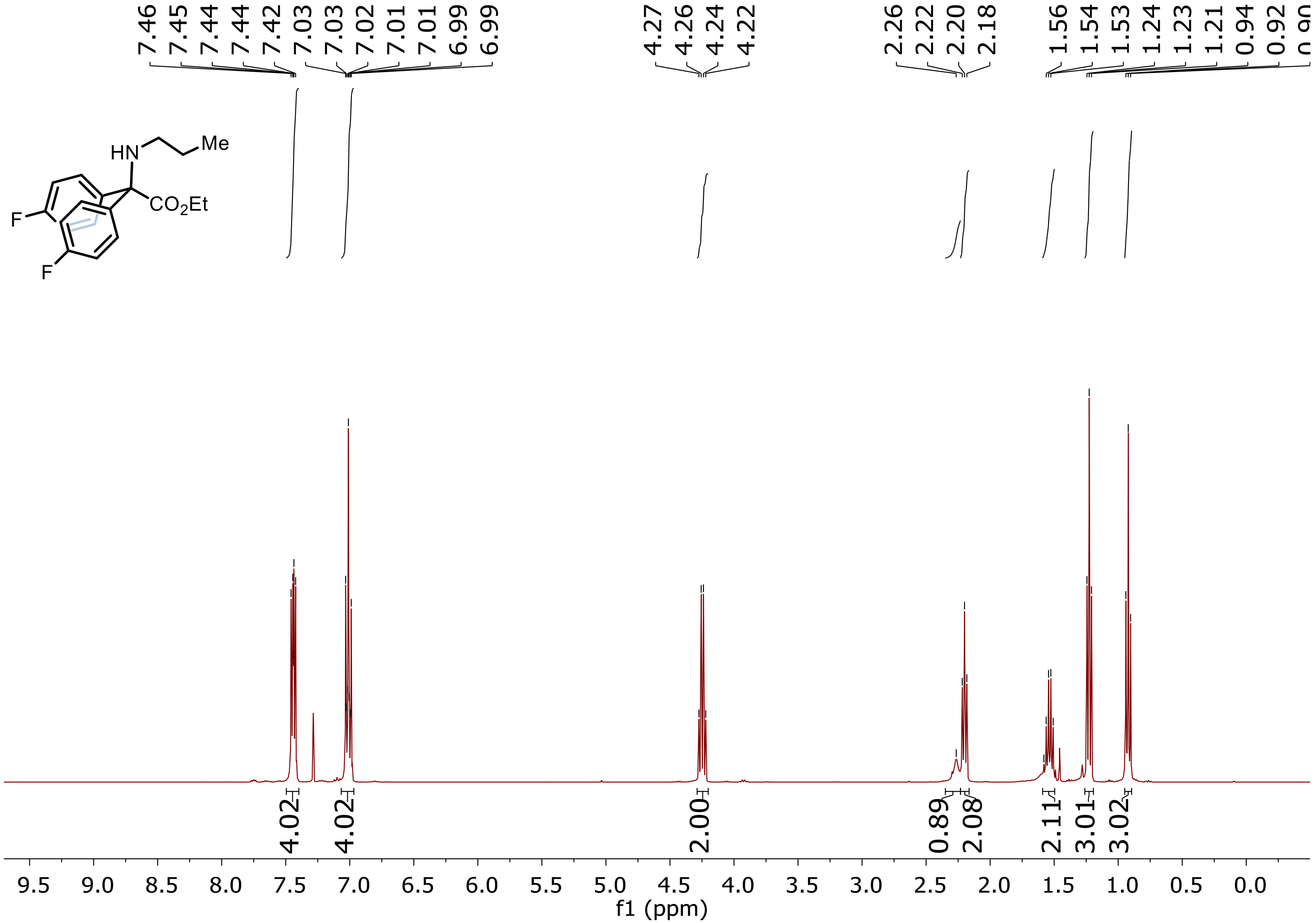

**Ethyl 2,2-bis(4-fluorophenyl)-2-(propyllamino)acetate (16) - $^{13}C\{^{1}H\}$ NMR (101 MHz, $CDCl_3$):**

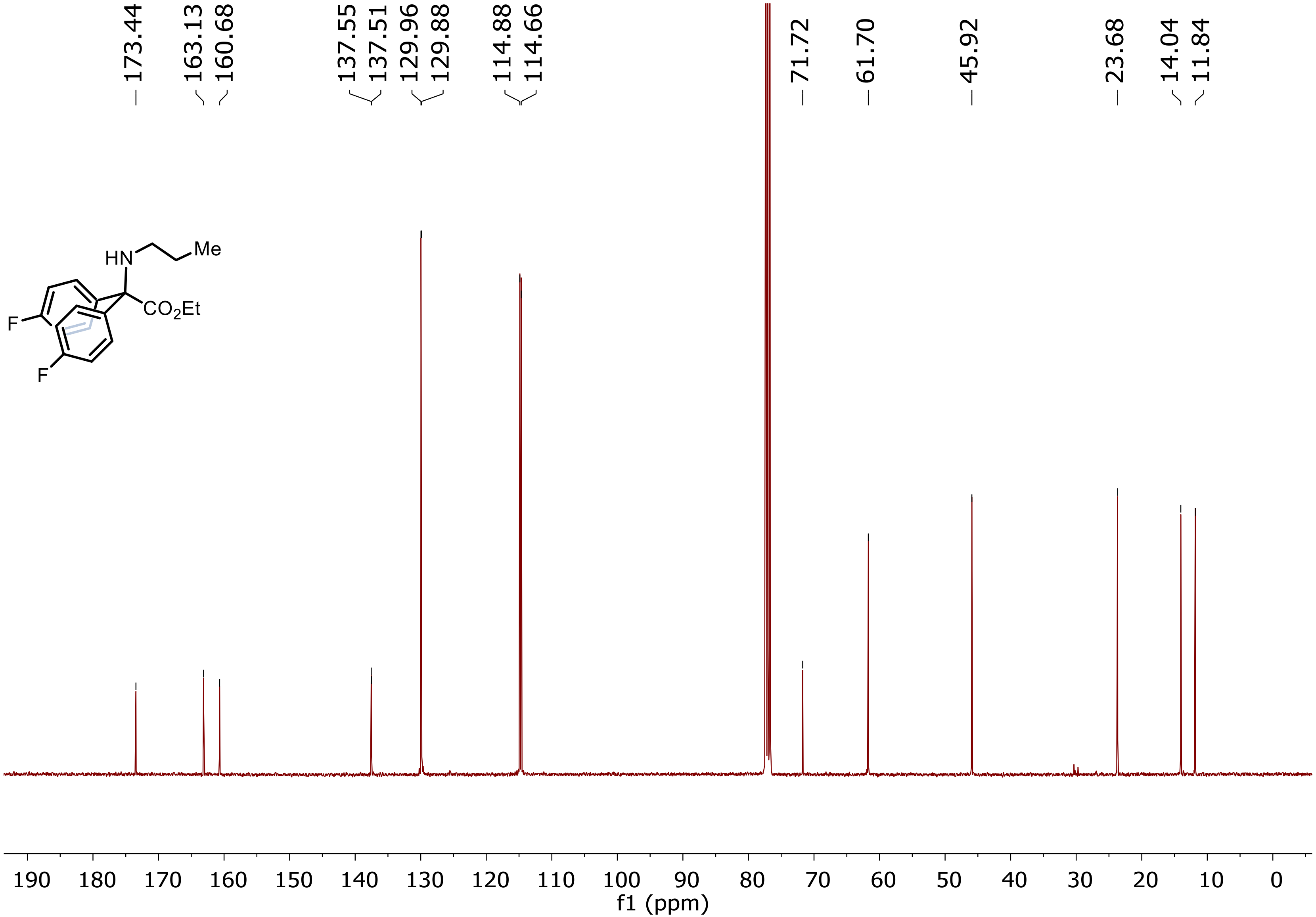

**Ethyl 2,2-bis(4-fluorophenyl)-2-(propyllamino)acetate (16) - $^{19}$F NMR (376 MHz, $CDCl_3$):**

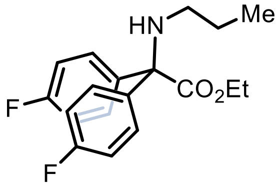


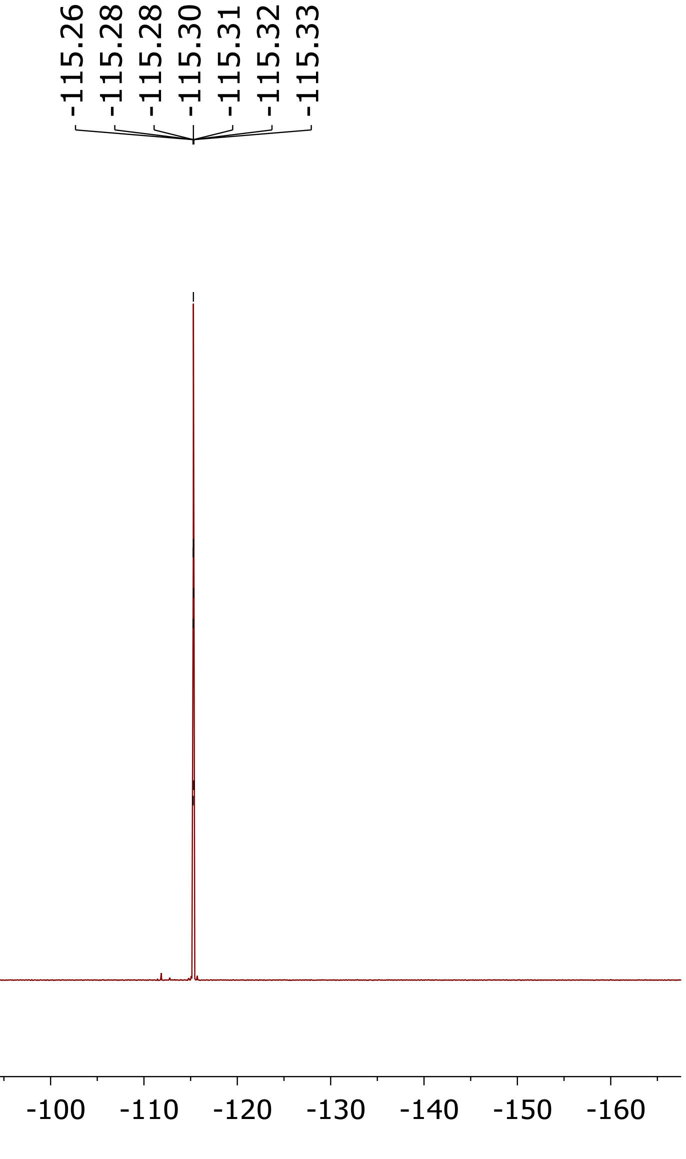

**Ethyl 2-(allylamino)-2,2-bis(4-fluorophenyl) acetate (17) - $^{1}H$ NMR (400 MHz, $CDCl_3$):**

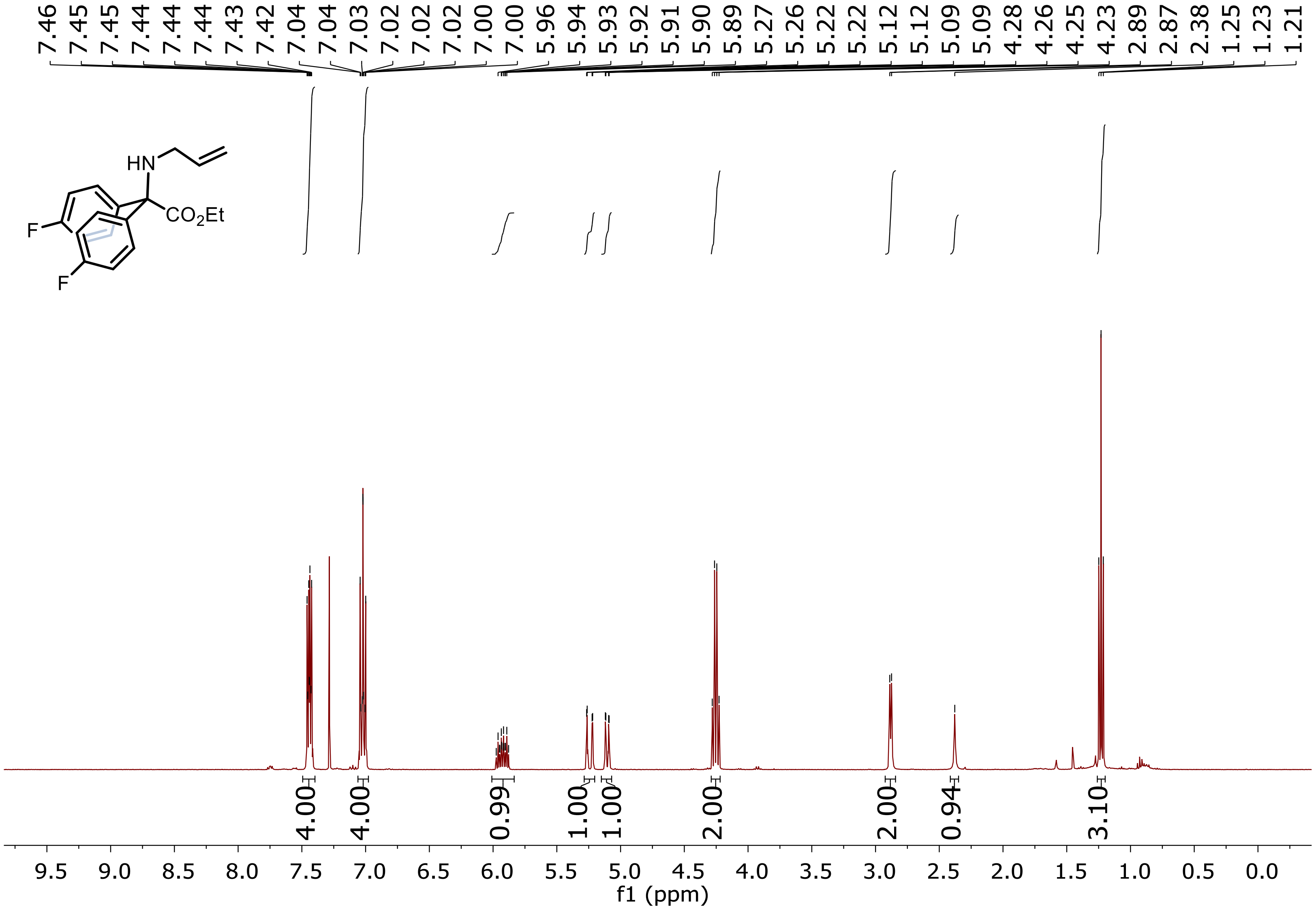

**Ethyl 2-(allylamino)-2,2-bis(4-fluorophenyl) acetate (17) - $^{13}C\{^{1}H\}$ NMR (101 MHz, $CDCl_3$):**

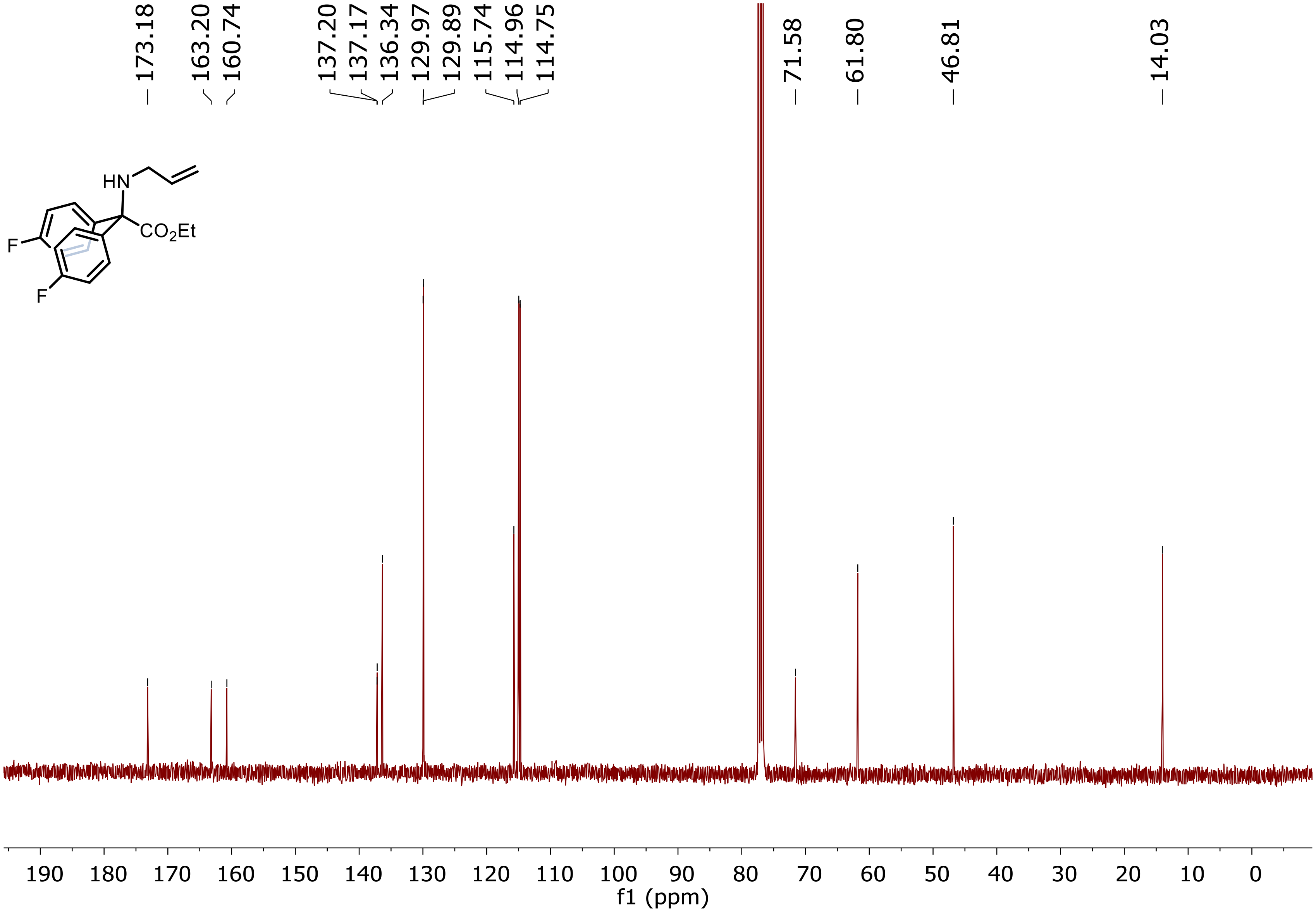

**Ethyl 2-(allylamino)-2,2-bis(4-fluorophenyl) acetate (17) - $^{19}$F NMR (376 MHz, $CDCl_3$):**

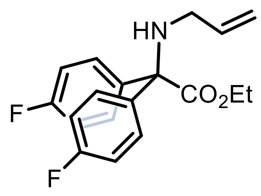


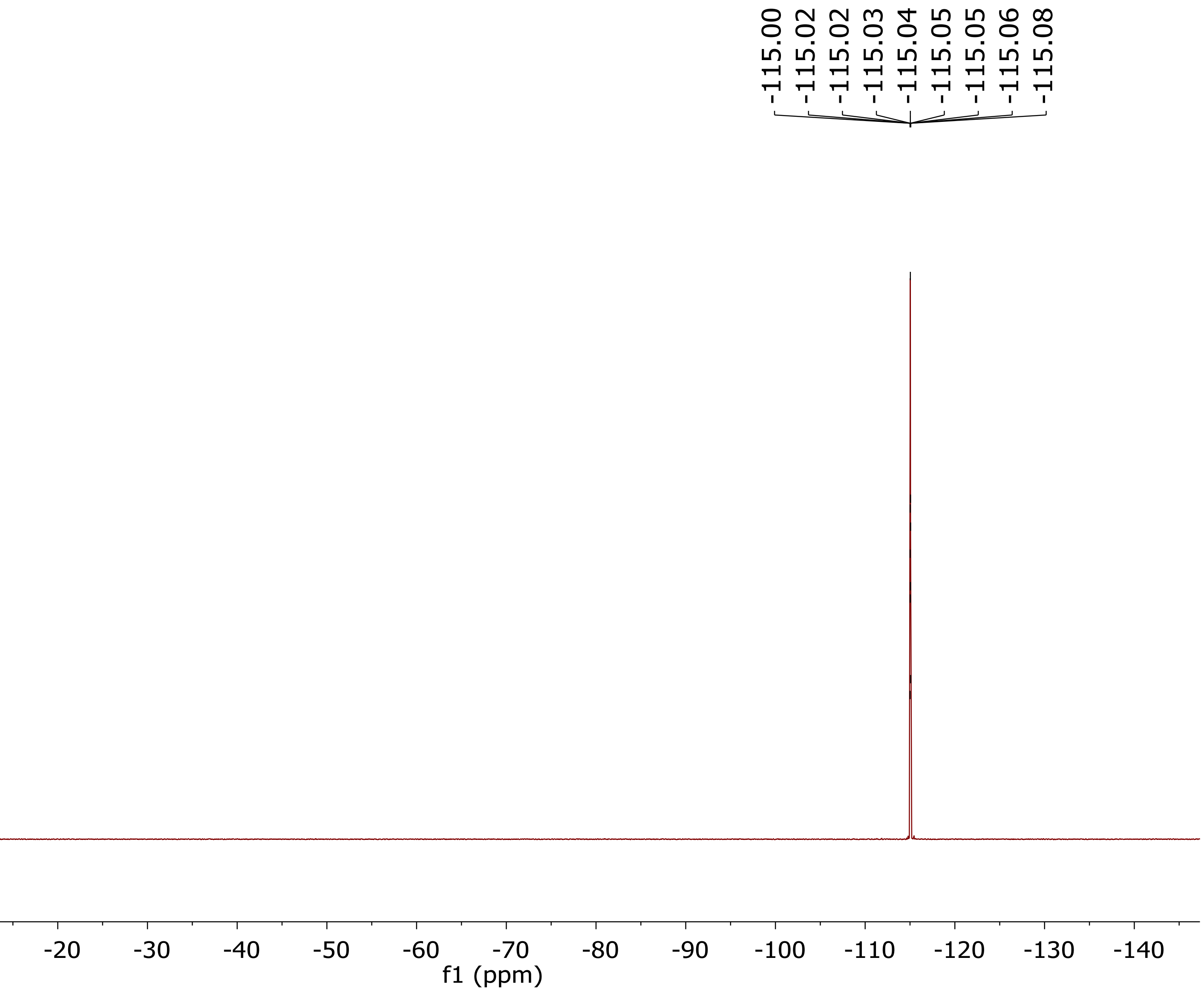

**Ethyl 2,2-bis(4-fluorophenyl)-2-(phenethylamino)acetate (18) - $^{1}H$ NMR (400 MHz, $CDCl_3$):**

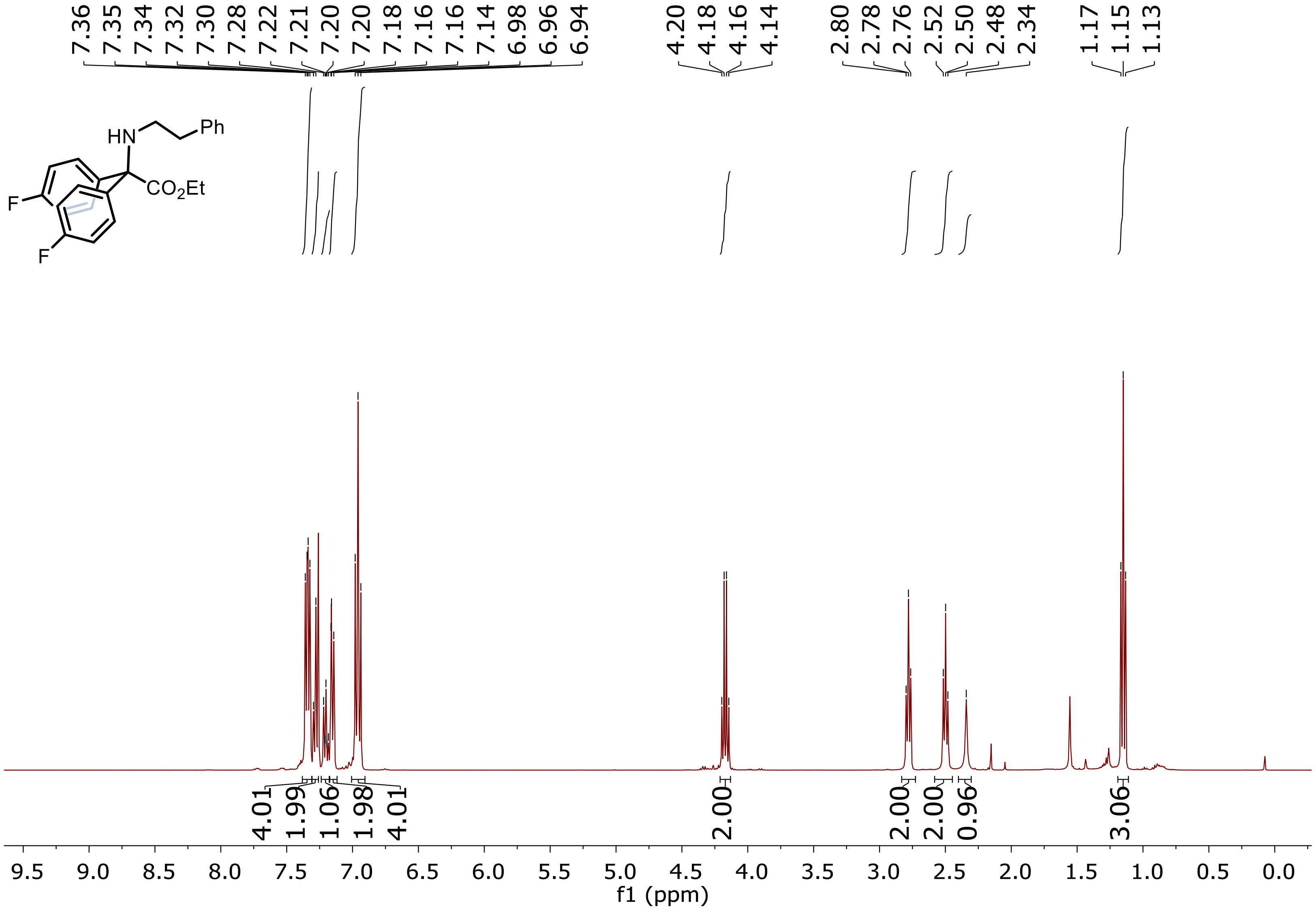

**Ethyl 2,2-bis(4-fluorophenyl)-2-(phenethylamino)acetate (18) - $^{13}C\{^{1}H\}$ NMR (101 MHz, $CDCl_3$):**

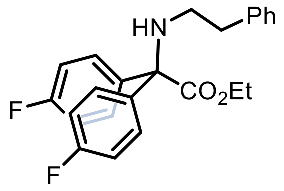


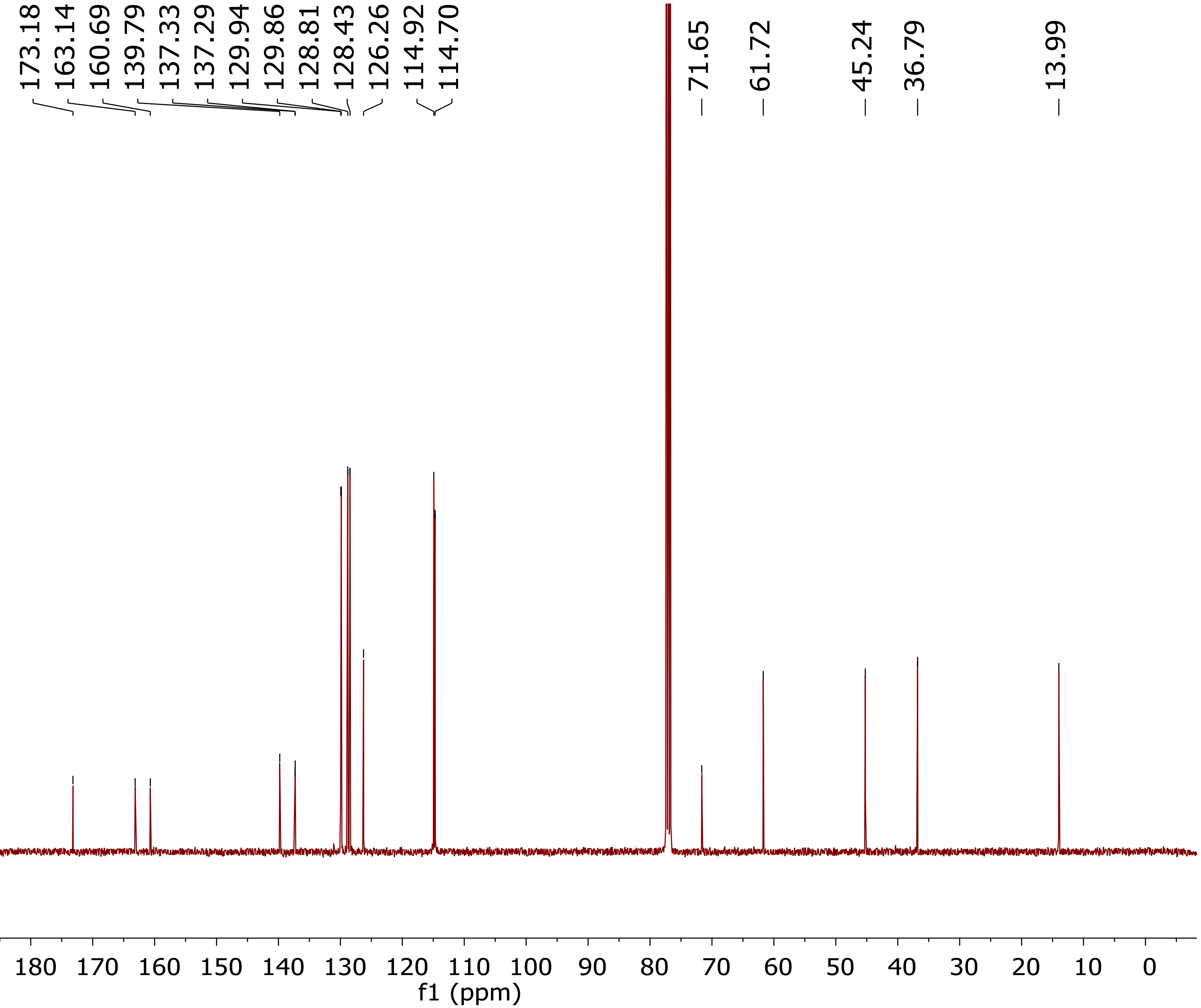

**Ethyl 2,2-bis(4-fluorophenyl)-2-(phenethylamino)acetate (18) - $^{19}$F NMR (376 MHz, $CDCl_3$):**

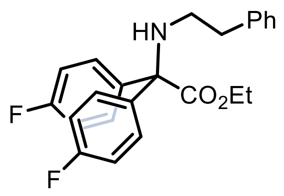


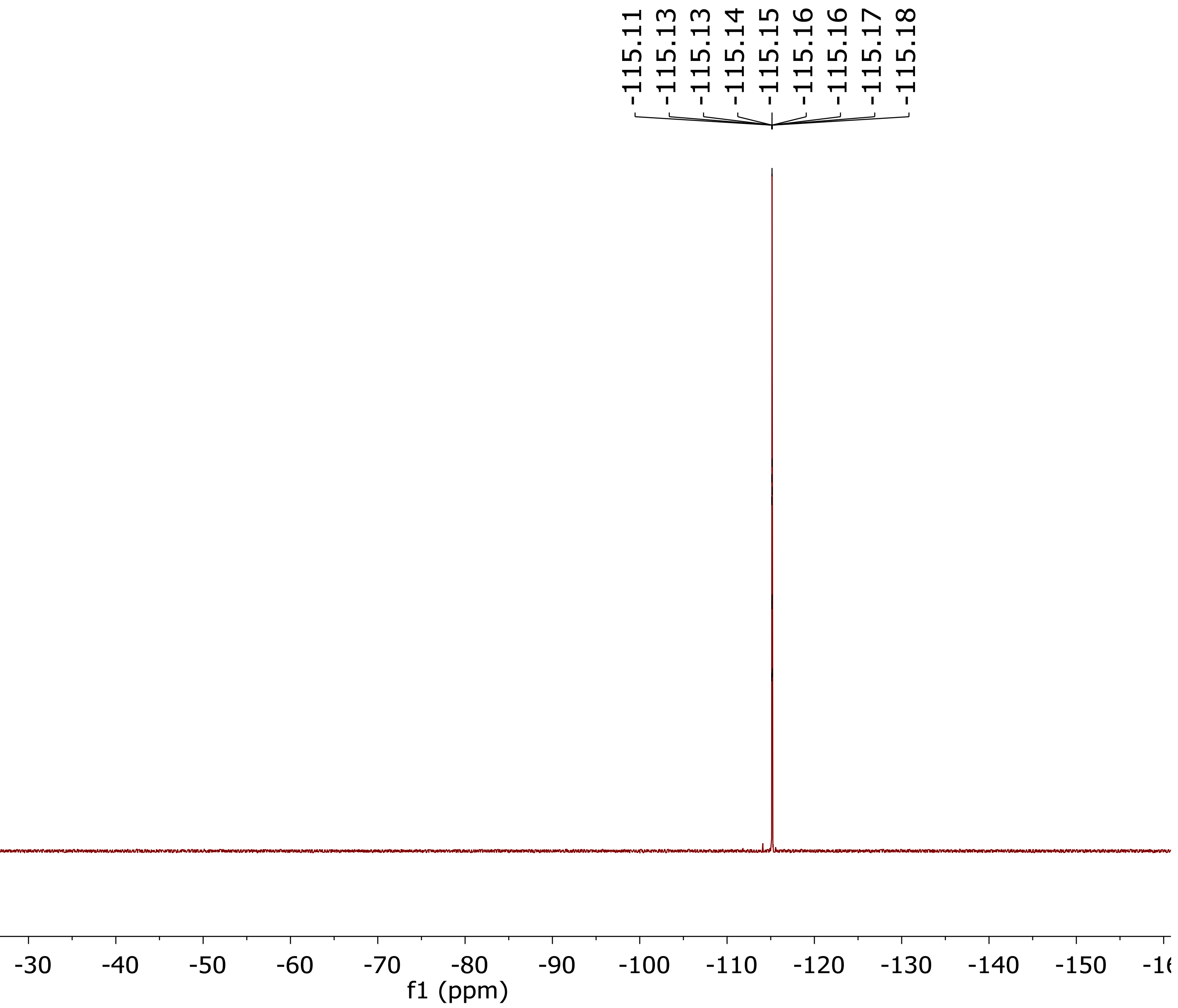

**Ethyl 2,2-bis(4-fluorophenyl)-2-(isobutylamino)acetate (19) - $^{1}$H NMR (400 MHz, $CDCl_3$):**

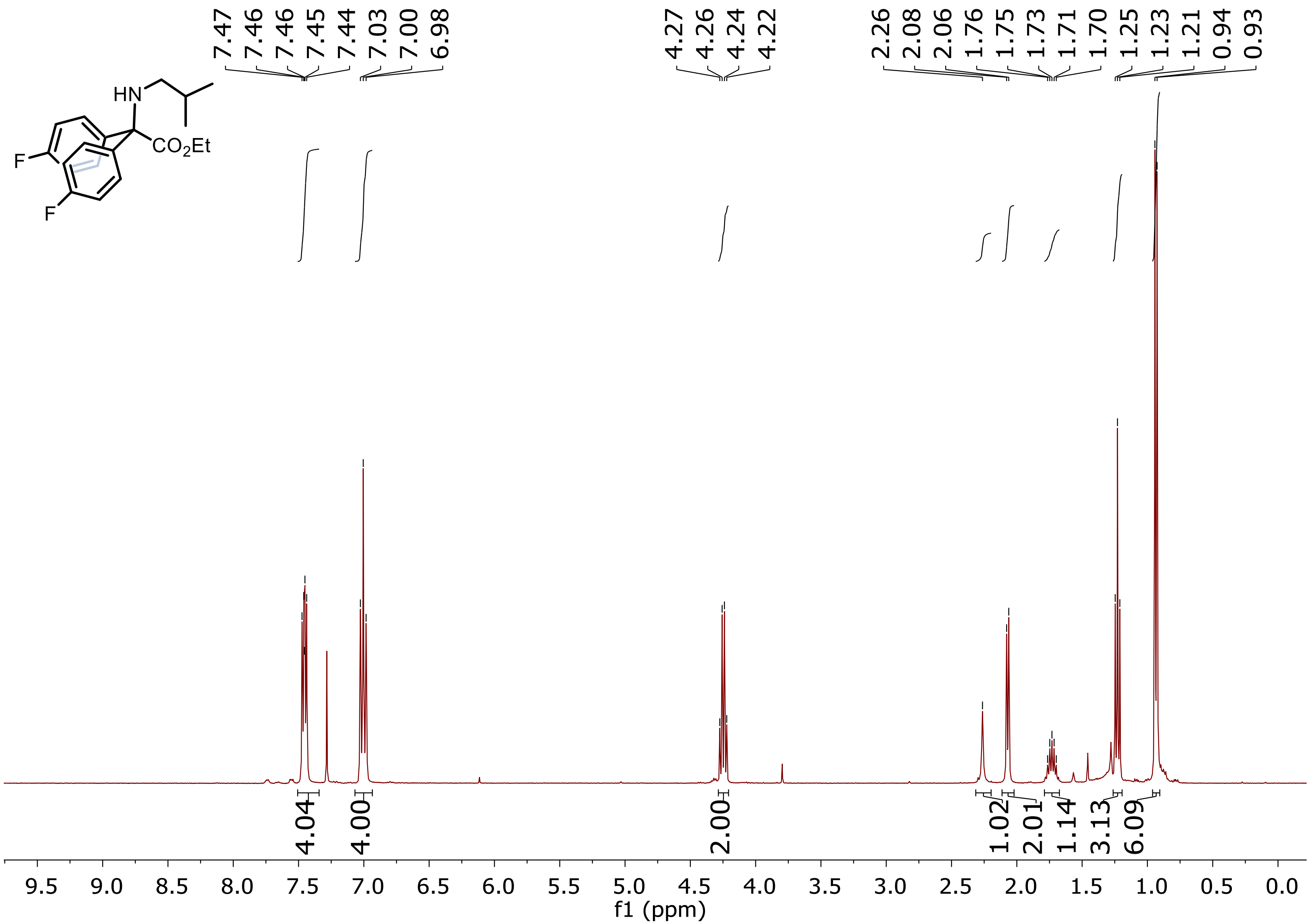

**Ethyl 2,2-bis(4-fluorophenyl)-2-(isobutylamino)acetate (19) - $^{13}C\{^1H\}$ NMR (101 MHz, $CDCl_3$):**

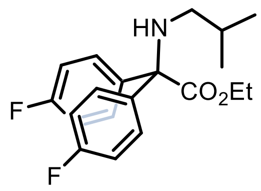


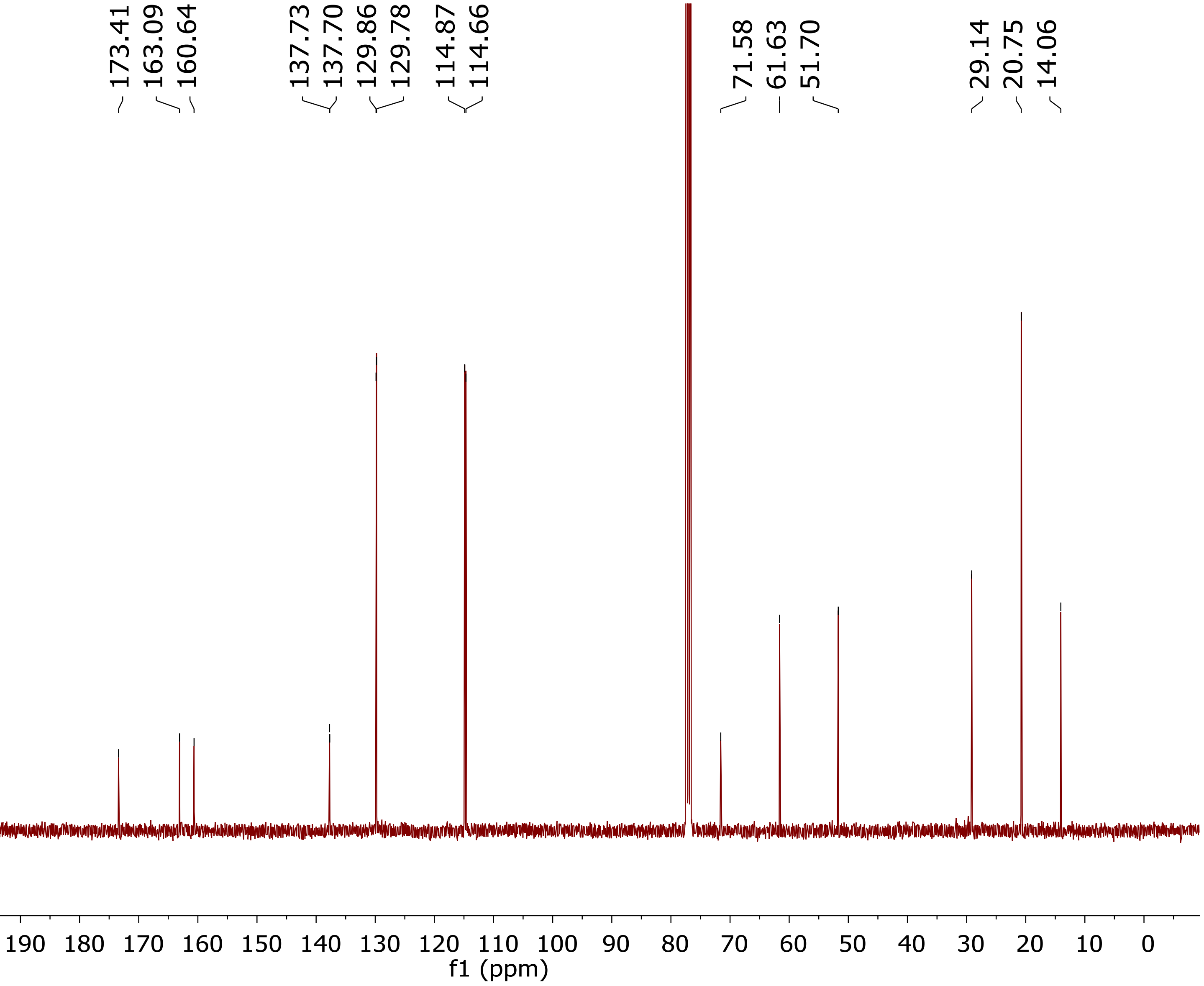

**Ethyl 2,2-bis(4-fluorophenyl)-2-(isobutylamino)acetate (19) - $^{19}$F NMR (376 MHz, $CDCl_3$):**

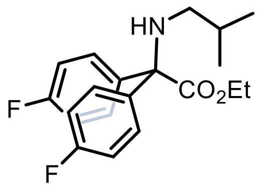


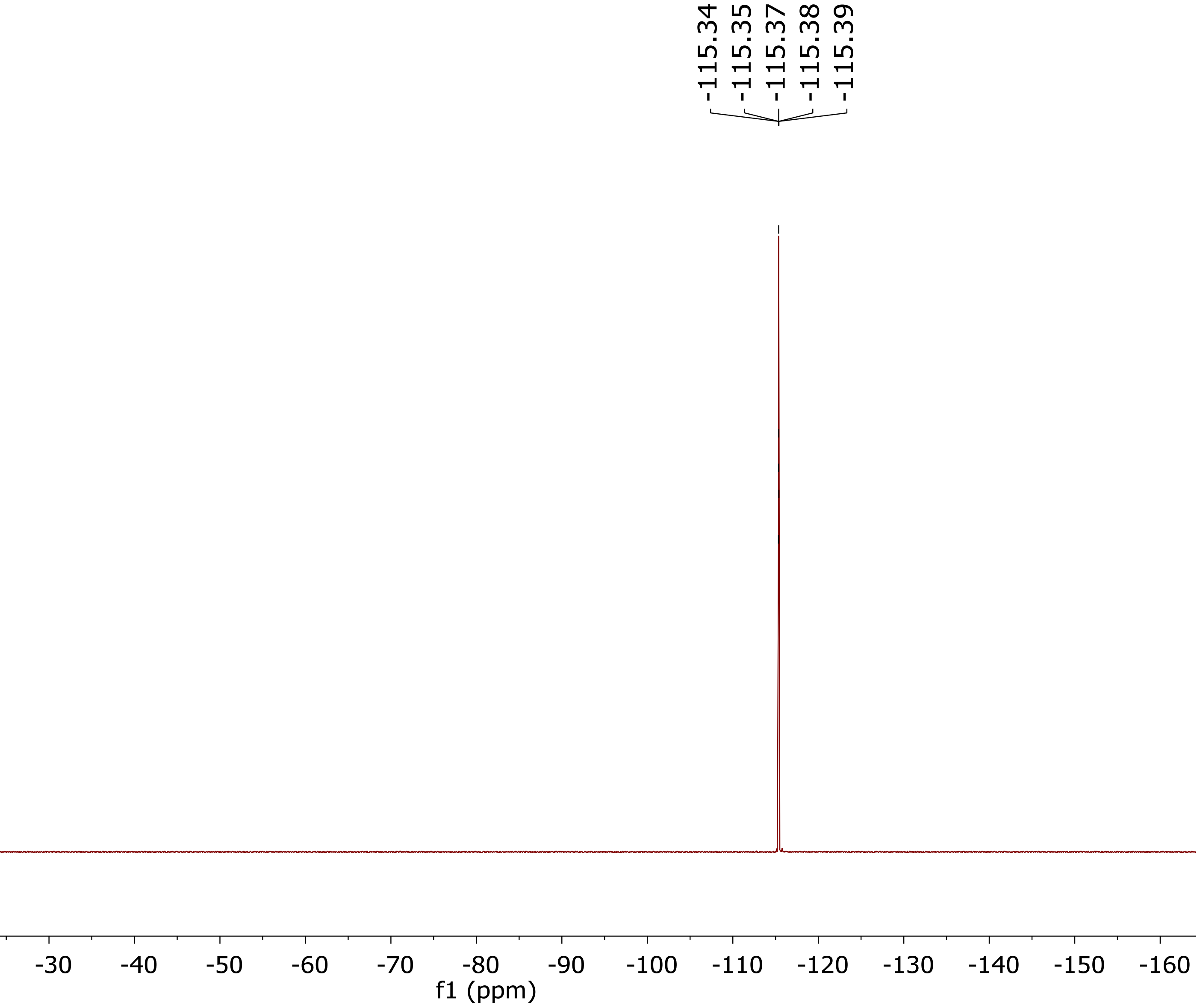

**Ethyl 2,2-bis(4-fluorophenyl)-2-((2-methoxyethyl)amino)acetate (20) - $^1$H NMR (400 MHz, $CDCl_3$):**

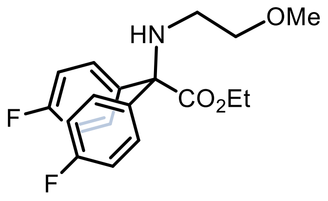


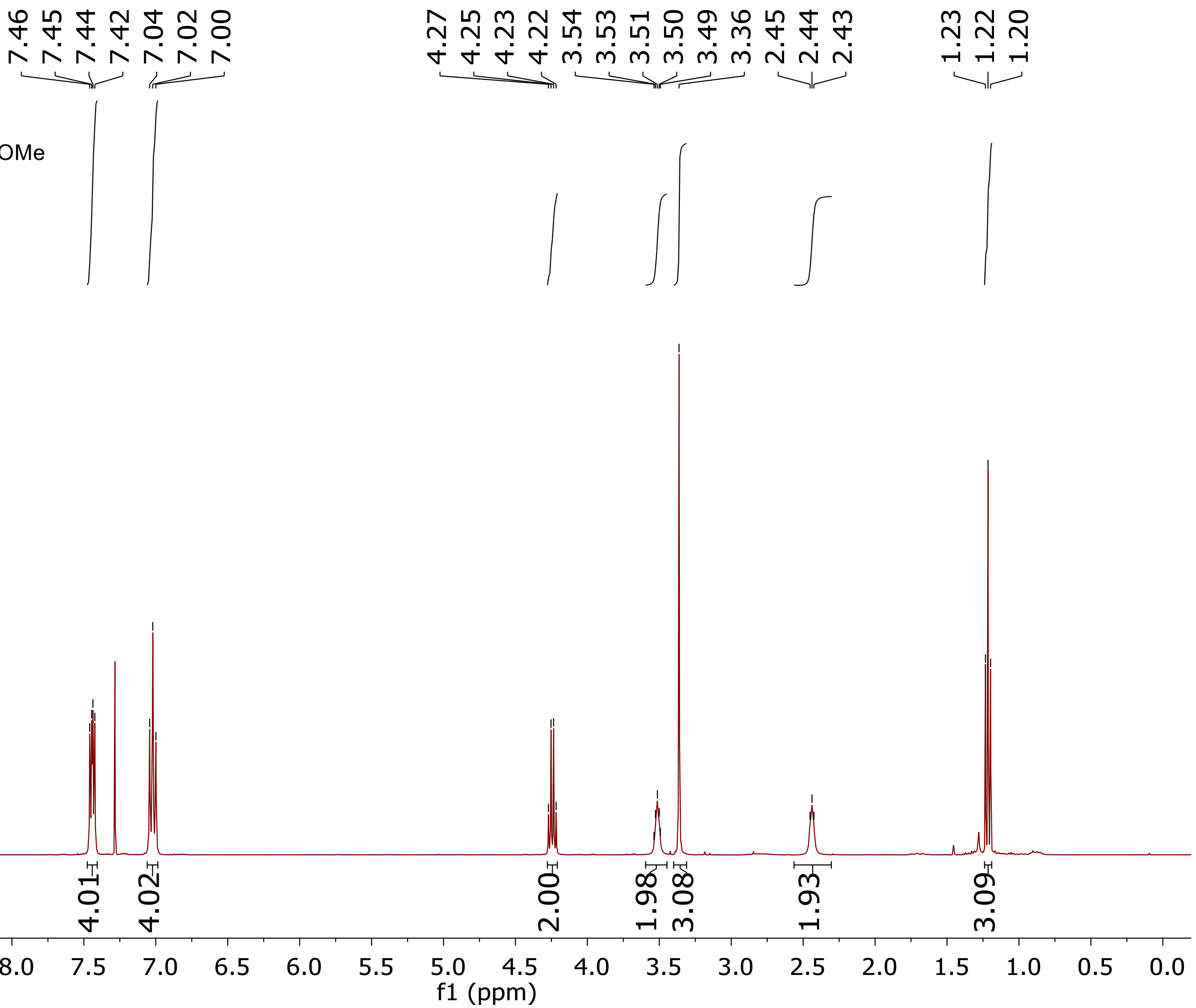

**Ethyl 2,2-bis(4-fluorophenyl)-2-((2-methoxyethyl)amino)acetate (20) - $^{13}C\{^1H\}$ NMR (101 MHz, $CDCl_3$):**

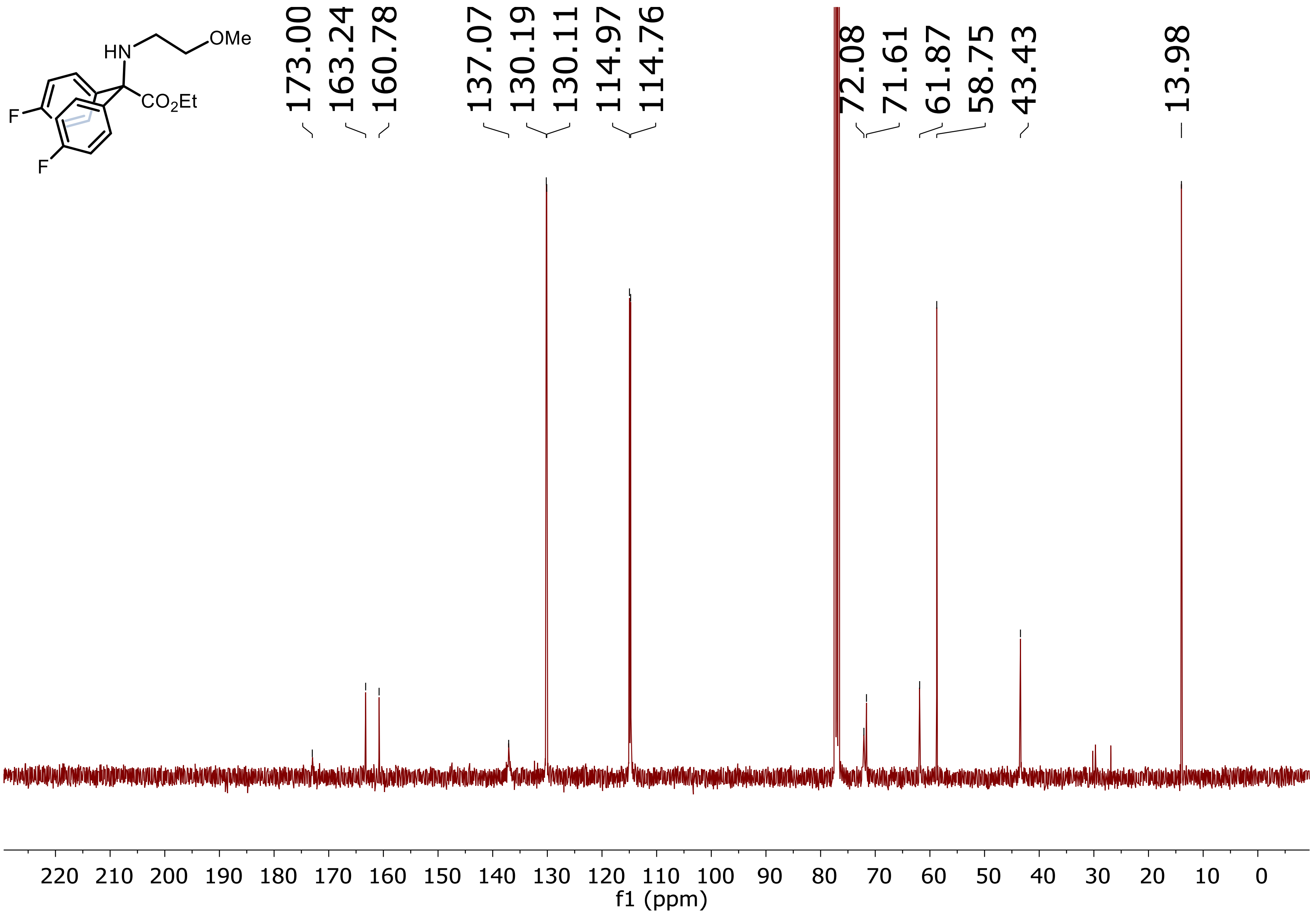

**Ethyl 2,2-bis(4-fluorophenyl)-2-((2-methoxyethyl)amino)acetate (20) - $^{19}$F NMR (376 MHz, $CDCl_3$):**

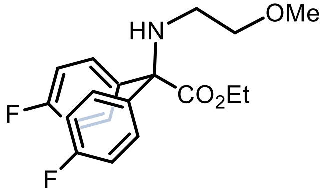


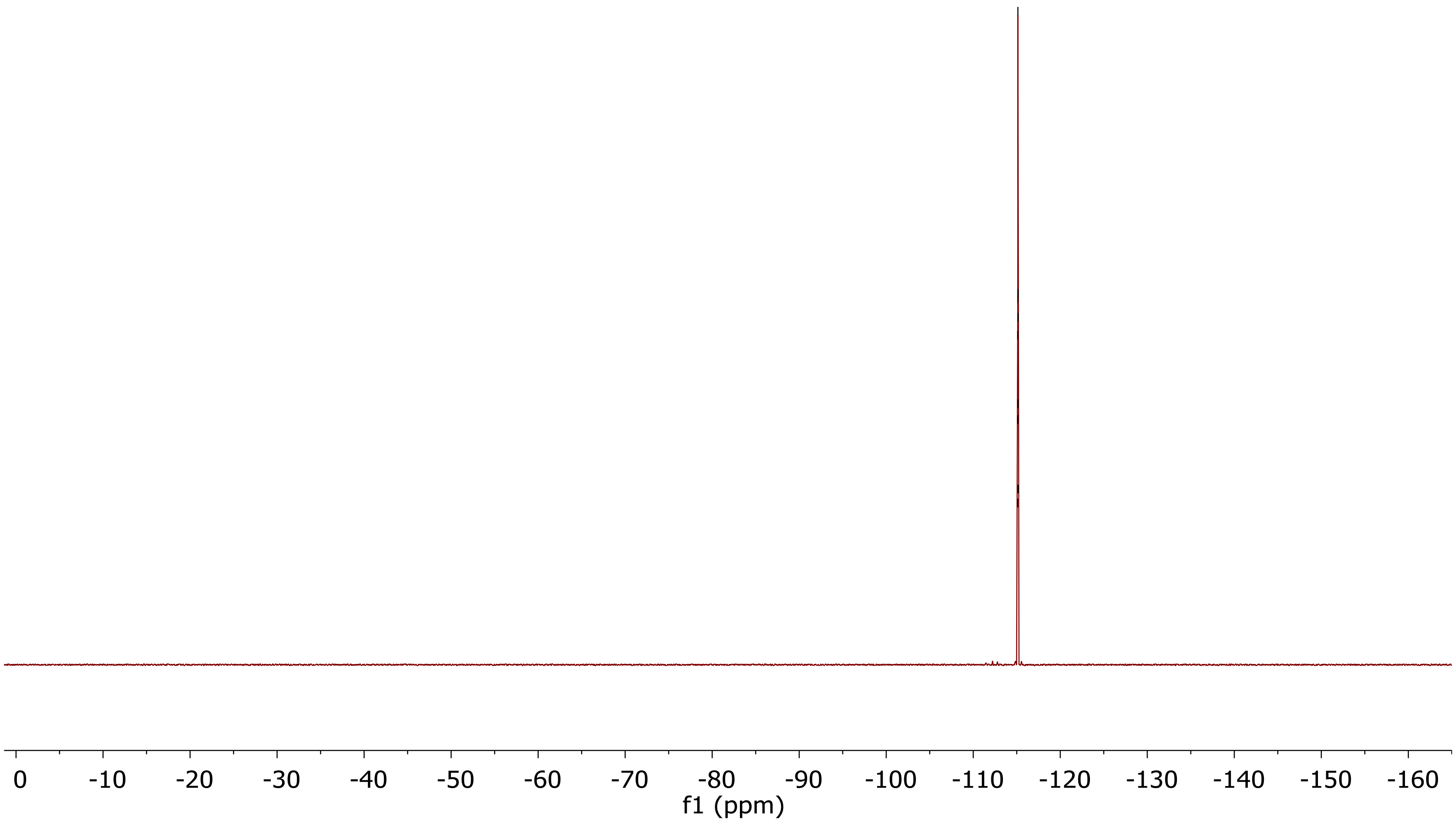

**Ethyl 2-(benzylamino)-2,2-bis(4-fluorophenyl) acetate (21) - $^1$H NMR (400 MHz, CDCl$_3$):**

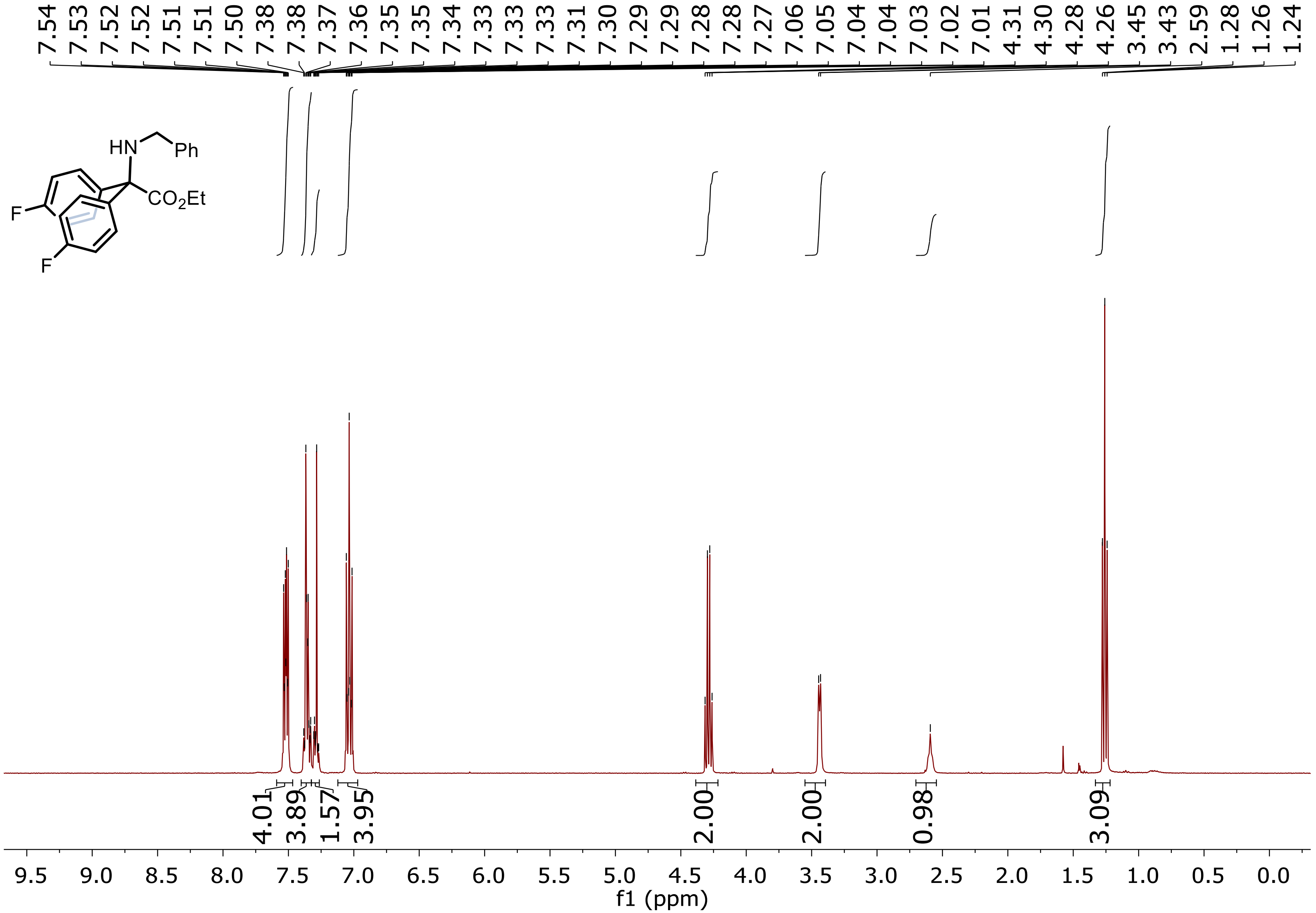

**Ethyl 2-(benzylamino)-2,2-bis(4-fluorophenyl) acetate (21) - $^{13}C\{^{1}H\}$ NMR (101 MHz, $CDCl_3$):**

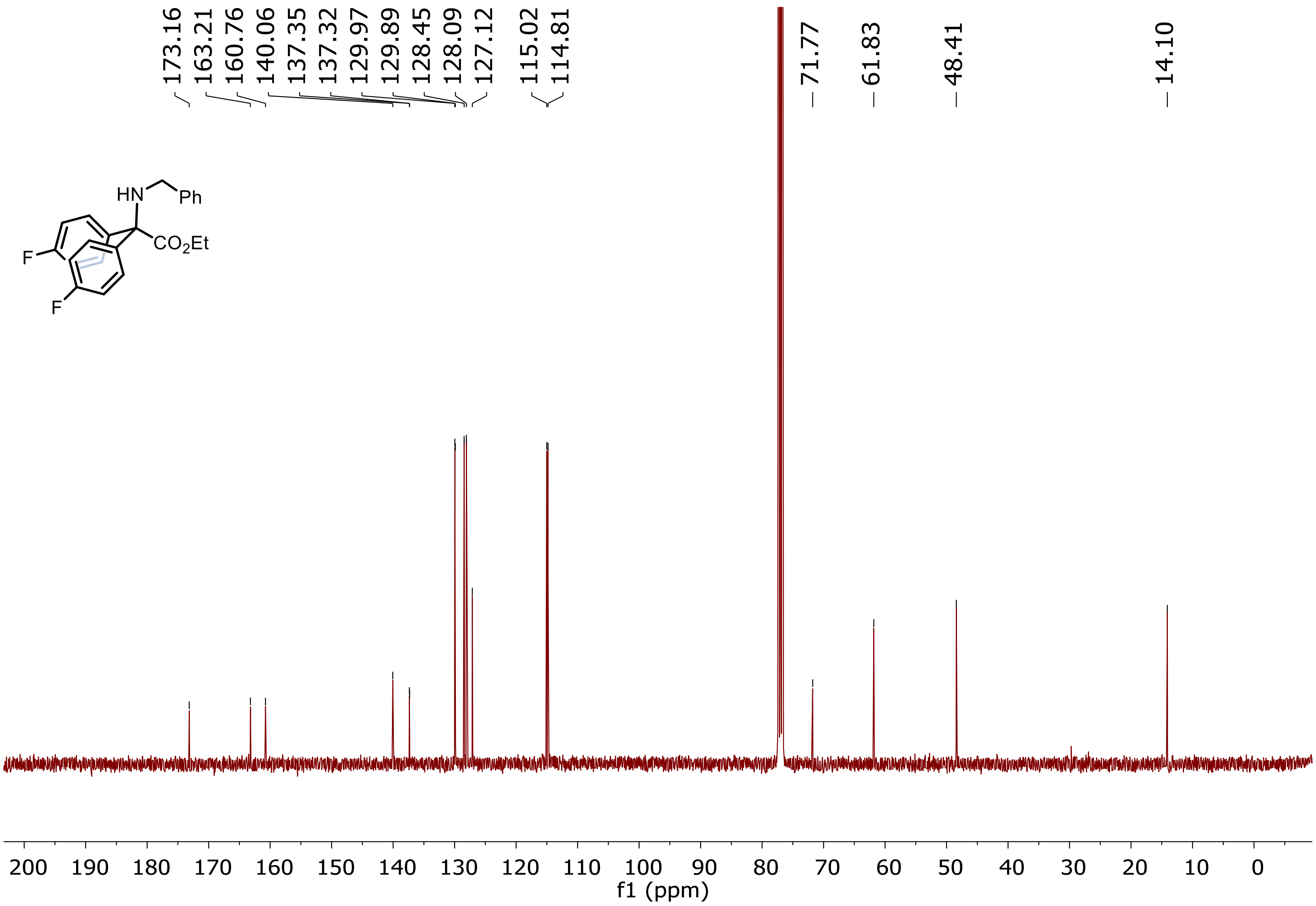

**Ethyl 2-(benzylamino)-2,2-bis(4-fluorophenyl) acetate (21) - $^{19}$F NMR (376 MHz, $CDCl_3$):**

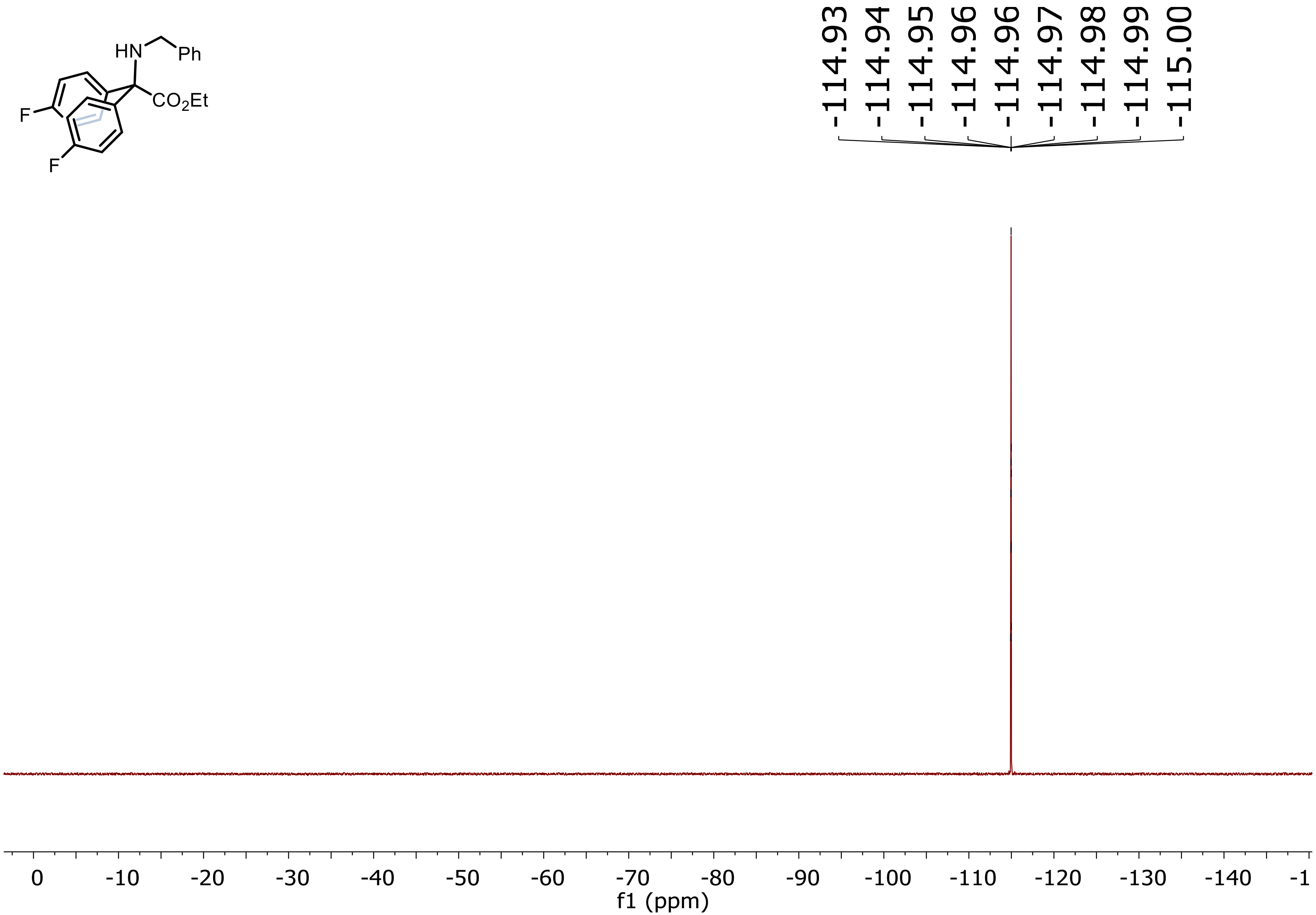

**Ethyl 2,2-bis(4-fluorophenyl)-2-((thiophen-2-ylmethyl)amino)acetate (22) - $^{1}$H NMR (400 MHz, $CDCl_3$):**

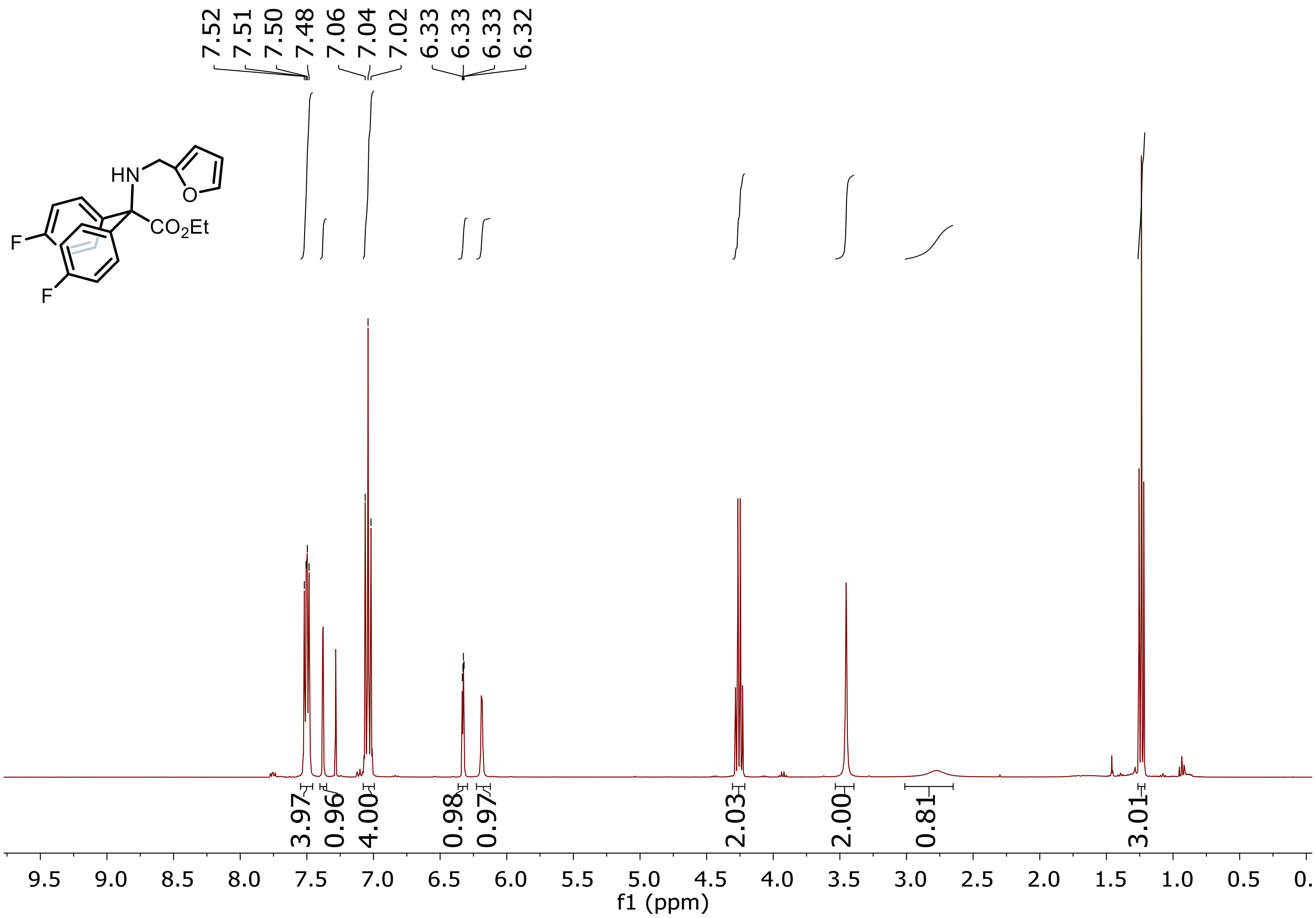

**Ethyl 2,2-bis(4-fluorophenyl)-2-((thiophen-2-ylmethyl)amino)acetate (22) - $^{13}C\{^1H\}$ NMR (101 MHz, $CDCl_3$):**

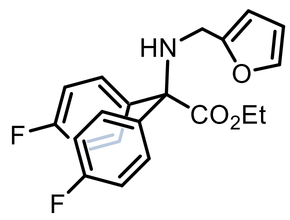


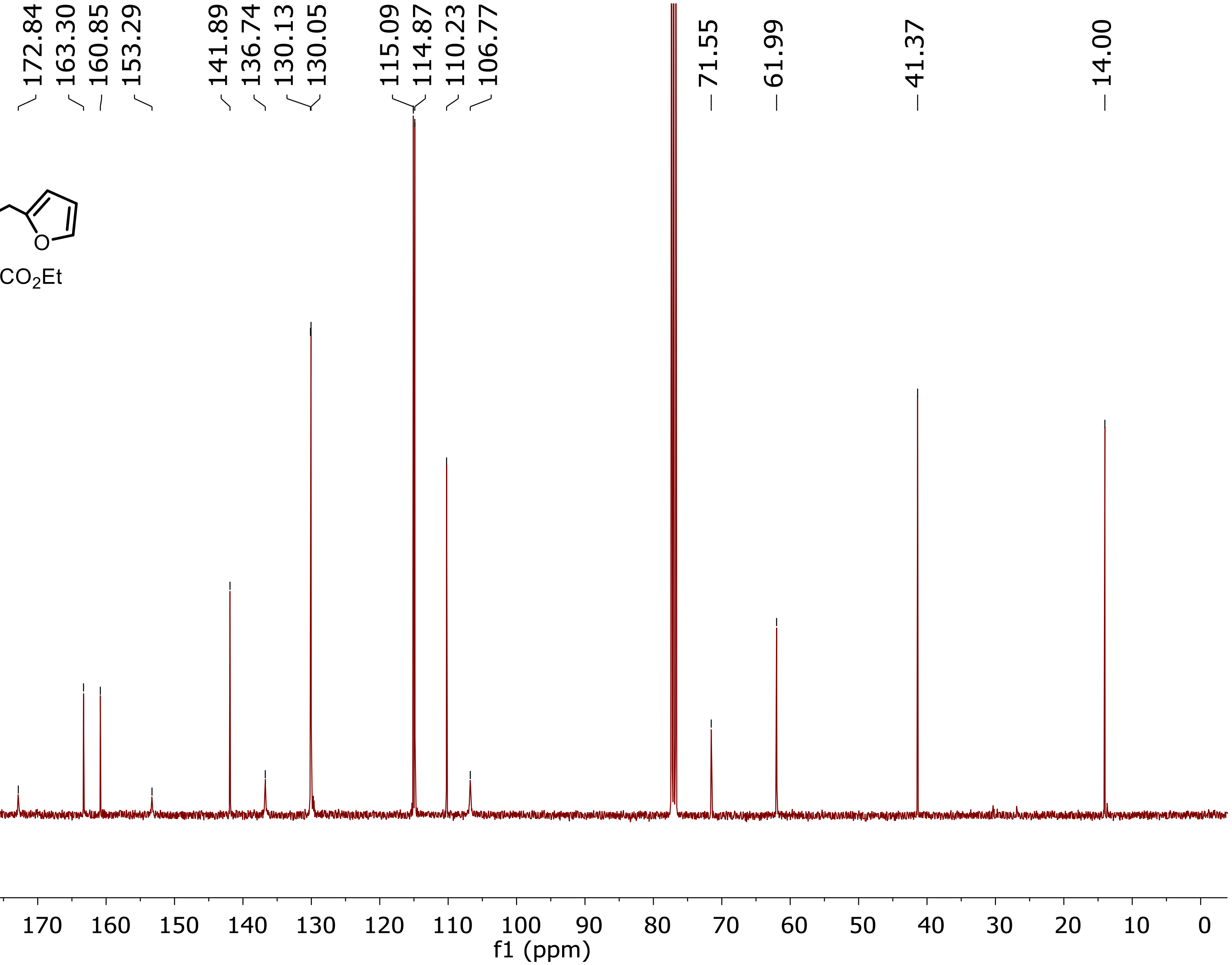

**Ethyl 2,2-bis(4-fluorophenyl)-2-((thiophen-2-ylmethyl)amino)acetate (22) -$^{19}$F NMR (376 MHz, CDCl$_3$):**

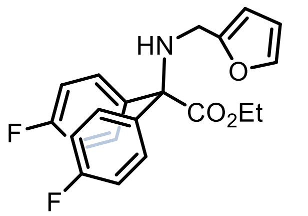


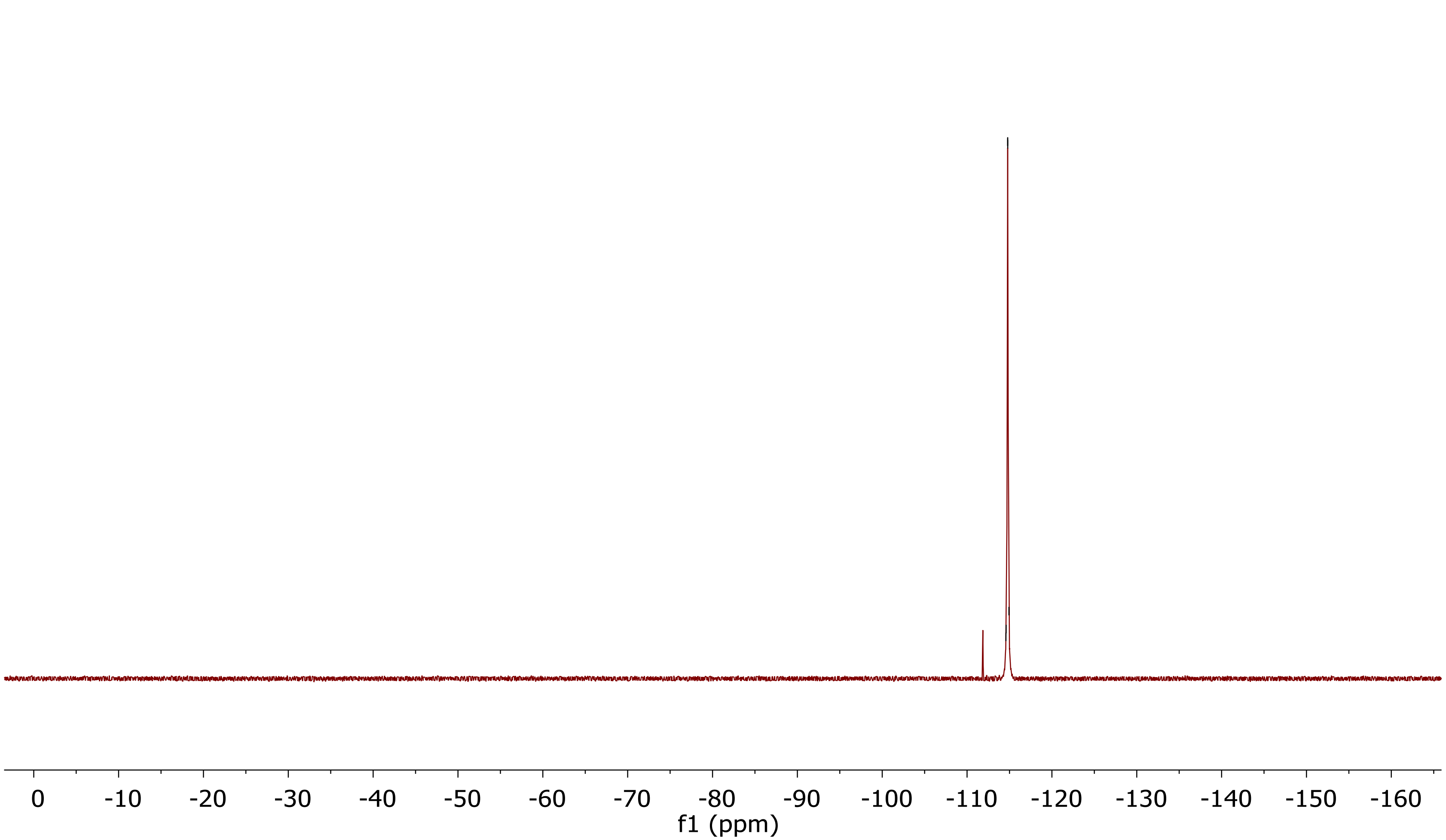

**Ethyl 2,2-bis(4-fluorophenyl)-2-((thiophen-2-ylmethyl)amino)acetate (23) - $^{1}$H NMR (400 MHz, $CDCl_3$):**

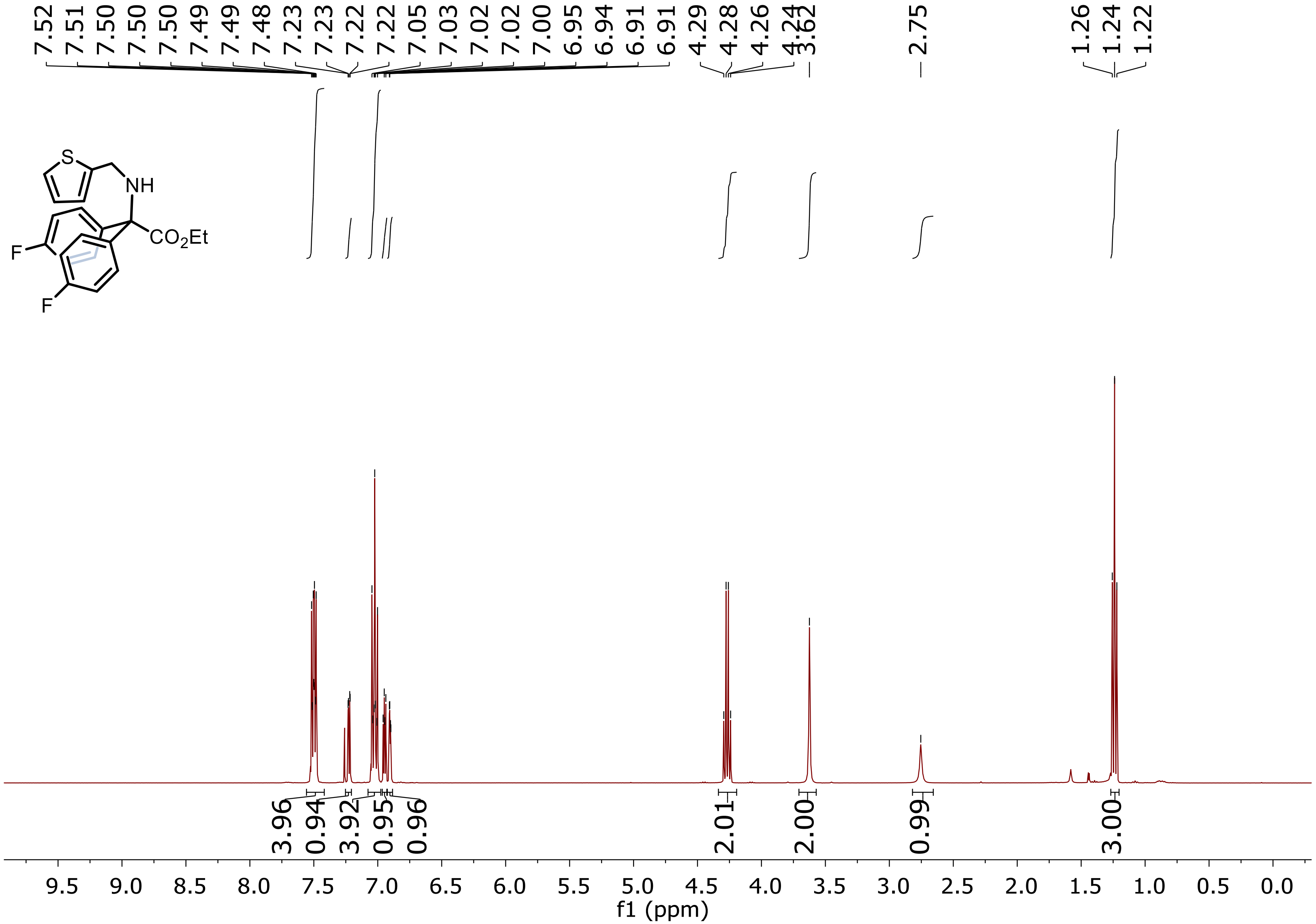

**Ethyl 2,2-bis(4-fluorophenyl)-2-((thiophen-2-ylmethyl)amino)acetate (23) - $^{13}C\{^{1}H\}$ NMR (101 MHz, $CDCl_3$):**

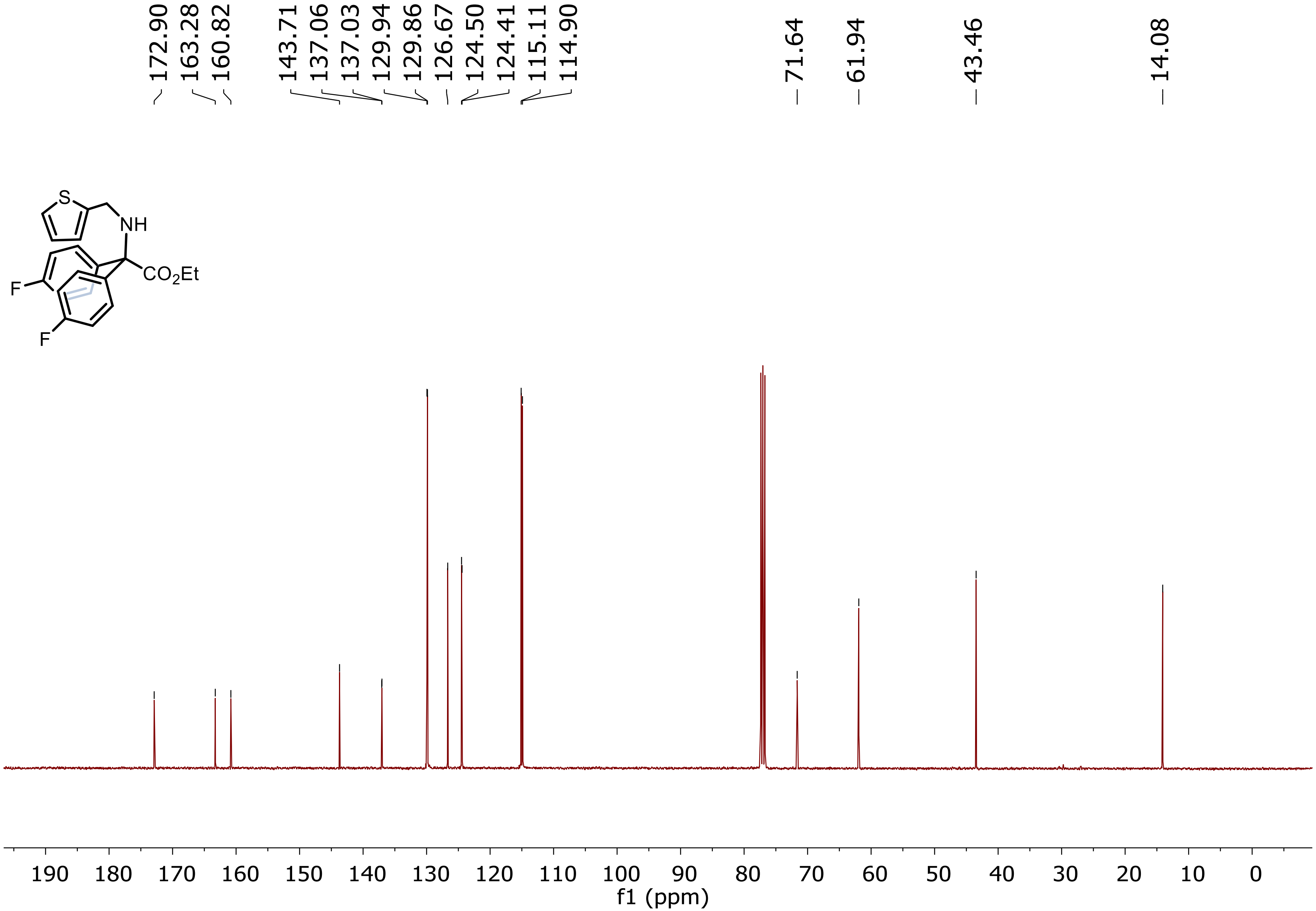

**Ethyl 2,2-bis(4-fluorophenyl)-2-((thiophen-2-ylmethyl)amino)acetate (23) - $^{19}$F NMR (376 MHz, $CDCl_3$):**

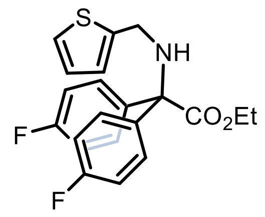


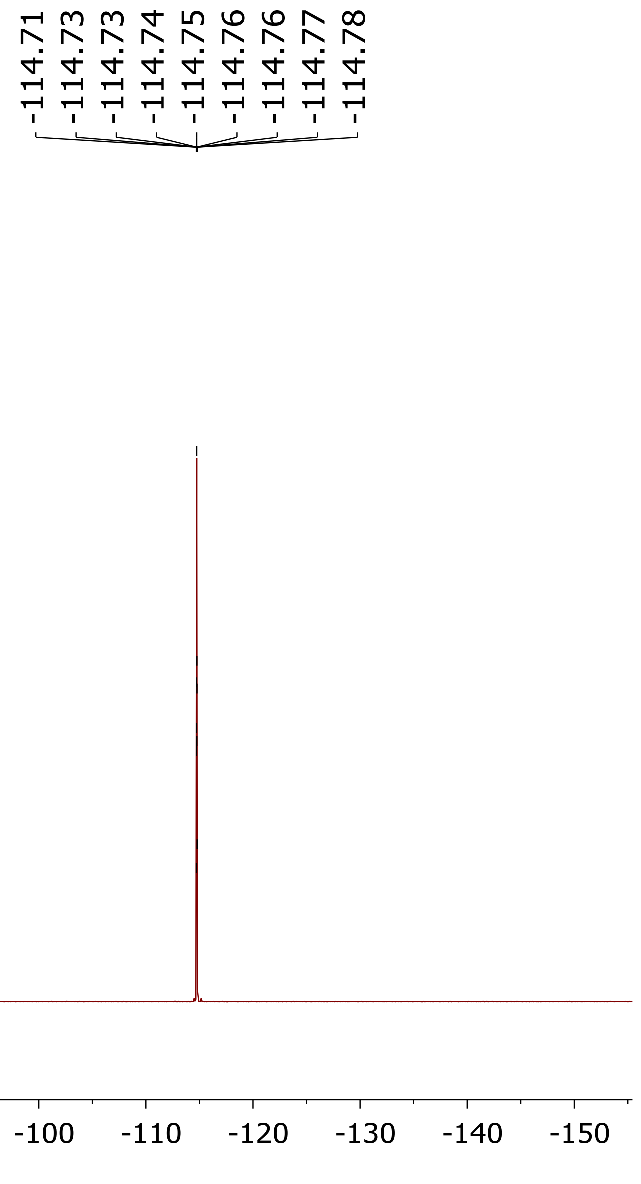

**Ethyl 2,2-bis(4-fluorophenyl)-2-((pyridin-2-ylmethyl)amino)acetate (24) - $^{1}$H NMR (400 MHz, $CDCl_3$):**

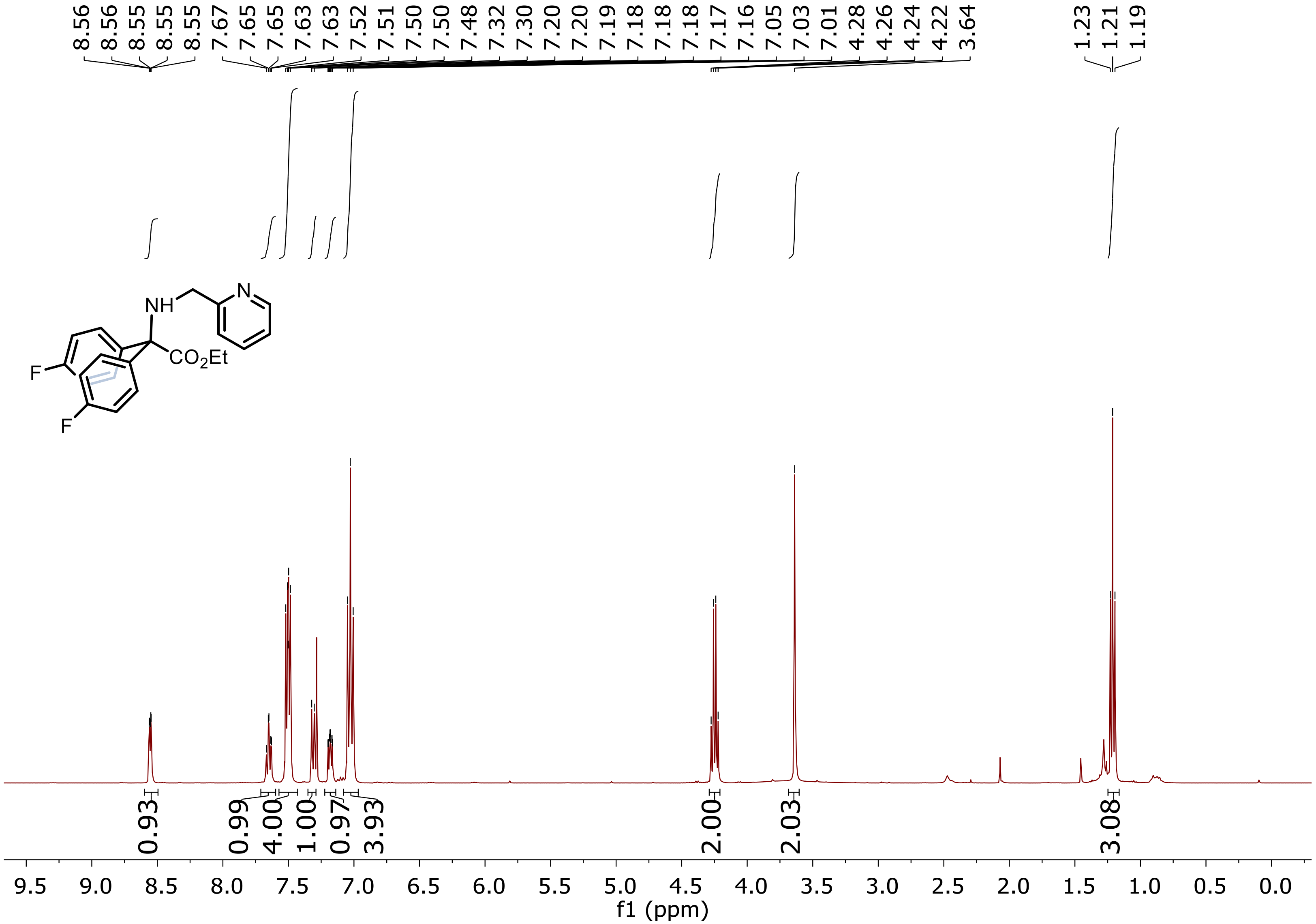

**Ethyl 2,2-bis(4-fluorophenyl)-2-((pyridin-2-ylmethyl)amino)acetate (24) - $^{13}C\{^{1}H\}$ NMR (101 MHz, $CDCl_3$):**

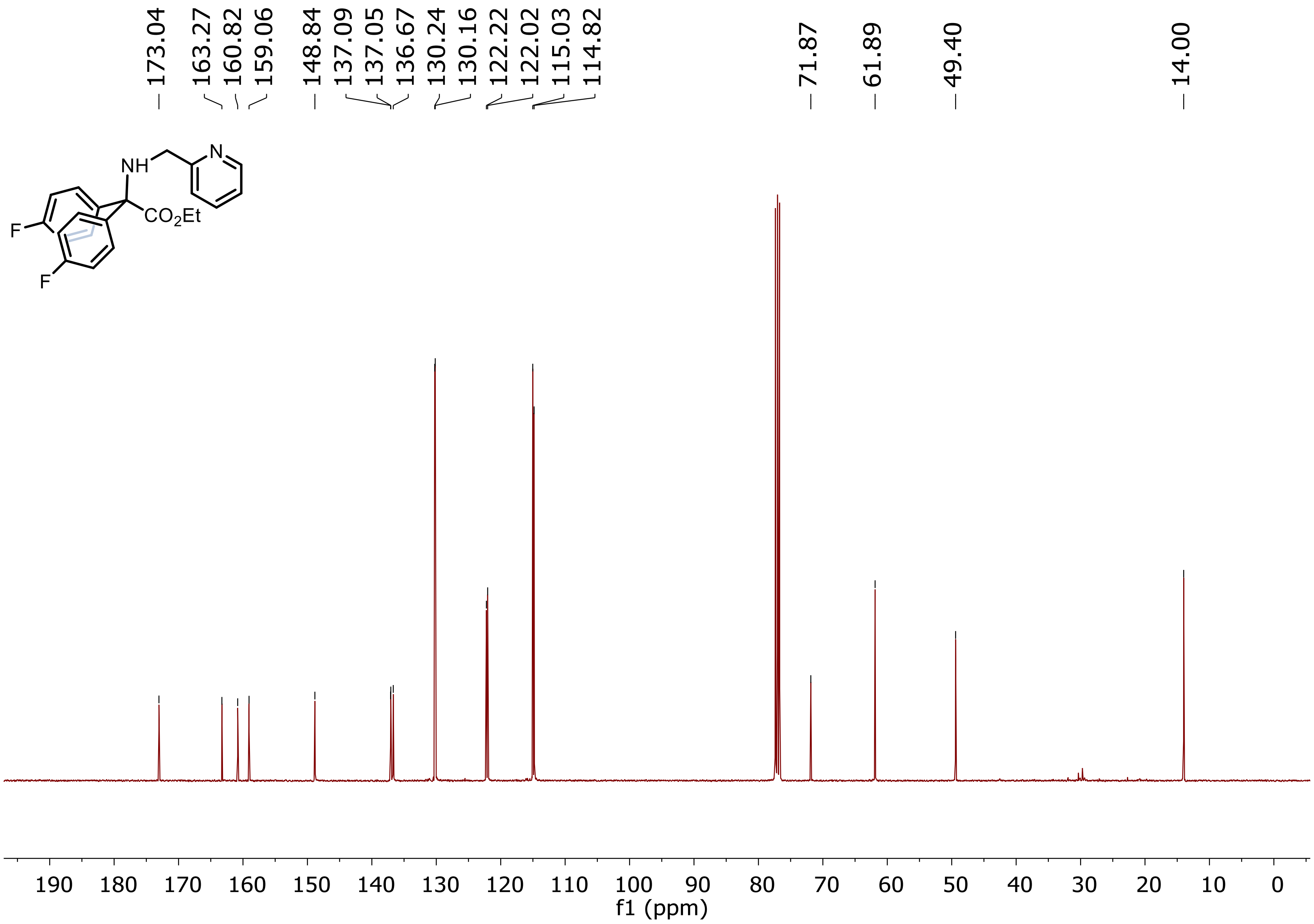

**Ethyl 2,2-bis(4-fluorophenyl)-2-((pyridin-2-ylmethyl)amino)acetate (24) - $^{19}$F NMR (376 MHz, $CDCl_3$):**

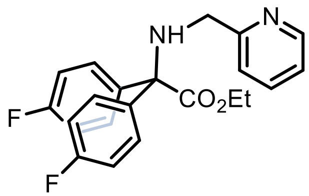


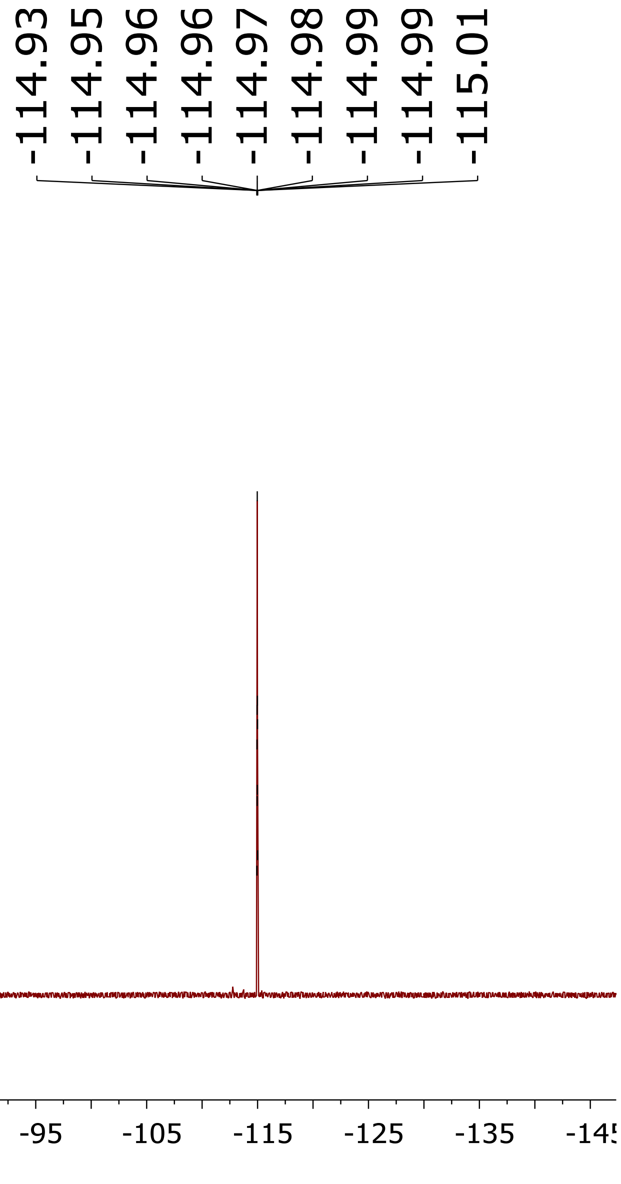

**Ethyl 2-(cyclohexylamino)-2,2-bis(4-fluorophenyl) acetate (25) - $^{1}$H NMR (400 MHz, $CDCl_3$):**

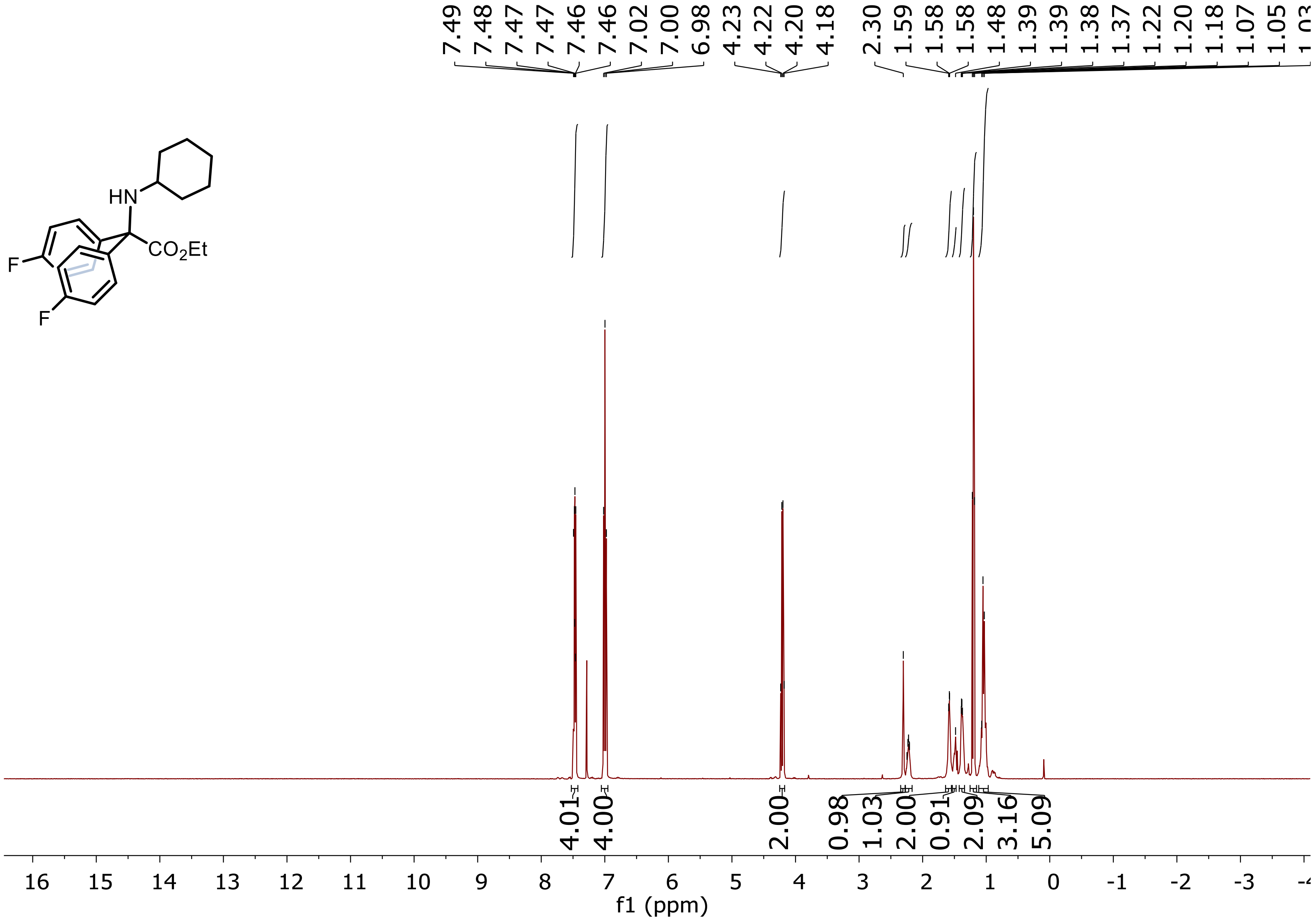

**Ethyl 2-(cyclohexylamino)-2,2-bis(4-fluorophenyl) acetate (25) - $^{13}C\{^{1}H\}$ NMR (101 MHz, $CDCl_3$):**

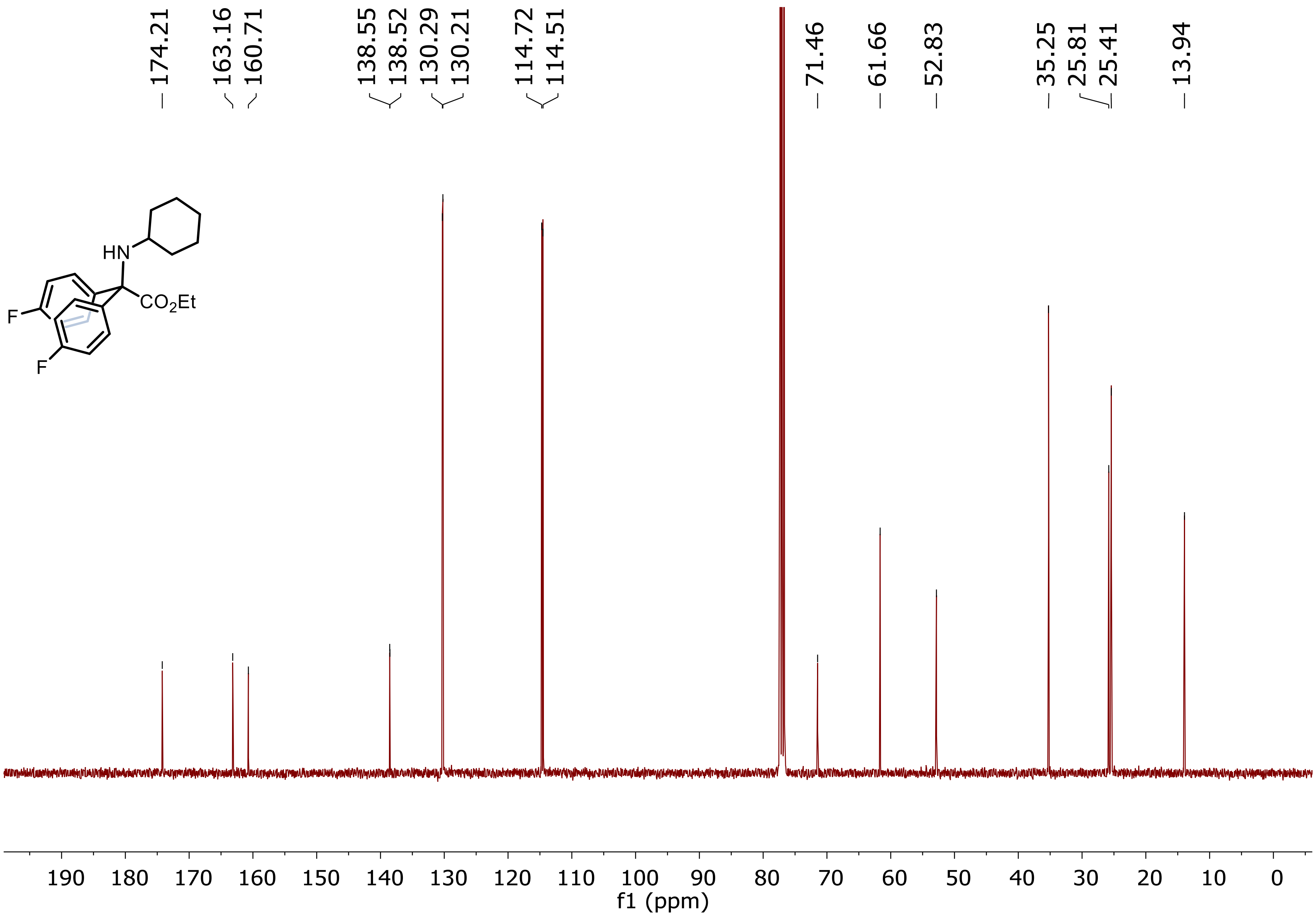

**Ethyl 2-(cyclohexylamino)-2,2-bis(4-fluorophenyl) acetate (25) - $^{19}$F NMR (376 MHz, $CDCl_3$):**

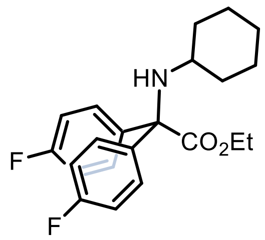

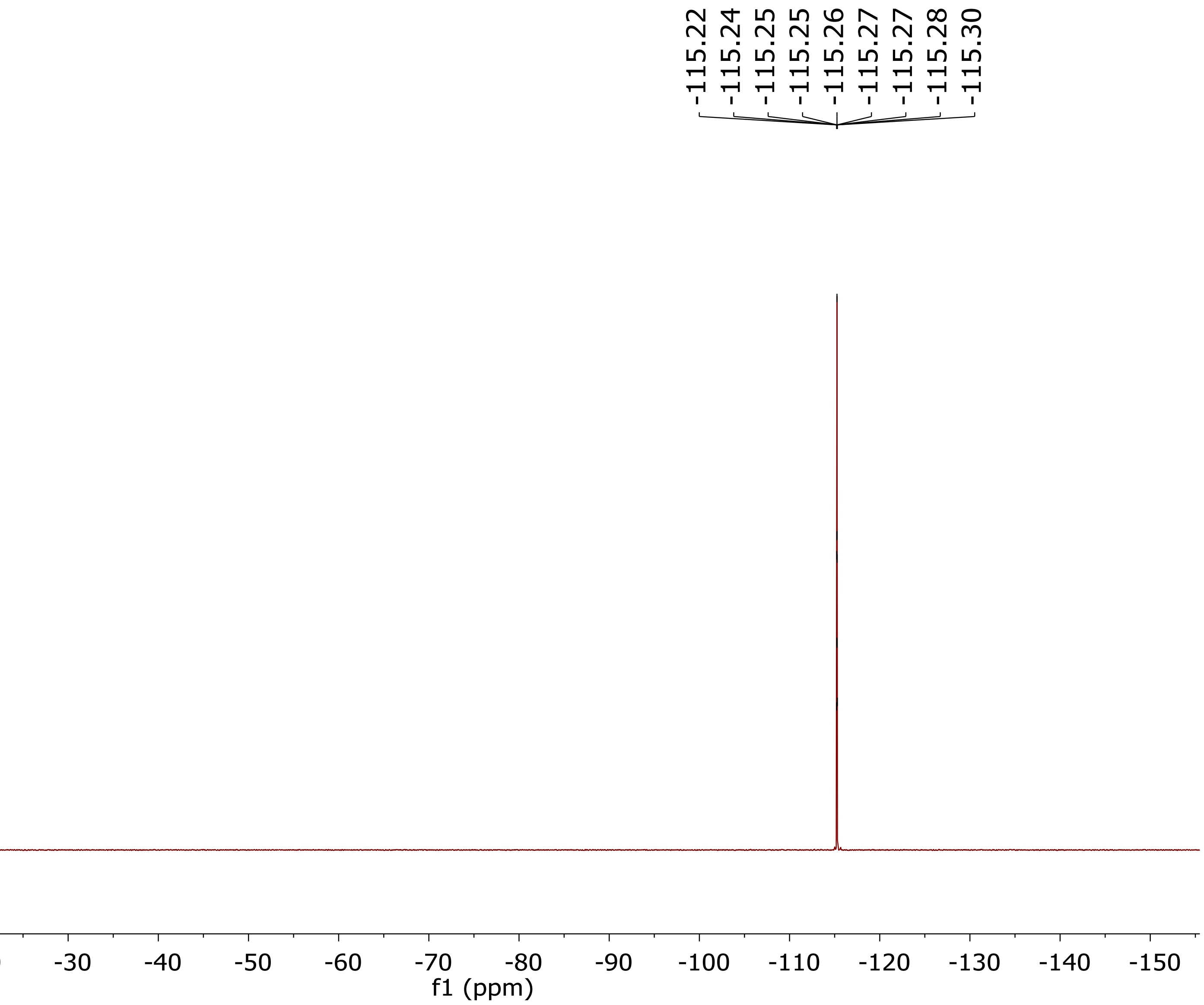

**Ethyl 2-((1-(2,6-dimethylphenoxy)propan-2-yl)amino)-2,2-bis(4-fluorophenyl)acetate (26) - $^1$H NMR (400 MHz, $CDCl_3$):**

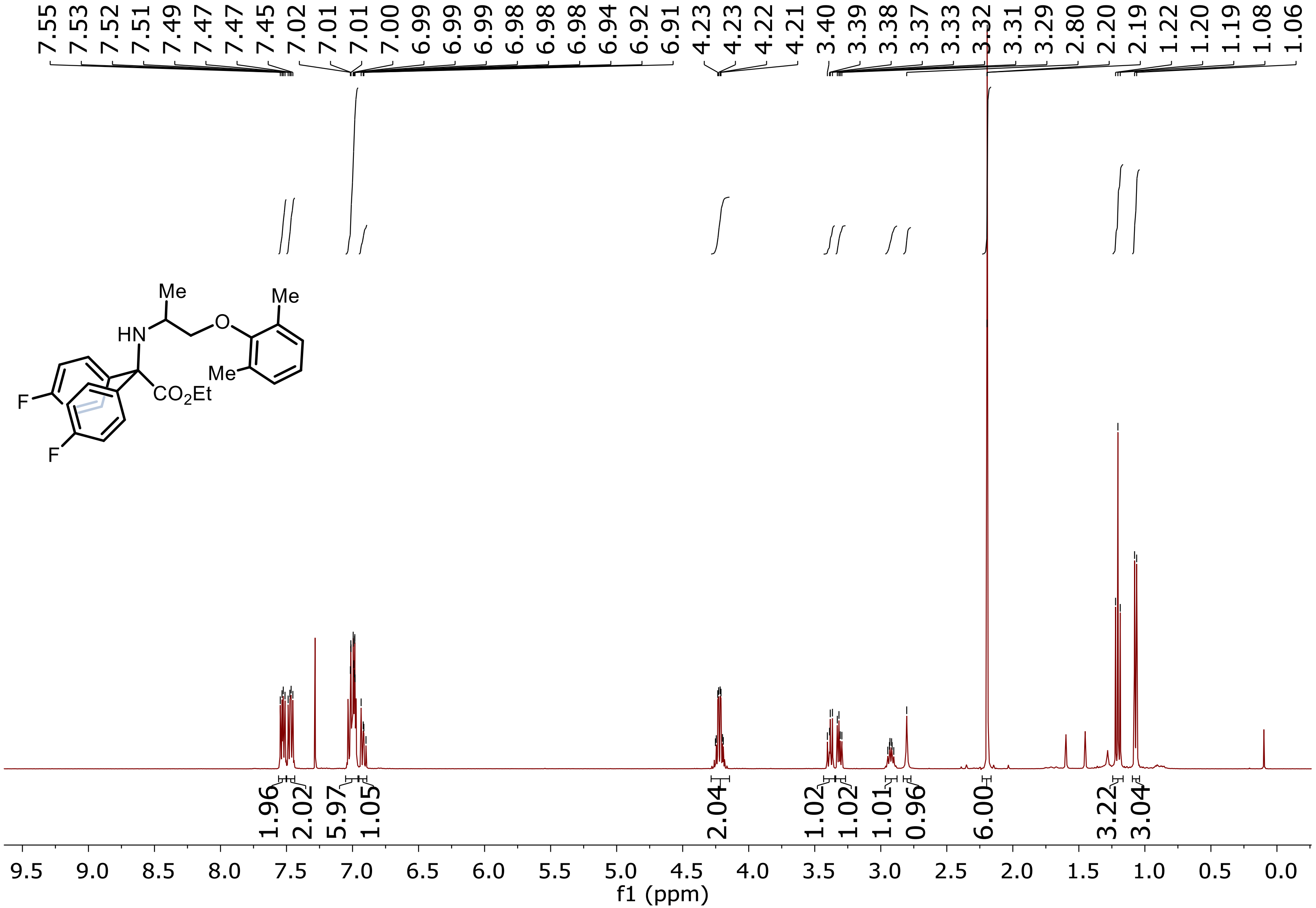

**Ethyl 2-((1-(2,6-dimethylphenoxy)propan-2-yl)amino)-2,2-bis(4-fluorophenyl)acetate (26) - $^{13}C\{^1H\}$ NMR (101 MHz, $CDCl_3$):**

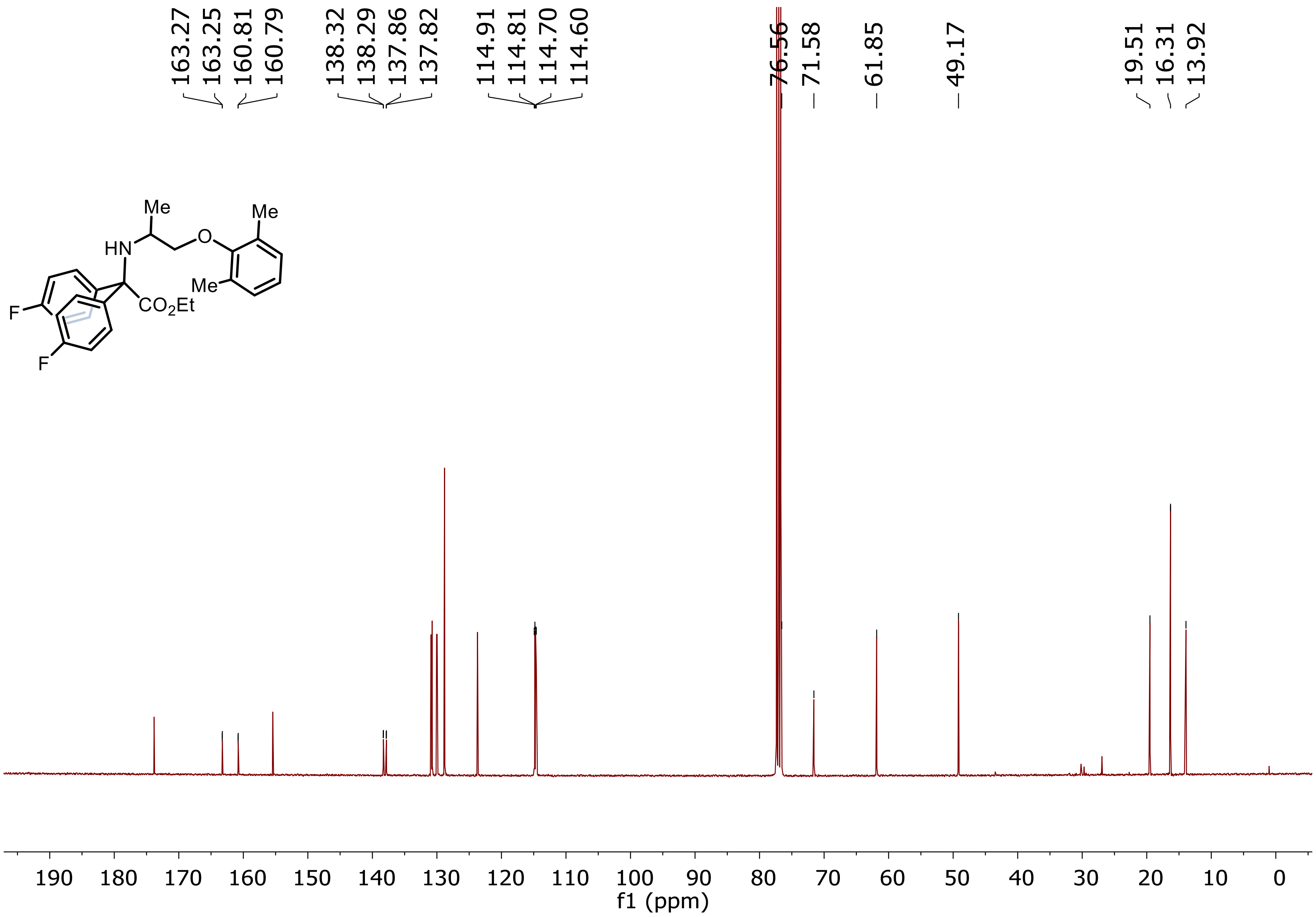

**Ethyl 2-((1-(2,6-dimethylphenoxy)propan-2-yl)amino)-2,2-bis(4-fluorophenyl)acetate (26) - $^{19}$F NMR (376 MHz, $CDCl_3$):**

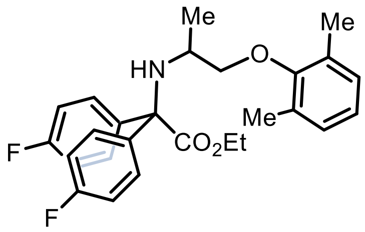


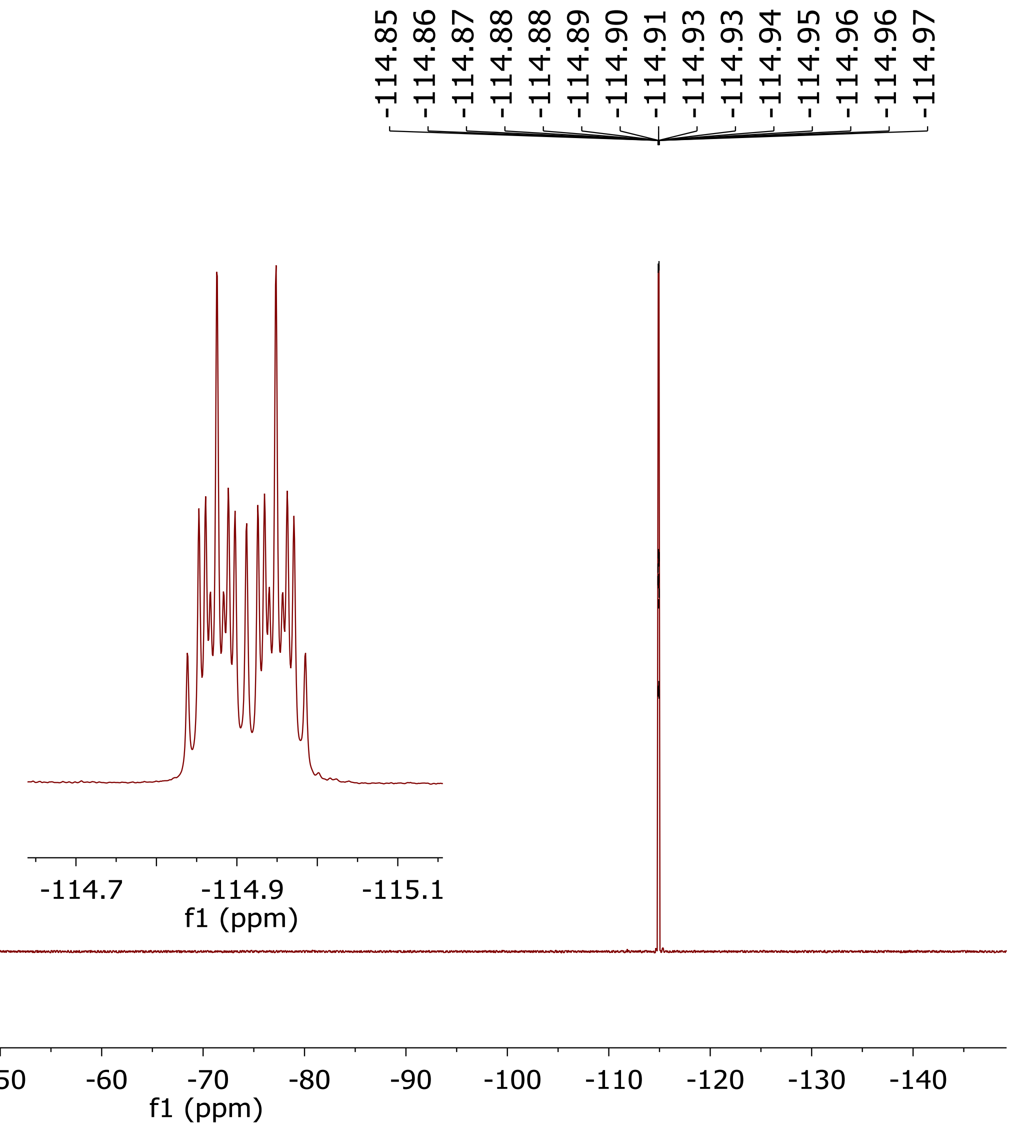

**Ethyl 2,2-bis(4-fluorophenyl)-2-((phenylcyclopropyl)amino)acetate (27) - $^{1}$H NMR (400 MHz, $CDCl_3$):**

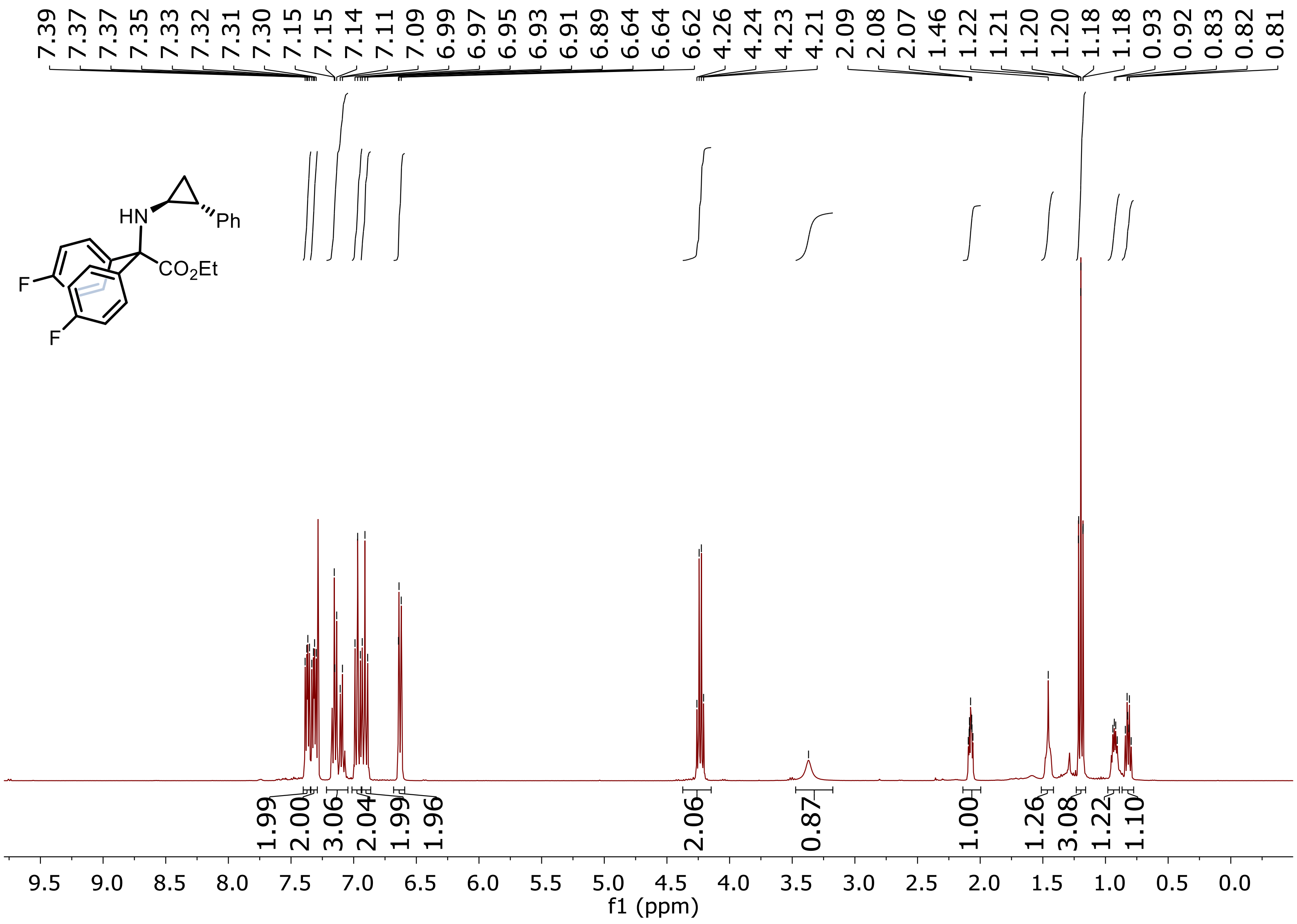

**Ethyl 2,2-bis(4-fluorophenyl)-2-((phenylcyclopropyl)amino)acetate (27) -$^{13}C\{^1H\}$ NMR (101 MHz, $CDCl_3$):**

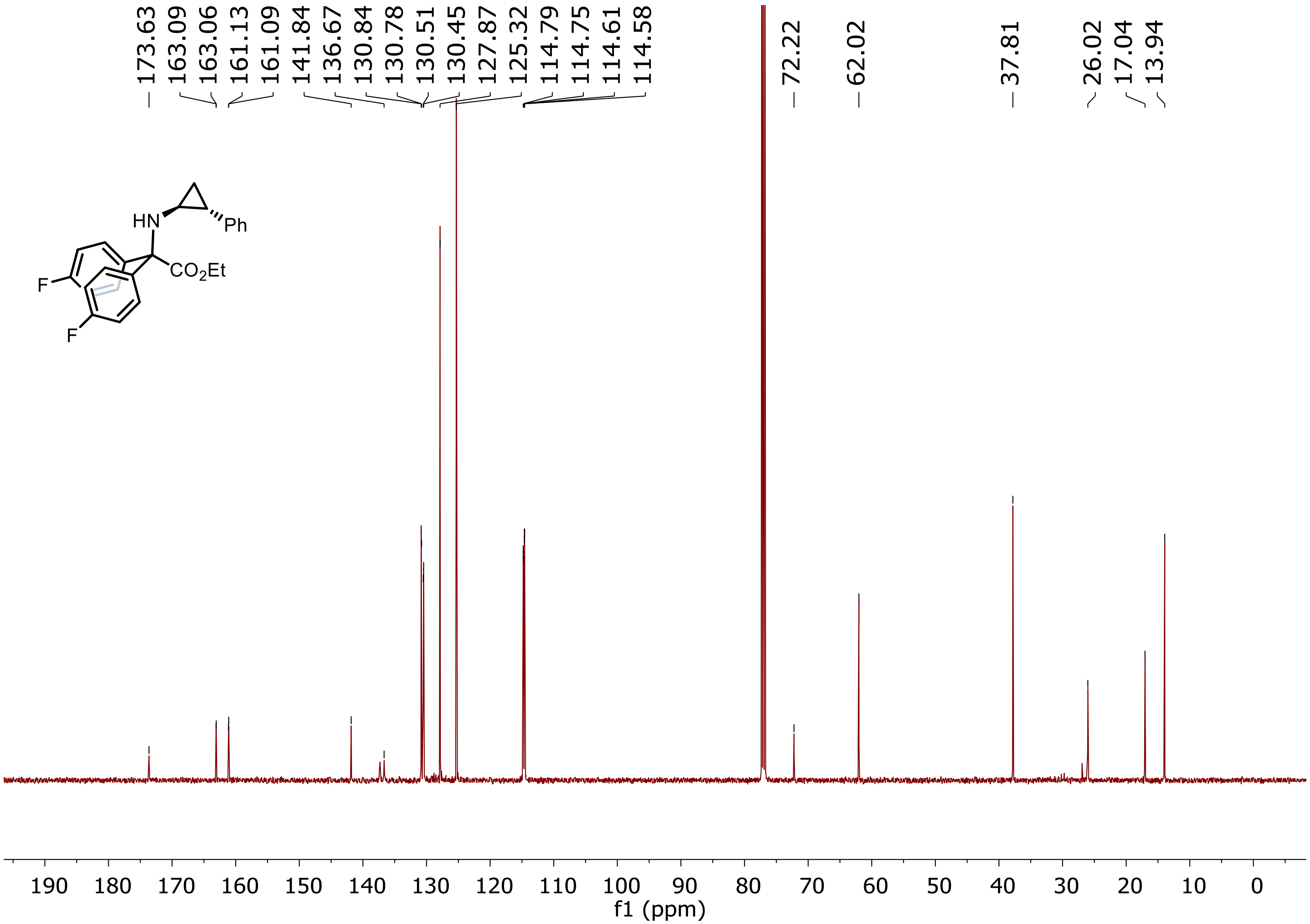

**Ethyl 2,2-bis(4-fluorophenyl)-2-((phenylcyclopropyl)amino)acetate (27) - $^{19}$F NMR (376 MHz, CDCl3):**

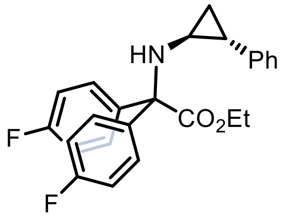


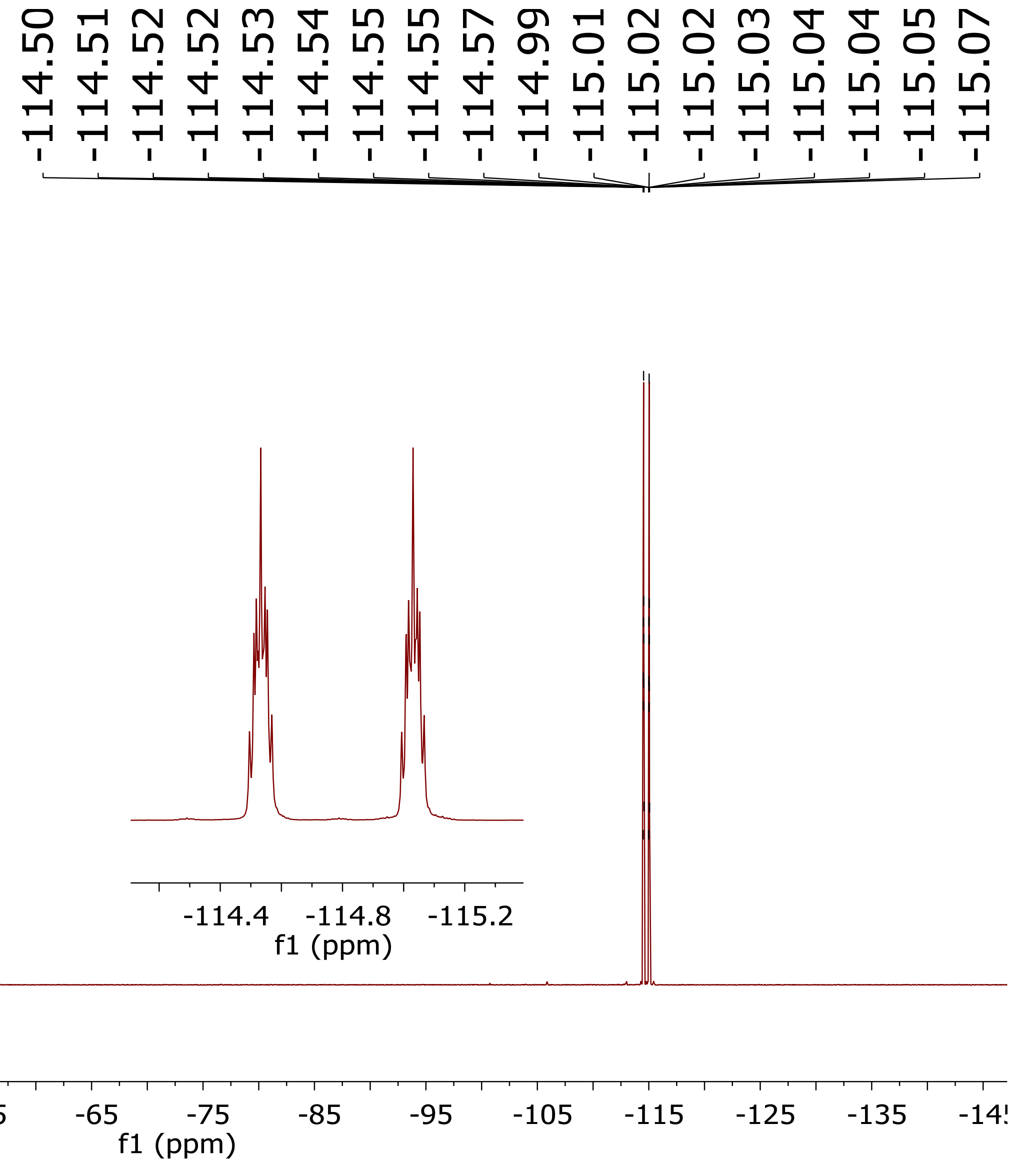

***tert*-butyl (2-ethoxy-1,1-bis(4-fluorophenyl)-2-oxoethyl)-L-valinate (28) - $^{1}$H NMR (400 MHz, $CDCl_3$):**

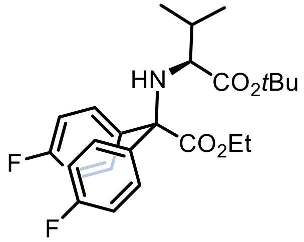


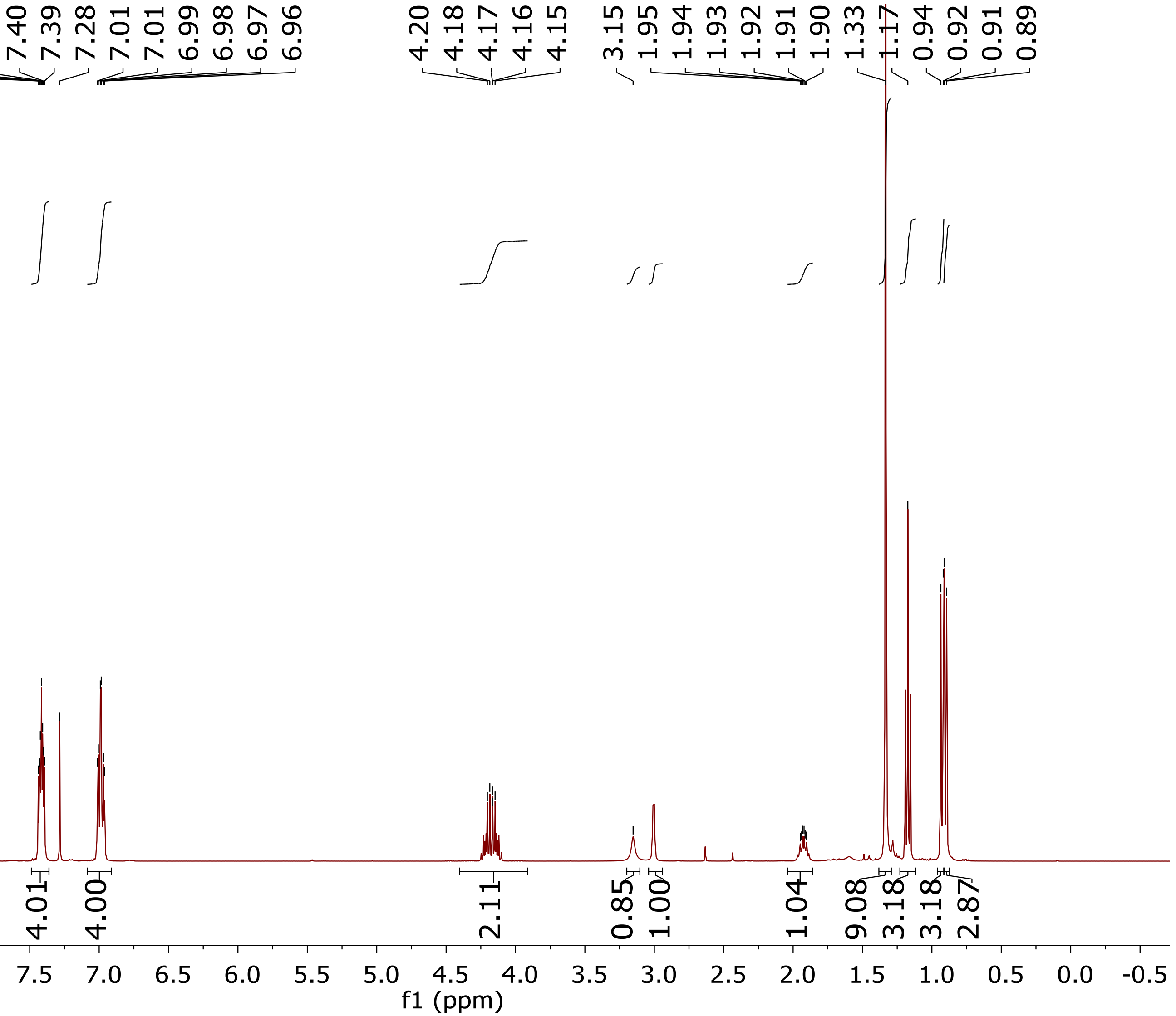

***tert*-butyl (2-ethoxy-1,1-bis(4-fluorophenyl)-2-oxoethyl)-L-valinate (28) - $^{13}C\{^1H\}$ NMR (101 MHz, $CDCl_3$):**

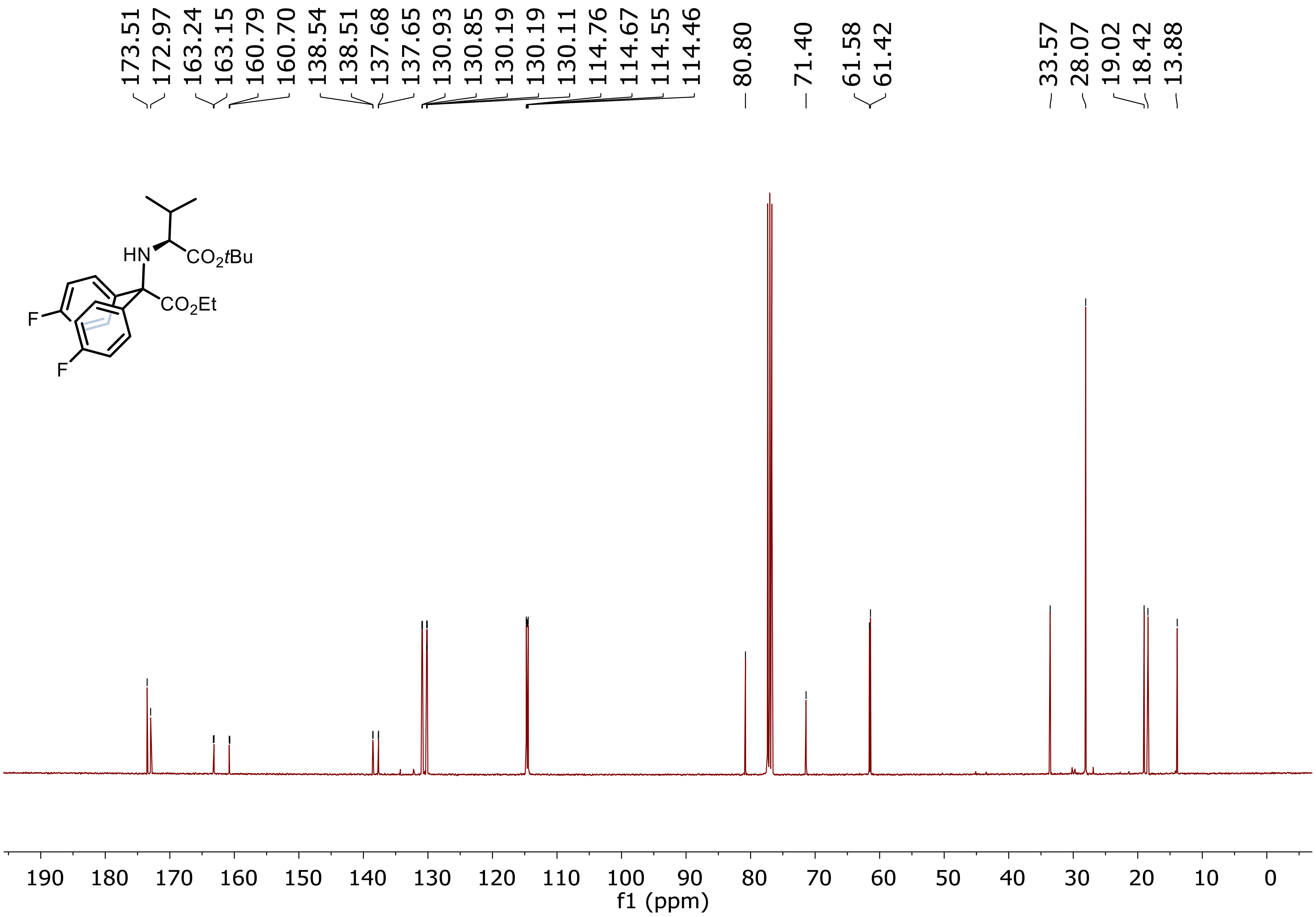

***tert*-butyl (2-ethoxy-1,1-bis(4-fluorophenyl)-2-oxoethyl)-L-valinate (28) - $^{19}$F NMR (376 MHz, $CDCl_3$):**

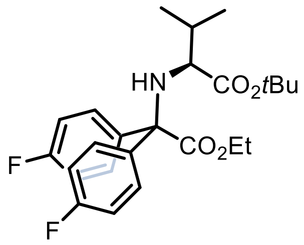

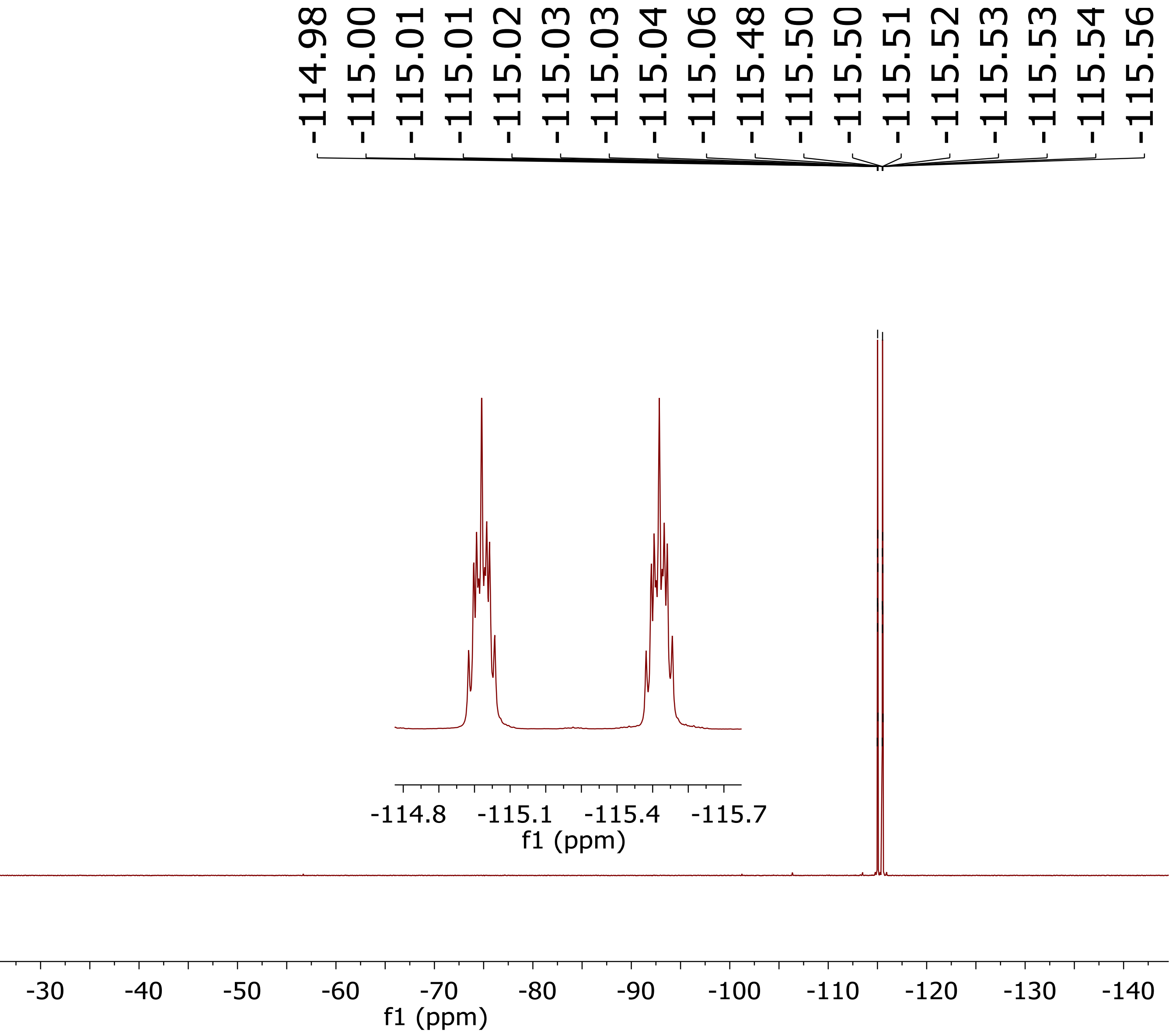

**Benzyl (2-ethoxy-1,1-bis(4-fluorophenyl)-2-oxoethyl)-L-prolinate (29) - $^{1}H$ NMR (400 MHz, $CDCl_3$):**

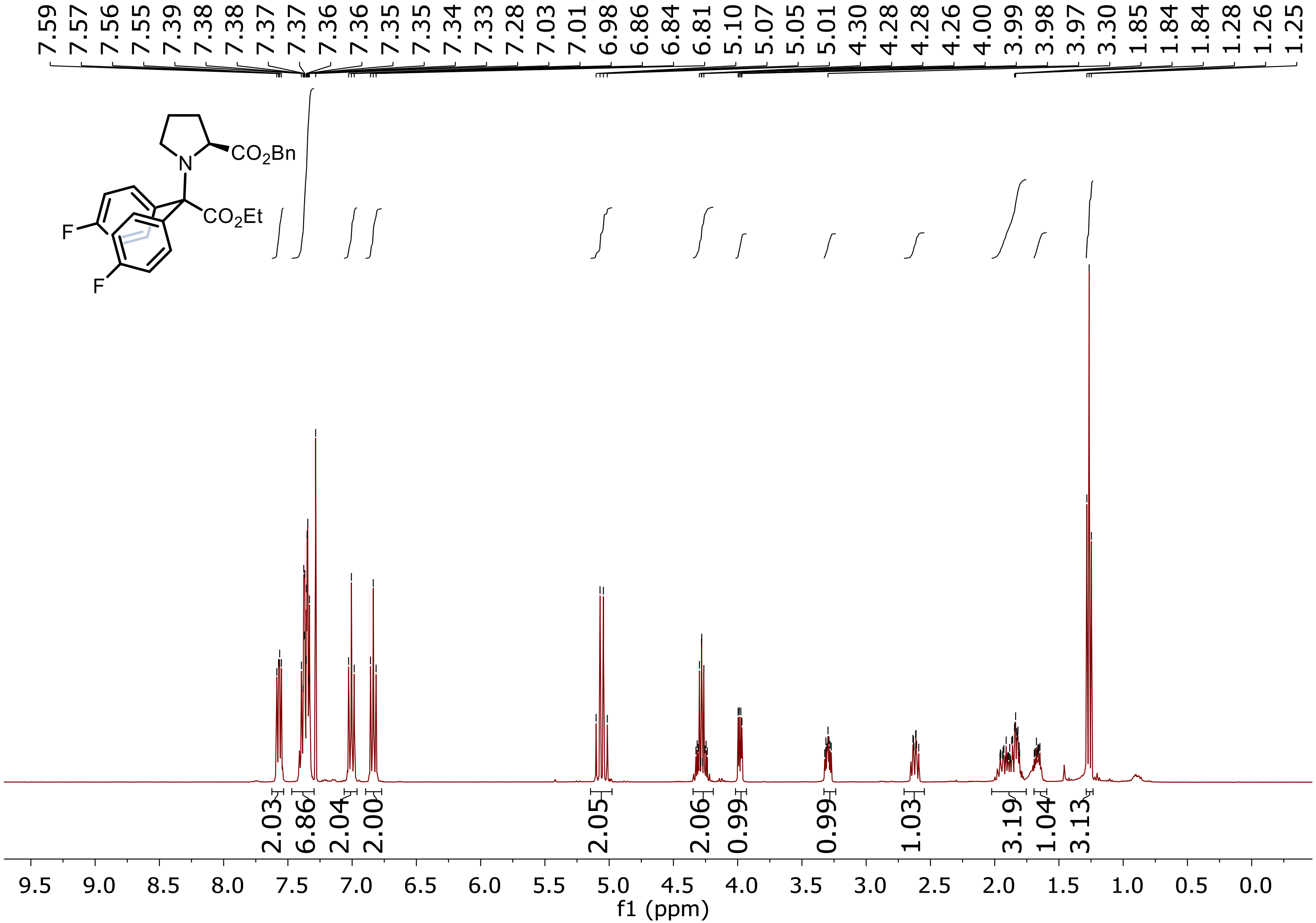

**Benzyl (2-ethoxy-1,1-bis(4-fluorophenyl)-2-oxoethyl)-L-prolinate (29) - $^{13}C\{^1H\}$ NMR (101 MHz, $CDCl_3$):**

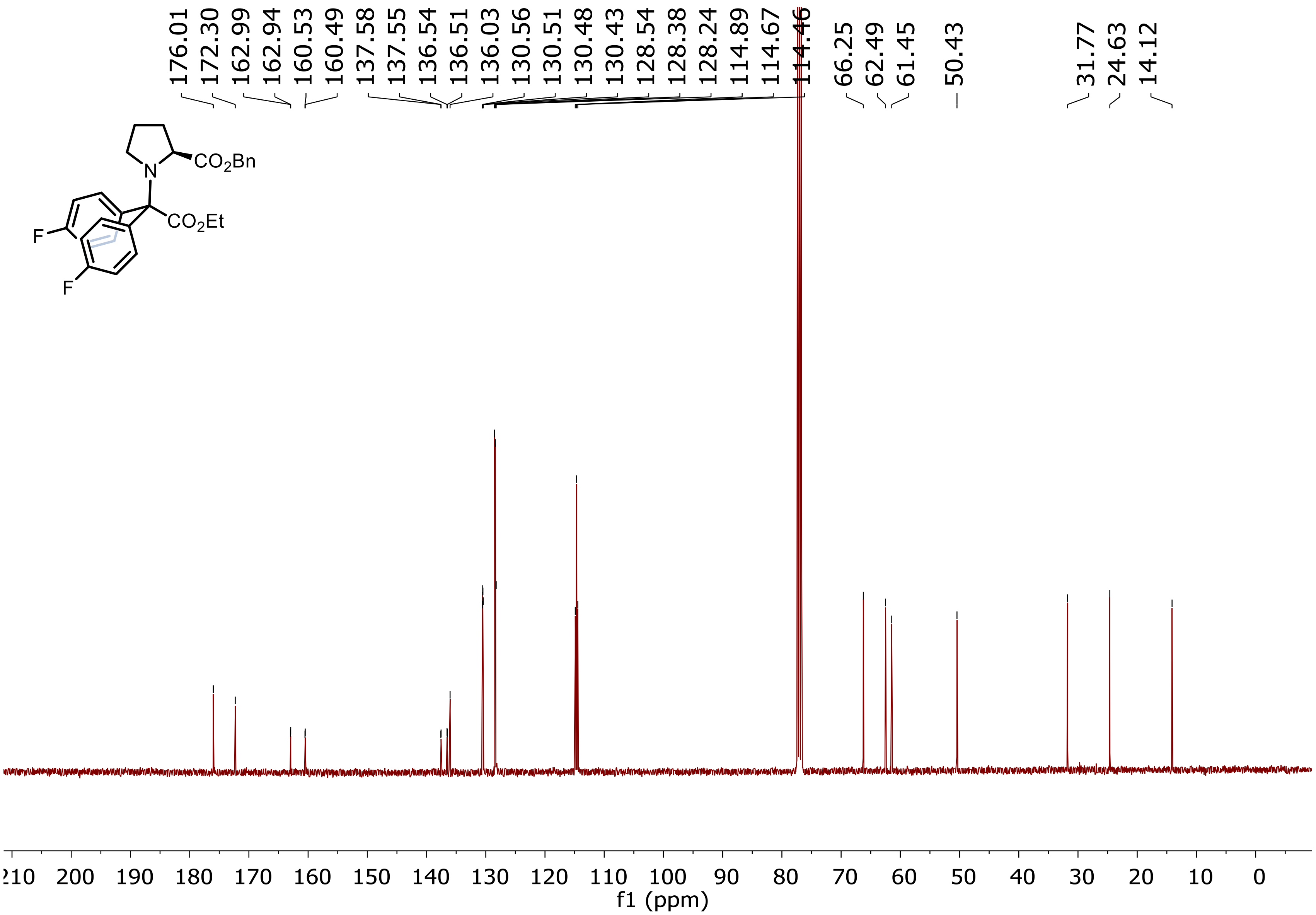

**Benzyl (2-ethoxy-1,1-bis(4-fluorophenyl)-2-oxoethyl)-L-prolinate (29) - $^{19}$F NMR (376 MHz, $CDCl_3$):**

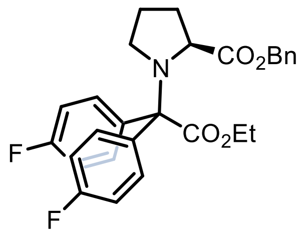


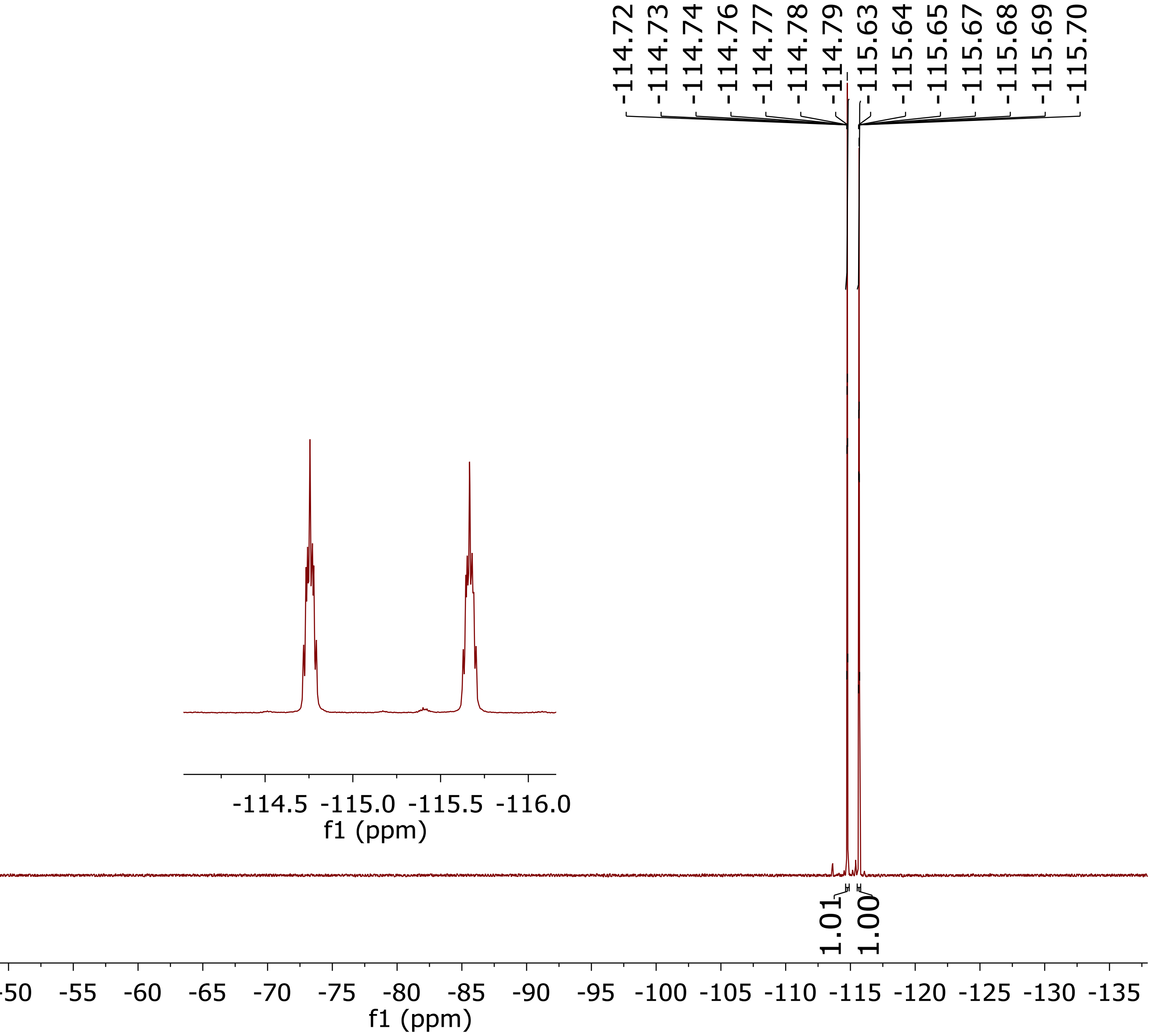

**Ethyl 2-(4-ethoxyphenyl)-2-(4-fluorophenyl)-2-(propylamino)acetate (30) - $^{1}H$ NMR (400 MHz, $CDCl_3$):**

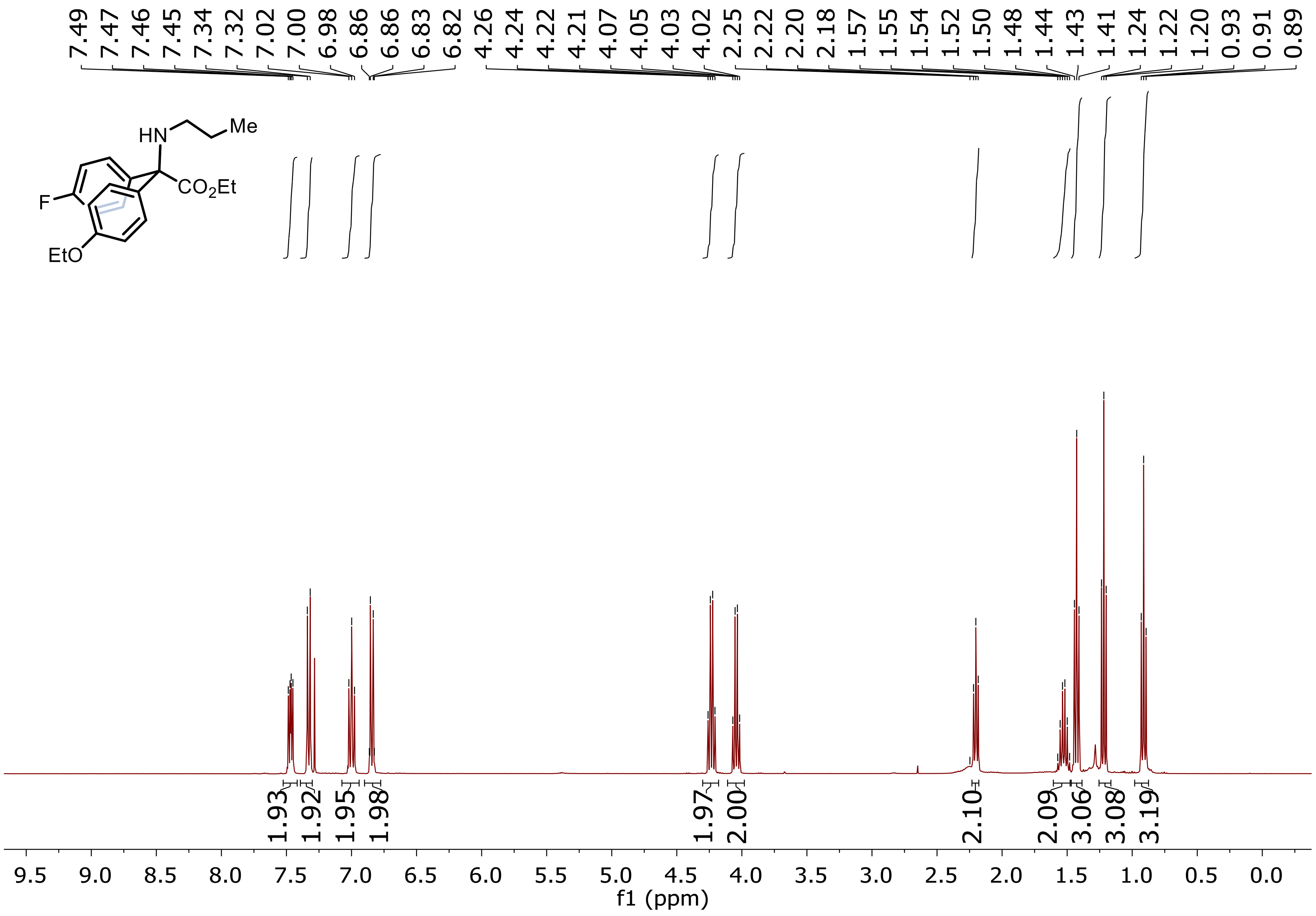

**Ethyl 2-(4-ethoxyphenyl)-2-(4-fluorophenyl)-2-(propylamino)acetate (30) - $^{13}C\{^{1}H\}$ NMR (101 MHz, $CDCl_3$):**

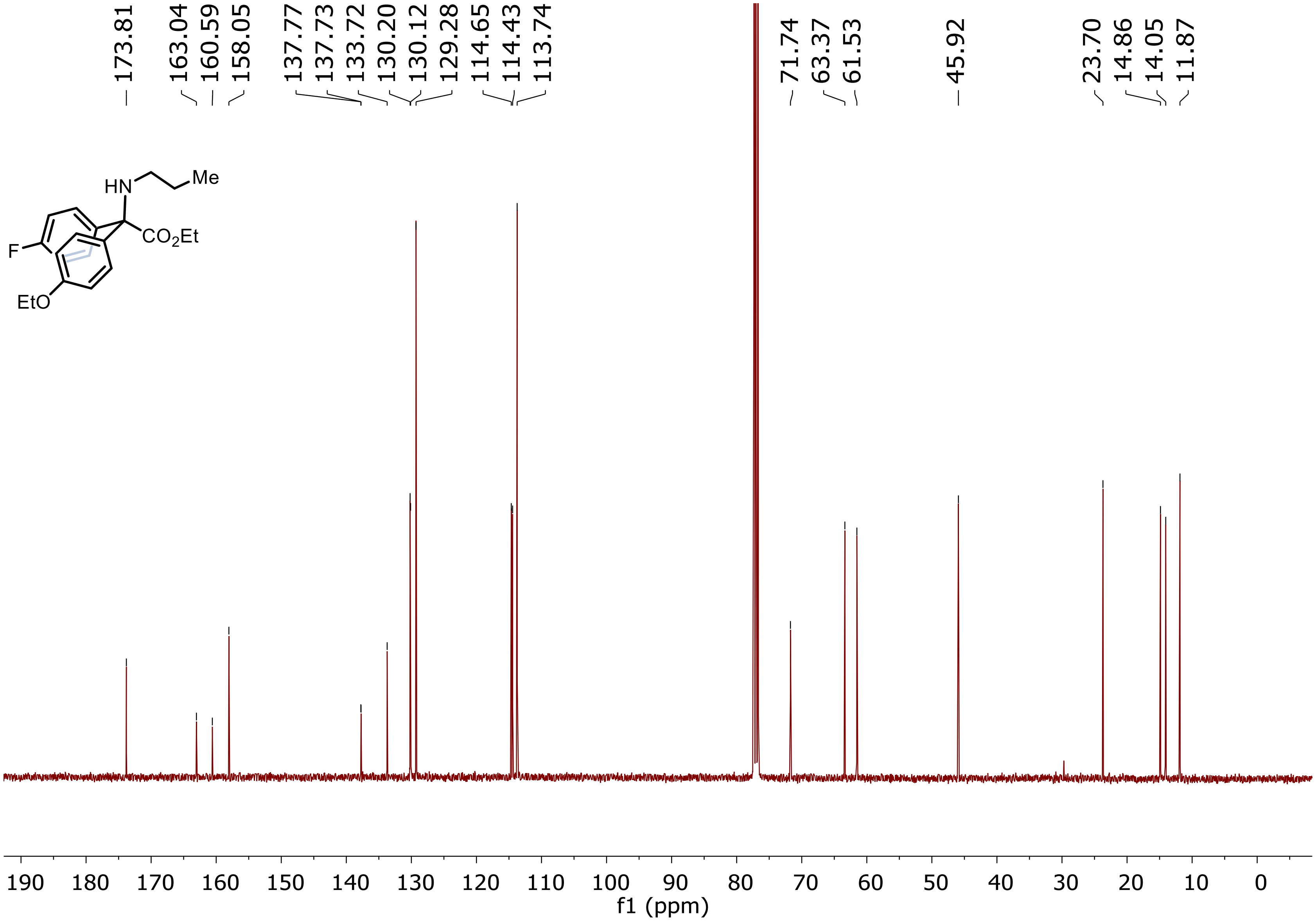

**Ethyl 2-(4-ethoxyphenyl)-2-(4-fluorophenyl)-2-(propylamino)acetate (30) - $^{19}$F NMR (376 MHz, $CDCl_3$):**

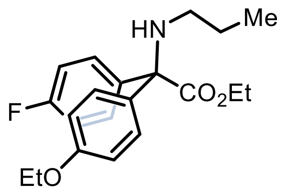


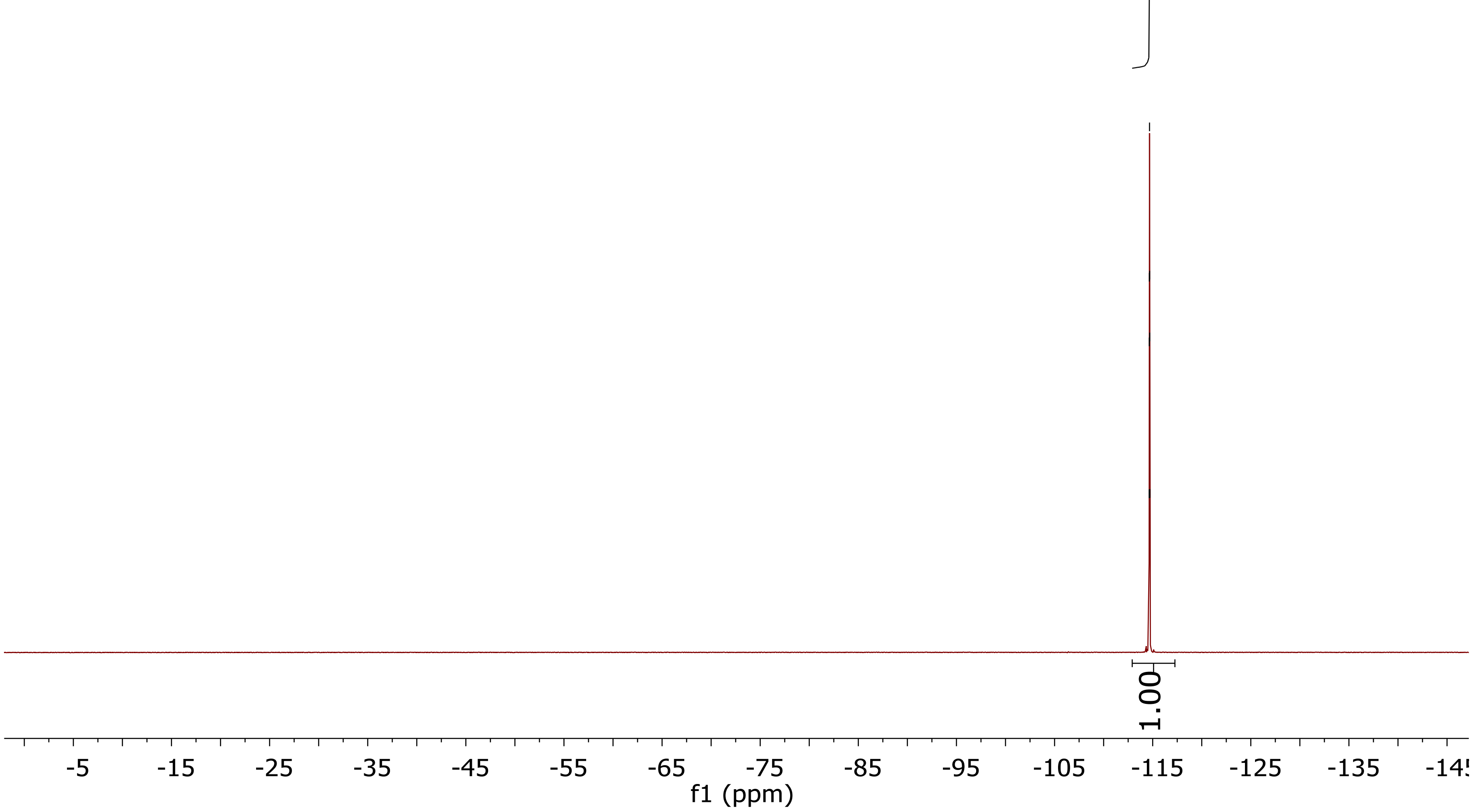

**Ethyl 2-(4-fluorophenyl)-2-(propylamino)-2-(p-tolyl)acetate (31) - $^{1}$H NMR (400 MHz, $CDCl_3$):**

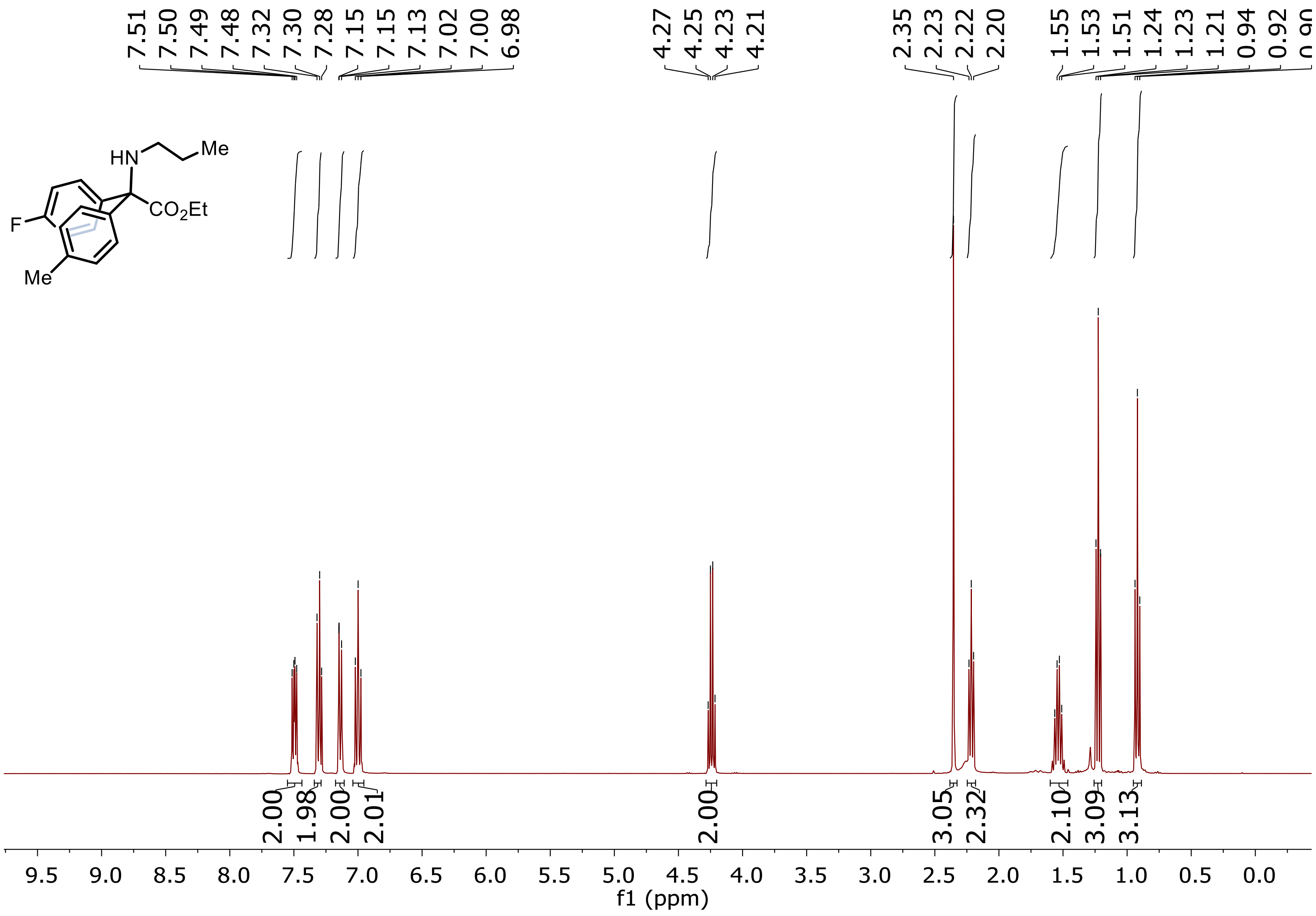

**Ethyl 2-(4-fluorophenyl)-2-(propylamino)-2-(p-tolyl)acetate (31) - $^{13}C\{^1H\}$ NMR (101 MHz, $CDCl_3$):**

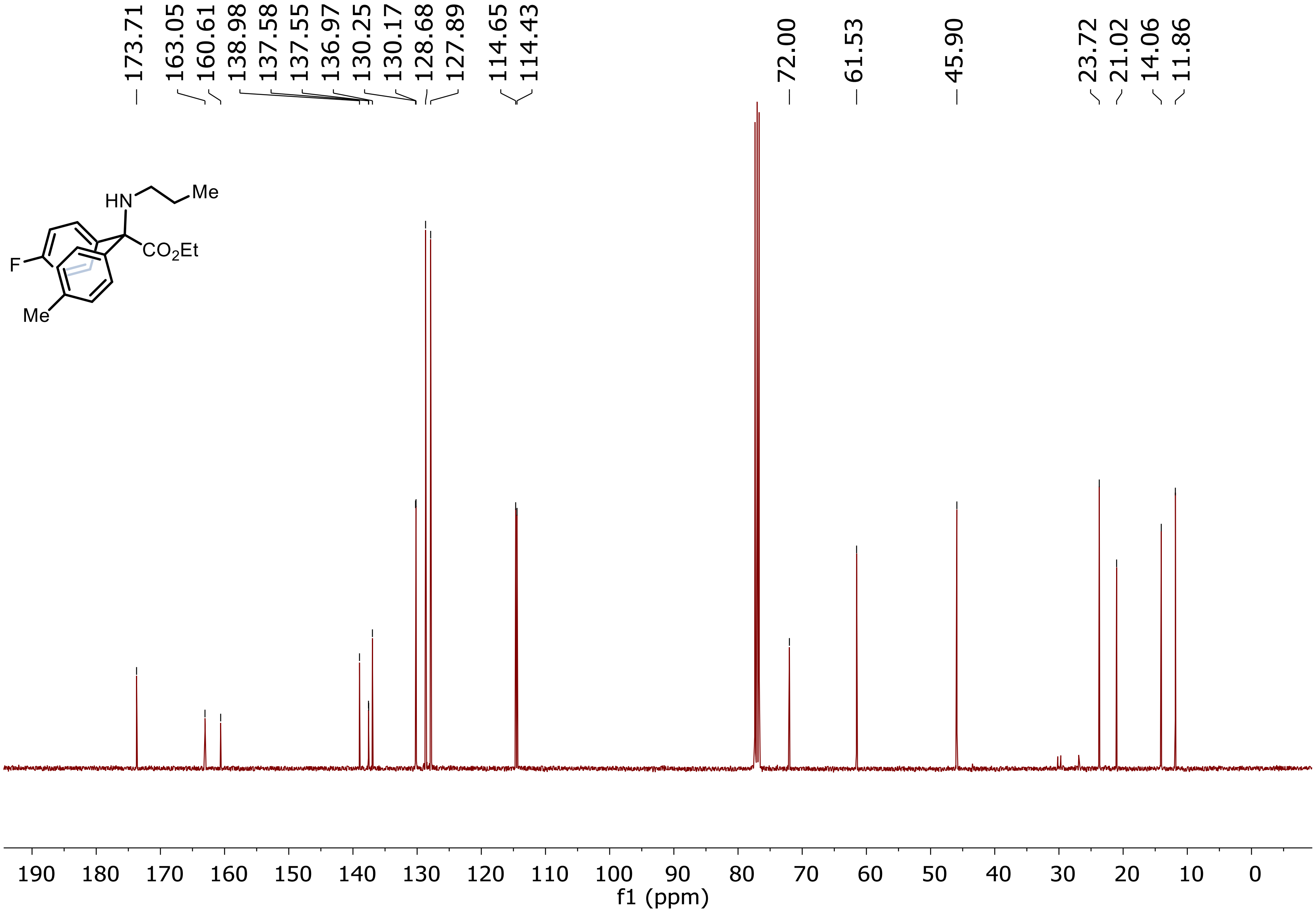

**Ethyl 2-(4-fluorophenyl)-2-(propylamino)-2-(p-tolyl)acetate (31) - $^{19}$F NMR (376 MHz, $CDCl_3$):**

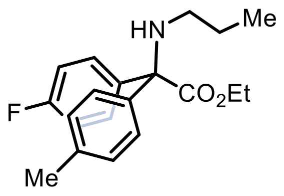

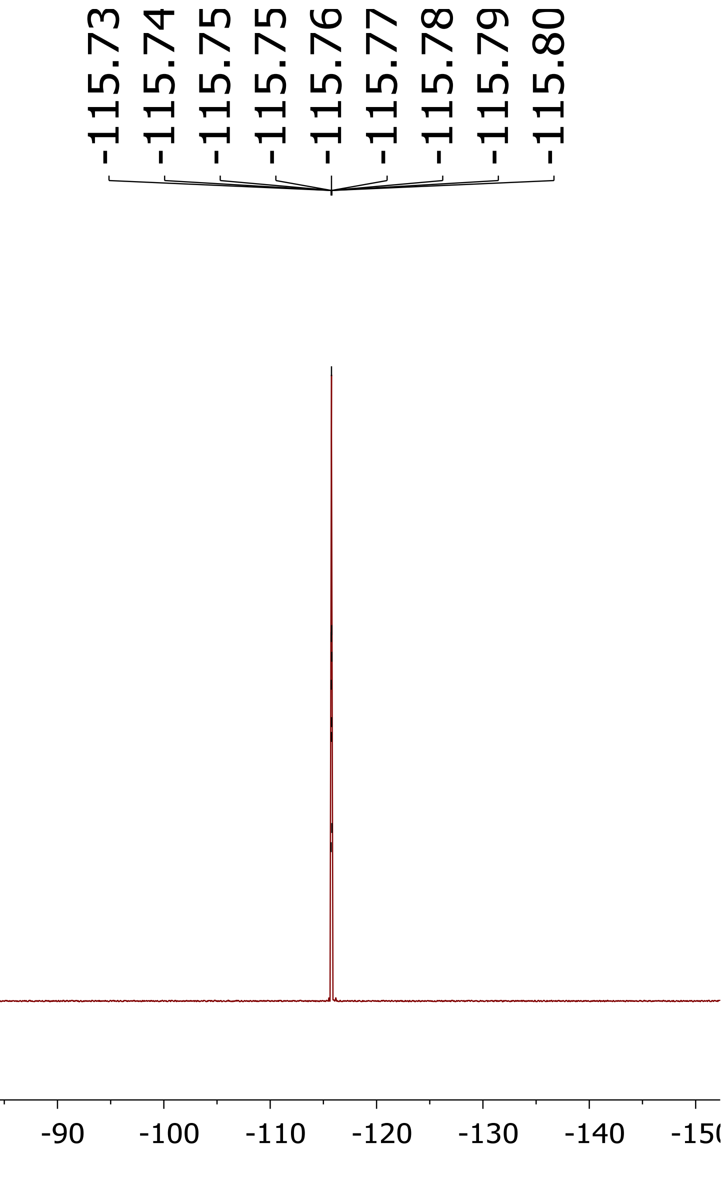

**Ethyl 2-(4-(tert-butyl)phenyl)-2-(4-fluorophenyl)-2-(propylamino)acetate (32) - $^{1}$H NMR (400 MHz, $CDCl_3$):**

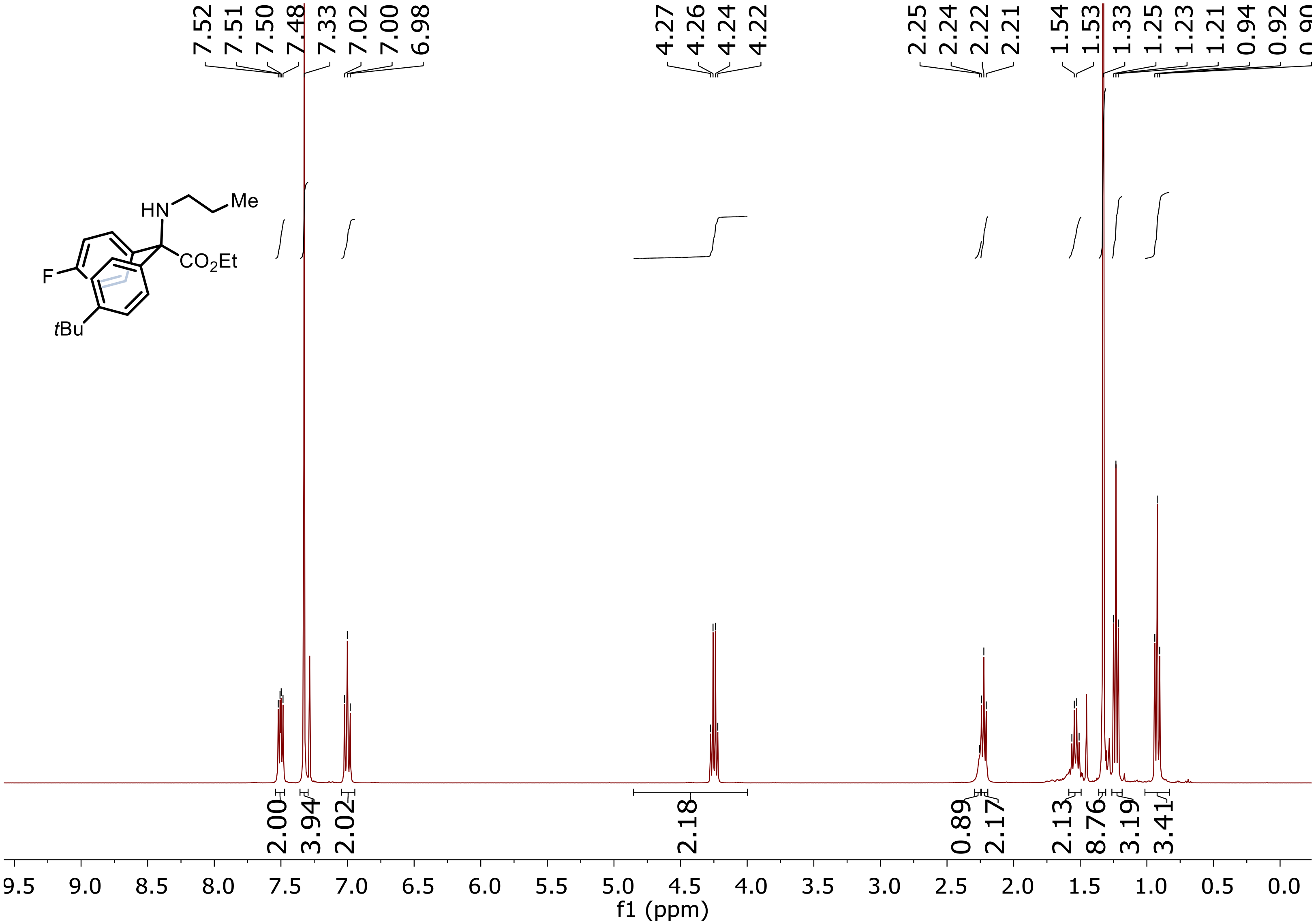

**Ethyl 2-(4-(tert-butyl)phenyl)-2-(4-fluorophenyl)-2-(propylamino)acetate (32) - $^{13}C\{^{1}H\}$ NMR (101 MHz, $CDCl_3$):**

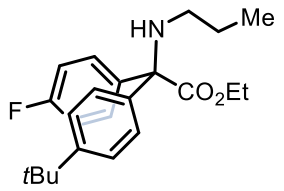


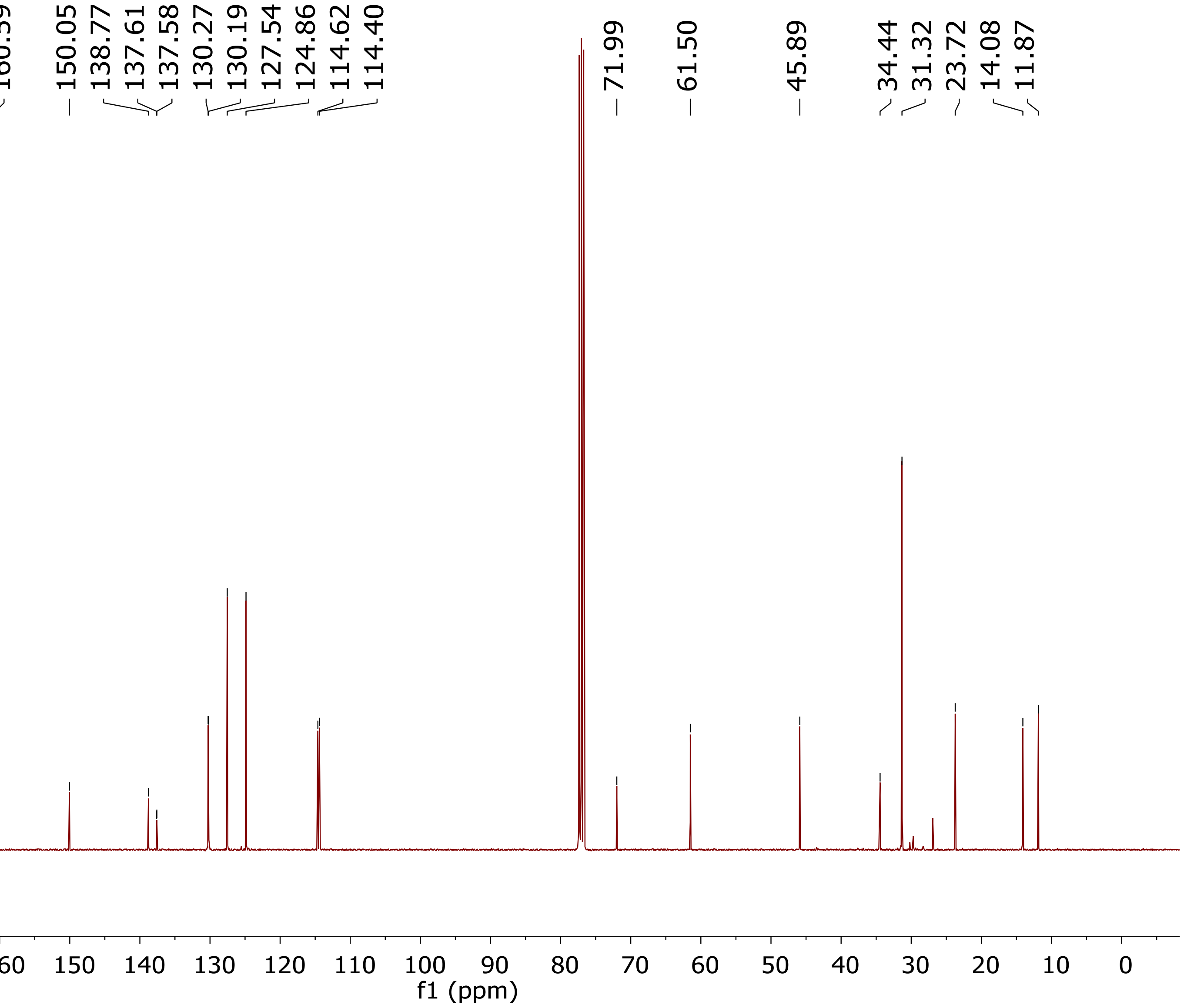

**Ethyl 2-(4-(tert-butyl)phenyl)-2-(4-fluorophenyl)-2-(propylamino)acetate (32) - $^{19}$F NMR (376 MHz, $CDCl_3$):**

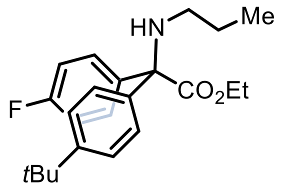


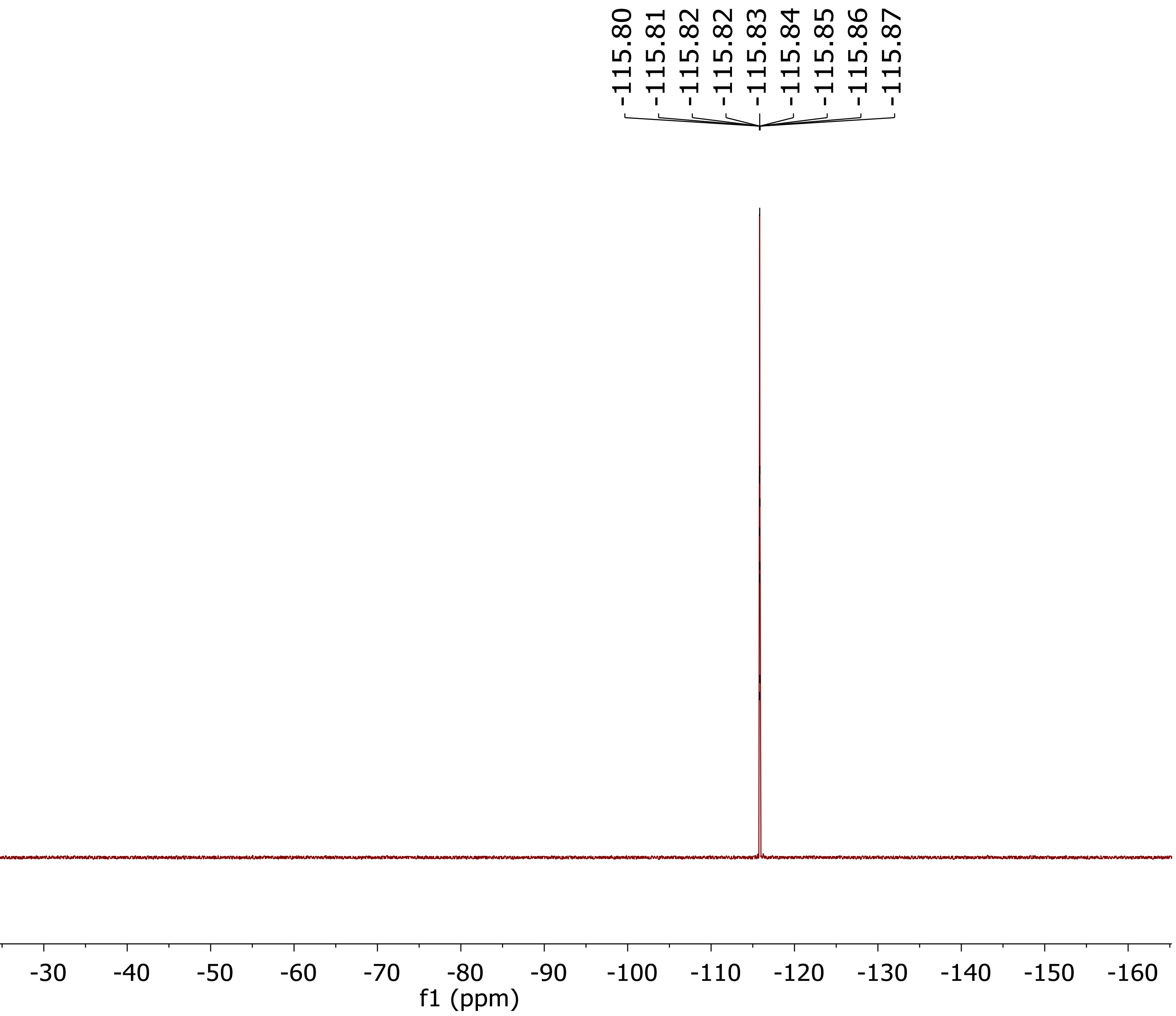

**Ethyl 2-(4-chlorophenyl)-2-(4-fluorophenyl)-2-(propylamino)acetate (33) - $^{1}$H NMR (400 MHz, $CDCl_3$):**

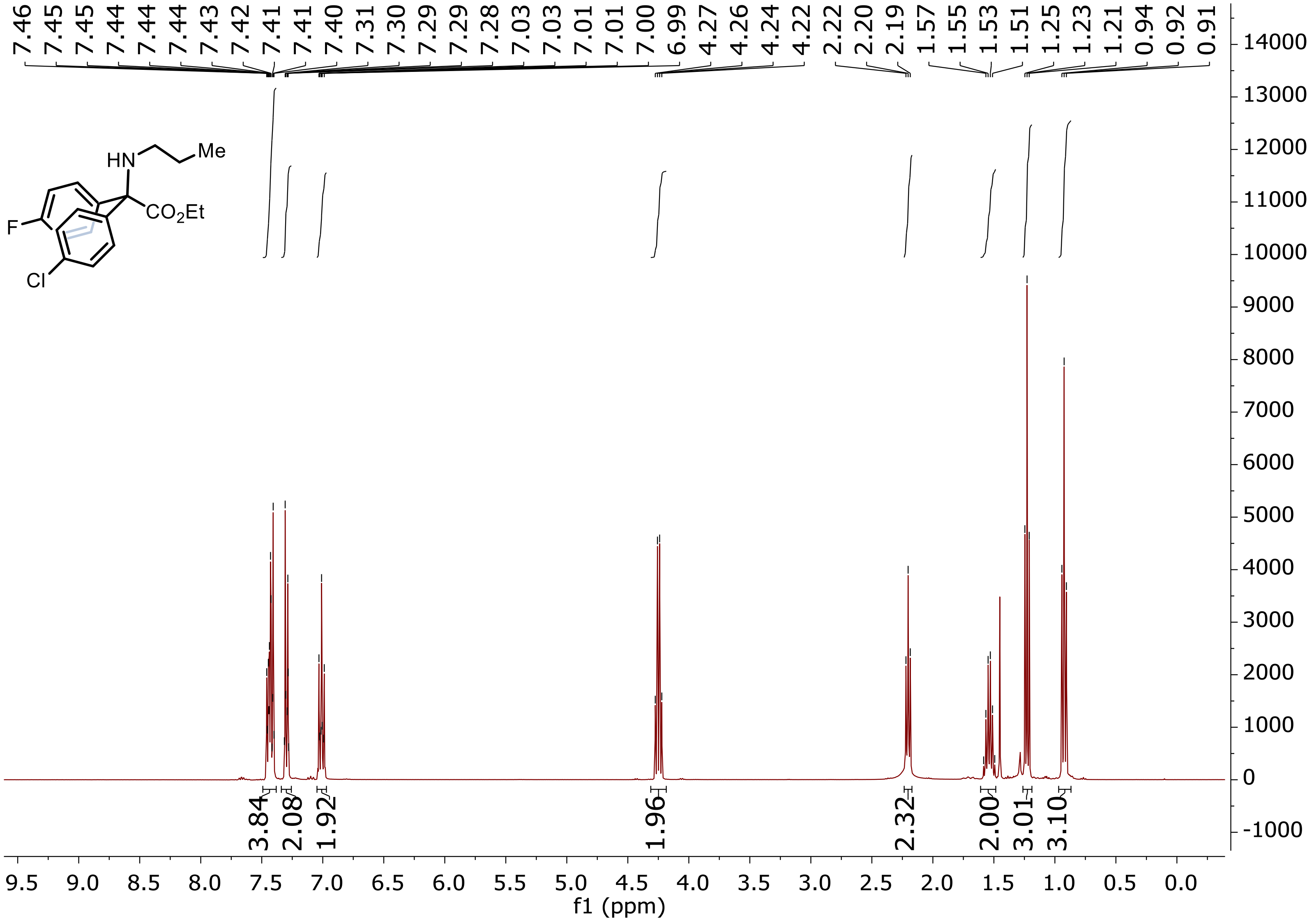

**Ethyl 2-(4-chlorophenyl)-2-(4-fluorophenyl)-2-(propylamino)acetate (33) - $^{13}C\{^{1}H\}$ NMR (101 MHz, $CDCl_3$):**

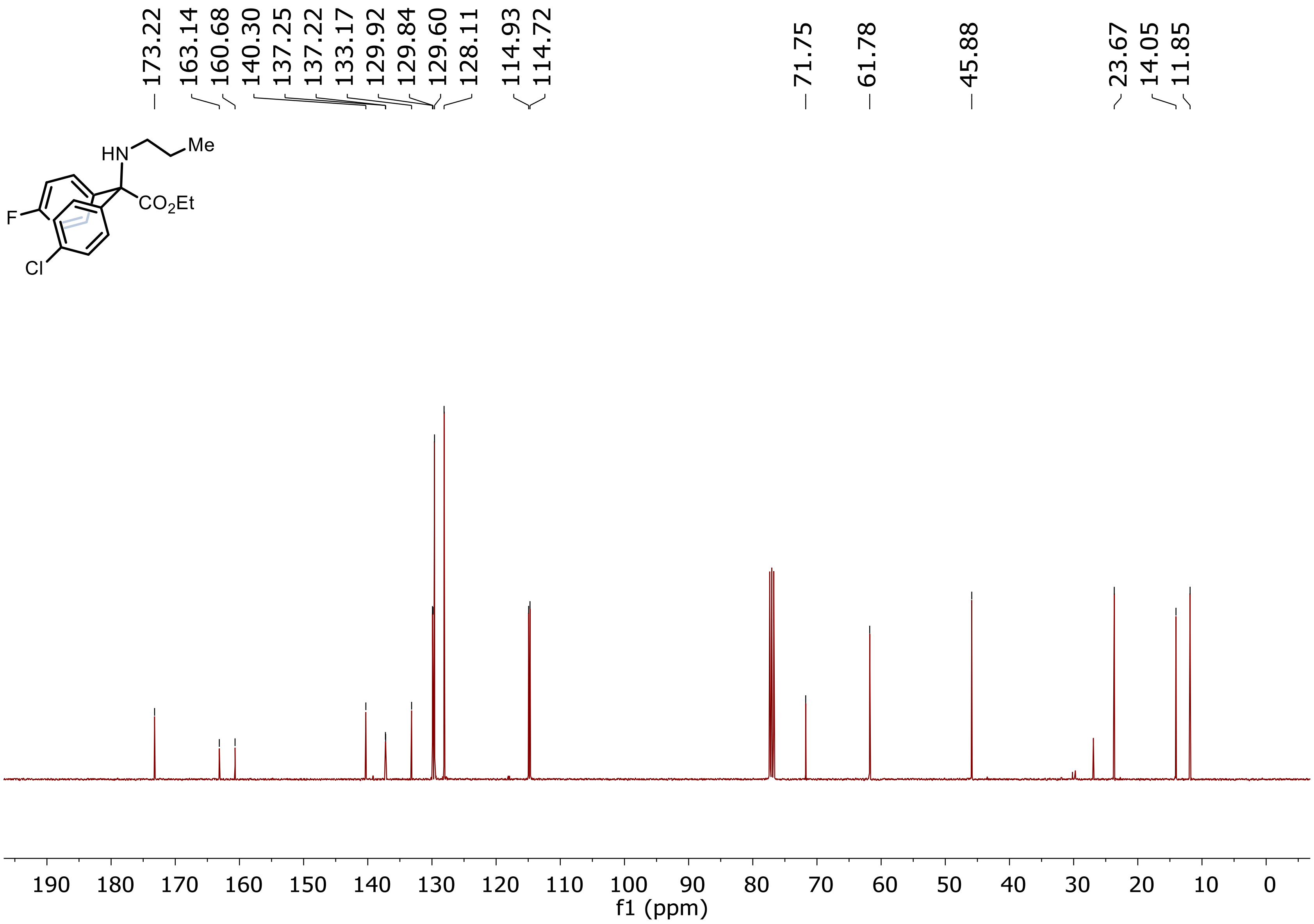

**Ethyl 2-(4-chlorophenyl)-2-(4-fluorophenyl)-2-(propylamino)acetate (33) - $^{19}$F NMR (376 MHz, $CDCl_3$):**

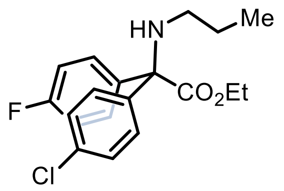

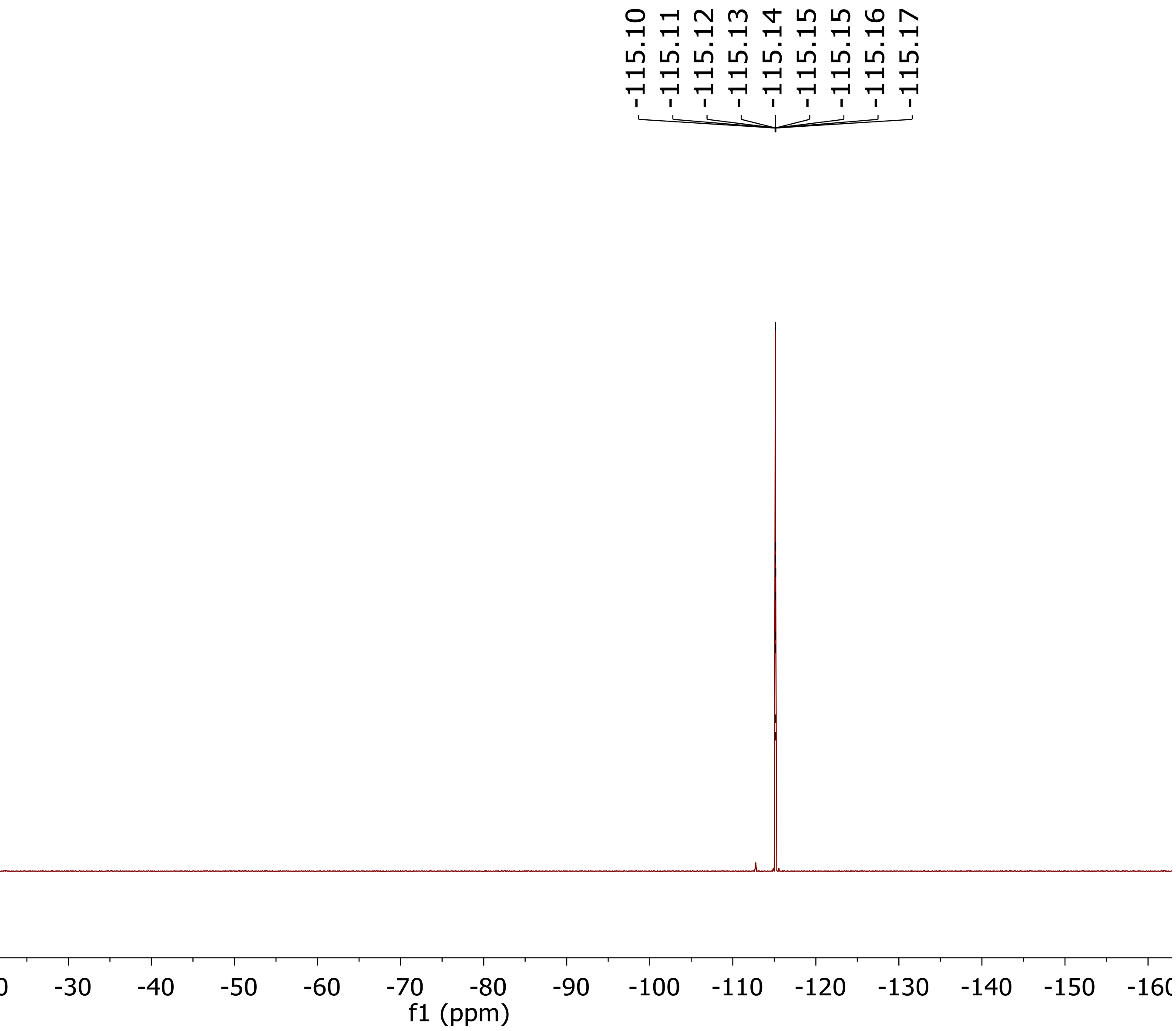

**Ethyl 2-(4-cyanophenyl)-2-(4-fluorophenyl)-2-(propylamino) acetate (34) - $^{1}$H NMR (400 MHz, $CDCl_3$):**

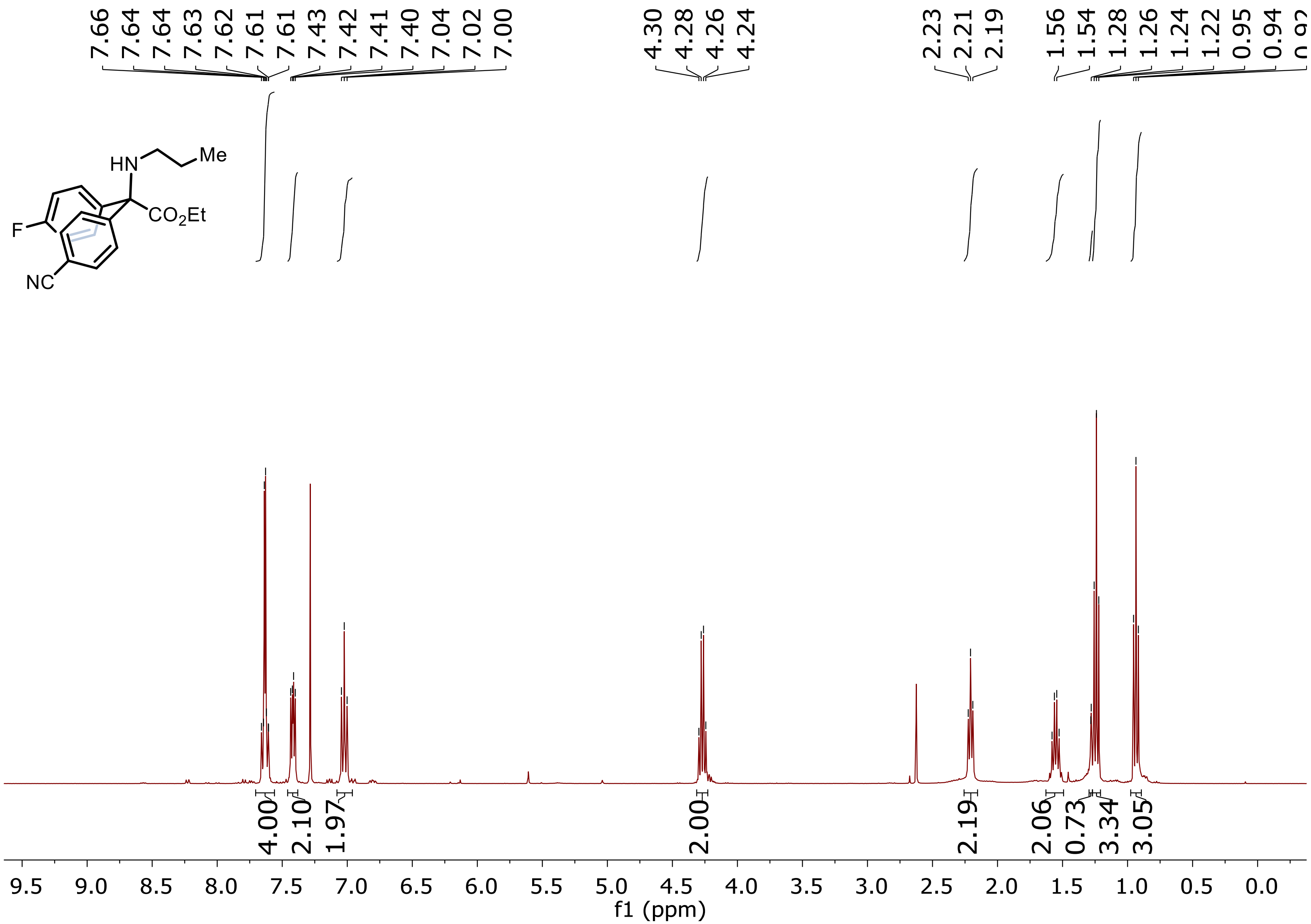

**Ethyl 2-(4-cyanophenyl)-2-(4-fluorophenyl)-2-(propylamino) acetate (34) - $^{13}C\{^{1}H\}$ NMR (101 MHz, $CDCl_3$):**

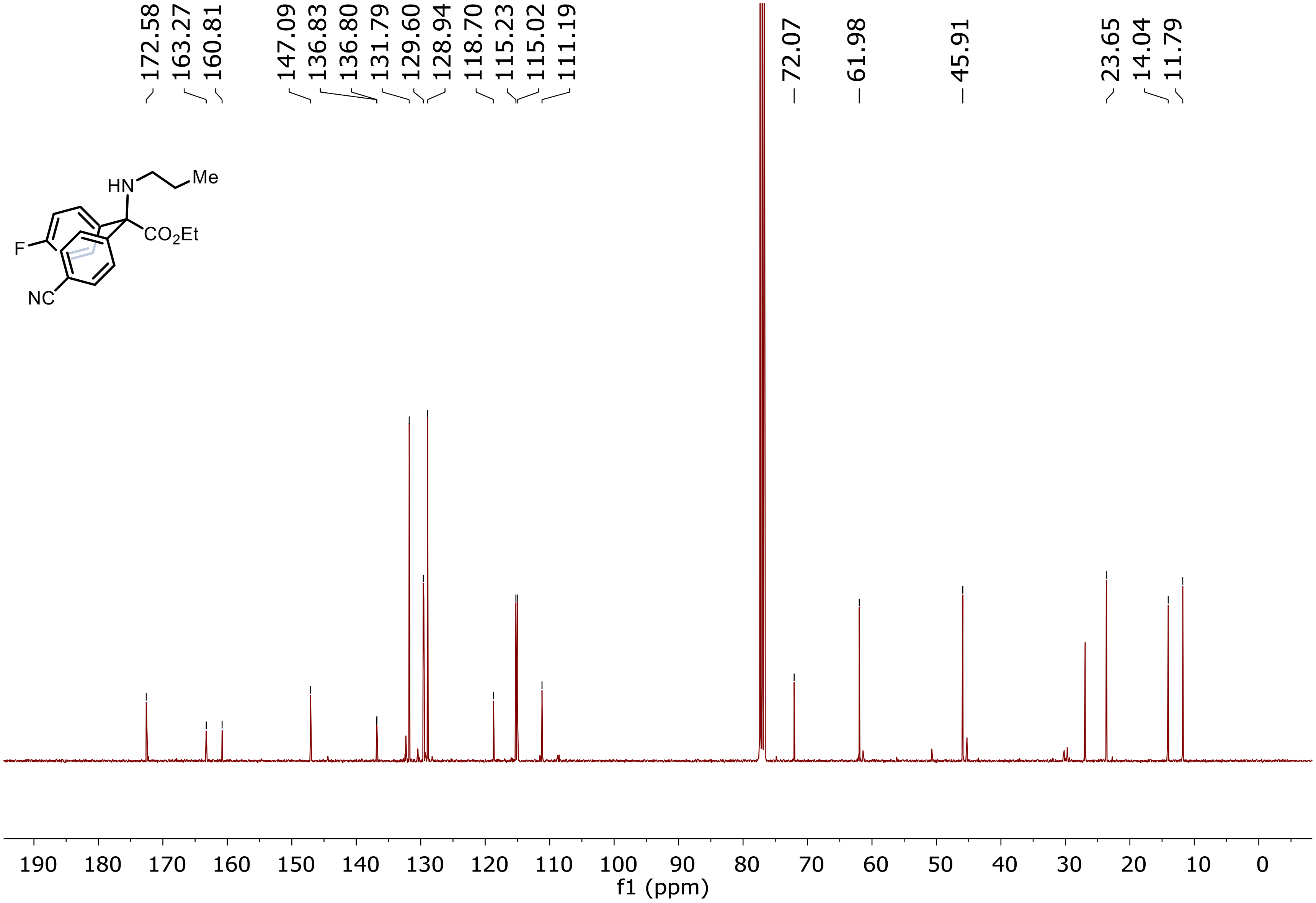

**Ethyl 2-(4-cyanophenyl)-2-(4-fluorophenyl)-2-(propylamino) acetate (34) - $^{19}$F NMR (376 MHz, $CDCl_3$):**

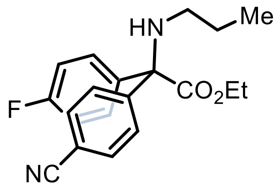


-114.42
-114.43
-114.44
-114.46
-114.47

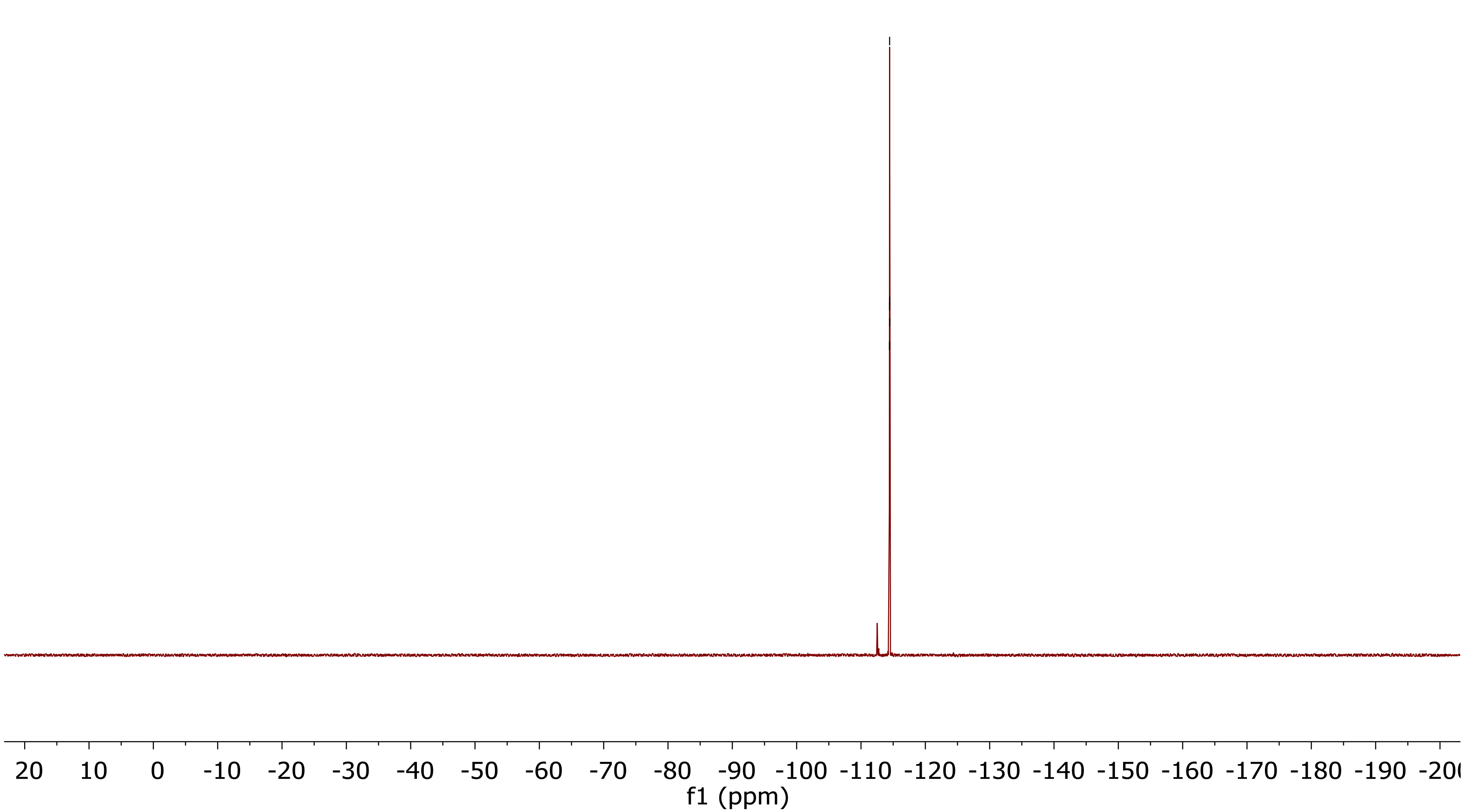

**Ethyl 2-(3-bromo-4-methoxyphenyl)-2-(4-fluorophenyl)-2-(propylamino)acetate (35) - $^{1}$H NMR (400 MHz, $CDCl_3$):**

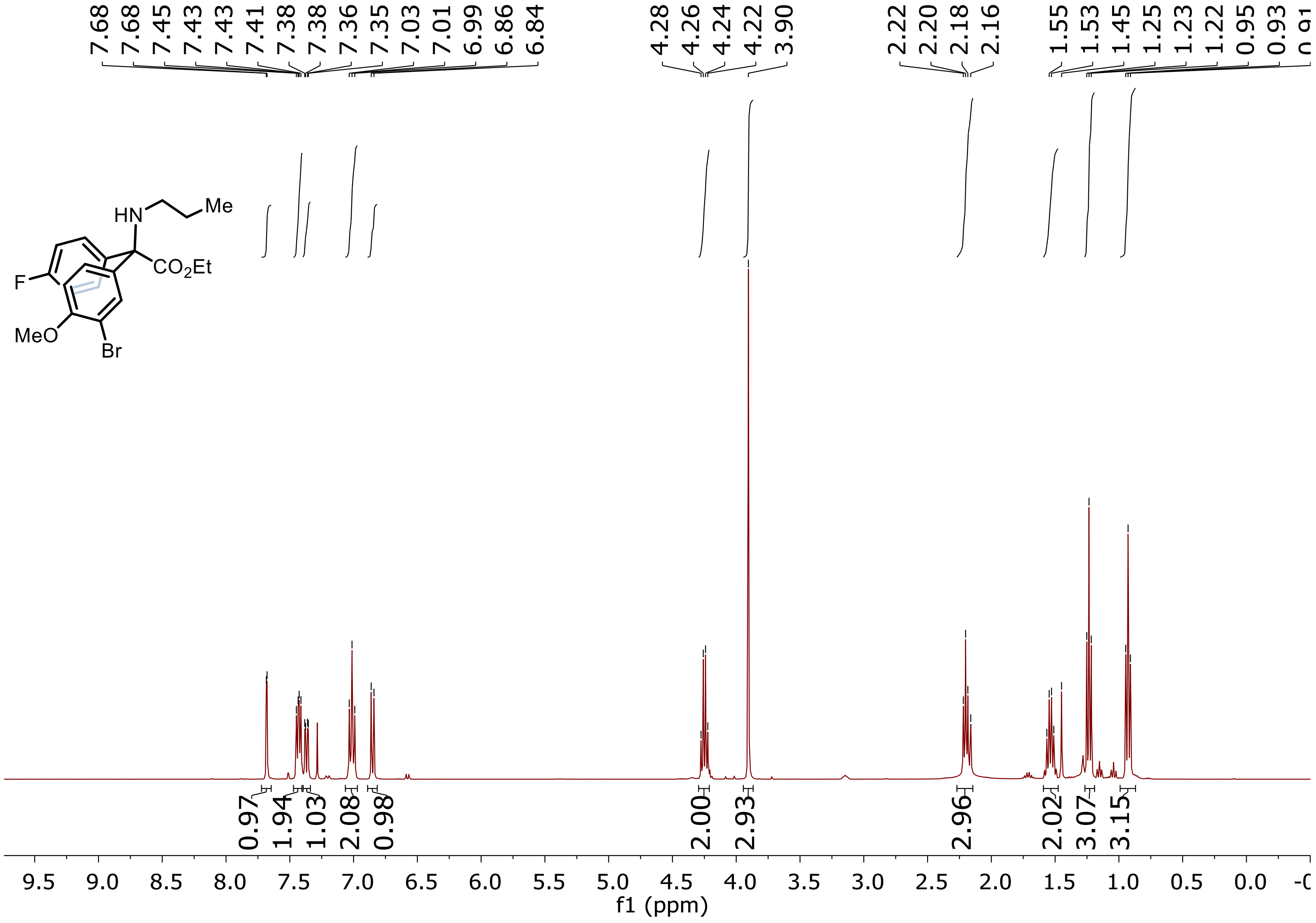

**Ethyl 2-(3-bromo-4-methoxyphenyl)-2-(4-fluorophenyl)-2-(propylamino)acetate (35) - $^{13}C\{^{1}H\}$ NMR (101 MHz, $CDCl_3$):**

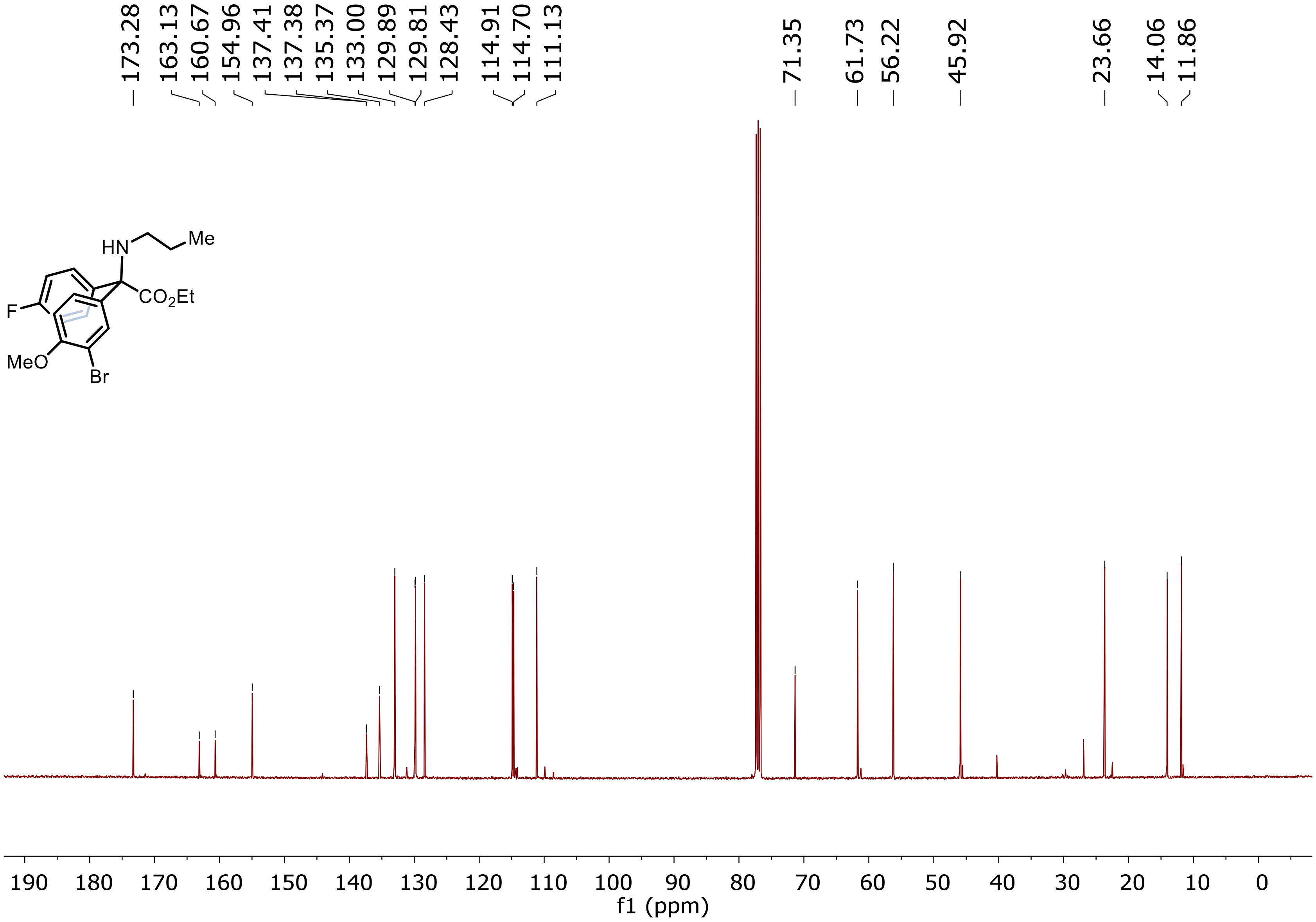

**Ethyl 2-(3-bromo-4-methoxyphenyl)-2-(4-fluorophenyl)-2-(propylamino)acetate (35) - $^{19}$F NMR (376 MHz, $CDCl_3$):**

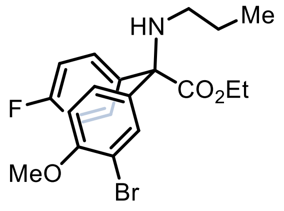


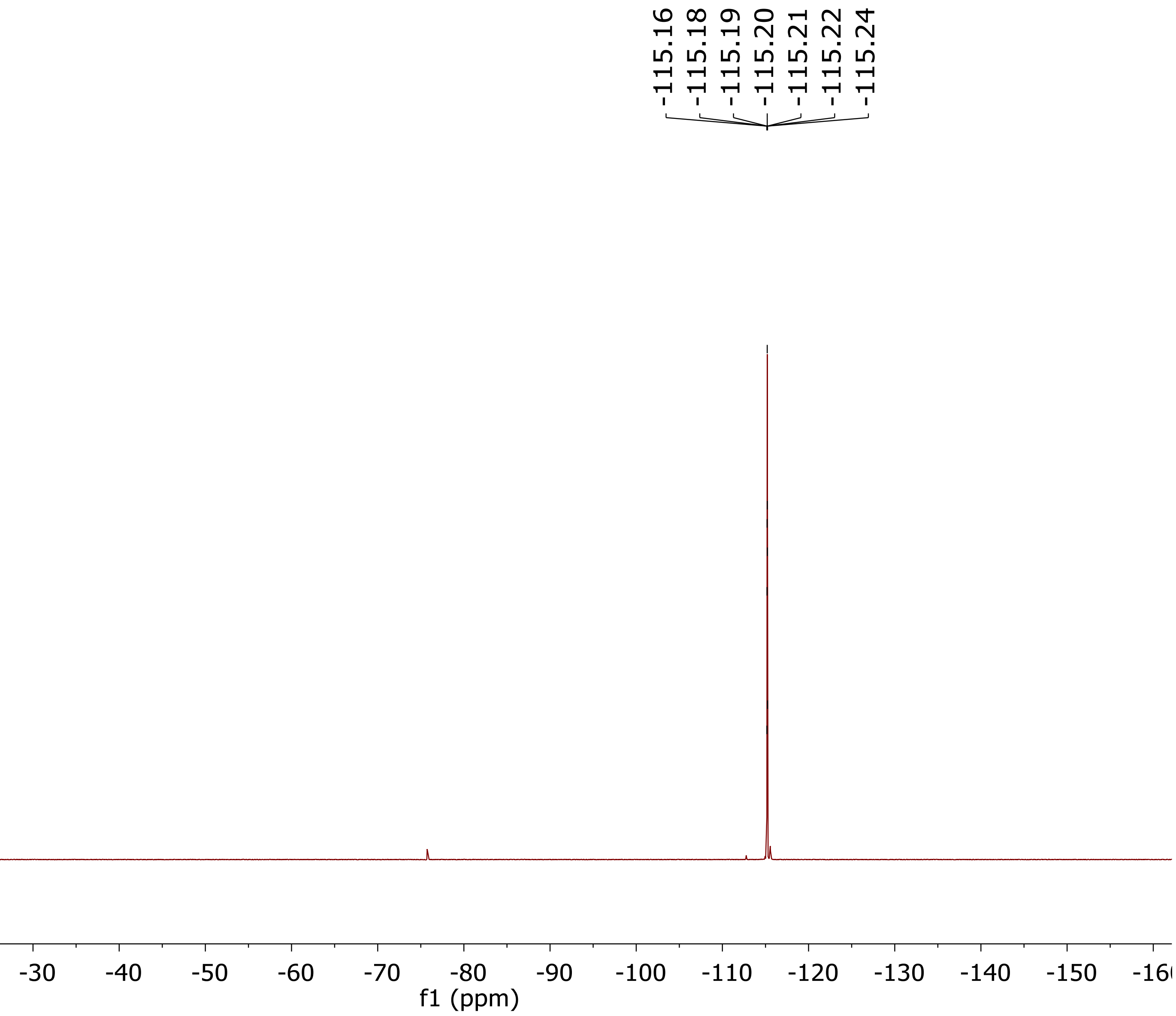

**Ethyl 2-(4-fluorophenyl)-2-(naphthalen-2-yl)-2-(propylamino)acetate (36) - $^{1}$H NMR (400 MHz, $CDCl_3$):**

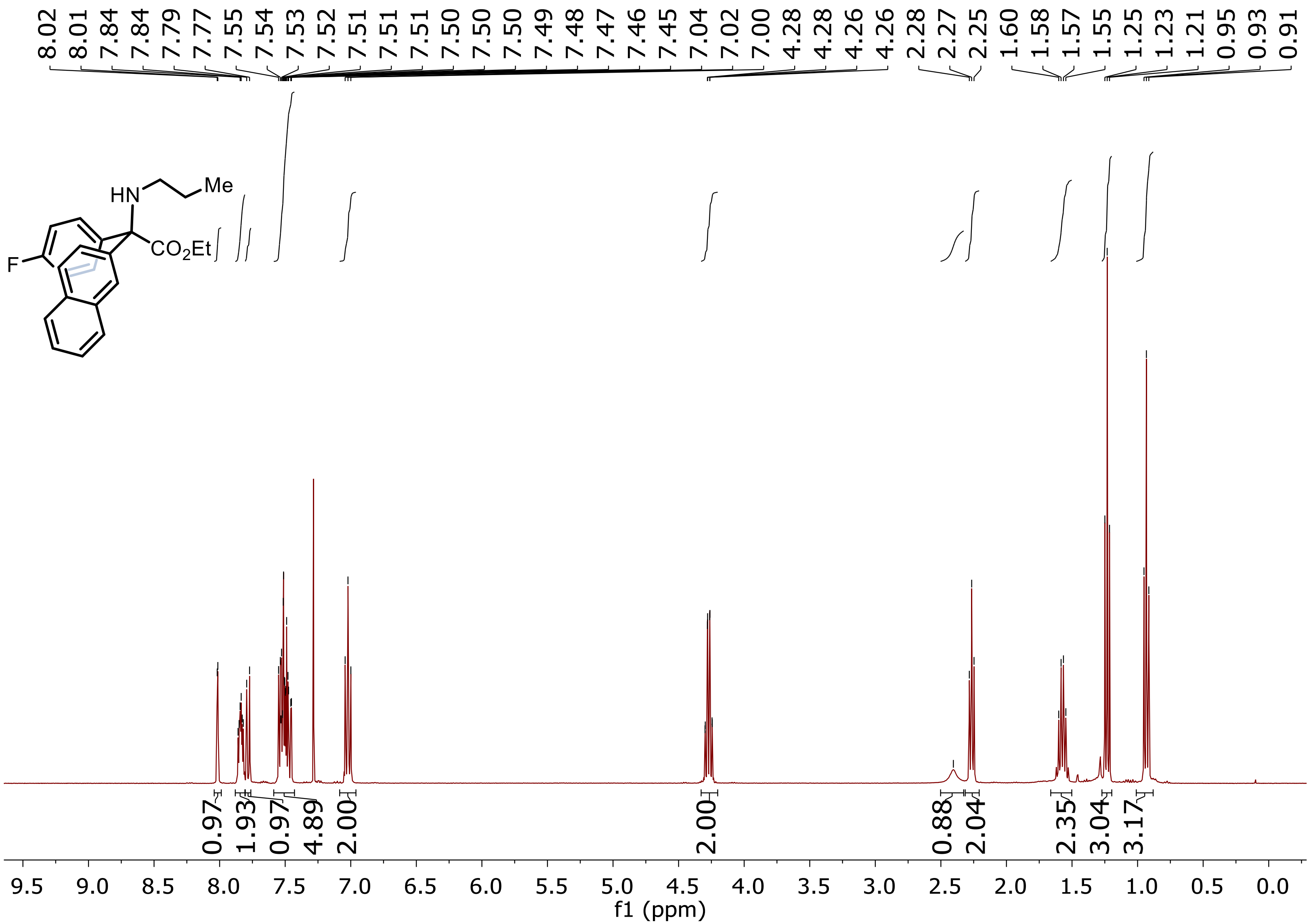

**Ethyl 2-(4-fluorophenyl)-2-(naphthalen-2-yl)-2-(propylamino)acetate (36) - $^{13}C\{^1H\}$ NMR (101 MHz, $CDCl_3$):**

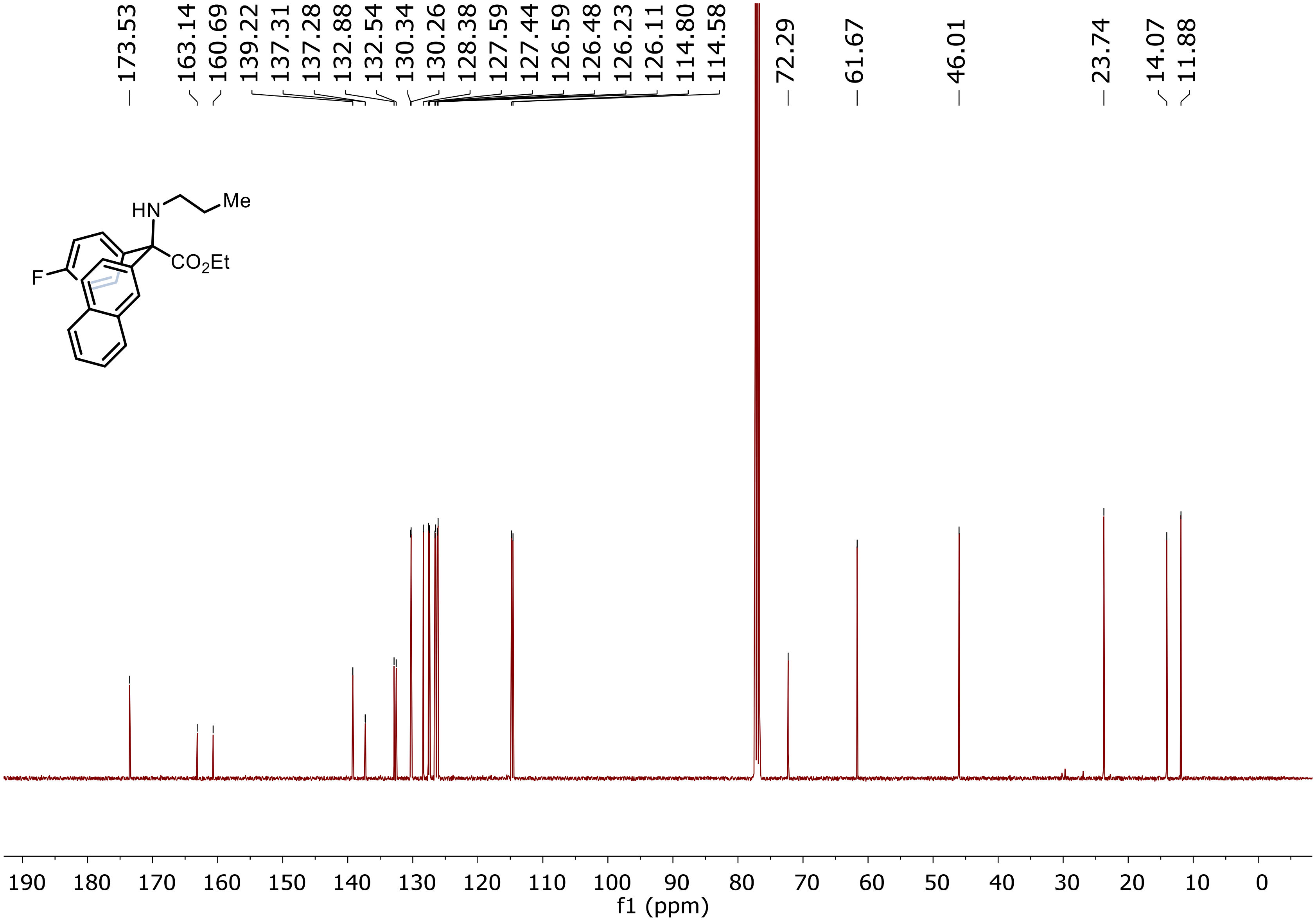

**Ethyl 2-(4-fluorophenyl)-2-(naphthalen-2-yl)-2-(propylamino)acetate (36) - $^{19}$F NMR (376 MHz, $CDCl_3$):**

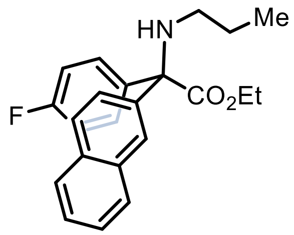


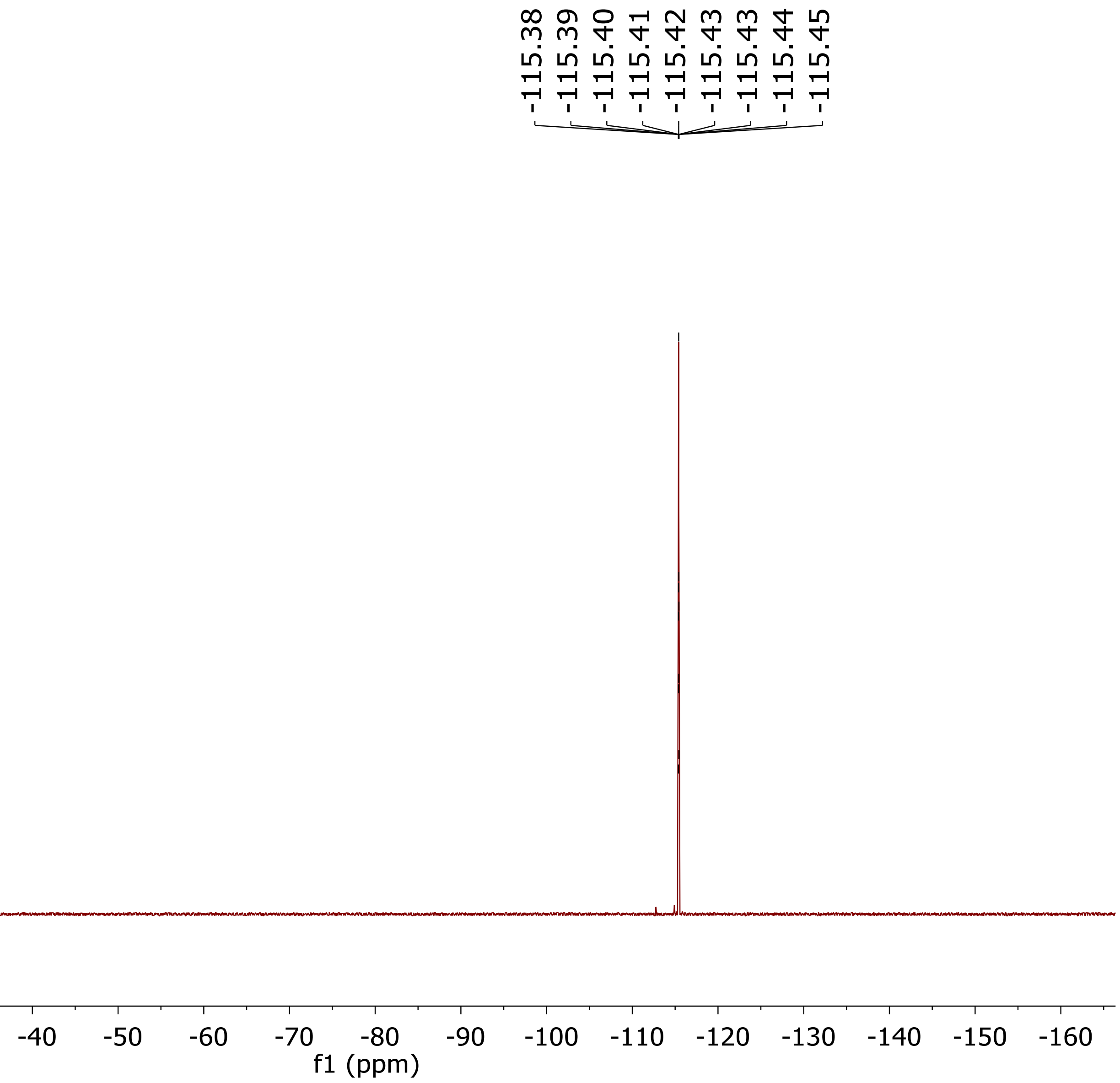

**Ethyl 2-(4-fluorophenyl)-2-(4-methoxyphenyl)-2-(propylamino)acetate (37) - $^1$H NMR (500 MHz, $CDCl_3$):**

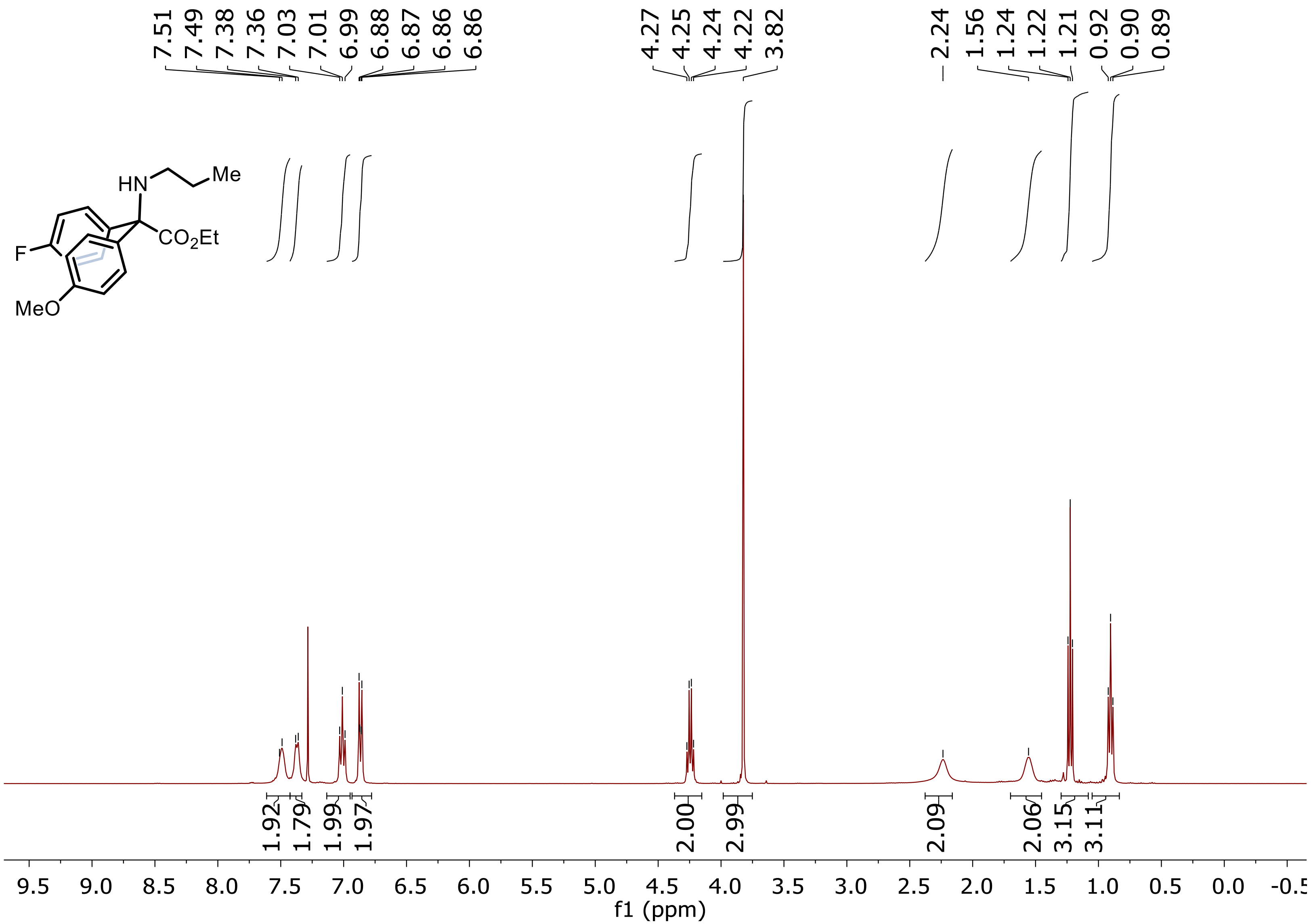

**Ethyl 2-(4-fluorophenyl)-2-(4-methoxyphenyl)-2-(propylamino)acetate (37) - $^{13}C\{^1H\}$ NMR (126 MHz, $CDCl_3$):**

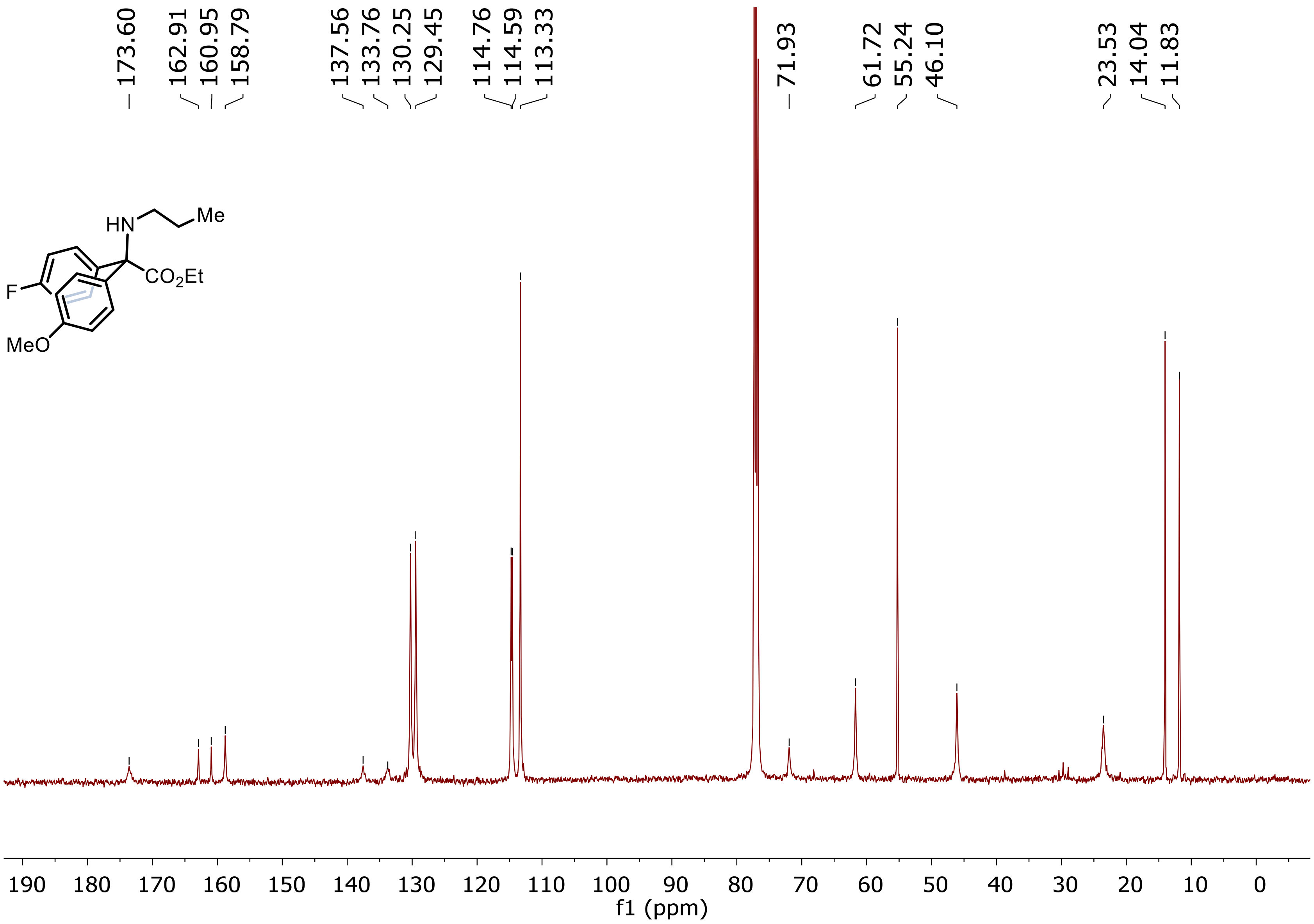

**Ethyl 2-(4-fluorophenyl)-2-(4-methoxyphenyl)-2-(propylamino)acetate (37) - $^{19}$F NMR (376 MHz, $CDCl_3$):**

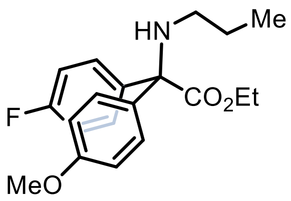

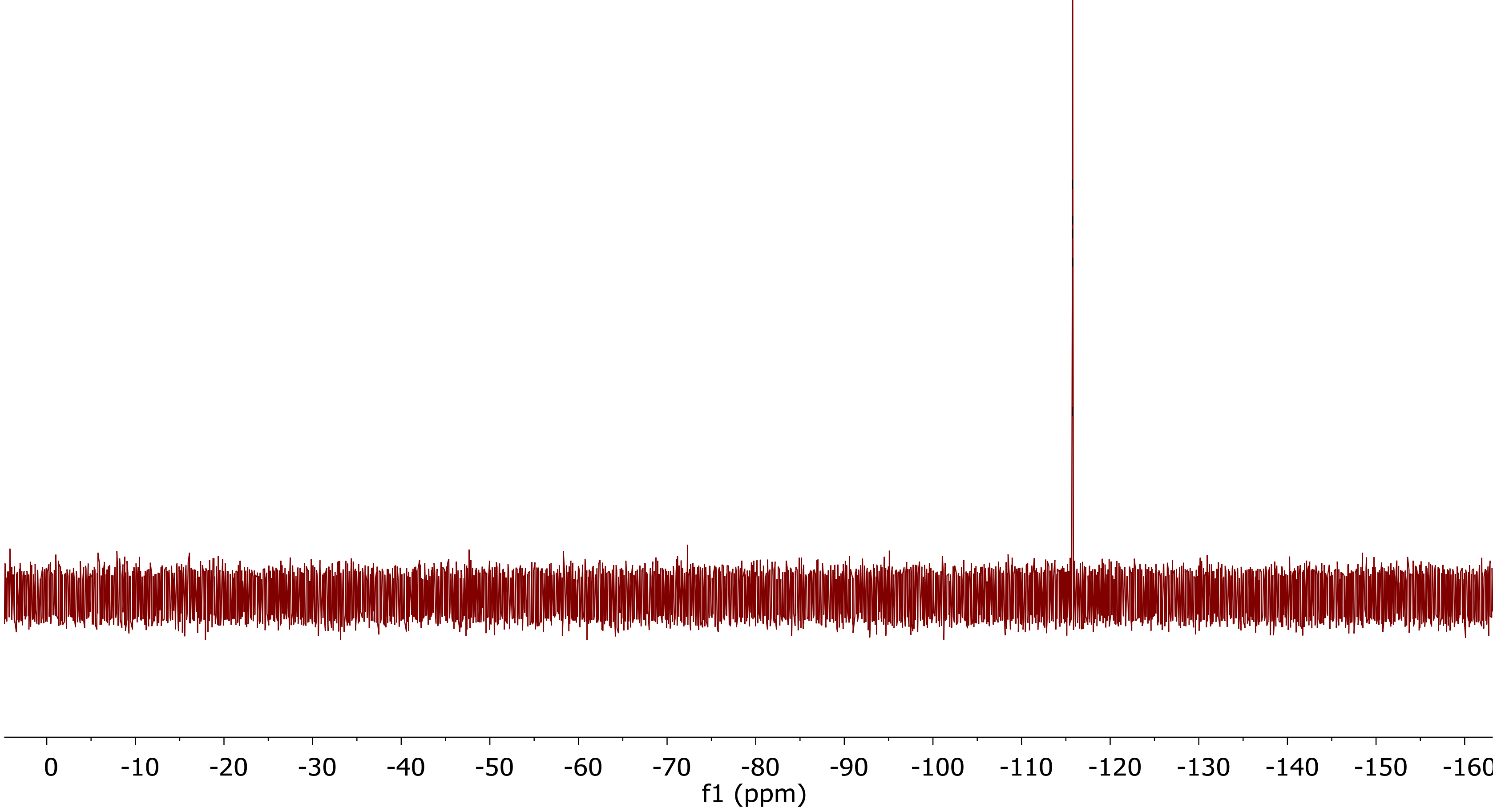

**Ethyl 2-amino-2,2-bis(4-fluorophenyl)acetate (38) - $^{1}$H NMR (400 MHz, $CDCl_3$):**

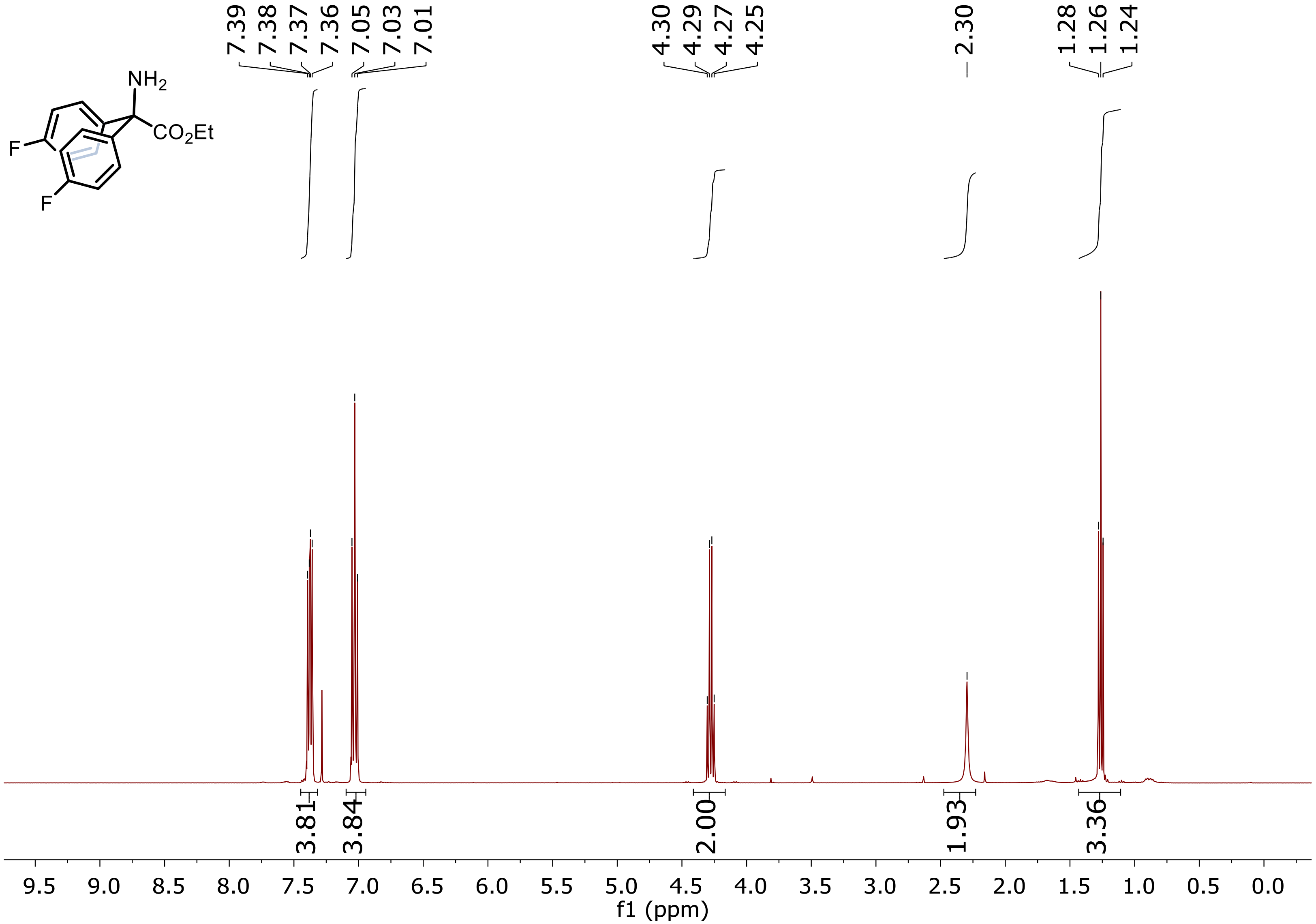

**Ethyl 2-amino-2,2-bis(4-fluorophenyl)acetate (38) - $^{13}C\{^1H\}$ NMR (101 MHz, $CDCl_3$):**

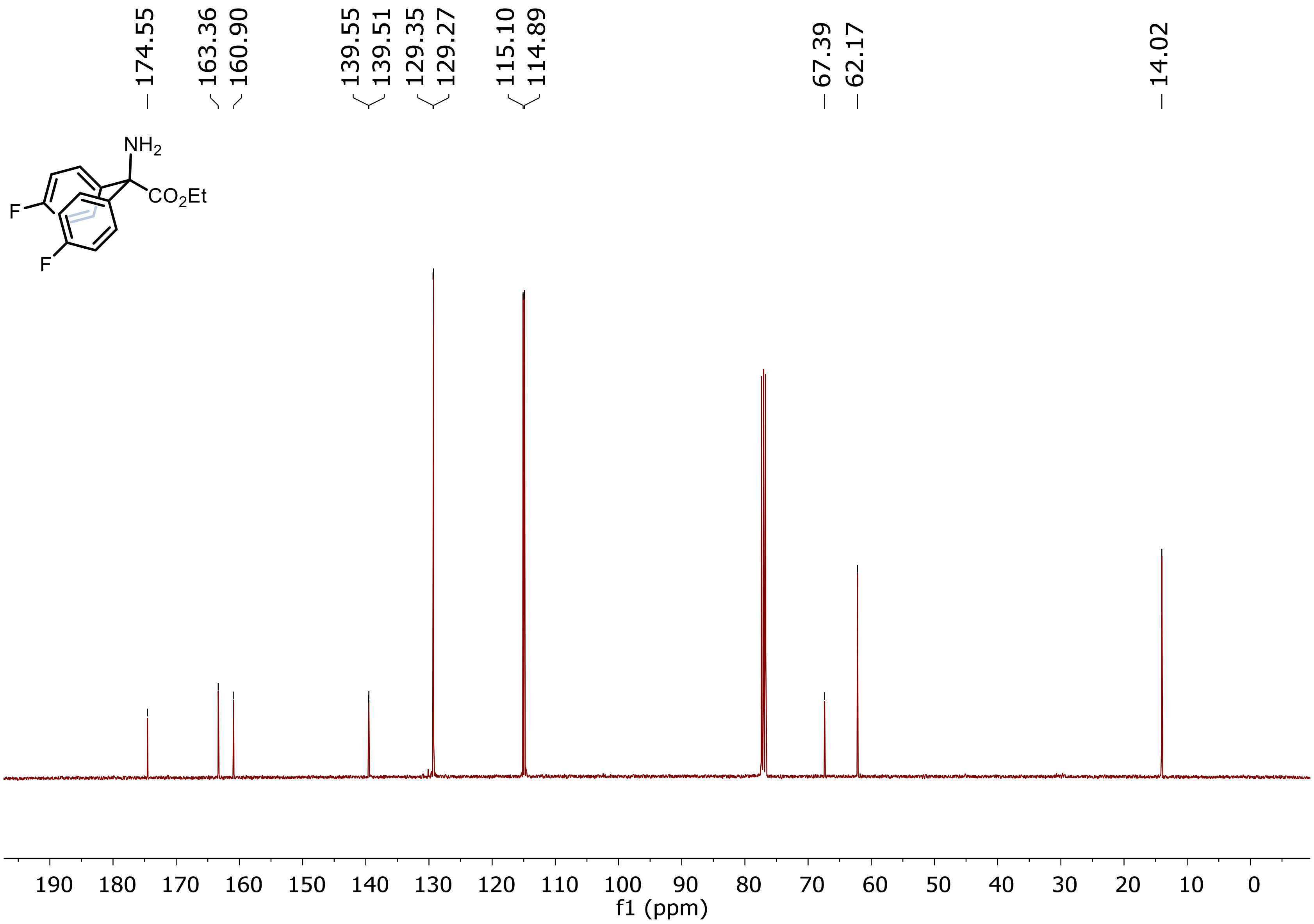

**Ethyl 2-amino-2,2-bis(4-fluorophenyl)acetate (38) - $^{19}$F NMR (376 MHz, $CDCl_3$):**

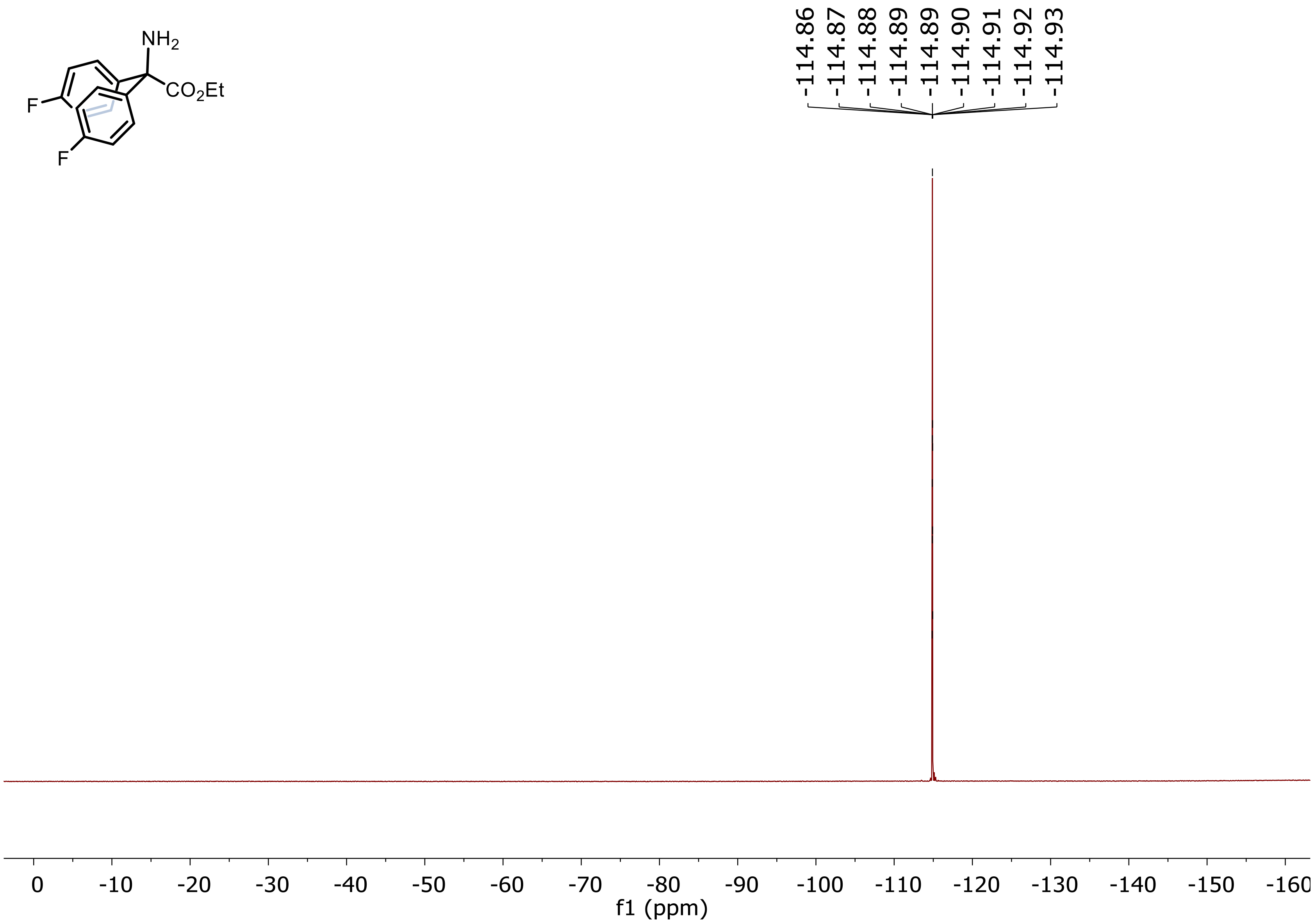

# 8. References

(1) Kastl, R.; Wennemers, H. Peptide-Catalyzed Stereoselective Conjugate Addition Reactions Generating All-Carbon Quaternary Stereogenic Centers. *Angew. Chem. Int. Ed.* **2013**, *52*, 7228–7232. https://doi.org/10.1002/anie.201301583.

(2) Nassar, Y.; Piva, O. A Short Route to Access Oxaspiro[n,3,3]Propellanes. *Org. Biomol. Chem.* **2020**, *18* (30), 5811–5815. https://doi.org/10.1039/D0OB01169J.

(3) Ludwig, C.; Dolny, M.; Götze, H.-J. Fourier-Transform Raman and Infrared Spectra and Normal Coordinate Analysis of the Triphenyl Compounds and Their Methyl-, Methoxy- and Fluoro-Substituted Derivatives of Arsenic, Antimony and Bismuth. *Spectrochim. Acta Part A Mol. Biomol. Spectrosc.* **1997**, *53* (13), 2363–2372. https://doi.org/https://doi.org/10.1016/S1386-1425(97)00176-5.

(4) Wada, M.; Natsume, S.; Suzuki, S.; Akira, U.; Nakamura, M.; Hayase, S.; Erabi, T. Two-Step Synthesis of Triarylmetals (As, Sb, Bi) Starting from the Metal Oxides and 2,6-Dimethoxybenzenethiol. *J. Organomet. Chem.* **1997**, *548* (2), 223–227. https://doi.org/https://doi.org/10.1016/S0022-328X(97)00421-X.

(5) Rudolph, K.; Wieber, M. Diorganobismutacetylide / Diorganobismuthacetylides. *Zeitschrift für Naturforsch. B* **1991**, *46* (10), 1319–1322. https://doi.org/doi:10.1515/znb-1991-1007.

(6) Preda, A.-M.; Krasowska, M.; Wrobel, L.; Kitschke, P.; Andrews, P. C.; MacLellan, J. G.; Mertens, L.; Korb, M.; Rüffer, T.; Lang, H.; Auer, A. A.; Mehring, M. Evaluation of Dispersion Type Metal···π Arene Interaction in Arylbismuth Compounds – an Experimental and Theoretical Study. *Beilstein J. Org. Chem.* **2018**, *14*, 2125–2145. https://doi.org/10.3762/bjoc.14.187.

(7) Jadhav, B. D.; Pardeshi, S. K. Synthesis and Catalytic Application of Pd Complex Catalysts: Atom-Efficient Cross-Coupling of Triarylbismuthines with Haloarenes and Acid Chlorides under Mild Conditions. *Appl. Organomet. Chem.* **2017**, *31* (4), e3591. https://doi.org/https://doi.org/10.1002/aoc.3591.

(8) Rahman, A. F. M. M.; Murafuji, T.; Ishibashi, M.; Miyoshi, Y.; Sugihara, Y. Effect of π-Accepting Substituent on the Reactivity and Spectroscopic Characteristics of

Triarylbismuthanes and Triarylbismuth Dihalides. *J. Organomet. Chem.* **2004**, *689* (21), 3395–3401. https://doi.org/https://doi.org/10.1016/j.jorganchem.2004.07.055.

(9) Matano, Y. Synthesis, Structure, and Reactions of Triaryl(Methyl)Bismuthonium Salts. *Organometallics* **2000**, *19* (12), 2258–2263. https://doi.org/10.1021/om000095d.

(10) Matano, Y.; Suzuki, T.; Iwata, T.; Shinokura, T.; Imahori, H. Remarkable Substituent Effects on the Oxidizing Ability of Tetraarylbismuthonium Tetrafluoroborates in Alcohol Oxidation. *Bull. Chem. Soc. Jpn.* **2008**, *81* (12), 1621–1628. https://doi.org/10.1246/bcsj.81.1621.

(11) Meng, Q.; Sun, Y.; Ratovelomanana-Vidal, V.; Genêt, J. P.; Zhang, Z. CeCl3·7H2O: An Effective Additive in Ru-Catalyzed Enantioselective Hydrogenation of Aromatic α-Ketoesters. *J. Org. Chem.* **2008**, *73* (10), 3842–3847. https://doi.org/10.1021/jo800228e.

(12) Ruan, M.; Chen, L.; Wen, Z.; Yang, F.; Ma, C.; Lu, C.; Yang, G.; Gao, M. Electrochemical Two-Electron Oxygen Reduction Reaction (ORR) Induced Aerobic Oxidation of α-Diazoesters. *Chem. Commun.* **2022**, *58* (13), 2168–2171. https://doi.org/10.1039/D1CC06945D.

(13) Bercot, E. A.; Rovis, T. Highly Efficient Nickel-Catalyzed Cross-Coupling of Succinic and Glutaric Anhydrides with Organozinc Reagents. *J. Am. Chem. Soc.* **2005**, *127* (1), 247–254. https://doi.org/10.1021/ja044588b.

(14) Garro-Helion, F.; Merzouk, A.; Guibe, F. Mild and Selective Palladium(0)-Catalyzed Deallylation of Allylic Amines. Allylamine and Diallylamine as Very Convenient Ammonia Equivalents for the Synthesis of Primary Amines. *J. Org. Chem.* **1993**, *58* (22), 6109–6113. https://doi.org/10.1021/jo00074a044.
