# Supplementary material for: Modular Synthesis of α,α-Diaryl α-Amino Esters via Bi(V)-Mediated Arylation/SN2-Displacement of Kukhtin–Ramirez Intermediates
Source: Org Lett. 2022 Oct 24;24(43):8002–7. doi: 10.1021/acs.orglett.2c03201 (PMC9641671; doi:10.1021/acs.orglett.2c03201)
Supplement: Supplementary file 2 — ol2c03201_si_002.zip [file ol2c03201_si_002.zip › FID_3a-d/3a/3a_19F/2/pdata/1/pcxac8.EJ003_conc_2_1.pdf]

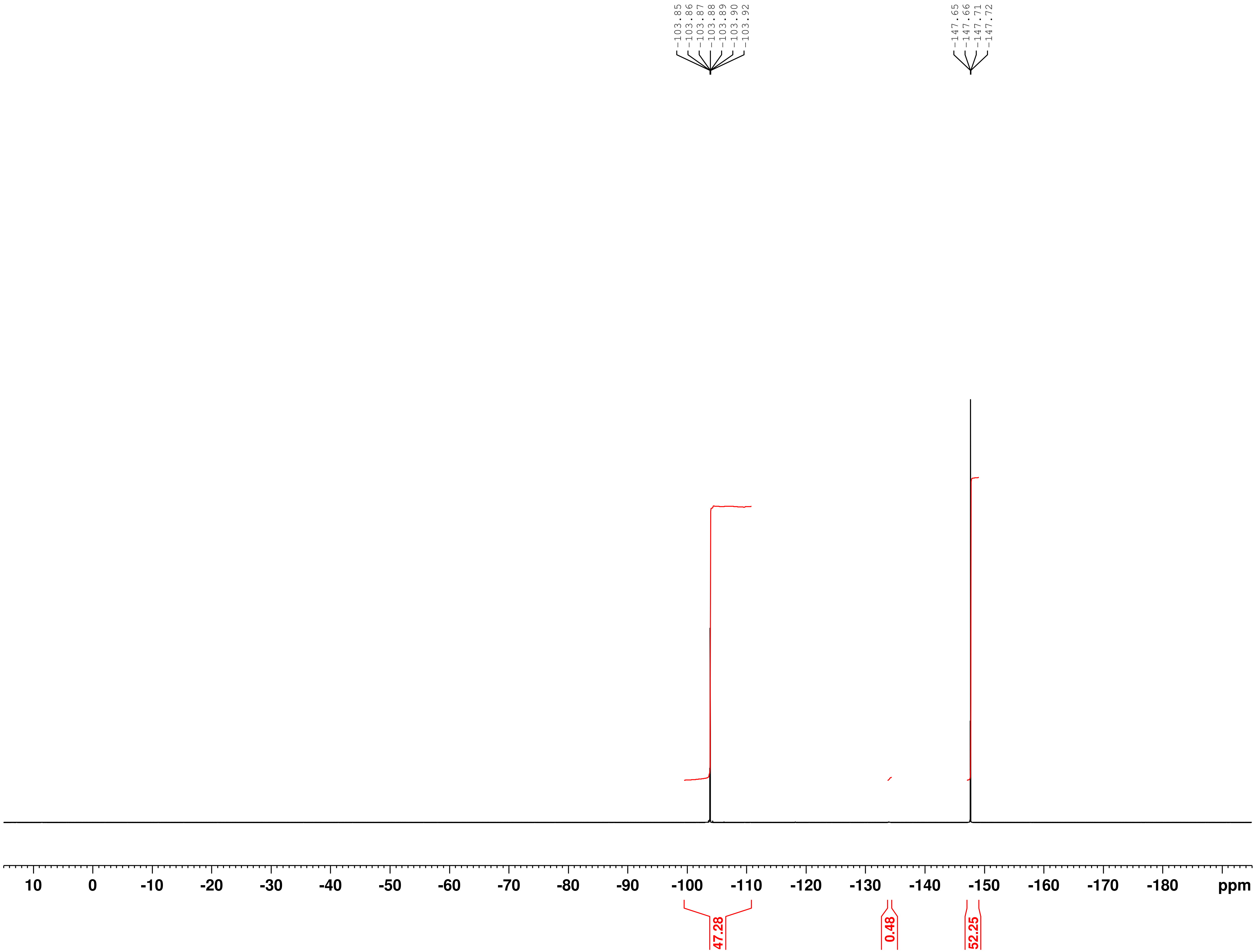


```
Current Data Parameters
NAME     pcxac8.EJ003_conc
EXPNO                    2
PROCNO                   1

F2 - Acquisition Parameters
Date_           20211116
Time               14.41 h
INSTRUM          av3400hd
PROBHD   Z122623_0053 (
PULPROG           zgflqn
TD                261896
SOLVENT            CDCl3
NS                    16
DS                     4
SWH            85227.273 Hz
FIDRES          0.650848 Hz
AQ             1.5364566 sec
RG                 12.56
DW                 5.867 usec
DE                 18.56 usec
TE                 298.0 K
D1            1.50000000 sec
TD0                    1
SFO1         376.5303412 MHz
NUC1                 19F
P1                 13.80 usec
PLW1          8.69999981 W

F2 - Processing parameters
SI                262144
SF           376.5642320 MHz
WDW                   EM
SSB      0
LB                  1.00 Hz
GB       0
PC                  1.00
```
