# Supplementary material for: Modular Synthesis of α,α-Diaryl α-Amino Esters via Bi(V)-Mediated Arylation/SN2-Displacement of Kukhtin–Ramirez Intermediates
Source: Org Lett. 2022 Oct 24;24(43):8002–7. doi: 10.1021/acs.orglett.2c03201 (PMC9641671; doi:10.1021/acs.orglett.2c03201)
Supplement: Supplementary file 2 — ol2c03201_si_002.zip [file ol2c03201_si_002.zip › FID_3a-d/3a/3a_1H/pdata/1/email_pcxac8.AC_pFBi+_1_1.pdf]

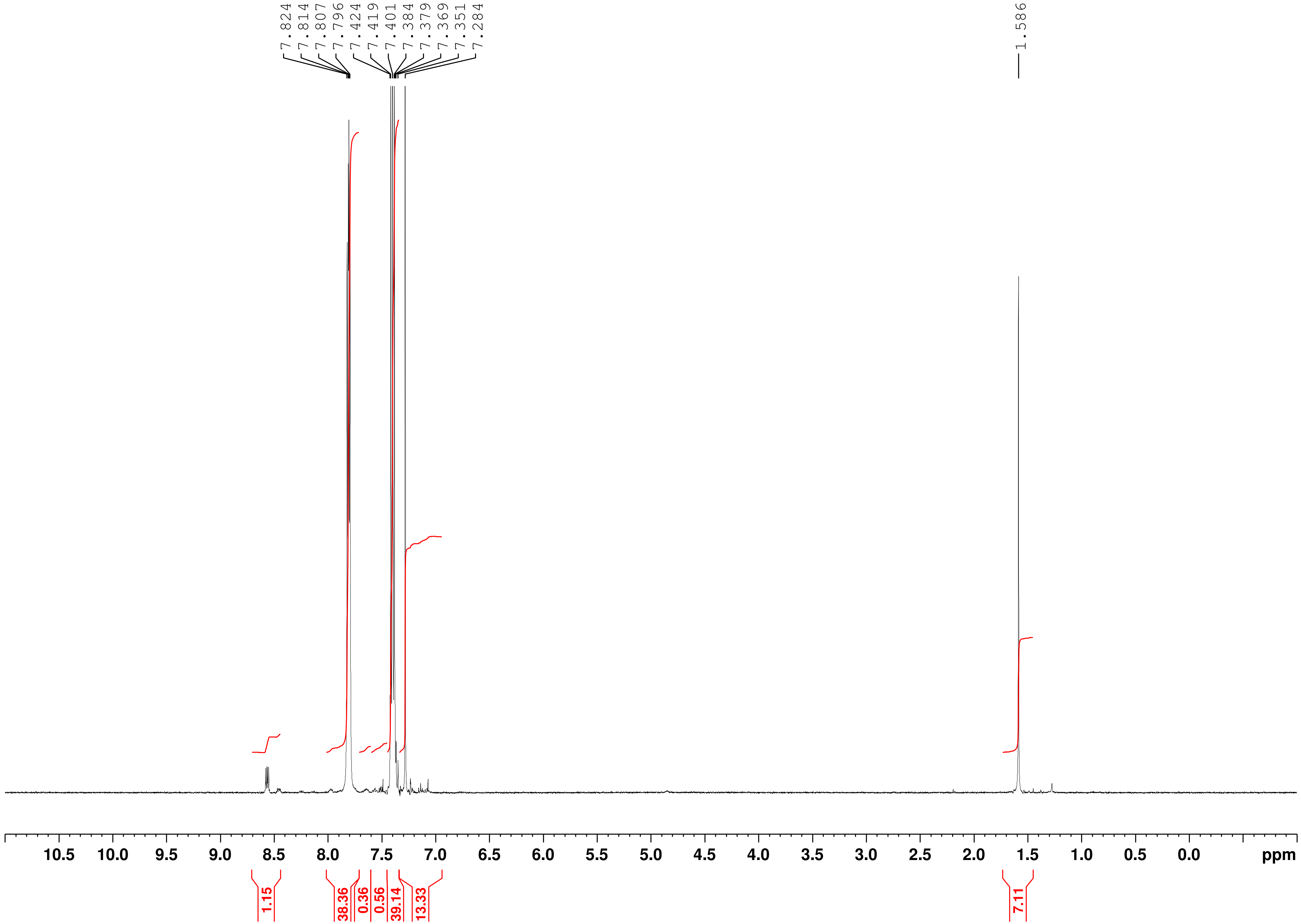


```
Current Data Parameters
NAME      pcxac8.AC_pFBi+
EXPNO                   1
PROCNO                  1

F2 - Acquisition Parameters
Date_            20220608
Time                14.10 h
INSTRUM            av3500
PROBHD   Z8007_0120 (PH
PULPROG              zg30
TD                  65536
SOLVENT             CDCl3
NS                     16
DS                      2
SWH             10288.065 Hz
FIDRES           0.313967 Hz
AQ              3.1850495 sec
RG                    362
DW                 48.600 usec
DE                   7.20 usec
TE                  298.0 K
D1             1.00000000 sec
TD0                     1
SFO1          500.1330885 MHz
NUC1                   1H
P1                  10.00 usec
PLW1          29.99200058 W

F2 - Processing parameters
SI                  65536
SF            500.1300000 MHz
WDW                    EM
SSB       0
LB                   0.30 Hz
GB        0
PC                   1.00
```
