# Supplementary material for: Modular Synthesis of α,α-Diaryl α-Amino Esters via Bi(V)-Mediated Arylation/SN2-Displacement of Kukhtin–Ramirez Intermediates
Source: Org Lett. 2022 Oct 24;24(43):8002–7. doi: 10.1021/acs.orglett.2c03201 (PMC9641671; doi:10.1021/acs.orglett.2c03201)
Supplement: Supplementary file 2 — ol2c03201_si_002.zip [file ol2c03201_si_002.zip › FID_3a-d/3b/3b_1H/pdata/1/pcxac8.AC341_trit_1_1.pdf]

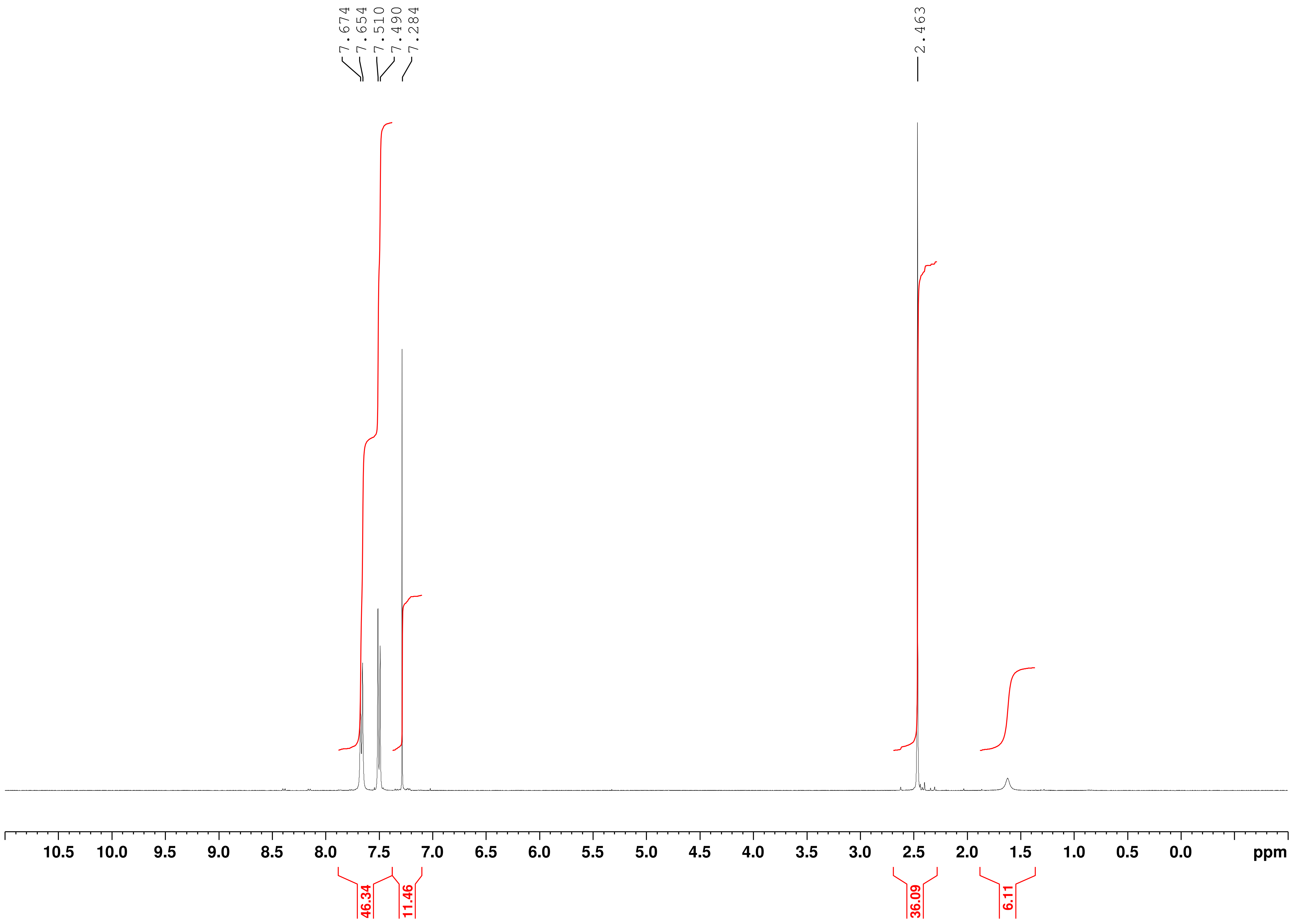


```
Current Data Parameters
NAME       pcxac8.AC341_trit
EXPNO                      1
PROCNO                     1

F2 - Acquisition Parameters
Date_               20220504
Time                   13.21 h
INSTRUM               av3400
PROBHD      Z104450_0225 (
PULPROG                 zg30
TD                     65536
SOLVENT                CDCl3
NS                        16
DS                         2
SWH                 8223.685 Hz
FIDRES              0.250967 Hz
AQ                 3.9845889 sec
RG                       512
DW                    60.800 usec
DE                     16.03 usec
TE                     298.0 K
D1                1.00000000 sec
TD0                        1
SFO1             400.0724706 MHz
NUC1                      1H
P1                     14.00 usec
PLW1             11.92800045 W

F2 - Processing parameters
SI                     65536
SF               400.0700000 MHz
WDW                       EM
SSB         0
LB                      0.30 Hz
GB          0
PC                      1.00
```
