# Supplementary material for: Modular Synthesis of α,α-Diaryl α-Amino Esters via Bi(V)-Mediated Arylation/SN2-Displacement of Kukhtin–Ramirez Intermediates
Source: Org Lett. 2022 Oct 24;24(43):8002–7. doi: 10.1021/acs.orglett.2c03201 (PMC9641671; doi:10.1021/acs.orglett.2c03201)
Supplement: Supplementary file 3 — ol2c03201_si_003.zip [file ol2c03201_si_003.zip › FID_8-12/10/10_1H/pdata/1/pcxac8.AC269_dryproduct_2_1.pdf]

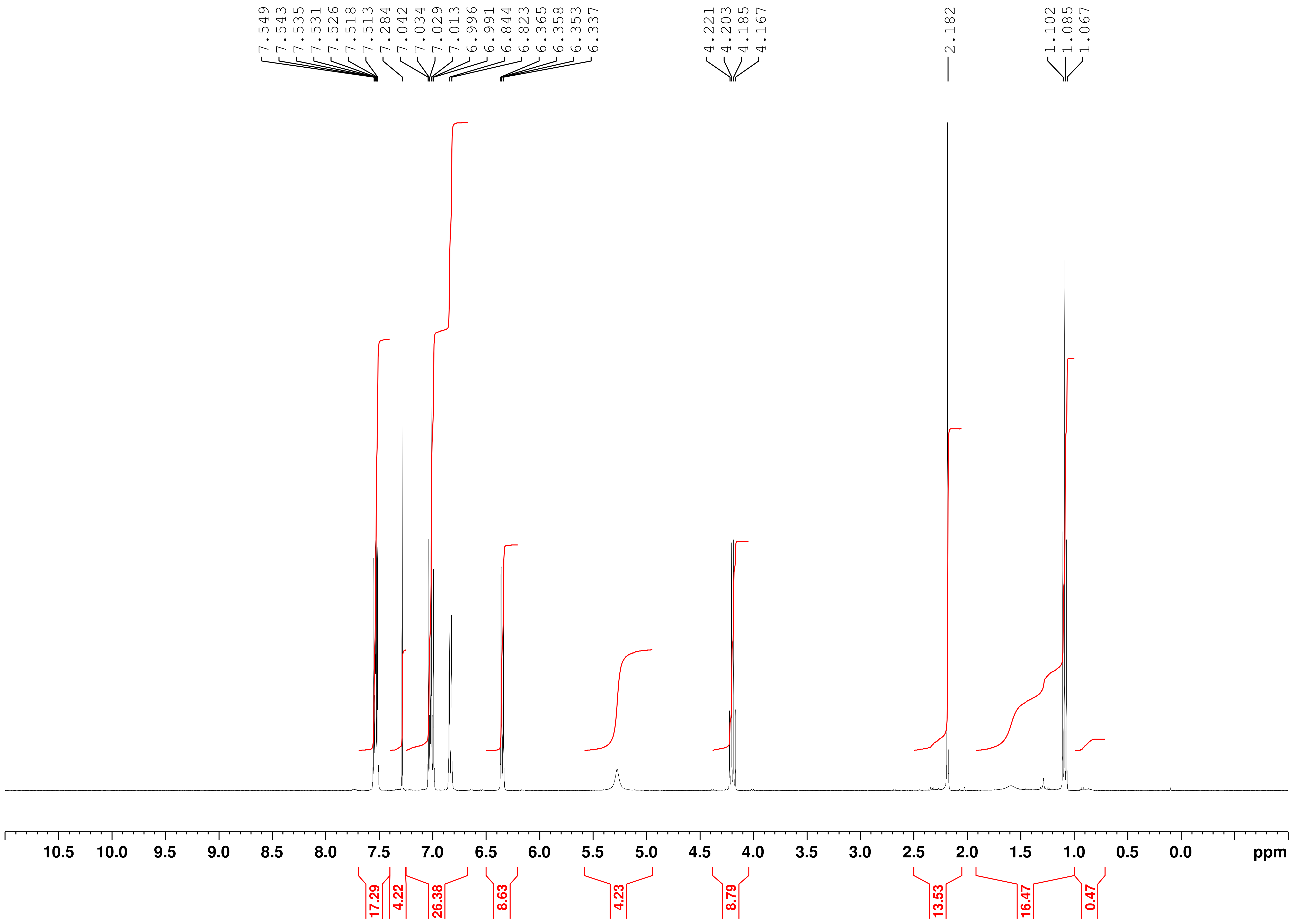


```
Current Data Parameters
NAME      pcxac8.AC269_dryproduct
EXPNO                    2
PROCNO                   1

F2 - Acquisition Parameters
Date_             20220219
Time                  1.13 h
INSTRUM              av3400
PROBHD   Z104450_0225 (
PULPROG               zg30
TD                   65536
SOLVENT              CDCl3
NS                      16
DS                       2
SWH               8223.685 Hz
FIDRES            0.250967 Hz
AQ               3.9845889 sec
RG                     406
DW                  60.800 usec
DE                   16.03 usec
TE                   298.0 K
D1              1.00000000 sec
TD0                      1
SFO1           400.0724706 MHz
NUC1                    1H
P1                   14.00 usec
PLW1           11.92800045 W

F2 - Processing parameters
SI                   65536
SF             400.0700000 MHz
WDW                     EM
SSB      0
LB                    0.30 Hz
GB       0
PC                    1.00
```
