# Supplementary material for: Modular Synthesis of α,α-Diaryl α-Amino Esters via Bi(V)-Mediated Arylation/SN2-Displacement of Kukhtin–Ramirez Intermediates
Source: Org Lett. 2022 Oct 24;24(43):8002–7. doi: 10.1021/acs.orglett.2c03201 (PMC9641671; doi:10.1021/acs.orglett.2c03201)
Supplement: Supplementary file 3 — ol2c03201_si_003.zip [file ol2c03201_si_003.zip › FID_8-12/10/10_COSY/pdata/1/pcxac8.AC269_dryproduct_3_1.pdf]

Double quantum filtered COSY Spectrum.

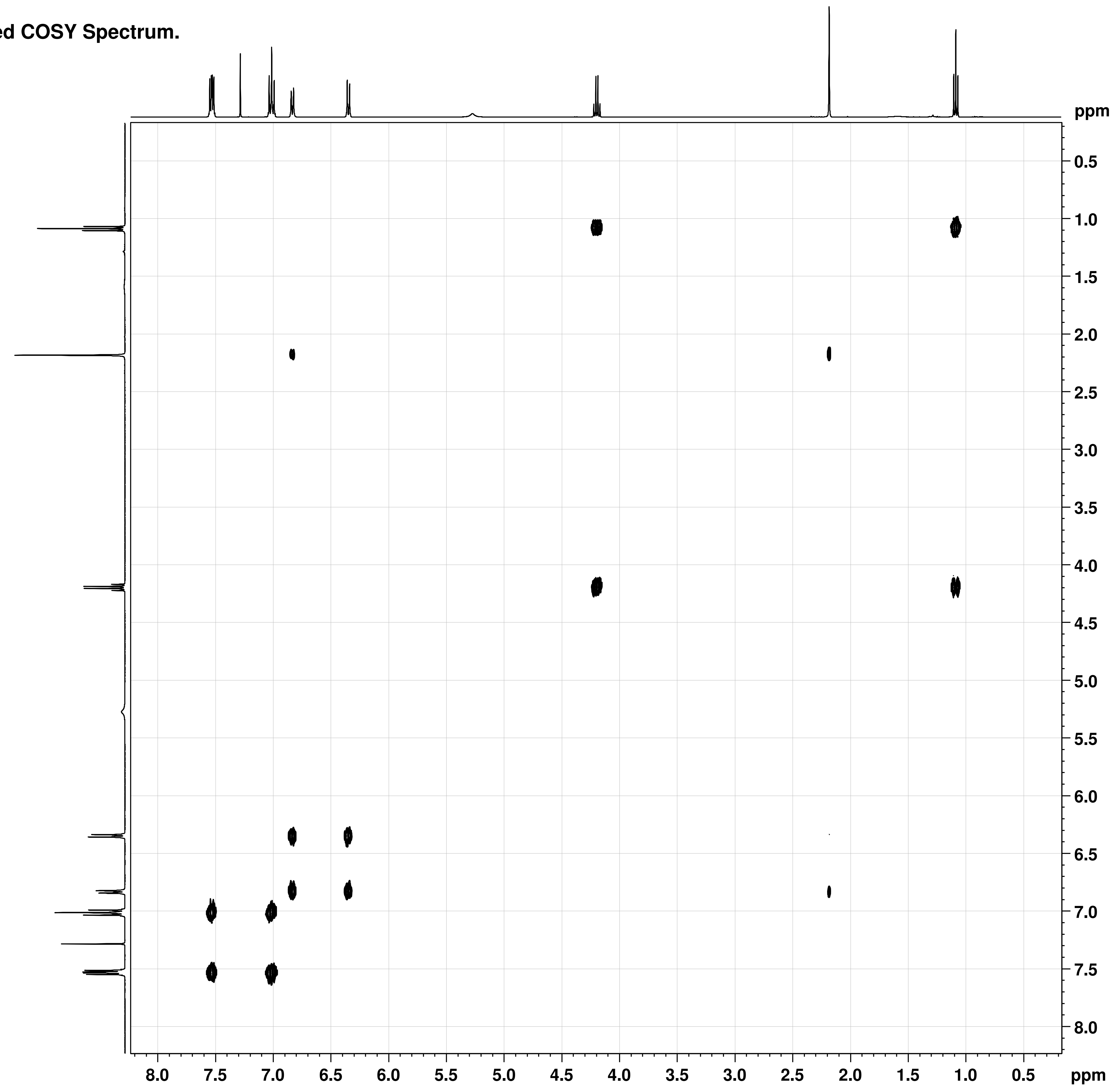


```
Current Data Parameters
NAME      pcxac8.AC269_dryprodu
EXPNO                      3
PROCNO                     1

F2 - Acquisition Parameters
Date_              20220219
Time                 1.14 h
INSTRUM              av3400
PROBHD   Z104450_0225 (
PULPROG    cosygpmfppqf
TD                     2048
SOLVENT               CDCl3
NS                        2
DS                       16
SWH              3556.188 Hz
FIDRES           3.472840 Hz
AQ              0.2879488 sec
RG                     2050
DW                140.600 usec
DE                   9.38 usec
TE                  298.0 K
D0             0.00000300 sec
D1             0.90838599 sec
D11            0.03000000 sec
D12            0.00002000 sec
D13            0.00000400 sec
D16            0.00020000 sec
IN0            0.00028120 sec
TDav                      1
SFO1         400.0716815 MHz
NUC1                     1H
P1                  14.00 usec
P17               2500.00 usec
PLW1          11.92800045 W
PLW10          2.59770012 W
GPNAM[1]       SMSQ10.100
GPZ1                16.00 %
GPNAM[2]       SMSQ10.100
GPZ2                12.00 %
GPNAM[3]       SMSQ10.100
GPZ3                40.00 %
P16               1000.00 usec

F1 - Acquisition parameters
TD                      128
SFO1            400.0717 MHz
FIDRES         55.565434 Hz
SW                 8.889 ppm
FnMODE                   QF

F2 - Processing parameters
SI                     2048
SF           400.0700000 MHz
WDW                    SINE
SSB       0
LB        0 Hz
GB        0
PC                     1.40

F1 - Processing parameters
SI                      512
MC2                      QF
SF           400.0700000 MHz
WDW                    SINE
SSB       0
LB        0 Hz
GB        0
```
