# Supplementary figures and images for: Modular Synthesis of α,α-Diaryl α-Amino Esters via Bi(V)-Mediated Arylation/SN2-Displacement of Kukhtin–Ramirez Intermediates
Source: Org Lett. 2022 Oct 24;24(43):8002–7. doi: 10.1021/acs.orglett.2c03201 (PMC9641671; doi:10.1021/acs.orglett.2c03201)

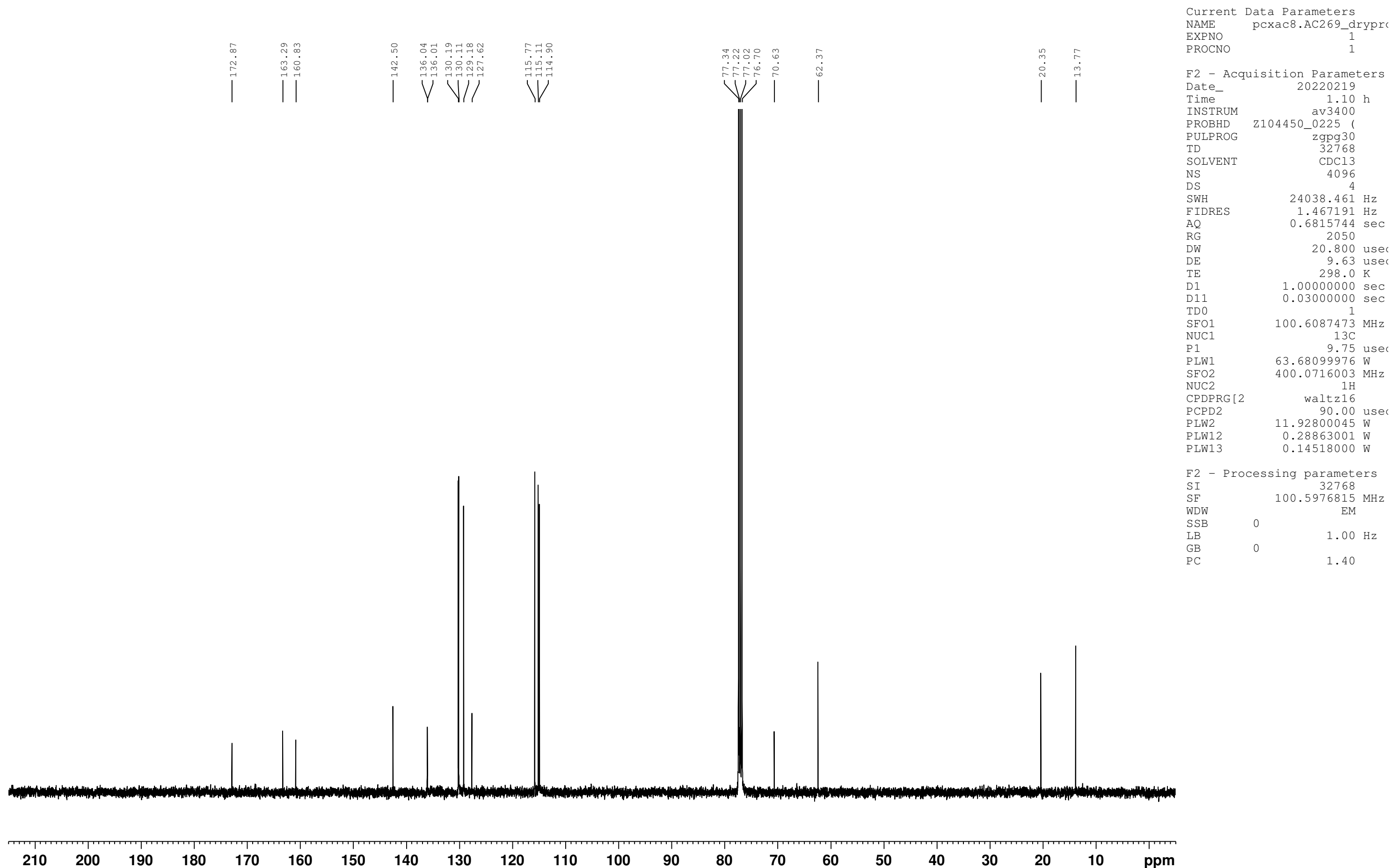

Supplement: Supplementary file 3 — ol2c03201_si_003.zip [file ol2c03201_si_003.zip › FID_8-12/10/10_13C/pdata/1/pcxac8.AC269_dryproduct_1_1.pdf]

HMBC spectrum: very low level one-bond artefacts may be observed.

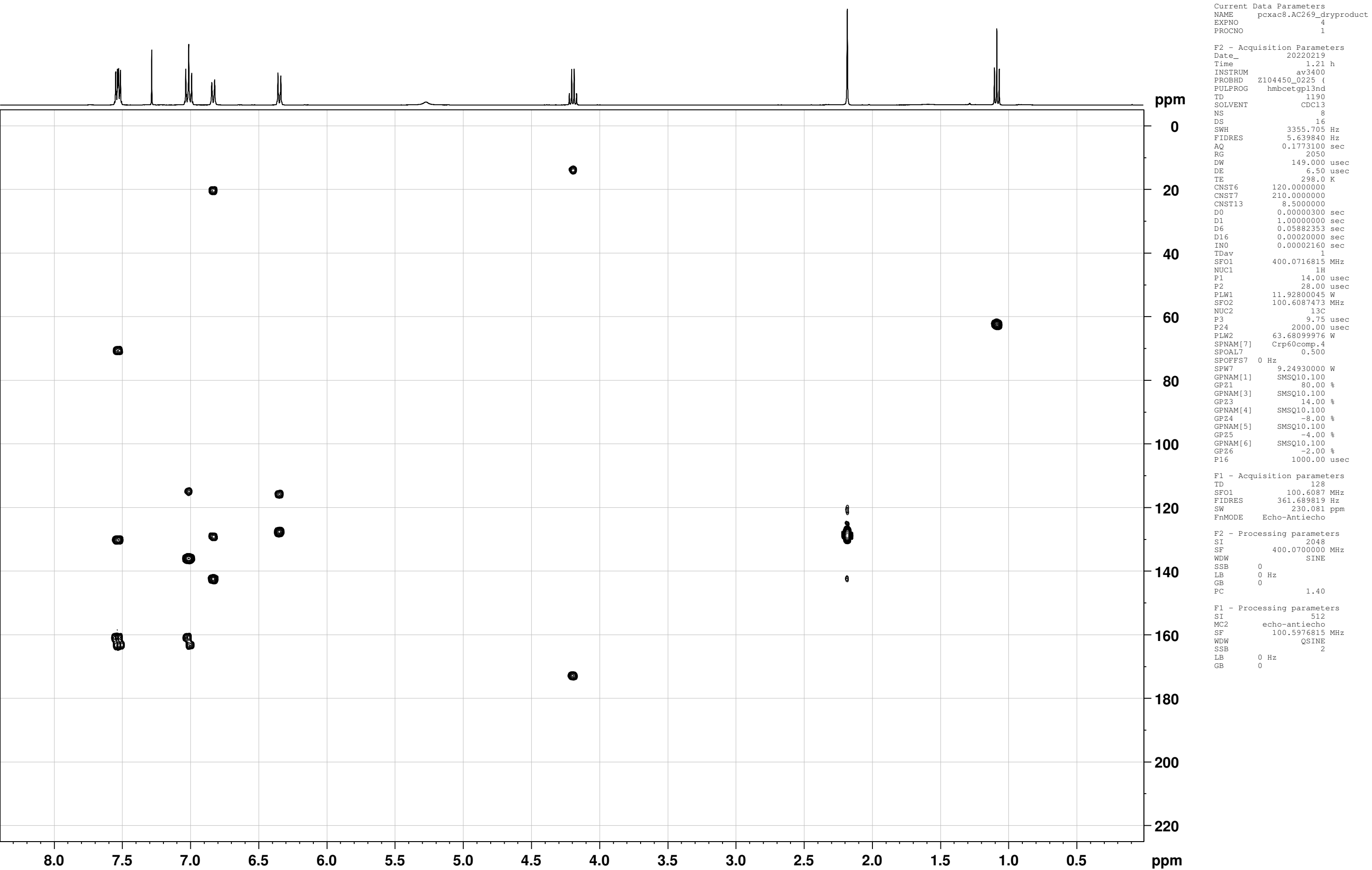

Supplement: Supplementary file 3 — ol2c03201_si_003.zip [file ol2c03201_si_003.zip › FID_8-12/10/10_HMBC/pdata/1/pcxac8.AC269_dryproduct_4_1.pdf]

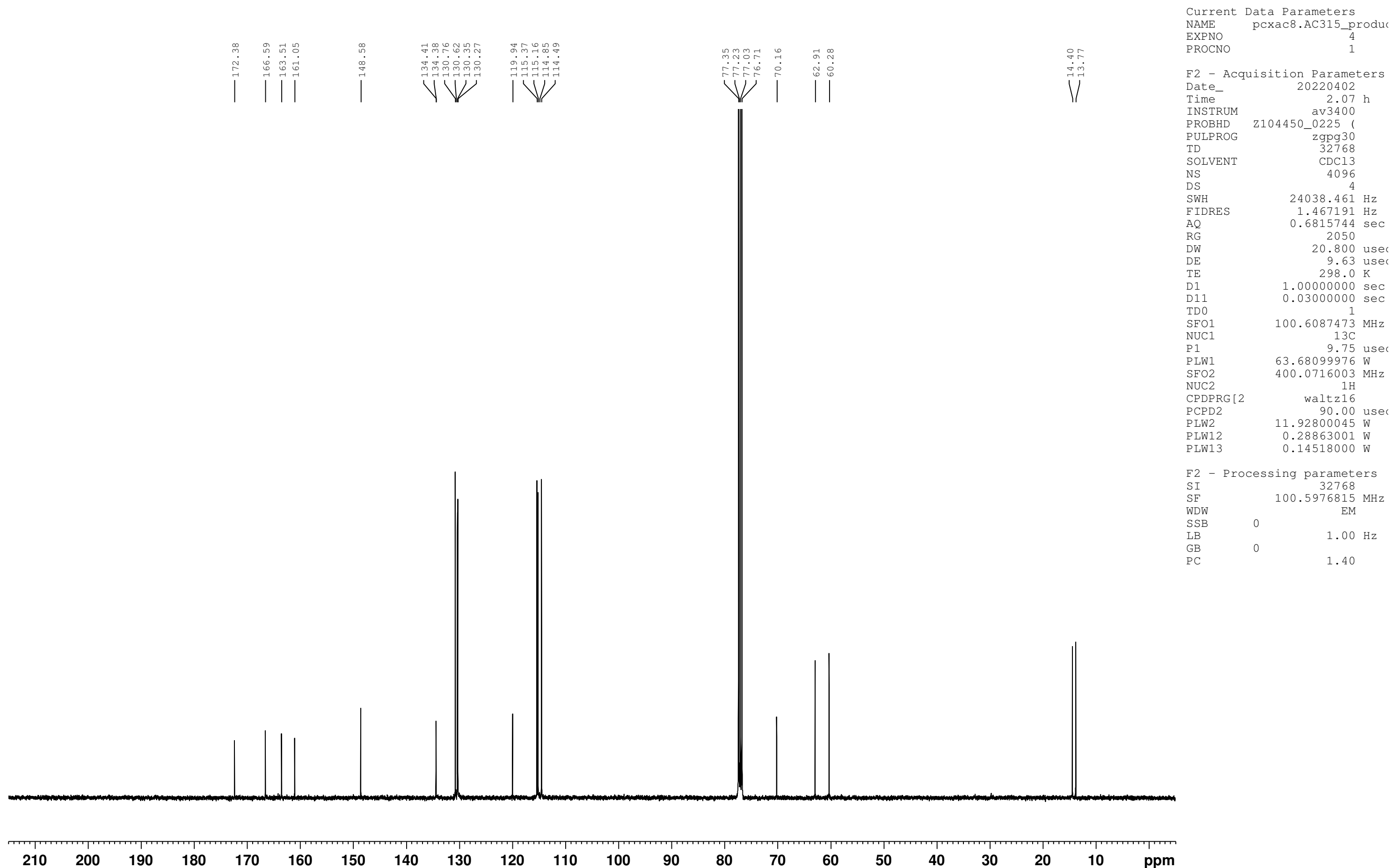

Supplement: Supplementary file 3 — ol2c03201_si_003.zip [file ol2c03201_si_003.zip › FID_8-12/11/11_13C/pdata/1/pcxac8.AC315_productdry_4_1.pdf]

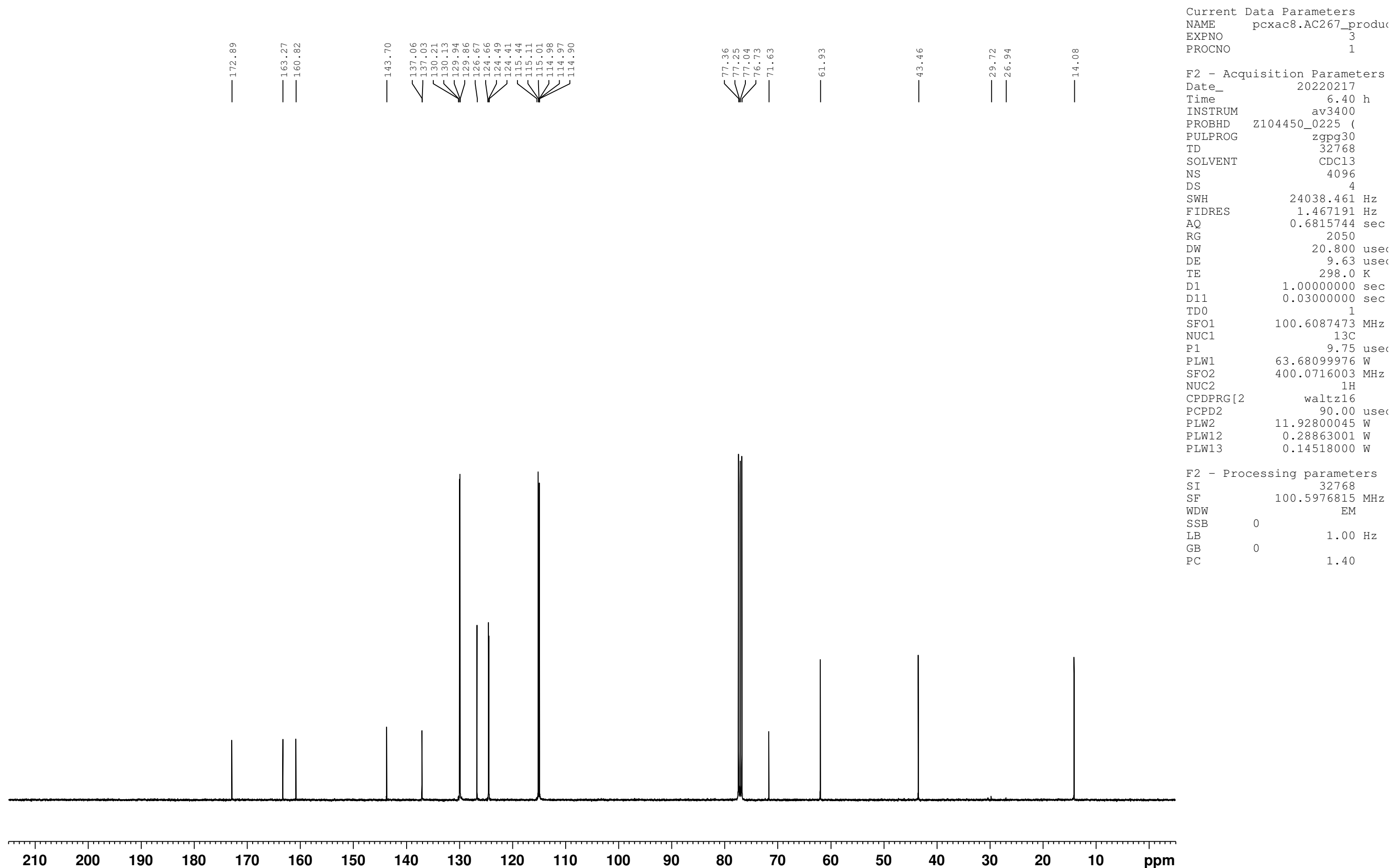

Supplement: Supplementary file 6 — ol2c03201_si_006.zip [file ol2c03201_si_006.zip › FID_23-27/23/23_13C/pdata/1/pcxac8.AC267_product_3_1.pdf]

HMBC spectrum: very low level one-bond artefacts may be observed.

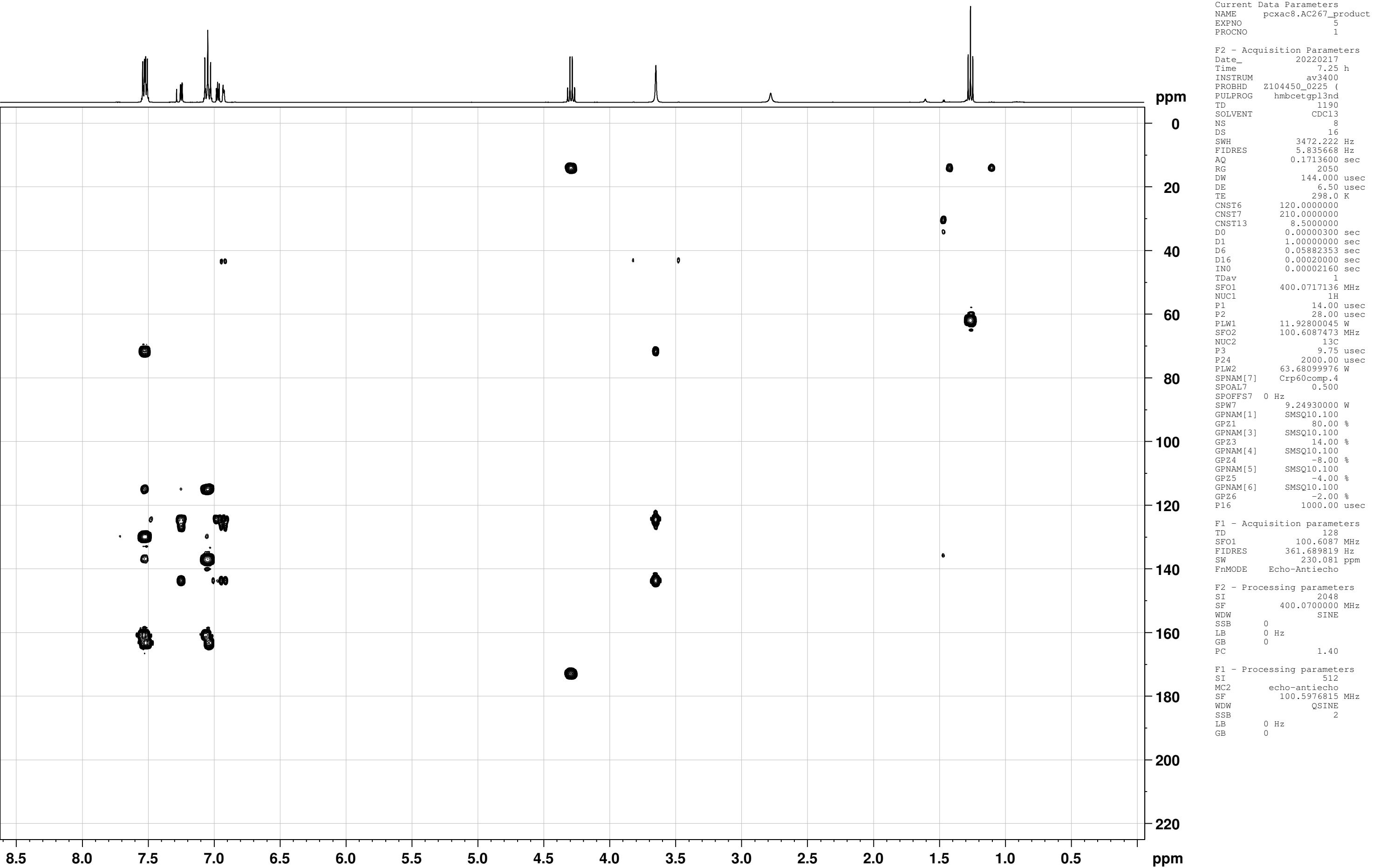

Supplement: Supplementary file 6 — ol2c03201_si_006.zip [file ol2c03201_si_006.zip › FID_23-27/23/23_HMBC/pdata/1/pcxac8.AC267_product_5_1.pdf]

Multiplicity-edited HSQC Spectrum: CH and Me up (black), CH2 down (grey).

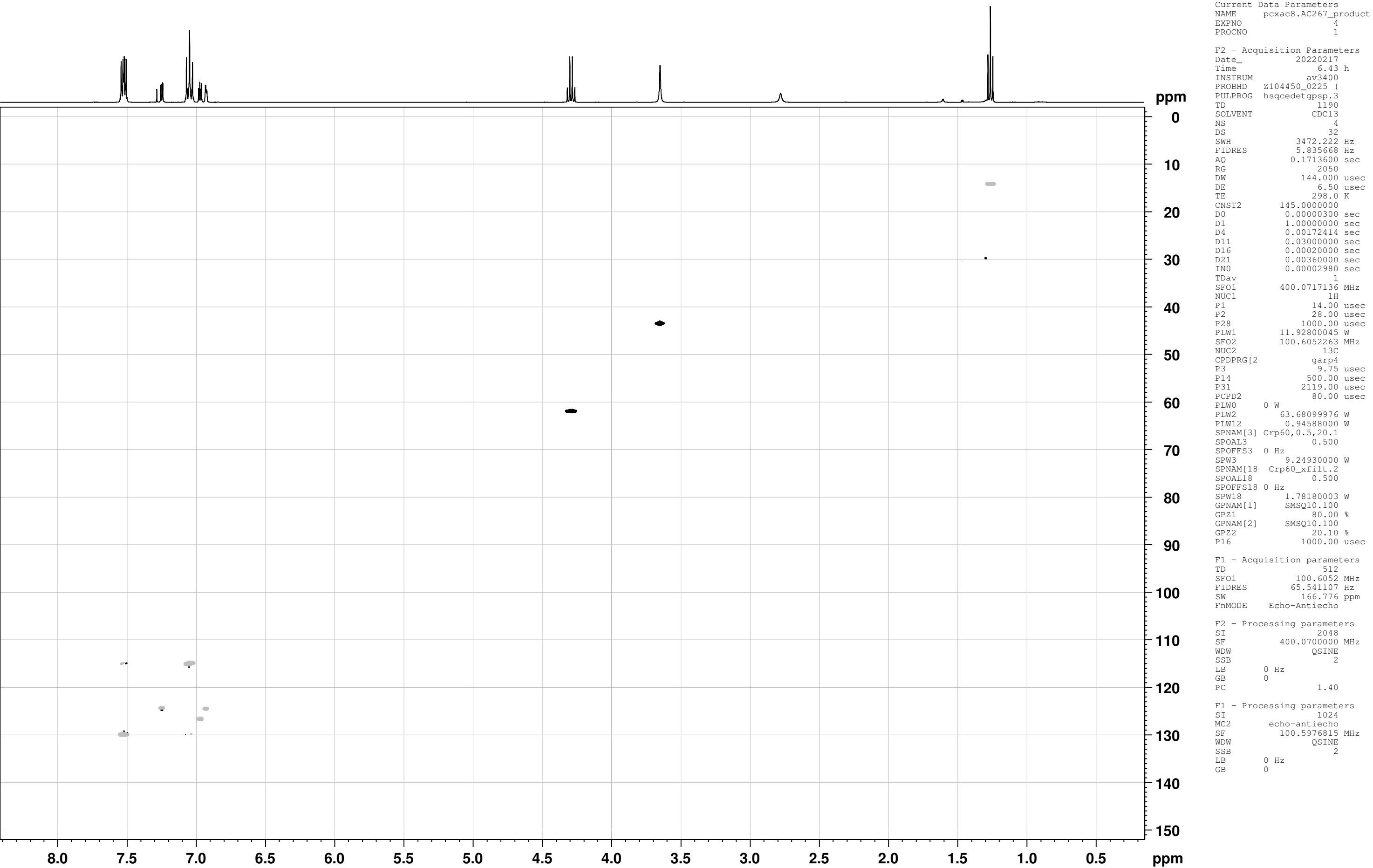

Supplement: Supplementary file 6 — ol2c03201_si_006.zip [file ol2c03201_si_006.zip › FID_23-27/23/23_HSQC/pdata/1/pcxac8.AC267_product_4_1.pdf]

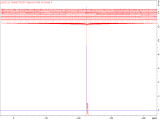

Supplement: Supplementary file 9 — ol2c03201_si_009.zip [file ol2c03201_si_009.zip › FID_Bi-cpds/pF Ar3Bi/19F/pdata/1/thumb.png]

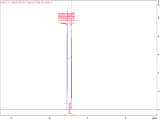

Supplement: Supplementary file 9 — ol2c03201_si_009.zip [file ol2c03201_si_009.zip › FID_Bi-cpds/pF Ar3Bi/1H/pdata/1/thumb.png]

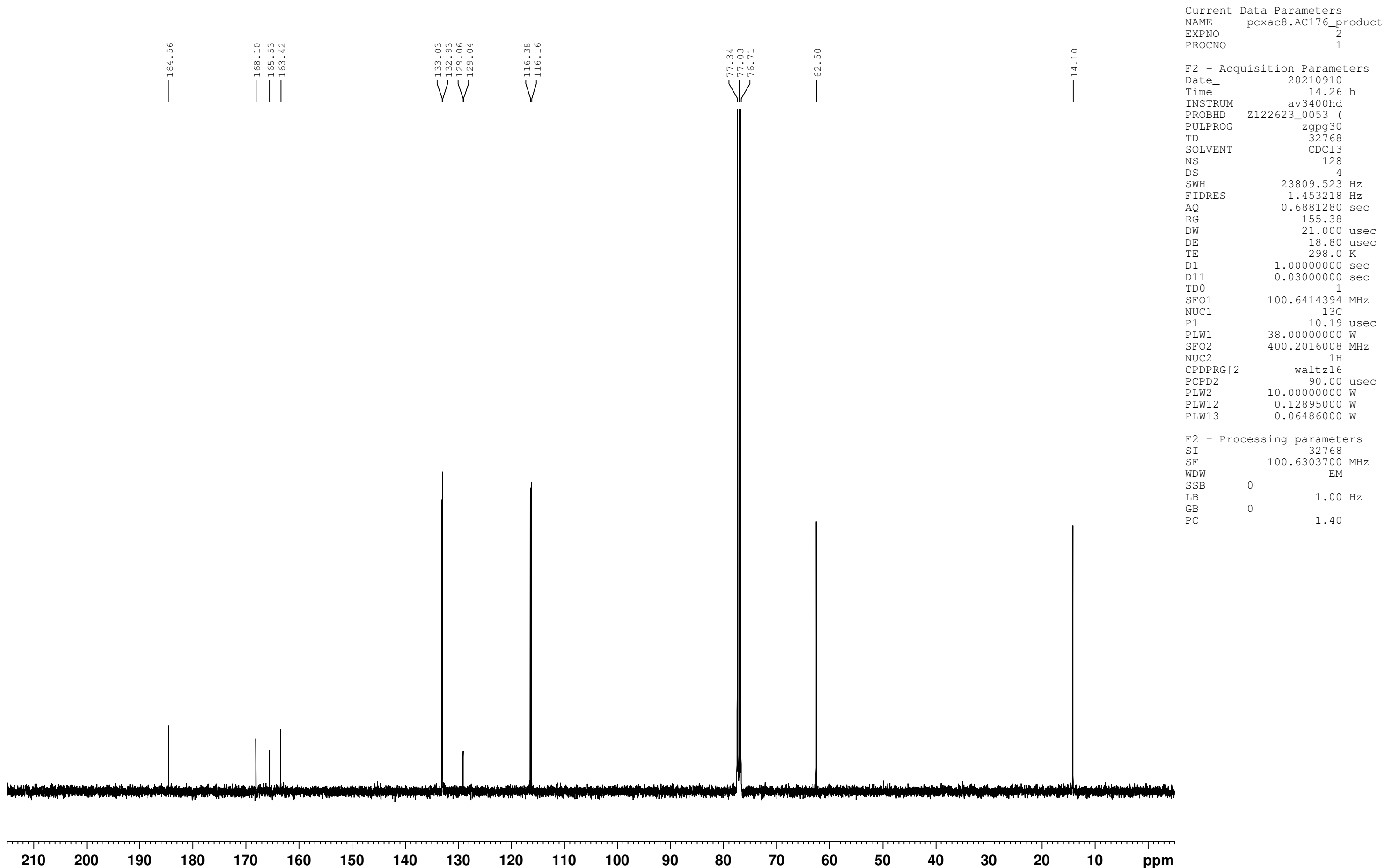

Supplement: Supplementary file 10 — ol2c03201_si_010.zip [file ol2c03201_si_010.zip › FID keto ester/FID keto ester/1a/13C/pdata/1/pcxac8.AC176_product_2_1.pdf]
