# Supplementary material for: Modular Synthesis of α,α-Diaryl α-Amino Esters via Bi(V)-Mediated Arylation/SN2-Displacement of Kukhtin–Ramirez Intermediates
Source: Org Lett. 2022 Oct 24;24(43):8002–7. doi: 10.1021/acs.orglett.2c03201 (PMC9641671; doi:10.1021/acs.orglett.2c03201)
Supplement: Supplementary file 5 — ol2c03201_si_005.zip [file ol2c03201_si_005.zip › FID_18-22/18/18_1H/pdata/1/pcxac8.AC229B_product_1_1.pdf]

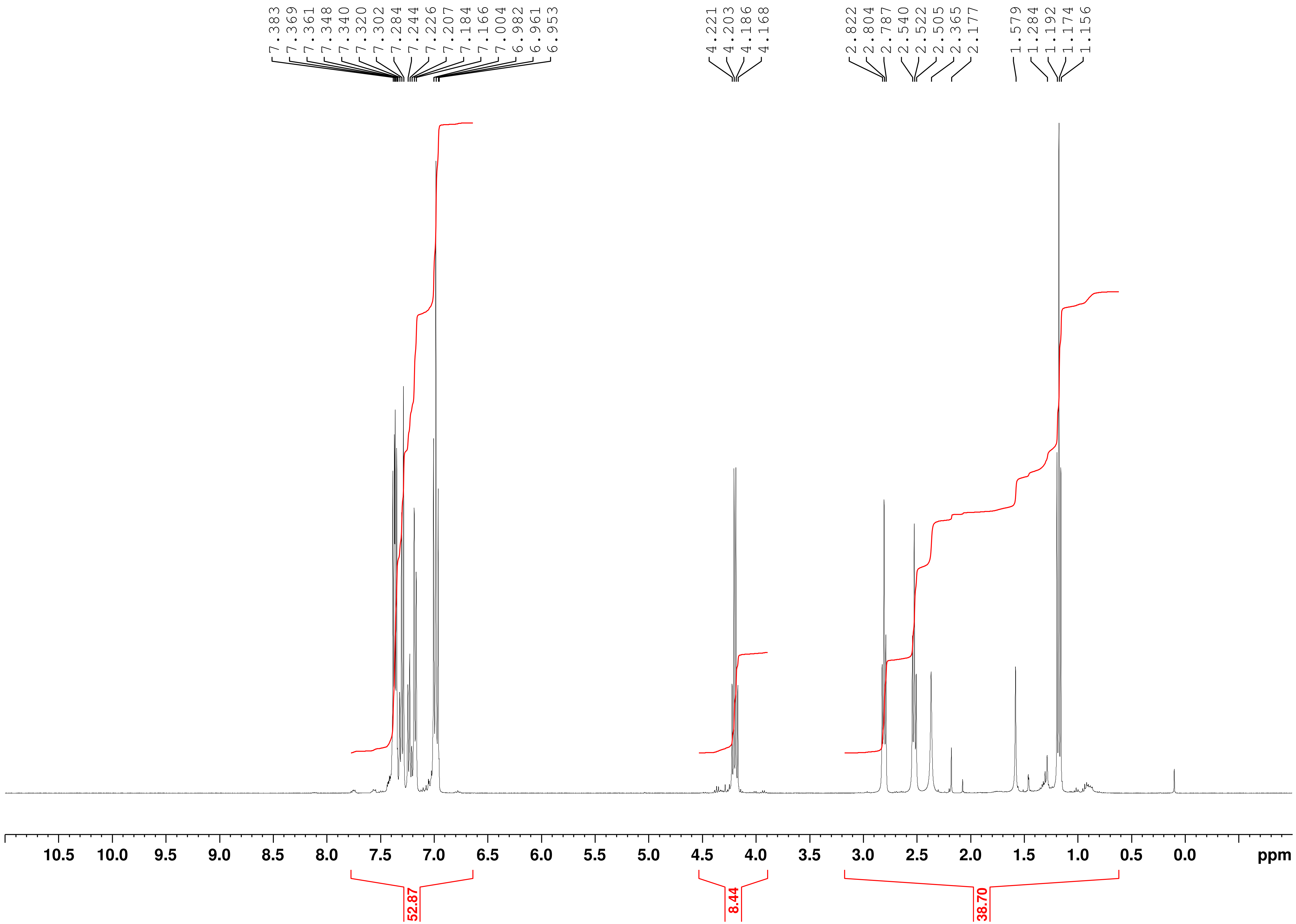


```
Current Data Parameters
NAME      pcxac8.AC229B_product
EXPNO                1
PROCNO               1

F2 - Acquisition Parameters
Date_           20211125
Time               17.35 h
INSTRUM         av3400hd
PROBHD   Z122623_0053 (
PULPROG             zg30
TD                 65536
SOLVENT            CDCl3
NS                    16
DS                     2
SWH             8223.685 Hz
FIDRES          0.250967 Hz
AQ             3.9845889 sec
RG                198.43
DW                60.800 usec
DE                 17.93 usec
TE                 298.0 K
D1            1.00000000 sec
TD0                    1
SFO1         400.2024714 MHz
NUC1                  1H
P1                 10.22 usec
PLW1         10.00000000 W

F2 - Processing parameters
SI                 65536
SF           400.2000000 MHz
WDW                   EM
SSB      0
LB                  0.30 Hz
GB       0
PC                  1.00
```
