# Supplementary material for: Modular Synthesis of α,α-Diaryl α-Amino Esters via Bi(V)-Mediated Arylation/SN2-Displacement of Kukhtin–Ramirez Intermediates
Source: Org Lett. 2022 Oct 24;24(43):8002–7. doi: 10.1021/acs.orglett.2c03201 (PMC9641671; doi:10.1021/acs.orglett.2c03201)
Supplement: Supplementary file 5 — ol2c03201_si_005.zip [file ol2c03201_si_005.zip › FID_18-22/18/18_COSY/pdata/1/pcxac8.AC229B_product_4_1.pdf]

Double quantum filtered COSY Spectrum.

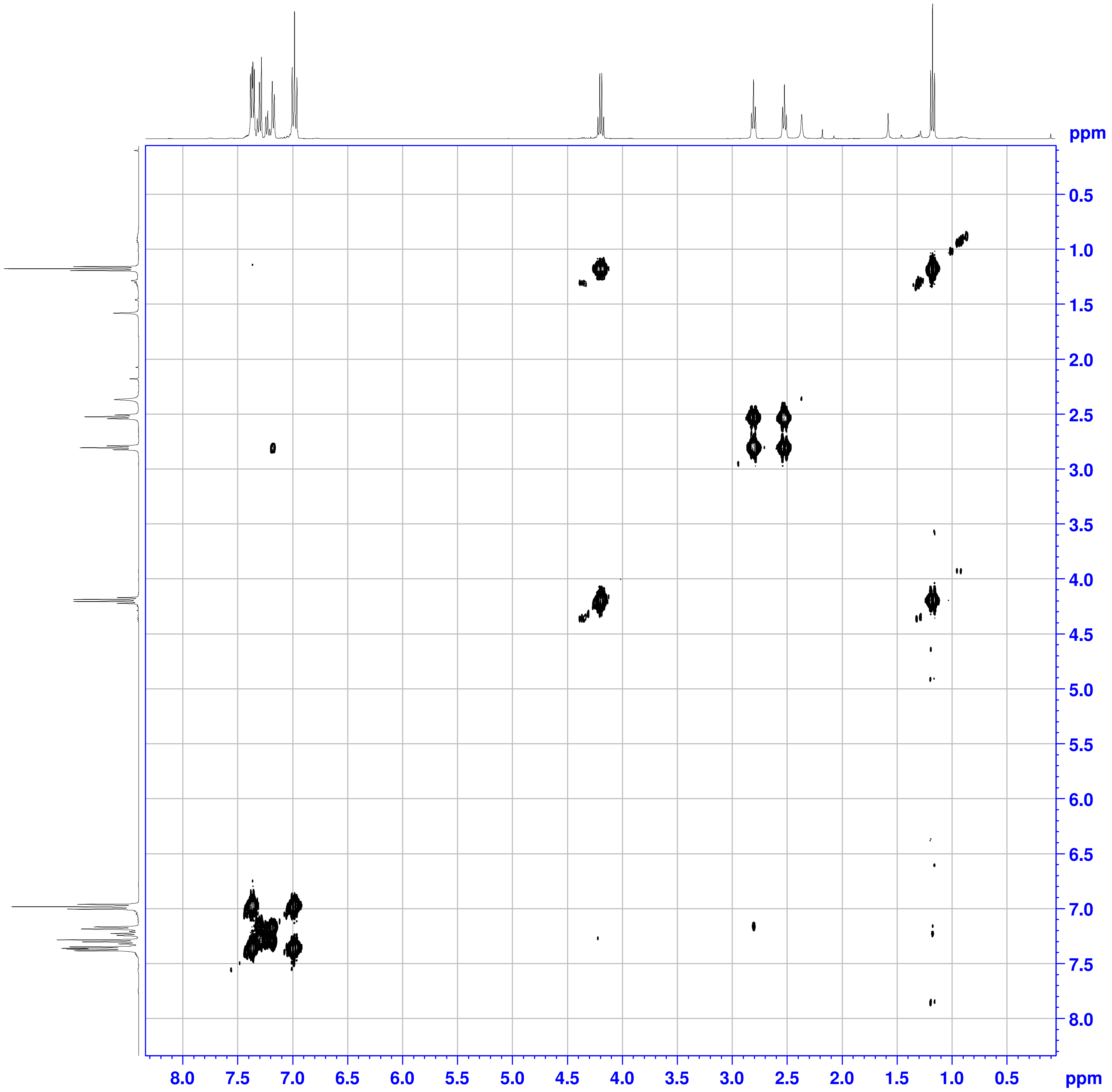


```
Current Data Parameters
NAME       pcxac8.AC229B_product
EXPNO                      4
PROCNO                     1

F2 - Acquisition Parameters
Date_             20211125
Time                 22.17 h
INSTRUM           av3400hd
PROBHD    Z122623_0053 (
PULPROG      cosygpmfppqf
TD                    2048
SOLVENT              CDCl3
NS                       2
DS                      16
SWH               3649.635 Hz
FIDRES            3.564097 Hz
AQ               0.2805760 sec
RG                  198.43
DW                 137.000 usec
DE                   27.10 usec
TE                   298.0 K
D0              0.00000300 sec
D1              0.91712409 sec
D11             0.03000000 sec
D12             0.00002000 sec
D13             0.00000400 sec
D16             0.00020000 sec
IN0             0.00027400 sec
TDav                     1
SFO1           400.2016798 MHz
NUC1                    1H
P1                   10.22 usec
P17                2500.00 usec
PLW1           10.00000000 W
PLW10           1.16050005 W
GPNAM[1]        SMSQ10.100
GPZ1                 16.00 %
GPNAM[2]        SMSQ10.100
GPZ2                 12.00 %
GPNAM[3]        SMSQ10.100
GPZ3                 40.00 %
P16                1000.00 usec

F1 - Acquisition parameters
TD                     128
SFO1              400.2017 MHz
FIDRES           57.025547 Hz
SW                   9.119 ppm
FnMODE                  QF

F2 - Processing parameters
SI                    2048
SF             400.2000000 MHz
WDW                   SINE
SSB       0
LB        0 Hz
GB        0
PC                    1.40

F1 - Processing parameters
SI                     512
MC2                     QF
SF             400.2000000 MHz
WDW                   SINE
SSB       0
LB        0 Hz
GB        0
```
