# Supplementary material for: Modular Synthesis of α,α-Diaryl α-Amino Esters via Bi(V)-Mediated Arylation/SN2-Displacement of Kukhtin–Ramirez Intermediates
Source: Org Lett. 2022 Oct 24;24(43):8002–7. doi: 10.1021/acs.orglett.2c03201 (PMC9641671; doi:10.1021/acs.orglett.2c03201)
Supplement: Supplementary file 6 — ol2c03201_si_006.zip [file ol2c03201_si_006.zip › FID_23-27/23/23_1H/pdata/1/pcxac8.AC267_product_1_1.pdf]

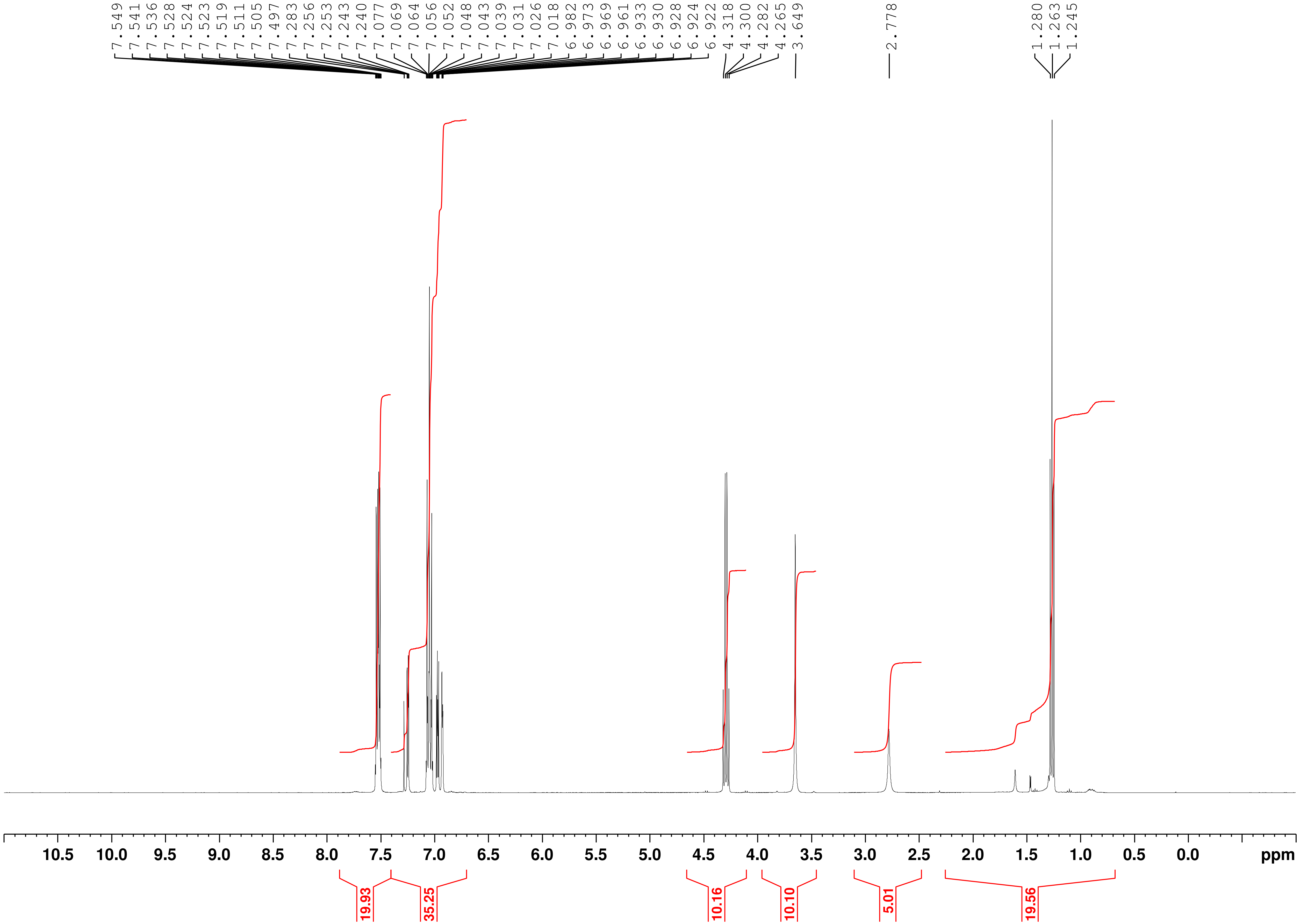

Current Data Parameters
NAME pcxac8.AC267_product
EXPNO 1
PROCNO 1

F2 - Acquisition Parameters
Date_ 20220216
Time 17.54 h
INSTRUM av3400
PROBHD Z104450_0225 (
PULPROG zg30
TD 65536
SOLVENT CDCl3
NS 16
DS 2
SWH 8223.685 Hz
FIDRES 0.250967 Hz
AQ 3.9845889 sec
RG 101
DW 60.800 usec
DE 16.03 usec
TE 298.0 K
D1 1.00000000 sec
TD0 1
SFO1 400.0724706 MHz
NUC1 1H
P1 14.00 usec
PLW1 11.92800045 W

F2 - Processing parameters
SI 65536
SF 400.0700000 MHz
WDW EM
SSB 0
LB 0.30 Hz
GB 0
PC 1.00
