# Supplementary material for: Modular Synthesis of α,α-Diaryl α-Amino Esters via Bi(V)-Mediated Arylation/SN2-Displacement of Kukhtin–Ramirez Intermediates
Source: Org Lett. 2022 Oct 24;24(43):8002–7. doi: 10.1021/acs.orglett.2c03201 (PMC9641671; doi:10.1021/acs.orglett.2c03201)
Supplement: Supplementary file 7 — ol2c03201_si_007.zip [file ol2c03201_si_007.zip › FID_28-32/28/28_13C/pdata/1/pcxac8.AC336_productdry_3_1.pdf]

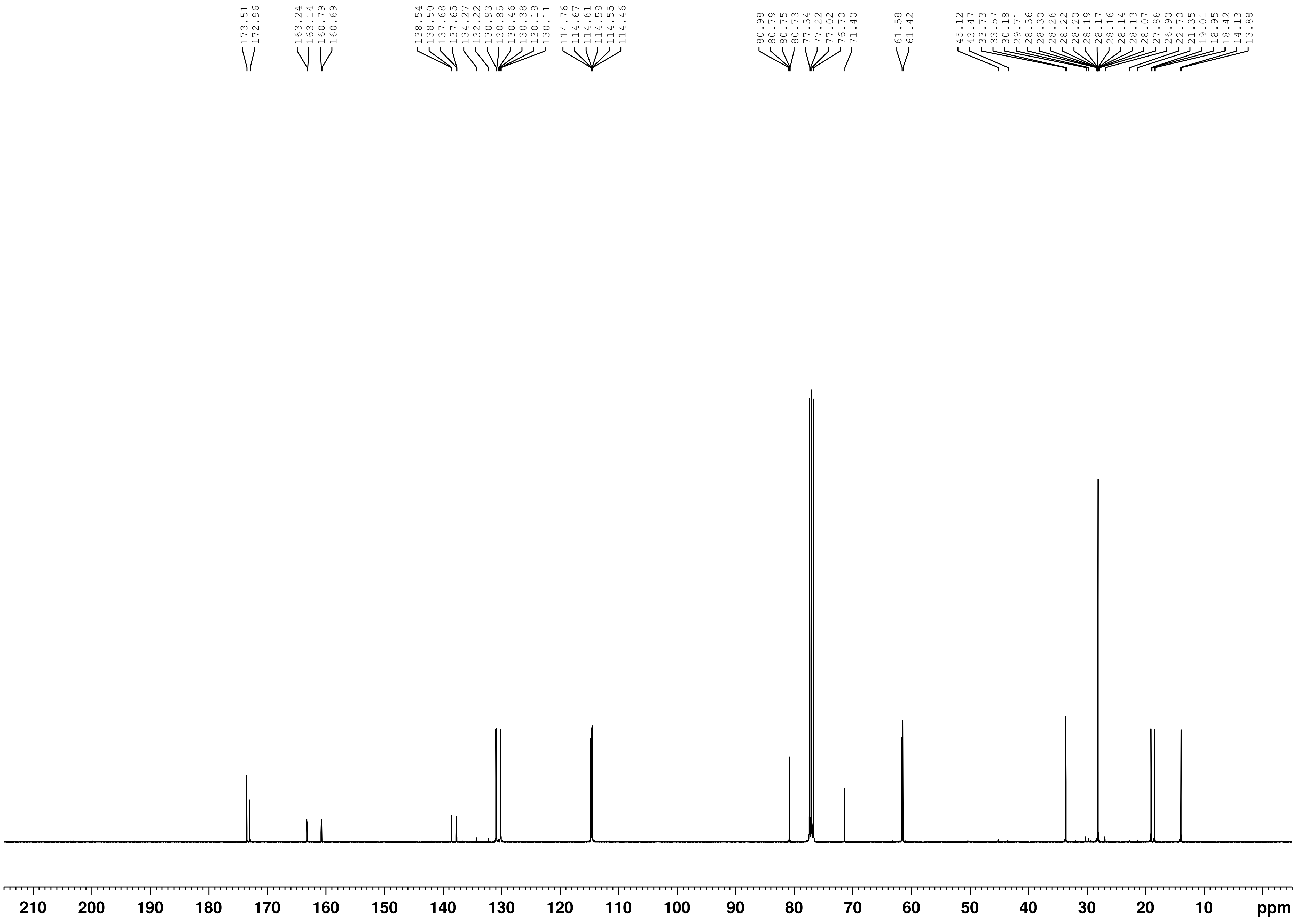

Current Data Parameters

NAME	pcxac8.AC336_productdry
EXPNO	3
PROCNO	1

F2 - Acquisition Parameters

Date_	20220429
Time	1.40 h
INSTRUM	av3400hd
PROBHD	Z122623_0053 (
PULPROG	zgpg30
TD	32768
SOLVENT	CDCl3
NS	4096
DS	4
SWH	23809.523 Hz
FIDRES	1.453218 Hz
AQ	0.6881280 sec
RG	125.13
DW	21.000 usec
DE	18.80 usec
TE	298.0 K
D1	1.00000000 sec
D11	0.03000000 sec
TD0	1
SFO1	100.6414394 MHz
NUC1	13C
P1	10.19 usec
PLW1	38.00000000 W
SFO2	400.2016008 MHz
NUC2	1H
CPDPRG[2	waltz16
PCPD2	90.00 usec
PLW2	10.00000000 W
PLW12	0.12895000 W
PLW13	0.06486000 W

F2 - Processing parameters

SI	32768
SF	100.6303700 MHz
WDW	EM
SSB	0
LB	1.00 Hz
GB	0
PC	1.40
