# Supplementary material for: Modular Synthesis of α,α-Diaryl α-Amino Esters via Bi(V)-Mediated Arylation/SN2-Displacement of Kukhtin–Ramirez Intermediates
Source: Org Lett. 2022 Oct 24;24(43):8002–7. doi: 10.1021/acs.orglett.2c03201 (PMC9641671; doi:10.1021/acs.orglett.2c03201)
Supplement: Supplementary file 7 — ol2c03201_si_007.zip [file ol2c03201_si_007.zip › FID_28-32/28/28_1H/pdata/1/pcxac8.AC336_productdry_1_1.pdf]

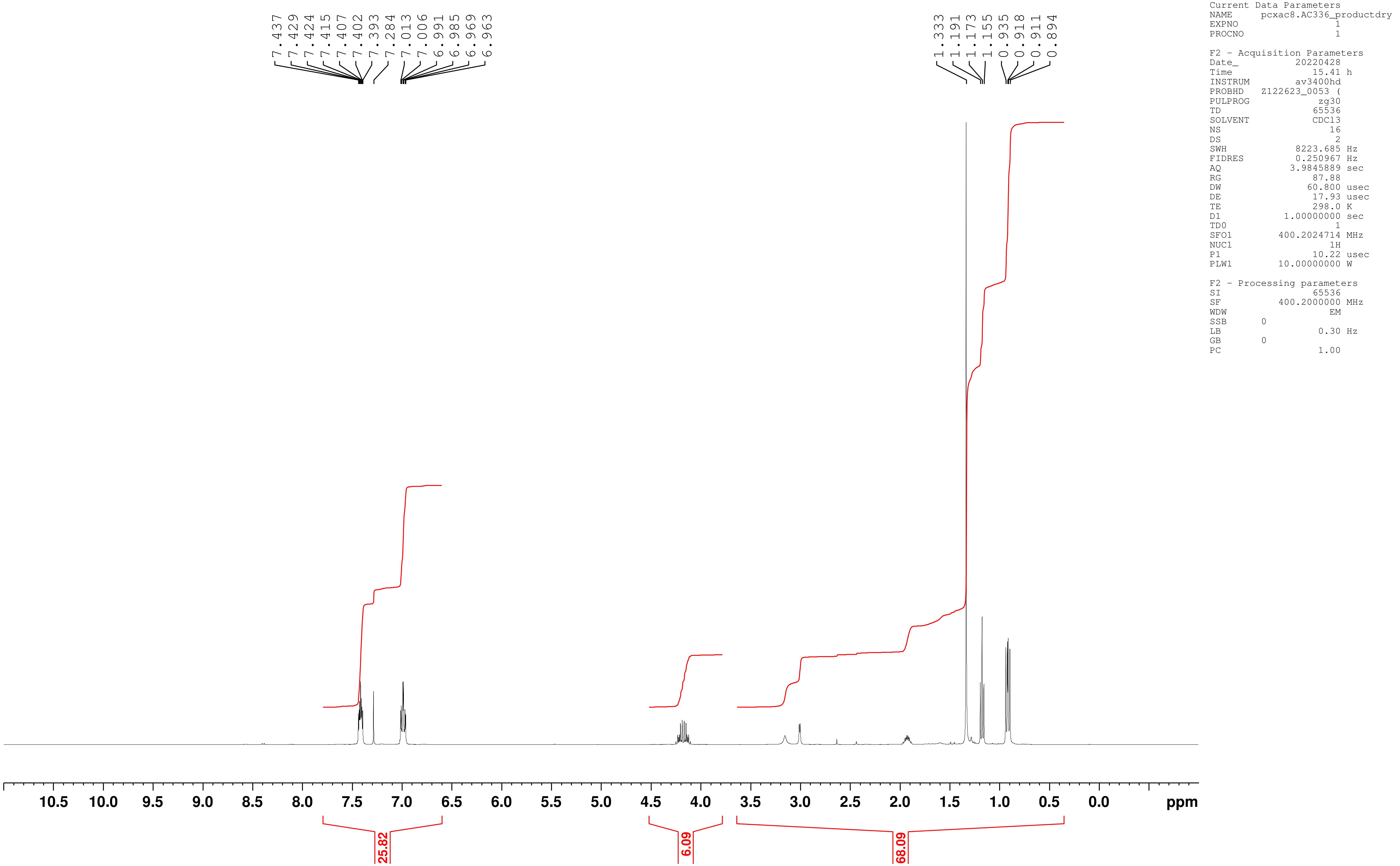
7.437
7.429
7.424
7.415
7.407
7.402
7.393
7.284
7.013
7.006
6.991
6.985
6.969
6.963
1.333
1.191
1.173
1.155
0.935
0.918
0.911
0.894
25.82
6.09
68.09
ppm
Current Data Parameters
NAME pcxac8.AC336_productdry
EXPNO 1
PROCNO 1
F2 - Acquisition Parameters
Date_ 20220428
Time 15.41 h
INSTRUM av3400hd
PROBHD Z122623_0053 (
PULPROG zg30
TD 65536
SOLVENT CDCl3
NS 16
DS 2
SWH 8223.685 Hz
FIDRES 0.250967 Hz
AQ 3.9845889 sec
RG 87.88
DW 60.800 usec
DE 17.93 usec
TE 298.0 K
D1 1.00000000 sec
TD0 1
SFO1 400.2024714 MHz
NUC1 1H
P1 10.22 usec
PLW1 10.00000000 W
F2 - Processing parameters
SI 65536
SF 400.2000000 MHz
WDW EM
SSB 0
LB 0.30 Hz
GB 0
PC 1.00
