# Supplementary material for: Modular Synthesis of α,α-Diaryl α-Amino Esters via Bi(V)-Mediated Arylation/SN2-Displacement of Kukhtin–Ramirez Intermediates
Source: Org Lett. 2022 Oct 24;24(43):8002–7. doi: 10.1021/acs.orglett.2c03201 (PMC9641671; doi:10.1021/acs.orglett.2c03201)
Supplement: Supplementary file 8 — ol2c03201_si_008.zip [file ol2c03201_si_008.zip › FID_33-38/34/34_19F/2/pdata/1/pcxac8.AC295_product_2_1.pdf]

Processed with back linear prediction to eliminate baseline artefacts: broad peaks may be attenuated. Standard data in file 100002

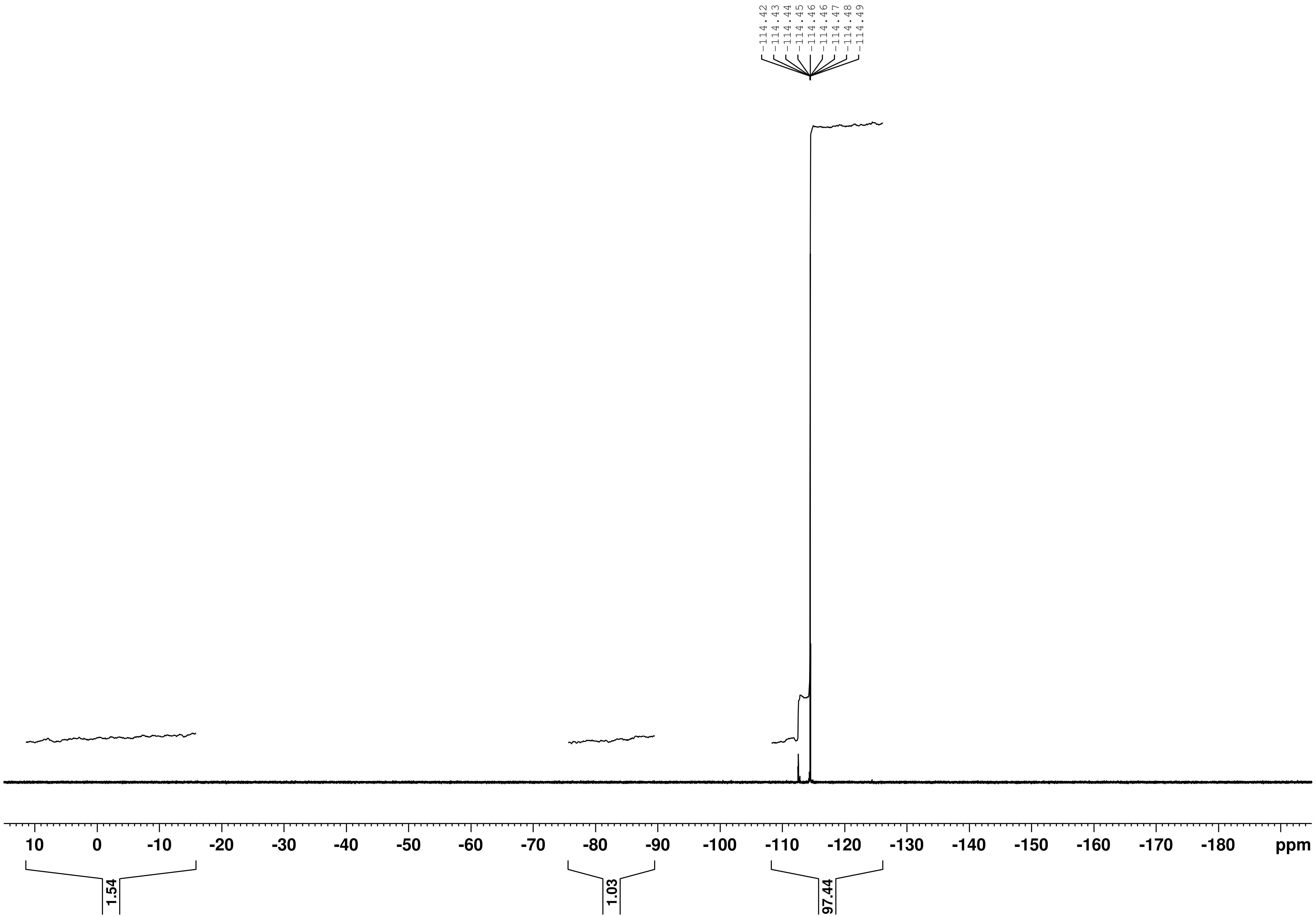


```
Current Data Parameters
NAME     pcxac8.AC295_product
EXPNO                    2
PROCNO                   1

F2 - Acquisition Parameters
Date_           20220314
Time               14.31 h
INSTRUM           av3400
PROBHD   Z104450_0225 (
PULPROG           zgflqn
TD                261992
SOLVENT            CDCl3
NS                    16
DS                     4
SWH           85227.273 Hz
FIDRES         0.650610 Hz
AQ            1.5370197 sec
RG                  1150
DW                 5.867 usec
DE                  6.77 usec
TE                 298.0 K
D1            1.50000000 sec
TD0                    1
SFO1        376.4080300 MHz
NUC1                 19F
P1                 13.30 usec
PLW1         16.11100006 W

F2 - Processing parameters
SI                262144
SF          376.4419098 MHz
WDW                   EM
SSB      0
LB                  1.00 Hz
GB       0
PC                  1.00
```
