# Supplementary material for: Modular Synthesis of α,α-Diaryl α-Amino Esters via Bi(V)-Mediated Arylation/SN2-Displacement of Kukhtin–Ramirez Intermediates
Source: Org Lett. 2022 Oct 24;24(43):8002–7. doi: 10.1021/acs.orglett.2c03201 (PMC9641671; doi:10.1021/acs.orglett.2c03201)
Supplement: Supplementary file 9 — ol2c03201_si_009.zip [file ol2c03201_si_009.zip › FID_Bi-cpds/pCl Ar3Bi/13C/pdata/1/email_pcxac8.AC360_dry2_2_1.pdf]

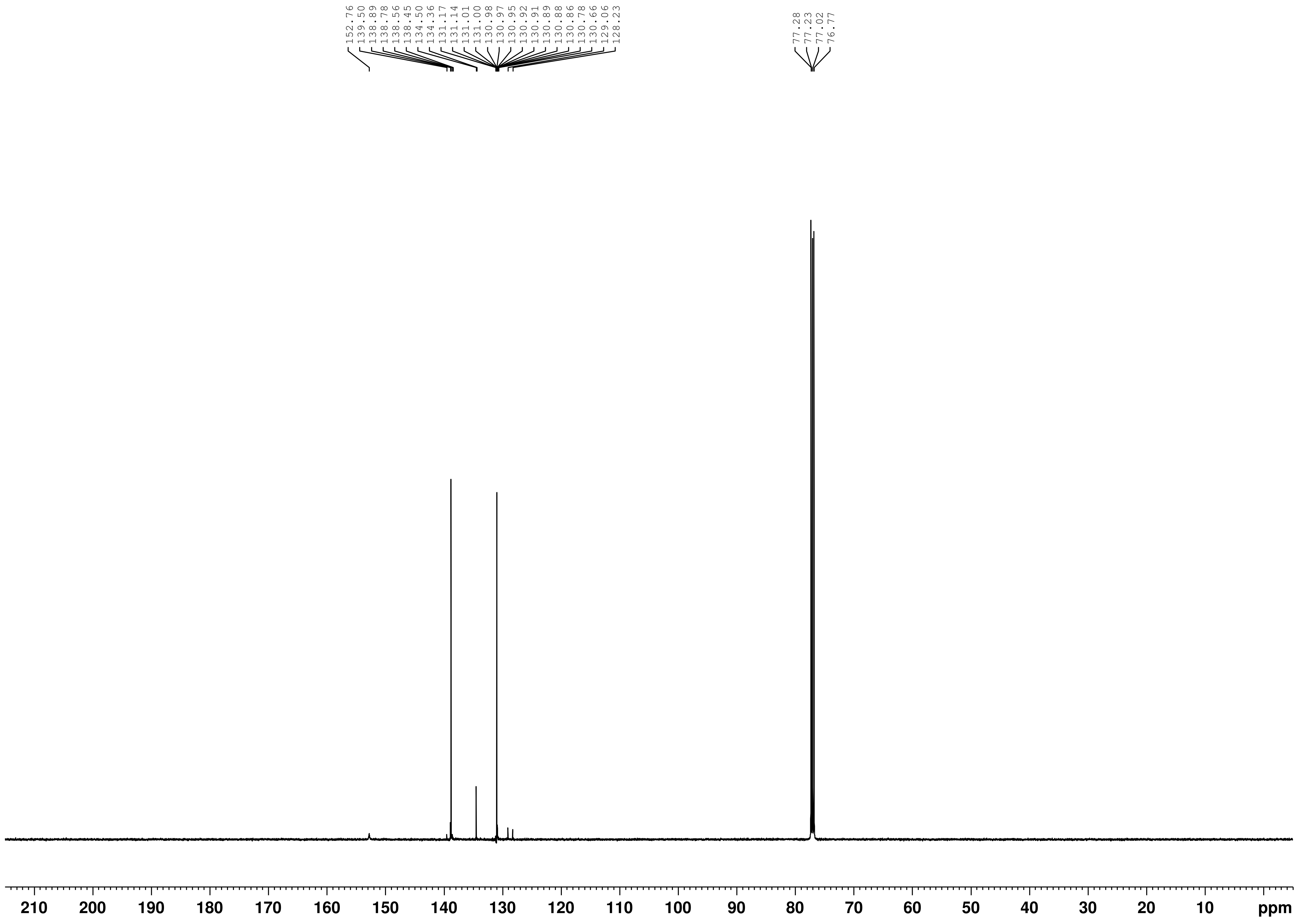

Current Data Parameters
NAME pcxac8.AC360_dry2
EXPNO 2
PROCNO 1

F2 - Acquisition Parameters
Date_ 20220609
Time 5.40 h
INSTRUM av3500
PROBHD Z8007_0120 (PH
PULPROG zgpg30
TD 32768
SOLVENT CDCl3
NS 8000
DS 32
SWH 29761.904 Hz
FIDRES 1.816522 Hz
AQ 0.5505024 sec
RG 2050
DW 16.800 usec
DE 9.58 usec
TE 298.0 K
D1 1.00000000 sec
D11 0.03000000 sec
TD0 1
SFO1 125.7716219 MHz
NUC1 13C
P1 9.25 usec
PLW1 59.84099960 W
SFO2 500.1320005 MHz
NUC2 1H
CPDPRG[2 waltz16
PCPD2 80.00 usec
PLW2 29.99200058 W
PLW12 0.46862000 W
PLW13 0.23571000 W

F2 - Processing parameters
SI 32768
SF 125.7577885 MHz
WDW EM
SSB 0
LB 1.00 Hz
GB 0
PC 1.40
