# Supplementary material for: Modular Synthesis of α,α-Diaryl α-Amino Esters via Bi(V)-Mediated Arylation/SN2-Displacement of Kukhtin–Ramirez Intermediates
Source: Org Lett. 2022 Oct 24;24(43):8002–7. doi: 10.1021/acs.orglett.2c03201 (PMC9641671; doi:10.1021/acs.orglett.2c03201)
Supplement: Supplementary file 9 — ol2c03201_si_009.zip [file ol2c03201_si_009.zip › FID_Bi-cpds/pF Ar3Bi/13C/pdata/1/k_ruf.KR039-P_7_1.pdf]

UserID k_ruf SampleID KR039-P SupervisorID Iball Slot Number 17

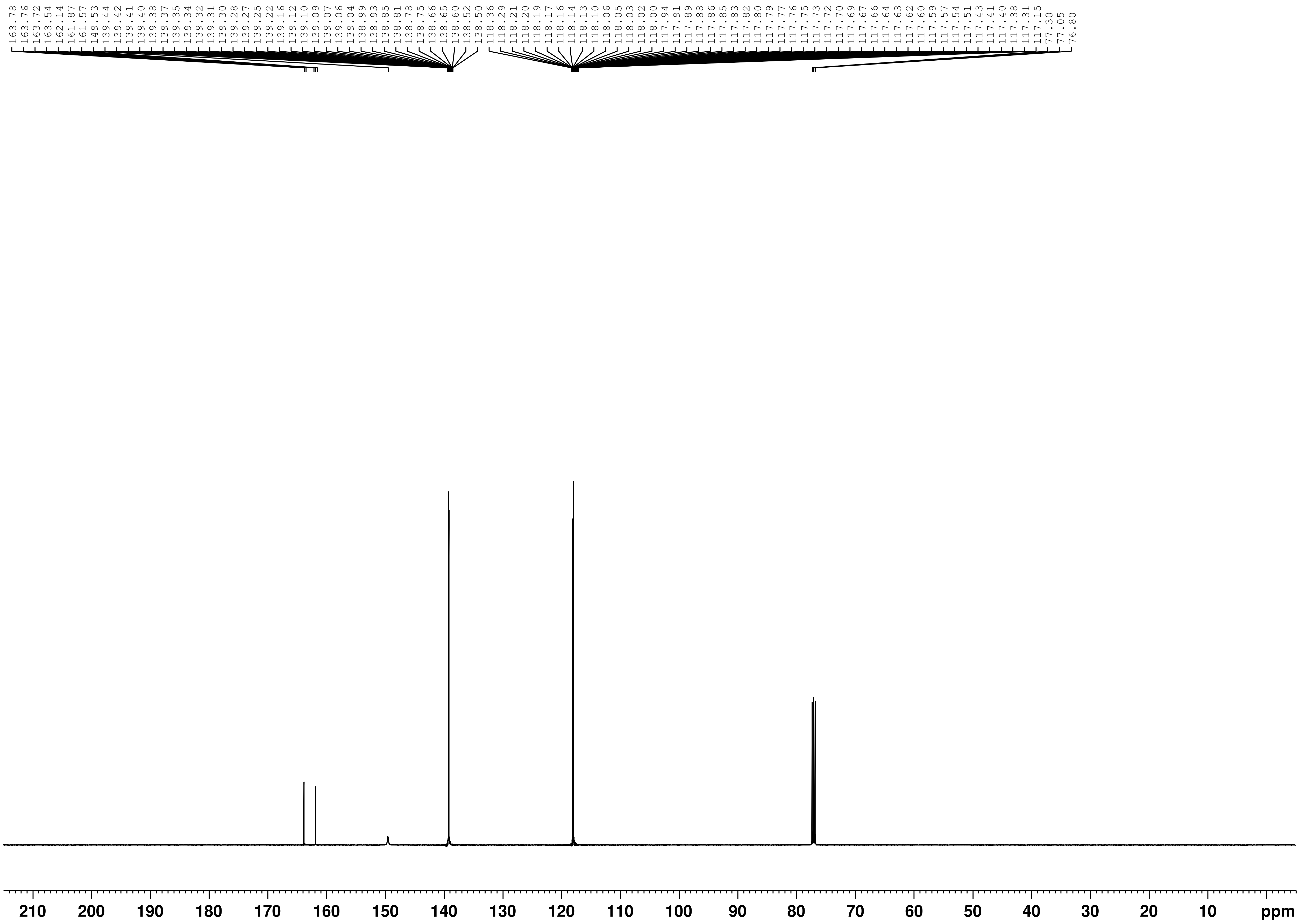


```
Current Data Parameters
NAME      k_ruf.KR039-P
EXPNO                 7
PROCNO                1

F2 - Acquisition Parameters
Date_          20190219
Time              19.56 h
INSTRUM       cnlnmr500
PROBHD   Z122624_0037 (
PULPROG         zgpg30
TD                32768
SOLVENT           CDCl3
NS                 1024
DS                    4
SWH          29761.904 Hz
FIDRES        1.816522 Hz
AQ           0.5505024 sec
RG               200.55
DW               16.800 usec
DE                20.85 usec
TE                298.0 K
D1          1.00000000 sec
D11         0.03000000 sec
TD0                   1
SFO1       125.7791662 MHz
NUC1                13C
P1                 9.90 usec
PLW1       57.00000000 W
SFO2       500.1620006 MHz
NUC2                 1H
CPDPRG[2         waltz16
PCPD2             80.00 usec
PLW2       12.60400009 W
PLW12       0.27888000 W
PLW13       0.14005999 W

F2 - Processing parameters
SI                32768
SF         125.7653320 MHz
WDW                  EM
SSB      0
LB                 1.00 Hz
GB       0
PC                 1.40
```
