# Supplementary material for: Modular Synthesis of α,α-Diaryl α-Amino Esters via Bi(V)-Mediated Arylation/SN2-Displacement of Kukhtin–Ramirez Intermediates
Source: Org Lett. 2022 Oct 24;24(43):8002–7. doi: 10.1021/acs.orglett.2c03201 (PMC9641671; doi:10.1021/acs.orglett.2c03201)
Supplement: Supplementary file 9 — ol2c03201_si_009.zip [file ol2c03201_si_009.zip › FID_Bi-cpds/pF Ar3Bi/19F/pdata/1/k_ruf.KR039-P_2_1.pdf]

UserID k_ruf SampleID KR039-P SupervisorID lball Slot Number 8

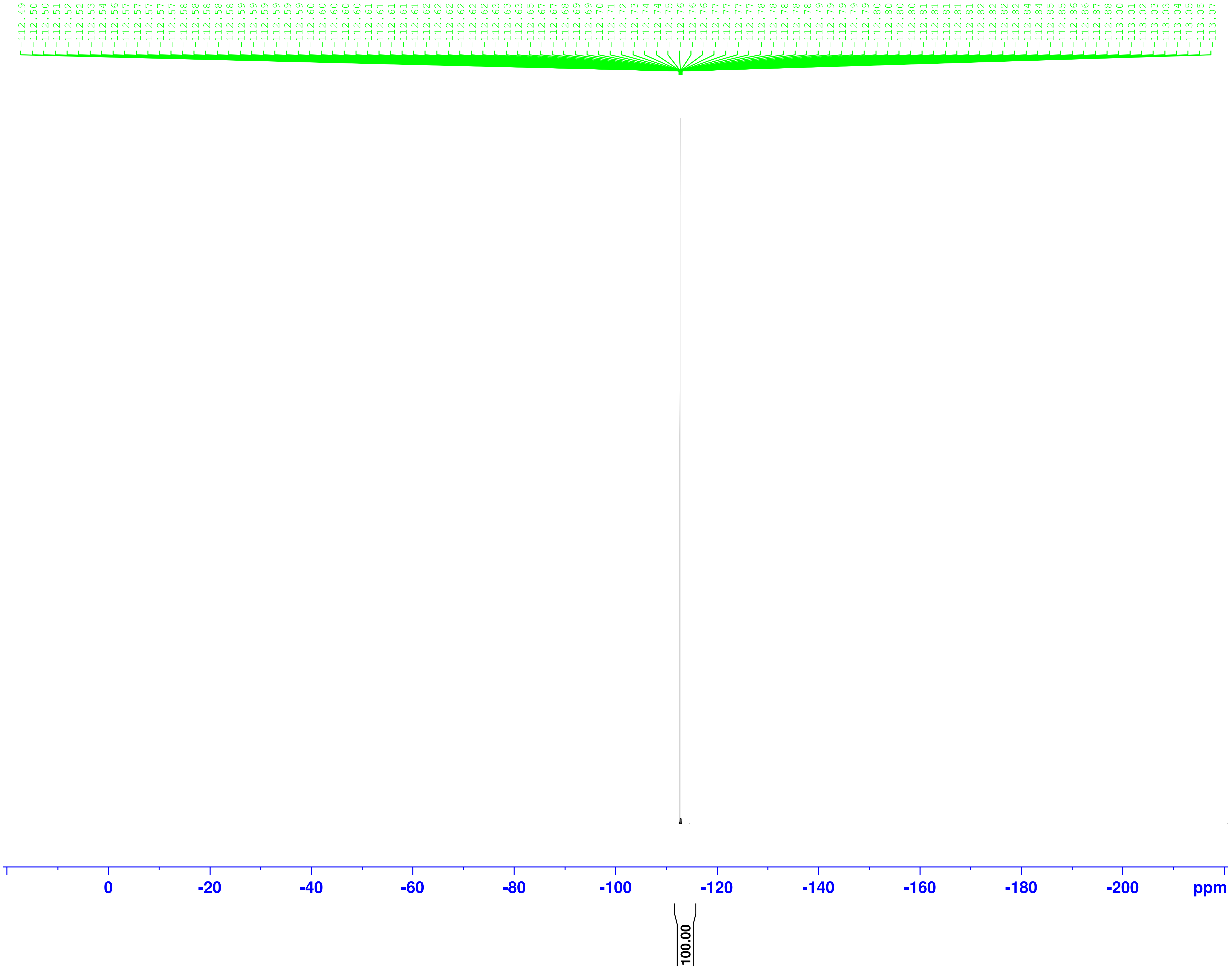

Current Data Parameters
NAME k_ruf.KR039-P
EXPNO 2
PROCNO 1

F2 - Acquisition Parameters
Date_ 20190219
Time 13.10 h
INSTRUM cnlnmr500
PROBHD Z122624_0037 (
PULPROG zgflqn
TD 261912
SOLVENT CDCl3
NS 16
DS 4
SWH 113636.367 Hz
FIDRES 0.867745 Hz
AQ 1.1524128 sec
RG 15.87
DW 4.400 usec
DE 30.00 usec
TE 298.0 K
D1 1.00000000 sec
TD0 1
SFO1 470.5735434 MHz
NUC1 19F
P1 14.00 usec
PLW1 13.47200012 W

F2 - Processing parameters
SI 262144
SF 470.6206054 MHz
WDW EM
SSB 0
LB 0.30 Hz
GB 0
PC 1.00
