# Supplementary material for: Modular Synthesis of α,α-Diaryl α-Amino Esters via Bi(V)-Mediated Arylation/SN2-Displacement of Kukhtin–Ramirez Intermediates
Source: Org Lett. 2022 Oct 24;24(43):8002–7. doi: 10.1021/acs.orglett.2c03201 (PMC9641671; doi:10.1021/acs.orglett.2c03201)
Supplement: Supplementary file 9 — ol2c03201_si_009.zip [file ol2c03201_si_009.zip › FID_Bi-cpds/pF Ar3Bi/1H/pdata/1/k_ruf.KR039-P_1_1.pdf]

UserID k_ruf SampleID KR039-P SupervisorID Iball Slot Number 8

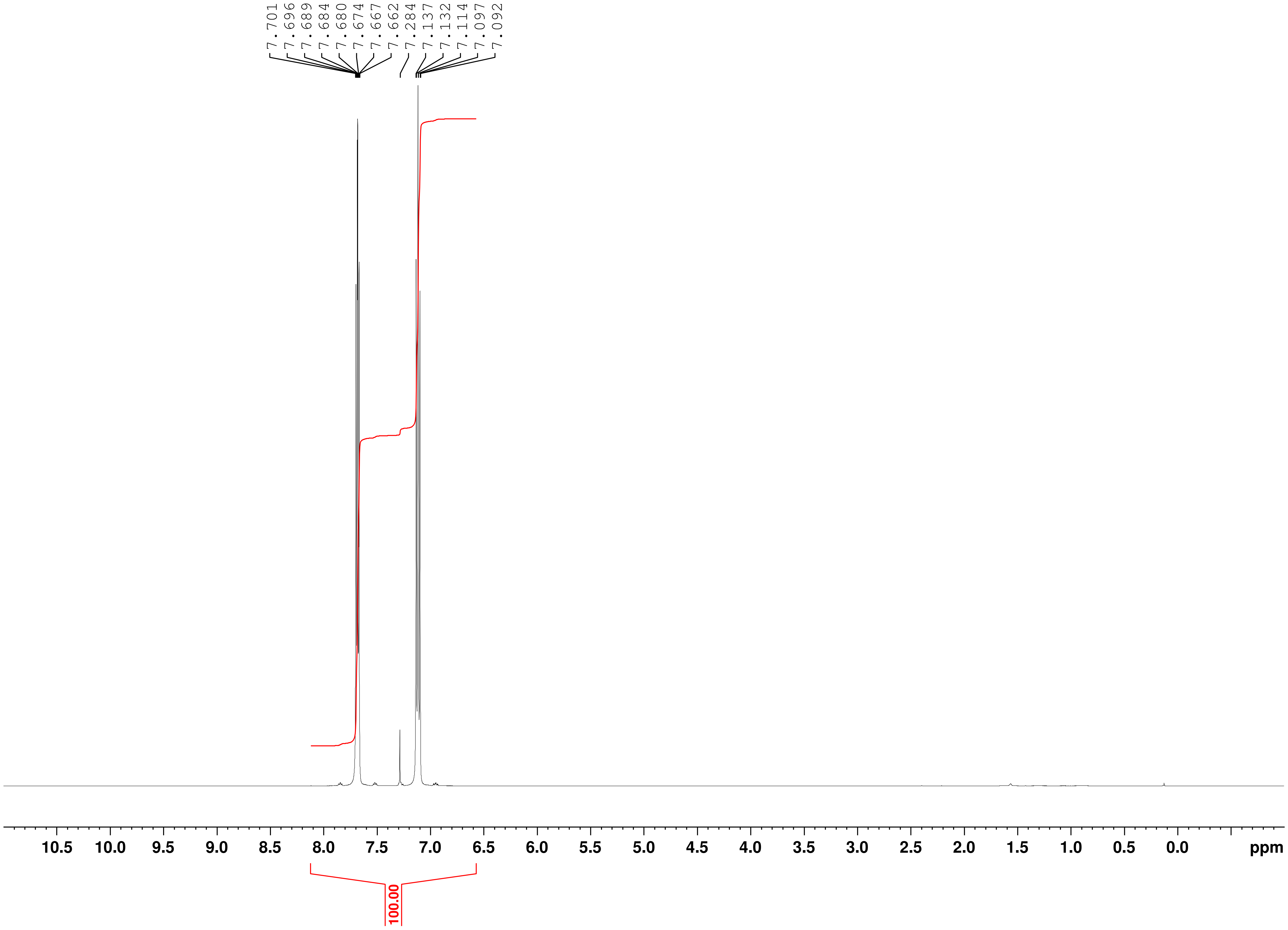

Current Data Parameters
NAME k_ruf.KR039-P
EXPNO 1
PROCNO 1

F2 - Acquisition Parameters
Date_ 20190219
Time 13.08 h
INSTRUM cnlnmr500
PROBHD Z122624_0037 (
PULPROG zg30
TD 65536
SOLVENT CDCl3
NS 16
DS 2
SWH 10288.065 Hz
FIDRES 0.313967 Hz
AQ 3.1850495 sec
RG 70.49
DW 48.600 usec
DE 44.15 usec
TE 298.0 K
D1 1.00000000 sec
TD0 1
SFO1 500.1630887 MHz
NUC1 1H
P1 11.90 usec
PLW1 12.60400009 W

F2 - Processing parameters
SI 65536
SF 500.1600000 MHz
WDW EM
SSB 0
LB 0.30 Hz
GB 0
PC 1.00
