# Supplementary material for: Modular Synthesis of α,α-Diaryl α-Amino Esters via Bi(V)-Mediated Arylation/SN2-Displacement of Kukhtin–Ramirez Intermediates
Source: Org Lett. 2022 Oct 24;24(43):8002–7. doi: 10.1021/acs.orglett.2c03201 (PMC9641671; doi:10.1021/acs.orglett.2c03201)
Supplement: Supplementary file 10 — ol2c03201_si_010.zip [file ol2c03201_si_010.zip › FID keto ester/FID keto ester/1c/13C/pdata/1/pcxac8.AC235_column2_dry_2_1.pdf]

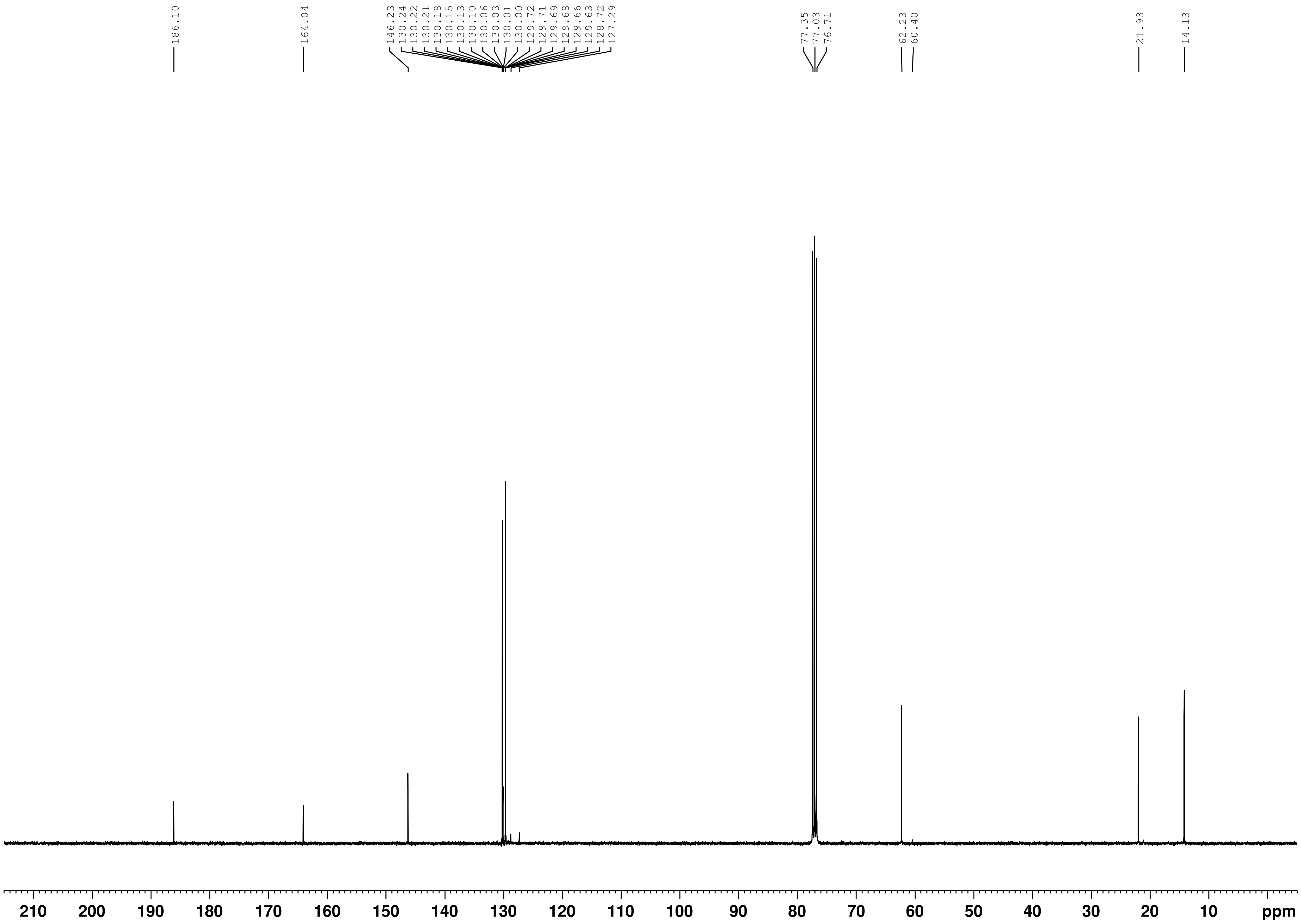


```
Current Data Parameters
NAME     pcxac8.AC235_colum
EXPNO                    2
PROCNO                   1

F2 - Acquisition Parameters
Date_            20211208
Time                21.04 h
INSTRUM            av3400
PROBHD   Z104450_0225 (
PULPROG           zgpg30
TD                 32768
SOLVENT            CDCl3
NS                  4096
DS                     4
SWH            24038.461 Hz
FIDRES          1.467191 Hz
AQ             0.6815744 sec
RG                  2050
DW                20.800 use
DE                  9.63 use
TE                 298.0 K
D1            1.00000000 sec
D11           0.03000000 sec
TD0                    1
SFO1         100.6087473 MHz
NUC1                 13C
P1                  9.75 use
PLW1         63.68099976 W
SFO2         400.0716003 MHz
NUC2                  1H
CPDPRG[2         waltz16
PCPD2              90.00 use
PLW2         11.92800045 W
PLW12         0.28863001 W
PLW13         0.14518000 W

F2 - Processing parameters
SI                 32768
SF           100.5976815 MHz
WDW                   EM
SSB      0
LB                  1.00 Hz
GB       0
PC                  1.40
```
