# Supplementary material for: Modular Synthesis of α,α-Diaryl α-Amino Esters via Bi(V)-Mediated Arylation/SN2-Displacement of Kukhtin–Ramirez Intermediates
Source: Org Lett. 2022 Oct 24;24(43):8002–7. doi: 10.1021/acs.orglett.2c03201 (PMC9641671; doi:10.1021/acs.orglett.2c03201)
Supplement: Supplementary file 10 — ol2c03201_si_010.zip [file ol2c03201_si_010.zip › FID keto ester/FID keto ester/1c/COSY/pdata/1/pcxac8.AC235_column2_dry_3_1.pdf]

Double quantum filtered COSY Spectrum.

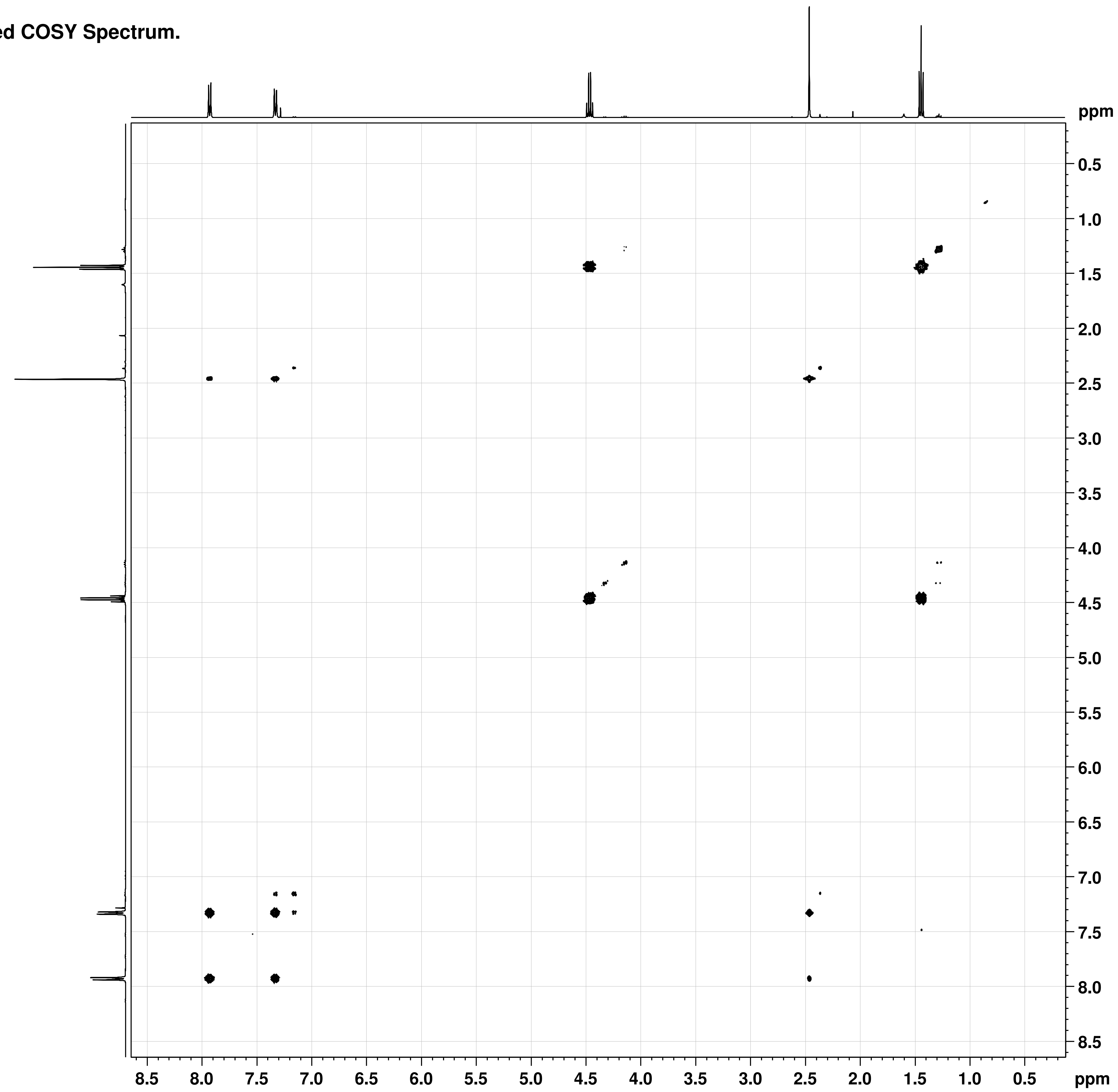


```
Current Data Parameters
NAME     pcxac8.AC235_column2_
EXPNO                     3
PROCNO                    1

F2 - Acquisition Parameters
Date_              20211208
Time                  21.06 h
INSTRUM              av3400
PROBHD   Z104450_0225 (
PULPROG    cosygpmfppqf
TD                     2048
SOLVENT               CDCl3
NS                        4
DS                       16
SWH                3746.254 Hz
FIDRES             3.658451 Hz
AQ                0.2733397 sec
RG                     2050
DW                  133.467 usec
DE                     8.63 usec
TE                    298.0 K
D0               0.00000300 sec
D1               0.92477000 sec
D11              0.03000000 sec
D12              0.00002000 sec
D13              0.00000400 sec
D16              0.00020000 sec
IN0              0.00026700 sec
TDav                      1
SFO1            400.0717556 MHz
NUC1                     1H
P1                    14.00 usec
P17                 2500.00 usec
PLW1            11.92800045 W
PLW10            2.59770012 W
GPNAM[1]         SMSQ10.100
GPZ1                  16.00 %
GPNAM[2]         SMSQ10.100
GPZ2                  12.00 %
GPNAM[3]         SMSQ10.100
GPZ3                  40.00 %
P16                 1000.00 usec

F1 - Acquisition parameters
TD                      512
SFO1               400.0718 MHz
FIDRES            14.630150 Hz
SW                    9.362 ppm
FnMODE                   QF

F2 - Processing parameters
SI                     2048
SF              400.0700000 MHz
WDW                    SINE
SSB      0
LB       0 Hz
GB       0
PC                     1.40

F1 - Processing parameters
SI                     2048
MC2                      QF
SF              400.0700000 MHz
WDW                    SINE
SSB      0
LB       0 Hz
GB       0
```
