# Supplementary material for: Modular Synthesis of α,α-Diaryl α-Amino Esters via Bi(V)-Mediated Arylation/SN2-Displacement of Kukhtin–Ramirez Intermediates
Source: Org Lett. 2022 Oct 24;24(43):8002–7. doi: 10.1021/acs.orglett.2c03201 (PMC9641671; doi:10.1021/acs.orglett.2c03201)
Supplement: Supplementary file 3 — ol2c03201_si_003.zip [file ol2c03201_si_003.zip › FID_8-12/11/11_19F/2/pdata/1/pcxac8.AC315_productdry_2_1.pdf]

Processed with back linear prediction to eliminate baseline artefacts: broad peaks may be attenuated. Standard data in file 100002

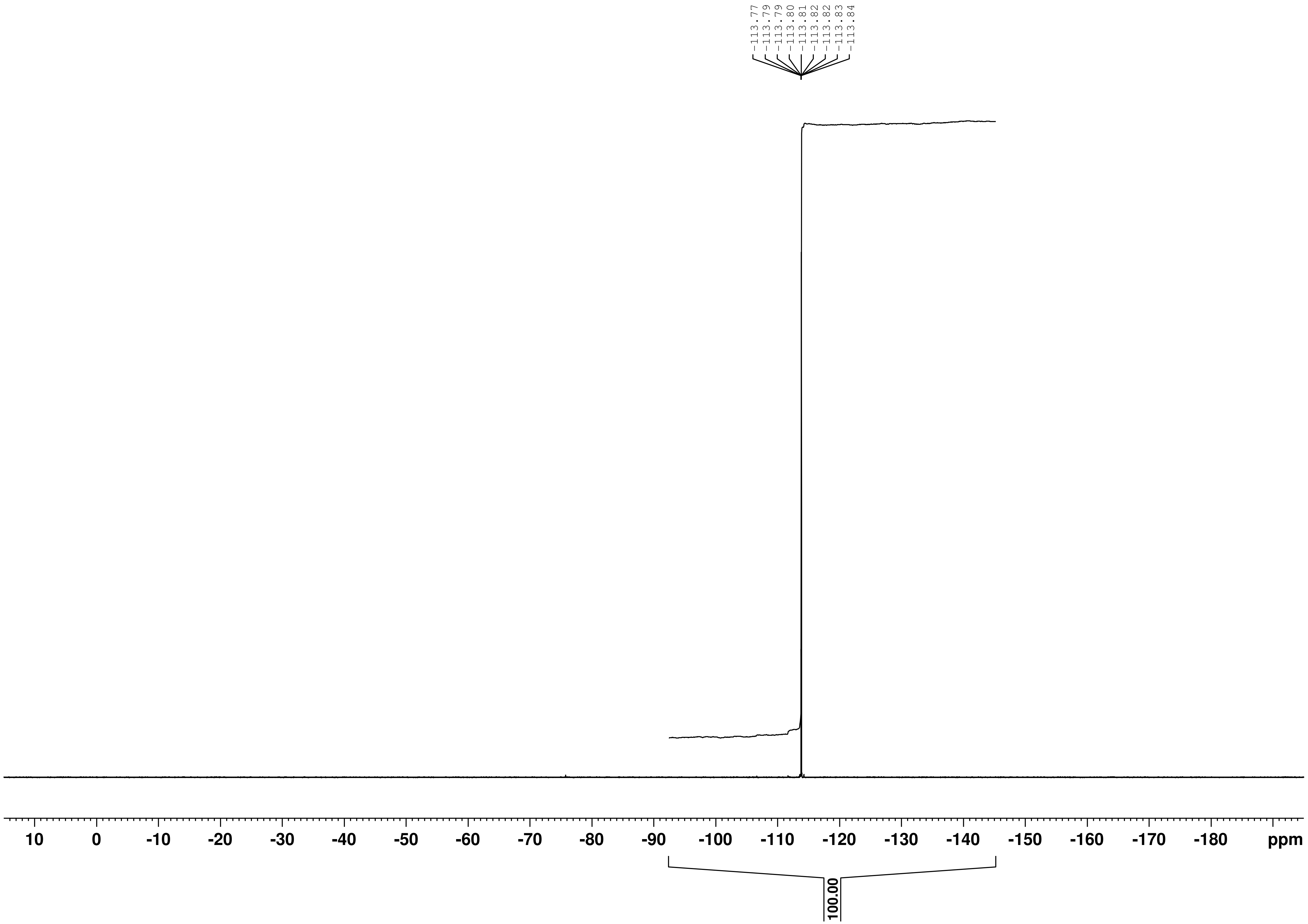


```
Current Data Parameters
NAME     pcxac8.AC315_productdry
EXPNO                  2
PROCNO                 1

F2 - Acquisition Parameters
Date_          20220401
Time              13.28 h
INSTRUM          av3400
PROBHD   Z104450_0225 (
PULPROG          zgflqn
TD               261992
SOLVENT           CDCl3
NS                   16
DS                    4
SWH          85227.273 Hz
FIDRES        0.650610 Hz
AQ           1.5370197 sec
RG                 1150
DW                5.867 usec
DE                 6.77 usec
TE                298.0 K
D1           1.50000000 sec
TD0                   1
SFO1        376.4080300 MHz
NUC1                19F
P1                13.30 usec
PLW1        16.11100006 W

F2 - Processing parameters
SI               262144
SF          376.4419098 MHz
WDW                  EM
SSB      0
LB                 1.00 Hz
GB       0
PC                 1.00
```
