# Supplementary material for: Modular Synthesis of α,α-Diaryl α-Amino Esters via Bi(V)-Mediated Arylation/SN2-Displacement of Kukhtin–Ramirez Intermediates
Source: Org Lett. 2022 Oct 24;24(43):8002–7. doi: 10.1021/acs.orglett.2c03201 (PMC9641671; doi:10.1021/acs.orglett.2c03201)
Supplement: Supplementary file 3 — ol2c03201_si_003.zip [file ol2c03201_si_003.zip › FID_8-12/11/11_1H/pdata/1/pcxac8.AC315_productdry_1_1.pdf]

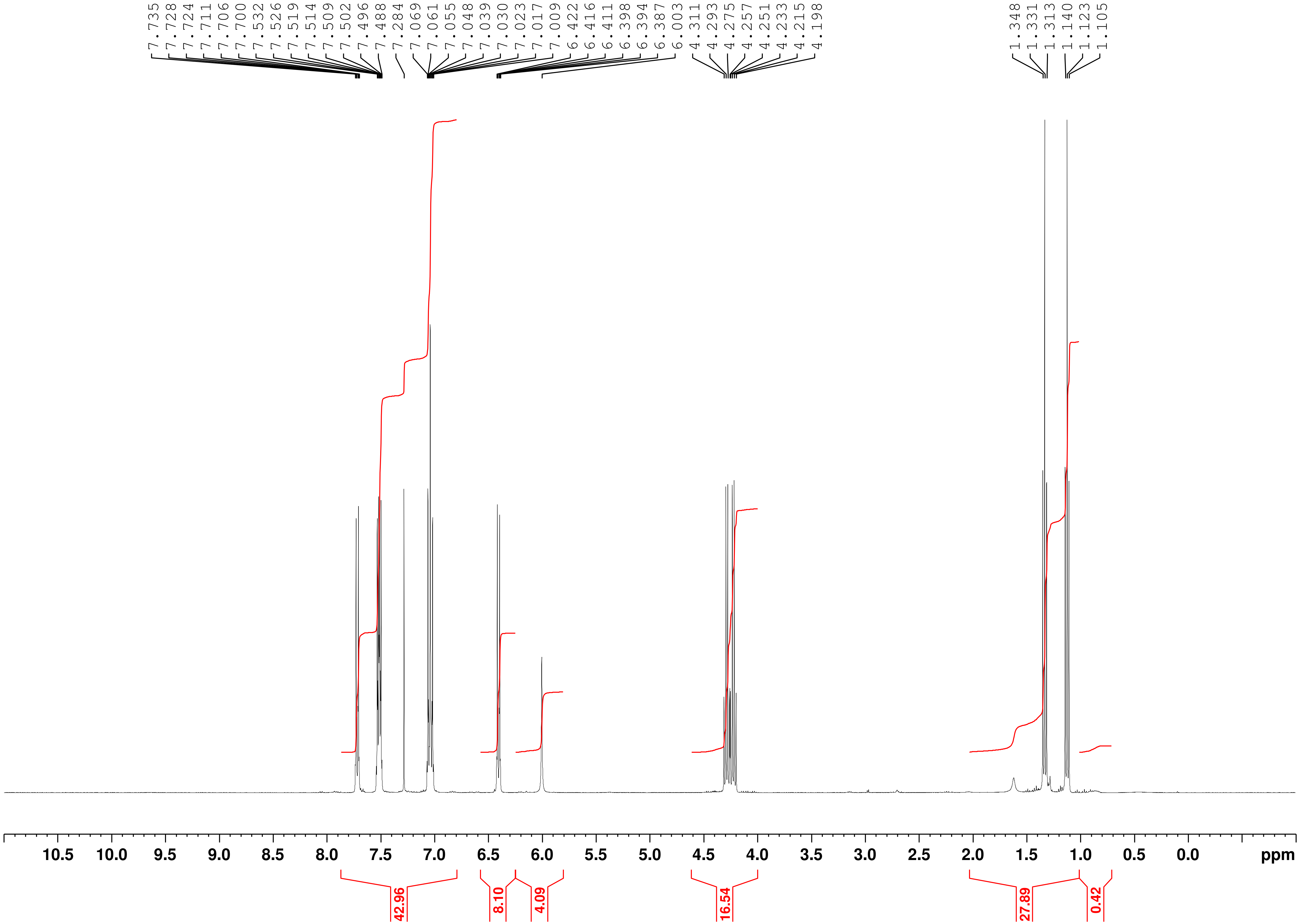

Current Data Parameters

NAME	pcxac8.AC315_productdry
EXPNO	1
PROCNO	1

F2 - Acquisition Parameters

Date_	20220401
Time	13.26 h
INSTRUM	av3400
PROBHD	Z104450_0225 (
PULPROG	zg30
TD	65536
SOLVENT	CDCl3
NS	16
DS	2
SWH	8223.685 Hz
FIDRES	0.250967 Hz
AQ	3.9845889 sec
RG	228
DW	60.800 usec
DE	16.03 usec
TE	298.0 K
D1	1.00000000 sec
TD0	1
SFO1	400.0724706 MHz
NUC1	1H
P1	14.00 usec
PLW1	11.92800045 W

F2 - Processing parameters

SI	65536
SF	400.0700000 MHz
WDW	EM
SSB	0
LB	0.30 Hz
GB	0
PC	1.00
