# Supplementary material for: Modular Synthesis of α,α-Diaryl α-Amino Esters via Bi(V)-Mediated Arylation/SN2-Displacement of Kukhtin–Ramirez Intermediates
Source: Org Lett. 2022 Oct 24;24(43):8002–7. doi: 10.1021/acs.orglett.2c03201 (PMC9641671; doi:10.1021/acs.orglett.2c03201)
Supplement: Supplementary file 3 — ol2c03201_si_003.zip [file ol2c03201_si_003.zip › FID_8-12/11/11_COSY/pdata/1/pcxac8.AC315_productdry_3_1.pdf]

Double quantum filtered COSY Spectrum.

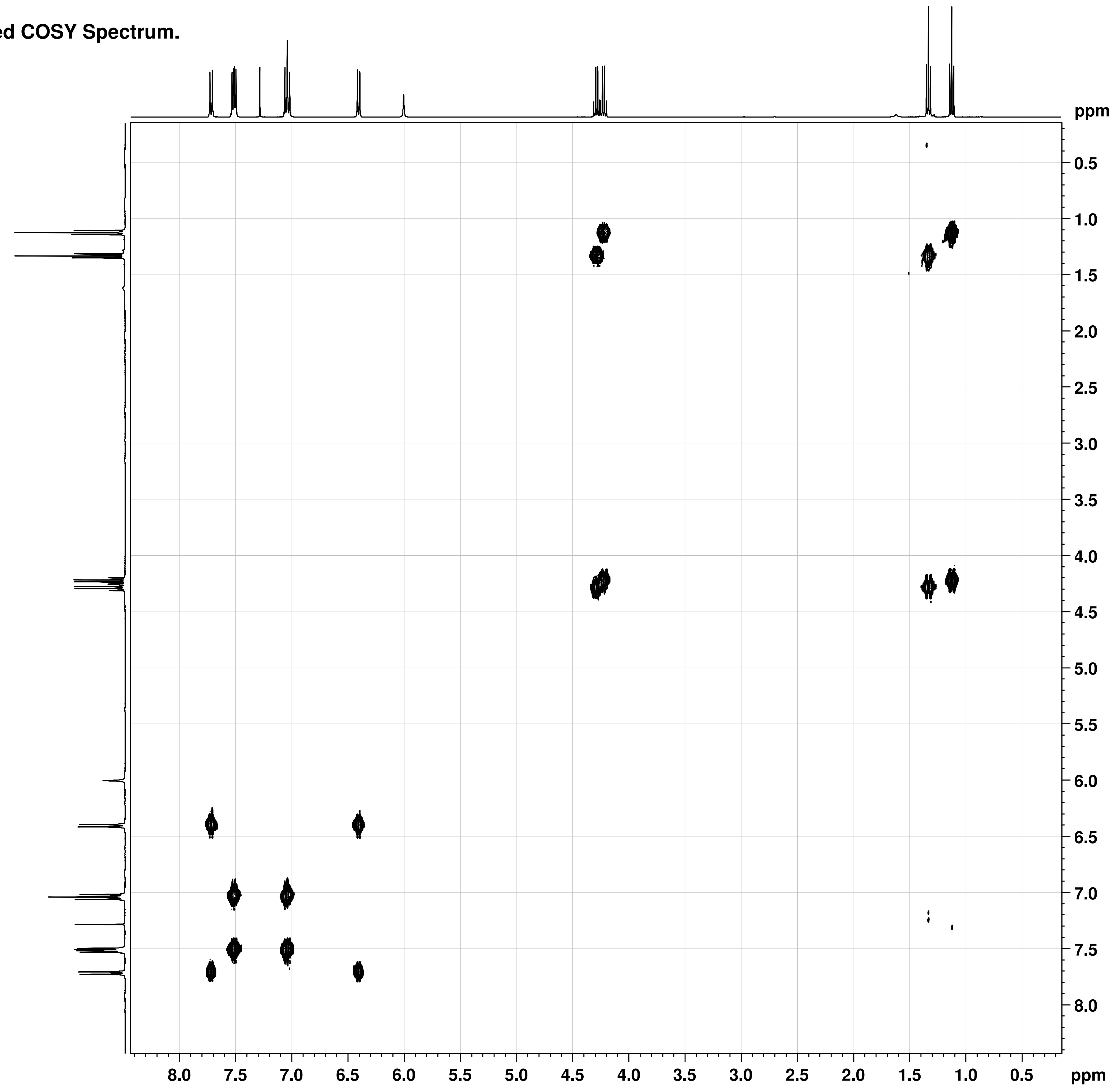


```
Current Data Parameters
NAME      pcxac8.AC315_productd
EXPNO                     3
PROCNO                    1

F2 - Acquisition Parameters
Date_            20220402
Time                 0.02 h
INSTRUM            av3400
PROBHD   Z104450_0225 (
PULPROG      cosygpmfppqf
TD                   2048
SOLVENT             CDCl3
NS                      2
DS                     16
SWH              3649.635 Hz
FIDRES           3.564097 Hz
AQ              0.2805760 sec
RG                   2050
DW                137.000 usec
DE                   8.87 usec
TE                  298.0 K
D0             0.00000300 sec
D1             0.91657799 sec
D11            0.03000000 sec
D12            0.00002000 sec
D13            0.00000400 sec
D16            0.00020000 sec
IN0            0.00027400 sec
TDav                    1
SFO1          400.0717169 MHz
NUC1                   1H
P1                  14.00 usec
P17               2500.00 usec
PLW1          11.92800045 W
PLW10          2.59770012 W
GPNAM[1]       SMSQ10.100
GPZ1                16.00 %
GPNAM[2]       SMSQ10.100
GPZ2                12.00 %
GPNAM[3]       SMSQ10.100
GPZ3                40.00 %
P16               1000.00 usec

F1 - Acquisition parameters
TD                    128
SFO1             400.0717 MHz
FIDRES          57.025547 Hz
SW                  9.122 ppm
FnMODE                 QF

F2 - Processing parameters
SI                   2048
SF            400.0700000 MHz
WDW                  SINE
SSB      0
LB       0 Hz
GB       0
PC                   1.40

F1 - Processing parameters
SI                    512
MC2                    QF
SF            400.0700000 MHz
WDW                  SINE
SSB      0
LB       0 Hz
GB       0
```
