# Supplementary material for: Modular Synthesis of α,α-Diaryl α-Amino Esters via Bi(V)-Mediated Arylation/SN2-Displacement of Kukhtin–Ramirez Intermediates
Source: Org Lett. 2022 Oct 24;24(43):8002–7. doi: 10.1021/acs.orglett.2c03201 (PMC9641671; doi:10.1021/acs.orglett.2c03201)
Supplement: Supplementary file 3 — ol2c03201_si_003.zip [file ol2c03201_si_003.zip › FID_8-12/12/12_13C/pdata/1/pcxac8.AC261_productdry_2_1.pdf]

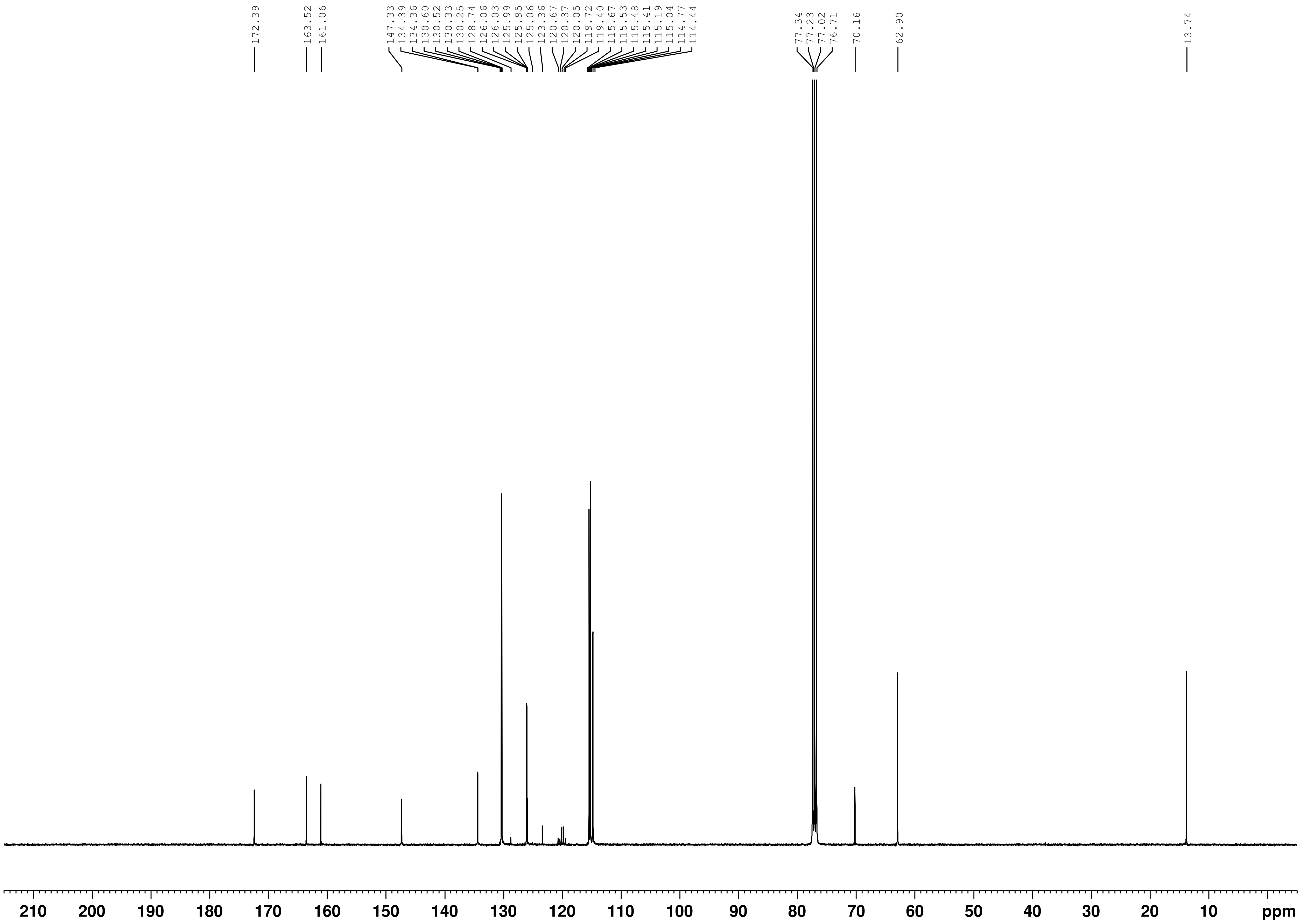


```
Current Data Parameters
NAME      pcxac8.AC261_productdry
EXPNO                   2
PROCNO                  1

F2 - Acquisition Parameters
Date_            20220204
Time                 2.40 h
INSTRUM          av3400hd
PROBHD   Z122623_0053 (
PULPROG            zgpg30
TD                  32768
SOLVENT             CDCl3
NS                   4096
DS                      4
SWH             23809.523 Hz
FIDRES           1.453218 Hz
AQ              0.6881280 sec
RG                 140.92
DW                 21.000 usec
DE                  18.80 usec
TE                  298.0 K
D1             1.00000000 sec
D11            0.03000000 sec
TD0                     1
SFO1         100.6414394 MHz
NUC1                  13C
P1                  10.19 usec
PLW1          38.00000000 W
SFO2         400.2016008 MHz
NUC2                   1H
CPDPRG[2          waltz16
PCPD2               90.00 usec
PLW2          10.00000000 W
PLW12          0.12895000 W
PLW13          0.06486000 W

F2 - Processing parameters
SI                  32768
SF           100.6303700 MHz
WDW                    EM
SSB      0
LB                   1.00 Hz
GB       0
PC                   1.40
```
