# Supplementary material for: Modular Synthesis of α,α-Diaryl α-Amino Esters via Bi(V)-Mediated Arylation/SN2-Displacement of Kukhtin–Ramirez Intermediates
Source: Org Lett. 2022 Oct 24;24(43):8002–7. doi: 10.1021/acs.orglett.2c03201 (PMC9641671; doi:10.1021/acs.orglett.2c03201)
Supplement: Supplementary file 3 — ol2c03201_si_003.zip [file ol2c03201_si_003.zip › FID_8-12/12/12_19F/4/pdata/1/pcxac8.AC261_productdry_4_1.pdf]

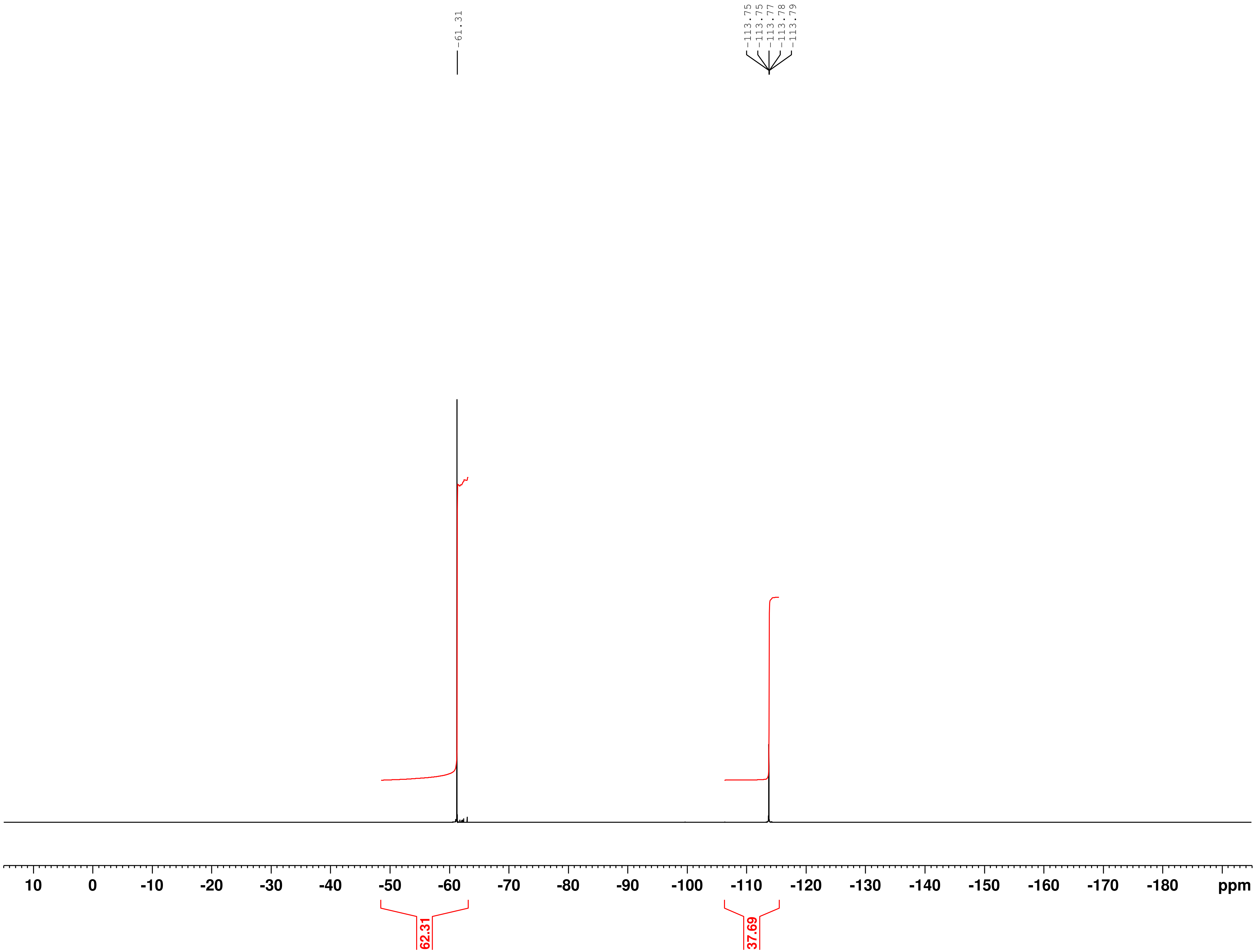

Current Data Parameters	
NAME	pcxac8.AC261_productdry
EXPNO	4
PROCNO	1
F2 - Acquisition Parameters	
Date_	20220204
Time	2.49 h
INSTRUM	av3400hd
PROBHD	Z122623_0053 (
PULPROG	zgflqn
TD	261896
SOLVENT	CDCl3
NS	16
DS	4
SWH	85227.273 Hz
FIDRES	0.650848 Hz
AQ	1.5364566 sec
RG	12.56
DW	5.867 usec
DE	18.56 usec
TE	298.0 K
D1	1.50000000 sec
TD0	1
SFO1	376.5303412 MHz
NUC1	19F
P1	13.80 usec
PLW1	8.69999981 W
F2 - Processing parameters	
SI	262144
SF	376.5642320 MHz
WDW	EM
SSB	0
LB	1.00 Hz
GB	0
PC	1.00
