# Supplementary material for: Modular Synthesis of α,α-Diaryl α-Amino Esters via Bi(V)-Mediated Arylation/SN2-Displacement of Kukhtin–Ramirez Intermediates
Source: Org Lett. 2022 Oct 24;24(43):8002–7. doi: 10.1021/acs.orglett.2c03201 (PMC9641671; doi:10.1021/acs.orglett.2c03201)
Supplement: Supplementary file 3 — ol2c03201_si_003.zip [file ol2c03201_si_003.zip › FID_8-12/12/12_COSY/pdata/1/pcxac8.AC261_productdry_3_1.pdf]

Double quantum filtered COSY Spectrum.

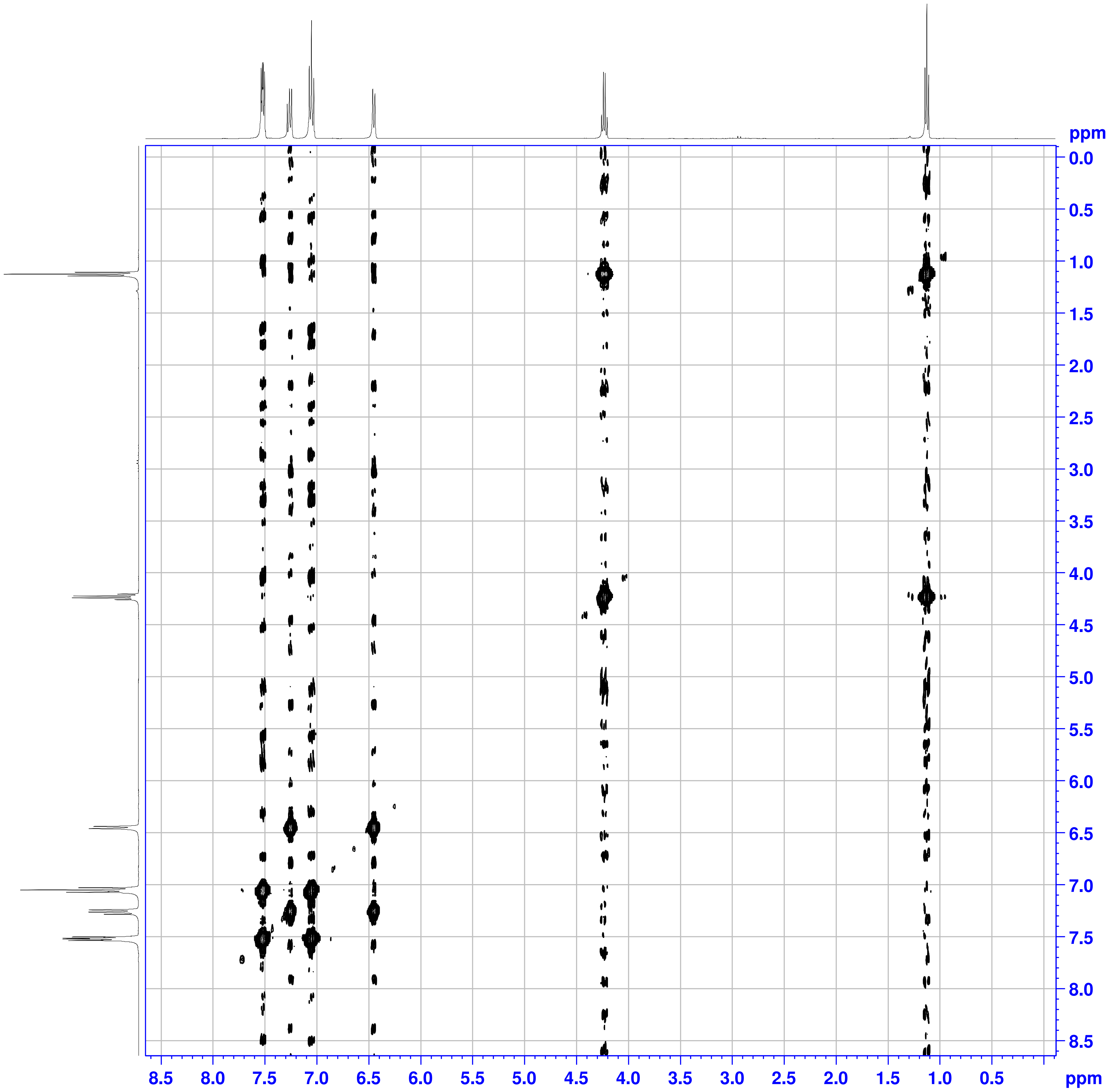

Current Data Parameters

NAME	pcxac8.AC261_productd
EXPNO	3
PROCNO	1

F2 - Acquisition Parameters

Date_	20220204
Time	2.42 h
INSTRUM	av3400hd
PROBHD	Z122623_0053 (
PULPROG	cosygpmfppqf
TD	2048
SOLVENT	CDCl3
NS	2
DS	16
SWH	3852.080 Hz
FIDRES	3.761797 Hz
AQ	0.2658304 sec
RG	198.43
DW	129.800 usec
DE	25.78 usec
TE	298.0 K
D0	0.00000300 sec
D1	0.93350810 sec
D11	0.03000000 sec
D12	0.00002000 sec
D13	0.00000400 sec
D16	0.00020000 sec
IN0	0.00025980 sec
TDav	1
SFO1	400.2017078 MHz
NUC1	1H
P1	10.22 usec
P17	2500.00 usec
PLW1	10.00000000 W
PLW10	1.16050005 W
GPNAM[1]	SMSQ10.100
GPZ1	16.00 %
GPNAM[2]	SMSQ10.100
GPZ2	12.00 %
GPNAM[3]	SMSQ10.100
GPZ3	40.00 %
P16	1000.00 usec

F1 - Acquisition parameters

TD	128
SFO1	400.2017 MHz
FIDRES	60.142418 Hz
SW	9.618 ppm
FnMODE	QF

F2 - Processing parameters

SI	2048
SF	400.2000000 MHz
WDW	SINE
SSB	0
LB	0 Hz
GB	0
PC	1.40

F1 - Processing parameters

SI	512
MC2	QF
SF	400.2000000 MHz
WDW	SINE
SSB	0
LB	0 Hz
GB	0
