# Supplementary material for: Modular Synthesis of α,α-Diaryl α-Amino Esters via Bi(V)-Mediated Arylation/SN2-Displacement of Kukhtin–Ramirez Intermediates
Source: Org Lett. 2022 Oct 24;24(43):8002–7. doi: 10.1021/acs.orglett.2c03201 (PMC9641671; doi:10.1021/acs.orglett.2c03201)
Supplement: Supplementary file 3 — ol2c03201_si_003.zip [file ol2c03201_si_003.zip › FID_8-12/8/8_COSY/pdata/1/pcxac8.AC258_prod_3_1.pdf]

Double quantum filtered COSY Spectrum.

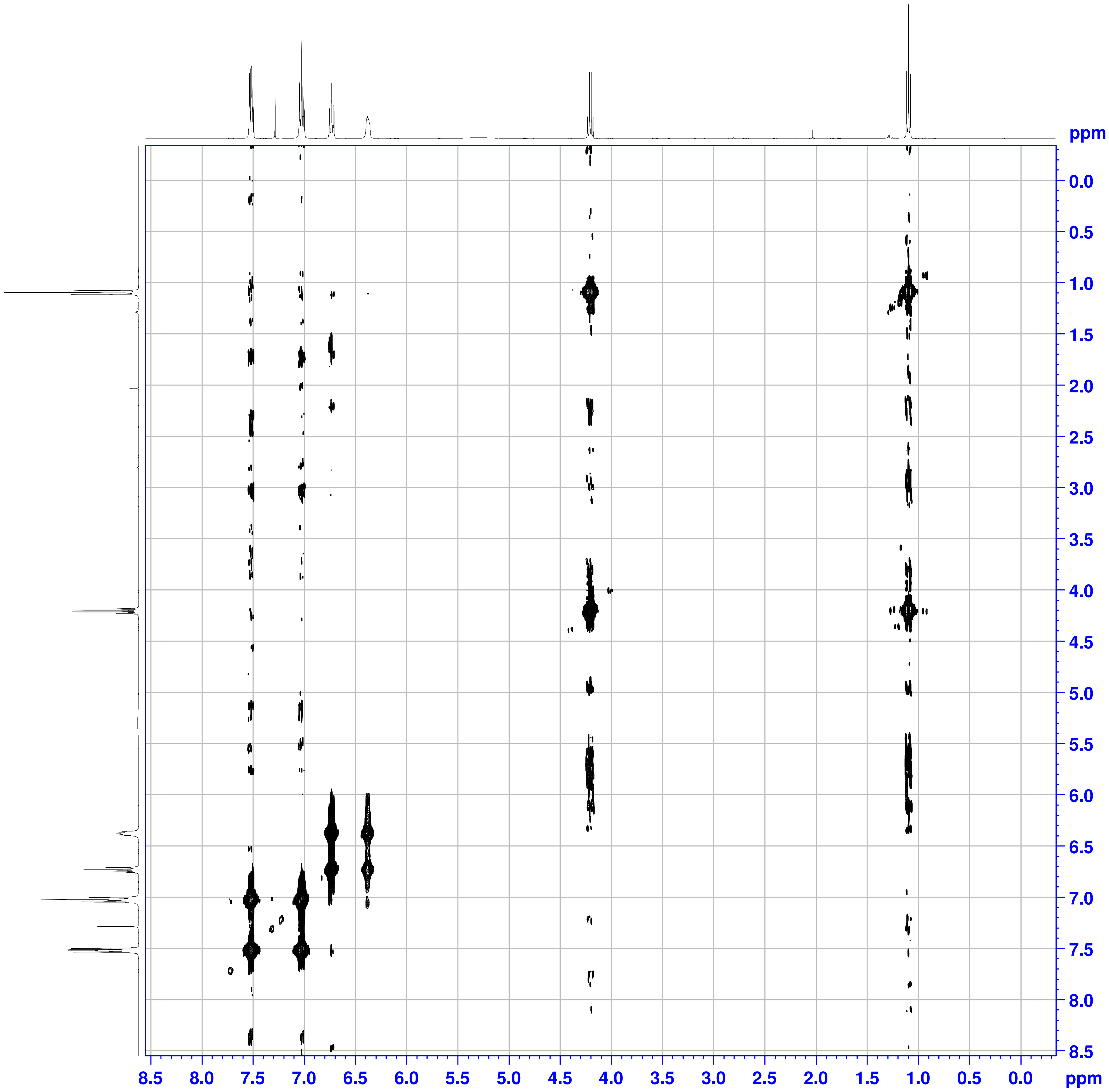

Current Data Parameters
NAME pcxac8.AC258_prod
EXPNO 3
PROCNO 1

F2 - Acquisition Parameters
Date_ 20220203
Time 0.42 h
INSTRUM av3400hd
PROBHD Z122623_0053 (
PULPROG cosygpmfppqf
TD 2048
SOLVENT CDCl3
NS 2
DS 16
SWH 3906.250 Hz
FIDRES 3.814697 Hz
AQ 0.2621440 sec
RG 198.43
DW 128.000 usec
DE 25.31 usec
TE 298.0 K
D0 0.00000300 sec
D1 0.93760413 sec
D11 0.03000000 sec
D12 0.00002000 sec
D13 0.00000400 sec
D16 0.00020000 sec
IN0 0.00025620 sec
TDav 1
SFO1 400.2016425 MHz
NUC1 1H
P1 10.22 usec
P17 2500.00 usec
PLW1 10.00000000 W
PLW10 1.16050005 W
GPNAM[1] SMSQ10.100
GPZ1 16.00 %
GPNAM[2] SMSQ10.100
GPZ2 12.00 %
GPNAM[3] SMSQ10.100
GPZ3 40.00 %
P16 1000.00 usec

F1 - Acquisition parameters
TD 128
SFO1 400.2016 MHz
FIDRES 60.987511 Hz
SW 9.753 ppm
FnMODE QF

F2 - Processing parameters
SI 2048
SF 400.2000000 MHz
WDW SINE
SSB 0
LB 0 Hz
GB 0
PC 1.40

F1 - Processing parameters
SI 512
MC2 QF
SF 400.2000000 MHz
WDW SINE
SSB 0
LB 0 Hz
GB 0
