# Supplementary material for: Modular Synthesis of α,α-Diaryl α-Amino Esters via Bi(V)-Mediated Arylation/SN2-Displacement of Kukhtin–Ramirez Intermediates
Source: Org Lett. 2022 Oct 24;24(43):8002–7. doi: 10.1021/acs.orglett.2c03201 (PMC9641671; doi:10.1021/acs.orglett.2c03201)
Supplement: Supplementary file 3 — ol2c03201_si_003.zip [file ol2c03201_si_003.zip › FID_8-12/8/8_HSQC/pdata/1/pcxac8.AC258_prod_4_1.pdf]

Multiplicity-edited HSQC Spectrum: CH and Me up (black), CH2 down (grey).

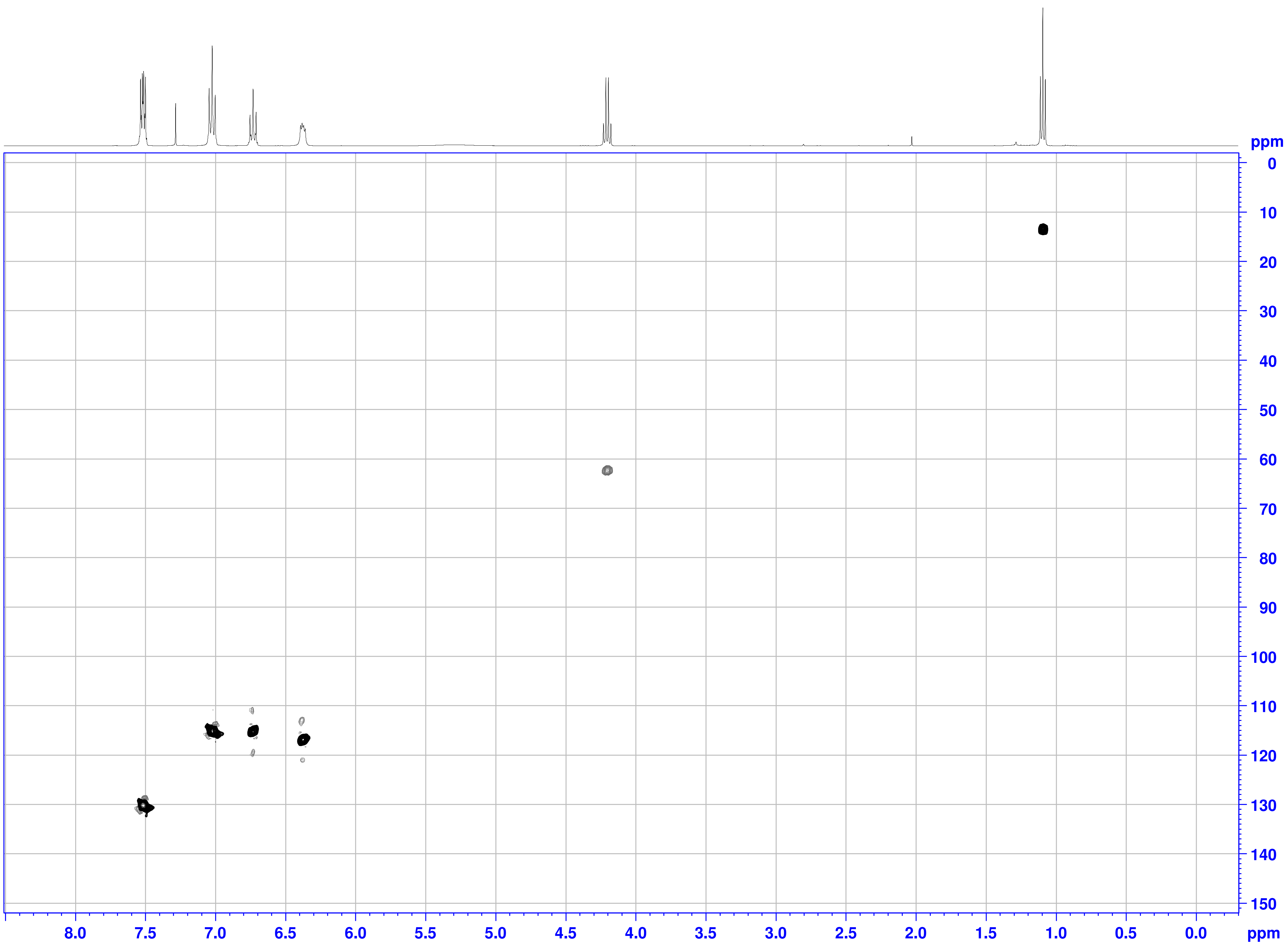

**Current Data Parameters**		
NAME	pcxac8.AC258_prod	
EXPNO	4	
PROCNO	1	
**F2 - Acquisition Parameters**		
Date_	20220203	
Time	0.49	h
INSTRUM	av3400hd	
PROBHD	Z122623_0053 (	
PULPROG	hsqcedetgpsp.3	
TD	1190	
SOLVENT	CDCl3	
NS	2	
DS	32	
SWH	3703.704	Hz
FIDRES	6.224712	Hz
AQ	0.1606500	sec
RG	198.43	
DW	135.000	usec
DE	10.00	usec
TE	298.0	K
CNST2	145.0000000	
D0	0.00000300	sec
D1	1.00000000	sec
D4	0.00172414	sec
D11	0.03000000	sec
D16	0.00020000	sec
D21	0.00360000	sec
IN0	0.00002980	sec
TDav	1	
SFO1	400.2016425	MHz
NUC1	1H	
P1	10.22	usec
P2	20.44	usec
P28	0	usec
PLW1	10.00000000	W
SFO2	100.6379173	MHz
NUC2	13C	
CPDPRG[2	garp4	
P3	10.70	usec
P14	500.00	usec
P31	2119.00	usec
PCPD2	80.00	usec
PLW0	0	W
PLW2	38.00000000	W
PLW12	0.67978001	W
SPNAM[3]	Crp60,0.5,20.1	
SPOAL3	0.500	
SPOFFS3	0	Hz
SPW3	6.64729977	W
SPNAM[18	Crp60_xfilt.2	
SPOAL18	0.500	
SPOFFS18	0	Hz
SPW18	1.28059995	W
GPNAM[1]	SMSQ10.100	
GPZ1	80.00	%
GPNAM[2]	SMSQ10.100	
GPZ2	20.10	%
P16	1000.00	usec
**F1 - Acquisition parameters**		
TD	128	
SFO1	100.6379	MHz
FIDRES	262.164429	Hz
SW	166.722	ppm
FnMODE	Echo-Antiecho	
**F2 - Processing parameters**		
SI	2048	
SF	400.2000000	MHz
WDW	QSINE	
SSB	2	
LB	0	Hz
GB	0	
PC	1.40	
**F1 - Processing parameters**		
SI	512	
MC2	echo-antiecho	
SF	100.6303700	MHz
WDW	QSINE	
SSB	2	
LB	0	Hz
GB	0	
