# Supplementary material for: Modular Synthesis of α,α-Diaryl α-Amino Esters via Bi(V)-Mediated Arylation/SN2-Displacement of Kukhtin–Ramirez Intermediates
Source: Org Lett. 2022 Oct 24;24(43):8002–7. doi: 10.1021/acs.orglett.2c03201 (PMC9641671; doi:10.1021/acs.orglett.2c03201)
Supplement: Supplementary file 3 — ol2c03201_si_003.zip [file ol2c03201_si_003.zip › FID_8-12/9/9_HSQC/pdata/1/pcxac8.AC259_prod_4_1.pdf]

Multiplicity-edited HSQC Spectrum: CH and Me up (black), CH2 down (grey).

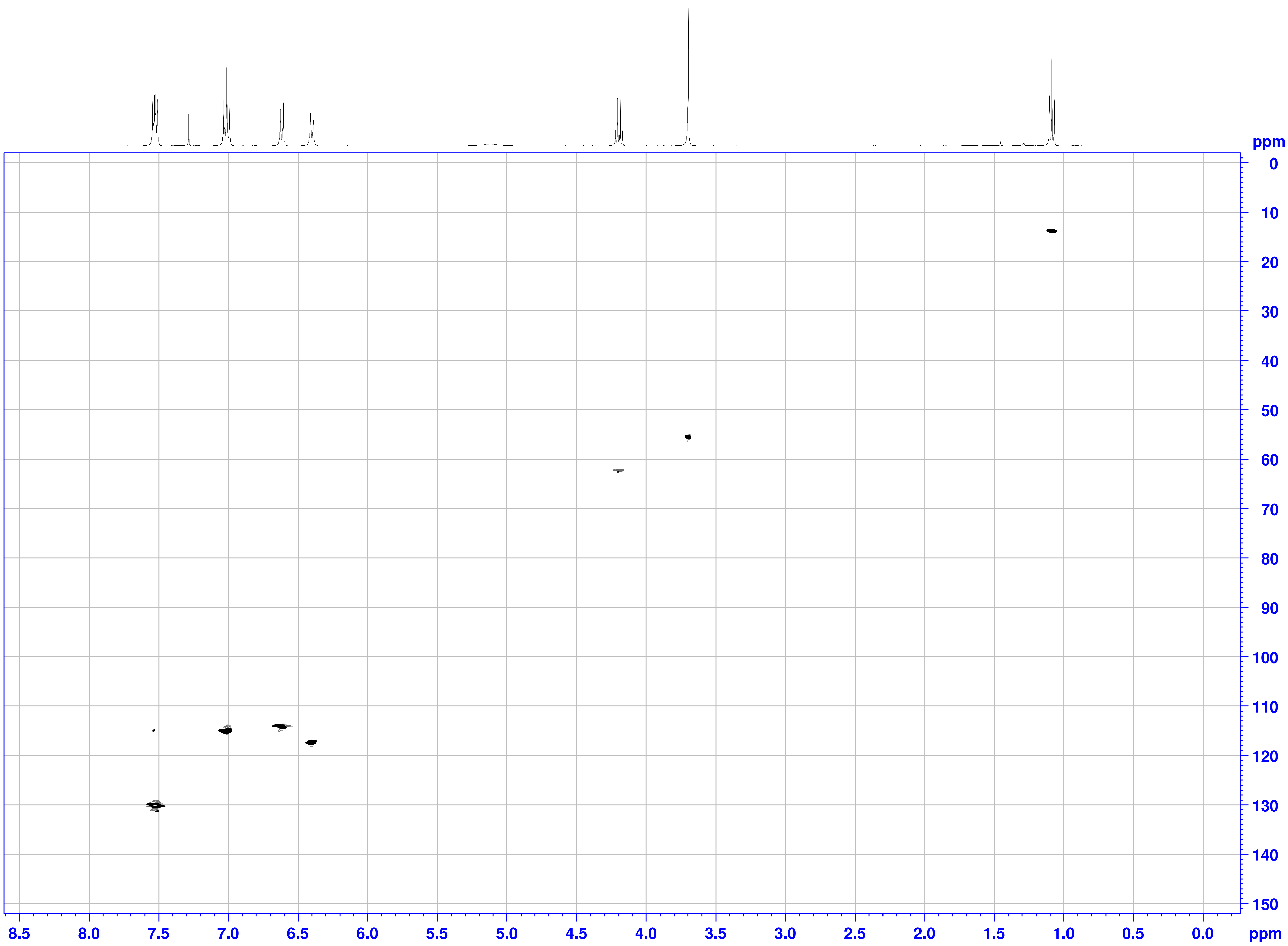

BRUKER

Current Data Parameters
NAME pcxac8.AC259_prod
EXPNO 4
PROCNO 1

F2 - Acquisition Parameters
Date_ 20220202
Time 21.53 h
INSTRUM av3400hd
PROBHD Z122623_0053 (
PULPROG hsqcedetgpsp.3
TD 1190
SOLVENT CDC13
NS 4
DS 32
SWH 3731.343 Hz
FIDRES 6.271165 Hz
AQ 0.1594600 sec
RG 18.01
DW 134.000 usec
DE 10.00 usec
TE 298.0 K
CNST2 145.0000000
D0 0.00000300 sec
D1 1.00000000 sec
D4 0.00172414 sec
D11 0.03000000 sec
D16 0.00020000 sec
D21 0.00360000 sec
IN0 0.00002980 sec
TDav 1
SFO1 400.2016698 MHz
NUC1 1H
P1 10.22 usec
P2 20.44 usec
P28 0 usec
PLW1 10.00000000 W
SFO2 100.6379173 MHz
NUC2 13C
CPDPRG[2 garp4
P3 10.70 usec
P14 500.00 usec
P31 2119.00 usec
PCPD2 80.00 usec
PLW0 0 W
PLW2 38.00000000 W
PLW12 0.67978001 W
SPNAM[3] Crp60,0.5,20.1
SPOAL3 0.500
SPOFFS3 0 Hz
SPW3 6.64729977 W
SPNAM[18 Crp60_xfilt.2
SPOAL18 0.500
SPOFFS18 0 Hz
SPW18 1.28059995 W
GPNAM[1] SMSQ10.100
GPZ1 80.00 %
GPNAM[2] SMSQ10.100
GPZ2 20.10 %
P16 1000.00 usec

F1 - Acquisition parameters
TD 512
SFO1 100.6379 MHz
FIDRES 65.541107 Hz
SW 166.722 ppm
FnMODE Echo-Antiecho

F2 - Processing parameters
SI 2048
SF 400.2000000 MHz
WDW QSINE
SSB 2
LB 0 Hz
GB 0
PC 1.40

F1 - Processing parameters
SI 1024
MC2 echo-antiecho
SF 100.6303700 MHz
WDW QSINE
SSB 2
LB 0 Hz
GB 0
