# Supplementary material for: Modular Synthesis of α,α-Diaryl α-Amino Esters via Bi(V)-Mediated Arylation/SN2-Displacement of Kukhtin–Ramirez Intermediates
Source: Org Lett. 2022 Oct 24;24(43):8002–7. doi: 10.1021/acs.orglett.2c03201 (PMC9641671; doi:10.1021/acs.orglett.2c03201)
Supplement: Supplementary file 4 — ol2c03201_si_004.zip [file ol2c03201_si_004.zip › FID_13-17/13/13_13C/pdata/1/pcxac8.AC272_product_3_1.pdf]

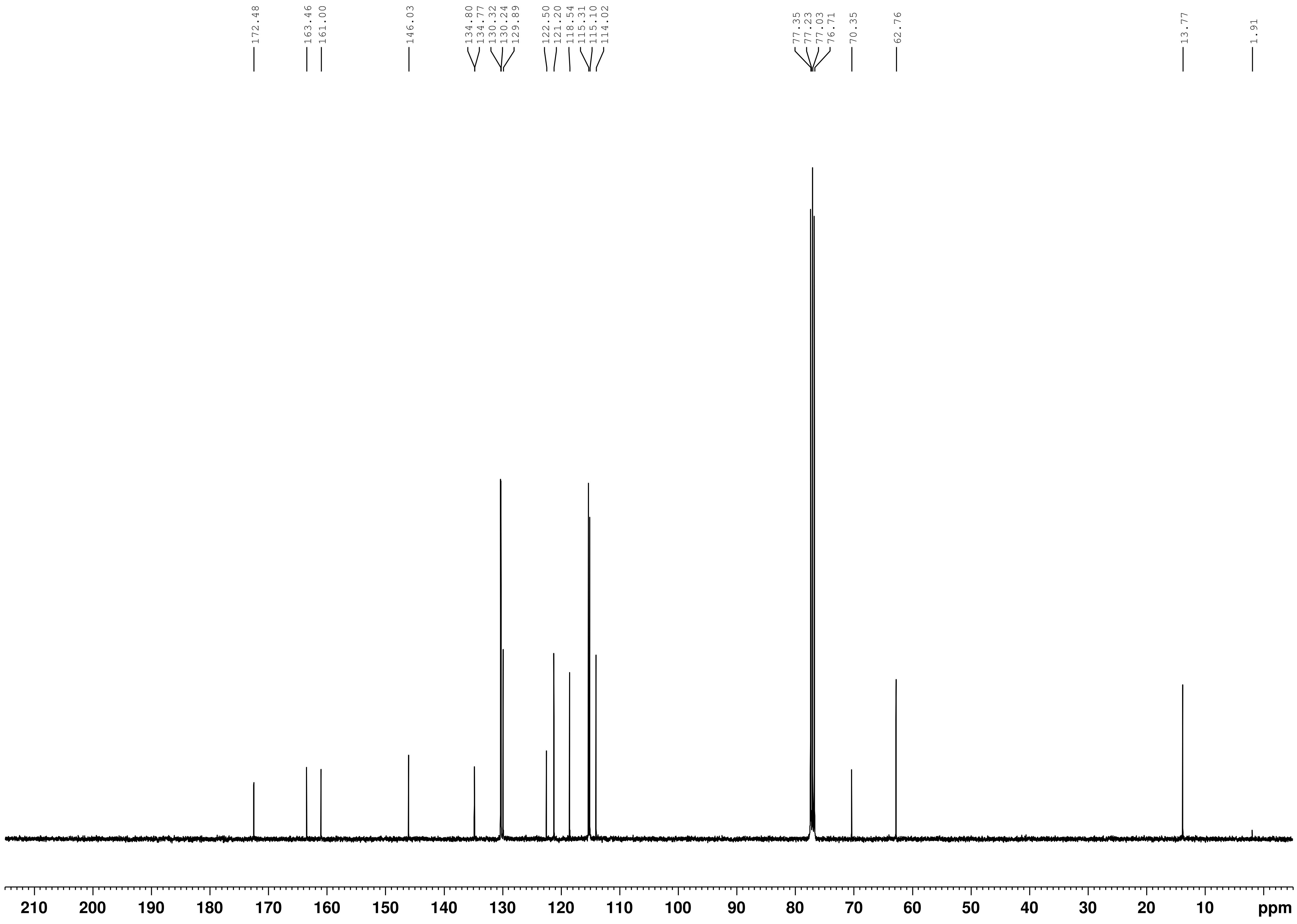


```
Current Data Parameters
NAME     pcxac8.AC272_produc
EXPNO               3
PROCNO              1

F2 - Acquisition Parameters
Date_          20220219
Time              11.01 h
INSTRUM          av3400
PROBHD   Z104450_0225 (
PULPROG          zgpg30
TD                32768
SOLVENT           CDCl3
NS                 4096
DS                    4
SWH          24038.461 Hz
FIDRES        1.467191 Hz
AQ           0.6815744 sec
RG                 2050
DW               20.800 usec
DE                 9.63 usec
TE                298.0 K
D1           1.00000000 sec
D11          0.03000000 sec
TD0                   1
SFO1        100.6087473 MHz
NUC1                13C
P1                 9.75 usec
PLW1        63.68099976 W
SFO2        400.0716003 MHz
NUC2                 1H
CPDPRG[2        waltz16
PCPD2             90.00 usec
PLW2        11.92800045 W
PLW12        0.28863001 W
PLW13        0.14518000 W

F2 - Processing parameters
SI                32768
SF          100.5976815 MHz
WDW                  EM
SSB        0
LB                 1.00 Hz
GB         0
PC                 1.40
```
