# Supplementary material for: Modular Synthesis of α,α-Diaryl α-Amino Esters via Bi(V)-Mediated Arylation/SN2-Displacement of Kukhtin–Ramirez Intermediates
Source: Org Lett. 2022 Oct 24;24(43):8002–7. doi: 10.1021/acs.orglett.2c03201 (PMC9641671; doi:10.1021/acs.orglett.2c03201)
Supplement: Supplementary file 4 — ol2c03201_si_004.zip [file ol2c03201_si_004.zip › FID_13-17/13/13_HMBC/pdata/1/pcxac8.AC272_product_6_1.pdf]

HMBC spectrum: very low level one-bond artefacts may be observed.

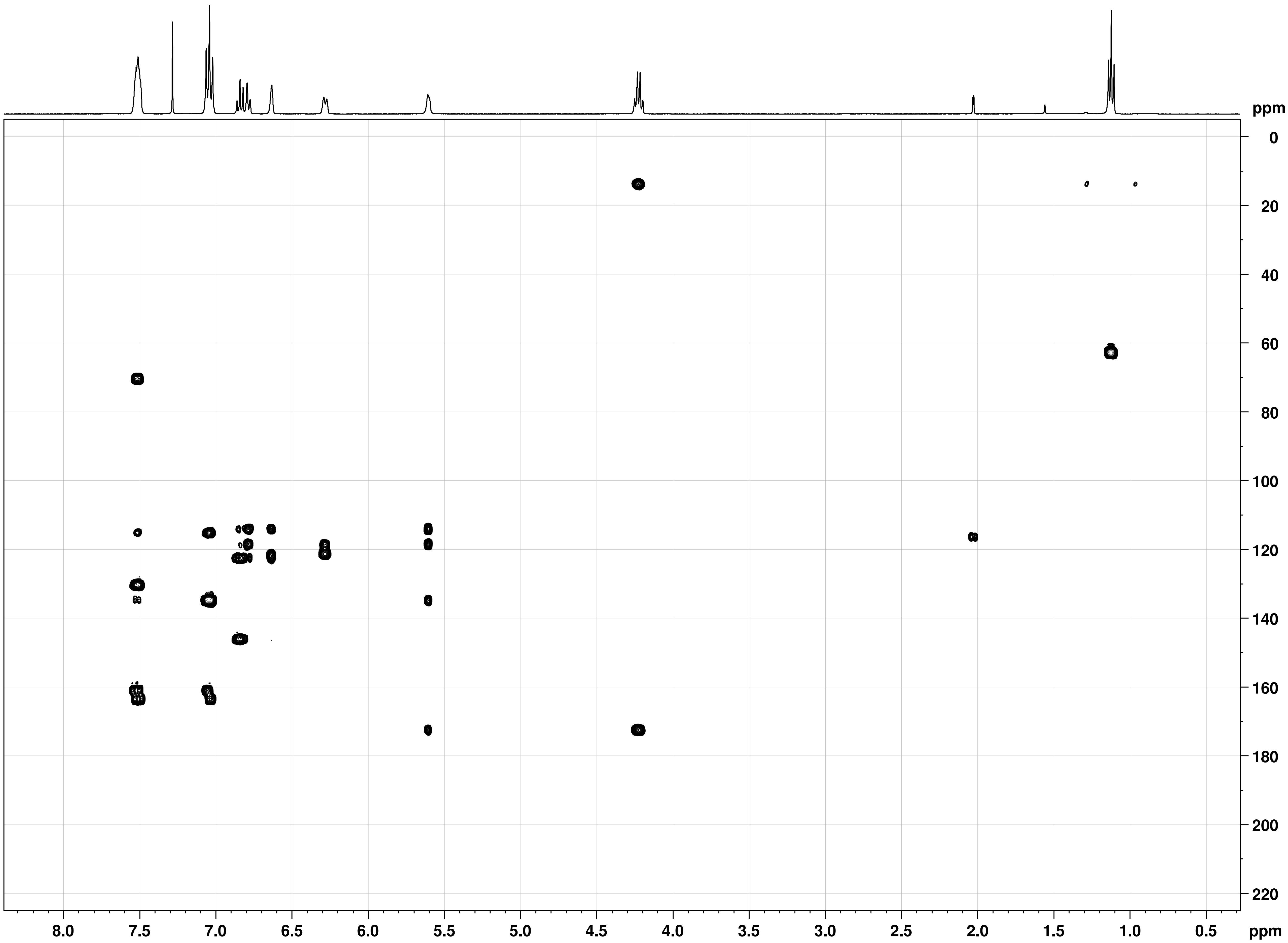

Current Data Parameters
NAME pcxac8.AC272_product
EXPNO 6
PROCNO 1

F2 - Acquisition Parameters
Date_ 20220219
Time 11.53 h
INSTRUM av3400
PROBHD Z104450_0225 (
PULPROG hmbcetgpl3nd
TD 1190
SOLVENT CDC13
NS 8
DS 16
SWH 3246.753 Hz
FIDRES 5.456728 Hz
AQ 0.1832600 sec
RG 2050
DW 154.000 usec
DE 6.50 usec
TE 298.0 K
CNST6 120.0000000
CNST7 210.0000000
CNST13 8.5000000
D0 0.00000300 sec
D1 1.00000000 sec
D6 0.05882353 sec
D16 0.00020000 sec
IN0 0.00002160 sec
TDav 1
SFO1 400.0717337 MHz
NUC1 1H
P1 14.00 usec
P2 28.00 usec
PLW1 11.92800045 W
SFO2 100.6087473 MHz
NUC2 13C
P3 9.75 usec
P24 2000.00 usec
PLW2 63.68099976 W
SPNAM[7] Crp60comp.4
SPOAL7 0.500
SPOFFS7 0 Hz
SPW7 9.24930000 W
GPNAM[1] SMSQ10.100
GPZ1 80.00 %
GPNAM[3] SMSQ10.100
GPZ3 14.00 %
GPNAM[4] SMSQ10.100
GPZ4 -8.00 %
GPNAM[5] SMSQ10.100
GPZ5 -4.00 %
GPNAM[6] SMSQ10.100
GPZ6 -2.00 %
P16 1000.00 usec

F1 - Acquisition parameters
TD 128
SFO1 100.6087 MHz
FIDRES 361.689819 Hz
SW 230.081 ppm
FnMODE Echo-Antiecho

F2 - Processing parameters
SI 2048
SF 400.0700000 MHz
WDW SINE
SSB 0
LB 0 Hz
GB 0
PC 1.40

F1 - Processing parameters
SI 512
MC2 echo-antiecho
SF 100.5976815 MHz
WDW QSINE
SSB 2
LB 0 Hz
GB 0
