# Supplementary material for: Modular Synthesis of α,α-Diaryl α-Amino Esters via Bi(V)-Mediated Arylation/SN2-Displacement of Kukhtin–Ramirez Intermediates
Source: Org Lett. 2022 Oct 24;24(43):8002–7. doi: 10.1021/acs.orglett.2c03201 (PMC9641671; doi:10.1021/acs.orglett.2c03201)
Supplement: Supplementary file 4 — ol2c03201_si_004.zip [file ol2c03201_si_004.zip › FID_13-17/13/13_HSQC/pdata/1/pcxac8.AC272_product_5_1.pdf]

Multiplicity-edited HSQC Spectrum: CH and Me up (black), CH2 down (grey).

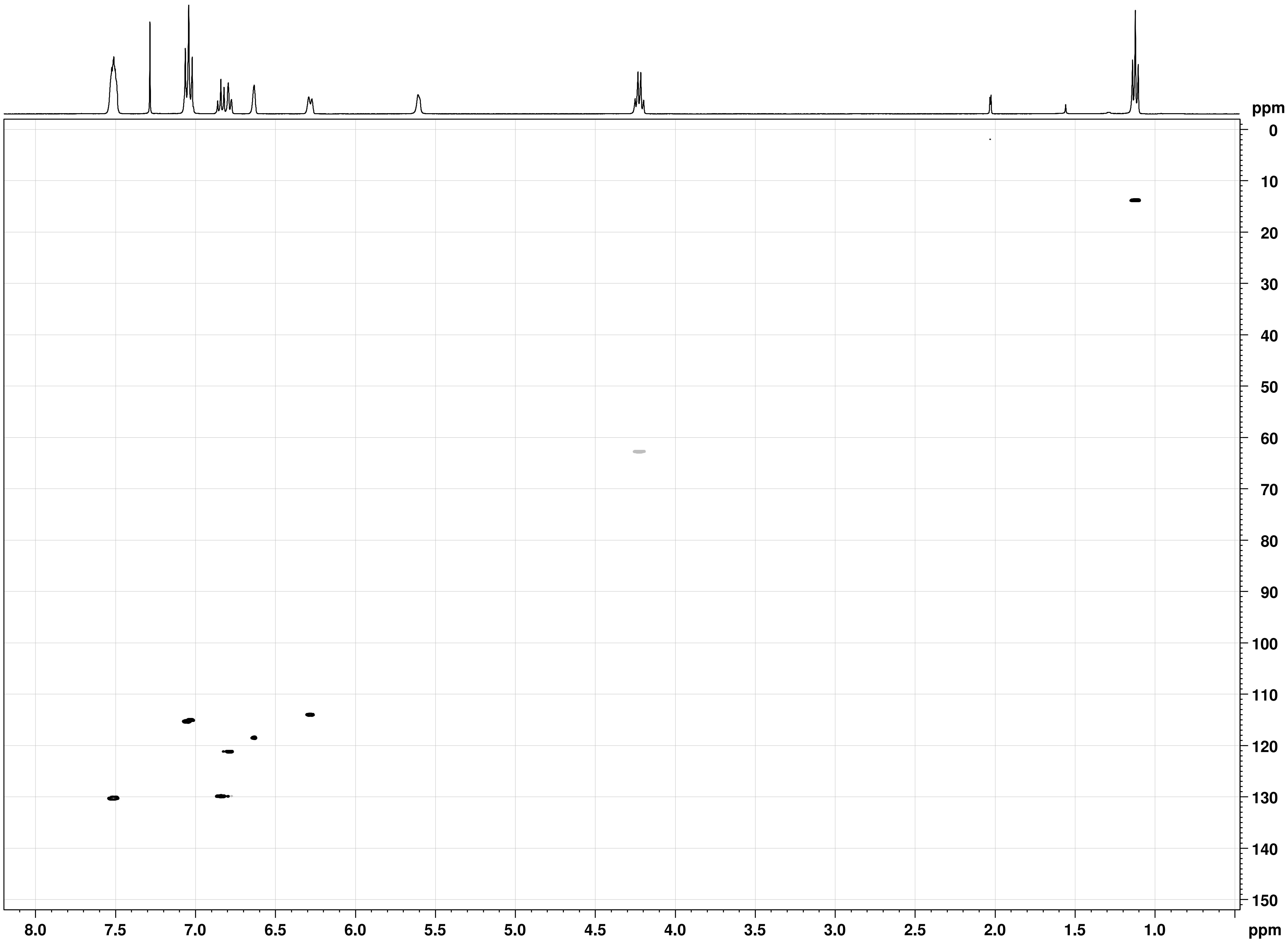


```
Current Data Parameters
NAME      pcxac8.AC272_product
EXPNO                    5
PROCNO                   1

F2 - Acquisition Parameters
Date_           20220219
Time               11.10 h
INSTRUM           av3400
PROBHD   Z104450_0225 (
PULPROG  hsqcedetgpsp.3
TD                  1190
SOLVENT            CDCl3
NS                     4
DS                    32
SWH             3246.753 Hz
FIDRES          5.456728 Hz
AQ             0.1832600 sec
RG                  2050
DW               154.000 usec
DE                  6.50 usec
TE                 298.0 K
CNST2       145.0000000
D0           0.00000300 sec
D1           1.00000000 sec
D4           0.00172414 sec
D11          0.03000000 sec
D16          0.00020000 sec
D21          0.00360000 sec
IN0          0.00002980 sec
TDav                   1
SFO1        400.0717337 MHz
NUC1                  1H
P1                 14.00 usec
P2                 28.00 usec
P28              1000.00 usec
PLW1         11.92800045 W
SFO2        100.6052263 MHz
NUC2                 13C
CPDPRG[2           garp4
P3                  9.75 usec
P14               500.00 usec
P31              2119.00 usec
PCPD2              80.00 usec
PLW0      0 W
PLW2         63.68099976 W
PLW12         0.94588000 W
SPNAM[3] Crp60,0.5,20.1
SPOAL3             0.500
SPOFFS3  0 Hz
SPW3          9.24930000 W
SPNAM[18  Crp60_xfilt.2
SPOAL18            0.500
SPOFFS18 0 Hz
SPW18         1.78180003 W
GPNAM[1]      SMSQ10.100
GPZ1               80.00 %
GPNAM[2]      SMSQ10.100
GPZ2               20.10 %
P16              1000.00 usec

F1 - Acquisition parameters
TD                   512
SFO1            100.6052 MHz
FIDRES         65.541107 Hz
SW               166.776 ppm
FnMODE     Echo-Antiecho

F2 - Processing parameters
SI                  2048
SF           400.0700000 MHz
WDW                QSINE
SSB                    2
LB        0 Hz
GB        0
PC                  1.40

F1 - Processing parameters
SI                  1024
MC2         echo-antiecho
SF           100.5976815 MHz
WDW                QSINE
SSB                    2
LB        0 Hz
GB        0
```
