# Supplementary material for: Modular Synthesis of α,α-Diaryl α-Amino Esters via Bi(V)-Mediated Arylation/SN2-Displacement of Kukhtin–Ramirez Intermediates
Source: Org Lett. 2022 Oct 24;24(43):8002–7. doi: 10.1021/acs.orglett.2c03201 (PMC9641671; doi:10.1021/acs.orglett.2c03201)
Supplement: Supplementary file 4 — ol2c03201_si_004.zip [file ol2c03201_si_004.zip › FID_13-17/15/15_19F/4/pdata/1/pcxac8.AC271_product2_4_1.pdf]

Processed with back linear prediction to eliminate baseline artefacts: broad peaks may be attenuated. Standard data in file 100004

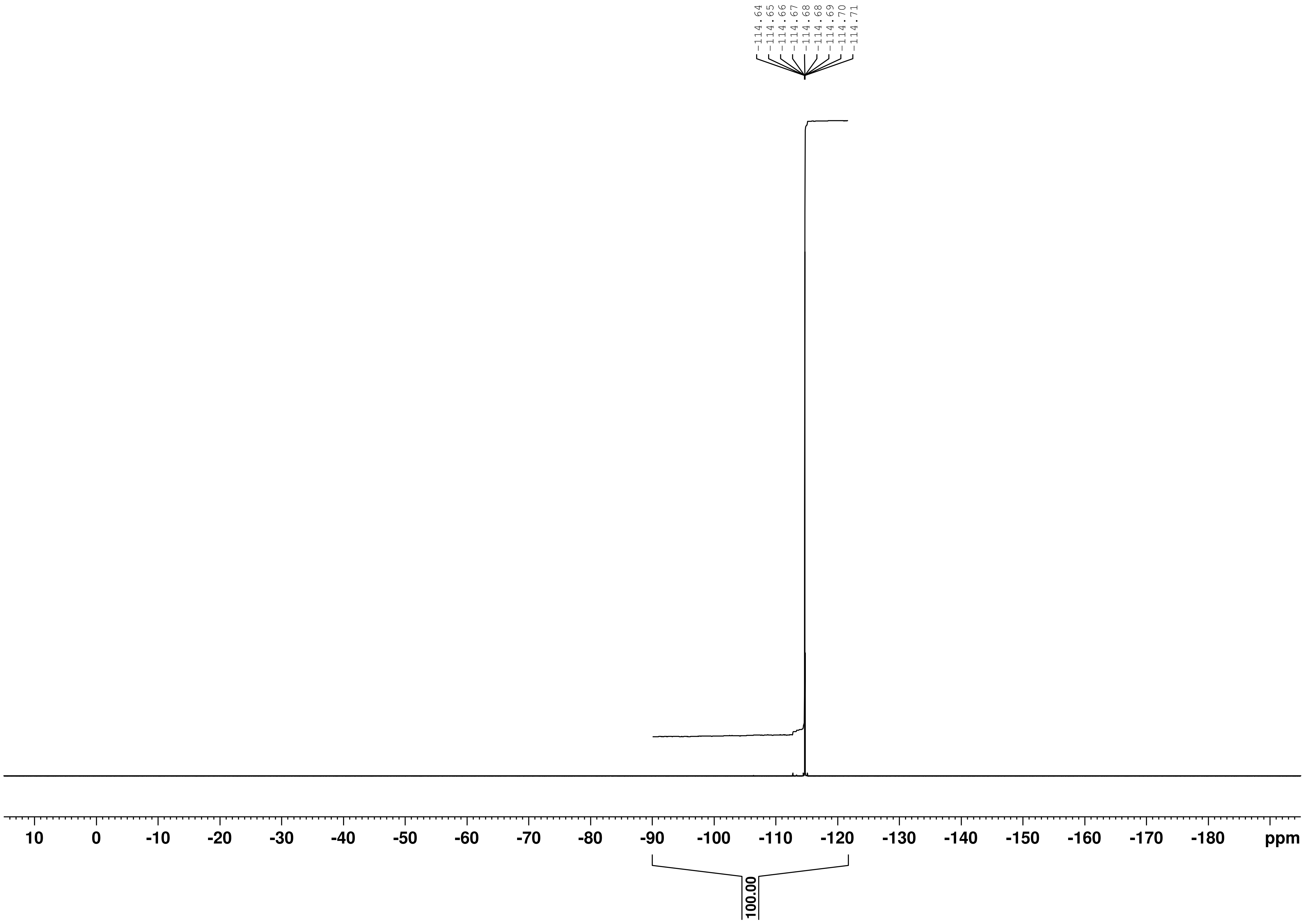


```
Current Data Parameters
NAME     pcxac8.AC271_product2
EXPNO                    4
PROCNO                   1

F2 - Acquisition Parameters
Date_          20220219
Time                8.07 h
INSTRUM           av3400
PROBHD   Z104450_0225 (
PULPROG           zgflqn
TD                261992
SOLVENT            CDCl3
NS                    16
DS                     4
SWH            85227.273 Hz
FIDRES          0.650610 Hz
AQ             1.5370197 sec
RG                  1030
DW                 5.867 usec
DE                  6.77 usec
TE                 298.0 K
D1            1.50000000 sec
TD0                    1
SFO1         376.4080300 MHz
NUC1                 19F
P1                 13.30 usec
PLW1         16.11100006 W

F2 - Processing parameters
SI                262144
SF           376.4419098 MHz
WDW                   EM
SSB      0
LB                  1.00 Hz
GB       0
PC                  1.00
```
