# Supplementary material for: Modular Synthesis of α,α-Diaryl α-Amino Esters via Bi(V)-Mediated Arylation/SN2-Displacement of Kukhtin–Ramirez Intermediates
Source: Org Lett. 2022 Oct 24;24(43):8002–7. doi: 10.1021/acs.orglett.2c03201 (PMC9641671; doi:10.1021/acs.orglett.2c03201)
Supplement: Supplementary file 4 — ol2c03201_si_004.zip [file ol2c03201_si_004.zip › FID_13-17/15/15_COSY/pdata/1/pcxac8.AC271_product2_3_1.pdf]

Double quantum filtered COSY Spectrum.

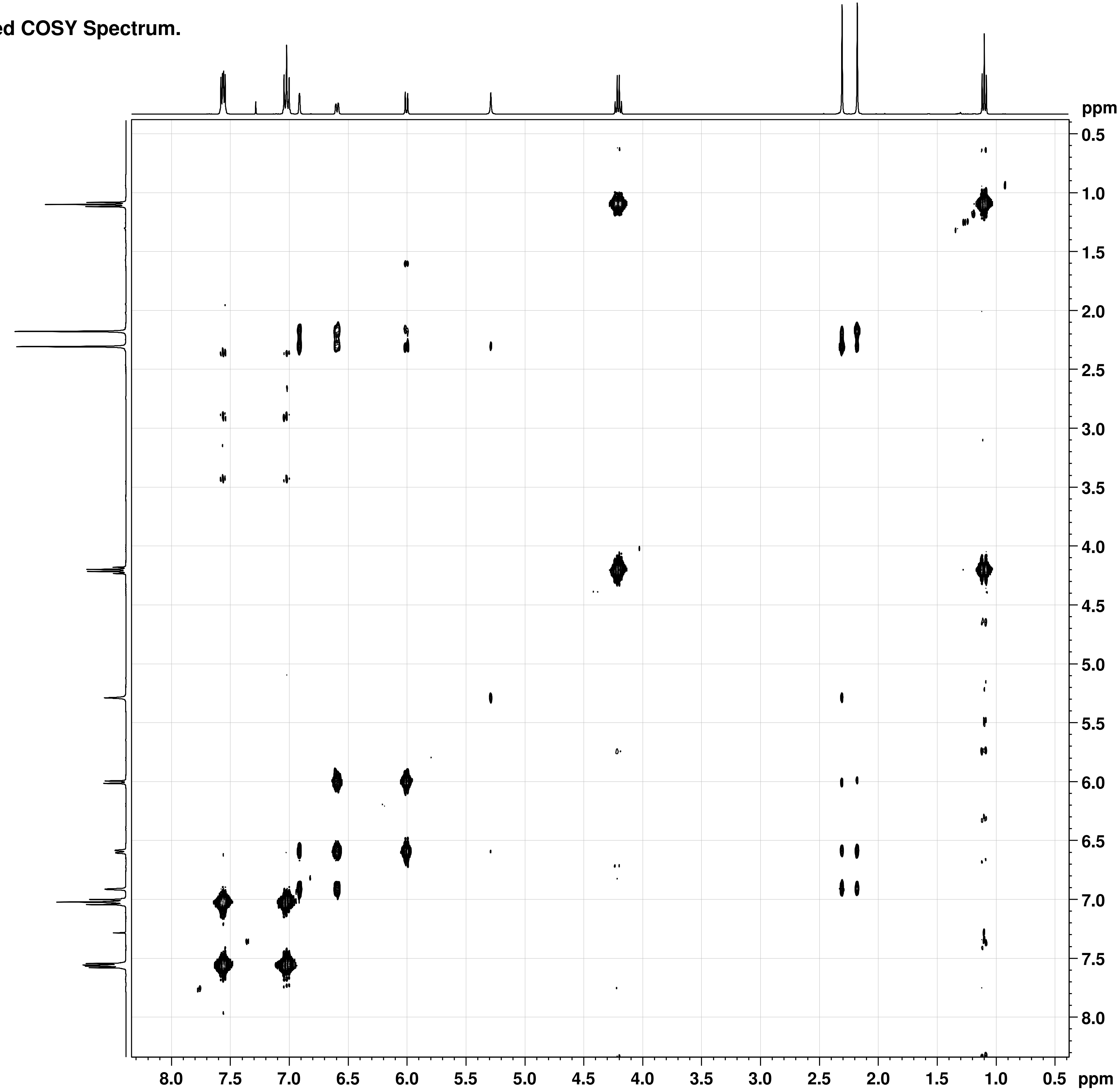


```
Current Data Parameters
NAME     pcxac8.AC271_product2
EXPNO                        3
PROCNO                       1

F2 - Acquisition Parameters
Date_              20220219
Time                   7.59 h
INSTRUM              av3400
PROBHD   Z104450_0225 (
PULPROG     cosygpmfppqf
TD                     2048
SOLVENT               CDCl3
NS                        2
DS                       16
SWH                3511.236 Hz
FIDRES             3.428941 Hz
AQ                0.2916352 sec
RG                     2050
DW                  142.400 usec
DE                     9.15 usec
TE                    298.0 K
D0               0.00000300 sec
D1               0.90429002 sec
D11              0.03000000 sec
D12              0.00002000 sec
D13              0.00000400 sec
D16              0.00020000 sec
IN0              0.00028480 sec
TDav                      1
SFO1            400.0717446 MHz
NUC1                     1H
P1                    14.00 usec
P17                 2500.00 usec
PLW1            11.92800045 W
PLW10            2.59770012 W
GPNAM[1]        SMSQ10.100
GPZ1                  16.00 %
GPNAM[2]        SMSQ10.100
GPZ2                  12.00 %
GPNAM[3]        SMSQ10.100
GPZ3                  40.00 %
P16                 1000.00 usec

F1 - Acquisition parameters
TD                      128
SFO1               400.0717 MHz
FIDRES            54.863060 Hz
SW                    8.777 ppm
FnMODE                   QF

F2 - Processing parameters
SI                     2048
SF              400.0700000 MHz
WDW                    SINE
SSB       0
LB        0 Hz
GB        0
PC                     1.40

F1 - Processing parameters
SI                      512
MC2                      QF
SF              400.0700000 MHz
WDW                    SINE
SSB       0
LB        0 Hz
GB        0
```
