# Supplementary material for: Modular Synthesis of α,α-Diaryl α-Amino Esters via Bi(V)-Mediated Arylation/SN2-Displacement of Kukhtin–Ramirez Intermediates
Source: Org Lett. 2022 Oct 24;24(43):8002–7. doi: 10.1021/acs.orglett.2c03201 (PMC9641671; doi:10.1021/acs.orglett.2c03201)
Supplement: Supplementary file 4 — ol2c03201_si_004.zip [file ol2c03201_si_004.zip › FID_13-17/15/15_HMBC/pdata/1/pcxac8.AC271_product2_6_1.pdf]

Multiplicity-edited HSQC Spectrum: CH and Me up (black), CH2 down (grey).

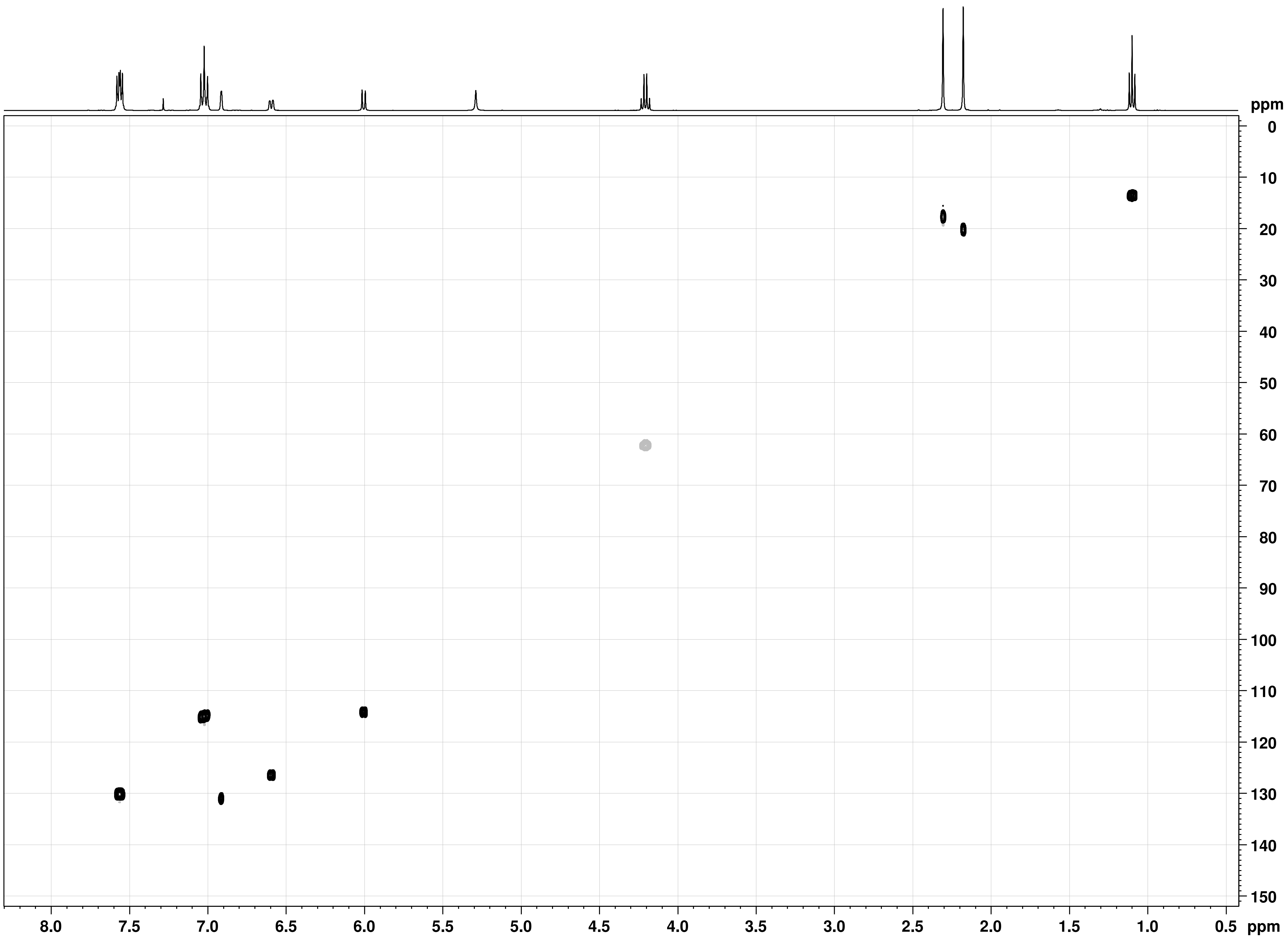


```
Current Data Parameters
NAME     pcxac8.AC271_product2
EXPNO                       6
PROCNO                      1

F2 - Acquisition Parameters
Date_              20220219
Time                   8.52 h
INSTRUM              av3400
PROBHD   Z104450_0225 (
PULPROG  hsqcedetgpsp.3
TD                     1190
SOLVENT               CDCl3
NS                        2
DS                       32
SWH                3311.258 Hz
FIDRES             5.565140 Hz
AQ                0.1796900 sec
RG                     2050
DW                  151.000 usec
DE                     6.50 usec
TE                    298.0 K
CNST2           145.0000000
D0               0.00000300 sec
D1               1.00000000 sec
D4               0.00172414 sec
D11              0.03000000 sec
D16              0.00020000 sec
D21              0.00360000 sec
IN0              0.00002980 sec
TDav                      1
SFO1            400.0717446 MHz
NUC1                     1H
P1                    14.00 usec
P2                    28.00 usec
P28                 1000.00 usec
PLW1            11.92800045 W
SFO2            100.6052263 MHz
NUC2                    13C
CPDPRG[2              garp4
P3                     9.75 usec
P14                  500.00 usec
P31                 2119.00 usec
PCPD2                 80.00 usec
PLW0     0 W
PLW2            63.68099976 W
PLW12            0.94588000 W
SPNAM[3] Crp60,0.5,20.1
SPOAL3                0.500
SPOFFS3  0 Hz
SPW3             9.24930000 W
SPNAM[18  Crp60_xfilt.2
SPOAL18               0.500
SPOFFS18 0 Hz
SPW18            1.78180003 W
GPNAM[1]     SMSQ10.100
GPZ1                  80.00 %
GPNAM[2]     SMSQ10.100
GPZ2                  20.10 %
P16                 1000.00 usec

F1 - Acquisition parameters
TD                      128
SFO1               100.6052 MHz
FIDRES           262.164429 Hz
SW                  166.776 ppm
FnMODE    Echo-Antiecho

F2 - Processing parameters
SI                     2048
SF              400.0700000 MHz
WDW                   QSINE
SSB                       2
LB       0 Hz
GB       0
PC                     1.40

F1 - Processing parameters
SI                      512
MC2          echo-antiecho
SF              100.5976815 MHz
WDW                   QSINE
SSB                       2
LB       0 Hz
GB       0
```
