# Supplementary material for: Modular Synthesis of α,α-Diaryl α-Amino Esters via Bi(V)-Mediated Arylation/SN2-Displacement of Kukhtin–Ramirez Intermediates
Source: Org Lett. 2022 Oct 24;24(43):8002–7. doi: 10.1021/acs.orglett.2c03201 (PMC9641671; doi:10.1021/acs.orglett.2c03201)
Supplement: Supplementary file 4 — ol2c03201_si_004.zip [file ol2c03201_si_004.zip › FID_13-17/15/15_HSQC/pdata/1/pcxac8.AC271_product2_5_1.pdf]

Multiplicity-edited HSQC Spectrum: CH and Me up (black), CH2 down (grey).

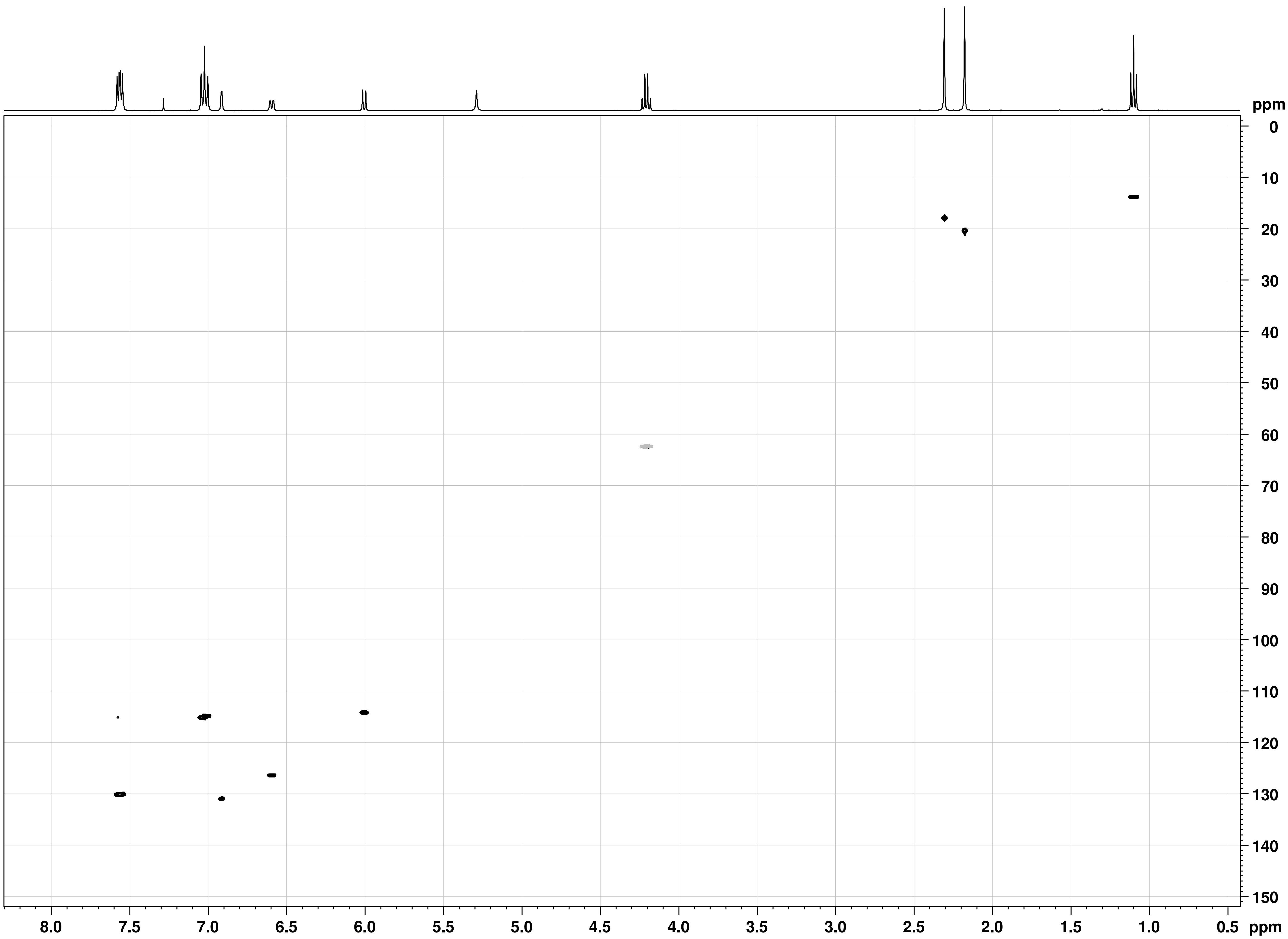


```
Current Data Parameters
NAME      pcxac8.AC271_product2
EXPNO                     5
PROCNO                    1

F2 - Acquisition Parameters
Date_             20220219
Time                  8.09 h
INSTRUM             av3400
PROBHD    Z104450_0225 (
PULPROG   hsqcedetgpsp.3
TD                    1190
SOLVENT              CDCl3
NS                       4
DS                      32
SWH               3311.258 Hz
FIDRES            5.565140 Hz
AQ               0.1796900 sec
RG                    2050
DW                 151.000 usec
DE                    6.50 usec
TE                   298.0 K
CNST2          145.0000000
D0              0.00000300 sec
D1              1.00000000 sec
D4              0.00172414 sec
D11             0.03000000 sec
D16             0.00020000 sec
D21             0.00360000 sec
IN0             0.00002980 sec
TDav                     1
SFO1           400.0717446 MHz
NUC1                    1H
P1                   14.00 usec
P2                   28.00 usec
P28                1000.00 usec
PLW1           11.92800045 W
SFO2           100.6052263 MHz
NUC2                   13C
CPDPRG[2             garp4
P3                    9.75 usec
P14                 500.00 usec
P31                2119.00 usec
PCPD2                80.00 usec
PLW0      0 W
PLW2           63.68099976 W
PLW12           0.94588000 W
SPNAM[3] Crp60,0.5,20.1
SPOAL3               0.500
SPOFFS3   0 Hz
SPW3            9.24930000 W
SPNAM[18  Crp60_xfilt.2
SPOAL18              0.500
SPOFFS18 0 Hz
SPW18           1.78180003 W
GPNAM[1]       SMSQ10.100
GPZ1                 80.00 %
GPNAM[2]       SMSQ10.100
GPZ2                 20.10 %
P16                1000.00 usec

F1 - Acquisition parameters
TD                     512
SFO1              100.6052 MHz
FIDRES           65.541107 Hz
SW                 166.776 ppm
FnMODE      Echo-Antiecho

F2 - Processing parameters
SI                    2048
SF             400.0700000 MHz
WDW                  QSINE
SSB                      2
LB        0 Hz
GB        0
PC                    1.40

F1 - Processing parameters
SI                    1024
MC2         echo-antiecho
SF             100.5976815 MHz
WDW                  QSINE
SSB                      2
LB        0 Hz
GB        0
```
