# Supplementary material for: Modular Synthesis of α,α-Diaryl α-Amino Esters via Bi(V)-Mediated Arylation/SN2-Displacement of Kukhtin–Ramirez Intermediates
Source: Org Lett. 2022 Oct 24;24(43):8002–7. doi: 10.1021/acs.orglett.2c03201 (PMC9641671; doi:10.1021/acs.orglett.2c03201)
Supplement: Supplementary file 4 — ol2c03201_si_004.zip [file ol2c03201_si_004.zip › FID_13-17/16/16_19F/2/pdata/1/pcxac8.AC238_product_2_1.pdf]

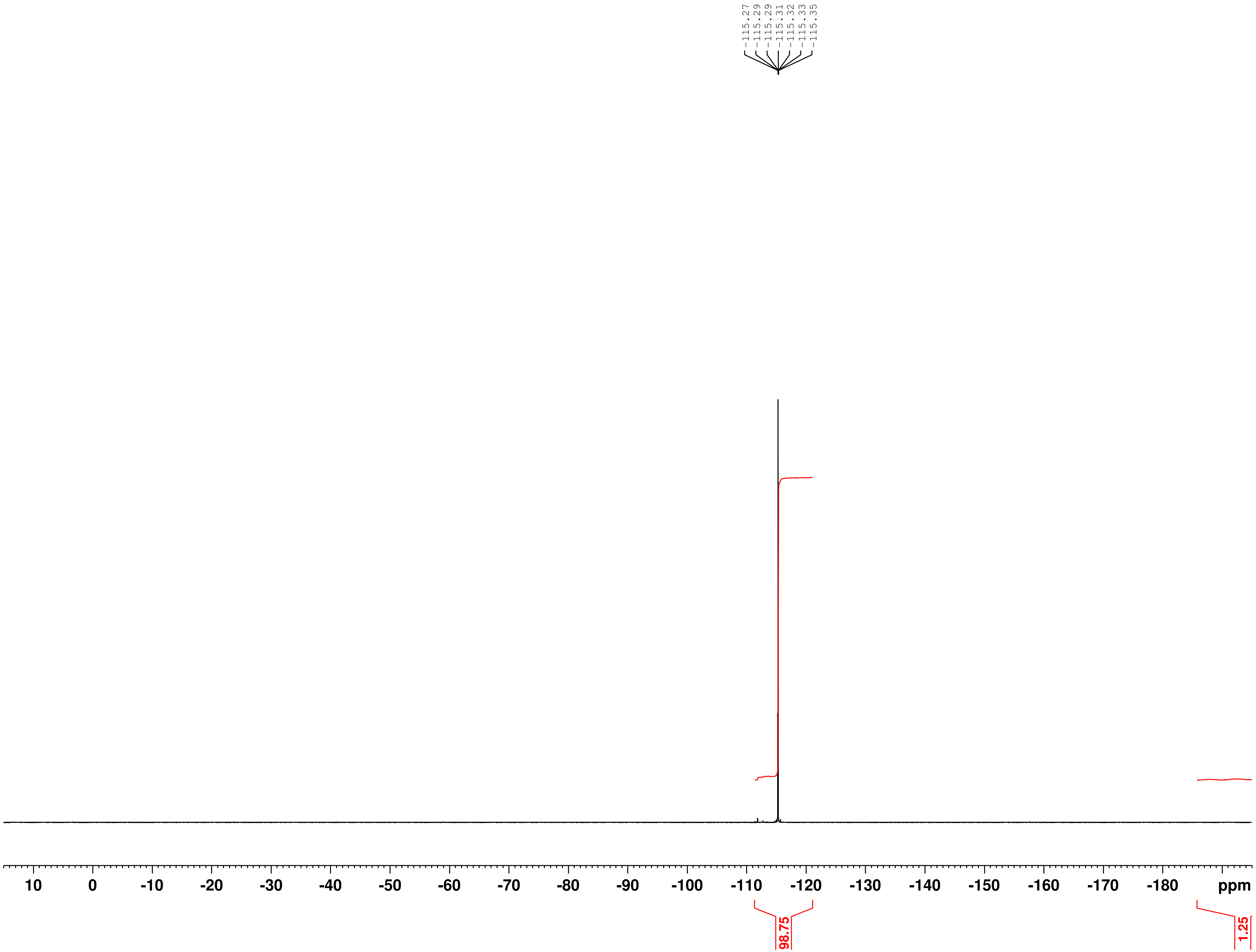

Current Data Parameters
NAME pcxac8.AC238_product
EXPNO 2
PROCNO 1

F2 - Acquisition Parameters
Date_ 20211214
Time 11.18 h
INSTRUM av3400hd
PROBHD Z122623_0053 (
PULPROG zgflqn
TD 261896
SOLVENT CDCl3
NS 16
DS 4
SWH 85227.273 Hz
FIDRES 0.650848 Hz
AQ 1.5364566 sec
RG 12.56
DW 5.867 usec
DE 18.56 usec
TE 298.0 K
D1 1.50000000 sec
TD0 1
SFO1 376.5303412 MHz
NUC1 19F
P1 13.80 usec
PLW1 8.69999981 W

F2 - Processing parameters
SI 262144
SF 376.5642320 MHz
WDW EM
SSB 0
LB 1.00 Hz
GB 0
PC 1.00
