# Supplementary material for: Modular Synthesis of α,α-Diaryl α-Amino Esters via Bi(V)-Mediated Arylation/SN2-Displacement of Kukhtin–Ramirez Intermediates
Source: Org Lett. 2022 Oct 24;24(43):8002–7. doi: 10.1021/acs.orglett.2c03201 (PMC9641671; doi:10.1021/acs.orglett.2c03201)
Supplement: Supplementary file 4 — ol2c03201_si_004.zip [file ol2c03201_si_004.zip › FID_13-17/16/16_1H/pdata/1/pcxac8.AC238_productdry_2_1.pdf]

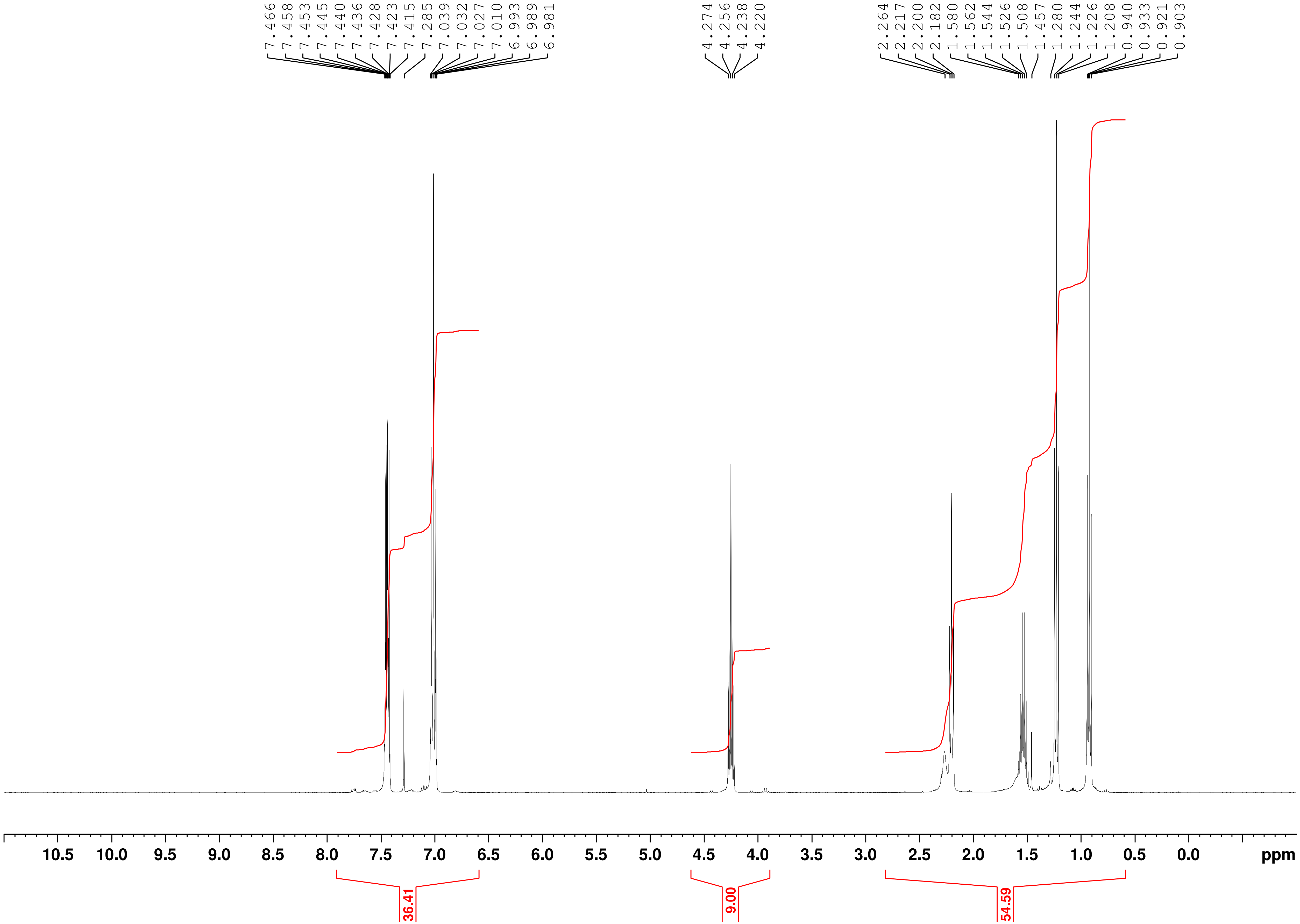


```
Current Data Parameters
NAME      pcxac8.AC238_productdry
EXPNO                   2
PROCNO                  1

F2 - Acquisition Parameters
Date_            20211214
Time                21.07 h
INSTRUM           av3400hd
PROBHD   Z122623_0053 (
PULPROG              zg30
TD                  65536
SOLVENT             CDCl3
NS                     16
DS                      2
SWH              8223.685 Hz
FIDRES           0.250967 Hz
AQ              3.9845889 sec
RG                 140.92
DW                 60.800 usec
DE                  17.93 usec
TE                  298.0 K
D1             1.00000000 sec
TD0                     1
SFO1          400.2024714 MHz
NUC1                   1H
P1                  10.22 usec
PLW1          10.00000000 W

F2 - Processing parameters
SI                  65536
SF            400.2000000 MHz
WDW                    EM
SSB      0
LB                   0.30 Hz
GB       0
PC                   1.00
```
