# Supplementary material for: Modular Synthesis of α,α-Diaryl α-Amino Esters via Bi(V)-Mediated Arylation/SN2-Displacement of Kukhtin–Ramirez Intermediates
Source: Org Lett. 2022 Oct 24;24(43):8002–7. doi: 10.1021/acs.orglett.2c03201 (PMC9641671; doi:10.1021/acs.orglett.2c03201)
Supplement: Supplementary file 4 — ol2c03201_si_004.zip [file ol2c03201_si_004.zip › FID_13-17/16/16_HSQC/pdata/1/pcxac8.AC238_productdry_3_1.pdf]

Multiplicity-edited HSQC Spectrum: CH and Me up (black), CH2 down (grey).

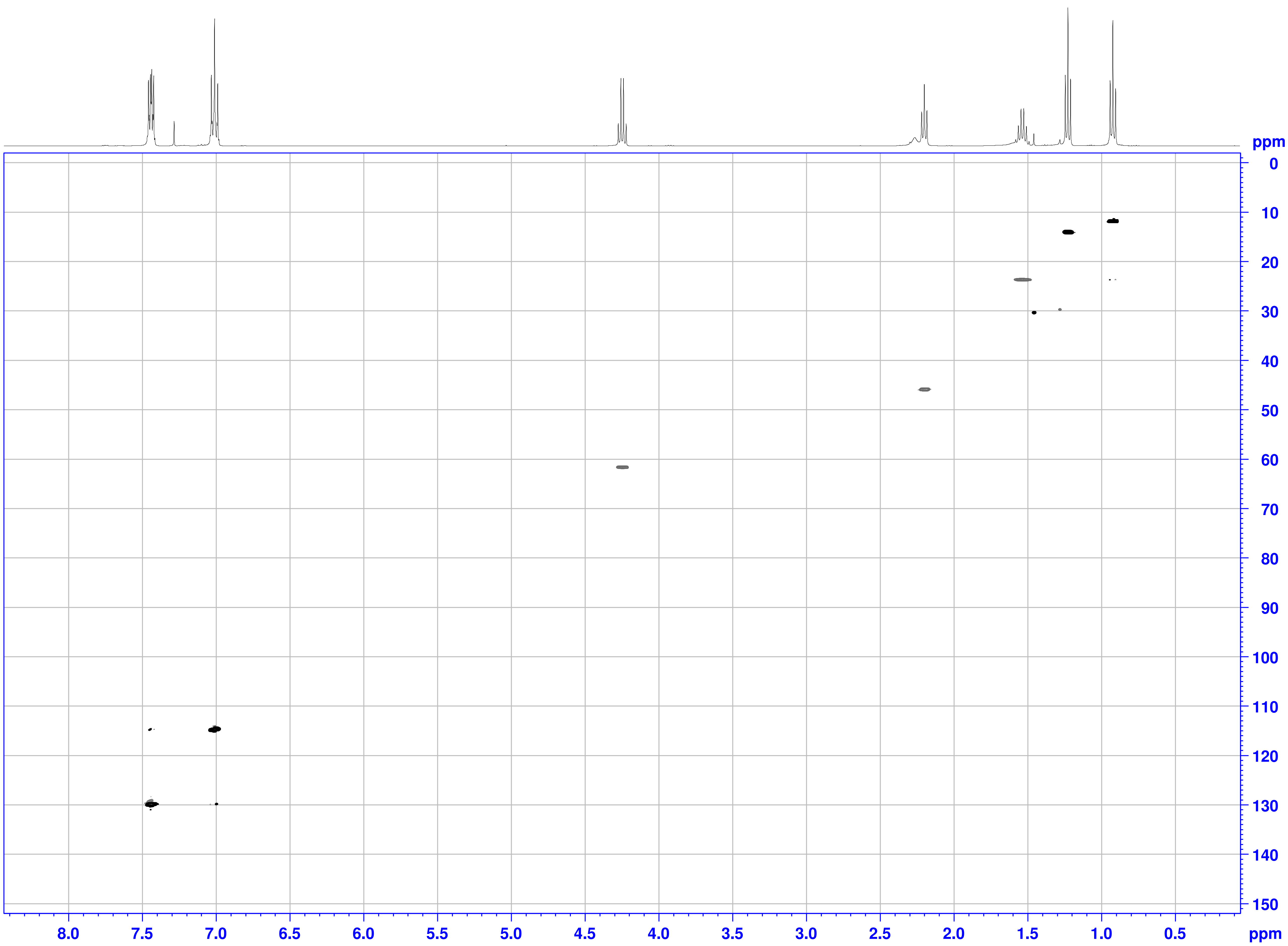


```
Current Data Parameters
NAME      pcxac8.AC238_productdry
EXPNO                       3
PROCNO                      1

F2 - Acquisition Parameters
Date_              20211214
Time                  21.09 h
INSTRUM            av3400hd
PROBHD    Z122623_0053 (
PULPROG   hsqcedetgpsp.3
TD                     1190
SOLVENT               CDCl3
NS                        4
DS                       32
SWH                3521.127 Hz
FIDRES             5.917860 Hz
AQ                0.1689800 sec
RG                   198.43
DW                  142.000 usec
DE                    10.00 usec
TE                    298.0 K
CNST2           145.0000000
D0               0.00000300 sec
D1               1.00000000 sec
D4               0.00172414 sec
D11              0.03000000 sec
D16              0.00020000 sec
D21              0.00360000 sec
IN0              0.00002980 sec
TDav                      1
SFO1            400.2017005 MHz
NUC1                     1H
P1                    10.22 usec
P2                    20.44 usec
P28       0 usec
PLW1            10.00000000 W
SFO2            100.6379173 MHz
NUC2                    13C
CPDPRG[2              garp4
P3                    10.70 usec
P14                  500.00 usec
P31                 2119.00 usec
PCPD2                 80.00 usec
PLW0      0 W
PLW2            38.00000000 W
PLW12            0.67978001 W
SPNAM[3] Crp60,0.5,20.1
SPOAL3                0.500
SPOFFS3   0 Hz
SPW3             6.64729977 W
SPNAM[18  Crp60_xfilt.2
SPOAL18               0.500
SPOFFS18 0 Hz
SPW18            1.28059995 W
GPNAM[1]        SMSQ10.100
GPZ1                  80.00 %
GPNAM[2]        SMSQ10.100
GPZ2                  20.10 %
P16                 1000.00 usec

F1 - Acquisition parameters
TD                      512
SFO1               100.6379 MHz
FIDRES            65.541107 Hz
SW                  166.722 ppm
FnMODE       Echo-Antiecho

F2 - Processing parameters
SI                     2048
SF              400.2000000 MHz
WDW                   QSINE
SSB                       2
LB        0 Hz
GB        0
PC                     1.40

F1 - Processing parameters
SI                     1024
MC2          echo-antiecho
SF              100.6303700 MHz
WDW                   QSINE
SSB                       2
LB        0 Hz
GB        0
```
