# Supplementary material for: Modular Synthesis of α,α-Diaryl α-Amino Esters via Bi(V)-Mediated Arylation/SN2-Displacement of Kukhtin–Ramirez Intermediates
Source: Org Lett. 2022 Oct 24;24(43):8002–7. doi: 10.1021/acs.orglett.2c03201 (PMC9641671; doi:10.1021/acs.orglett.2c03201)
Supplement: Supplementary file 4 — ol2c03201_si_004.zip [file ol2c03201_si_004.zip › FID_13-17/17/17_19F/2/pdata/1/pcxac8.AC237_column_2_1.pdf]

Processed with back linear prediction to eliminate baseline artefacts: broad peaks may be attenuated. Standard data in file 100002

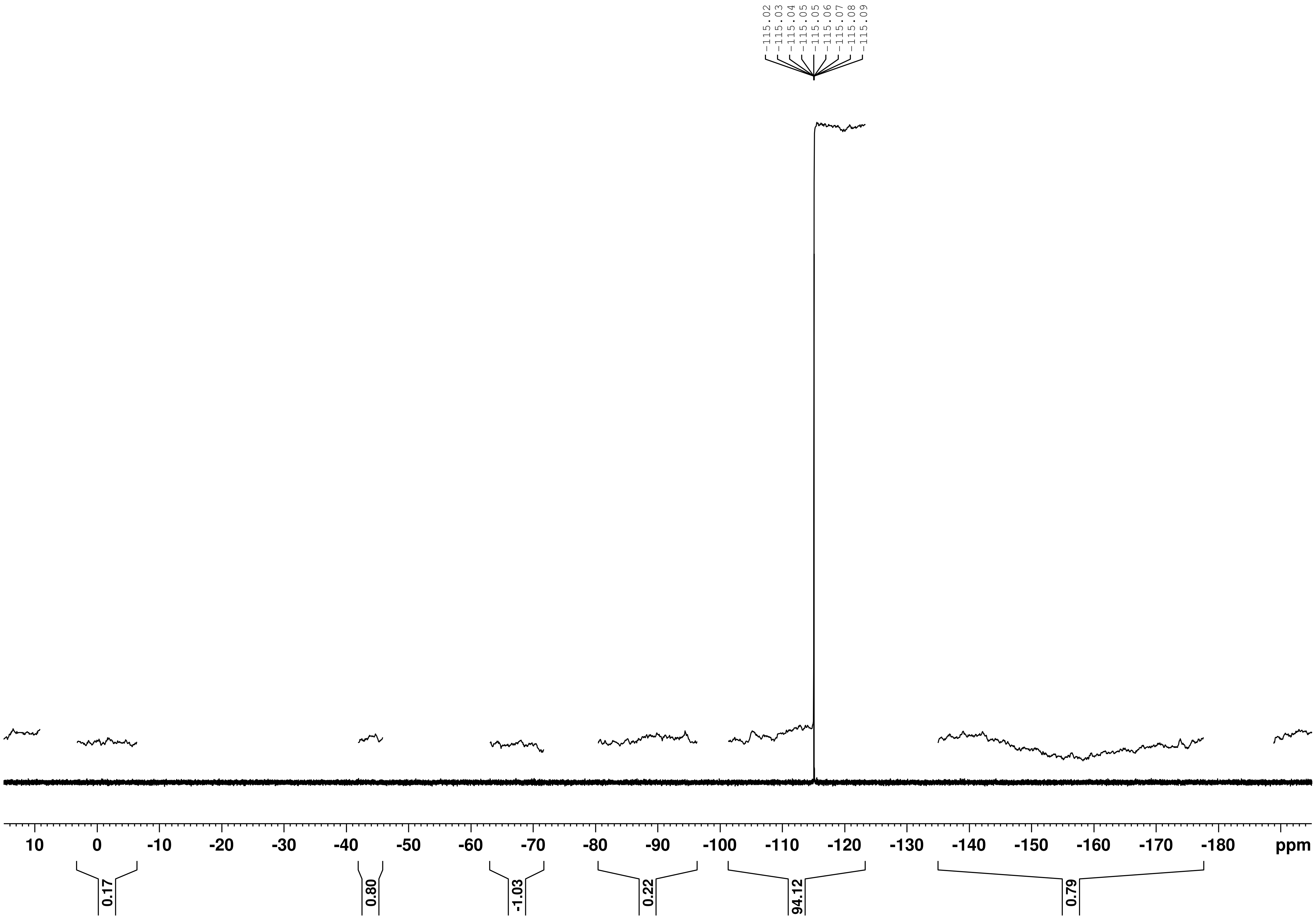

Current Data Parameters
NAME pcxac8.AC237_column
EXPNO 2
PROCNO 1

F2 - Acquisition Parameters
Date_ 20211210
Time 16.04 h
INSTRUM av3400
PROBHD Z104450_0225 (
PULPROG zgflqn
TD 261992
SOLVENT CDCl3
NS 16
DS 4
SWH 85227.273 Hz
FIDRES 0.650610 Hz
AQ 1.5370197 sec
RG 724
DW 5.867 usec
DE 6.77 usec
TE 298.0 K
D1 1.50000000 sec
TD0 1
SFO1 376.4080300 MHz
NUC1 19F
P1 13.30 usec
PLW1 16.11100006 W

F2 - Processing parameters
SI 262144
SF 376.4419098 MHz
WDW EM
SSB 0
LB 1.00 Hz
GB 0
PC 1.00
