# Supplementary material for: Modular Synthesis of α,α-Diaryl α-Amino Esters via Bi(V)-Mediated Arylation/SN2-Displacement of Kukhtin–Ramirez Intermediates
Source: Org Lett. 2022 Oct 24;24(43):8002–7. doi: 10.1021/acs.orglett.2c03201 (PMC9641671; doi:10.1021/acs.orglett.2c03201)
Supplement: Supplementary file 4 — ol2c03201_si_004.zip [file ol2c03201_si_004.zip › FID_13-17/17/17_HMBC/pdata/1/pcxac8.AC242_productdry_5_1.pdf]

HMBC spectrum: very low level one-bond artefacts may be observed.

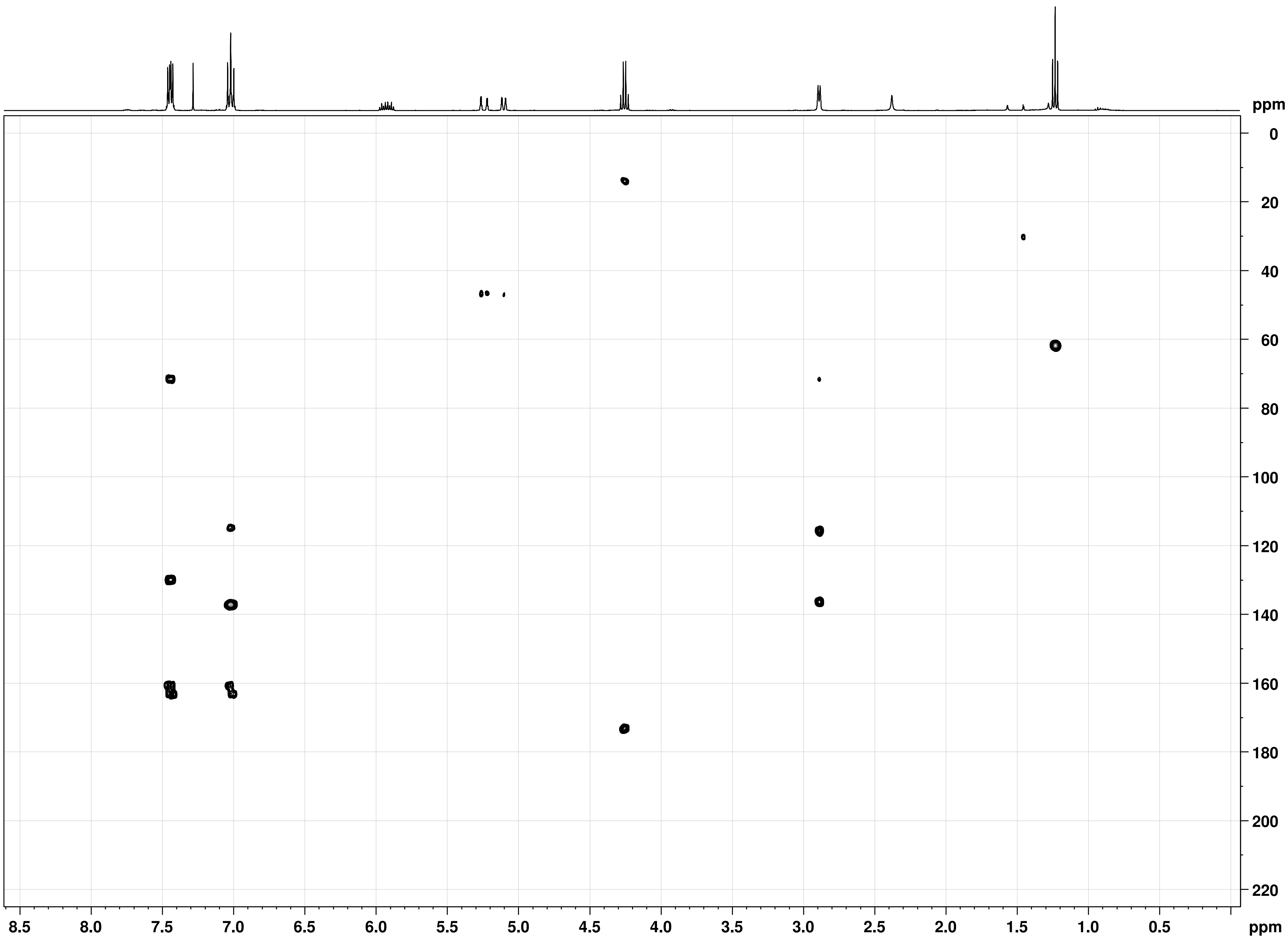


```
Current Data Parameters
NAME     pcxac8.AC242_productdry
EXPNO                   5
PROCNO                  1

F2 - Acquisition Parameters
Date_           20220115
Time                6.10 h
INSTRUM            av3400
PROBHD   Z104450_0225 (
PULPROG    hmbcetgpl3nd
TD                  1190
SOLVENT            CDCl3
NS                     8
DS                    16
SWH             3472.222 Hz
FIDRES          5.835668 Hz
AQ             0.1713600 sec
RG                  2050
DW               144.000 usec
DE                  6.50 usec
TE                 298.0 K
CNST6        120.0000000
CNST7        210.0000000
CNST13         8.5000000
D0            0.00000300 sec
D1            1.00000000 sec
D6            0.05882353 sec
D16           0.00020000 sec
IN0           0.00002160 sec
TDav                   1
SFO1         400.0717095 MHz
NUC1                  1H
P1                 14.00 usec
P2                 28.00 usec
PLW1         11.92800045 W
SFO2         100.6087473 MHz
NUC2                 13C
P3                  9.75 usec
P24              2000.00 usec
PLW2         63.68099976 W
SPNAM[7]     Crp60comp.4
SPOAL7             0.500
SPOFFS7  0 Hz
SPW7          9.24930000 W
GPNAM[1]      SMSQ10.100
GPZ1               80.00 %
GPNAM[3]      SMSQ10.100
GPZ3               14.00 %
GPNAM[4]      SMSQ10.100
GPZ4               -8.00 %
GPNAM[5]      SMSQ10.100
GPZ5               -4.00 %
GPNAM[6]      SMSQ10.100
GPZ6               -2.00 %
P16              1000.00 usec

F1 - Acquisition parameters
TD                   128
SFO1            100.6087 MHz
FIDRES        361.689819 Hz
SW               230.081 ppm
FnMODE    Echo-Antiecho

F2 - Processing parameters
SI                  2048
SF           400.0700000 MHz
WDW                 SINE
SSB      0
LB       0 Hz
GB       0
PC                  1.40

F1 - Processing parameters
SI                   512
MC2       echo-antiecho
SF           100.5976815 MHz
WDW                QSINE
SSB                    2
LB       0 Hz
GB       0
```
