# Supplementary material for: Modular Synthesis of α,α-Diaryl α-Amino Esters via Bi(V)-Mediated Arylation/SN2-Displacement of Kukhtin–Ramirez Intermediates
Source: Org Lett. 2022 Oct 24;24(43):8002–7. doi: 10.1021/acs.orglett.2c03201 (PMC9641671; doi:10.1021/acs.orglett.2c03201)
Supplement: Supplementary file 5 — ol2c03201_si_005.zip [file ol2c03201_si_005.zip › FID_18-22/18/18_HSQC/pdata/1/pcxac8.AC229B_product_5_1.pdf]

HMBC spectrum: very low level one-bond artefacts may be observed.

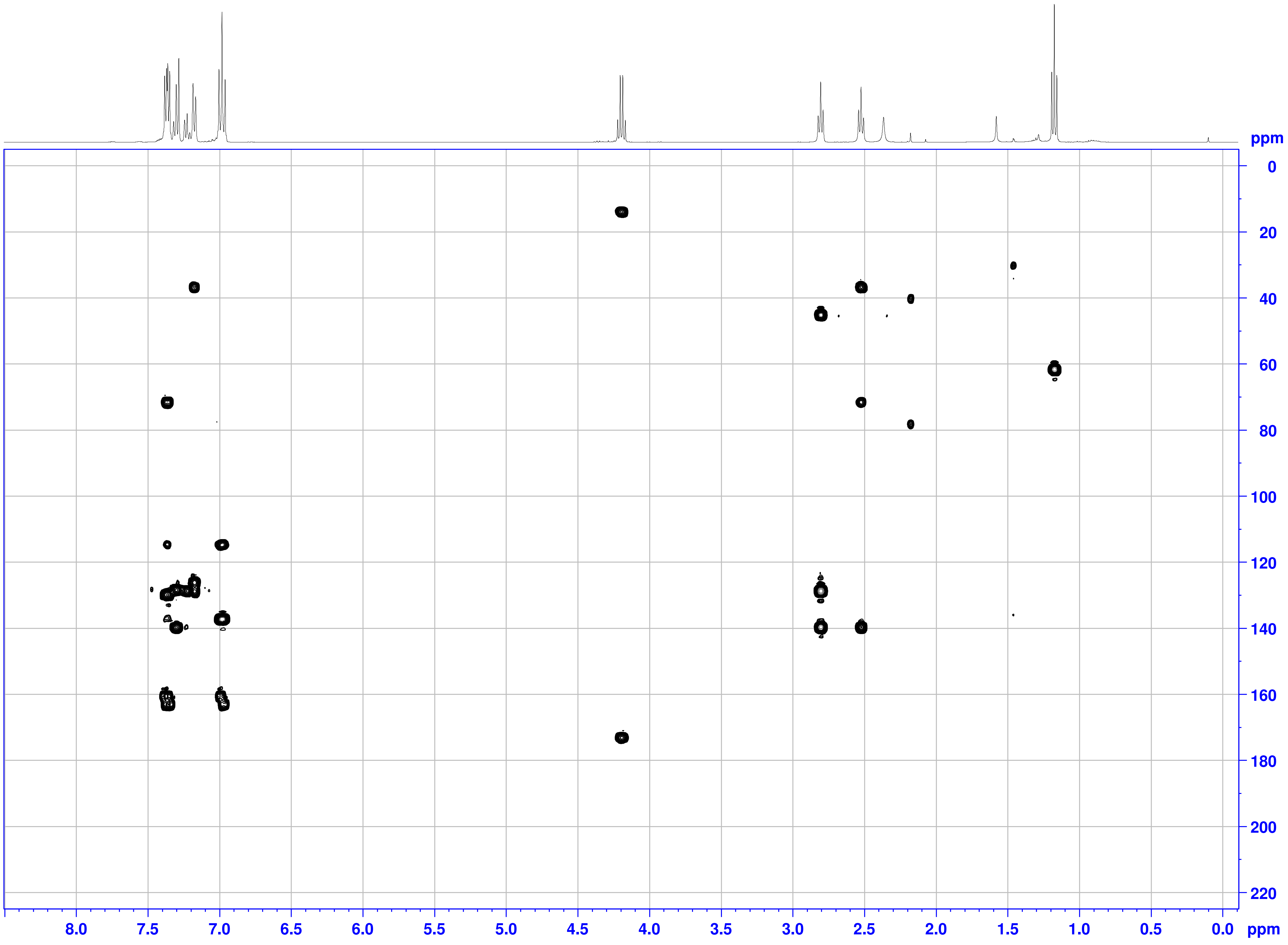


```
Current Data Parameters
NAME     pcxac8.AC229B_product
EXPNO                   5
PROCNO                  1

F2 - Acquisition Parameters
Date_          20211125
Time              22.24 h
INSTRUM        av3400hd
PROBHD   Z122623_0053 (
PULPROG     hmbcetgpl3nd
TD                 1190
SOLVENT            CDCl3
NS                    8
DS                   16
SWH            3448.276 Hz
FIDRES         5.795422 Hz
AQ            0.1725500 sec
RG               198.43
DW              145.000 usec
DE                10.00 usec
TE                298.0 K
CNST6       120.0000000
CNST7       175.0000000
CNST13        8.5000000
D0           0.00000300 sec
D1           1.00000000 sec
D6           0.05882353 sec
D16          0.00020000 sec
IN0          0.00002160 sec
TDav                  1
SFO1        400.2016798 MHz
NUC1                 1H
P1                10.22 usec
P2                20.44 usec
PLW1        10.00000000 W
SFO2        100.6414394 MHz
NUC2                13C
P3                10.70 usec
P24             2000.00 usec
PLW2        38.00000000 W
SPNAM[7]    Crp60comp.4
SPOAL7            0.500
SPOFFS7  0 Hz
SPW7         6.64729977 W
GPNAM[1]     SMSQ10.100
GPZ1              80.00 %
GPNAM[3]     SMSQ10.100
GPZ3              14.00 %
GPNAM[4]     SMSQ10.100
GPZ4              -8.00 %
GPNAM[5]     SMSQ10.100
GPZ5              -4.00 %
GPNAM[6]     SMSQ10.100
GPZ6              -2.00 %
P16             1000.00 usec

F1 - Acquisition parameters
TD                  128
SFO1           100.6414 MHz
FIDRES       361.689819 Hz
SW              230.006 ppm
FnMODE    Echo-Antiecho

F2 - Processing parameters
SI                 2048
SF          400.2000000 MHz
WDW                SINE
SSB      0
LB       0 Hz
GB       0
PC                 1.40

F1 - Processing parameters
SI                  512
MC2      echo-antiecho
SF          100.6303700 MHz
WDW               QSINE
SSB                   2
LB       0 Hz
GB       0
```
