# Supplementary material for: Modular Synthesis of α,α-Diaryl α-Amino Esters via Bi(V)-Mediated Arylation/SN2-Displacement of Kukhtin–Ramirez Intermediates
Source: Org Lett. 2022 Oct 24;24(43):8002–7. doi: 10.1021/acs.orglett.2c03201 (PMC9641671; doi:10.1021/acs.orglett.2c03201)
Supplement: Supplementary file 5 — ol2c03201_si_005.zip [file ol2c03201_si_005.zip › FID_18-22/19/19_HMBC/pdata/1/pcxac8.AC241_productdry3_5_1.pdf]

HMBC spectrum: very low level one-bond artefacts may be observed.

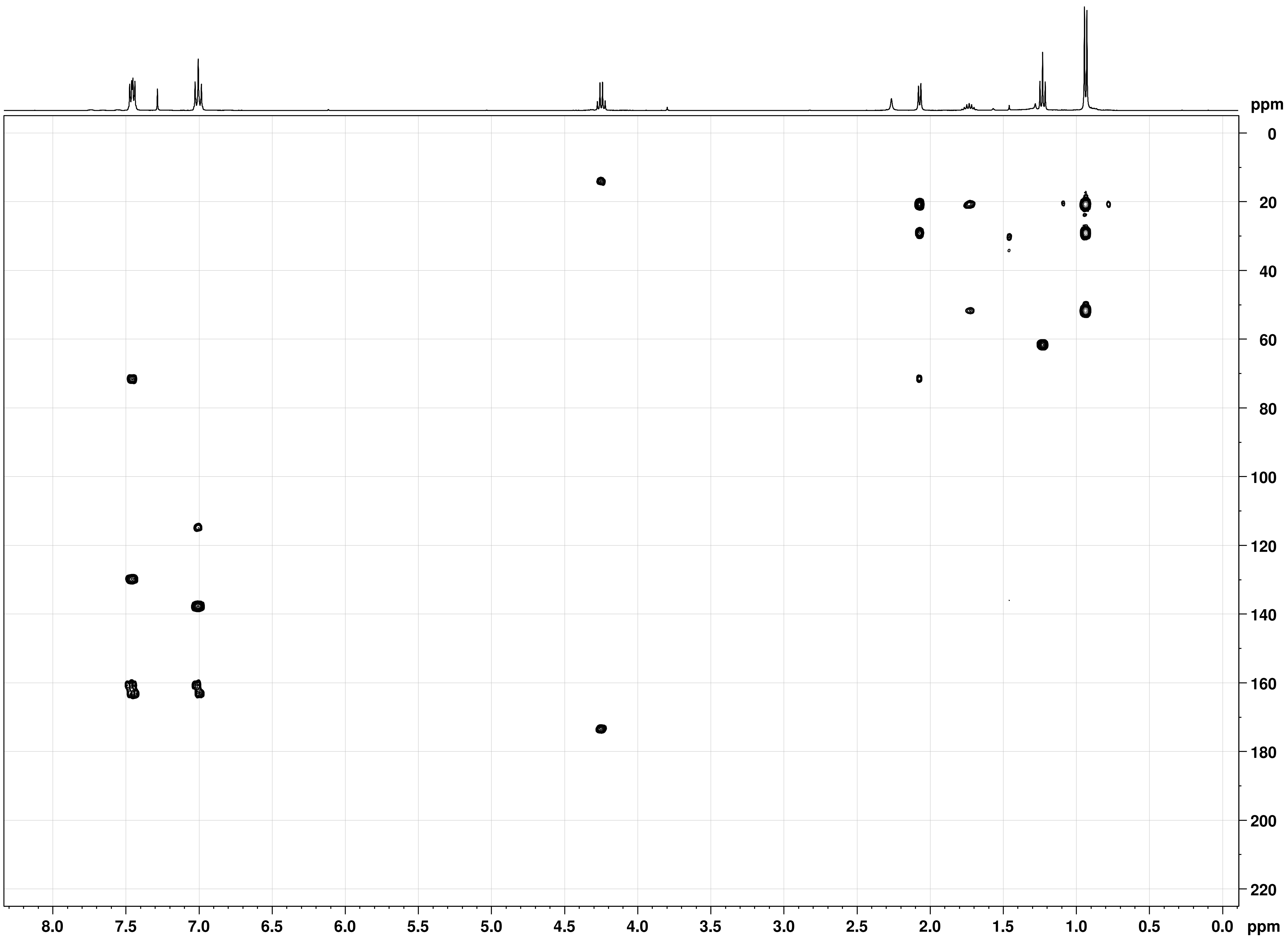


```
Current Data Parameters
NAME     pcxac8.AC241_productdry3
EXPNO                   5
PROCNO                  1

F2 - Acquisition Parameters
Date_            20220112
Time                23.58 h
INSTRUM            av3400
PROBHD   Z104450_0225 (
PULPROG     hmbcetgpl3nd
TD                   1190
SOLVENT             CDCl3
NS                      8
DS                     16
SWH              3378.378 Hz
FIDRES           5.677947 Hz
AQ              0.1761200 sec
RG                   2050
DW                148.000 usec
DE                   6.50 usec
TE                  298.0 K
CNST6         120.0000000
CNST7         210.0000000
CNST13          8.5000000
D0             0.00000300 sec
D1             1.00000000 sec
D6             0.05882353 sec
D16            0.00020000 sec
IN0            0.00002160 sec
TDav                    1
SFO1          400.0716451 MHz
NUC1                   1H
P1                  14.00 usec
P2                  28.00 usec
PLW1          11.92800045 W
SFO2          100.6087473 MHz
NUC2                  13C
P3                   9.75 usec
P24               2000.00 usec
PLW2          63.68099976 W
SPNAM[7]     Crp60comp.4
SPOAL7              0.500
SPOFFS7  0 Hz
SPW7           9.24930000 W
GPNAM[1]      SMSQ10.100
GPZ1                80.00 %
GPNAM[3]      SMSQ10.100
GPZ3                14.00 %
GPNAM[4]      SMSQ10.100
GPZ4                -8.00 %
GPNAM[5]      SMSQ10.100
GPZ5                -4.00 %
GPNAM[6]      SMSQ10.100
GPZ6                -2.00 %
P16               1000.00 usec

F1 - Acquisition parameters
TD                    128
SFO1             100.6087 MHz
FIDRES         361.689819 Hz
SW                230.081 ppm
FnMODE     Echo-Antiecho

F2 - Processing parameters
SI                   2048
SF            400.0700000 MHz
WDW                  SINE
SSB      0
LB       0 Hz
GB       0
PC                   1.40

F1 - Processing parameters
SI                    512
MC2        echo-antiecho
SF            100.5976815 MHz
WDW                 QSINE
SSB                     2
LB       0 Hz
GB       0
```
