# Supplementary material for: Modular Synthesis of α,α-Diaryl α-Amino Esters via Bi(V)-Mediated Arylation/SN2-Displacement of Kukhtin–Ramirez Intermediates
Source: Org Lett. 2022 Oct 24;24(43):8002–7. doi: 10.1021/acs.orglett.2c03201 (PMC9641671; doi:10.1021/acs.orglett.2c03201)
Supplement: Supplementary file 5 — ol2c03201_si_005.zip [file ol2c03201_si_005.zip › FID_18-22/19/19_HSQB/pdata/1/pcxac8.AC241_productdry3_4_1.pdf]

Multiplicity-edited HSQC Spectrum: CH and Me up (black), CH2 down (grey).

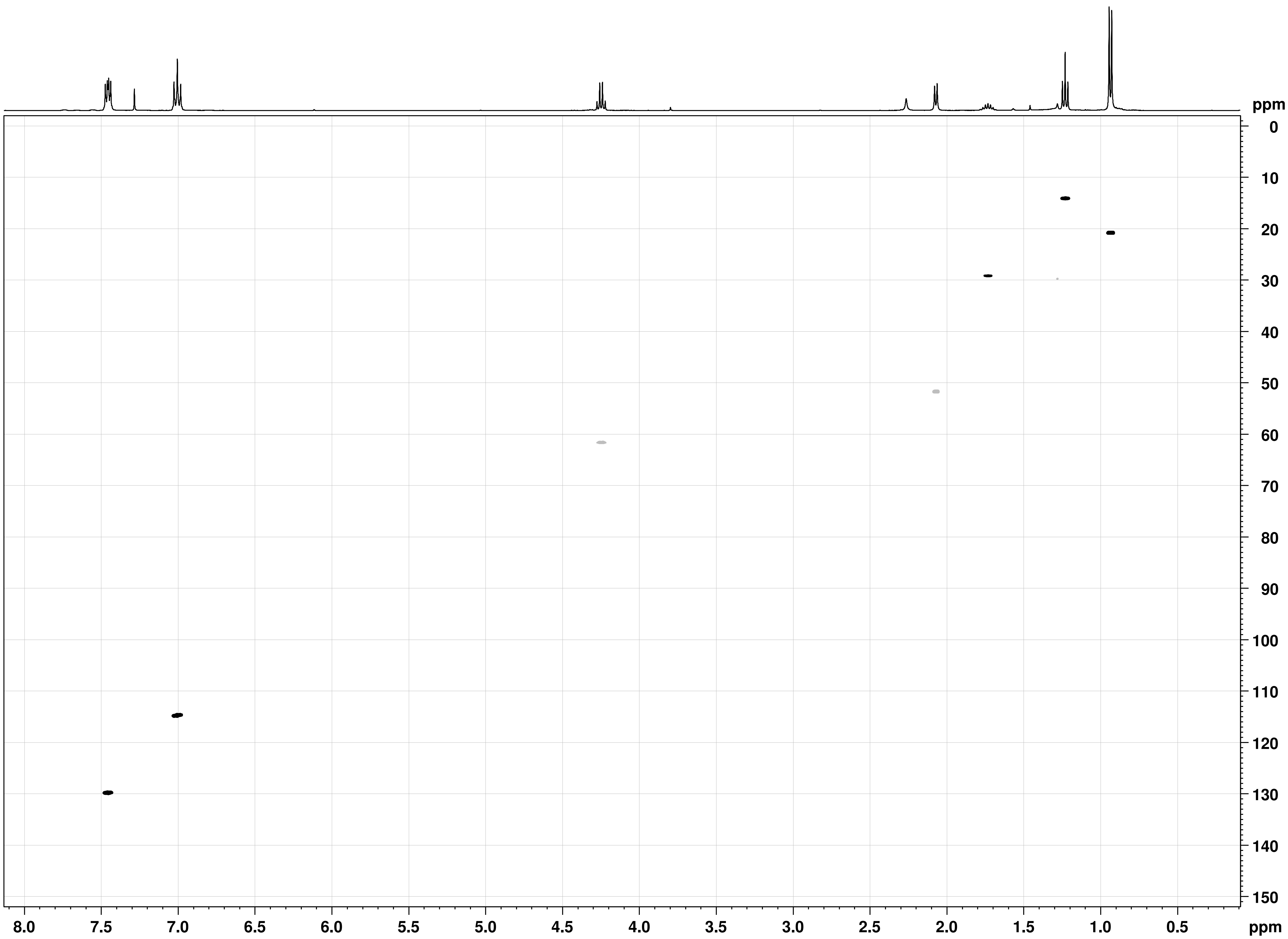

Current Data Parameters
NAME pcxac8.AC241_productdry3
EXPNO 4
PROCNO 1

F2 - Acquisition Parameters
Date_ 20220112
Time 23.15 h
INSTRUM av3400
PROBHD Z104450_0225 (
PULPROG hsqcedetgpsp.3
TD 1190
SOLVENT CDCl3
NS 4
DS 32
SWH 3378.378 Hz
FIDRES 5.677947 Hz
AQ 0.1761200 sec
RG 2050
DW 148.000 usec
DE 6.50 usec
TE 298.0 K
CNST2 145.0000000
D0 0.00000300 sec
D1 1.00000000 sec
D4 0.00172414 sec
D11 0.03000000 sec
D16 0.00020000 sec
D21 0.00360000 sec
IN0 0.00002980 sec
TDav 1
SFO1 400.0716451 MHz
NUC1 1H
P1 14.00 usec
P2 28.00 usec
P28 1000.00 usec
PLW1 11.92800045 W
SFO2 100.6052263 MHz
NUC2 13C
CPDPRG[2 garp4
P3 9.75 usec
P14 500.00 usec
P31 2119.00 usec
PCPD2 80.00 usec
PLW0 0 W
PLW2 63.68099976 W
PLW12 0.94588000 W
SPNAM[3] Crp60,0.5,20.1
SPOAL3 0.500
SPOFFS3 0 Hz
SPW3 9.24930000 W
SPNAM[18 Crp60_xfilt.2
SPOAL18 0.500
SPOFFS18 0 Hz
SPW18 1.78180003 W
GPNAM[1] SMSQ10.100
GPZ1 80.00 %
GPNAM[2] SMSQ10.100
GPZ2 20.10 %
P16 1000.00 usec

F1 - Acquisition parameters
TD 512
SFO1 100.6052 MHz
FIDRES 65.541107 Hz
SW 166.776 ppm
FnMODE Echo-Antiecho

F2 - Processing parameters
SI 2048
SF 400.0700000 MHz
WDW QSINE
SSB 2
LB 0 Hz
GB 0
PC 1.40

F1 - Processing parameters
SI 1024
MC2 echo-antiecho
SF 100.5976815 MHz
WDW QSINE
SSB 2
LB 0 Hz
GB 0
