# Supplementary material for: Modular Synthesis of α,α-Diaryl α-Amino Esters via Bi(V)-Mediated Arylation/SN2-Displacement of Kukhtin–Ramirez Intermediates
Source: Org Lett. 2022 Oct 24;24(43):8002–7. doi: 10.1021/acs.orglett.2c03201 (PMC9641671; doi:10.1021/acs.orglett.2c03201)
Supplement: Supplementary file 5 — ol2c03201_si_005.zip [file ol2c03201_si_005.zip › FID_18-22/20/20_13C/pdata/1/pcxac8.AC340_productdry_2_1.pdf]

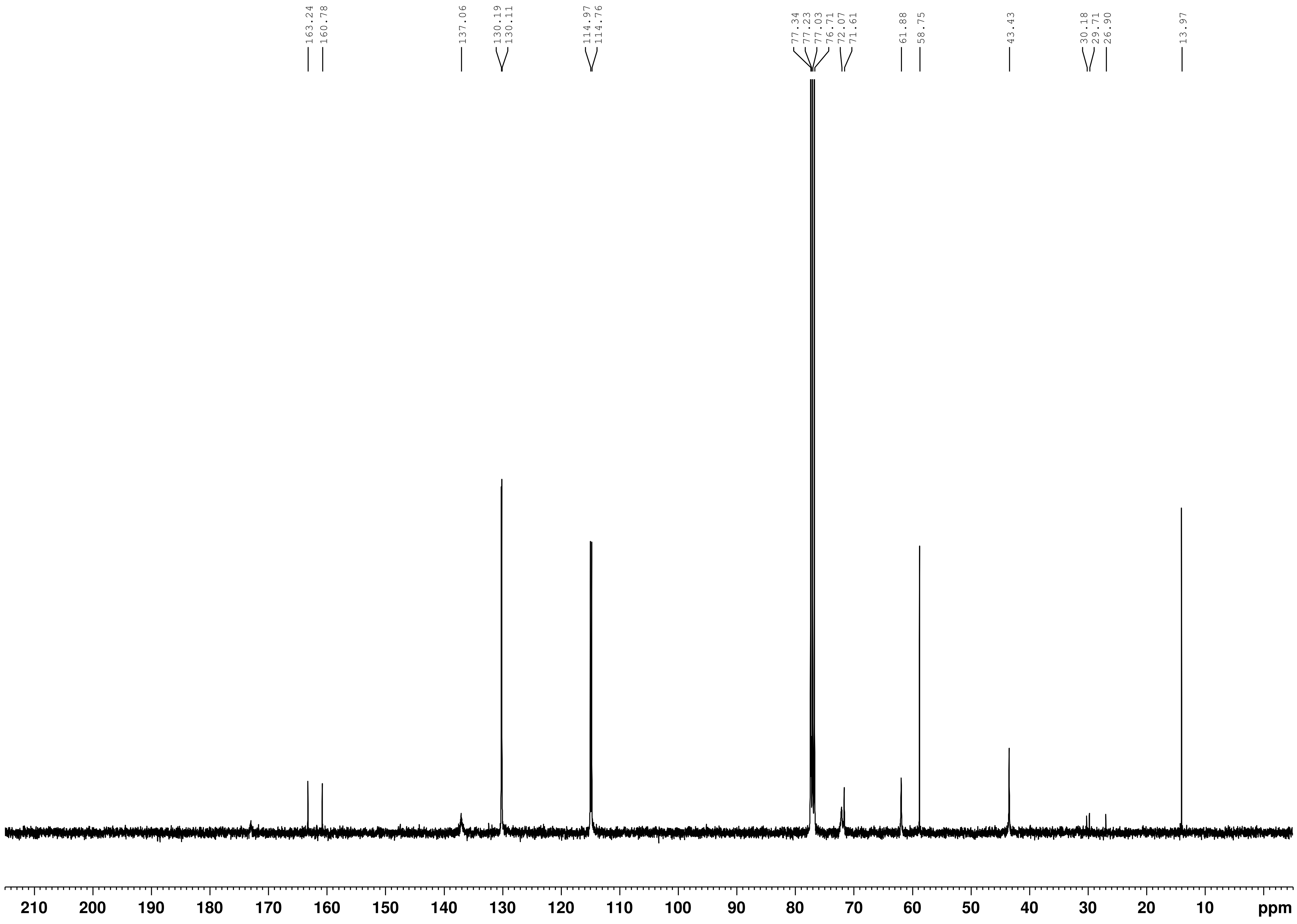

Current Data Parameters
NAME pcxac8.AC340_produc
EXPNO 2
PROCNO 1

F2 - Acquisition Parameters
Date_ 20220503
Time 23.28 h
INSTRUM av3400
PROBHD Z104450_0225 (
PULPROG zgpg30
TD 32768
SOLVENT CDCl3
NS 4096
DS 4
SWH 24038.461 Hz
FIDRES 1.467191 Hz
AQ 0.6815744 sec
RG 2050
DW 20.800 usec
DE 9.63 usec
TE 298.0 K
D1 1.00000000 sec
D11 0.03000000 sec
TD0 1
SFO1 100.6087473 MHz
NUC1 13C
P1 9.75 usec
PLW1 63.68099976 W
SFO2 400.0716003 MHz
NUC2 1H
CPDPRG[2 waltz16
PCPD2 90.00 usec
PLW2 11.92800045 W
PLW12 0.28863001 W
PLW13 0.14518000 W

F2 - Processing parameters
SI 32768
SF 100.5976815 MHz
WDW EM
SSB 0
LB 1.00 Hz
GB 0
PC 1.40
