# Supplementary material for: Modular Synthesis of α,α-Diaryl α-Amino Esters via Bi(V)-Mediated Arylation/SN2-Displacement of Kukhtin–Ramirez Intermediates
Source: Org Lett. 2022 Oct 24;24(43):8002–7. doi: 10.1021/acs.orglett.2c03201 (PMC9641671; doi:10.1021/acs.orglett.2c03201)
Supplement: Supplementary file 5 — ol2c03201_si_005.zip [file ol2c03201_si_005.zip › FID_18-22/20/20_19F/1/pdata/1/pcxac8.AC340_19F_1_1.pdf]

Processed with back linear prediction to eliminate baseline artefacts: broad peaks may be attenuated. Standard data in file 100001

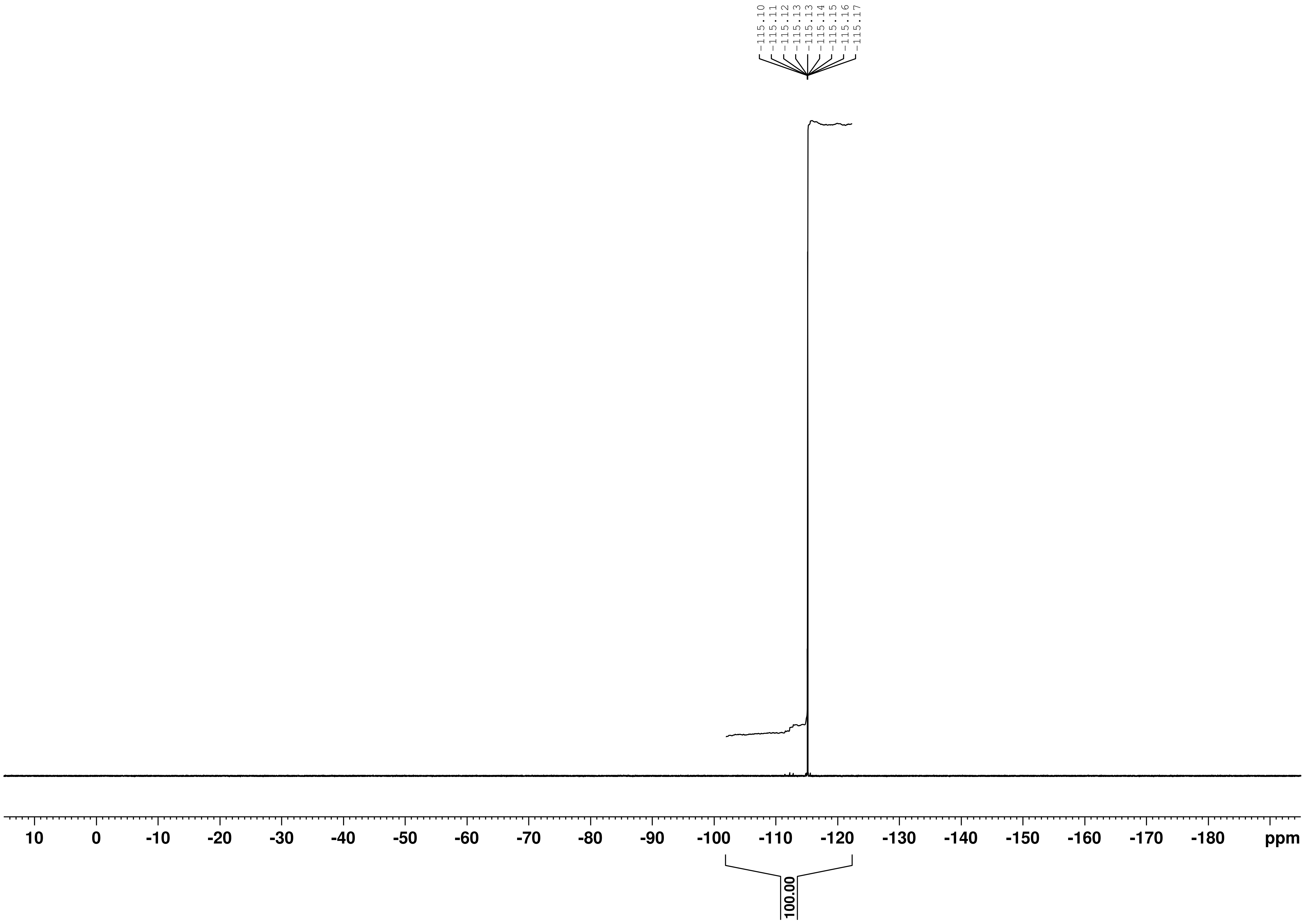


```
Current Data Parameters
NAME      pcxac8.AC340_19F
EXPNO                    1
PROCNO                   1

F2 - Acquisition Parameters
Date_             20220617
Time                 14.55 h
INSTRUM             av3400
PROBHD    Z104450_0225 (
PULPROG             zgflqn
TD                  261992
SOLVENT              CDCl3
NS                      32
DS                       4
SWH              85227.273 Hz
FIDRES            0.650610 Hz
AQ               1.5370197 sec
RG                    1620
DW                   5.867 usec
DE                    6.77 usec
TE                   298.0 K
D1              1.50000000 sec
TD0                      1
SFO1           376.4080300 MHz
NUC1                   19F
P1                   13.30 usec
PLW1           16.11100006 W

F2 - Processing parameters
SI                  262144
SF             376.4419098 MHz
WDW                     EM
SSB       0
LB                    1.00 Hz
GB        0
PC                    1.00
```
