# Supplementary material for: Modular Synthesis of α,α-Diaryl α-Amino Esters via Bi(V)-Mediated Arylation/SN2-Displacement of Kukhtin–Ramirez Intermediates
Source: Org Lett. 2022 Oct 24;24(43):8002–7. doi: 10.1021/acs.orglett.2c03201 (PMC9641671; doi:10.1021/acs.orglett.2c03201)
Supplement: Supplementary file 5 — ol2c03201_si_005.zip [file ol2c03201_si_005.zip › FID_18-22/21/21_13C/pdata/1/pcxac8.AC234A_dry_bis_2_1.pdf]

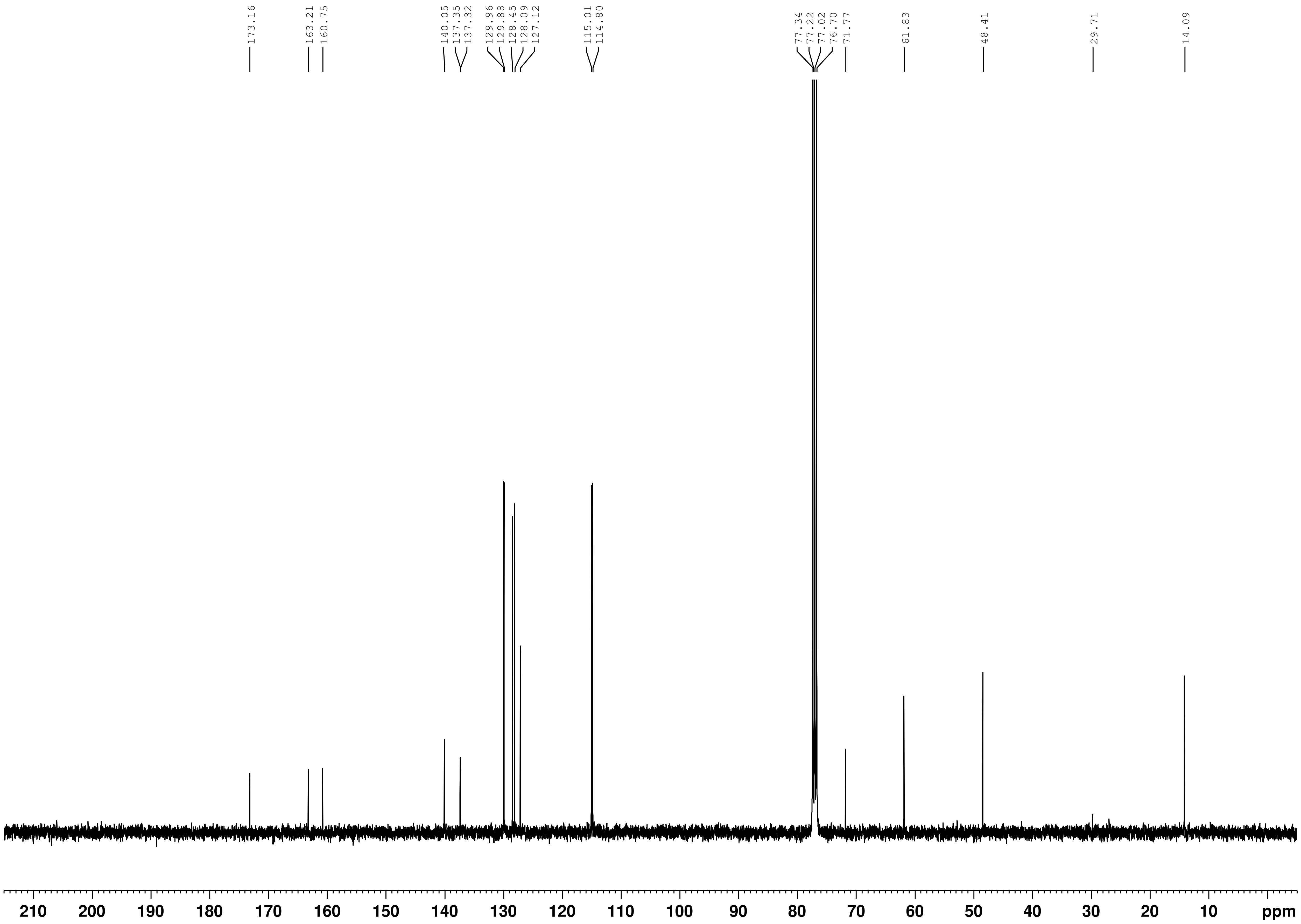

Current Data Parameters

NAME	pcxac8.AC234A_dry_[illegible]
EXPNO	2
PROCNO	1

F2 - Acquisition Parameters

Date_	20211209
Time	21.04 h
INSTRUM	av3400
PROBHD	Z104450_0225 (
PULPROG	zgpg30
TD	32768
SOLVENT	CDCl3
NS	4096
DS	4
SWH	24038.461 Hz
FIDRES	1.467191 Hz
AQ	0.6815744 sec
RG	2050
DW	20.800 usec
DE	9.63 usec
TE	298.0 K
D1	1.00000000 sec
D11	0.03000000 sec
TD0	1
SFO1	100.6087473 MHz
NUC1	13C
P1	9.75 usec
PLW1	63.68099976 W
SFO2	400.0716003 MHz
NUC2	1H
CPDPRG[2	waltz16
PCPD2	90.00 usec
PLW2	11.92800045 W
PLW12	0.28863001 W
PLW13	0.14518000 W

F2 - Processing parameters

SI	32768
SF	100.5976815 MHz
WDW	EM
SSB	0
LB	1.00 Hz
GB	0
PC	1.40
