# Supplementary material for: Modular Synthesis of α,α-Diaryl α-Amino Esters via Bi(V)-Mediated Arylation/SN2-Displacement of Kukhtin–Ramirez Intermediates
Source: Org Lett. 2022 Oct 24;24(43):8002–7. doi: 10.1021/acs.orglett.2c03201 (PMC9641671; doi:10.1021/acs.orglett.2c03201)
Supplement: Supplementary file 5 — ol2c03201_si_005.zip [file ol2c03201_si_005.zip › FID_18-22/21/21_COSY/pdata/1/pcxac8.AC234A_dry_bis_3_1.pdf]

Double quantum filtered COSY Spectrum.

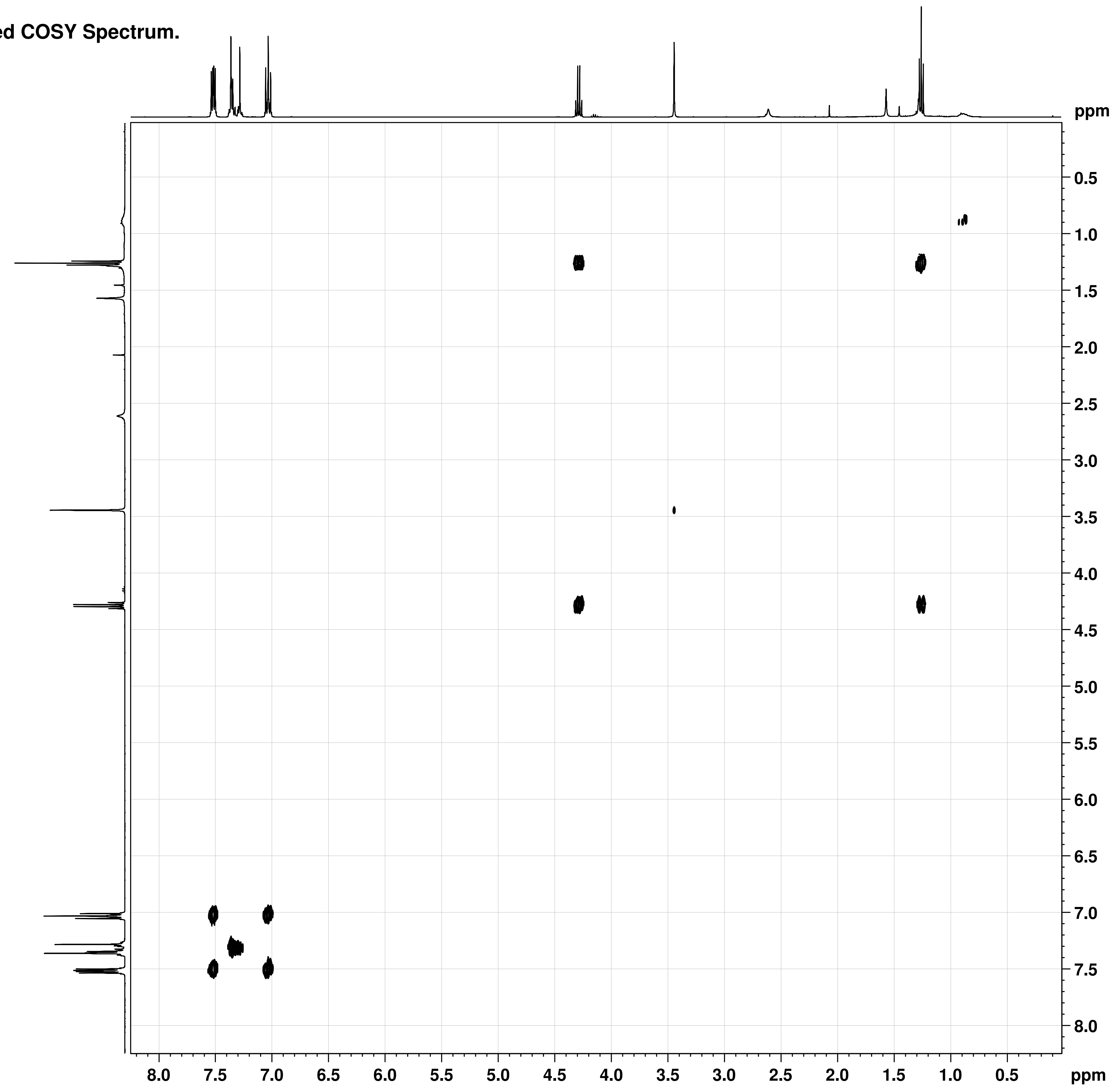

Current Data Parameters

NAME	pcxac8.AC234A_dry_bis
EXPNO	3
PROCNO	1

F2 - Acquisition Parameters

Date_	20211209
Time	21.05 h
INSTRUM	av3400
PROBHD	Z104450_0225 (
PULPROG	cosygpmfppqf
TD	2048
SOLVENT	CDCl3
NS	2
DS	16
SWH	3625.816 Hz
FIDRES	3.540836 Hz
AQ	0.2824192 sec
RG	2050
DW	137.900 usec
DE	8.84 usec
TE	298.0 K
D0	0.00000300 sec
D1	0.91452998 sec
D11	0.03000000 sec
D12	0.00002000 sec
D13	0.00000400 sec
D16	0.00020000 sec
IN0	0.00027580 sec
TDav	1
SFO1	400.0716543 MHz
NUC1	1H
P1	14.00 usec
P17	2500.00 usec
PLW1	11.92800045 W
PLW10	2.59770012 W
GPNAM[1]	SMSQ10.100
GPZ1	16.00 %
GPNAM[2]	SMSQ10.100
GPZ2	12.00 %
GPNAM[3]	SMSQ10.100
GPZ3	40.00 %
P16	1000.00 usec

F1 - Acquisition parameters

TD	128
SFO1	400.0717 MHz
FIDRES	56.653374 Hz
SW	9.063 ppm
FnMODE	QF

F2 - Processing parameters

SI	2048
SF	400.0700000 MHz
WDW	SINE
SSB	0
LB	0 Hz
GB	0
PC	1.40

F1 - Processing parameters

SI	512
MC2	QF
SF	400.0700000 MHz
WDW	SINE
SSB	0
LB	0 Hz
GB	0
