# Supplementary material for: Modular Synthesis of α,α-Diaryl α-Amino Esters via Bi(V)-Mediated Arylation/SN2-Displacement of Kukhtin–Ramirez Intermediates
Source: Org Lett. 2022 Oct 24;24(43):8002–7. doi: 10.1021/acs.orglett.2c03201 (PMC9641671; doi:10.1021/acs.orglett.2c03201)
Supplement: Supplementary file 5 — ol2c03201_si_005.zip [file ol2c03201_si_005.zip › FID_18-22/21/21_HMBC/pdata/1/pcxac8.AC234A_dry_bis_5_1.pdf]

HMBC spectrum: very low level one-bond artefacts may be observed.

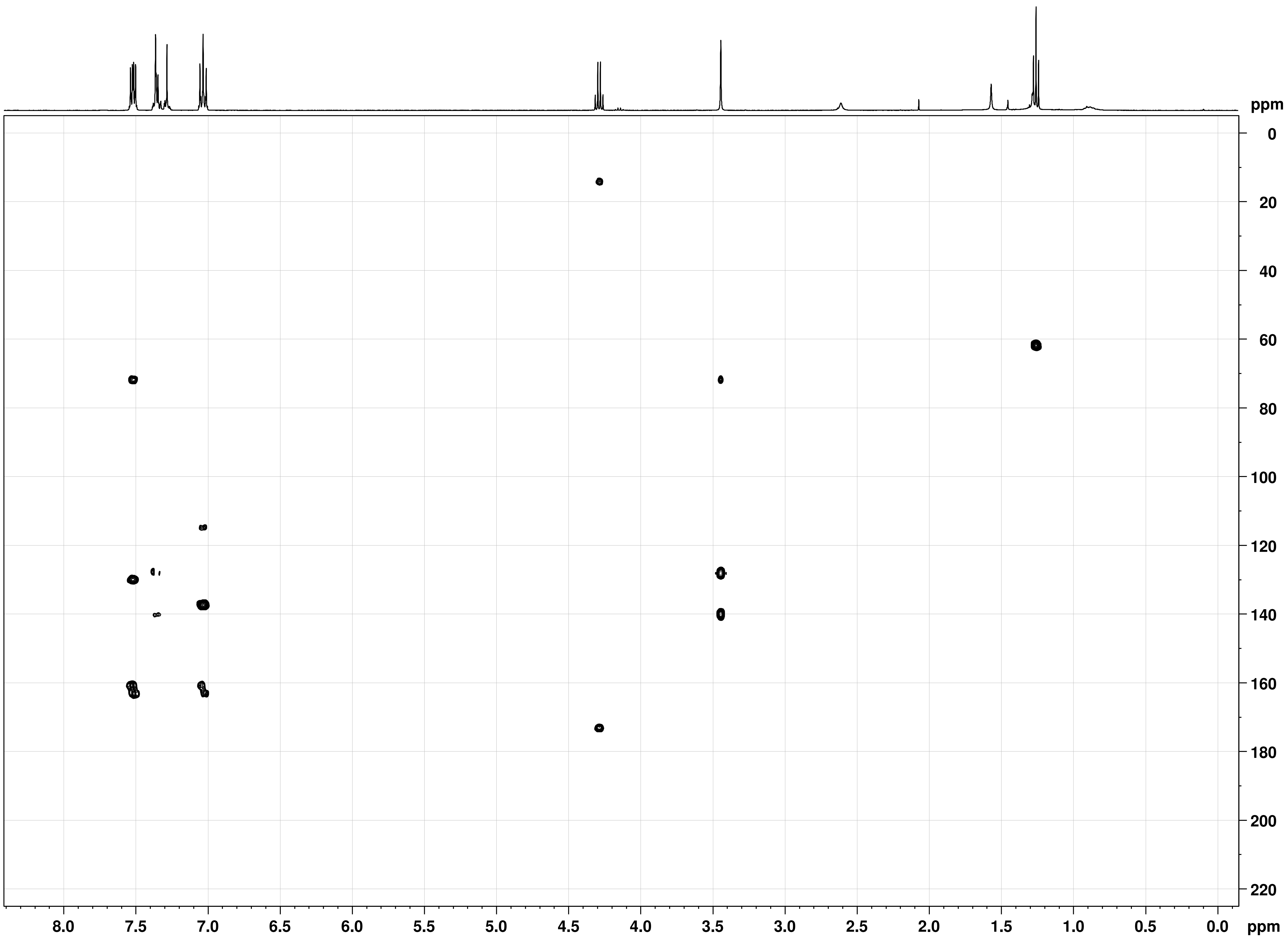


```
Current Data Parameters
NAME     pcxac8.AC234A_dry_bis
EXPNO                    5
PROCNO                   1

F2 - Acquisition Parameters
Date_             20211209
Time                 21.56 h
INSTRUM             av3400
PROBHD   Z104450_0225 (
PULPROG    hmbcetgpl3nd
TD                    1190
SOLVENT              CDCl3
NS                       8
DS                      16
SWH               3424.657 Hz
FIDRES            5.755727 Hz
AQ               0.1737400 sec
RG                    2050
DW                 146.000 usec
DE                    6.50 usec
TE                   298.0 K
CNST6          120.0000000
CNST7          210.0000000
CNST13           8.5000000
D0              0.00000300 sec
D1              1.00000000 sec
D6              0.05882353 sec
D16             0.00020000 sec
IN0             0.00002160 sec
TDav                     1
SFO1           400.0716543 MHz
NUC1                    1H
P1                   14.00 usec
P2                   28.00 usec
PLW1           11.92800045 W
SFO2           100.6087473 MHz
NUC2                   13C
P3                    9.75 usec
P24                2000.00 usec
PLW2           63.68099976 W
SPNAM[7]       Crp60comp.4
SPOAL7               0.500
SPOFFS7  0 Hz
SPW7            9.24930000 W
GPNAM[1]        SMSQ10.100
GPZ1                 80.00 %
GPNAM[3]        SMSQ10.100
GPZ3                 14.00 %
GPNAM[4]        SMSQ10.100
GPZ4                 -8.00 %
GPNAM[5]        SMSQ10.100
GPZ5                 -4.00 %
GPNAM[6]        SMSQ10.100
GPZ6                 -2.00 %
P16                1000.00 usec

F1 - Acquisition parameters
TD                     128
SFO1              100.6087 MHz
FIDRES          361.689819 Hz
SW                 230.081 ppm
FnMODE      Echo-Antiecho

F2 - Processing parameters
SI                    2048
SF             400.0700000 MHz
WDW                   SINE
SSB      0
LB       0 Hz
GB       0
PC                    1.40

F1 - Processing parameters
SI                     512
MC2          echo-antiecho
SF             100.5976815 MHz
WDW                  QSINE
SSB                      2
LB       0 Hz
GB       0
```
