# Supplementary material for: Modular Synthesis of α,α-Diaryl α-Amino Esters via Bi(V)-Mediated Arylation/SN2-Displacement of Kukhtin–Ramirez Intermediates
Source: Org Lett. 2022 Oct 24;24(43):8002–7. doi: 10.1021/acs.orglett.2c03201 (PMC9641671; doi:10.1021/acs.orglett.2c03201)
Supplement: Supplementary file 5 — ol2c03201_si_005.zip [file ol2c03201_si_005.zip › FID_18-22/22/22_1H/pdata/1/pcxac8.AC257_product2_1_1.pdf]

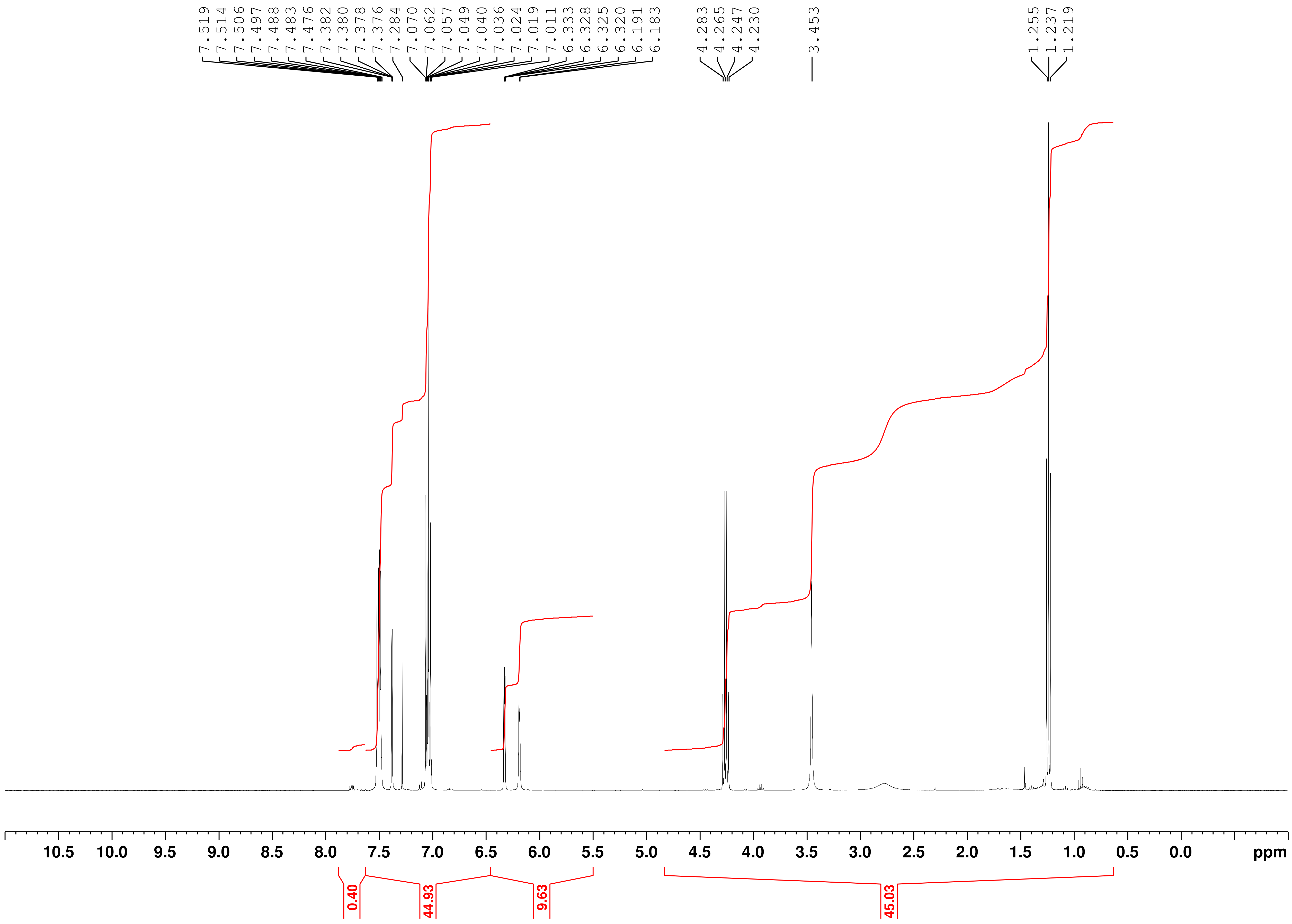


```
Current Data Parameters
NAME      pcxac8.AC257_product2
EXPNO                 1
PROCNO                1

F2 - Acquisition Parameters
Date_          20220201
Time              23.34 h
INSTRUM           av3400
PROBHD   Z104450_0225 (
PULPROG            zg30
TD                65536
SOLVENT           CDCl3
NS                   16
DS                    2
SWH            8223.685 Hz
FIDRES         0.250967 Hz
AQ            3.9845889 sec
RG                  203
DW               60.800 usec
DE                16.03 usec
TE                298.0 K
D1           1.00000000 sec
TD0                   1
SFO1        400.0724706 MHz
NUC1                 1H
P1                14.00 usec
PLW1        11.92800045 W

F2 - Processing parameters
SI                65536
SF          400.0700000 MHz
WDW                  EM
SSB      0
LB                 0.30 Hz
GB       0
PC                 1.00
```
