# Supplementary material for: Modular Synthesis of α,α-Diaryl α-Amino Esters via Bi(V)-Mediated Arylation/SN2-Displacement of Kukhtin–Ramirez Intermediates
Source: Org Lett. 2022 Oct 24;24(43):8002–7. doi: 10.1021/acs.orglett.2c03201 (PMC9641671; doi:10.1021/acs.orglett.2c03201)
Supplement: Supplementary file 5 — ol2c03201_si_005.zip [file ol2c03201_si_005.zip › FID_18-22/22/22_COSY/pdata/1/pcxac8.AC257_product2_3_1.pdf]

Double quantum filtered COSY Spectrum.

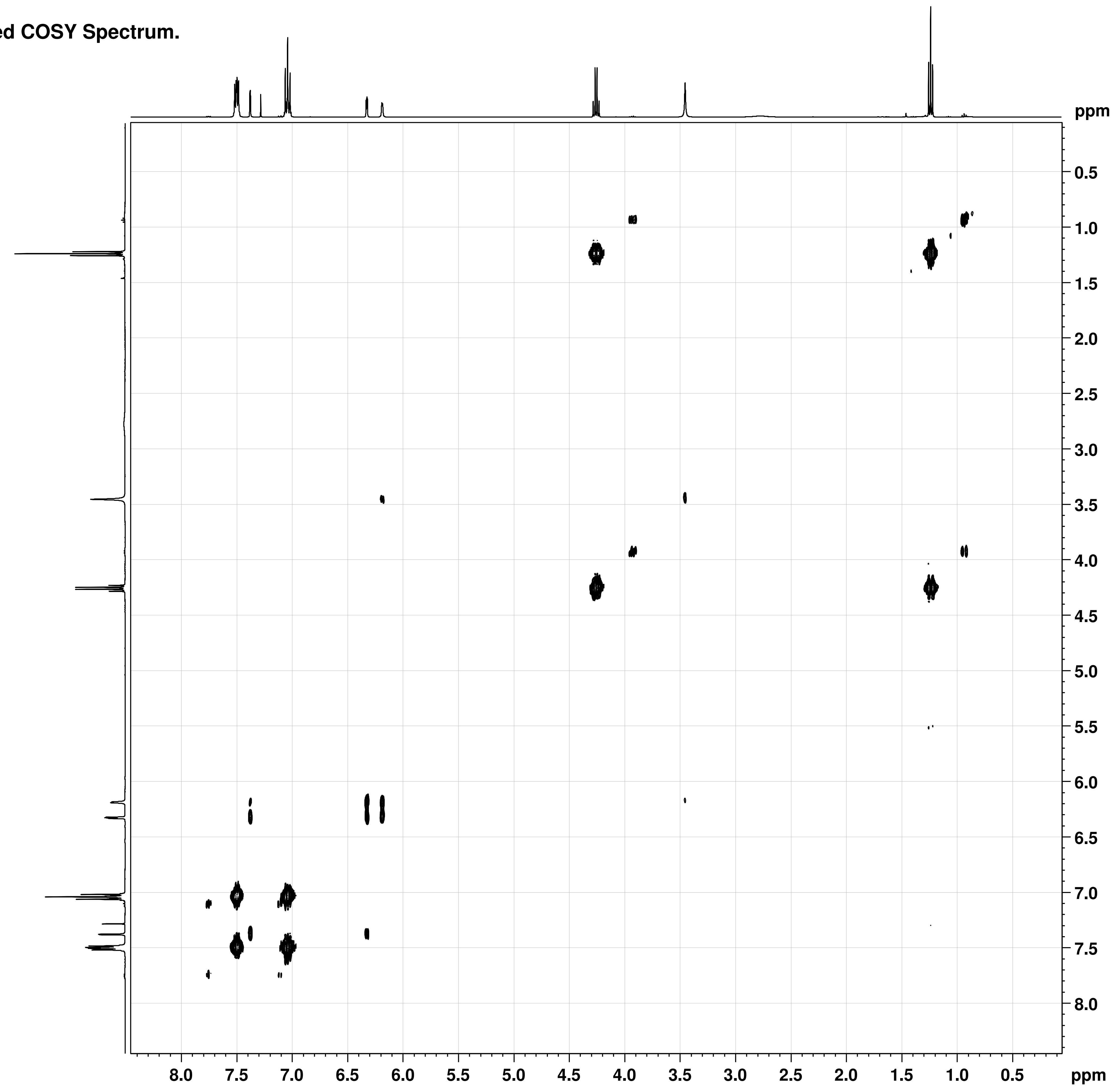


```
Current Data Parameters
NAME     pcxac8.AC257_product2
EXPNO                        3
PROCNO                       1

F2 - Acquisition Parameters
Date_             20220202
Time                  1.36 h
INSTRUM             av3400
PROBHD   Z104450_0225 (
PULPROG    cosygpmfppqf
TD                    2048
SOLVENT              CDCl3
NS                       2
DS                      16
SWH               3698.225 Hz
FIDRES            3.611548 Hz
AQ               0.2768896 sec
RG                    2050
DW                 135.200 usec
DE                    8.70 usec
TE                   298.0 K
D0              0.00000300 sec
D1              0.92067403 sec
D11             0.03000000 sec
D12             0.00002000 sec
D13             0.00000400 sec
D16             0.00020000 sec
IN0             0.00027060 sec
TDav                     1
SFO1           400.0717030 MHz
NUC1                    1H
P1                   14.00 usec
P17                2500.00 usec
PLW1           11.92800045 W
PLW10           2.59770012 W
GPNAM[1]        SMSQ10.100
GPZ1                 16.00 %
GPNAM[2]        SMSQ10.100
GPZ2                 12.00 %
GPNAM[3]        SMSQ10.100
GPZ3                 40.00 %
P16                1000.00 usec

F1 - Acquisition parameters
TD                     128
SFO1              400.0717 MHz
FIDRES           57.742054 Hz
SW                   9.237 ppm
FnMODE                  QF

F2 - Processing parameters
SI                    2048
SF             400.0700000 MHz
WDW                   SINE
SSB      0
LB       0 Hz
GB       0
PC                    1.40

F1 - Processing parameters
SI                     512
MC2                     QF
SF             400.0700000 MHz
WDW                   SINE
SSB      0
LB       0 Hz
GB       0
```
