# Supplementary material for: Modular Synthesis of α,α-Diaryl α-Amino Esters via Bi(V)-Mediated Arylation/SN2-Displacement of Kukhtin–Ramirez Intermediates
Source: Org Lett. 2022 Oct 24;24(43):8002–7. doi: 10.1021/acs.orglett.2c03201 (PMC9641671; doi:10.1021/acs.orglett.2c03201)
Supplement: Supplementary file 5 — ol2c03201_si_005.zip [file ol2c03201_si_005.zip › FID_18-22/22/22_HMBC/pdata/1/pcxac8.AC257_product2_6_1.pdf]

Multiplicity-edited HSQC Spectrum: CH and Me up (black), CH2 down (grey).

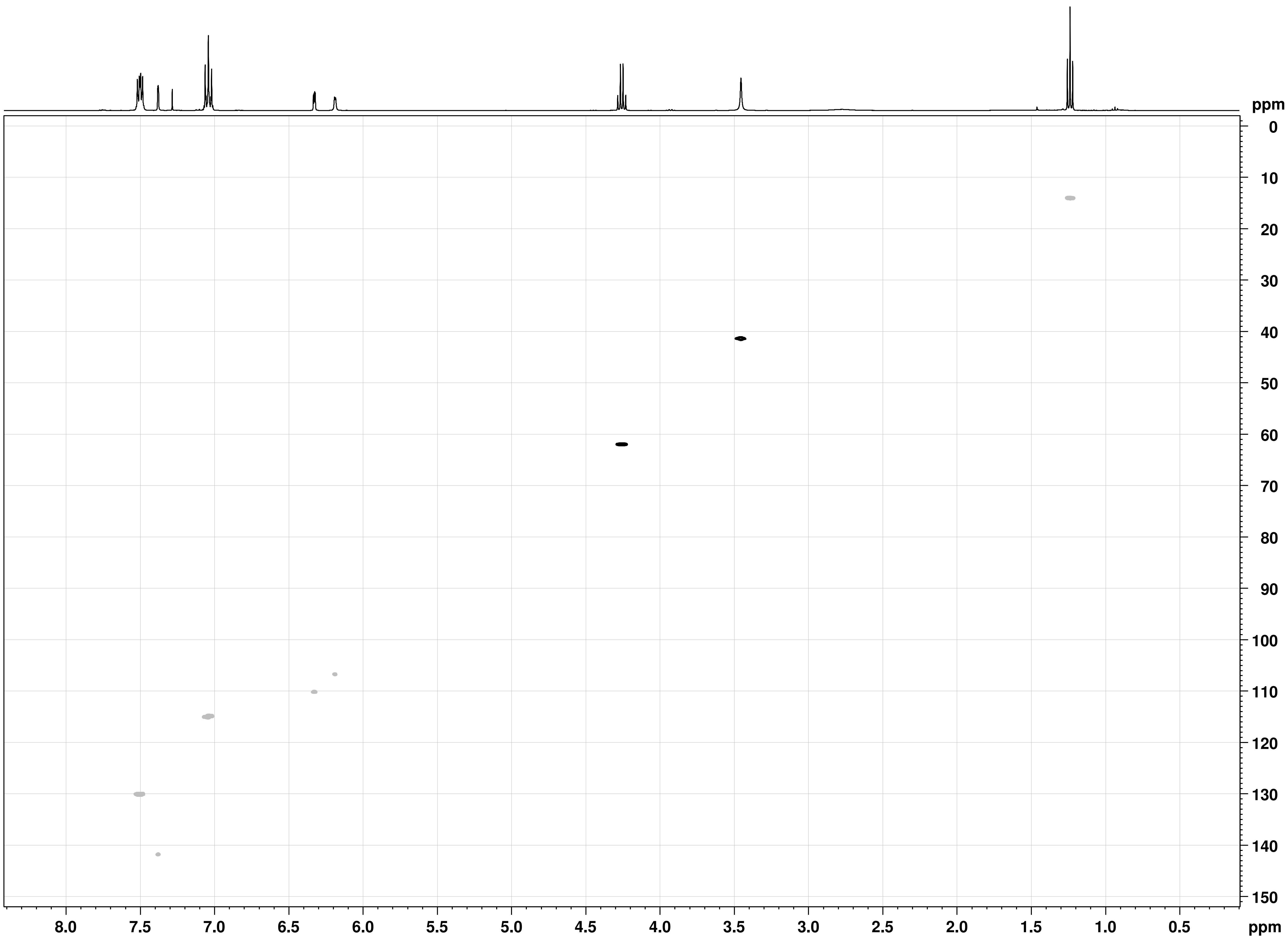


```
Current Data Parameters
NAME     pcxac8.AC257_product2
EXPNO                6
PROCNO               1

F2 - Acquisition Parameters
Date_          20220202
Time               2.09 h
INSTRUM           av3400
PROBHD   Z104450_0225 (
PULPROG  hsqcedetgpsp.3
TD                1190
SOLVENT          CDCl3
NS                   4
DS                  32
SWH           3496.503 Hz
FIDRES        5.876476 Hz
AQ           0.1701700 sec
RG                2050
DW             143.000 usec
DE               6.50 usec
TE               298.0 K
CNST2      145.0000000
D0          0.00000300 sec
D1          1.00000000 sec
D4          0.00172414 sec
D11         0.03000000 sec
D16         0.00020000 sec
D21         0.00360000 sec
IN0         0.00002980 sec
TDav                 1
SFO1       400.0717030 MHz
NUC1                1H
P1               14.00 usec
P2               28.00 usec
P28            1000.00 usec
PLW1       11.92800045 W
SFO2       100.6052263 MHz
NUC2               13C
CPDPRG[2          garp4
P3                9.75 usec
P14             500.00 usec
P31            2119.00 usec
PCPD2            80.00 usec
PLW0     0 W
PLW2       63.68099976 W
PLW12       0.94588000 W
SPNAM[3] Crp60,0.5,20.1
SPOAL3           0.500
SPOFFS3  0 Hz
SPW3        9.24930000 W
SPNAM[18  Crp60_xfilt.2
SPOAL18          0.500
SPOFFS18 0 Hz
SPW18       1.78180003 W
GPNAM[1]     SMSQ10.100
GPZ1             80.00 %
GPNAM[2]     SMSQ10.100
GPZ2             20.10 %
P16            1000.00 usec

F1 - Acquisition parameters
TD                 512
SFO1          100.6052 MHz
FIDRES       65.541107 Hz
SW             166.776 ppm
FnMODE    Echo-Antiecho

F2 - Processing parameters
SI                2048
SF         400.0700000 MHz
WDW              QSINE
SSB                  2
LB       0 Hz
GB       0
PC                1.40

F1 - Processing parameters
SI                1024
MC2       echo-antiecho
SF         100.5976815 MHz
WDW              QSINE
SSB                  2
LB       0 Hz
GB       0
```
