# Supplementary material for: Modular Synthesis of α,α-Diaryl α-Amino Esters via Bi(V)-Mediated Arylation/SN2-Displacement of Kukhtin–Ramirez Intermediates
Source: Org Lett. 2022 Oct 24;24(43):8002–7. doi: 10.1021/acs.orglett.2c03201 (PMC9641671; doi:10.1021/acs.orglett.2c03201)
Supplement: Supplementary file 5 — ol2c03201_si_005.zip [file ol2c03201_si_005.zip › FID_18-22/22/22_HSQC/pdata/1/pcxac8.AC257_product2_5_1.pdf]

HMBC spectrum: very low level one-bond artefacts may be observed.

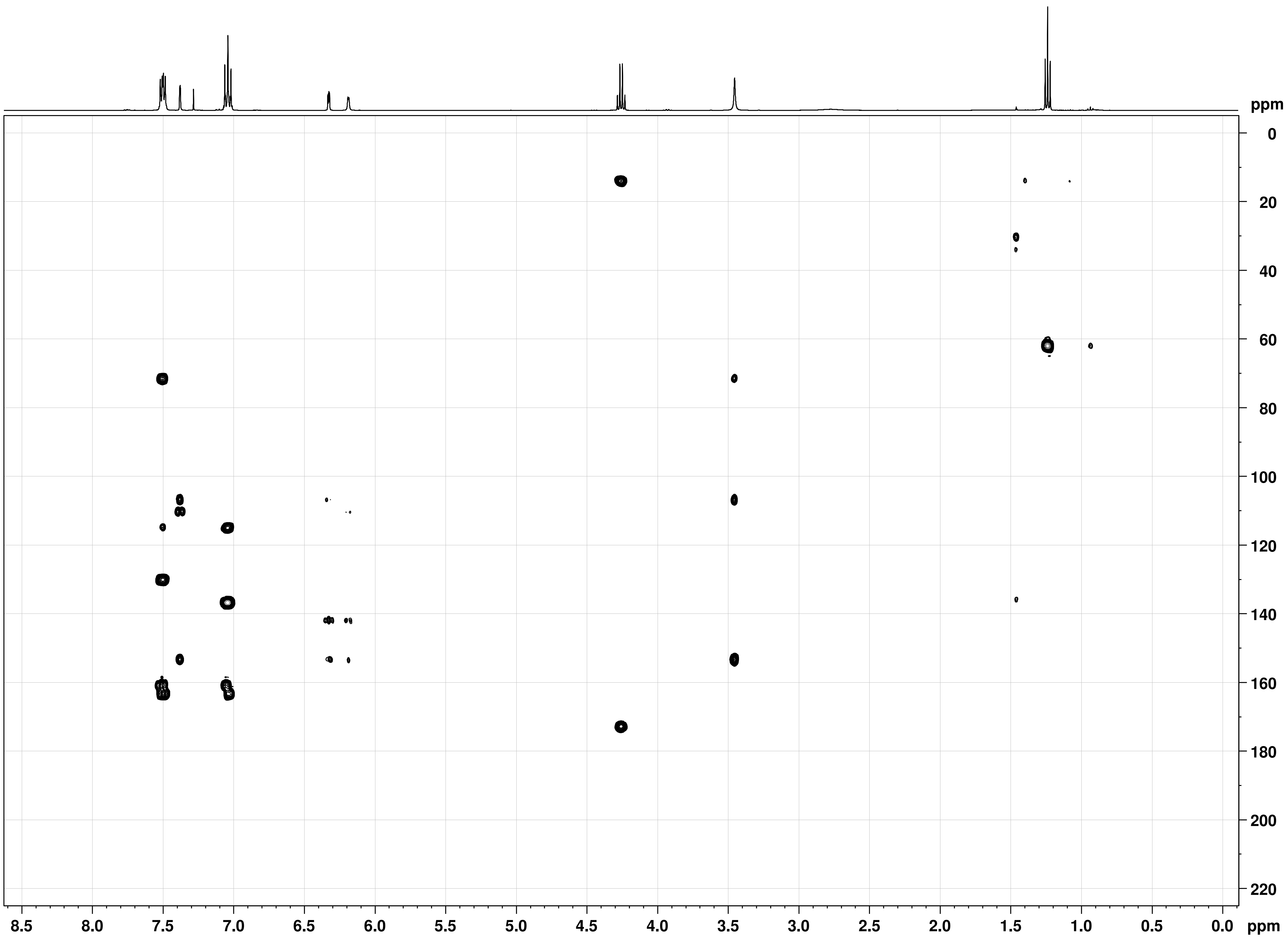


```
Current Data Parameters
NAME      pcxac8.AC257_product2
EXPNO                     5
PROCNO                    1

F2 - Acquisition Parameters
Date_              20220202
Time                 1.45 h
INSTRUM               av3400
PROBHD   Z104450_0225 (
PULPROG    hmbcetgpl3nd
TD                     1190
SOLVENT               CDC13
NS                        8
DS                       16
SWH               3496.503 Hz
FIDRES            5.876476 Hz
AQ               0.1701700 sec
RG                     2050
DW                  143.000 usec
DE                    6.50 usec
TE                   298.0 K
CNST6           120.0000000
CNST7           210.0000000
CNST13            8.5000000
D0               0.00000300 sec
D1               1.00000000 sec
D6               0.05882353 sec
D16              0.00020000 sec
IN0              0.00002160 sec
TDav                      1
SFO1          400.0717030 MHz
NUC1                     1H
P1                    14.00 usec
P2                    28.00 usec
PLW1            11.92800045 W
SFO2          100.6087473 MHz
NUC2                    13C
P3                     9.75 usec
P24                 2000.00 usec
PLW2            63.68099976 W
SPNAM[7]     Crp60comp.4
SPOAL7                0.500
SPOFFS7  0 Hz
SPW7             9.24930000 W
GPNAM[1]      SMSQ10.100
GPZ1                  80.00 %
GPNAM[3]      SMSQ10.100
GPZ3                  14.00 %
GPNAM[4]      SMSQ10.100
GPZ4                  -8.00 %
GPNAM[5]      SMSQ10.100
GPZ5                  -4.00 %
GPNAM[6]      SMSQ10.100
GPZ6                  -2.00 %
P16                 1000.00 usec

F1 - Acquisition parameters
TD                      128
SFO1           100.6087 MHz
FIDRES        361.689819 Hz
SW               230.081 ppm
FnMODE     Echo-Antiecho

F2 - Processing parameters
SI                     2048
SF            400.0700000 MHz
WDW                    SINE
SSB       0
LB        0 Hz
GB        0
PC                     1.40

F1 - Processing parameters
SI                      512
MC2         echo-antiecho
SF            100.5976815 MHz
WDW                   QSINE
SSB                       2
LB        0 Hz
GB        0
```
