# Supplementary material for: Modular Synthesis of α,α-Diaryl α-Amino Esters via Bi(V)-Mediated Arylation/SN2-Displacement of Kukhtin–Ramirez Intermediates
Source: Org Lett. 2022 Oct 24;24(43):8002–7. doi: 10.1021/acs.orglett.2c03201 (PMC9641671; doi:10.1021/acs.orglett.2c03201)
Supplement: Supplementary file 6 — ol2c03201_si_006.zip [file ol2c03201_si_006.zip › FID_23-27/24/24_13C/pdata/1/pcxac8.AC335_F2_prod_dry_3_1.pdf]

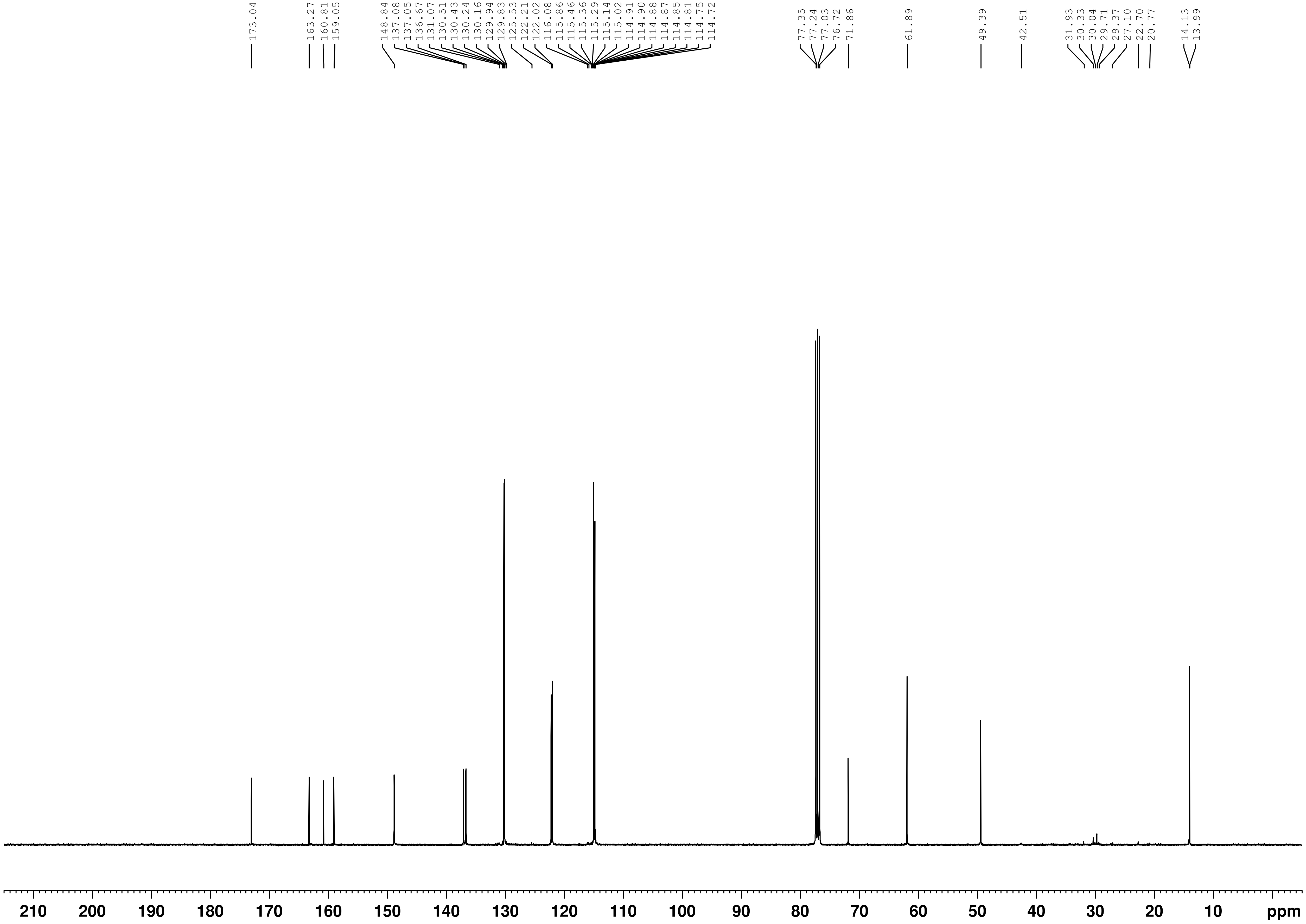

Current Data Parameters
NAME pcxac8.AC335_F2_prod_dry
EXPNO 3
PROCNO 1

F2 - Acquisition Parameters
Date_ 20220506
Time 23.11 h
INSTRUM av3400hd
PROBHD Z122623_0053 (
PULPROG zgpg30
TD 32768
SOLVENT CDCl3
NS 4096
DS 4
SWH 23809.523 Hz
FIDRES 1.453218 Hz
AQ 0.6881280 sec
RG 140.92
DW 21.000 usec
DE 18.80 usec
TE 298.0 K
D1 1.00000000 sec
D11 0.03000000 sec
TD0 1
SFO1 100.6414394 MHz
NUC1 13C
P1 10.19 usec
PLW1 38.00000000 W
SFO2 400.2016008 MHz
NUC2 1H
CPDPRG[2 waltz16
PCPD2 90.00 usec
PLW2 10.00000000 W
PLW12 0.12895000 W
PLW13 0.06486000 W

F2 - Processing parameters
SI 32768
SF 100.6303700 MHz
WDW EM
SSB 0
LB 1.00 Hz
GB 0
PC 1.40
