# Supplementary material for: Modular Synthesis of α,α-Diaryl α-Amino Esters via Bi(V)-Mediated Arylation/SN2-Displacement of Kukhtin–Ramirez Intermediates
Source: Org Lett. 2022 Oct 24;24(43):8002–7. doi: 10.1021/acs.orglett.2c03201 (PMC9641671; doi:10.1021/acs.orglett.2c03201)
Supplement: Supplementary file 6 — ol2c03201_si_006.zip [file ol2c03201_si_006.zip › FID_23-27/25/25_1H/pdata/1/pcxac8.AC249_productdry_1_1.pdf]

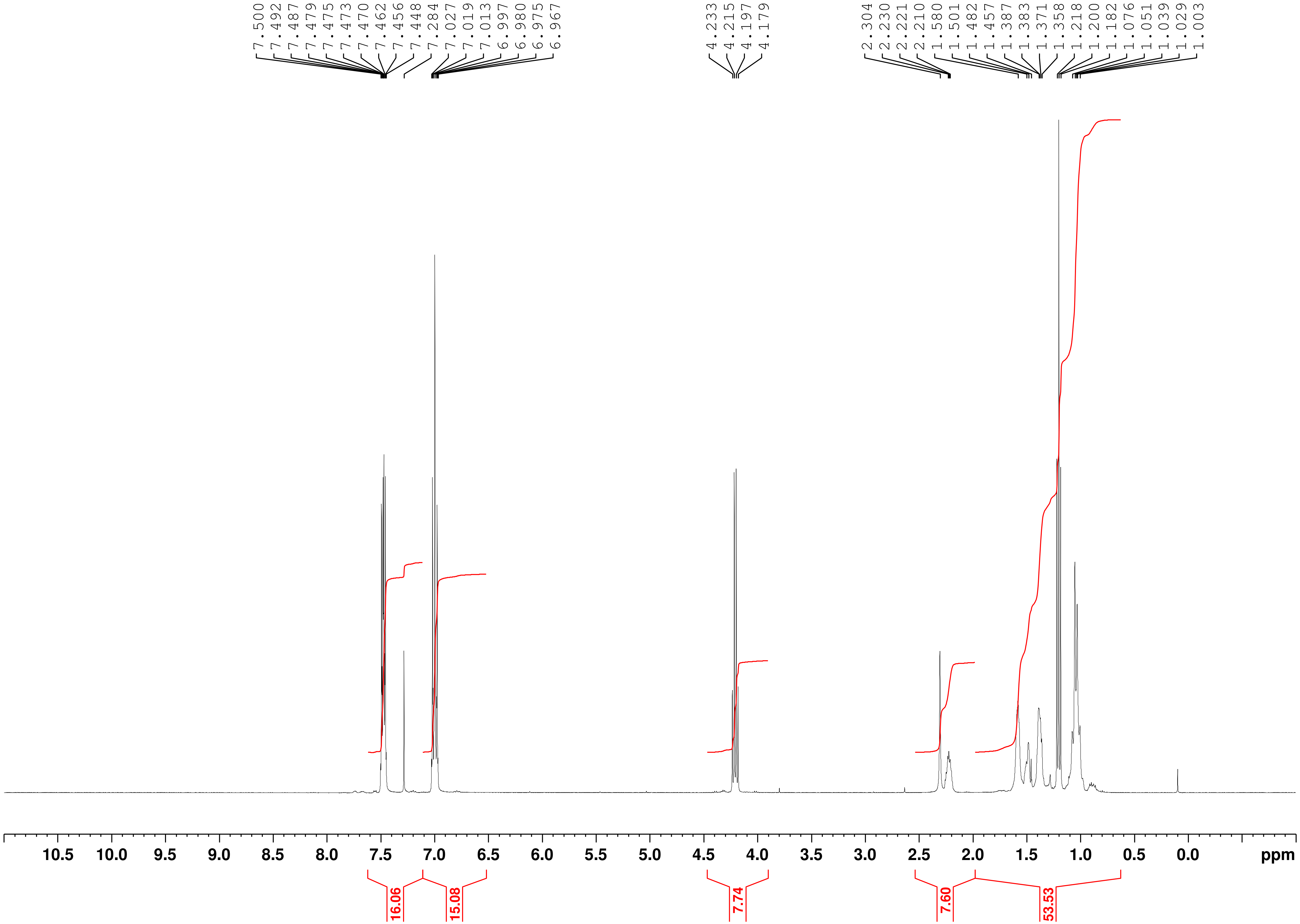


```
Current Data Parameters
NAME      pcxac8.AC249_productdry
EXPNO                   1
PROCNO                  1

F2 - Acquisition Parameters
Date_            20220119
Time                16.20 h
INSTRUM            av3400
PROBHD   Z104450_0225 (
PULPROG              zg30
TD                  65536
SOLVENT             CDCl3
NS                     16
DS                      2
SWH              8223.685 Hz
FIDRES           0.250967 Hz
AQ              3.9845889 sec
RG                    228
DW                 60.800 usec
DE                  16.03 usec
TE                  298.0 K
D1             1.00000000 sec
TD0                     1
SFO1          400.0724706 MHz
NUC1                   1H
P1                  14.00 usec
PLW1          11.92800045 W

F2 - Processing parameters
SI                  65536
SF            400.0700000 MHz
WDW                    EM
SSB      0
LB                   0.30 Hz
GB       0
PC                   1.00
```
