# Supplementary material for: Modular Synthesis of α,α-Diaryl α-Amino Esters via Bi(V)-Mediated Arylation/SN2-Displacement of Kukhtin–Ramirez Intermediates
Source: Org Lett. 2022 Oct 24;24(43):8002–7. doi: 10.1021/acs.orglett.2c03201 (PMC9641671; doi:10.1021/acs.orglett.2c03201)
Supplement: Supplementary file 6 — ol2c03201_si_006.zip [file ol2c03201_si_006.zip › FID_23-27/25/25_COSY/pdata/1/pcxac8.AC249_productdry_3_1.pdf]

Double quantum filtered COSY Spectrum.

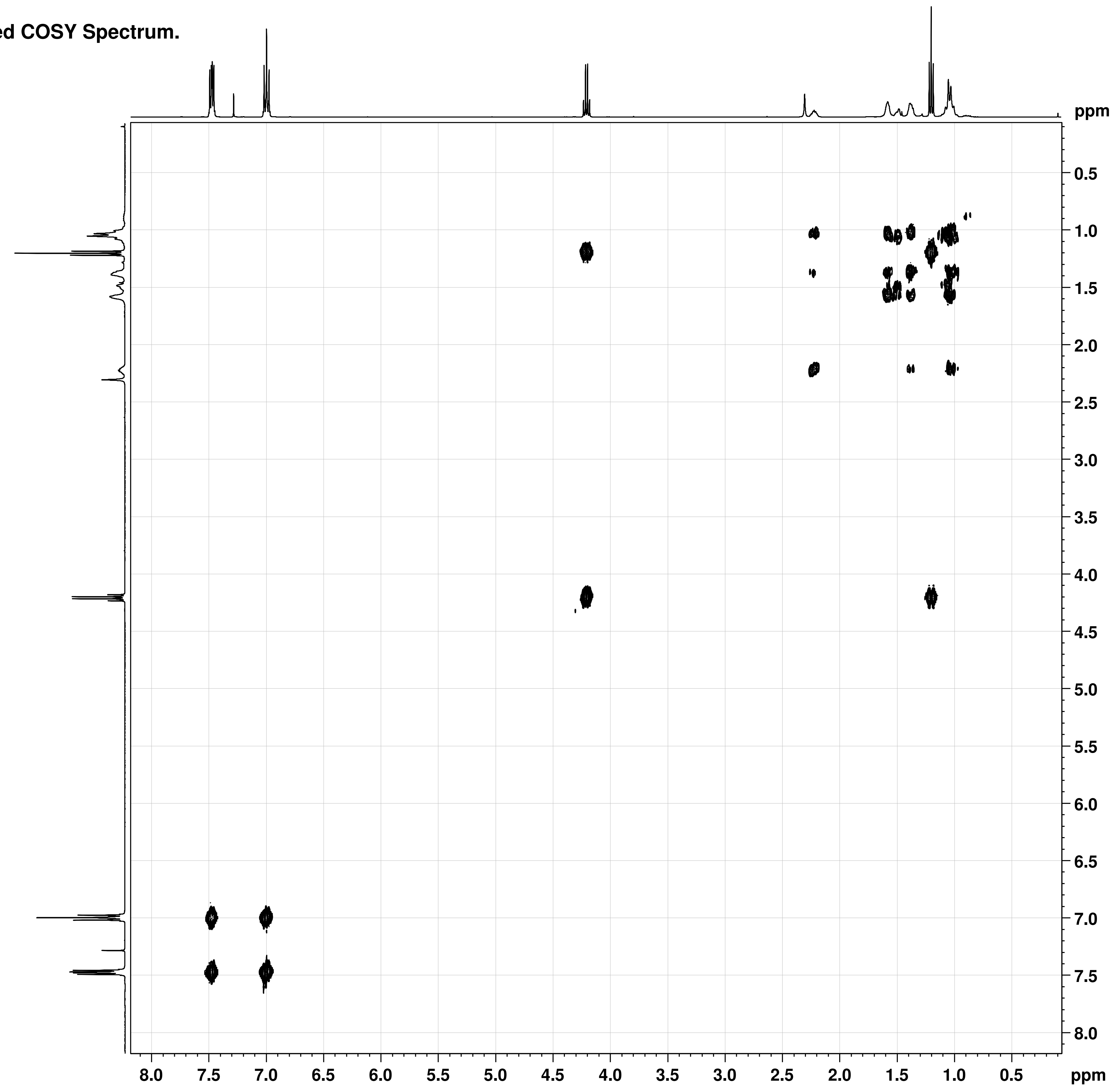

Current Data Parameters
NAME pcxac8.AC249_productd
EXPNO 3
PROCNO 1

F2 - Acquisition Parameters
Date_ 20220120
Time 6.08 h
INSTRUM av3400
PROBHD Z104450_0225 (
PULPROG cosygpmfppqf
TD 2048
SOLVENT CDCl3
NS 2
DS 16
SWH 3578.244 Hz
FIDRES 3.494379 Hz
AQ 0.2861739 sec
RG 2050
DW 139.733 usec
DE 9.07 usec
TE 298.0 K
D0 0.00000300 sec
D1 0.91043401 sec
D11 0.03000000 sec
D12 0.00002000 sec
D13 0.00000400 sec
D16 0.00020000 sec
IN0 0.00027940 sec
TDav 1
SFO1 400.0716500 MHz
NUC1 1H
P1 14.00 usec
P17 2500.00 usec
PLW1 11.92800045 W
PLW10 2.59770012 W
GPNAM[1] SMSQ10.100
GPZ1 16.00 %
GPNAM[2] SMSQ10.100
GPZ2 12.00 %
GPNAM[3] SMSQ10.100
GPZ3 40.00 %
P16 1000.00 usec

F1 - Acquisition parameters
TD 128
SFO1 400.0716 MHz
FIDRES 55.923409 Hz
SW 8.946 ppm
FnMODE QF

F2 - Processing parameters
SI 2048
SF 400.0700000 MHz
WDW SINE
SSB 0
LB 0 Hz
GB 0
PC 1.40

F1 - Processing parameters
SI 512
MC2 QF
SF 400.0700000 MHz
WDW SINE
SSB 0
LB 0 Hz
GB 0
