# Supplementary material for: Modular Synthesis of α,α-Diaryl α-Amino Esters via Bi(V)-Mediated Arylation/SN2-Displacement of Kukhtin–Ramirez Intermediates
Source: Org Lett. 2022 Oct 24;24(43):8002–7. doi: 10.1021/acs.orglett.2c03201 (PMC9641671; doi:10.1021/acs.orglett.2c03201)
Supplement: Supplementary file 6 — ol2c03201_si_006.zip [file ol2c03201_si_006.zip › FID_23-27/25/25_HMBC/pdata/1/pcxac8.AC249_productdry_5_1.pdf]

HMBC spectrum: very low level one-bond artefacts may be observed.

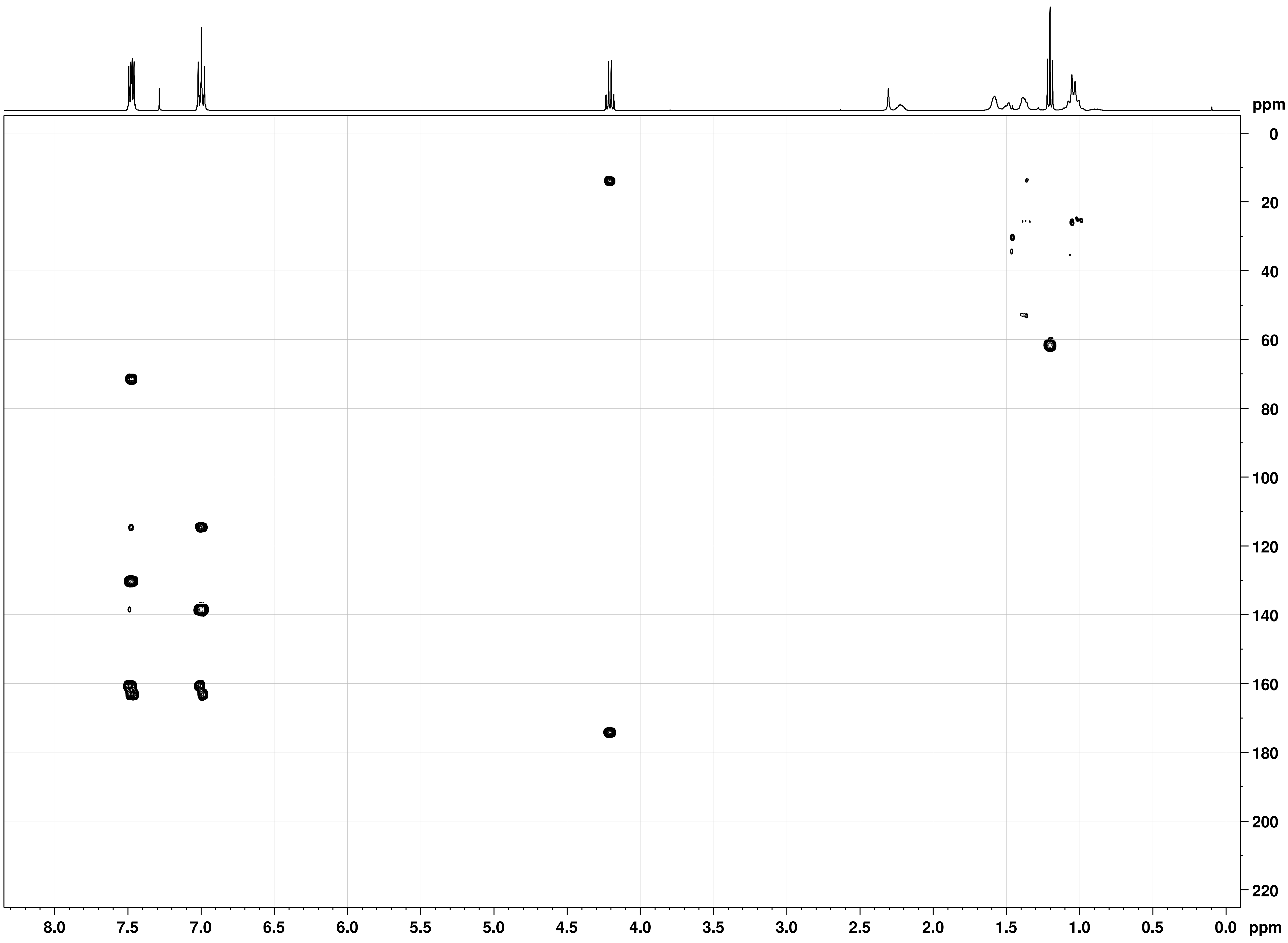


```
Current Data Parameters
NAME     pcxac8.AC249_productdry
EXPNO                     5
PROCNO                    1

F2 - Acquisition Parameters
Date_              20220120
Time                 6.16 h
INSTRUM              av3400
PROBHD    Z104450_0225 (
PULPROG     hmbcetgpl3nd
TD                     1190
SOLVENT               CDCl3
NS                        8
DS                       16
SWH              3378.378 Hz
FIDRES           5.677947 Hz
AQ              0.1761200 sec
RG                     2050
DW                  148.000 usec
DE                     6.50 usec
TE                    298.0 K
CNST6        120.0000000
CNST7        210.0000000
CNST13         8.5000000
D0            0.00000300 sec
D1            1.00000000 sec
D6            0.05882353 sec
D16           0.00020000 sec
IN0           0.00002160 sec
TDav                      1
SFO1         400.0716500 MHz
NUC1                     1H
P1                    14.00 usec
P2                    28.00 usec
PLW1         11.92800045 W
SFO2         100.6087473 MHz
NUC2                    13C
P3                     9.75 usec
P24                 2000.00 usec
PLW2         63.68099976 W
SPNAM[7]     Crp60comp.4
SPOAL7                0.500
SPOFFS7  0 Hz
SPW7          9.24930000 W
GPNAM[1]      SMSQ10.100
GPZ1                  80.00 %
GPNAM[3]      SMSQ10.100
GPZ3                  14.00 %
GPNAM[4]      SMSQ10.100
GPZ4                  -8.00 %
GPNAM[5]      SMSQ10.100
GPZ5                  -4.00 %
GPNAM[6]      SMSQ10.100
GPZ6                  -2.00 %
P16                 1000.00 usec

F1 - Acquisition parameters
TD                      128
SFO1             100.6087 MHz
FIDRES         361.689819 Hz
SW                  230.081 ppm
FnMODE      Echo-Antiecho

F2 - Processing parameters
SI                     2048
SF           400.0700000 MHz
WDW                    SINE
SSB      0
LB       0 Hz
GB       0
PC                     1.40

F1 - Processing parameters
SI                      512
MC2         echo-antiecho
SF           100.5976815 MHz
WDW                   QSINE
SSB                       2
LB       0 Hz
GB       0
```
