# Supplementary material for: Modular Synthesis of α,α-Diaryl α-Amino Esters via Bi(V)-Mediated Arylation/SN2-Displacement of Kukhtin–Ramirez Intermediates
Source: Org Lett. 2022 Oct 24;24(43):8002–7. doi: 10.1021/acs.orglett.2c03201 (PMC9641671; doi:10.1021/acs.orglett.2c03201)
Supplement: Supplementary file 6 — ol2c03201_si_006.zip [file ol2c03201_si_006.zip › FID_23-27/26/26_HMBC/pdata/1/pcxac8.AC312_productdry_4_1.pdf]

HMBC spectrum: very low level one-bond artefacts may be observed.

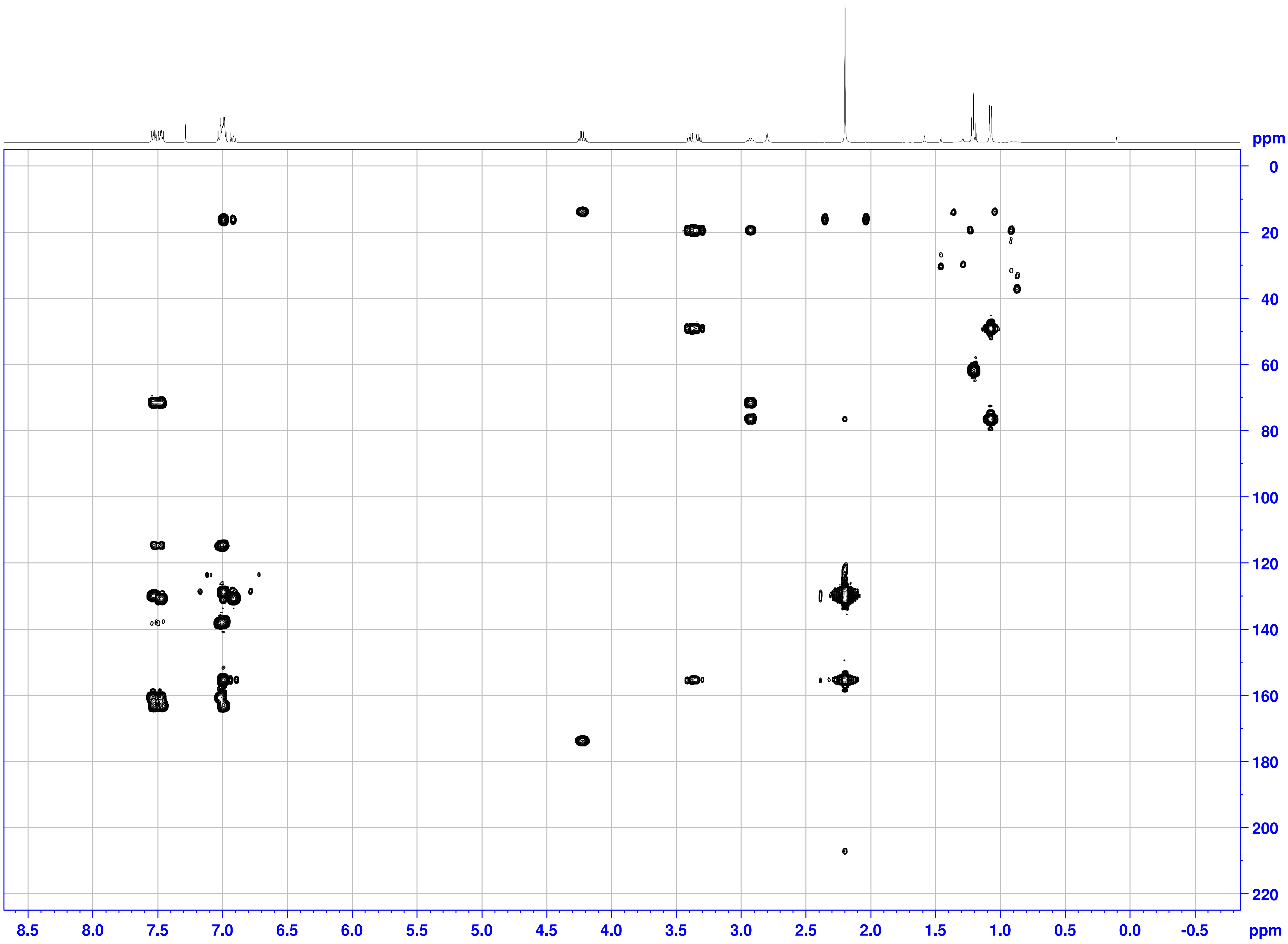


```
Current Data Parameters
NAME      pcxac8.AC312_productdry
EXPNO                    4
PROCNO                   1

F2 - Acquisition Parameters
Date_             20220331
Time                0.36 h
INSTRUM          av3400hd
PROBHD    Z122623_0053 (
PULPROG     hmbcetgp13nd
TD                    1190
SOLVENT              CDCl3
NS                       8
DS                      16
SWH            3816.794 Hz
FIDRES         6.414780 Hz
AQ            0.1558900 sec
RG                  198.43
DW                 131.000 usec
DE                   10.00 usec
TE                   298.0 K
CNST6          120.0000000
CNST7          175.0000000
CNST13           8.5000000
D0             0.00000300 sec
D1             1.00000000 sec
D6             0.05882353 sec
D16            0.00020000 sec
IN0            0.00002160 sec
TDav                     1
SFO1           400.2015680 MHz
NUC1                    1H
P1                   10.22 usec
P2                   20.44 usec
PLW1           10.00000000 W
SFO2           100.6414394 MHz
NUC2                   13C
P3                   10.70 usec
P24                2000.00 usec
PLW2           38.00000000 W
SPNAM[7]      Crp60comp.4
SPOAL7               0.500
SPOFFS7   0 Hz
SPW7            6.64729977 W
GPNAM[1]        SMSQ10.100
GPZ1                 80.00 %
GPNAM[3]        SMSQ10.100
GPZ3                 14.00 %
GPNAM[4]        SMSQ10.100
GPZ4                 -8.00 %
GPNAM[5]        SMSQ10.100
GPZ5                 -4.00 %
GPNAM[6]        SMSQ10.100
GPZ6                 -2.00 %
P16                1000.00 usec

F1 - Acquisition parameters
TD                     128
SFO1              100.6414 MHz
FIDRES          361.689819 Hz
SW                 230.006 ppm
FnMODE        Echo-Antiecho

F2 - Processing parameters
SI                    2048
SF             400.2000000 MHz
WDW                   SINE
SSB       0
LB        0 Hz
GB        0
PC                    1.40

F1 - Processing parameters
SI                     512
MC2          echo-antiecho
SF             100.6303700 MHz
WDW                  QSINE
SSB                      2
LB        0 Hz
GB        0
```
