# Supplementary material for: Modular Synthesis of α,α-Diaryl α-Amino Esters via Bi(V)-Mediated Arylation/SN2-Displacement of Kukhtin–Ramirez Intermediates
Source: Org Lett. 2022 Oct 24;24(43):8002–7. doi: 10.1021/acs.orglett.2c03201 (PMC9641671; doi:10.1021/acs.orglett.2c03201)
Supplement: Supplementary file 6 — ol2c03201_si_006.zip [file ol2c03201_si_006.zip › FID_23-27/27/27_13C/pdata/1/email_pcxac8.AC363_column_2_1.pdf]

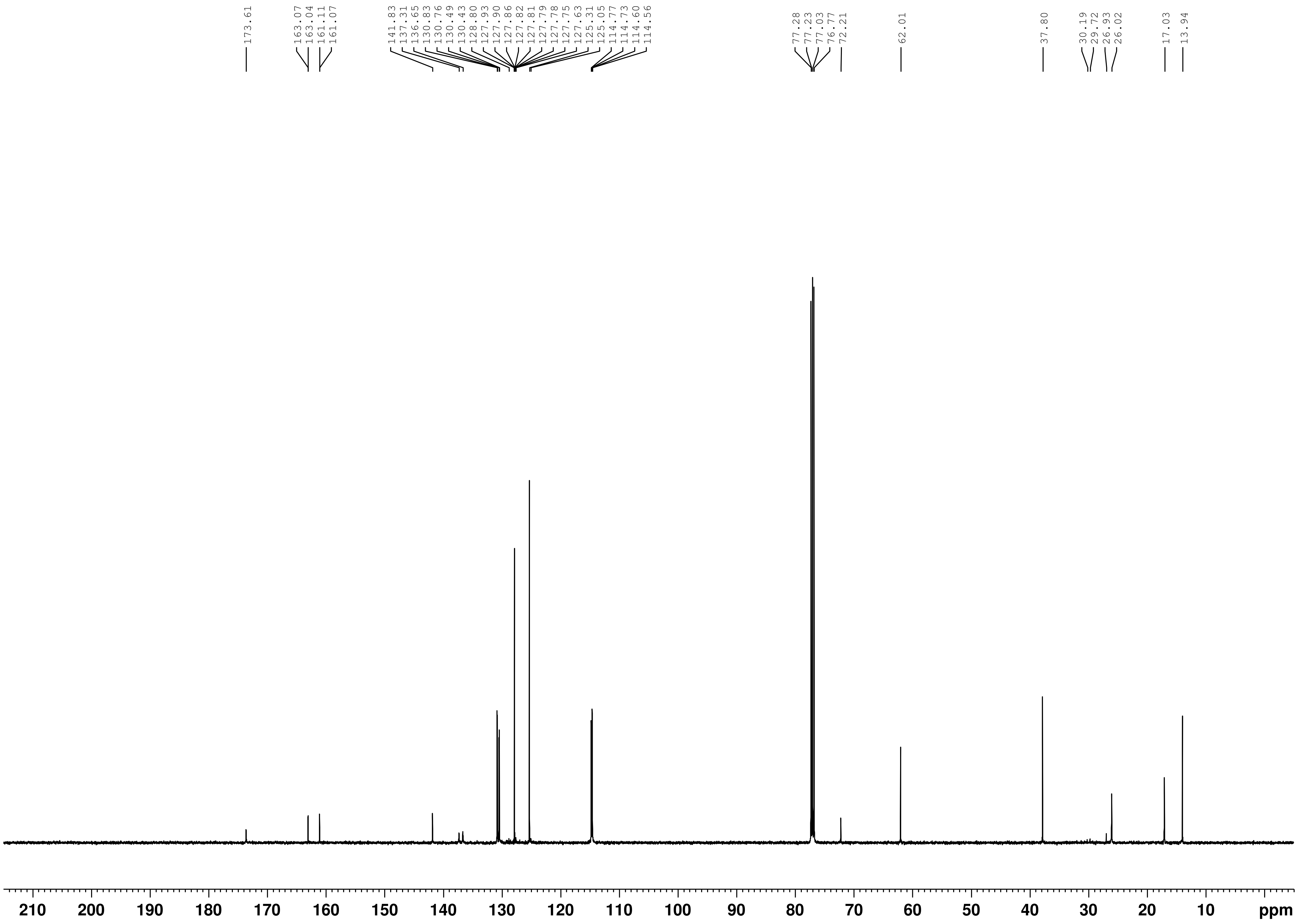


```
Current Data Parameters
NAME     pcxac8.AC363_column
EXPNO                  2
PROCNO                 1

F2 - Acquisition Parameters
Date_           20220611
Time               13.28 h
INSTRUM           av3500
PROBHD   Z8007_0120 (PH
PULPROG          zgpg30
TD                 32768
SOLVENT            CDCl3
NS                  6000
DS                    32
SWH            29761.904 Hz
FIDRES          1.816522 Hz
AQ             0.5505024 sec
RG                  2050
DW                16.800 usec
DE                  9.58 usec
TE                 298.0 K
D1            1.00000000 sec
D11           0.03000000 sec
TD0                    1
SFO1         125.7716219 MHz
NUC1                 13C
P1                  9.25 usec
PLW1         59.84099960 W
SFO2         500.1320005 MHz
NUC2                  1H
CPDPRG[2         waltz16
PCPD2              80.00 usec
PLW2         29.99200058 W
PLW12         0.46862000 W
PLW13         0.23571000 W

F2 - Processing parameters
SI                 32768
SF           125.7577885 MHz
WDW                   EM
SSB      0
LB                  1.00 Hz
GB       0
PC                  1.40
```
