# Supplementary material for: Modular Synthesis of α,α-Diaryl α-Amino Esters via Bi(V)-Mediated Arylation/SN2-Displacement of Kukhtin–Ramirez Intermediates
Source: Org Lett. 2022 Oct 24;24(43):8002–7. doi: 10.1021/acs.orglett.2c03201 (PMC9641671; doi:10.1021/acs.orglett.2c03201)
Supplement: Supplementary file 6 — ol2c03201_si_006.zip [file ol2c03201_si_006.zip › FID_23-27/27/27_HSQC/pdata/1/email_pcxac8.AC363_column_4_1.pdf]

Multiplicity-edited HSQC Spectrum: CH and Me up (black), CH2 down (grey).

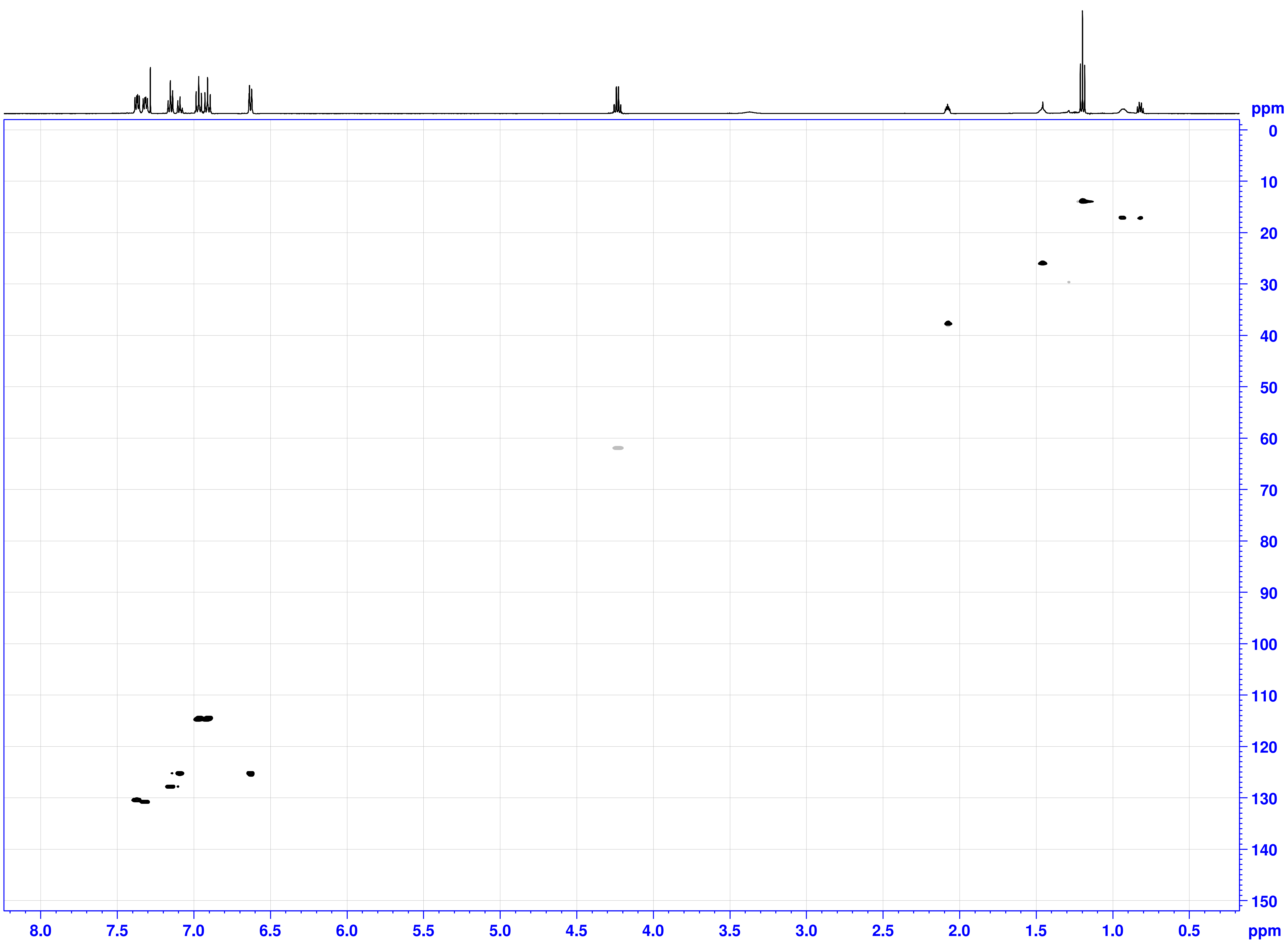


```
Current Data Parameters
NAME      pcxac8.AC363_column
EXPNO                       4
PROCNO                      1

F2 - Acquisition Parameters
Date_              20220611
Time                  13.35 h
INSTRUM               av3500
PROBHD   Z8007_0120 (PH
PULPROG  hsqcedetgpsp.3
TD                     1190
SOLVENT               CDCl3
NS                        8
DS                       32
SWH                4237.288 Hz
FIDRES             7.121493 Hz
AQ                0.1404200 sec
RG                     2050
DW                  118.000 usec
DE                     6.50 usec
TE                    298.0 K
CNST2         145.0000000
D0             0.00000300 sec
D1             1.00000000 sec
D4             0.00172414 sec
D11            0.03000000 sec
D16            0.00020000 sec
D21            0.00360000 sec
IN0            0.00002380 sec
TDav                      1
SFO1         500.1321041 MHz
NUC1                     1H
P1                    10.00 usec
P2                    20.00 usec
P28                 1000.00 usec
PLW1         29.99200058 W
SFO2         125.7672204 MHz
NUC2                    13C
CPDPRG[2              garp4
P3                     9.25 usec
P14                  500.00 usec
P31                 1895.00 usec
PCPD2                 81.24 usec
PLW0      0 W
PLW2         59.84099960 W
PLW12         0.77578998 W
SPNAM[3] Crp60,0.5,20.1
SPOAL3                0.500
SPOFFS3  0 Hz
SPW3          7.82299995 W
SPNAM[18  Crp60_xfilt.2
SPOAL18               0.500
SPOFFS18 0 Hz
SPW18         1.88440001 W
GPNAM[1]       SMSQ10.100
GPZ1                  80.00 %
GPNAM[2]       SMSQ10.100
GPZ2                  20.10 %
P16                 1000.00 usec

F1 - Acquisition parameters
TD                      512
SFO1            125.7672 MHz
FIDRES         82.064079 Hz
SW               167.042 ppm
FnMODE      Echo-Antiecho

F2 - Processing parameters
SI                     2048
SF           500.1300000 MHz
WDW                   QSINE
SSB                       2
LB        0 Hz
GB        0
PC                     1.40

F1 - Processing parameters
SI                      512
MC2            echo-antiecho
SF           125.7577885 MHz
WDW                   QSINE
SSB                       2
LB        0 Hz
GB        0
```
