# Supplementary material for: Modular Synthesis of α,α-Diaryl α-Amino Esters via Bi(V)-Mediated Arylation/SN2-Displacement of Kukhtin–Ramirez Intermediates
Source: Org Lett. 2022 Oct 24;24(43):8002–7. doi: 10.1021/acs.orglett.2c03201 (PMC9641671; doi:10.1021/acs.orglett.2c03201)
Supplement: Supplementary file 7 — ol2c03201_si_007.zip [file ol2c03201_si_007.zip › FID_28-32/29/29_COSY/pdata/1/pcxac8.AC367_F2_4_1.pdf]

Double quantum filtered COSY Spectrum.

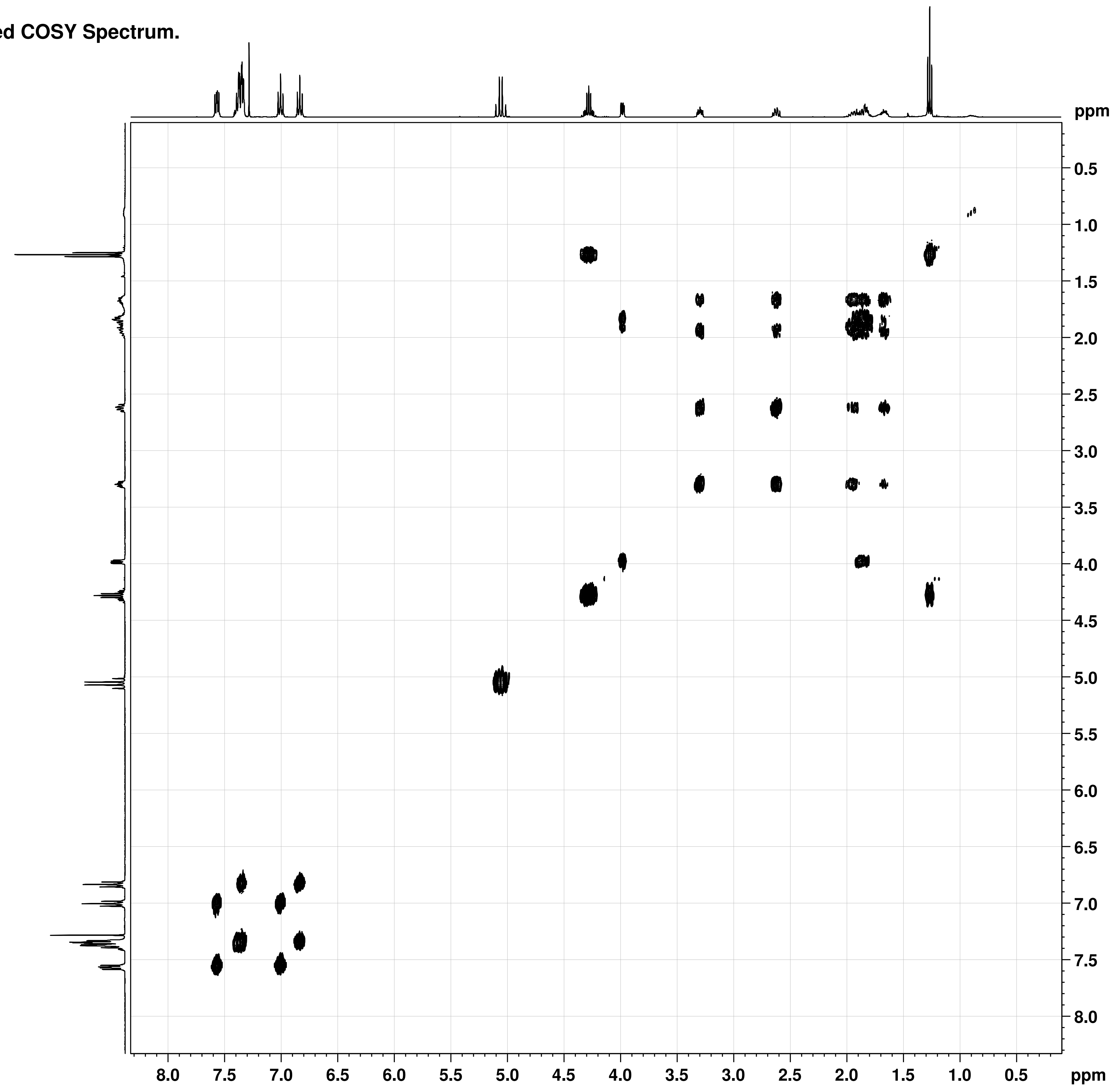


```
Current Data Parameters
NAME       pcxac8.AC367_F2
EXPNO                    4
PROCNO                   1

F2 - Acquisition Parameters
Date_             20220622
Time               21.05 h
INSTRUM             av3400
PROBHD   Z104450_0225 (
PULPROG    cosygpmfppqf
TD                    2048
SOLVENT              CDCl3
NS                       2
DS                      16
SWH             3625.816 Hz
FIDRES          3.540836 Hz
AQ             0.2824192 sec
RG                    2050
DW               137.900 usec
DE                  8.84 usec
TE                 298.0 K
D0            0.00000300 sec
D1            0.91452998 sec
D11           0.03000000 sec
D12           0.00002000 sec
D13           0.00000400 sec
D16           0.00020000 sec
IN0           0.00027580 sec
TDav                     1
SFO1         400.0716869 MHz
NUC1                    1H
P1                 14.00 usec
P17              2500.00 usec
PLW1         11.92800045 W
PLW10         2.59770012 W
GPNAM[1]      SMSQ10.100
GPZ1               16.00 %
GPNAM[2]      SMSQ10.100
GPZ2               12.00 %
GPNAM[3]      SMSQ10.100
GPZ3               40.00 %
P16              1000.00 usec

F1 - Acquisition parameters
TD                     128
SFO1            400.0717 MHz
FIDRES         56.653374 Hz
SW                 9.063 ppm
FnMODE                  QF

F2 - Processing parameters
SI                    2048
SF           400.0700000 MHz
WDW                   SINE
SSB       0
LB        0 Hz
GB        0
PC                    1.40

F1 - Processing parameters
SI                     512
MC2                     QF
SF           400.0700000 MHz
WDW                   SINE
SSB       0
LB        0 Hz
GB        0
```
