# Supplementary material for: Modular Synthesis of α,α-Diaryl α-Amino Esters via Bi(V)-Mediated Arylation/SN2-Displacement of Kukhtin–Ramirez Intermediates
Source: Org Lett. 2022 Oct 24;24(43):8002–7. doi: 10.1021/acs.orglett.2c03201 (PMC9641671; doi:10.1021/acs.orglett.2c03201)
Supplement: Supplementary file 7 — ol2c03201_si_007.zip [file ol2c03201_si_007.zip › FID_28-32/31/31_19F/4/pdata/1/pcxac8.AC304_productdry_4_1.pdf]

Processed with back linear prediction to eliminate baseline artefacts: broad peaks may be attenuated. Standard data in file 100004

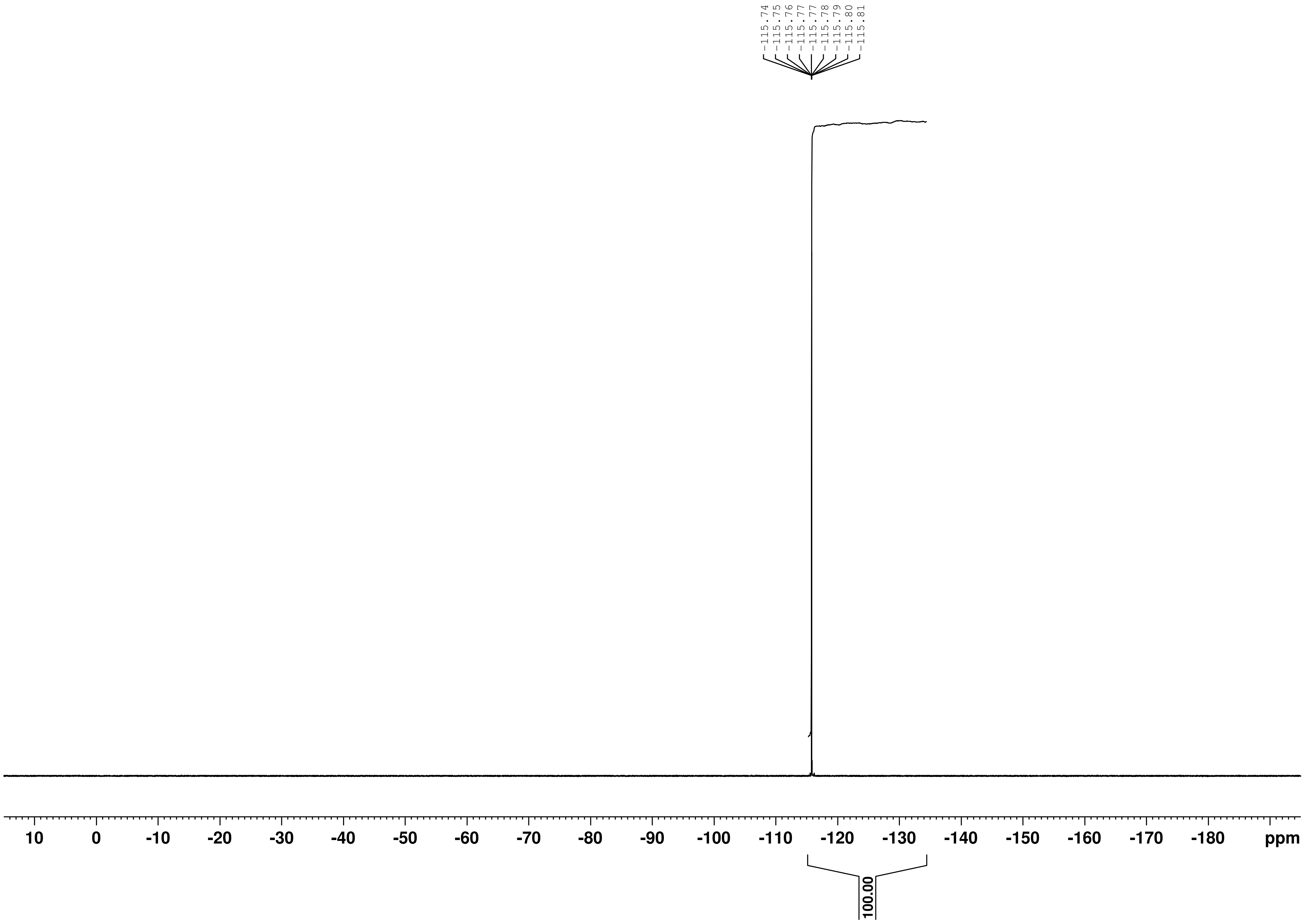


```
Current Data Parameters
NAME     pcxac8.AC304_productdry
EXPNO                  4
PROCNO                 1

F2 - Acquisition Parameters
Date_          20220318
Time              23.17 h
INSTRUM          av3400
PROBHD   Z104450_0225 (
PULPROG          zgflqn
TD               261992
SOLVENT           CDCl3
NS                   16
DS                    4
SWH          85227.273 Hz
FIDRES        0.650610 Hz
AQ           1.5370197 sec
RG                1030
DW               5.867 usec
DE                6.77 usec
TE               298.0 K
D1          1.50000000 sec
TD0                  1
SFO1       376.4080300 MHz
NUC1               19F
P1               13.30 usec
PLW1       16.11100006 W

F2 - Processing parameters
SI              262144
SF         376.4419098 MHz
WDW                 EM
SSB      0
LB                1.00 Hz
GB       0
PC                1.00
```
