# Supplementary material for: Modular Synthesis of α,α-Diaryl α-Amino Esters via Bi(V)-Mediated Arylation/SN2-Displacement of Kukhtin–Ramirez Intermediates
Source: Org Lett. 2022 Oct 24;24(43):8002–7. doi: 10.1021/acs.orglett.2c03201 (PMC9641671; doi:10.1021/acs.orglett.2c03201)
Supplement: Supplementary file 7 — ol2c03201_si_007.zip [file ol2c03201_si_007.zip › FID_28-32/31/31_HSQC/pdata/1/pcxac8.AC304_productdry_5_1.pdf]

Multiplicity-edited HSQC Spectrum: CH and Me up (black), CH2 down (grey).

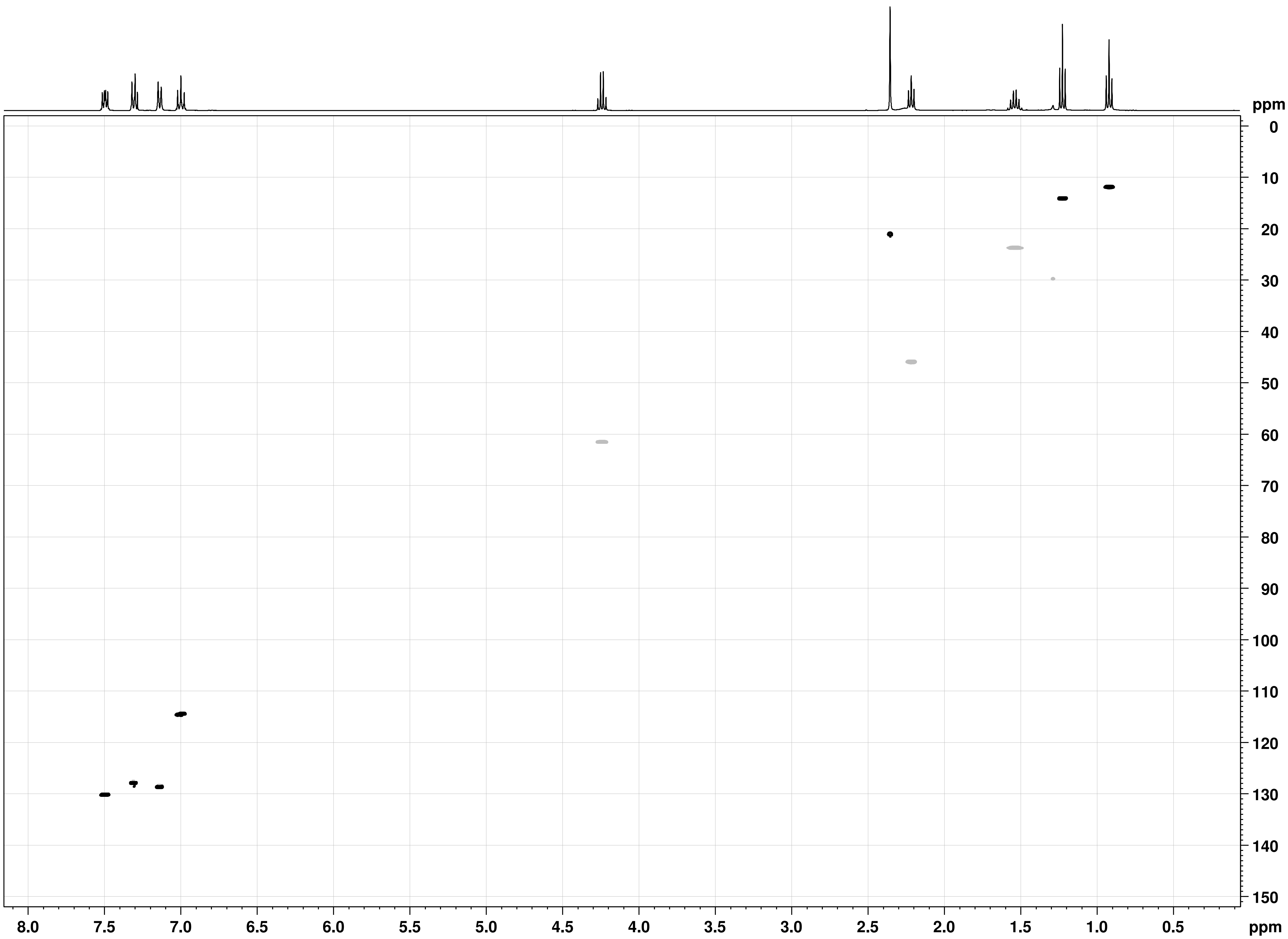


```
Current Data Parameters
NAME      pcxac8.AC304_productdry
EXPNO                   5
PROCNO                  1

F2 - Acquisition Parameters
Date_          20220318
Time              23.20 h
INSTRUM          av3400
PROBHD   Z104450_0225 (
PULPROG  hsqcedetgpsp.3
TD                 1190
SOLVENT            CDCl3
NS                    4
DS                   32
SWH            3401.361 Hz
FIDRES         5.716572 Hz
AQ            0.1749300 sec
RG                 2050
DW              147.000 usec
DE                 6.50 usec
TE                298.0 K
CNST2       145.0000000
D0           0.00000300 sec
D1           1.00000000 sec
D4           0.00172414 sec
D11          0.03000000 sec
D16          0.00020000 sec
D21          0.00360000 sec
IN0          0.00002980 sec
TDav                  1
SFO1        400.0716444 MHz
NUC1                 1H
P1                14.00 usec
P2                28.00 usec
P28             1000.00 usec
PLW1        11.92800045 W
SFO2        100.6052263 MHz
NUC2                13C
CPDPRG[2          garp4
P3                 9.75 usec
P14              500.00 usec
P31             2119.00 usec
PCPD2             80.00 usec
PLW0     0 W
PLW2        63.68099976 W
PLW12        0.94588000 W
SPNAM[3] Crp60,0.5,20.1
SPOAL3            0.500
SPOFFS3  0 Hz
SPW3         9.24930000 W
SPNAM[18  Crp60_xfilt.2
SPOAL18           0.500
SPOFFS18 0 Hz
SPW18        1.78180003 W
GPNAM[1]     SMSQ10.100
GPZ1              80.00 %
GPNAM[2]     SMSQ10.100
GPZ2              20.10 %
P16             1000.00 usec

F1 - Acquisition parameters
TD                  512
SFO1           100.6052 MHz
FIDRES        65.541107 Hz
SW              166.776 ppm
FnMODE    Echo-Antiecho

F2 - Processing parameters
SI                 2048
SF          400.0700000 MHz
WDW               QSINE
SSB                   2
LB       0 Hz
GB       0
PC                 1.40

F1 - Processing parameters
SI                 1024
MC2        echo-antiecho
SF          100.5976815 MHz
WDW               QSINE
SSB                   2
LB       0 Hz
GB       0
```
