# Supplementary material for: Modular Synthesis of α,α-Diaryl α-Amino Esters via Bi(V)-Mediated Arylation/SN2-Displacement of Kukhtin–Ramirez Intermediates
Source: Org Lett. 2022 Oct 24;24(43):8002–7. doi: 10.1021/acs.orglett.2c03201 (PMC9641671; doi:10.1021/acs.orglett.2c03201)
Supplement: Supplementary file 7 — ol2c03201_si_007.zip [file ol2c03201_si_007.zip › FID_28-32/32/32_19F/2/pdata/1/pcxac8.AC308_productdry_2_1.pdf]

Processed with back linear prediction to eliminate baseline artefacts: broad peaks may be attenuated. Standard data in file 100002

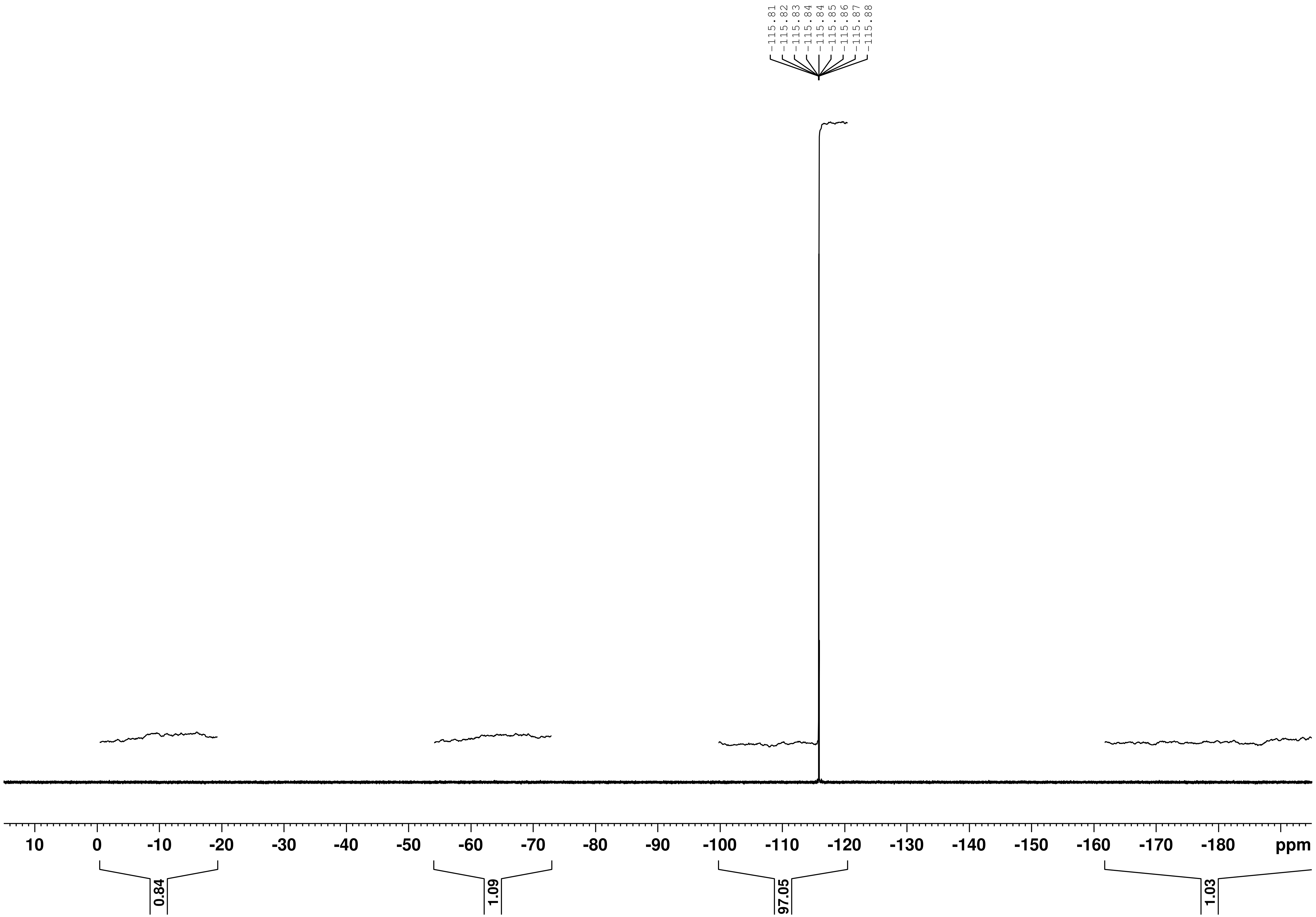

Current Data Parameters

NAME	pcxac8.AC308_productdry	
EXPNO	2	
PROCNO	1	

F2 - Acquisition Parameters

Date_	20220328	
Time	12.18	h
INSTRUM	av3400	
PROBHD	Z104450_0225 (	
PULPROG	zgflqn	
TD	261992	
SOLVENT	CDCl3	
NS	16	
DS	4	
SWH	85227.273	Hz
FIDRES	0.650610	Hz
AQ	1.5370197	sec
RG	1150	
DW	5.867	usec
DE	6.77	usec
TE	298.0	K
D1	1.50000000	sec
TD0	1	
SFO1	376.4080300	MHz
NUC1	19F	
P1	13.30	usec
PLW1	16.11100006	W

F2 - Processing parameters

SI	262144	
SF	376.4419098	MHz
WDW	EM	
SSB	0	
LB	1.00	Hz
GB	0	
PC	1.00	
