# Supplementary material for: Modular Synthesis of α,α-Diaryl α-Amino Esters via Bi(V)-Mediated Arylation/SN2-Displacement of Kukhtin–Ramirez Intermediates
Source: Org Lett. 2022 Oct 24;24(43):8002–7. doi: 10.1021/acs.orglett.2c03201 (PMC9641671; doi:10.1021/acs.orglett.2c03201)
Supplement: Supplementary file 7 — ol2c03201_si_007.zip [file ol2c03201_si_007.zip › FID_28-32/32/32_COSY/pdata/1/pcxac8.ac308_productdry_2_1.pdf]

Double quantum filtered COSY Spectrum.

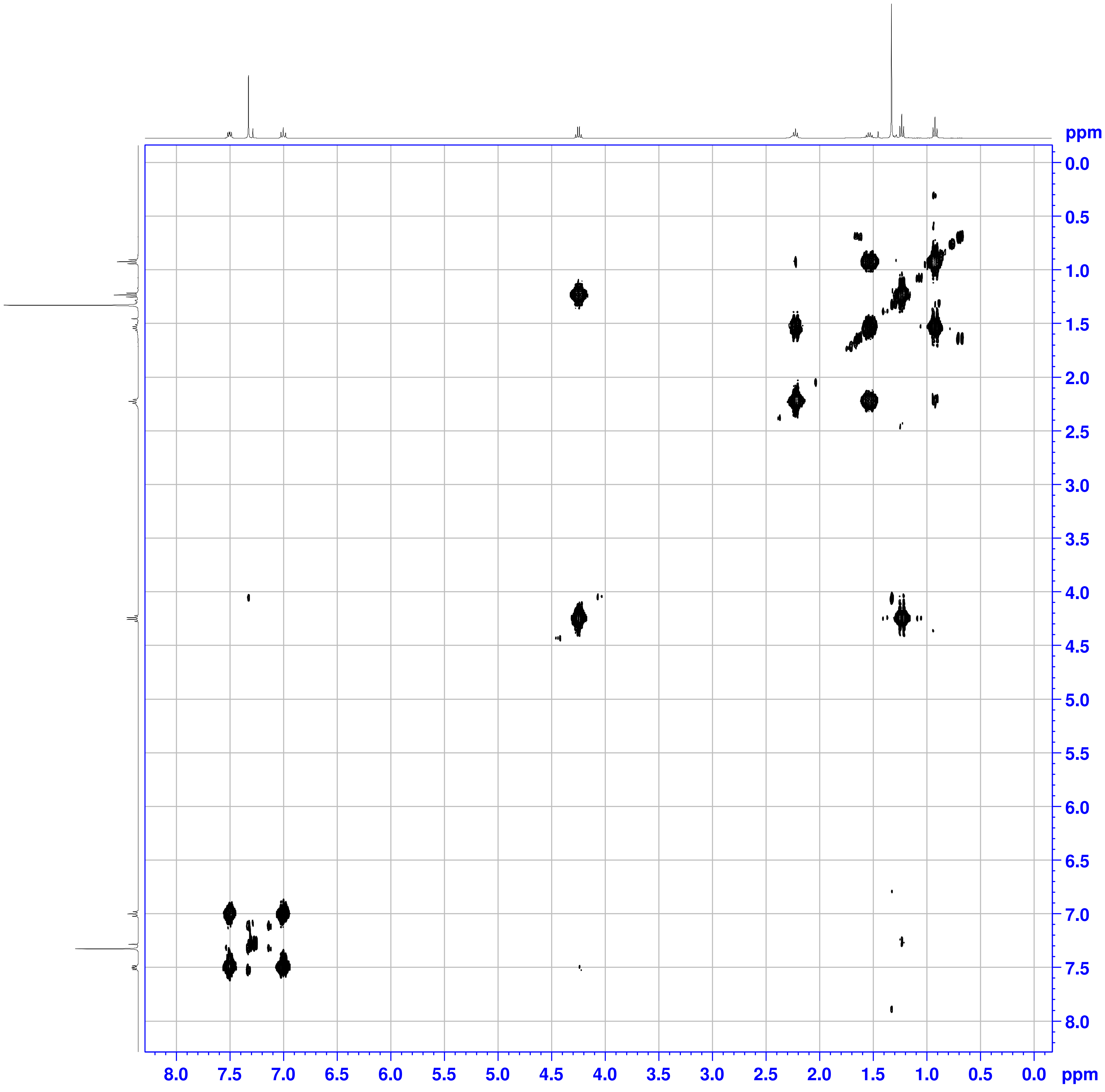


```
Current Data Parameters
NAME      pcxac8.ac308_productd
EXPNO                     2
PROCNO                    1

F2 - Acquisition Parameters
Date_              20220329
Time                  12.32 h
INSTRUM           av3400hd
PROBHD    Z122623_0053 (
PULPROG      cosygpmfppqf
TD                     2048
SOLVENT               CDCl3
NS                        2
DS                       16
SWH                3723.008 Hz
FIDRES             3.635750 Hz
AQ                0.2750464 sec
RG                   198.43
DW                  134.300 usec
DE                    10.02 usec
TE                    298.0 K
D0               0.00000300 sec
D1               0.92326808 sec
D11              0.03000000 sec
D12              0.00002000 sec
D13              0.00000400 sec
D16              0.00020000 sec
IN0              0.00026880 sec
TDav                      1
SFO1            400.2016257 MHz
NUC1                     1H
P1                    10.22 usec
P17                 2500.00 usec
PLW1            10.00000000 W
PLW10            1.16050005 W
GPNAM[1]        SMSQ10.100
GPZ1                  16.00 %
GPNAM[2]        SMSQ10.100
GPZ2                  12.00 %
GPNAM[3]        SMSQ10.100
GPZ3                  40.00 %
P16                 1000.00 usec

F1 - Acquisition parameters
TD                      128
SFO1               400.2016 MHz
FIDRES            58.128719 Hz
SW                    9.296 ppm
FnMODE                   QF

F2 - Processing parameters
SI                     2048
SF              400.2000000 MHz
WDW                    SINE
SSB       0
LB        0 Hz
GB        0
PC                     1.40

F1 - Processing parameters
SI                      512
MC2                      QF
SF              400.2000000 MHz
WDW                    SINE
SSB       0
LB        0 Hz
GB        0
```
