# Supplementary material for: Modular Synthesis of α,α-Diaryl α-Amino Esters via Bi(V)-Mediated Arylation/SN2-Displacement of Kukhtin–Ramirez Intermediates
Source: Org Lett. 2022 Oct 24;24(43):8002–7. doi: 10.1021/acs.orglett.2c03201 (PMC9641671; doi:10.1021/acs.orglett.2c03201)
Supplement: Supplementary file 7 — ol2c03201_si_007.zip [file ol2c03201_si_007.zip › FID_28-32/32/32_HSQC/pdata/1/pcxac8.ac308_productdry_5_1.pdf]

Multiplicity-edited HSQC Spectrum: CH and Me up (black), CH2 down (grey).

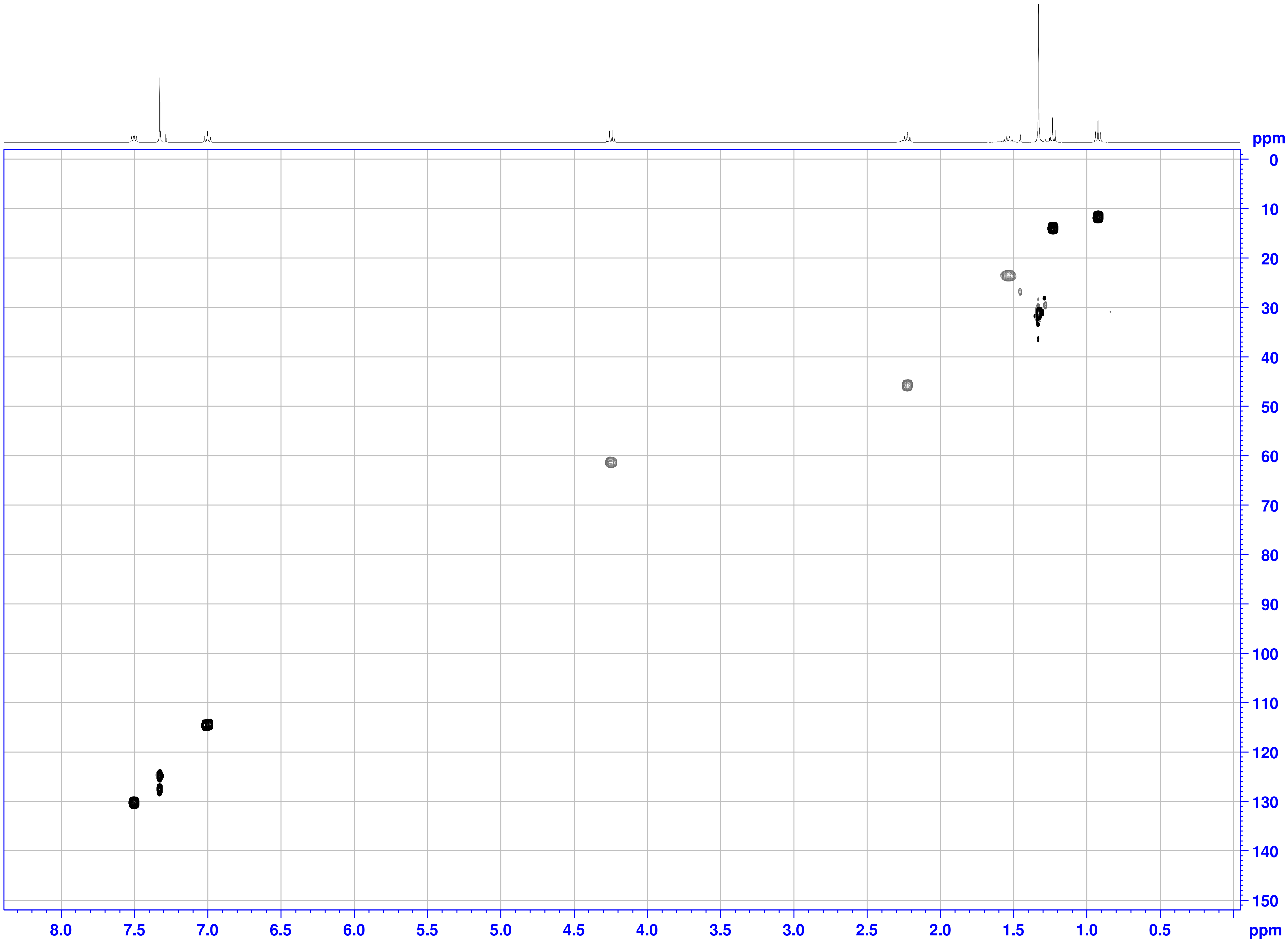


```
Current Data Parameters
NAME      pcxac8.ac308_productdry
EXPNO                   5
PROCNO                  1

F2 - Acquisition Parameters
Date_            20220330
Time                10.39 h
INSTRUM          av3400hd
PROBHD    Z122623_0053 (
PULPROG   hsqcedetgpsp.3
TD                   1190
SOLVENT             CDCl3
NS                      2
DS                     32
SWH              3546.099 Hz
FIDRES           5.959831 Hz
AQ              0.1677900 sec
RG                 198.43
DW                141.000 usec
DE                  10.00 usec
TE                  298.0 K
CNST2         145.0000000
D0             0.00000300 sec
D1             1.00000000 sec
D4             0.00172414 sec
D11            0.03000000 sec
D16            0.00020000 sec
D21            0.00360000 sec
IN0            0.00002980 sec
TDav                    1
SFO1          400.2016695 MHz
NUC1                   1H
P1                  10.22 usec
P2                  20.44 usec
P28       0 usec
PLW1          10.00000000 W
SFO2          100.6379173 MHz
NUC2                  13C
CPDPRG[2            garp4
P3                  10.70 usec
P14                500.00 usec
P31               2119.00 usec
PCPD2               80.00 usec
PLW0      0 W
PLW2          38.00000000 W
PLW12          0.67978001 W
SPNAM[3] Crp60,0.5,20.1
SPOAL3              0.500
SPOFFS3   0 Hz
SPW3           6.64729977 W
SPNAM[18  Crp60_xfilt.2
SPOAL18             0.500
SPOFFS18 0 Hz
SPW18          1.28059995 W
GPNAM[1]      SMSQ10.100
GPZ1                80.00 %
GPNAM[2]      SMSQ10.100
GPZ2                20.10 %
P16               1000.00 usec

F1 - Acquisition parameters
TD                    128
SFO1             100.6379 MHz
FIDRES         262.164429 Hz
SW                166.722 ppm
FnMODE    Echo-Antiecho

F2 - Processing parameters
SI                   2048
SF            400.2000000 MHz
WDW                 QSINE
SSB                     2
LB        0 Hz
GB        0
PC                   1.40

F1 - Processing parameters
SI                    512
MC2        echo-antiecho
SF            100.6303700 MHz
WDW                 QSINE
SSB                     2
LB        0 Hz
GB        0
```
