# Supplementary material for: Modular Synthesis of α,α-Diaryl α-Amino Esters via Bi(V)-Mediated Arylation/SN2-Displacement of Kukhtin–Ramirez Intermediates
Source: Org Lett. 2022 Oct 24;24(43):8002–7. doi: 10.1021/acs.orglett.2c03201 (PMC9641671; doi:10.1021/acs.orglett.2c03201)
Supplement: Supplementary file 8 — ol2c03201_si_008.zip [file ol2c03201_si_008.zip › FID_33-38/36/36_19F/4/pdata/1/pcxac8.AC300_productdry_4_1.pdf]

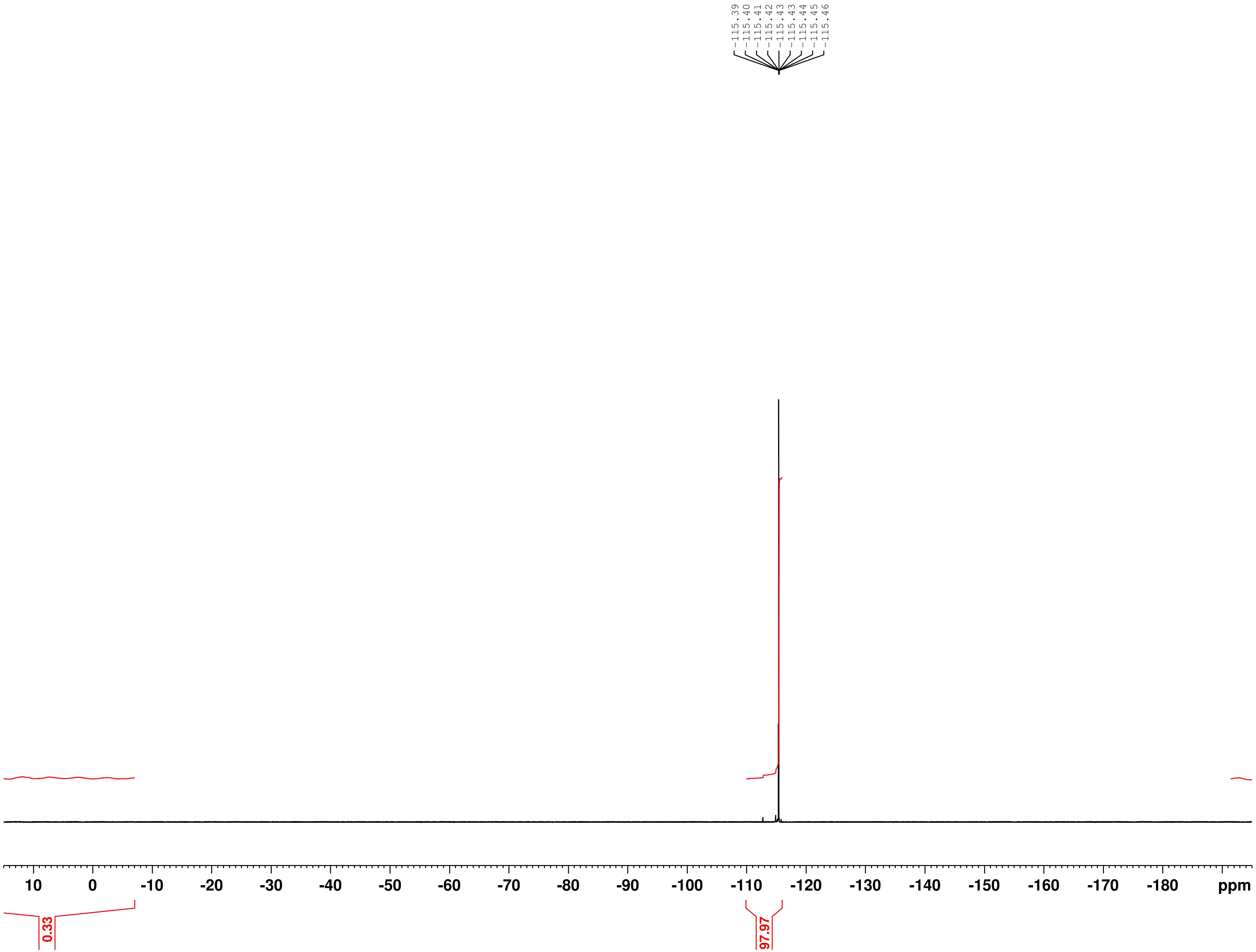


```
Current Data Parameters
NAME      pcxac8.AC300_productdry
EXPNO                       4
PROCNO                      1

F2 - Acquisition Parameters
Date_              20220321
Time                  22.03 h
INSTRUM            av3400hd
PROBHD   Z122623_0053 (
PULPROG              zgflqn
TD                   261896
SOLVENT               CDCl3
NS                       16
DS                        4
SWH              85227.273 Hz
FIDRES            0.650848 Hz
AQ               1.5364566 sec
RG                   12.56
DW                   5.867 usec
DE                   18.56 usec
TE                   298.0 K
D1              1.50000000 sec
TD0                      1
SFO1          376.5303412 MHz
NUC1                   19F
P1                   13.80 usec
PLW1            8.69999981 W

F2 - Processing parameters
SI                   262144
SF            376.5642320 MHz
WDW                      EM
SSB      0
LB                    1.00 Hz
GB       0
PC                    1.00
```
