# Supplementary material for: Modular Synthesis of α,α-Diaryl α-Amino Esters via Bi(V)-Mediated Arylation/SN2-Displacement of Kukhtin–Ramirez Intermediates
Source: Org Lett. 2022 Oct 24;24(43):8002–7. doi: 10.1021/acs.orglett.2c03201 (PMC9641671; doi:10.1021/acs.orglett.2c03201)
Supplement: Supplementary file 8 — ol2c03201_si_008.zip [file ol2c03201_si_008.zip › FID_33-38/36/36_HSQC/pdata/1/pcxac8.AC300_productdry_5_1.pdf]

Multiplicity-edited HSQC Spectrum: CH and Me up (black), CH2 down (grey).

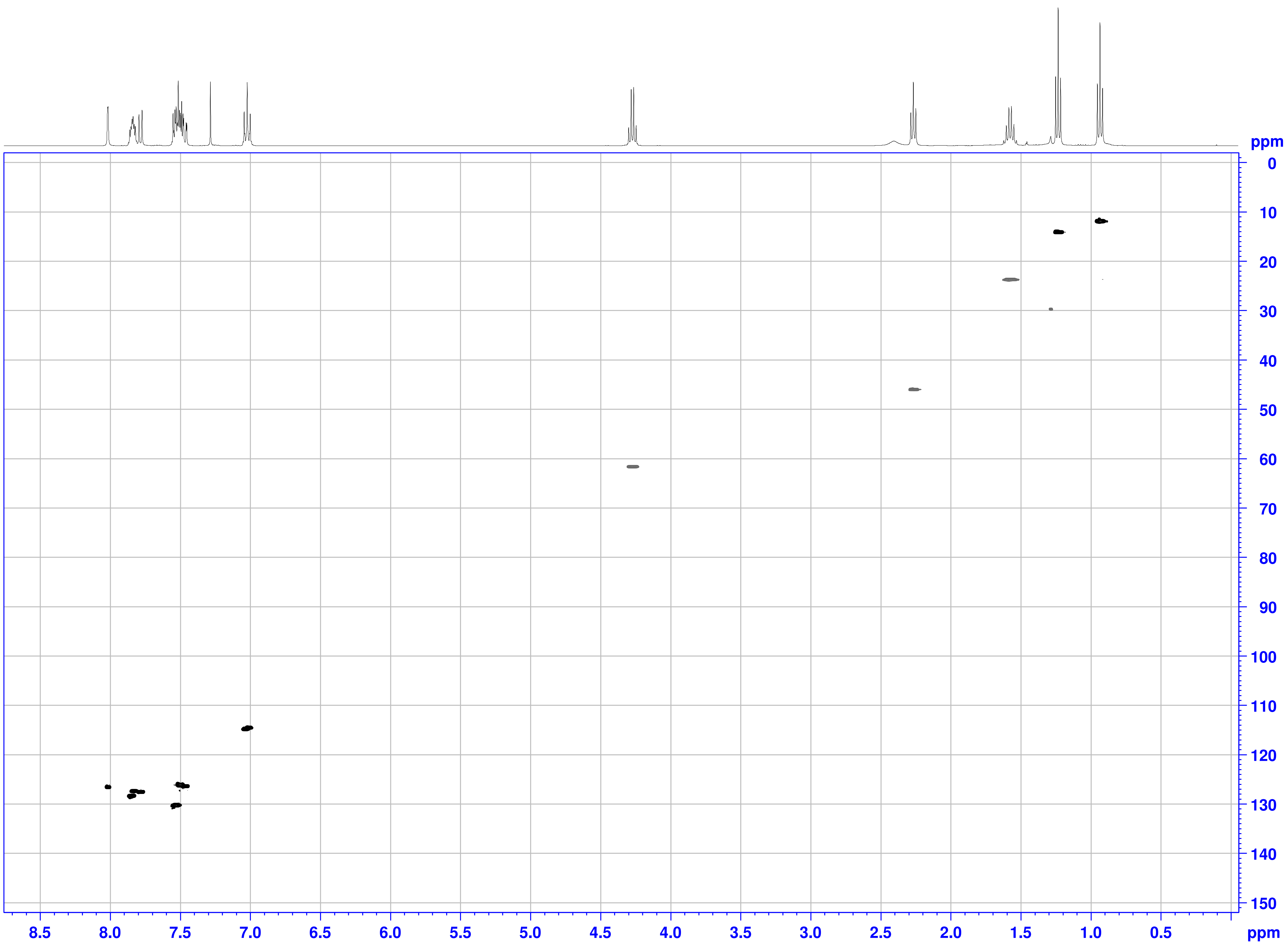


```
Current Data Parameters
NAME      pcxac8.AC300_productdry
EXPNO                       5
PROCNO                      1

F2 - Acquisition Parameters
Date_           20220321
Time               22.05 h
INSTRUM         av3400hd
PROBHD    Z122623_0053 (
PULPROG   hsqcedetgpsp.3
TD                  1190
SOLVENT            CDCl3
NS                     4
DS                    32
SWH             3703.704 Hz
FIDRES          6.224712 Hz
AQ             0.1606500 sec
RG                198.43
DW               135.000 usec
DE                 10.00 usec
TE                 298.0 K
CNST2        145.0000000
D0            0.00000300 sec
D1            1.00000000 sec
D4            0.00172414 sec
D11           0.03000000 sec
D16           0.00020000 sec
D21           0.00360000 sec
IN0           0.00002980 sec
TDav                   1
SFO1         400.2017420 MHz
NUC1                  1H
P1                 10.22 usec
P2                 20.44 usec
P28       0 usec
PLW1         10.00000000 W
SFO2         100.6379173 MHz
NUC2                 13C
CPDPRG[2           garp4
P3                 10.70 usec
P14               500.00 usec
P31              2119.00 usec
PCPD2              80.00 usec
PLW0      0 W
PLW2         38.00000000 W
PLW12         0.67978001 W
SPNAM[3] Crp60,0.5,20.1
SPOAL3             0.500
SPOFFS3   0 Hz
SPW3          6.64729977 W
SPNAM[18  Crp60_xfilt.2
SPOAL18            0.500
SPOFFS18 0 Hz
SPW18         1.28059995 W
GPNAM[1]      SMSQ10.100
GPZ1               80.00 %
GPNAM[2]      SMSQ10.100
GPZ2               20.10 %
P16              1000.00 usec

F1 - Acquisition parameters
TD                   512
SFO1            100.6379 MHz
FIDRES         65.541107 Hz
SW               166.722 ppm
FnMODE     Echo-Antiecho

F2 - Processing parameters
SI                  2048
SF           400.2000000 MHz
WDW                QSINE
SSB                    2
LB        0 Hz
GB        0
PC                  1.40

F1 - Processing parameters
SI                  1024
MC2        echo-antiecho
SF           100.6303700 MHz
WDW                QSINE
SSB                    2
LB        0 Hz
GB        0
```
