# Supplementary material for: Modular Synthesis of α,α-Diaryl α-Amino Esters via Bi(V)-Mediated Arylation/SN2-Displacement of Kukhtin–Ramirez Intermediates
Source: Org Lett. 2022 Oct 24;24(43):8002–7. doi: 10.1021/acs.orglett.2c03201 (PMC9641671; doi:10.1021/acs.orglett.2c03201)
Supplement: Supplementary file 8 — ol2c03201_si_008.zip [file ol2c03201_si_008.zip › FID_33-38/37/37_13C/1/pdata/1/email_pcxac8.AC345_13C_1_1.pdf]

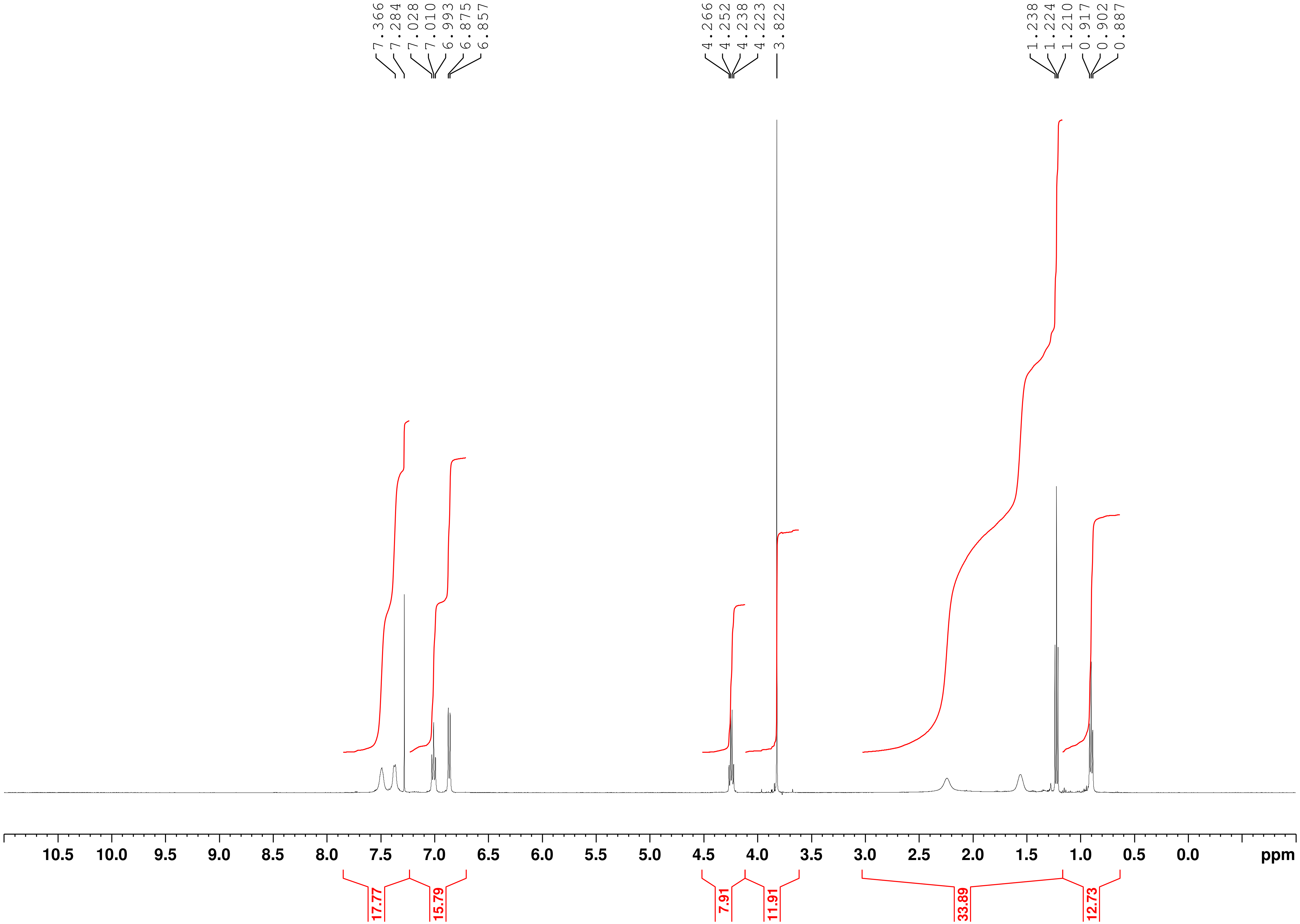

Current Data Parameters
NAME pcxac8.AC345_13C
EXPNO 1
PROCNO 1

F2 - Acquisition Parameters
Date_ 20220512
Time 15.57 h
INSTRUM av3500
PROBHD Z8007_0120 (PH
PULPROG zg30
TD 65536
SOLVENT CDCl3
NS 16
DS 2
SWH 10288.065 Hz
FIDRES 0.313967 Hz
AQ 3.1850495 sec
RG 228
DW 48.600 usec
DE 7.20 usec
TE 298.0 K
D1 1.00000000 sec
TD0 1
SFO1 500.1330885 MHz
NUC1 1H
P1 10.00 usec
PLW1 29.99200058 W

F2 - Processing parameters
SI 65536
SF 500.1300000 MHz
WDW EM
SSB 0
LB 0.30 Hz
GB 0
PC 1.00
