# Supplementary material for: Modular Synthesis of α,α-Diaryl α-Amino Esters via Bi(V)-Mediated Arylation/SN2-Displacement of Kukhtin–Ramirez Intermediates
Source: Org Lett. 2022 Oct 24;24(43):8002–7. doi: 10.1021/acs.orglett.2c03201 (PMC9641671; doi:10.1021/acs.orglett.2c03201)
Supplement: Supplementary file 8 — ol2c03201_si_008.zip [file ol2c03201_si_008.zip › FID_33-38/37/37_13C/2/pdata/1/email_pcxac8.AC345_13C_2_1.pdf]

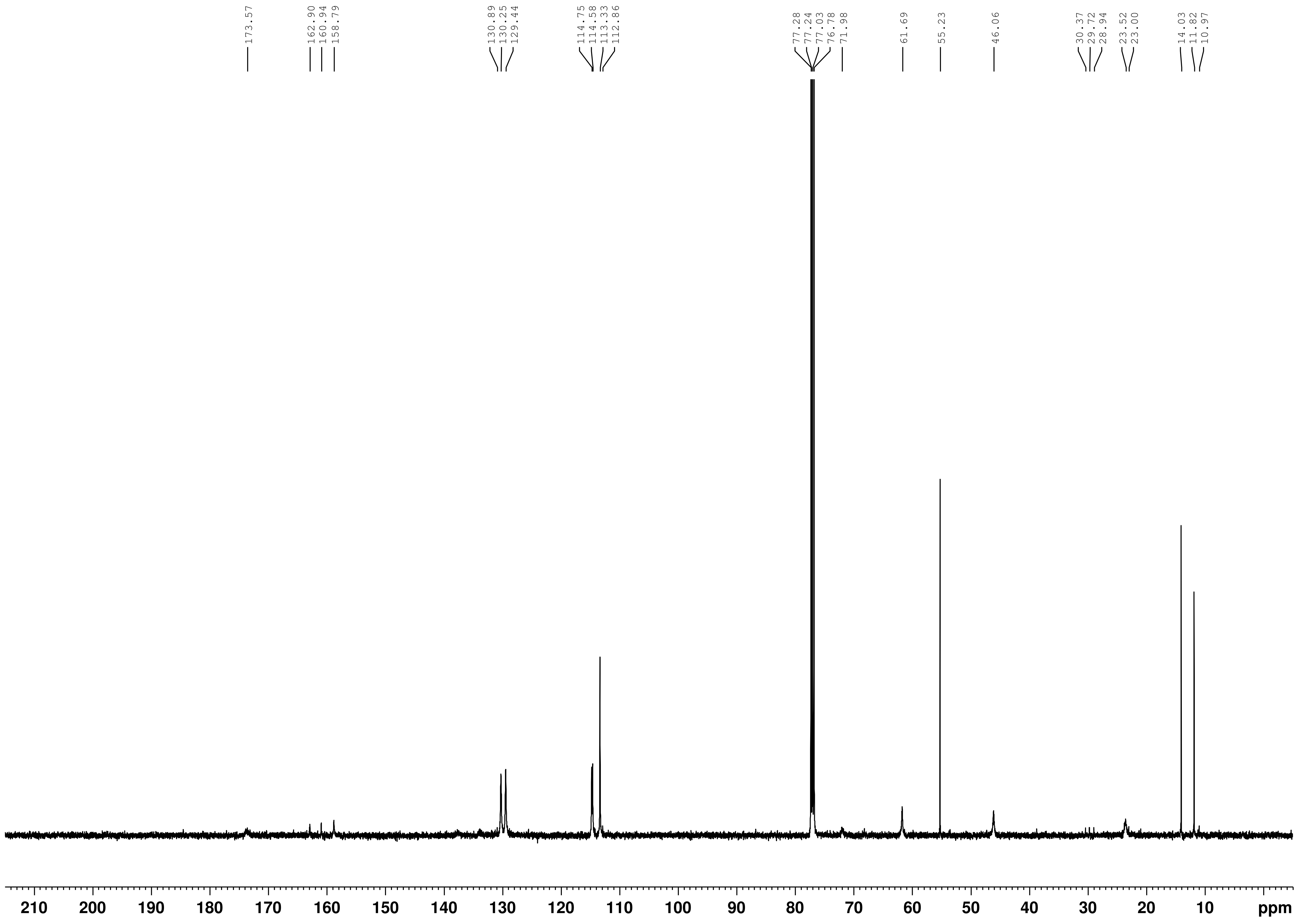

Current Data Parameters
NAME pcxac8.AC345_13C
EXPNO 2
PROCNO 1

F2 - Acquisition Parameters

Date_	20220512	
Time	19.34	h
INSTRUM	av3500	
PROBHD	Z8007_0120 (PH	
PULPROG	zgpg30	
TD	32768	
SOLVENT	CDCl3	
NS	8000	
DS	32	
SWH	29761.904	Hz
FIDRES	1.816522	Hz
AQ	0.5505024	sec
RG	2050	
DW	16.800	usec
DE	9.58	usec
TE	298.0	K
D1	1.00000000	sec
D11	0.03000000	sec
TD0	1	
SFO1	125.7716219	MHz
NUC1	13C	
P1	9.25	usec
PLW1	59.84099960	W
SFO2	500.1320005	MHz
NUC2	1H	
CPDPRG[2	waltz16	
PCPD2	80.00	usec
PLW2	29.99200058	W
PLW12	0.46862000	W
PLW13	0.23571000	W

F2 - Processing parameters

SI	32768	
SF	125.7577885	MHz
WDW	EM	
SSB	0	
LB	1.00	Hz
GB	0	
PC	1.40	
