# Supplementary material for: Modular Synthesis of α,α-Diaryl α-Amino Esters via Bi(V)-Mediated Arylation/SN2-Displacement of Kukhtin–Ramirez Intermediates
Source: Org Lett. 2022 Oct 24;24(43):8002–7. doi: 10.1021/acs.orglett.2c03201 (PMC9641671; doi:10.1021/acs.orglett.2c03201)
Supplement: Supplementary file 8 — ol2c03201_si_008.zip [file ol2c03201_si_008.zip › FID_33-38/37/37_13C/3/pdata/1/email_pcxac8.AC345_13C_3_1.pdf]

Double quantum filtered COSY Spectrum.

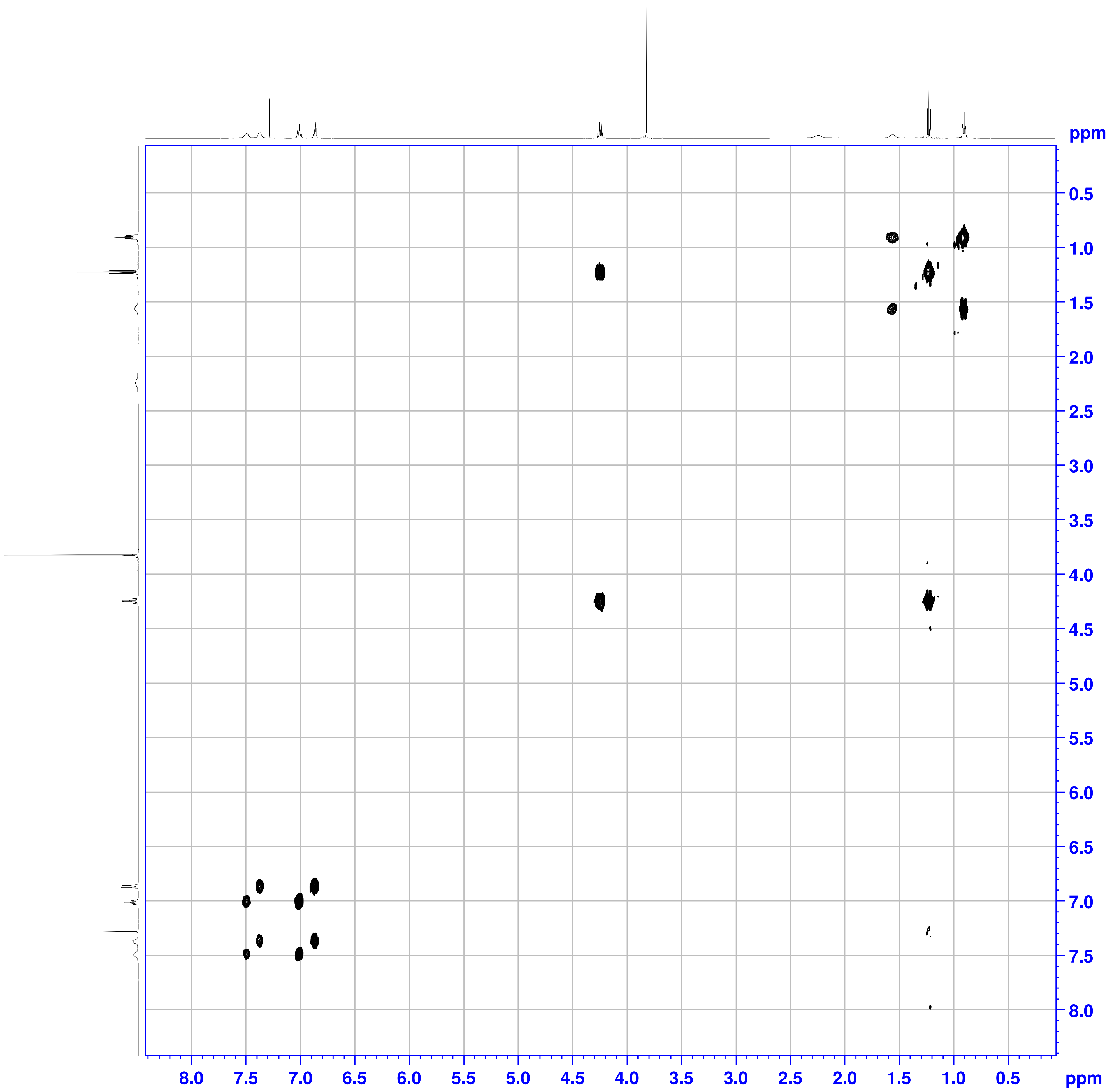


```
Current Data Parameters
NAME        pcxac8.AC345_13C
EXPNO                      3
PROCNO                     1

F2 - Acquisition Parameters
Date_               20220512
Time                   19.35 h
INSTRUM               av3500
PROBHD     Z8007_0120 (PH
PULPROG        cosygpmfppqf
TD                      2048
SOLVENT                CDCl3
NS                         2
DS                        16
SWH                 4601.227 Hz
FIDRES              4.493386 Hz
AQ                 0.2225493 sec
RG                      2050
DW                   108.667 usec
DE                      7.00 usec
TE                     298.0 K
D0                0.00000300 sec
D1                0.93569273 sec
D11               0.03000000 sec
D12               0.00002000 sec
D13               0.00000400 sec
D16               0.00020000 sec
IN0               0.00021740 sec
TDav                       1
SFO1             500.1321220 MHz
NUC1                      1H
P1                     10.00 usec
P17                  2500.00 usec
PLW1             29.99200058 W
PLW10             3.33240008 W
GPNAM[1]         SMSQ10.100
GPZ1                   16.00 %
GPNAM[2]         SMSQ10.100
GPZ2                   12.00 %
GPNAM[3]         SMSQ10.100
GPZ3                   40.00 %
P16                  1000.00 usec

F1 - Acquisition parameters
TD                       128
SFO1                500.1321 MHz
FIDRES             71.872124 Hz
SW                     9.197 ppm
FnMODE                    QF

F2 - Processing parameters
SI                      2048
SF               500.1300000 MHz
WDW                     SINE
SSB       0
LB        0 Hz
GB        0
PC                      1.40

F1 - Processing parameters
SI                       512
MC2                       QF
SF               500.1300000 MHz
WDW                     SINE
SSB       0
LB        0 Hz
GB        0
```
