# Supplementary material for: Modular Synthesis of α,α-Diaryl α-Amino Esters via Bi(V)-Mediated Arylation/SN2-Displacement of Kukhtin–Ramirez Intermediates
Source: Org Lett. 2022 Oct 24;24(43):8002–7. doi: 10.1021/acs.orglett.2c03201 (PMC9641671; doi:10.1021/acs.orglett.2c03201)
Supplement: Supplementary file 8 — ol2c03201_si_008.zip [file ol2c03201_si_008.zip › FID_33-38/37/37_13C/4/pdata/1/email_pcxac8.AC345_13C_4_1.pdf]

Multiplicity-edited HSQC Spectrum: CH and Me up (black), CH2 down (grey).

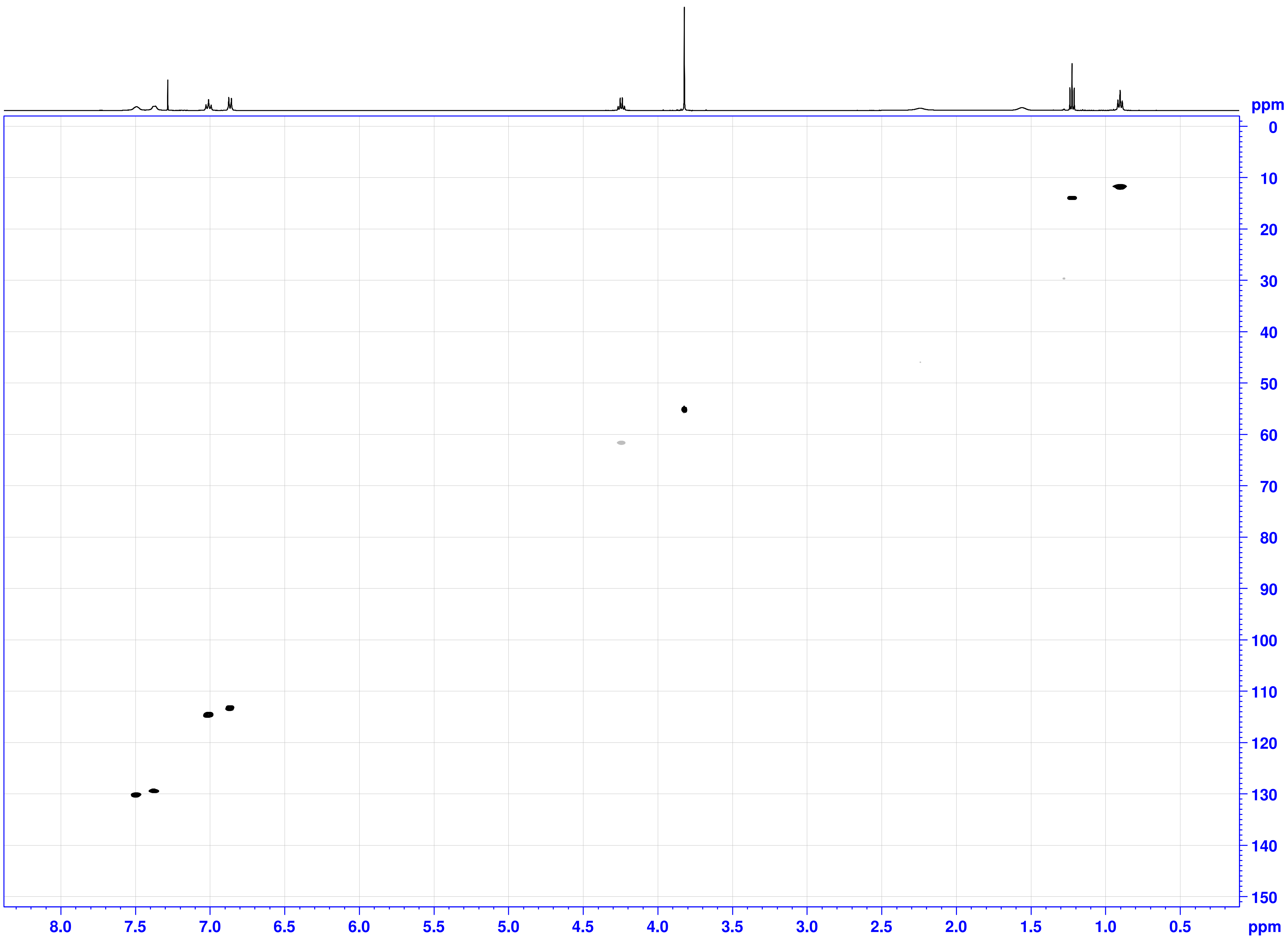


```
Current Data Parameters
NAME       pcxac8.AC345_13C
EXPNO                     4
PROCNO                    1

F2 - Acquisition Parameters
Date_              20220512
Time                  19.41 h
INSTRUM               av3500
PROBHD     Z8007_0120 (PH
PULPROG    hsqcedetgpsp.3
TD                     1190
SOLVENT               CDCl3
NS                        8
DS                       32
SWH                4347.826 Hz
FIDRES             7.307271 Hz
AQ                0.1368500 sec
RG                     2050
DW                  115.000 usec
DE                     6.50 usec
TE                    298.0 K
CNST2           145.0000000
D0               0.00000300 sec
D1               1.00000000 sec
D4               0.00172414 sec
D11              0.03000000 sec
D16              0.00020000 sec
D21              0.00360000 sec
IN0              0.00002380 sec
TDav                      1
SFO1            500.1321220 MHz
NUC1                     1H
P1                    10.00 usec
P2                    20.00 usec
P28                 1000.00 usec
PLW1            29.99200058 W
SFO2            125.7672204 MHz
NUC2                    13C
CPDPRG[2              garp4
P3                     9.25 usec
P14                  500.00 usec
P31                 1895.00 usec
PCPD2                 81.24 usec
PLW0       0 W
PLW2            59.84099960 W
PLW12            0.77578998 W
SPNAM[3]  Crp60,0.5,20.1
SPOAL3                0.500
SPOFFS3    0 Hz
SPW3             7.82299995 W
SPNAM[18  Crp60_xfilt.2
SPOAL18               0.500
SPOFFS18   0 Hz
SPW18            1.88440001 W
GPNAM[1]        SMSQ10.100
GPZ1                  80.00 %
GPNAM[2]        SMSQ10.100
GPZ2                  20.10 %
P16                 1000.00 usec

F1 - Acquisition parameters
TD                      512
SFO1               125.7672 MHz
FIDRES            82.064079 Hz
SW                  167.042 ppm
FnMODE         Echo-Antiecho

F2 - Processing parameters
SI                     2048
SF              500.1300000 MHz
WDW                   QSINE
SSB                       2
LB         0 Hz
GB         0
PC                     1.40

F1 - Processing parameters
SI                      512
MC2           echo-antiecho
SF              125.7577885 MHz
WDW                   QSINE
SSB                       2
LB         0 Hz
GB         0
```
