# Supplementary material for: Modular Synthesis of α,α-Diaryl α-Amino Esters via Bi(V)-Mediated Arylation/SN2-Displacement of Kukhtin–Ramirez Intermediates
Source: Org Lett. 2022 Oct 24;24(43):8002–7. doi: 10.1021/acs.orglett.2c03201 (PMC9641671; doi:10.1021/acs.orglett.2c03201)
Supplement: Supplementary file 8 — ol2c03201_si_008.zip [file ol2c03201_si_008.zip › FID_33-38/37/37_19F/2/pdata/1/pcxac8.AC345_column_2_1.pdf]

Processed with back linear prediction to eliminate baseline artefacts: broad peaks may be attenuated. Standard data in file 100002

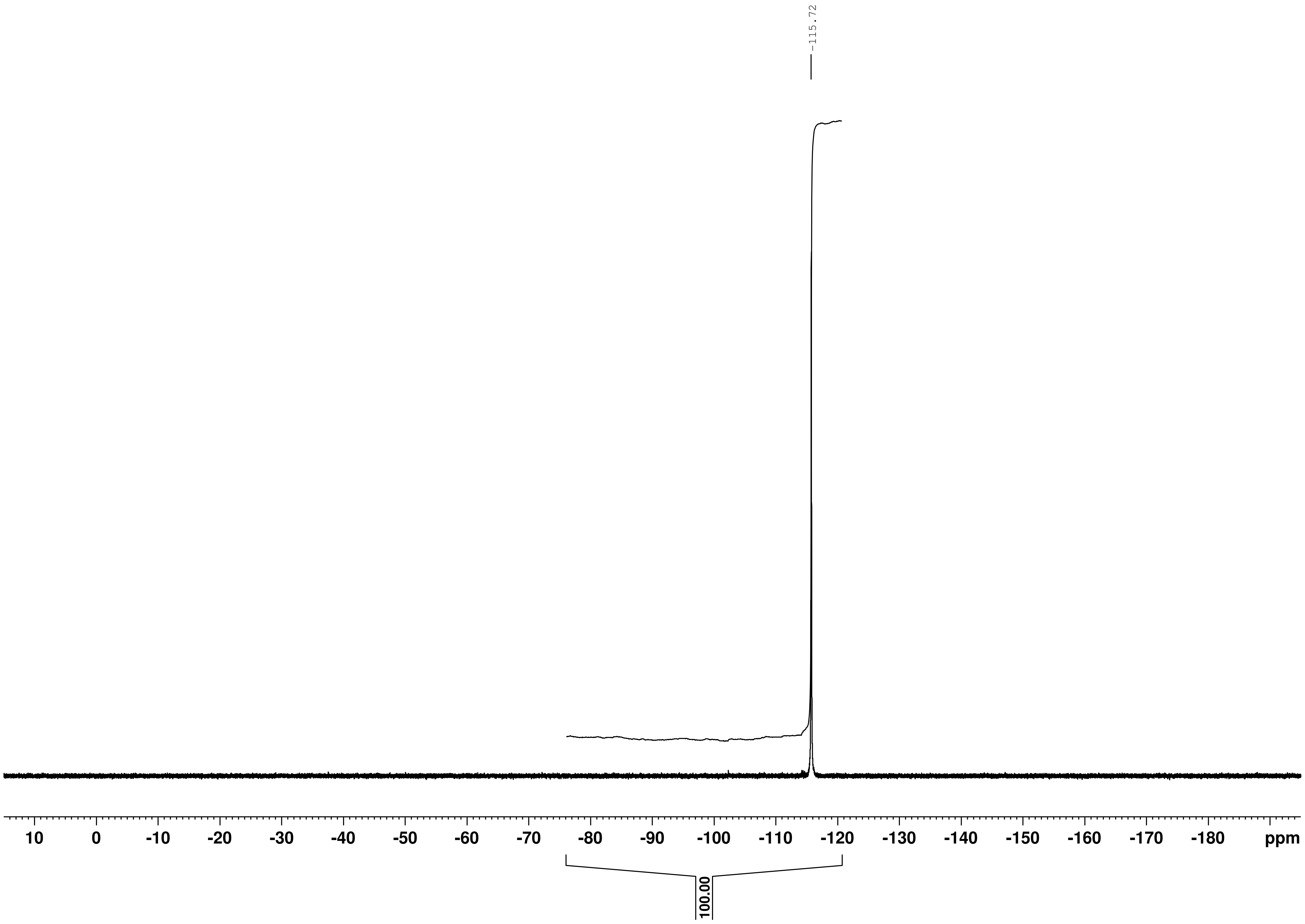

Current Data Parameters

NAME	pcxac8.AC345_column
EXPNO	2
PROCNO	1

F2 - Acquisition Parameters

Date_	20220510
Time	9.41 h
INSTRUM	av3400
PROBHD	Z104450_0225 (
PULPROG	zgflqn
TD	261992
SOLVENT	CDCl3
NS	16
DS	4
SWH	85227.273 Hz
FIDRES	0.650610 Hz
AQ	1.5370197 sec
RG	1290
DW	5.867 usec
DE	6.77 usec
TE	298.0 K
D1	1.50000000 sec
TD0	1
SFO1	376.4080300 MHz
NUC1	19F
P1	13.30 usec
PLW1	16.11100006 W

F2 - Processing parameters

SI	262144
SF	376.4419098 MHz
WDW	EM
SSB	0
LB	1.00 Hz
GB	0
PC	1.00
