# Supplementary material for: Modular Synthesis of α,α-Diaryl α-Amino Esters via Bi(V)-Mediated Arylation/SN2-Displacement of Kukhtin–Ramirez Intermediates
Source: Org Lett. 2022 Oct 24;24(43):8002–7. doi: 10.1021/acs.orglett.2c03201 (PMC9641671; doi:10.1021/acs.orglett.2c03201)
Supplement: Supplementary file 8 — ol2c03201_si_008.zip [file ol2c03201_si_008.zip › FID_33-38/37/pcxac8.AC354_product/1/pdata/1/pcxac8.AC354_product_1_1.pdf]

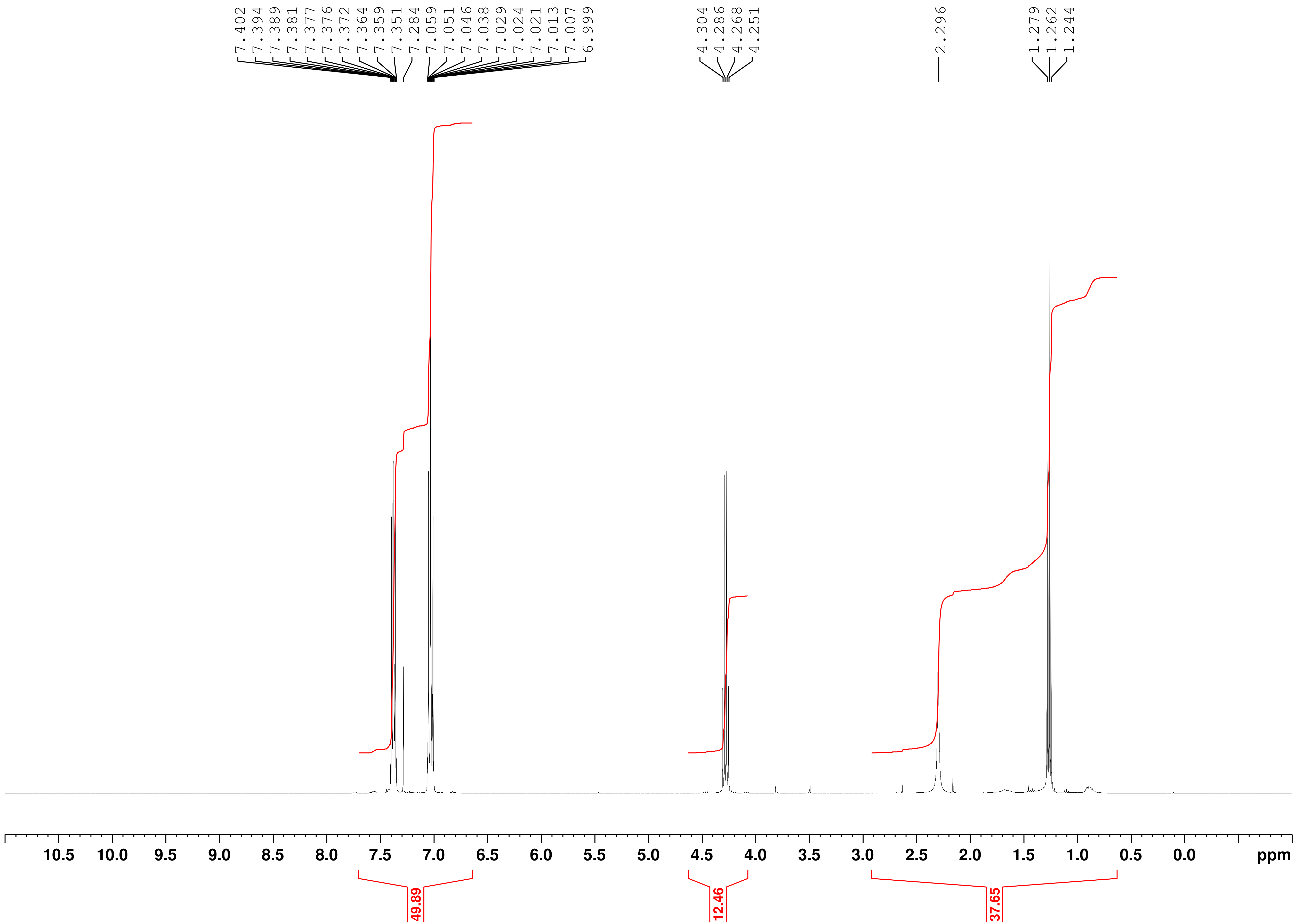


```
Current Data Parameters
NAME      pcxac8.AC354_product
EXPNO                        1
PROCNO                       1

F2 - Acquisition Parameters
Date_              20220517
Time                  19.05 h
INSTRUM              av3400
PROBHD   Z104450_0225 (
PULPROG                zg30
TD                    65536
SOLVENT               CDCl3
NS                       16
DS                        2
SWH                8223.685 Hz
FIDRES             0.250967 Hz
AQ                3.9845889 sec
RG                      181
DW                   60.800 usec
DE                    16.03 usec
TE                    298.0 K
D1               1.00000000 sec
TD0                       1
SFO1            400.0724706 MHz
NUC1                     1H
P1                    14.00 usec
PLW1            11.92800045 W

F2 - Processing parameters
SI                    65536
SF              400.0700000 MHz
WDW                      EM
SSB       0
LB                     0.30 Hz
GB        0
PC                     1.00
```
