# Supplementary material for: Modular Synthesis of α,α-Diaryl α-Amino Esters via Bi(V)-Mediated Arylation/SN2-Displacement of Kukhtin–Ramirez Intermediates
Source: Org Lett. 2022 Oct 24;24(43):8002–7. doi: 10.1021/acs.orglett.2c03201 (PMC9641671; doi:10.1021/acs.orglett.2c03201)
Supplement: Supplementary file 9 — ol2c03201_si_009.zip [file ol2c03201_si_009.zip › FID_Bi-cpds/pMeO Ar3Bi/1H/pdata/1/email_pcxac8.PMeOAr3Bi_1_1.pdf]

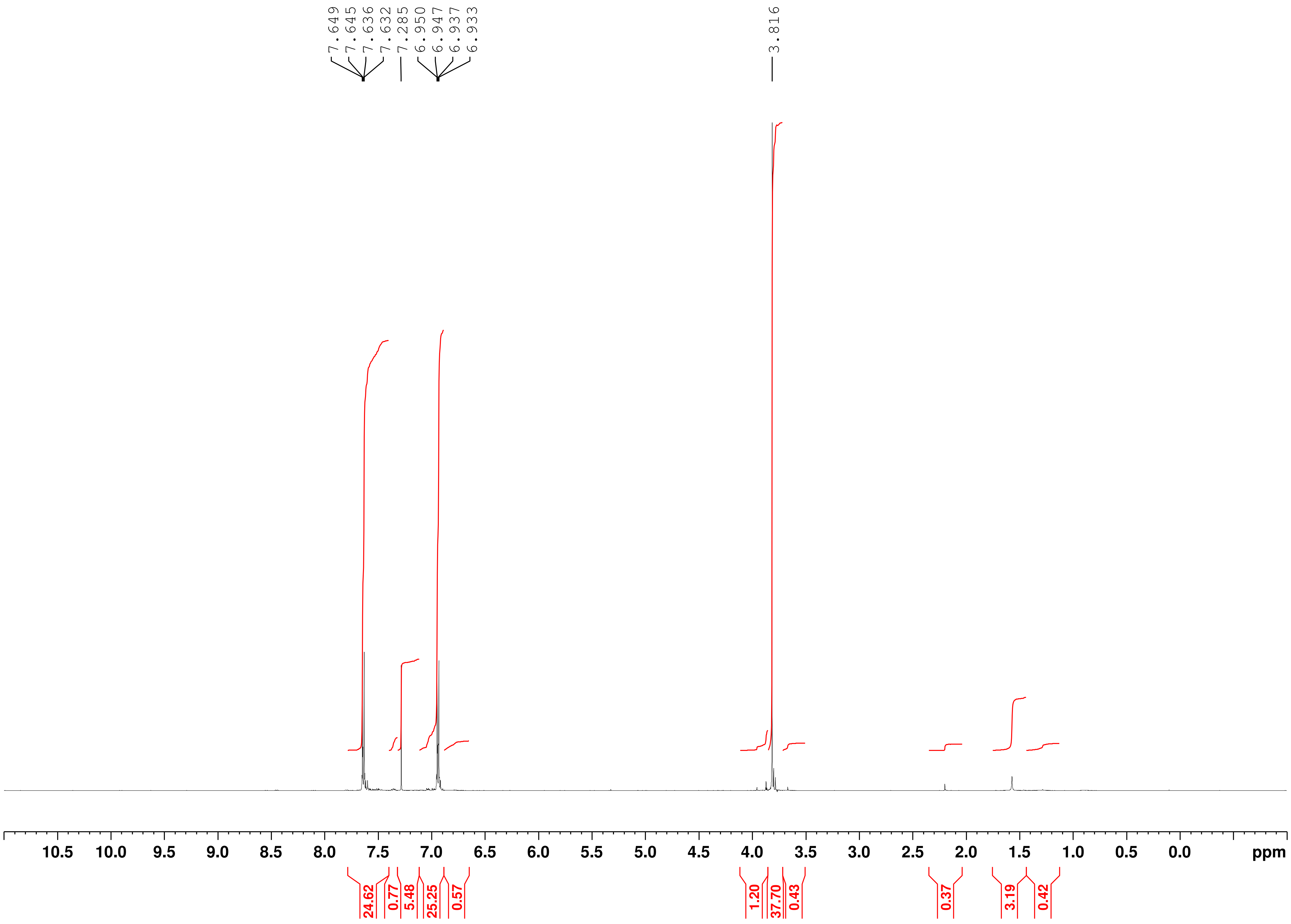


```
Current Data Parameters
NAME       pcxac8.PMeOAr3Bi
EXPNO                     1
PROCNO                    1

F2 - Acquisition Parameters
Date_              20220608
Time                  15.06 h
INSTRUM              av3500
PROBHD    Z8007_0120 (PH
PULPROG                zg30
TD                    65536
SOLVENT               CDCl3
NS                       16
DS                        2
SWH               10288.065 Hz
FIDRES             0.313967 Hz
AQ                3.1850495 sec
RG                      322
DW                   48.600 usec
DE                     7.20 usec
TE                    298.0 K
D1               1.00000000 sec
TD0                       1
SFO1            500.1330885 MHz
NUC1                     1H
P1                    10.00 usec
PLW1            29.99200058 W

F2 - Processing parameters
SI                    65536
SF              500.1300000 MHz
WDW                      EM
SSB       0
LB                     0.30 Hz
GB        0
PC                     1.00
```
