# Supplementary material for: Modular Synthesis of α,α-Diaryl α-Amino Esters via Bi(V)-Mediated Arylation/SN2-Displacement of Kukhtin–Ramirez Intermediates
Source: Org Lett. 2022 Oct 24;24(43):8002–7. doi: 10.1021/acs.orglett.2c03201 (PMC9641671; doi:10.1021/acs.orglett.2c03201)
Supplement: Supplementary file 10 — ol2c03201_si_010.zip [file ol2c03201_si_010.zip › FID keto ester/FID keto ester/1h/13C/pdata/1/pcxac8.AC227_F2_2_1.pdf]

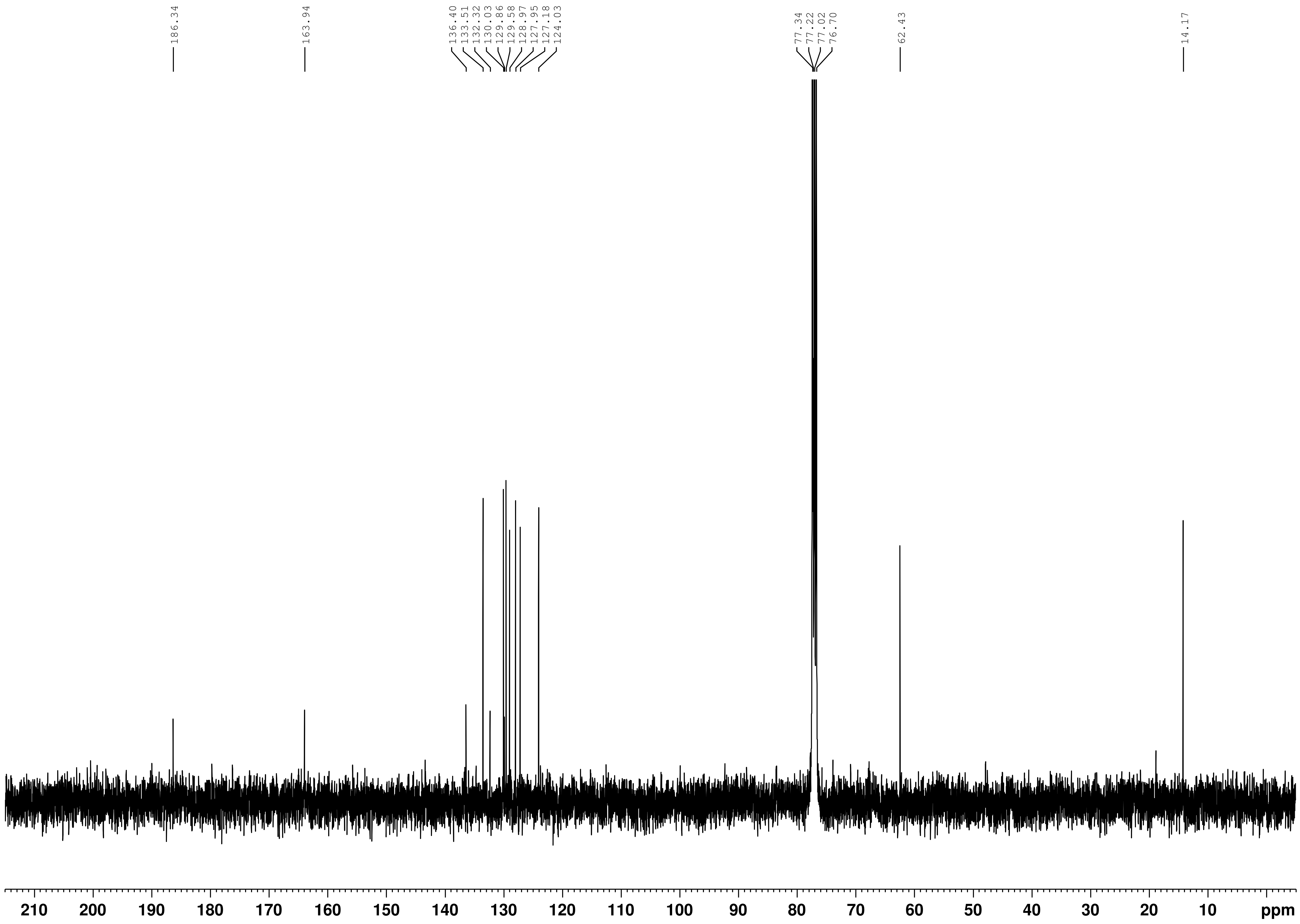


```
Current Data Parameters
NAME     pcxac8.AC227_F2
EXPNO                  2
PROCNO                 1

F2 - Acquisition Parameters
Date_           20220127
Time               22.16 h
INSTRUM         av3400hd
PROBHD   Z122623_0053 (
PULPROG           zgpg30
TD                 32768
SOLVENT            CDCl3
NS                  1024
DS                     4
SWH            23809.523 Hz
FIDRES          1.453218 Hz
AQ             0.6881280 sec
RG                 87.88
DW                21.000 usec
DE                 18.80 usec
TE                 298.0 K
D1            1.00000000 sec
D11           0.03000000 sec
TD0                    1
SFO1         100.6414394 MHz
NUC1                 13C
P1                 10.19 usec
PLW1         38.00000000 W
SFO2         400.2016008 MHz
NUC2                  1H
CPDPRG[2         waltz16
PCPD2              90.00 usec
PLW2         10.00000000 W
PLW12         0.12895000 W
PLW13         0.06486000 W

F2 - Processing parameters
SI                 32768
SF           100.6303700 MHz
WDW                   EM
SSB      0
LB                  1.00 Hz
GB       0
PC                  1.40
```
