# Supplementary material for: Modular Synthesis of α,α-Diaryl α-Amino Esters via Bi(V)-Mediated Arylation/SN2-Displacement of Kukhtin–Ramirez Intermediates
Source: Org Lett. 2022 Oct 24;24(43):8002–7. doi: 10.1021/acs.orglett.2c03201 (PMC9641671; doi:10.1021/acs.orglett.2c03201)
Supplement: Supplementary file 10 — ol2c03201_si_010.zip [file ol2c03201_si_010.zip › FID keto ester/FID keto ester/1h/COSY/pdata/1/pcxac8.AC227_F2_3_1.pdf]

Double quantum filtered COSY Spectrum.

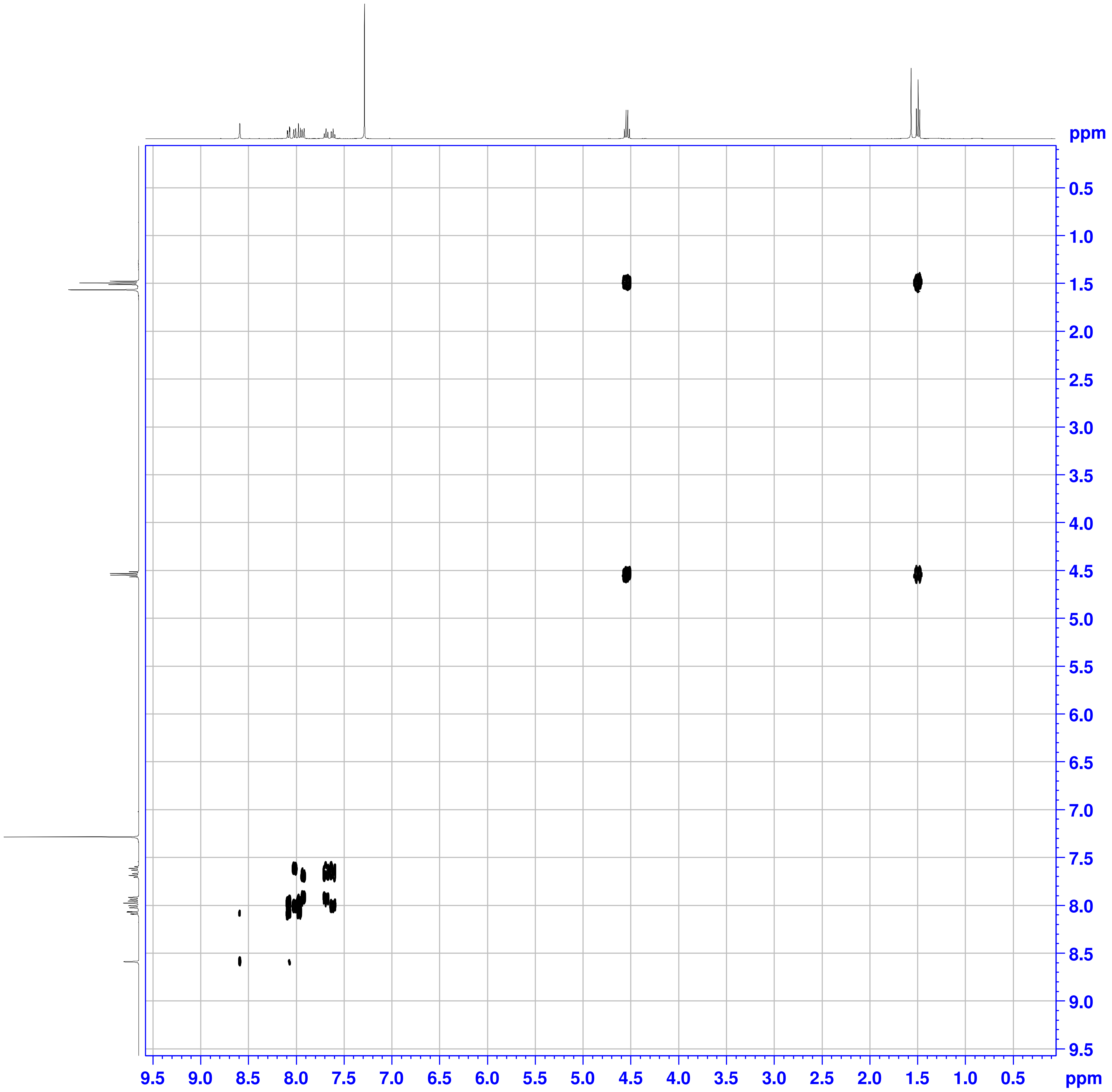


```
Current Data Parameters
NAME     pcxac8.AC227_F2
EXPNO                  3
PROCNO                 1

F2 - Acquisition Parameters
Date_           20220127
Time               22.17 h
INSTRUM          av3400hd
PROBHD   Z122623_0053 (
PULPROG      cosygpmfppqf
TD                  2048
SOLVENT            CDCl3
NS                     2
DS                    16
SWH             4170.142 Hz
FIDRES          4.072404 Hz
AQ             0.2455552 sec
RG                198.43
DW               119.900 usec
DE                 23.92 usec
TE                 298.0 K
D0            0.00000300 sec
D1            0.95603621 sec
D11           0.03000000 sec
D12           0.00002000 sec
D13           0.00000400 sec
D16           0.00020000 sec
IN0           0.00024000 sec
TDav                   1
SFO1         400.2019273 MHz
NUC1                  1H
P1                 10.22 usec
P17              2500.00 usec
PLW1         10.00000000 W
PLW10         1.16050005 W
GPNAM[1]      SMSQ10.100
GPZ1               16.00 %
GPNAM[2]      SMSQ10.100
GPZ2               12.00 %
GPNAM[3]      SMSQ10.100
GPZ3               40.00 %
P16              1000.00 usec

F1 - Acquisition parameters
TD                   128
SFO1            400.2019 MHz
FIDRES         65.104164 Hz
SW                10.411 ppm
FnMODE                QF

F2 - Processing parameters
SI                  2048
SF           400.2000000 MHz
WDW                 SINE
SSB      0
LB       0 Hz
GB       0
PC                  1.40

F1 - Processing parameters
SI                   512
MC2                   QF
SF           400.2000000 MHz
WDW                 SINE
SSB      0
LB       0 Hz
GB       0
```
